# Candidate SNP Markers Significantly Altering the Affinity of the TATA-Binding Protein for the Promoters of Human Genes Associated with Primary Open-Angle Glaucoma

**DOI:** 10.3390/ijms252312802

**Published:** 2024-11-28

**Authors:** Karina Zolotareva, Polina A. Dotsenko, Nikolay Podkolodnyy, Roman Ivanov, Aelita-Luiza Makarova, Irina Chadaeva, Anton Bogomolov, Pavel S. Demenkov, Vladimir Ivanisenko, Dmitry Oshchepkov, Mikhail Ponomarenko

**Affiliations:** 1 Institute of Cytology and Genetics, Siberian Branch, Russian Academy of Sciences (ICG SB RAS), Novosibirsk 630090, Russia; ka125699ri@yandex.ru (K.Z.); dotsenko-polina@mail.ru (P.A.D.); pnl@bionet.nsc.ru (N.P.); makarovaaa@bionet.nsc.ru (A.-L.M.); ichadaeva@bionet.nsc.ru (I.C.); mantis_anton@bionet.nsc.ru (A.B.); demps@bionet.nsc.ru (P.S.D.); salix@bionet.nsc.ru (V.I.); diman@bionet.nsc.ru (D.O.); 2 Kurchatov Genome Center at the ICG SB RAS, Novosibirsk 630090, Russia; 3 Department of Natural Sciences, Novosibirsk State University, Novosibirsk 630090, Russia; 4 Institute of Computational Mathematics and Mathematical Geophysics, SB RAS, Novosibirsk 630090, Russia

**Keywords:** human, primary open-angle glaucoma, gene, promoter, TBP, TATA box, SNP, candidate SNP marker, gene expression change, phylostratigraphic age, natural selection, in silico verification

## Abstract

Primary open-angle glaucoma (POAG) is the most common form of glaucoma. This condition leads to optic nerve degeneration and eventually to blindness. Tobacco smoking, alcohol consumption, fast-food diets, obesity, heavy weight lifting, high-intensity physical exercises, and many other bad habits are lifestyle-related risk factors for POAG. By contrast, moderate-intensity aerobic exercise and the Mediterranean diet can alleviate POAG. In this work, we for the first time estimated the phylostratigraphic age indices (PAIs) of all 153 POAG-related human genes in the NCBI Gene Database. This allowed us to separate them into two groups: POAG-related genes that appeared before and after the phylum Chordata, that is, ophthalmologically speaking, before and after the camera-type eye evolved. Next, in the POAG-related genes’ promoters, we in silico predicted all 3835 candidate SNP markers that significantly change the TATA-binding protein (TBP) affinity for these promoters and, through this molecular mechanism, the expression levels of these genes. Finally, we verified our results against five independent web services—PANTHER, DAVID, STRING, MetaScape, and GeneMANIA—as well as the ClinVar database. It was concluded that POAG is likely to be a symptom of the human self-domestication syndrome, a downside of being civilized.

## 1. Introduction

Primary open-angle glaucoma (POAG) is the most common form of glaucoma, which is optic nerve degeneration that slowly progresses over years and inevitably leads to blindness [1,2]. According to an editorial [3], an operational definition of glaucoma as a set of symptoms that can be attributed to this condition is still a source of dispute across international healthcare organizations due to non-uniform levels of healthcare for different communities worldwide. However, if access to healthcare were easier [4,5], optic nerve damage could be detected directly [6] rather than by reliance on high intraocular pressure (IOP), which is today’s practice [7,8]. The risk factors for POAG often named in the literature are hypertension [9], age-related diseases [10], genetic susceptibility, developmental disorders [11], tobacco smoking [12], alcohol consumption [13], fast-food diets [14], obesity [15], heavy weight lifting, and high-intensity physical exercise; meanwhile, the Mediterranean diet [16] and moderate-intensity aerobic exercise are mentioned as being protective against the development of this condition [17]. In addition, according to the WHO [18], the prevalence of anxiety (9.8%) and depression (5.8%) in POAG correlates with its severity. Consequently, past illnesses and bad habits may aggravate POAG, while a healthy lifestyle may alleviate it.

Moreover, POAG appears to be a genetically complex and intricate disease with risk factors that may contribute to its progression depending on both genetic and epigenetic backgrounds along with environmental factors rather than independently [19]. Nevertheless, high IOP is the only known modifiable risk factor for POAG [20] and the cornerstone of anti-POAG therapy [21]. Finally, the universal demand for research into POAG is so great that out of 9244 original POAG-related articles cited in the freely accessible PubMed database [22], 464 have been added in the year 2024 as on 31 October 2024.

In the post-genome era of life sciences, genome-wide experimental data on single-nucleotide polymorphisms (SNPs) [23] and the results of their scrutiny using Genome-Wide Association Studies (GWAS) [24] and Quantitative Trait Locus (QTL) analysis [25] have gained special importance. With the advent of new diagnostic tools, such as optical coherence tomography (OCT) and standard automated perimetry (SAP)—which are supposed to directly determine the amount of damage to the optic nerve [4,5,6]—in addition to the traditional method based on measuring IOP [7,8,20], the search for genetic and molecular-biological markers of POAG that can enhance the diagnostic abilities of high IOP as a traditional clinical marker of this disease has become a trend [26].

GWAS and QTL results allowed POAG-associated loci and the POAG-related genes within these loci to be identified on human chromosomes. The number of POAG-related genes varies from one source of information to the next, reported as 7 [27], 11 [28,29], 18 [30], 26 [31], 27 [32], and 39 in the OMIM database [33]; 153 in the Indian Genetic Disease Database (IGDD) [34,35]; and 153 in the NCBI Gene Database [36] (accessed on 10 July 2024). Bioinformatics-driven meta-analyses [37] allowed the list of the POAG-related genes to be expanded to 522 by adding human genes associated with ocular hypertension (OHT), juvenile open-angle glaucoma (JOAG), primary congenital glaucoma (PCG), and normal tension glaucoma (NTG) as pathologies diagnostically similar to POAG.

The ClinVar database entries related to the biomedical SNP markers of predisposition to human diseases [38] included 1155 biomedical SNP markers of predisposition to POAG as on 10 July 2024. (Hereinafter, “biomedical” should be understood as “clinical” for POAG patients and as “experimental” for laboratory POAG models using human or animal cells, tissues, and/or organs.) Most of these SNP markers modify the protein-coding regions and, consequently, protein structures and functions in an irreversible way. Regulatory region variants (regulatory SNPs) that only change the concentrations of the affected genes’ products occur much less frequently, and their effects can be compensated for by changing lifestyle or through the use of medication [39]. One of the regulatory SNP markers of predisposition to POAG is rs1143627:T in the promoter of the *IL1B* gene for human interleukin 1β, as a comparison of POAG patients and healthy individuals in Brazil suggests [40]. This SNP changes the affinity of this promoter to the TATA-binding protein (TBP) during the formation of the preinitiation complex [41], which is absolutely required for transcription initiation in knockout TBP-/- animals [42] and for a non-specific increase in gene expression levels proportional to the increase in TBP affinity for their promoters [43]. Consequently, an in silico prediction of candidate SNP markers that are capable of significantly changing TBP affinity for the promoters of the POAG-related genes can help identify the universal molecular mechanisms underlying processes involved in POAG pathogenesis.

We have previously developed and published SNP_TATA_Comparator, a freely available web service [44] for assessing in silico the level of significance *p* of the difference between TBP affinity for the ancestral and the minor variant of a given SNP in a human gene promoter. The use of this web service enabled us to predict candidate SNP markers for a broad range of disorders like age-related diseases [45], hypertension [46], and atherosclerosis [47], to name a few. SNP_TATA_Comparator was independently used by other researchers in a clinical search for SNP markers of predisposition to age-specific pulmonary tuberculosis [48]. The use of SNP_TATA_Comparator on the entire human genome resulted in Human_SNP_TATAdb [49], a knowledge base on all human genome-wide SNP markers that can either upregulate or downregulate protein-coding genes by changing TBP affinity for the promoters regulating these genes. We have also developed OrthoWeb [50,51], a freely available software package for estimating the phylostratigraphic age indices in automated mode, and ANDSystem [52], a toolbox for data mining analysis of the literature sources and databases. Here, we applied these two developments to perform a comprehensive analysis of the relationships between evolutionary origins, molecular mechanisms of function, and the whole-genome pattern for all 153 POAG-related genes in the NCBI Gene Database [36] as on 10 July 2024. The results obtained were verified against five independent web services—PANTHER [53], DAVID [54], STRING [55], MetaScape [56], and GeneMANIA [57]—as well as the ClinVar database [38].

## 2. Results

**Figure 1:**
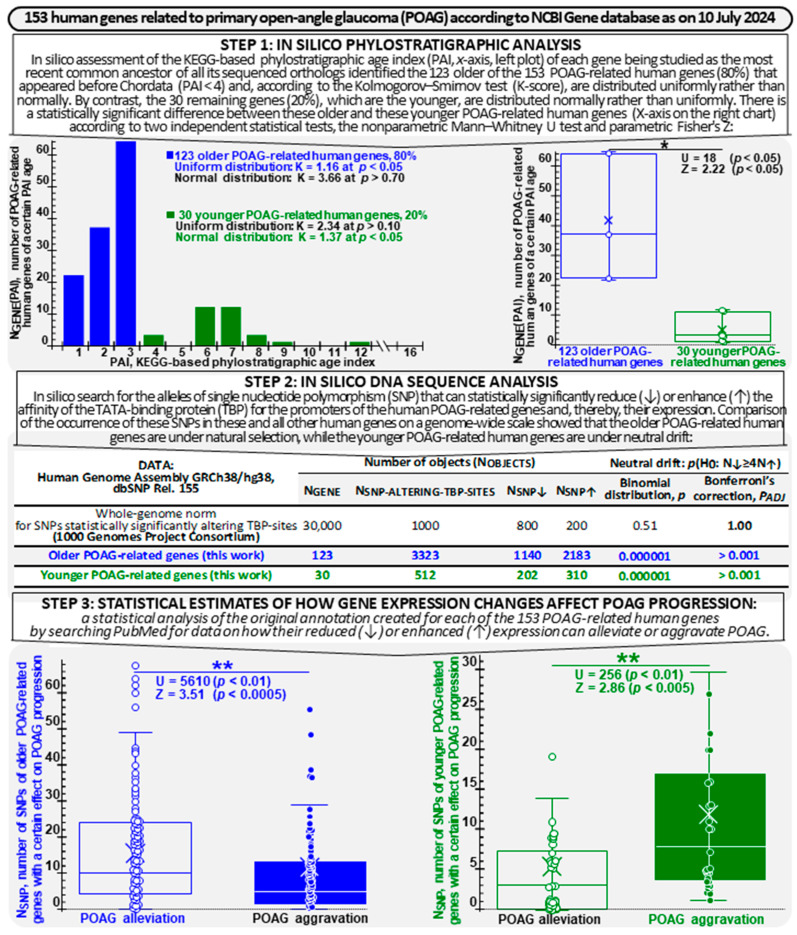
Flowchart depicting our step-by-step exploration of all 153 human genes associated with primary open-angle glaucoma (hereinafter: POAG-related genes) according to the NCBI Gene Database [36] as accessed on 10 July 2024. Legend: for the box-and-whisker plots, the height is the interquartile range (IQR) between the first (bottom) quartile (25%) and the third upper quartile (75%), the middle line is the median (50%), the oblique crosses (symbol “X”) are the arithmetic mean, the error bar (symbol “I”) is the 95% confidence interval, the circles depict the dataset in question; K, U, Z, along with *p* and P_ADJ_ are, respectively, the scores of Kolmogorov–Smirnov, Mann–Whitney U, and Fisher’s tests along with their statistical significance without and with Bonferroni’s correction for multiple comparisons; the single asterisk (symbol “*”) and the double asterisk (symbol “**”) stand for statistical significance at *p* < 0.05 and *p* < 0.01, respectively; PAI is the phylostratigraphic age index according to Kyoto Encyclopedia of Genes and Genomes (hereinafter: KEGG-based) [59,60], the numerical in silico estimates whereof are given in Table S1 (see Supplementary Materials), namely: 1. Cellular organism as the conventional root of the phylogenetic tree of life, 4100 million years ago (Mya) [61]; 2. Eukaryota, 1850 Mya [62]; 3. Metazoa, 665 Mya [63]; 4. Chordata, 541 Mya [64]; 5. Craniata, 535 Mya [64]; 6. Vertebrata, 525 Mya [65]; 7. Euteleostomi, 420 Mya [66]; 8. Mammalia, 225 Mya [67]; 9. Eutheria, 160 Mya [68]; 10. Euarchontoglires, 65 Mya [69]; 11. Primates, 55 Mya [70]; 12. Haplorrhini, 50 Mya [71]; 13. Catarrhini, 44 Mya [72]; 14. Hominidae, 17 Mya [73]; 15. Homo, 2.8 Mya [74]; 16. Homo sapiens, 0.35 Mya [75].

We explored all genes (n = 153) that, according to the NCBI Gene Database [36] as on 10 July 2024, are associated with POAG (see the top row of Figure 1). The GeneCards-related symbols [58] for each of these genes are listed in the “Human Gene” column of Table S1 (hereinafter, any digit prefixed with ”S” signifies that the corresponding figure or table is posted in Supplementary Materials [59,60,61,62,63,64,65,66,67,68,69,70,71,72,73,74,75,76,77,78,79,80,81,82,83,84,85,86,87,88,89,90,91,92,93,94,95,96,97,98,99,100,101,102,103,104,105,106,107,108,109,110,111,112,113,114,115,116,117,118,119,120,121,122,123,124,125,126,127,128,129,130,131,132,133,134,135,136,137,138,139,140,141,142,143,144,145,146,147,148,149,150,151,152,153,154,155,156,157,158,159,160,161,162,163,164,165,166,167,168,169,170,171,172,173,174,175,176,177,178,179,180,181,182,183,184,185,186,187,188,189,190,191,192,193,194,195,196,197,198,199,200,201,202,203,204,205,206,207,208,209,210,211,212,213,214,215,216,217,218,219,220,221,222,223,224,225,226,227,228,229,230,231,232,233,234,235,236,237,238,239,240,241,242,243,244,245,246,247,248,249,250,251,252,253,254,255,256,257,258,259,260,261,262,263,264,265,266,267,268,269,270,271,272,273,274,275,276,277,278,279,280,281,282,283,284,285,286,287,288,289,290,291,292,293,294,295,296,297,298,299,300,301,302,303,304,305,306,307,308,309,310,311,312,313,314,315,316,317,318,319,320,321,322,323,324,325,326,327,328,329,330,331,332,333,334,335,336,337,338,339,340,341,342,343,344,345,346,347,348,349,350,351,352,353,354,355,356,357,358,359,360,361,362,363,364,365,366,367,368,369,370,371,372,373,374,375,376,377,378,379,380,381,382,383,384,385,386,387,388,389,390,391,392,393,394,395,396,397,398,399,400,401,402,403,404,405,406,407,408,409,410,411,412,413,414,415,416,417,418,419,420,421,422,423,424,425,426,427,428,429,430,431,432,433,434,435,436,437,438,439,440,441,442,443,444,445,446,447,448,449,450,451,452,453,454,455,456,457,458,459,460,461,462,463,464,465,466,467,468,469,470,471,472,473,474,475,476,477,478,479,480,481,482,483,484,485,486,487,488,489,490,491,492,493,494,495,496,497,498,499,500,501,502,503,504,505,506,507,508,509,510,511,512,513,514,515,516,517,518,519,520,521,522,523,524,525,526,527,528,529,530,531,532,533,534,535,536,537,538,539,540,541,542,543,544,545,546,547,548,549,550,551,552,553,554,555,556,557,558,559,560,561,562,563,564,565,566,567,568,569,570,571,572,573,574,575,576,577,578,579,580,581,582,583,584,585,586,587]).

### 2.1. Assessment of the Phylostratigraphic Age Index (PAI) of the POAG-Related Genes

In step 1, we performed a phylostratigraphic analysis of all 153 POAG-related genes (Figure 1, the down arrow labeled “Step 1: in silico phylostratigraphic analysis”) by applying our publicly available software package OrthoWeb [50,51] (accessed on 10 July 2024) previously developed as a plug-in [588,589] to the web-based software environment Cytoscape [590] (for details, see Section 4.2).

In brief, the NCBI Gene identifier (ID) of a given human gene was used as input data for OrthoWeb [50,51], which automatically searched for all freely available DNA sequences of the corresponding orthologous animal genes according to the KEGG Orthology (KO) Database [59]. We then identified the taxonomic rank of the most recent common ancestor of these animal species as the “phylogenetic age index (PAI)”. The numerical value of this index is taken as its projection onto the taxonomic scale of molecular evolution according to the Kyoto Encyclopedia of Genes and Genomes (KEGG) [60]. Such numerical values were produced by OrthoWeb as outputs [50,51] for each human gene examined (the rightmost column “the most recent common ancestor” of Table S1).

We then performed a statistical analysis on the PAI scores obtained for all 153 POAG-related genes (for the results, see the histogram and the box-and-whisker plot in the upper half of Figure 1). As can be seen in the left-hand plot, the PAI score distribution of the 123 POAG-related genes (80%, blue) that appeared before the phylum Chordata (PAI = 4) is significantly closer to the uniform distribution (Kolmogorov–Smirnov test: K = 1.16 at *p* < 0.05) rather than to the normal distribution (K = 3.66 at *p* > 0.70). By contrast, the PAI score distribution of the remaining 30 POAG-related genes (20%, green) appears to be normal (K = 1.37 at *p* < 0.05) rather than uniform (K = 2.34 at *p* > 0.10). The uniform and normal distributions often approximate the frequencies of a biological object corresponding to the opposite extremes—(i) independent of one another [591], and (ii) interdependent on one another as integral parts of a whole—in line with the Central Limit Theorem [592]. Hence, the following question arises: do the differences between the 123 POAG-related genes that emerged before Chordata (PAI < 4) and the 30 POAG-related genes that emerged after (PAI ≥ 4) Chordata have any biological sense? We will, for brevity’s sake, call the former genes “older” rather than “more ancient” and the latter genes “younger” rather than “more recent” throughout.

The box-and-whisker plots in the upper right of Figure 1 show the differences in PAI scores between the POAG-related genes—123 older (blue) and 30 younger (green)—that were statistically significant according to the nonparametric Mann–Whitney *U* test (*U* = 18 at *p* < 0.05) and the parametric test Fisher’s Z (*Z* = 2.22 at *p* < 0.05) run independently of each other.

Thus, we can conclude that the significant difference between the 123 older POAG-related genes, which appeared before Chordata, and the remaining 30 younger POAG-related genes, which appeared before or after Chordata, seems to be fairly robust to various independent statistical criteria.

### 2.2. Data Mining Analysis of the POAG-Related Genes with ANDSystem

After completion of step 1, we performed data mining from the publicly available literature sources and databases to verify the differences found between the 123 older and the 30 younger POAG-related genes. With the use of ANDSystem [52] (accessed on 10 July 2024), we reconstructed two associative networks (see Figures S1 and S2) based on the two POAG-related gene sets (see Section 4.3).

Figures S1 and S2 graphically depict the data-mining results showing which molecular pathways can integrate the genes that appeared before or after Chordata. Figure S1 shows how the 123 older POAG-related genes contribute to the biological processes “pathogenesis” and “apoptotic process” as the two most significantly well-supported molecular pathways identified by the use of ANDSystem [52] (statistical significance levels after Bonferroni’s correction for multiple comparisons were P_ADJ_ = 10^−114^ and P_ADJ_ = 10^−102^, respectively). Briefly, once fed with the input gene sets, ANDSystem [52] automatically extracted data from PubMed articles [22] and many other publicly available databases. In this figure, the rightmost column contains 17 of the 123 older POAG-related genes; these 17 genes were not involved in either pathogenesis or apoptotic processes because we heuristically limited our analysis to as few as the two most significant pathways, allowing for the possibility that these genes may function in other pathways.

Additionally, 9 and 15 older POAG-related genes were found to correspond to pathogenesis and apoptotic processes, respectively. The remaining 82 older POAG-related genes appear between the circular arrow icons textually labeled with either “pathogenesis” or “apoptotic process”, which symbolizes their involvement in both molecular pathways at once.

As can be seen from Figure S2 (the notation being the same as above), five of the thirty younger POAG-related genes are involved in inflammatory responses; three, in immune responses; three, in neither; and each of the remaining nineteen participates in both pathways at a time.

Altogether, Figures S1 and S2 illustrate that the 123 older POAG-related genes are likely to differ from the 30 younger in at least four molecular pathways in which they function. However, this conclusion is only valid if these results are robust to variations across different independent data mining tools. For this reason, we fed the two human gene sets—the 123 older and the 30 younger POAG-related genes—to the independent web services PANTHER [53], DAVID [54], STRING [55], MetaScape [56], and GeneMANIA [57], which implement data mining. The results obtained are shown in Table 1.

### 2.3. Verification of ANDSystem Outputs for POAG-Related Genes Against Results Obtained with Independent Data Mining Tools

As can be seen from Table 1 [593], the results obtained using the five independent data mining tools and expressed in identifiers of either Gene Ontology terms [593] or KEGG pathways [60] were in part consistent with each other and in part not. Taking this into account, we heuristically searched the PubMed database [22] for original scientific articles in which competing Gene Ontology terms or KEGG pathways co-occurred in the same molecular genetic context (see Table 2).

For example, row #1 of Table 2 shows “pathogenesis” as ANDSystem outputs [52] (Figure S1) and “response to hypoxia” as DAVID outputs [54] (Table 2 [594,595,596,597,598,599,600,601,602,603,604,605,606,607,608,609,610,611,612,613,614,615,616,617,618]), even though the input data were identical, namely, the 123 older POAG-related genes. The rightmost column cites a biomedical review indicating that hypoxia-induced ocular injuries contribute to POAG pathogenesis, supporting the consistency of the data mining results. A row-by-row examination of Table 2 led us to the conclusion that there is no contradiction between the data mining results obtained with ANDSystem [52], on the one hand, and PANTHER [53], DAVID [54], STRING [55], MetaScape [56], and GeneMANIA [57], on the other hand. This may indicate that the 123 older and the 30 younger POAG-related genes function in different molecular pathways.

### 2.4. Supervised Annotation of the Effects of Changes in the POAG-Related Genes’ Expression Levels on POAG Alleviation and Aggravation

In step 2, we compiled biomedical data from PubMed [22] (accessed on 10 July 2024) on the effect of underexpression and overexpression of the 153 POAG-related genes that can alleviate or aggravate POAG (for details, see Section 4.4). A summary of POAG-related biomedical reports and reviews is provided in Table S2.

### 2.5. In Silico Estimation of the Effects of SNPs in the POAG-Related Genes’ Promoters on TBP Affinity for These Genes’ Promoters

With Human_SNP_TATAdb [49], we selected, within the promoters of all 153 POAG-related genes, the SNPs that—according to our previous in silico estimates—statistically significantly change the expression levels of these genes, as briefly described in Section 4.5 and in more detail in Supplementary Materials, the Section S1 named “supplementary methods for DNA sequence analysis”.

Table S3 lists all 3835 SNPs selected for analysis, each annotated with POAG-related biomedical data on how downregulation and upregulation of the 123 POAG-related genes can aggravate or alleviate POAG (Table S2).

### 2.6. Selective In Vitro Verification of In Silico Estimates of the Effects of SNPs in Human Gene Promoters on TBP Affinity for These Promoters

Before proceeding to statistical analysis of the effects that the POAG-induced changes in the expression levels of POAG-related genes should have on POAG progression according to our in silico estimation, we randomly verified these estimates using the corresponding experimental values. We had obtained and published these estimates previously (see Table S4). Row #1 of Table S4 presents a biomedical SNP marker of predisposition to POAG, rs1143627:T, which, according to a cohort-based study in Brazil [40], occurs in the human gene *IL1B*. As can be seen, this SNP marker corresponds to a C→T substitution, which leads to the formation of the canonical form of the TBP binding site, the TATA box (appears in uppercase further): “ttttgaaagcCataaaaacag” → “ttttgaaagcTATAAAAacag”. Based on our in silico estimates, this event accounts for about a 2.5-fold reduction in the equilibrium dissociation constant, K_D_, of the TBP–promoter complex, from 4.50 ± 0.39 to 1.76 ± 0.17 nanomoles per liter (nM) (Table S4). The ratio of these estimates, K_D_(min)/K_D_(WT) = 1.76/4.50 = 0.39, corresponds to 0.24, the value that we measured experimentally in one of our previous efforts [438].

**Figure 2:**
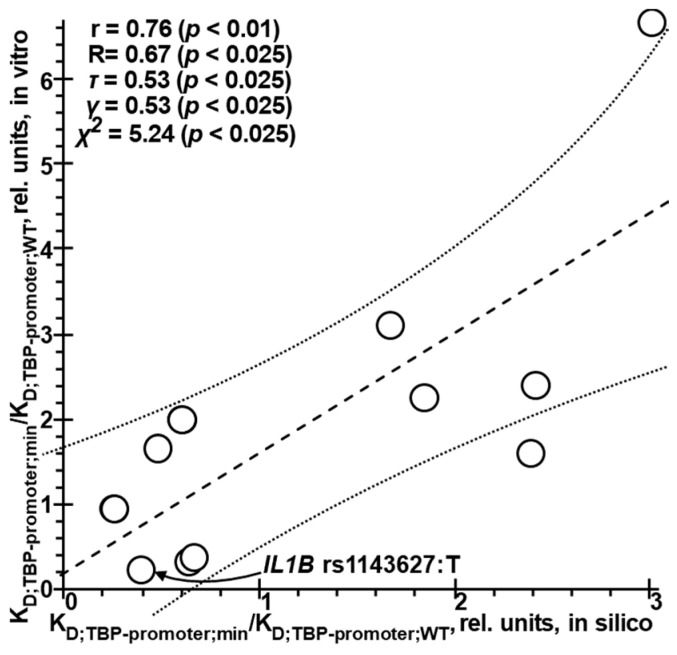
Significant correlations between the in silico—predicted (the *x*-axis) and in vitro—measured (the *y*-axis) K_D_ values of the equilibrium dissociation constant of the TBP–promoter complex expressed as the ratio of their estimates for minor alleles (min) to their estimates for the corresponding ancestral alleles (WT) (see Table S4 for graphical representation). Legend: the (→) arrow points to rs1143627:T in the promoter of the IL1B gene for human interleukin 1β, a clinically proven SNP marker of predisposition to POAG in Brazil [40]; dashed and dotted lines are linear regression and limits of its 95% confidence interval, as calculated by STATISTICA (StatSoft^TM^, Tulsa, OK, USA); r, R, τ, γ χ^2^, and *p* are, respectively, the linear correlation, Spearman’s rank correlation, Kendall’s rank correlation, Goodman–Kruskal generalized correlation coefficients, Pearson’s chi-squared statistic, and their statistical significance.

Figure 2 is a graphical representation of Table S3, where the “→” arrow points to rs1143627:T, the biomedical SNP marker of predisposition to POAG in Brazil [40]. This figure shows a total of 11 pairs of similar in silico estimates (the *x*-axis) against experimental in vitro data (the *y*-axis), which, according to five statistical criteria, significantly correlate with each other. The area between the two dotted curves corresponds to the 95% confidence interval, into which rs1143627:T falls [40]. This observation further supports the relevance of our previously made in silico estimates of the effects of SNPs on TBP affinity for human gene promoters, and reinforces the need for further in silico exploration of the effects of these SNPs on the promoters of POAG-related genes relating to predisposition to POAG. These estimates were published in the form of the Human_SNP_TATAdb knowledge base [49].

### 2.7. Frequencies of the SNPs That Significantly Change TBP Affinity for the Promoters of the POAG-Related Genes and for the Promoters of All Human Genes

We compared the occurrence of the 3835 SNPs that downregulate or upregulate the 153 POAG-related genes under study with the occurrence of the corresponding regulatory SNPs across the human genome, according to the 1000 Genomes Project Consortium [619,620,621] (see the table in the center of Figure 1). Row #1 of this table shows that, according to the 1000 Genomes Consortium [619,620,621], each individual human genome differs from the reference human genome by having an average of some 1000 regulatory SNPs, of which about 800 and 200 are, respectively, associated with underexpression and overexpression of the human genes whose promoters carry these SNPs.

Within the framework of Haldane’s dilemma [622] and Kimura’s theory of neutral evolution [623], this four-fold prevalence of the deleterious over beneficial regulatory SNPs corresponds to neutral drift in humans as a biological species on a genome-wide scale. As can be seen from the next two rows of the table, the occurrence of the SNPs that change the expression levels of the 123 older POAG-related genes and the 30 younger POAG-related genes significantly differs from the genome-wide pattern of the occurrence of regulatory SNPs under neutral drift (Figure 1: P_ADJ_ < 10^−3^, binomial distribution with Bonferroni’s correction).

Thus, we can conclude that all 153 POAG-related genes considered are under natural selection against their downregulation.

### 2.8. Assessing the Effects of SNP-Induced Increases and Decreases in the Expression Levels of the Older and Younger POAG-Related Genes on POAG Alleviation and Aggravation

In step 3, in order to understand where selection that is acting on the 153 POAG-related genes (the table in Figure 1) is directed, we statistically analyzed the annotations in this work on how often the candidate SNP markers that change the expression levels of these genes can alleviate or aggravate POAG (Table S4). The lower part of Figure 1 depicts the results obtained independently for the 123 older POAG-related genes (the left-hand (blue) box-and-whisker plot) and for the 30 younger POAG-related genes (the right-hand (green) box-and-whisker plot). As can be seen, the candidate SNP markers that significantly change the expression levels of the 123 older POAG-related genes mostly alleviate POAG, while those that have implications for the 30 younger POAG-related genes mostly aggravate it, according to the Mann–Whitney *U* test and Fisher’s Z.

### 2.9. Verification Results for the Proposed Candidate SNP Markers of POAG Using ClinVar Entries Related to Biomedical SNP Markers of Diseases

With the use of the publicly available database ClinVar [38] (accessed on 10 July 2024) containing biomedically proven SNP markers for human diseases, we checked each of the 3835 candidate SNP markers in the 153 POAG-related genes; these SNPs were taken from Human_SNP_TATAdb [49] (Table S5) as described in Section 4.7. Table S5 contains each of these 82 biomedical SNP markers associated with human diseases, these markers being known to change the expression levels of the 153 POAG-related genes, while Table 3 provides only a brief summary.

For example, row #1 of Table 3 contains two biomedical SNP markers for Tangier disease, rs886063317:C and rs886063317:G, which occur within the promoters of the human gene *ABCA1*. Table S5 details these as two nucleotide substitutions uppercased within their 10-base-pair flanks, namely: “agccgaatctAgcgctcggtg” → “agccgaatctCgcgctcggtg” and “agccgaatctAgcgctcggtg” → “agccgaatctGgcgctcggtg”. These substitutions may significantly downregulate this gene, thus potentially aggravating POAG through this molecular mechanism. Row #1 in the rightmost column of Table 3 references a comprehensive review [443] on the comorbidity of POAG and Tangier disease, highlighting the two biomedical SNP markers as an agreement between the examined entries of the ClinVar database [38] and Human_SNP_TATAdb knowledge base [49]. The bottom row of Table 3 summarizes that 17 out of the 18 human genes examined show similar agreements between these sources of information, with only one mismatch (the human gene *CP*). This level of agreement between the actual and predicted estimates of the effects of the SNP markers on POAG suggests statistical significance (P_ADJ_ < 0.05, binomial distribution criterion with Bonferroni’s correction).

### 2.10. RNA-Seq Data on Domestic and Wild Animals for Verification of the Proposed Candidate SNP Markers That Change the Expression Levels of the POAG-Related Genes

As can be seen in Figure 1, the 153 POAG-related genes are under natural selection (the table in step 2) on the one hand; on the other hand, this natural selection has two opposite directions (the box-and-whisker plots in step 3): one toward POAG alleviation and one toward POAG aggravation. This phenomenon is known as destabilizing (disruptive) natural selection, which characterizes, in particular, animal domestication [624].

This gave us another opportunity to independently verify the proposed candidate SNP markers that change the expression levels of the POAG-related genes against the transcriptome profiles of domestic and wild animals. To this end, we collected all relevant RNA-Seq data that we could find using the PubMed databases [22] as on 10 July 2024 (for details, see Section 4.8).

Table S6 presents 19 datasets taken from 12 original papers [45,46,566,567,568,569,570,571,572], where a total of 2912 DEGs were identified in nine tissues of seven pairs of domestic and wild animals. With the use of the “Paralogs” section of the freely available GeneCards database [58], we found, for each of the 153 POAG-related genes, its homologous animal DEGs (Table S7 and Table 4).

For example, the bottom row (#78) of Table S7 describes the human gene *VDR*, which encodes the vitamin D receptor. According to the left half of this row, *VDR* deficiency contributes to hypertension, which aggravates POAG [9], while elevated *VDR* protects against retinal ganglion cell loss, which alleviates POAG [429]. As is evident from the right-hand half of this row, the guinea pig has *VDR* deficiency in the frontal cortex, which its wild counterpart cavy does not [567]. We compared the *VDR* levels in both *Cavia* species against those in their nearest common ancestor, which is traditionally approximated by the mean value of the trait in question [625,626,627]. The comparison revealed that Vdr is deficient in the guinea pig and aggravates POAG and elevated in the cavy and alleviates POAG. Table S7 similarly describes expression changes in domestic and wild animals relative to those in their nearest common ancestors for 119 and 27 animal genes, which are homologous to the 123 older and the 30 younger POAG-related human genes, respectively (Table 4). As the entries in the bottom row of Table 4 suggest, the 123 older POAG-related genes are statistically significantly different from the 30 younger POAG-related genes in terms of the phenotypic manifestations of their animal homologs: POAG is alleviated in the wild animals and aggravated in the domestic animals (*p* < 0.05, binomial distribution test). That is why POAG aggravation during animal domestication provides evidence in favor of destabilizing natural selection, to which the POAG-related genes are subject (see Figure 1), within the current concept of the human self-domestication syndrome [628].

## 3. Discussion

### 3.1. Why the 153 POAG-Related Genes?

The most disputable point of this work is perhaps why we chose these 153 POAG-related genes. On the one hand, this choice led to the biological results being reported herein, while, on the other hand, the functional importance of many human gene loci that have been associated with POAG by GWAS and QTL remains unclear or unsupported by independent studies. Moreover, analysis of freely available sources revealed some reporting from 7 to 153 POAG-related genes [27,28,29,30,31,32,33,34], depending on the age and objectives of the research paper. Curiously, we found a source reporting as many as 522 for a meta-analysis of POAG and diagnostically similar diseases, namely, OHT, JOAG, PCG, and NTG [37].

A single NCBI Gene [36] query made on 10 July 2024 (for technical details, see Section 4.1) returned a 153-strong set of POAG-related genes, as did an independent source [34,35]. That encouraged us to proceed with these 153 POAG-related genes to ensure that an independent researcher will make the same choice.

### 3.2. POAG-Related Genes That Appeared Before and After Chordata Became Different as Lampreys Evolved the Camera-Type Eye

In this study, we for the first time found that the POAG-related genes that had appeared before and after Chordata differ from each other (see Figure 1 and Table 4). This is consistent with the current understanding of the molecular evolution of the eye [629]. Indeed, lancelets of the genus Branchiostoma, some of the older chordates have only Hesse’s eyecups [630,631], while the somewhat more evolutionarily advanced lampreys of the family Petromyzontidae have eyespot-like immature eyes beneath a non-transparent skin in their larva but their adults possess a camera-type eye [632], as do all the others in the human lineage on the Tree of Life [629]. As the only known modifiable risk factor for POAG [20] is high intraocular pressure (IOP), which is impossible without the camera-type eye, then the phylostratigraphic dilemma of having appeared before and after Chordata, in the context of POAG, comes down to the ophthalmological dilemma of having appeared before and after the camera-type eye. Thus, our finding that the POAG-related genes that appeared before and after Chordata aligns with current knowledge of both molecular evolution and POAG.

### 3.3. The 123 Older POAG-Related Genes Responsible for Pathogenesis and Apoptosis Play a Critical Role in How Misfolded Protein Aggregates Can Aggravate POAG

The blue-colored histogram in Figure 1 depicts the uniform distribution of the 123 older genes of all 153 POAG-related genes across three taxa—Cellular organism, Eukaryota, and Metazoa—on the evolutionary scale of Kyoto Encyclopedia of Genes and Genomes, KEGG [60]. Additionally, Figure S1 depicts the associative network of how these genes may contribute to pathogenesis and the apoptotic process based on our data mining through publicly available reports and databases. Indeed, in a cellular model of POAG [633], intracellular aggregates of misfolded proteins in the trabecular meshwork can induce apoptosis as a kind of pathogenesis, playing a key role in how misfolded protein aggregates can aggravate POAG. We can therefore state that our findings on the 123 older POAG-related genes that appeared before Chordata are consistent with what is known about the role of apoptosis in POAG pathogenesis.

Put together, these facts suggest that the uniform distribution of the 123 older POAG-related genes with respect to PAI (the blue-colored histogram in the upper left of Figure 1) may be interpreted biologically: the genes that produce misfolded proteins aggregating in intracellular complexes and thus aggravating POAG may have appeared independently in the course of evolution.

### 3.4. Normal Distribution of PAI Values of the 30 Younger POAG-Related Genes Involved in the Immune Response Has a Peak at Vertebrata, When Adaptive Immunity Appeared

The green-colored histogram in the upper left of Figure 1 corresponds to the normal distribution, peaking between the subphylum Vertebrata (PAI = 6525 Mya [65]) and the clade Euteleostomi (PAI = 7420 Mya [66]), according to the evolutionary scale of the Kyoto Encyclopedia of Genes and Genomes, KEGG [60]. Figure S2 shows the association gene network of these genes, where they are mainly involved in the immune response, according to our data mining through freely available publications and databases. This aligns with an original study [634] and a comprehensive review [635], both reporting on how the Transib transposon in jawed vertebrates (Gnathostomata, approximately 500 Mya) gave rise to the part of adaptive immunity that is able to recognize antigens due to V(D)J recombination in immunoglobulin genes during antibody maturation. Therefore, our results on the 30 younger POAG-related genes, which appeared after *Chordata*, are consistent with how immunity and POAG are viewed by the scientific community.

In summary, we propose a feasible biological interpretation of the normal distribution of the 30 younger POAG-related genes with respect to PAI (the green-colored histogram in the upper left of Figure 1): it is possible that shortly after *Chordata* evolved the camera-type eye, the natural selection of new POAG-related genes started to act so as to provide adaptive support to this eye’s architecture by producing the immune and inflammatory responses to POAG aggravating factors.

### 3.5. Differences Between Domestic and Wild Animals in How POAG-Related Genes Alleviate or Aggravate POAG Fit in with Current Views of Natural Selection in Domestic and Wild Animals

The bottom row of Table 4 shows that the pressure natural selection exerts on wild animals may alleviate POAG, while artificial selection during animal domestication may aggravate POAG, as our in silico calculations suggest. This conclusion is consistent with one made by Zhang and colleagues [636], stating that natural selection eliminates sick animal individuals in the wild, while artificial animal selection during domestication does not as it serves human needs; for example, the pig is farmed primarily for pork, no matter what the POAG status.

### 3.6. POAG as a Symptom of the Human Self-Domestication Syndrome Is Consistent with POAG Aggravation by Anthropogenic Factors

The above-discussed considerations suggest that POAG may be a symptom of the human self-domestication syndrome [628]. With PubMed [22], we found a medical opinion that industrialization increases POAG prevalence by facilitating the transmission of *Helicobacter pylori* infection [637]. In addition, a recent cohort-based study in Shanghai [638] revealed environmental pollution with fine particulate matter as a risk factor for POAG through elevation of intraocular pressure. Finally, according to a comprehensive biomedical review [639], circadian rhythm disruption aggravates POAG.

By summarizing the examples of how anthropogenic factors that never occur in the wild but can aggravate POAG nonetheless, we can conclude that this may be a symptom of the human self-domestication syndrome [628], a downside of being civilized.

### 3.7. Study Limitations

Admittedly, the setup of our in silico studies places limitations on the use of the 3835 candidate SNP markers that significantly alter TBP affinity for the promoters of the 153 POAG-related genes and, through this molecular mechanism, the expression levels of these genes. Because the consideration of these candidate SNP markers, for example in adjuvant therapy of POAG, may lead to health deterioration, any such uses should be preceded by cohort-based studies of these SNPs.

The results presented herein were obtained by considering TBP interactions with human gene promoters during the initiation of these genes’ transcription—specifically, at the point that these promoters that are densely packed into a transcriptionally inactive nucleosome re-assemble into the preinitiation complex as a stand-alone modulator of these genes’ expression levels, as independent experiments suggest (see for example [43]). Because the molecular mechanism operates in a step-by-step manner and so caters for the tissue-, stage-, and other types of specificity of human gene expression, our results reflect only the most general, non-specific patterns of aggravating or alleviating effects that the proposed candidate SNP markers have on POAG.

Finally, our in silico estimates of the phenotypic manifestation of the proposed candidate SNP markers of POAG are not absolute facts but are statistically significant: they are relevant only on a whole-genome scale for *Homo sapiens* as a biological species in the human lineage on the Tree of Life.

## 4. Materials and Methods

### 4.1. The Human Genes

For the purposes of our work, we entered the search query [“primary open-angle glaucoma” AND “Homo sapiens”] in the NCBI Gene online database search toolbox [36], with the filters “Genomic”, “Ensembl”, “Protein-coding genes”, “Annotated genes”, and “Current” set (accessed on 10 July 2024). The search returned 153 human genes (see the leftmost column “Human Gene” of Table S1).

### 4.2. In Silico Rating of the KEGG-Based Phylostratigraphic Age Index (PAI) of a Human Gene

We calculated the KEGG-based [59,60] PAI of a given human gene using its NCBI Entrez gene number as input data for OrthoWeb [50,51] (accessed on 10 July 2024), a plugin [588,589] within Cytoscape [590]. This yielded the most recent common ancestor of all animal species, whose DNA sequence of this gene is already sequenced and documented in the KEGG Orthology (KO) database [59]. Thus, the evolutionary rank scale used according to KEGG [60] was as follows: 1. Cellular organism as the conventional root of the phylogenetic tree of life, 4100 Mya [61]; 2. Eukaryota, 1850 Mya [62]; 3. Metazoa, 665 Mya [63]; 4. Chordata, 541 Mya [64]; 5. Craniata, 535 Mya [64]; 6. Vertebrata, 525 Mya [65]; 7. Euteleostomi, 420 Mya [66]; 8. Mammalia, 225 Mya [67]; 9. Eutheria, 160 Mya [68]; 10. Euarchontoglires, 65 Mya [69]; 11. Primates, 55 Mya [70]; 12. Haplorrhini, 50 Mya [71]; 13. Catarrhini, 44 Mya [72]; 14. Hominidae, 17 Mya [73]; 15. Homo, 2.8 Mya [74]; 16. *Homo sapiens*, 0.35 Mya [75].

### 4.3. Data Mining Analysis of Freely Available Publications and Databases Related to POAG

We performed data mining using ANDSystem [52] accessed on 10 July 2024, run in automated mode with “Human, [Human gene list], [Two most reasonable Pathways]” as input data, with all other parameters set to default. ANDSystem outputs for two variants of the [Human gene list]—(i) 123 older POAG-related genes and (ii) 30 younger POAG-related genes—are graphically represented as two associative gene networks (Figure S1 and Figure S2, respectively).

After that, we used the same 123 older and 30 younger POAG-related genes as inputs for PANTHER [53], DAVID [54], STRING [55], MetaScape [56], and GeneMANIA [57]. The outputs are given in Table 1.

Finally, we heuristically identified PubMed [22] articles that confirmed either agreement or disagreement between the ANDSystem outputs [52] and the results obtained by the above-listed data mining toolboxes (see Table 2).

### 4.4. Biomedical Data on the Effect of Underexpression and Overexpression of the POAG-Related Genes on POAG Alleviation and Aggravation

The biomedical data used in this work on how underexpression and overexpression of the POAG-related genes aggravates or alleviates POAG were obtained from Lu’s (2011) original article found in PubMed (accessed on 10 July 2024) using its web query service (see Table S2).

### 4.5. In Silico Estimation of How SNPs in the POAG-Related Genes’ Promoters Change These Genes’ Expression Levels

The in silico estimates for the levels of statistical significance of the effect of SNPs in the 90-base-pair proximal regions of the promoters of the 153 POAG-related genes on their underexpression and overexpression were taken from Human_SNP_TATAdb [49].

Briefly, we have previously analyzed [49] all 5,305,816 SNPs in the proximal 90-base-pair regions before the TSSs of all 63,141 protein-coding transcriptomes from all 19,314 genes annotated in the human whole reference genome from the assembly GRCh38/hg38 according to Ensembl [39] and dbSNP [437] accessed on 1 August 2023 (for the details of the algorithm used, see Supplementary Materials: Section S1). At each elementary step, SNP_TATA_Comparator [44] run in the automated mode helped assess the level of significance of the difference between the in silico estimates of TBP affinity for a given minor and the corresponding ancestral variant of an SNP in a human gene promoter according to the formulas in Supplementary Materials: Section S1. Each minor allele was assessed separately and if the estimate suggested a significant increase or decrease in TBP-promoter affinity and eventually in the expression levels of the corresponding human gene [43] against the ancestral allele being the norm, that minor allele along with the related calculations and results was recorded in Human_SNP_TATAdb [49]. In total, this knowledge base describes 445,875 SNPs, of which each can, according to our in silico estimates, significantly change the expression level of 1 of the 63,141 protein-coding human genes as compared to the reference variant of that gene taken for the norm.

Table S3 lists all the 3835 candidate SNP markers of changes in the expression levels of all the 153 POAG-related genes that were selected from among all 445,875 such SNPs identified for all the 63,141 protein-coding human genes and freely available in our the Human_SNP_TATAdb knowledge base [49], https://www.sysbio.ru/Human_SNP_TATAdb/ (accessed on 10 July 2024).

### 4.6. Selective Verification of the In Silico Estimates of the Effect of SNPs in Human Gene Promoters on TBP Affinity for These Promoters Against the Norm

Selective verification of the in silico estimates of the effect of SNPs in human gene promoters on TBP affinity for these promoters against the norm was performed using our previously published affinities changed experimentally with the following 11 SNPs: rs1402972626, rs20067072, rs1393008234, rs1452787381, rs1452787381, rs183433761, rs367781716, rs750827465, rs72661131, rs563763767, and rs1143627. The last in the row is a biomedical SNP marker of predisposition to POAG in Brazil, as a cohort-based study suggests [438] (see Table S4 for numerical representation and Figure 2 for a graphical representation).

### 4.7. Verification Methods for the Proposed Candidate SNP Markers of POAG Using ClinVar Entries Related to Biomedical SNP Markers of Diseases

Among the 3835 candidate SNP markers associated with significant changes in the expression levels of the 153 POAG-related genes, 82 were biomedically proven SNP markers linked to specific human diseases, according to the ClinVar database [38]. The information on these 82 SNPs is given in full in Table S5 and in brief in Table 3.

### 4.8. Differentially Expressed Genes (DEGs) in Domesticated Animals and Their Nearest Wild Counterparts

With the use of the web query search service of the PubMed database [22] accessed on 10 July 2024, we compiled all independent experimental transcriptome profiling datasets of tissues from domestic and wild animals [45,46,566,567,568,569,570,571,572,573,574,575] (see Table S6). Next, with the use of the “Paralogs” section of the GeneCards database [58], for each of the 153 POAG-related genes, we identified the paralogous animal genes that the domestic and wild animals express differentially; see Table S7 for detailed information and Table 4 for a summary.

### 4.9. Statistical Analysis

We performed the Kolmogorov–Smirnov test, Mann–Whitney *U* test, Fisher’s Z, Pearson’s linear correlation test, Spearman’s rank correlation test, Kendall’s rank correlation test, the Goodman–Kruskal generalized correlation test, Pearson’s chi-squared test, and the binomial distribution test using appropriate options in STATISTICA (StatSoft^TM^, Tulsa, OK, USA).

## 5. Conclusions

In this work, we have for the first time estimated the phylostratigraphic age indices (PAIs) of all 153 POAG-related genes in the NCBI Gene Database [36] accessed on 10 July 2024 (Table S1). This allowed us to separate them into two sets, one with the 123 older and one with the 30 younger POAG-related genes, which appeared before and after the phylum Chordata, respectively, or, in ophthalmological terms, before and after the evolution of the camera-type eye.

Next, we have for the first time predicted in silico all 3835 candidate SNP markers in the 90-base-pair promoters of these 153 POAG-related genes. These SNP markers significantly change TBP affinity for these promoters and, through this molecular mechanism, the expression levels of these genes, according to Ensembl [39] and dbSNP [437]. The 3835 candidate SNP markers are freely available in our Human_SNP_TATAdb database [49].

Finally, with the use of the biomedically proven SNP markers for human diseases in the ClinVar database [38] and 2905 DEGs of domestic and wild animals [45,46,568,571,572], we have selectively verified 82 of the 3835 candidate SNP markers within the 90-base-pair proximal promoters of 18 out of the 153 POAG-related genes.

## Figures and Tables

**Table 1:** Annotation of the 123 older and 30 younger POAG-related genes in the Gene Ontology [593] or KEGG [60] categories using five publicly available web services, PANTHER [53], DAVID [54], STRING [55], MetaScape [56], and GeneMANIA [57].

Category	No	123 Older POAG-Related Genes			30 Younger POAG-Related Genes		
		ID	Term	P_ADJ_	ID	Term	P_ADJ_
PANTHER [53]							
Biological Process	1	GO0070887	cellular response to chemical stimulus	10^−24^	GO0051241	negative regulation of multicellular organismal process	10^−5^
	2	GO0042221	response to chemical	10^−23^	GO0009617	response to bacterium	10^−5^
Molecular Function	3	GO0042802	identical protein binding	10^−7^	GO0005102	signaling receptor binding	10^−5^
	4	GO0019899	enzyme binding	10^−7^	GO0030545	signaling receptor regulator activity	10^−4^
Cell Component	5	GO0005615	extracellular space	10^−7^	GO0005576	extracellular region	10^−3^
	6	GO0031982	vesicle	10^−6^	GO0042825	transporter associated with antigen presentation (TAP) complex	10^−2^
DAVID [54]							
Biological Process	7	GO0001666	response to hypoxia	10^−6^	GO0010575	positive regulation of vascular endothelial growth factor production	10^−2^
	8	GO0051045	negative regulation of membrane protein ectodomain proteolysis	10^−6^	GO0032755	positive regulation of interleukin-6 production	10^−2^
Molecular Function	9	GO0002020	protease binding	10^−5^	GO0005125	cytokine activity	10^−4^
	10	GO0042802	identical protein binding	10^−4^	GO0005149	interleukin-1 receptor binding	0.05
Cell Component	11	GO0031012	extracellular matrix	10^−6^	GO0005576	extracellular region	10^−8^
	12	GO0005615	extracellular space	10^−4^	GO0005615	extracellular space	10^−5^
KEGG Pathway	13	hsa05205	proteoglycans in cancer	10^−15^	hsa04933	AGE-RAGE signaling pathway in diabetic complications	10^−4^
	14	hsa05417	lipid and atherosclerosis	10^−11^	hsa05332	graft-versus-host disease	10^−2^
STRING [55]							
Biological Process	15	GO0010033	response to organic substance	10^−21^	GO0009617	response to bacterium	10^−7^
	16	GO1901700	response to oxygen-containing compound	10^−21^	GO0006953	acute-phase response	10^−6^
Molecular Function	17	GO0005515	protein binding	10^−9^	GO0005102	signaling receptor binding	10^−4^
	18	GO0042802	identical protein binding	10^−8^	GO0030545	signaling receptor regulator activity	10^−4^
Cell Component	19	GO0005576	extracellular space	10^−6^	GO0005576	extracellular region	10^−3^
	20	GO0031982	vesicle	10^−5^	GO0005615	extracellular space	10^−2^
KEGG Pathway	21	hsa05205	proteoglycans in cancer	10^−17^	hsa04933	AGE-RAGE signaling pathway in diabetic complications	10^−7^
	22	hsa05200	pathways in cancer	10^−12^	hsa05332	graft-versus-host disease	10^−4^
MetaScape [56]							
Biological Process	23	GO0009725	response to hormone	10^−20^	GO0009617	response to bacterium	10^−7^
	24	GO0009410	response to xenobiotic stimulus	10^−19^	GO0007162	negative regulation of cell adhesion	10^−4^
KEGG Pathway	25	hsa05205	proteoglycans in cancer	10^−23^	hsa04933	AGE-RAGE signaling pathway in diabetic complications	10^−7^
	26	hsa05200	pathways in cancer	10^−18^	hsa05163	human cytomegalovirus infection	10^−4^
GeneMANIA [57]							
Biological Process	27	GO2001233	regulation of apoptotic signaling pathway	10^−10^	GO0071219	cellular response to molecule of bacterial origin	10^−20^
	28	GO0008285	negative regulation of cell population proliferation	10^−21^	GO0071216	cellular response to biotic stimulus	10^−19^

Note. P_ADJ_, the significance level adjusted for multiple comparisons as estimated in the specified web services.

**Table 2:** Comparison of ANDSystem [52] outputs with the results from other web services for assessing the enrichment of Gene Ontology terms [593] or KEGG pathways [60] in the gene groups.

ANDSystem [52]	#	PANTHER [53], DAVID [54], STRING [55], MetaScape [56], GeneMANIA [57]: Gene Ontology Terms and KEGG Pathways	Where ANDSystem [52] Agrees with PANTHER [53], DAVID [54], STRING [55], MetaScape [56], GeneMANIA [57] in Their Outcome Within PubMed [22]	
*123 older POAG-related genes studied in this work*				
pathogenesis	1	GO0001666: response to hypoxia	hypoxia-caused ocular injuries speed POAG pathogenesis [594]	
	2	GO0051045: negative regulation of membrane protein ectodomain proteolysis	within human disease models using dogs [595]: GO0051045 is one of the seventeen best GO-terms specifying heart failure pathogenesis	
	3	GO0002020: protease binding GO0042802: identical protein binding GO0005515: protein binding GO0019899: enzyme binding	within the bioinformatics meta-analysis of POAG-related transcriptome data along with GO-annotation [596]: protease binding and protein–protein interactions were found to accelerate POAG pathogenesis	
	4	GO0031012: extracellular matrix GO0005615: extracellular space	according to a comprehensive biomedical review [597]: extracellular matrix and space remodeling accelerate POAG pathogenesis	
	5	GO0010033: response to organic substance GO0042221: response to chemical	in human disease models using dog tears [598]: haptoglobin-based response to organic substances speeds POAG pathogenesis	
	6	GO1901700: response to oxygen-containing compound	oxidative stress can accelerate POAG pathogenesis [599]	
	7	GO0009725: response to hormone GO0070887: cellular response to chemical stimulus	during pregnancy and post-menopause, neuroprotective estrogen hormone therapy slows POAG pathogenesis [600]	
	8	GO0009410: response to xenobiotic stimulus	GO0009410 is a term specifying POAG pathogenesis [601]	
	9	GO0008285: negative regulation of cell population proliferation	within a cohort biomedical transcriptome meta-analysis [602]: GO0008285 is the best GO-term specifying POAG pathogenesis	
	10	hsa05205: proteoglycans in cancer hsa05200: pathways in cancer	in a cohort transcriptome meta-analysis [602]: hsa05200 is among the top five KEGG-pathways specifying POAG pathogenesis	
	11	hsa05417: lipid and atherosclerosis	in a cohort study [603]: atherosclerosis spurs POAG pathogenesis	
apoptotic process	12	GO2001233: regulation of apoptotic signaling pathway	according to a comprehensive biomedical review [604]: retinal ganglion cell apoptosis contributes to POAG pathogenesis	
	13	GO0031982: vesicle	apoptotic bodies are one of the types of extracellular vesicles [605]	
*30 younger POAG-related genes studied in this work*				
inflammatory response	14	GO0010575: positive regulation of vascular endothelial growth factor production	according to cohort clinical study [606]: vascular endothelial growth factor (VEGF) excess contributes to inflammation in POAG	
	15	GO0032755: positive regulation of IL6 production	IL6 excess contributes to inflammatory response in POAG [258]	
	16	GO0005125: cytokine activity	in a cohort study [258]: cytokine IL6 contributes to inflammation	
	17	GO0005149: interleukin-1 receptor binding	IL1 binds to its receptor, raising the inflammatory response [607]	
	18	GO0006953: acute-phase response	GO0006953 is a GO-term specifying inflammation in POAG [608]	
immune response	19	GO0005576: extracellular region GO0005615: extracellular space	within a cohort sclera sample study [609]: defects in the extracellular region and space can provoke an immune response in POAG	
	20	GO0009617: response to bacterium	dysbiosis in the gut–retina axis triggers an immune response [610]	
	21	GO0005102: signaling receptor binding GO0030545: signaling receptor regulator activity	a retrospective meta-analysis [611]: GO0005102 and GO0030545 are GO-terms specifying the immune response in osteoporosis	
	22	GO0007162: negative regulation of cell adhesion	altered cell adhesion causes an immune response in POAG [612]	
	23	GO0071219: cellular response to molecule of bacterial origin	within meta-analysis of both KEGG and Omnibus Database [613]: bacterial origin molecules can cause a cellular immune response	
	24	GO0071216: cellular response to biotic stimulus	a biotic stimulus can provoke a cellular immune response [613]	
	25	hsa04933: AGE-RAGE signaling pathway in diabetic complications	within human POAG models using pig [614]: hsa04933 is among the top ten KEGG pathways specifying the immune response	
	26	hsa05332: graft-versus-host disease (GvHD)	immune response can contribute to GvHD pathogenesis [615]	
	27	hsa05163: human cytomegalovirus infection	immune response to cytomegalovirus can aggravate POAG [616]	
	28	GO0051241: negative regulation of multicellular organismal process	human anticancer therapy models using mice [617]: GO0051241 is among the top ten GO-terms specifying the immune response	
	29	GO0042825: TAP complex	the TAP complex can contribute to the immune response [618]	

Note: hereinafter, “biomedical” should be understood as “clinical” for POAG patients and as “experimental” for laboratory POAG models using human or animal cells, tissues, and/or organs.

**Table 3:** Biomedical SNP markers taken from the ClinVar database [38] and their in silico estimated effects on human gene expression (Δ: “↓” reduced, “↑” increased), which can aggravate (“▼”) or alleviate (“▲”) POAG (☼), according to the Human_SNP_TATAdb database [49].

ClinVar Database [38]				Human_SNP_TATAdb Database [49]			∑ + -
#	NCBI Gene Symbol (Entrez Gene ID)	dbSNP ID:min [437]	Susceptibility to Human Disease	Δ: ↓ ↑	☼: ▼ ▲	How Susceptibility to Human Disease, Biomedical Markers of Which Are the SNPs in Question, Can Aggravate or Alleviate POAG According to the Current State of the PubMed Database, as Cited Using [Refs]	
1	*ABCA1* (19)	rs886063317:C, rs886063317:G	Tangier disease	↓	▼	Tangier disease (also known as familial high-density lipoprotein deficiency) is comorbid with POAG [443]	+
2	*ABCB1* (5243)	rs1584915287:A	Tramadol response	↑	▼	a healthy man experienced glaucomatous vision impairment after injecting tramadol as a painkiller [444]	+
3	*ATXN2* (6311)	rs695871:G	Spinocerebellar ataxia type 2	↑	▼	near-threshold glaucomatous changes in the optic nerve in patients with Spinocerebellar ataxia type 2 [445]	+
4	*BMP4* (652)	rs774069849:A	orofacial cleft, microphthalmia with brain and digit anomalies	↑	▼	these morpho-ontogenetic disorders of the human head development inevitably lead to developmental disorders of the eyes, as parts of the head colocalized along with all its other parts that can manifest as congenital, early-onset, pediatric, and juvenile forms of various glaucomas, including POAG [446]	+
5	*CP* (1356)	rs151304828:T	Ferroxidase deficiency	↑	▼	this biomedical SNP marker is a result of screening in healthy volunteers that after an in silico analysis of both the literature and factual biomedical data was labeled “Conflicting Pathogenicity Classifications”, while database Human_SNP_TATAdb documents—an in silico estimate of this SNP—was labeled “Ferroxidase excess “ rather than “Ferroxidase deficiency”, in line with a biomedical report [448] on this excess, which can be an antioxidant protector against inevitable damage of the optic nerve head sensing light flux	-
6	*ELN* (2006)	rs41410045:G, rs41410045:T, rs537200597:A, rs537200597:T	Supravalvar aortic stenosis, Cutis laxa, Williams syndrome	↑	▼	these four hereditary connective tissue disorders are capable of manifesting in morpho-ontogenetic eye defects such as a subluxation lentis leading to various congenital, early-onset, pediatric, and juvenile forms of glaucoma, including POAG [449]	+
7	*FAS* (355)	rs558072404:A	Autoimmune lymphoproliferative syndrome type 1	↑	▼	this disease is a form of lymphoproliferative disorder resulting from infection-related post-traumatic and\or post-surgery complications, which can lead to various morphological changes in eyes that can elevate intraocular pressure, aggravating POAG [450]	+
8	*GRIN2B* (2904)	rs797044930:T	Intellectual disability	↑	▼	intellectual disability can manifest with movement disorders, cortical development malformations, and cortical visual impairment [451], which are comorbid with POAG [452]	+
9	*IL1B* (3553)	rs1143627:T	Gastric cancer at *Helicobacter pylori*, POAG, and 57 inflammation-related disorders	↑	▼	all these diseases are comorbid with POAG [452,453,454,455,456,457,458,459,460,461,462,463,464,465,466,467,468,469,470,471,472,473,474,475,476,477,478,479,480,481,482,483,484,485,486,487,488,489,490,491,492,493,494,495,496,497,498,499,500,501,502,503,504,505,506,507,508,509,510,511,512,513,514,515,516,517,518,519,520,521,522,523,524,525,526,527,528,529,530,531,532,533,534,535,536,537,538,539,540,541,542,543,544,545,546,547,548,549,550,551,552,553,554], as readers can find in Table S5, namely, row #9, penultimate column on the right (see Supplementary Materials)	+
10	*PMM2* (5373)	rs751782324:A	PMM2-congenital glycosylation disorder	↓	▼	PMM2-congenital glycosylation disorder can aggravate POAG [330]	+
11	*LDLR* (3949)	rs1357531646:G, rs1568582310:C, rs747068848:C, rs879254501:C, rs879254502:C, rs879254503:G, rs879254505:C, rs879254506:C, rs879254507:G, rs879254511:C, rs969658891:A	Familial hypercholesterolemia	↓	▼	according to a cohort-based biomedical study [313]: familial hypercholesterolemia occurs in patients with POAG more often compared to those without POAG, which may be the aggravation of POAG	+
		rs121908042:A, rs193922571:A, rs201102461:A, rs2077073153:T, rs2077269298:A, rs730882080:T, rs730882081:G, rs762139262:T, rs769383881:A, rs769383881:T, rs869320648:A, rs875989899:T, rs879254486:T, rs879254502:A, ars879254506:A	Familial hypercholesterolemia and pathogenic cardiovascular phenotype	↑		according to cohort biomedical studies: both familial hypercholesterolemia occurs in patients with POAG more often compared to those without POAG [313] as well as pathogenic cardiovascular phenotype, which can aggravate POAG [555]	
12	*MFN2* (9927)	rs568548916:A, rs886045216:T, rs973376897:A	hereditary motor and sensory neuropathy with optic atrophy	↑	▼	according to a diseasome gene network encompassing human genes contributing simultaneously to amyotrophic lateral sclerosis and other diseases [556]: POAG along with hereditary motor and sensory neuropathy with optic atrophy is comorbid to amyotrophic lateral sclerosis	+
13	*MUTYH* (4595)	rs1645057147:C, rs752665489:G	hereditary cancer predisposition syndrome	↓	▼	hereditary cancer predisposition syndrome is associated with congenital hypertrophy of retinal pigment epithelium, which can cause advanced glaucomatous damage in the optic nerve that can aggravate POAG [557]	+
		rs1060504202:A, rs1064795596:A, rs1338038953:A, rs1553127879:T, rs1553136984:A, rs1553137062:A, rs1570591700:A, rs1570591700:T, rs1570591736:A, rs2275602:T, rs587788237:A, rs753502884:T, rs755928199:A, rs755928199:C, rs755928199:T, rs758246147:A, rs766584437:A, rs766584437:T, rs767402084:A, rs767402084:C, rs774530388:T, rs876658588:A, rs878854188:G, rs878854188:T		↑			
14	*STAT3* (6774)	rs780393027:A, rs902564848:T	hyper-IgE recurrent infection syndrome	↑	▼	hyper-IgE recurrent infection syndrome 1 can cause both ocular allergy and allergic conjunctivitis, the treatment of which with corticosteroids has a side effect in the aggravation of POAG [558]	+
15	*TAP1* (6890)	rs1408055208:T, rs202053684:T	MHC class I deficiency	↑	▼	MHC class I-knockout mice show a similarity to the very early stages of POAG development [559]	+
16	*TCF4* (6925)	rs1555710523:T, rs17522826:T, rs2047109965:T, rs2061383201:A	Pitt–Hopkins syndrome	↑	▼	high myopia is both a symptom of Pitt–Hopkins syndrome [560] and a risk factor for POAG [561]	+
17	*TP53* (7157)	rs1457582183:T, rs1597400604:A, rs34361146:A	Li–Fraumeni and cancer predisposition syndromes	↑	▼	tobacco smoking is a risk factor for POAG as well as for both Li–Fraumeni and hereditary cancer-predisposition syndromes [563]	+
18	*TXNRD2* (10587)	rs182857388:T, rs886509891:T	primary dilated cardiomyopathy	↑	▼	choroidal thickness in children with chronic heart failure through dilated cardiomyopathy is decreased [564] and is observed alongside POAG [565]	+

Total (∑: “+” as coincidence, “-” as mismatch): 17 coincidences and 1 mismatch (*p* < 0.0001, P_ADJ_ < 0.05; binomial distribution)**.**

**Table 4:** Verification of the results obtained by analysis of POAG-related genes using RNA-Seq data on domestic animals and their wild counterparts.

	(a) Animals	The Number of DEGs Whose Expression Changed in the Same Direction as Their Homologous Human Genes with a Given Effect on the Alleviation and Aggravation of POAG			
(b) Human		123 Older POAG-Related Genes		30 Younger POAG-Related Genes	
		Wild	Domestic	Wild	Domestic
**The Effect of Changes in the Expression of the POAG-Related Genes on the Alleviation and Aggravation of POAG**	**Alleviation**	69	60	12	8
	**Aggravation**	50	59	15	19
**Binomial Distribution, *p***		<0.05	>0.40	>0.30	<0.05

## Data Availability

The data presented in this study are publicly available in [Human_SNP_TATAdb] at [DOI: 10.18699/VJGB-23-85].

## Supplementary Materials

The supporting information can be downloaded at: https://www.mdpi.com/article/10.3390/ijms252312802/s1.

## Abbreviations

AAO	American Academy of Ophthalmology
APGS	Asia-Pacific Glaucoma Society
DEG	Differentially Expressed Gene
DT	digital tonometry
EGS	European Glaucoma Society
IOP	intraocular pressure
IQR	interquartile range as a height of a given box-and-whisker plot
JOAG	juvenile open-angle glaucoma
KEGG	Kyoto Encyclopedia of Genes and Genomes
KO	KEGG Orthology
Mya	million years ago
NTG	normal tension glaucoma
OCT	optical coherence tomography
OHT	ocular hypertension
PAI	phylostratigraphic age index
PCG	primary congenital glaucoma
POAG	primary open-angle glaucoma
SAP	standard automated perimetry
SNP	single-nucleotide polymorphism
TBP	TATA-binding protein
WHO	World Health Organization
