# Supplementary material for: Candidate SNP Markers Significantly Altering the Affinity of the TATA-Binding Protein for the Promoters of Human Genes Associated with Primary Open-Angle Glaucoma
Source: Int J Mol Sci. 2024 Nov 28;25(23):12802. doi: 10.3390/ijms252312802 (PMC11641052; doi:10.3390/ijms252312802)
Supplement: Supplementary file 1 [file ijms-25-12802-s001.zip › ijms-3262350-supplementary.pdf]

Supplementary Materials

Candidate SNP Markers Significantly Altering the Affinity of the TATA-Binding Protein for the Promoters of Human Genes Associated with Primary Open-Angle Glaucoma

Karina Zolotareva [1,2], Polina A. Dotsenko [1,2,3], Nikolay Podkolodnyy [1,2,4], Roman Ivanov [1], Aelita-Luiza Makarova [1], Irina Chadaeva [1,2], Anton Bogomolov [1,3], Pavel S. Demenkov [1,2], Vladimir Ivanisenko [1,2,3], Dmitry Oshchepkov [1,2,3] and Mikhail Ponomarenko [1,2,*]

1 Institute of Cytology and Genetics, Siberian Branch, Russian Academy of Sciences (ICG SB RAS), Novosibirsk 630090, Russia
2 Kurchatov Genome Center at the ICG SB RAS, Novosibirsk 630090, Russia
3 Department of Natural Sciences, Novosibirsk State University, Novosibirsk 630090, Russia
4 Institute of Computational Mathematics and Mathematical Geophysics, SB RAS, Novosibirsk 630090, Russia
* Correspondence: pon@bionet.nsc.ru; Tel.: +7-3833634991 (ext. 1311)

Abstract: Primary open-angle glaucoma (POAG) is the most common form of glaucoma. This condition leads to optic nerve degeneration and eventually to blindness. Tobacco smoking, alcohol consumption, fast-food diets, obesity, heavy weight lifting, high-intensity physical exercises, and many other bad habits are lifestyle-related risk factors for POAG. By contrast, moderate-intensity aerobic exercise and the Mediterranean diet can alleviate POAG. In this work, we for the first time estimated the phylostratigraphic age indices (PAIs) of all 153 POAG-related human genes in the NCBI Gene Database. This allowed us to separate them into two groups: POAG-related genes that appeared before and after the phylum Chordata, that is, ophthalmologically speaking, before and after the camera-type eye evolved. Next, in the POAG-related genes' promoters, we in silico predicted all 3835 candidate SNP markers that significantly change the TATA-binding protein (TBP) affinity for these promoters and, through this molecular mechanism, the expression levels of these genes. Finally, we verified our results against five independent web services—PANTHER, DAVID, STRING, MetaScape, and GeneMANIA—as well as the ClinVar database. It was concluded that POAG is likely to be a symptom of the human self-domestication syndrome, a downside of being civilized.

Keywords: human; primary open-angle glaucoma; gene; promoter; TBP; TATA box; SNP; candidate SNP marker; gene expression change; phylostratigraphic age; natural selection; in silico verification

---

CONTENTS

Table S1. KEGG-based [59,60] phylostratigraphic age indices (PAIs) of 153 human genes associated with the primary open-angle glaucoma (POAG) according to the NCBI Gene database accessed on 10 July 2024.

Figure S1. The associative network of how the 123 oldest POAG-related human genes under study may contribute to two most statistically reliable molecular genetic pathways, pathogenesis ($P_{ADJ} < 10^{-15}$) and apoptotic process ($P_{ADJ} < 10^{-12}$), according to our publicly available toolbox ANDSystem [52] with "Human", "pathways" and the list of the 123 oldest POAG-related human genes as input.

Figure S2. The associative network of how the 30 youngest POAG-related human genes under study may contribute to two most statistically significant molecular genetic pathways, inflammation response ($P_{ADJ} < 10^{-5}$) and immune response ($P_{ADJ} < 10^{-3}$), according to our publicly available toolbox ANDSystem [52] with "Human", "pathways" and the list of these genes as input.

Table S2. Effects of downregulation or upregulation of the human POAG-related genes on the alleviation and aggravation of POAG according to the NCBI Gene database [36], with annotations made according to the PubMed database [22] accessed on 10 July 2024.

Table S3. Candidate SNP markers in the 90-bp proximal promoters of 153 human genes associated with POAG according to our *in silico* analysis.

Table S4. Verification of the i*n silico* estimates of changes in TBP affinity for the minor (min) and the ancestral (WT) alleles of SNPs in human genes promoters taken from the Human_SNP_TATAdb knowledge base [49] against previously published experimental data on the same changes obtained *in vitro* by the electrophoretic mobility shift assay (EMSA), including rs1143627:T in the human gene IL1B, a clinically proven SNP marker for susceptibility to POAG in Brazil according to a cohort-based original research article [40]

Table S5. Comparison between the biomedically proven SNP markers for human diseases according to the ClinVar database [38] (access on 10 July 2024) and their annotation according to our *in silico* analysis.

Table S6. RNA-Seq data on domestic animals versus their wild counterparts (PubMed data [22]).

Table S7. Comparison of the effects of unidirectional changes (a) in the expression of the human POAG-related genes on the alleviation and aggravation of POAG and (b) in the expression of the corresponding animal homologous genes that are differentially expressed (DEG) during domestication on the microevolutionary events leading to differences between domestic and wild animals according to our *in silico* analysis.

Section S1. Supplementary methods for DNA sequence analysis.

Table S1. KEGG-based [59,60] phylostratigraphic age indices (PAIs) of 153 human genes associated with the primary open-angle glaucoma (POAG) according to the NCBI Gene database accessed on 10 July 2024.

Human gene		The most recent common ancestor		Human gene		The most recent common ancestor		Human gene		The most recent common ancestor	
#	*Symbol*	Taxon	PAI	#	*Symbol*	Taxon	PAI	#	*Symbol*	Taxon	PAI
1	ACE	Cellular Organisms	1	52	TIMP4	Eukaryota	2	103	ROCK1	Metazoa	3
2	APEX1	Cellular Organisms	1	53	TMCO1	Eukaryota	2	104	SEC14L2	Metazoa	3
3	CAT	Cellular Organisms	1	54	TP53	Eukaryota	2	105	SIX1	Metazoa	3
4	COL1A1	Cellular Organisms	1	55	TXNRD2	Eukaryota	2	106	SIX6	Metazoa	3
5	ELN	Cellular Organisms	1	56	VAV2	Eukaryota	2	107	SLC4A10	Metazoa	3
6	ENO4	Cellular Organisms	1	57	VAV3	Eukaryota	2	108	SLCO2A1	Metazoa	3
7	FNDC3B	Cellular Organisms	1	58	WDR36	Eukaryota	2	109	SPARC	Metazoa	3
8	GMDS	Cellular Organisms	1	59	XRCC1	Eukaryota	2	110	STAT3	Metazoa	3
9	GPX1	Cellular Organisms	1	60	ACVR1	Metazoa	3	111	TBK1	Metazoa	3
10	GSTM1	Cellular Organisms	1	61	ADIPOQ	Metazoa	3	112	TCF4	Metazoa	3
11	GSTO1	Cellular Organisms	1	62	ADRB2	Metazoa	3	113	TGFB2	Metazoa	3
12	GSTO2	Cellular Organisms	1	63	AFAP1	Metazoa	3	114	TGFBR3	Metazoa	3
13	GSTP1	Cellular Organisms	1	64	APBB2	Metazoa	3	115	TIMP1	Metazoa	3
14	GSTT1	Cellular Organisms	1	65	ASB10	Metazoa	3	116	TIMP3	Metazoa	3
15	MFN1	Cellular Organisms	1	66	ATXN2	Metazoa	3	117	TLR2	Metazoa	3
16	MUTYH	Cellular Organisms	1	67	B4GALT3	Metazoa	3	118	TLR4	Metazoa	3
17	OGG1	Cellular Organisms	1	68	BMP4	Metazoa	3	119	TNF	Metazoa	3
18	PADI2	Cellular Organisms	1	69	CACNA2D1	Metazoa	3	120	TRPC6	Metazoa	3
19	PMM2	Cellular Organisms	1	70	CAV1	Metazoa	3	121	TRPM5	Metazoa	3
20	PON1	Cellular Organisms	1	71	CAV2	Metazoa	3	122	VDR	Metazoa	3
21	TIMP2	Cellular Organisms	1	72	CDKN1A	Metazoa	3	123	VEGFA	Metazoa	3
22	TMTC2	Cellular Organisms	1	73	CDKN2B	Metazoa	3	124	CYP1B1	Chordata	4
23	ABCA1	Eukaryota	2	74	CNTNAP4	Metazoa	3	125	LOXL1	Chordata	4
24	ABCB1	Eukaryota	2	75	COL8A1	Metazoa	3	126	MMP2	Chordata	4
25	ABCC4	Eukaryota	2	76	COL8A2	Metazoa	3	127	CDH1	Vertebrata	6
26	AQP1	Eukaryota	2	77	CP	Metazoa	3	128	COCH	Vertebrata	6
27	ARHGEF12	Eukaryota	2	78	CXCR3	Metazoa	3	129	EDN1	Vertebrata	6
28	ATOH7	Eukaryota	2	79	CYP1A1	Metazoa	3	130	ESR2	Vertebrata	6
29	CDC7	Eukaryota	2	80	CYP46A1	Metazoa	3	131	IL1B	Vertebrata	6
30	CPAMD8	Eukaryota	2	81	EFEMP1	Metazoa	3	132	IL6	Vertebrata	6
31	CPNE1	Eukaryota	2	82	ESR1	Metazoa	3	133	PLXDC2	Vertebrata	6
32	DGCR8	Eukaryota	2	83	FAS	Metazoa	3	134	PTGFR	Vertebrata	6
33	EGFR	Eukaryota	2	84	FASLG	Metazoa	3	135	SERPINE1	Vertebrata	6
34	EPO	Eukaryota	2	85	FOXC1	Metazoa	3	136	TAP1	Vertebrata	6
35	HDAC6	Eukaryota	2	86	GAS7	Metazoa	3	137	TAP2	Vertebrata	6
36	HSPA4	Eukaryota	2	87	GLIS3	Metazoa	3	138	AGER	Euteleostomi	7
37	HSPA5	Eukaryota	2	88	GRIN2B	Metazoa	3	139	APOE	Euteleostomi	7
38	IGF1R	Eukaryota	2	89	HES1	Metazoa	3	140	DEFB4A	Euteleostomi	7
39	IL10	Eukaryota	2	90	LDLR	Metazoa	3	141	HAMP	Euteleostomi	7
40	MFN2	Eukaryota	2	91	LTBP2	Metazoa	3	142	IL1RN	Euteleostomi	7
41	MPP7	Eukaryota	2	92	MMP1	Metazoa	3	143	IL2	Euteleostomi	7
42	MTHFR	Eukaryota	2	93	MMP12	Metazoa	3	144	IL6R	Euteleostomi	7
43	NOS2	Eukaryota	2	94	MMP9	Metazoa	3	145	MLIP	Euteleostomi	7
44	NOS3	Eukaryota	2	95	MYOC	Metazoa	3	146	NCKAP5	Euteleostomi	7
45	OPA1	Eukaryota	2	96	NRG2	Metazoa	3	147	OPTC	Euteleostomi	7
46	PARP1	Eukaryota	2	97	NTF4	Metazoa	3	148	SPP1	Euteleostomi	7
47	PTGS1	Eukaryota	2	98	NTM	Metazoa	3	149	CDKN2A	Mammalia	8
48	RHOA	Eukaryota	2	99	NTRK2	Metazoa	3	150	IL1A	Mammalia	8
49	ROCK2	Eukaryota	2	100	OPTN	Metazoa	3	151	ITIH1	Mammalia	8
50	SIRT1	Eukaryota	2	101	PPARG	Metazoa	3	152	LRRC27	Eutheria	9
51	SLC23A2	Eukaryota	2	102	PRNP	Metazoa	3	153	CYP2C19	Haplorrhini	12

Notes. Hereinafter, PAI, a gene's phylostratigraphic age index evaluated against the KEGG-based scale [60] using the freely available web service OrthoWeb [51]; Mya, million years ago; KEGG-based PAI scale: 1, Cellular organism as the nearest common ancestor of all living organisms with the genomes deciphered to date as the root of their phylogenetic tree, 4100 Mya [61]; 2, Eukaryota, 1850 Mya [62]; 3, Metazoa, 665 Mya [63]; 4, Chordata, 541 Mya [64]; 5, Craniata, 535 Mya [64]; 6, Vertebrata, 525 Mya [65]; 7, Euteleostomi, 420 Mya [66]; 8, Mammalia, 225 Mya [67]; 9, Eutheria 160, 160 Mya [68]; 10, Euarchontoglires, 65 Mya [69]; 11, Primates, 55 Mya [70]; 12, Haplorrhini, 50 Mya [71]; 13, Catarrhini, 44 Mya [72]; 14, Hominidae, 17 Mya [73]; 15, Homo, 2.8 Mya [74]; 16, Homo sapiens, 0.35 Mya [75]. Genes: *ABCA1,* ATP binding cassette subfamily A member; *ABCB1,* ATP binding cassette subfamily B member 1; *ABCC4,* ATP binding cassette subfamily C member 4; *ACE,* angiotensin I converting enzyme; *ACVR1,* activin A receptor type 1; *ADIPOQ,* adiponectin, C1Q and collagen domain containing; *ADRB2,* adrenoceptor beta 2; *AFAP1,* actin filament associated protein 1; *AGER,* advanced glycosylation end-product specific receptor; *APBB2,* amyloid beta precursor protein binding family B member 2; *APEX1,* apurinic/apyrimidinic endodeoxyribonuclease 1; *APOE,* apolipoprotein E; *AQP1,* aquaporin 1 (Colton blood group); *ARHGEF12,* Rho guanine nucleotide exchange factor 12; *ASB10,* ankyrin repeat and SOCS box containing 10; *ATOH7,* atonal bHLH transcription factor 7; *ATXN2,* ataxin 2; *B4GALT3,* beta-1,4-galactosyltransferase 3; *BMP4,* bone morphogenetic protein 4; *CACNA2D1,* calcium voltage-gated channel auxiliary subunit alpha2delta 1; *CAT,* catalase; *CAV1,* caveolin 1; *CAV2,* caveolin 2; *CDC7,* cell division cycle 7; *CDH1,* cadherin 1; *CDKN1A,* cyclin dependent kinase inhibitor 1A; *CDKN2A,* cyclin dependent kinase inhibitor 2A; *CDKN2B,* cyclin dependent kinase inhibitor 2B; *CNTNAP4,* contactin associated protein family member 4; *COCH,* cochlin; *COL1A1,* collagen type I alpha 1 chain; *COL8A1,* collagen type VIII alpha 1 chain; *COL8A2,* collagen type VIII alpha 2 chain; *CP,* ceruloplasmin; *CPAMD8,* C3 and PZP like alpha-2-macroglobulin domain containing 8; *CPNE1,* copine 1; *CXCR3,* C-X-C motif chemokine receptor 3; *CYP1A1,* cytochrome P450 family 1 subfamily A member 1; *CYP1B1,* cytochrome P450 family 1 subfamily B member 1; *CYP2C19,* cytochrome P450 family 2 subfamily C member 19; *CYP46A1,* cytochrome P450 family 46 subfamily A member 1; *DEFB4A,* defensin beta 4A; *DGCR8,* DGCR8 microprocessor complex subunit; *EDN1,* endothelin 1; *EFEMP1,* EGF containing fibulin extracellular matrix protein 1; *EGFR,* epidermal growth factor receptor; *ELN,* elastin; *ENO4,* enolase 4; *EPO,* erythropoietin; *ESR1,* estrogen receptor 1; *ESR2,* estrogen receptor 2; *FAS,* Fas cell surface death receptor; *FASLG,* Fas ligand; *FNDC3B,* fibronectin type III domain containing 3B; *FOXC1,* forkhead box C1; *GAS7,* growth arrest specific 7; *GLIS3,* GLIS family zinc finger 3; *GMDS,* GDP-mannose 4,6-dehydratase; *GPX1,* glutathione peroxidase 1; *GRIN2B,* glutamate ionotropic receptor NMDA type subunit 2B; *GSTM1,* glutathione S-transferase mu 1; *GSTO1,* glutathione S-transferase omega 1; *GSTO2,* glutathione S-transferase omega 2; *GSTP1,* glutathione S-transferase pi 1; *GSTT1,* glutathione S-transferase theta 1; *HAMP,* hepcidin antimicrobial peptide; *HDAC6,* histone deacetylase 6; *HES1,* hes family bHLH transcription factor 1; *HSPA4,* heat shock protein family A (Hsp70) member 4; *HSPA5,* heat shock protein family A (Hsp70) member 5; *IGF1R,* insulin like growth factor 1 receptor; *IL10,* interleukin 10; *IL1A,* interleukin 1 alpha; *IL1B,* interleukin 1 beta; *IL1RN,* interleukin 1 receptor antagonist; *IL2,* interleukin 2; *IL6,* interleukin 6; *IL6R,* interleukin 6 receptor; *ITIH1,* inter-alpha-trypsin inhibitor heavy chain 1; *LDLR,* low density lipoprotein receptor; *LOXL1,* lysyl oxidase like 1; *LRRC27,* leucine rich repeat containing 27; *LTBP2,* latent transforming growth factor beta binding protein 2; *MFN1,* mitofusin 1; *MFN2,* mitofusin 2; *MLIP,* muscular LMNA interacting protein; *MMP1,* matrix metallopeptidase 1; *MMP12,* matrix metallopeptidase 12; *MMP2,* matrix metallopeptidase 2; *MMP9,* matrix metallopeptidase 9; *MPP7,* MAGUK p55 scaffold protein 7; *MTHFR,* methylenetetrahydrofolate reductase; *MUTYH,* mutY DNA glycosylase; *MYOC,* myocilin; *NCKAP5,* NCK associated protein 5; *NOS2,* nitric oxide synthase 2; *NOS3,* nitric oxide synthase 3; *NRG2,* neuregulin 2; *NTF4,* neurotrophin 4; *NTM,* neurotrimin; *NTRK2,* neurotrophic receptor tyrosine kinase 2; *OGG1,* 8-oxoguanine DNA glycosylase; *OPA1,* OPA1 mitochondrial dynamin like GTPase; *OPTC,* opticin; *OPTN,* optineurin; *PADI2,* peptidyl arginine deiminase 2; *PARP1,* poly(ADP-ribose) polymerase 1; *PLXDC2,* plexin domain containing 2; *PMM2,* phosphomannomutase 2; *PON1,* paraoxonase 1; *PPARG,* peroxisome proliferator activated receptor gamma; *PRNP,* prion protein (Kanno blood group); *PTGFR,* prostaglandin F receptor; *PTGS1,* prostaglandin-endoperoxide synthase 1; *RHOA,* ras homolog family member A; *ROCK1,* Rho associated coiled-coil containing protein kinase 1; *ROCK2,* Rho associated coiled-coil containing protein kinase 2; *SEC14L2,* SEC14 like lipid binding 2; *SERPINE1,* serpin family E member 1; *SIRT1,* sirtuin 1; *SIX1,* SIX homeobox 1; *SIX6,* SIX homeobox 6; *SLC23A2,* solute carrier family 23 member 2; *SLC4A10,* solute carrier family 4 member 10; *SLCO2A1,* solute carrier organic anion transporter family member 2A1; *SPARC,* secreted protein acidic and cysteine rich; *SPP1,* secreted phosphoprotein 1; *STAT3,* signal transducer and activator of transcription 3; *TAP1,* transporter 1, ATP binding cassette subfamily B member; *TAP2,* transporter 2, ATP binding cassette subfamily B member; *TBK1,* TANK binding kinase 1; *TCF4,* transcription factor 4; *TGFB2,* transforming growth factor beta 2; *TGFBR3,* transforming growth factor beta receptor 3; *TIMP1,* TIMP metallopeptidase inhibitor 1; *TIMP2,* TIMP metallopeptidase inhibitor 2; *TIMP3,* TIMP metallopeptidase inhibitor 3; *TIMP4,* TIMP metallopeptidase inhibitor 4; *TLR2,* toll like receptor 2; *TLR4,* toll like receptor 4; *TMCO1,* transmembrane and coiled-coil domains 1; *TMTC2,* transmembrane O-mannosyltransferase targeting cadherins 2; *TNF,* tumor necrosis factor; *TP53,* tumor protein p53; *TRPC6,* transient receptor potential cation channel subfamily C member 6; *TRPM5,* transient receptor potential cation channel subfamily M member 5; *TXNRD2,* thioredoxin reductase 2; *VAV2,* vav guanine nucleotide exchange factor 2; *VAV3,* vav guanine nucleotide exchange factor 3; *VDR,* vitamin D receptor; *VEGFA,* vascular endothelial growth factor A; *WDR36,* WD repeat domain 36; *XRCC1,* X-ray repair cross complementing 1.

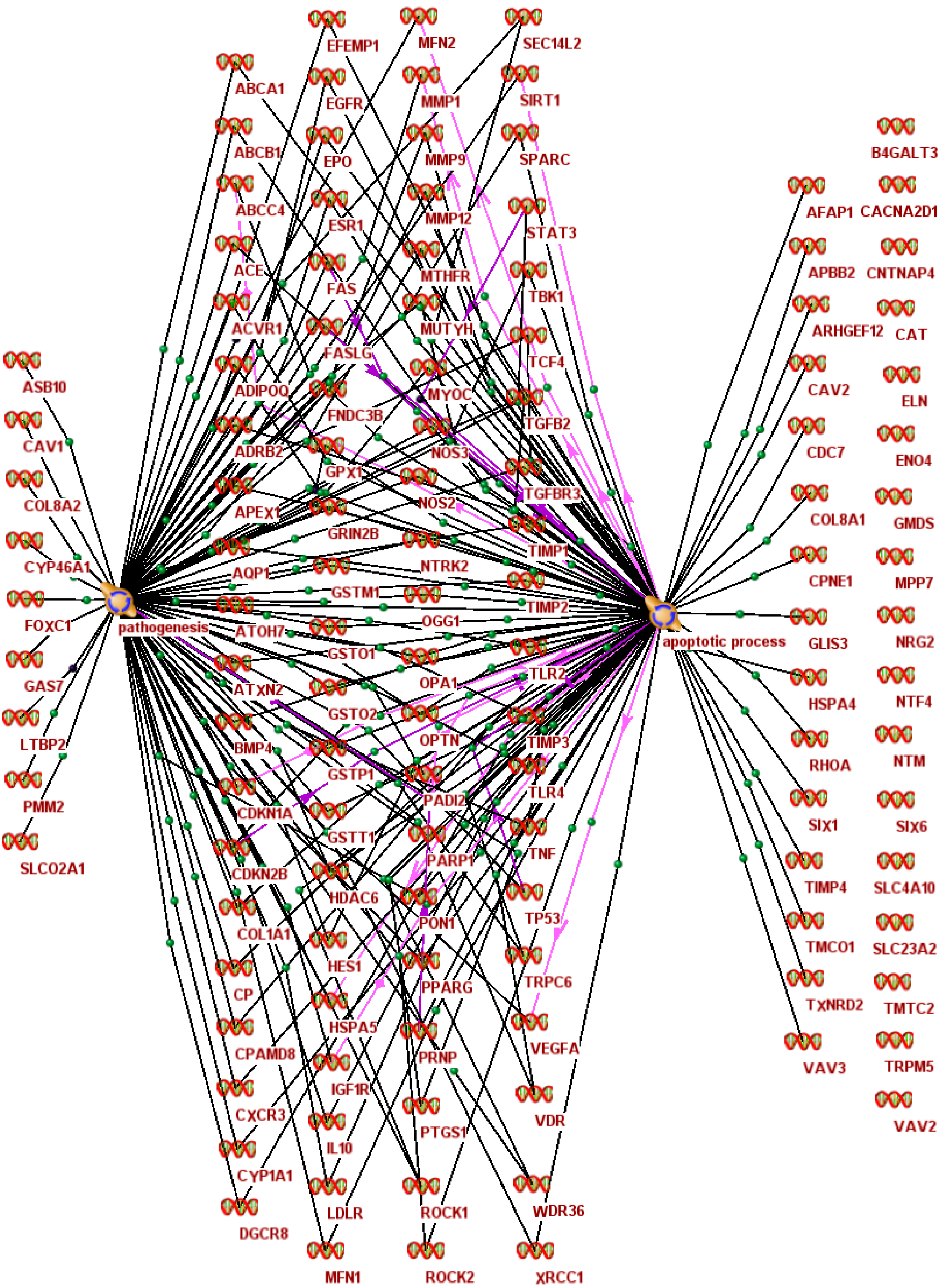

Figure S1. The associative network of how the 123 oldest POAG-related human genes under study may contribute to two most statistically reliable molecular genetic pathways, pathogenesis (PADJ < 10-15) and apoptotic process (PADJ < 10-12), according to our publicly available toolbox ANDSystem [52] with "Human", "pathways" and the list of the 123 oldest POAG-related human genes as input. *Legend*: "DNA helix"icons, gene; arrows: barb, activation; T-shaped, repression; black, involvement; pink, effect; purple, contribution.

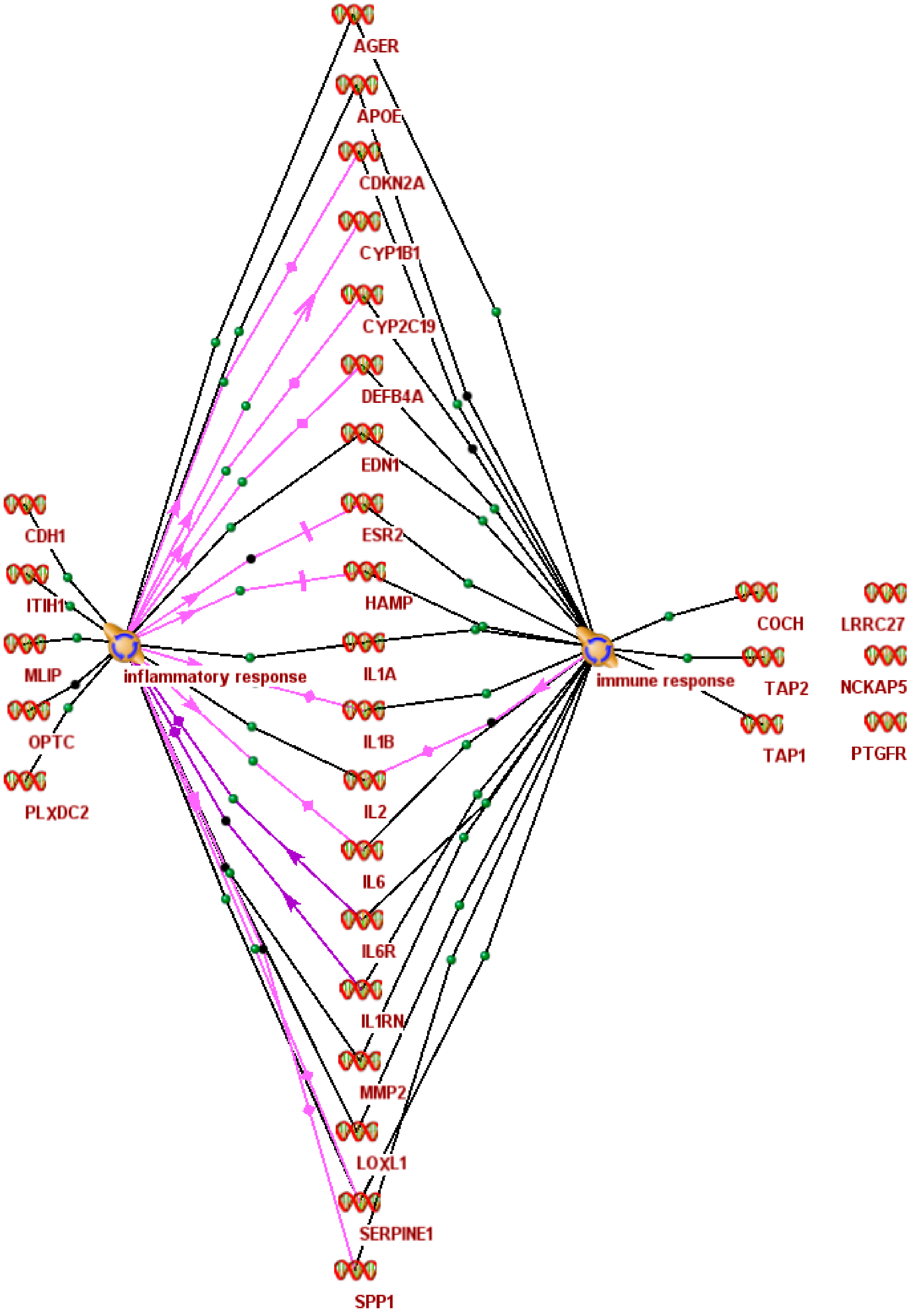

Figure S2. The associative network of how the 30 youngest POAG-related human genes under study may contribute to two most statistically significant molecular genetic pathways, inflammation response ($P_{ADJ} < 10^{-5}$) and immune response ($P_{ADJ} < 10^{-3}$), according to our publicly available toolbox ANDSystem [52] with "Human", "pathways" and the list of these genes as input. *Legend*: see legends to Figure S1.

Table S2. Effects of downregulation or upregulation of the human POAG-related genes on the alleviation and aggravation of POAG according to the NCBI Gene database [36], with annotations made according to the PubMed database [22] accessed on 10 July 2024.

No	NCBI Gene *Symbol* (ID)	Effect of changes in human gene expression on the development of primary open-angle glaucoma (POAG, ☼: "▼" aggravation, "▲" alleviation) [Reference]			
		**Deficit (↓)**	☼ ▼ ▲	**Excess (↑)**	☼ ▼ ▲
1	*ABCA1* (19)	within human disease models using Abca1-null mice [76]: accelerated vision loss can worsen POAG	▼	in a cohort-based biomedical study [77]: ABCA1 excess is a biomedical molecular marker for POAG	▼
2	*ABCB1* (5243)	within a biomedical pharmacological study using radiolabeled verapamil [78]: for this adjuvant anti-glaucoma medication [79], an ABCB1 inhibitor increased influx to and decreased outflow from the retina across the blood-retinal barrier	▲	within a cohort-based biomedical study [80]: the increasing level of ABCB1 expression with an increase in the duration of drug treatment may contribute to the molecular mechanisms of the emergence and progression of multidrug resistance, which may delay POAG treatment	▼
3	*ABCC4* (10257)	within human POAG models using Dutch-Belted rabbits subjected with topical application of MK571 as the low molecular weight Abcc4 inhibitor [81]: reduced intraocular pressure alleviating POAG	▲	within a comparative study of human pre- versus post-chemotherapy retinoblastoma cell transcriptome normalized using adjacent non-cancer cells [82]: anticancer chemotherapy increased ABCC4 expression by 33% along with an alleviation of retinoblastoma, which is comorbid to POAG according to a biomedical review [83]	▲
4	*ACE* (1636)	according to a comprehensive pharmacological mini-review [84]: ACE-inhibitors may be promising medications against POAG due to reducing angiotensin II levels that may have beneficial effects via lowering vascular superoxide anion production	▲	within a biomedical pharmaceutical study [85]: captopril (SQ 14225, ACE-inhibitor) reduced intraocular pressure, with no effects on heart rate and pupil diameter as a new promising anti-glaucomatous medication	▼
5	*ACVR1* (90)	within a Saudi cohort-based biomedical study [86]: minor allele "G" of the biomedical SNP marker rs12997 of POAG can upregulate ACVR1 through the target-site damage for miR-330-3p that may result in *ACVR1* downregulation by this miRNA as norm	▲	according to comprehensive review [87]: *ACVR1* upregulation follows 1 hour after an increase in intraocular pressure aggravating POAG	▼
6	*ADIPOQ* (9370)	within human disease cellular models using AdipoQ-deficient mice pre-adipocyte cell culture [88]: low AdipoQ level may be a molecular marker for obesity, insulin-resistance, and diabetes, which altogether form metabolic syndrome rising risks of POAG [89]	▼	within human disease cellular models using AdipoQ-deficient mice pre-adipocyte cell culture processed with thapsigargin as a new promising phytochemical medication inducing integrated stress response [88]: threefold increase in Adipoq protein level through improved translation as a protection against oxidative stress, obesity, insulin-resistance, and diabetes that altogether can relieve metabolic syndrome and reduce risks of POAG [89]	▲
7	*ADRB2* (154)	within an *in vitro* autoradiographic study of beta-adrenergic specific binding sites on the human retinal vessels [90]: ADRB2-blockers are drugs in the POAG treatment to reduce intraocular pressure	▲	within a biomedical cohort-based study [91]: ADRB2-excess along with its agonistic autoantibodies are biomedical molecular markers for any POAG development state	▼
8	*AFAP1* (60312)	within human disease cellular models using human AFAP1-knockdown cells [92]: reduced actin stress fiber cross-linking and decreased adhesion to fibronectin, each of which may slow a progression of POAG [93]	▲	according to an exhaustive retrospective review of POAG-related bioinformatic prioritization together with functional annotation of transcriptome-wide association studies [94]: AFAP1 upregulation might potentially protect against POAG	▲
9	*AGER* (177)	within human disease models using Ager-knockout mice subjected with an artificial diabetes [95]: protection against many retinopathic lesions, especially those related to innate immune responses as a prevention against diabetic retinopathy, which is comorbid with POAG, at least as age-related disease [96]	▲	within human disease models using diabetic rats treated with Zerumbone as a bioactive compound extracted from the rhizomes of *Zingiber zerumbet* [97]: Zerumbone has reversed back Ager-upregulation accompanied by both disarrangement and thickness reduction in retinal layers as symptoms of retinopathy, which is comorbid with POAG, at least as an age-related disease [96]	▼
10	*APBB2* (323)	within human disease models using *Apbb2*-knockout mice [98]: cortical cataracts, reduced lens transparency, and ocular muscle dysfunction that altogether may aggravate POAG [99]	▼	within biomedical cohort-based genome-wide APBB2 SNP association study of a population of Polish centenarians [100]: APBB2 overexpressing/beta-amyloid accumulating alleles fit in with longevity along with the very first phenotypic manifestations of cognitive impairment only in the elderly, as something like neuroprotection against POAG	▲

Table S2. *Cont.*

No	NCBI Gene *Symbol* (ID)	Effect of changes in human gene expression on the development of primary open-angle glaucoma (POAG, ☼: "▼" aggravation, "▲" alleviation) [Reference]			
		**Deficit (↓)**	☼ ▼ ▲	**Excess (↑)**	☼ ▼ ▲
11	*APEX1* (328)	within human disease models using mice subjected with an artificially induced retinal angiogenesis, and, next, treated with APX3330 as a small molecule inhibitor of Apex1 redox activity [101]: blocked retinal neovascularization, which can provoke severe peripheral retinal hemorrhages aggravating POAG [102]	▲	within a cohort-based biomedical study using liquid chromatography-mass spectrometry [103]: *APEX1* upregulation in tear fluid of patients with primary Sjogren's syndrome is a biomedical molecular marker for ocular dryness, which may result in dry eye disease, which is comorbid to POAG at least as a side effect of antihypertensive medications in POAG [104]	▼
12	*APOE* (348)	within human disease models using *ApoE*-deficient mice [105]: neuroprotection against axonal damage-induced retinal ganglion cell death	▲	in cohort-based biomedical study using quantitative real-time polymerase chain reaction (qPCR) and enzyme immunosorbent assays (ELISA test) [106]: excess of both APOE mRNA and APOE protein are biomedical molecular markers for POAG	▼
13	*AQP1* (358)	within human POAG models using mice lacking Aqp1 [107]: reduced both aqueous fluid production and intraocular pressure that might alleviate POAG	▲	according to a comprehensive biomedical review [108]: within human post-injury trabecular meshwork regeneration cellular models the using adipose-derived stem cells, AQP1 excess along with improved wound healing in that might slow down optic nerve head damage as the alleviation of POAG	▲
14	*ARHGEF12 (23365)*	within a retrospective literature meta-analysis [109]: *ARHGEF12* deficit might be a candidate molecular marker for reduced organism viability	▼	according to a retrospective literature meta-analysis [109]: improved eye development	▲
15	*ASB10 (136371)*	according to comprehensive review [110]: ASB10 mutational loss-of-function may impair an adequate response to inflammatory signals in the eye that could lead to glaucomatous vision loss	▼	within cellular POAG models using human and mice cells [111]: improved autophagy, which may alleviate POAG associated with increased intraocular pressure [112]	▲
16	*ATOH7* (220202)	within a biomedical cohort-based study of gene copy number variation using microarrays of blood samples [30]: only *ATOH7* deletion was significantly frequent in POAG	▼	within the transcriptome of retinal ganglion cells as a comparative model of their artificially induced regeneration versus their artificially induced degeneration using mice and zebrafish [113]: Atoh7 excess fits regeneration preventing POAD	▲
17	*ATXN2* (6311)	within human disease models using *Atxn2*-knockout mice [114]: dyslipidemia and insulin resistance as well as progressive obesity, which are positively correlating with POAG [115]	▼	within human neurodegenerative disease models using Atxn2-overexpressing mice [116]: neurodegeneration, which can aggravate POAG [117]	▼
18	*B4GALT3* (8703)	within a cohort-based biomedical study [118] along with human disease models using Chinese hamster ovary cells [119]: B4GALT3 deficit can lead glycan deficiency in the trabecular meshwork that can contribute to POAG	▼	within a retrospective comparative cohort-based biomedical data meta-analysis [120] along with human neoplasia models using a large variety of human cancer cell lines [121]: B4GALT3 excess can elevate risk of neoplasia, which can reduce lifespan in POAG compared to lifespan in other glaucomas	▼
19	*BMP4* (652)	within human disease models using mice subjected with an artificial glaucoma induction and, next, treated with recombinant adeno-associated virus carrying the human CHRDL1 gene as BMP4-blocker [122]: BMP4-blockade decreases the number of surviving retinal ganglion cells	▼	according to a cohort-based biomedical study [123]: BMP4 excess is a biomedically proven molecular marker for corneal dystrophy comorbid to POAG [124]	▼
20	*CACNA2D1* (781)	within cellular models of human eye injuries [125]: CACNA2D1-blockers improve eye injury repair that can relieve POAG	▲	within cellular models of human diseases using transgenic mice overexpressing *Cacna2d1* [126]: pain hypersensitivity that can worsen POAG	▼
21	*CAT* (847)	within a cohort-based blood serum and tear study [127]: catalase insufficiency in lacrimal fluid can be a biomedical molecular marker of free radical oxidation activation, antioxidant defense system suppression and endothelium dysfunction that altogether can worsen POAG	▼	within *in vitro* cellular POAG models using human trabecular meshwork cell line subjected with extracellular vesicles derived from the non-pigmented ciliary epithelium [128]: catalase upregulation reduced oxidative stress and thus alleviated POAG	▲

Table S2. *Cont.*

No	NCBI Gene *Symbol* (ID)	Effect of changes in human gene expression on the development of primary open-angle glaucoma (POAG, ☼: "▼" aggravation, "▲" alleviation) [Reference] Deficit (↓)	☼ ▼ ▲	Excess (↑)	☼ ▼ ▲
22	*CAV1* (857)	within human disease models using Cav1-knockout mice [129]: blood-retinal barrier breakdown, mural cell alteration, and venous enlargement with branch veins being frequent sites of breakdown that altogether may contribute to retinal disorders with vascular pathologies, including retinopathy, uveoretinitis, and POAG	▼	within human POAG cellular models using human embryonic kidney cell line HEK293 transfected by vector with one more mice Cav1 gene [130]: caveolin-1 excess along with protection against lipotoxicity, which is risk factor for elevated intraocular pressure aggravating POAG [131]	▲
23	*CAV2* (858)	within a biomedical cohort-based comparative study of minor G versus ancestral T alleles of the known biomedical SNP-marker rs17588172 for POAG using Korean participants [132]: minor G allele downregulates CAV2 that elevates intraocular pressure worsening POAG	▼	within cellular POAG models using differentiated rat PC12 cell line [133]: caveolin-2 is up-regulated in response to the mechanical injury of differentiated PC12 cells that can improve post-traumatic neuron repair retarding POAG development	▲
24	*CDC7* (8317)	within a cohort-based study [134]: CDC7-blockers within a gene-target therapy can retard atherogenesis as a risk-factor of POAG [135]	▲	within a cohort-based biomedical study [136]: Cdc7 overexpression contributes to DNA-damage resistance that can alleviate POAG [137]	▲
25	*CDH1* (999)	within human disease cellular models [138]: CDH1 insufficiency as a molecular marker for differentiation to epithelial cells can elevate risks of epithelial to mesenchymal transition and, thus, fibrosis as a post-POAG-surgery complication	▼	within human disease cellular models [138]: CDH1 excess as a molecular marker for differentiation to epithelial cells can prevent epithelial to mesenchymal transition and, thus, fibrosis as a post-POAG-surgery complication	▲
26	*CDKN1A* (1026)	according to a comprehensive retrospective biomedical review [139]: CDKNA1A-deficiency can complicate wound healing after glaucoma filtration surgery that may be prevented due to an adjunctive ocular gene therapy using an adenovirus vector containing an additional copy of the human CDKN1A gene	▼	within human disease models using human lung fibroblast strain CRL-1262 under UV-irradiation [140]: UV dose-dependent overexpression of CDKN1A along with either cell survival due to this dose-dependent excess of CDKN1A arresting the cell cycle until DNA damage caused by UV radiation is successfully repaired, or CDKN1A-induced cell death if DNA damage in question appears to be too great and unrepairable	▲
27	*CDKN2A* (1029)	within a retrospective comprehensive complex GWAS and QTL meta-analysis [141]: three biomedically proven SNP-markers rs1063192, rs7865618 and rs2157719 for POAG are associated with CDKN2A decreased expression gene in the cerebral cortex	▼	according to comprehensive biomedical review [142]: CDKN2A excess can prevent atherosclerosis aggravating POAG [143]	▲
28	*CDKN2B* (1030)	within human POAG models using *Cdkn2b*-knockout mice [144]: more vulnerable to retinal ganglion cell loss in response to elevated intraocular pressure that can aggravate POAG	▼	within a phytopharmaceutical microarray-based transcriptome study using murine microglial cells treated with 15-methoxypinusolidic acid as a modern promising anti-POAG phytomedication extracted from *Biota orientalis* [145]: Cdkn2b excess causing microglia cell cycle arrest, which can inhibit proliferation of microglia cells and induce apoptosis of these cells that may slow down neurodegeneration of both optic nerve and retina	▲
29	*CNTNAP4* (85445)	within a cohort-based biomedical study [146]: CNTNAP4-deficit is a biomedical molecular marker for major depressive disorder, which is comorbid with POAG at least as an age-related developmental neurodegenerative disorder [147]	▼	within a biomedical cohort-based transcriptome study of neurodegenerative diseases systematized using five non-overlapping clusters by means of multiple sclerosis severity degrees according to magnetic resonance imaging scans [148]: CNTNAP4 upregulation was detected in the only inactive form of the mildest smallest neurodegenerations, which can themselves heal over time, that may correspond the slowest neurodegeneration of optic nerve alleviating POAG	▲
30	*COCH* (1690)	within human anti-bacterial humoral innate immune response models using Coch-knockout mice [149]: less immune response after infection with different bacteria as susceptibility to bacterial infections that can aggravate POAG according to a biomedical case report [150]	▼	according to a comprehensive review of human disease models using young DBA/2J mice susceptible to POAG [151]: Coch-excess may be a biomedical molecular marker for elevated intraocular pressure, retinal ganglion cell degeneration, and optic nerve damage in older ages	▼
31	*COL1A1* (1277)	within human POAG filtration surgery models using mice [152]: auxiliary treatment with mitomycin-C during surgery downregulates Col1a1 and simultaneously prevents fibrosis as post-surgery complication	▲	within a biomedical cohort-based RNA-Seq study [153]: COL1A1 excess is a biomedical molecular marker for POAG	▼

Table S2. *Cont.*

No	NCBI Gene *Symbol* (ID)	Effect of changes in human gene expression on the development of primary open-angle glaucoma (POAG, ☼: "▼" aggravation, "▲" alleviation) [Reference]			
		**Deficit (↓)**	☼ ▼ ▲	**Excess (↑)**	☼ ▼ ▲
32	*COL8A1* (1295)	within human disease models using Col8a1-knockout mice [154]: thinning of both corneal stroma and of Descemet's membrane as symptoms of POAG [155]	▼	within a biomedical cohort-based transcriptome study of microneedling therapy [156]: COL8A1 upregulation is a biomedical molecular marker for wound healing, which is the major problem of glaucoma surgery according to a comprehensive retrospective review [157]	▲
33	*COL8A2* (1296)	within human POAG models using Col8a2-defective mutant mice [158]: retinal ganglion cell damage resistance, which can alleviate POAG	▲	within both biomedical cohort-based transcriptome study of post-surgical corneal wound healing in glaucoma patients and biomedical glaucoma surgery models using mice [159]: COL8A2 upregulation is one among three most vital gene expression changes during the very first week of wound healing immediately after glaucoma surgery until its fibrotic complication may begin that can require auxiliary therapy suppressing collagen-encoding genes	▲
34	*CP* (1356)	within a biomedical cohort comparison study of patients with cataracts with and without POAG [160]: low serum ceruloplasmin levels are molecular markers of POAG risk in cataract	▼	within nonhuman primate POAG models using male juvenile cynomolgus monkeys (*Macaca fascicularis*) treated with an argon laser to scar the trabecular meshwork in one eye until an intraocular pressure increased indicating glaucomatous eye [161]: ceruloplasmin excess in the retina that may be needed as part of antioxidant system, which must counteract the inevitable damaging effects of free radicals on the head of the optic nerve when perceiving light flux as a vital function of the eyes	▲
35	*CPAMD8* (27151)	within human disease models using zebrafish embryos carrying the CRISPR/Cas9-disruped *Cpamd8* gene [162]: anterior segment dysgenesis as microphthalmia, which is comorbid with POAG [163]	▼	within human disease cellular models using both human glioblastoma U251 and rhabdomyosarcoma RD cell lines [164]: CPAMD8 excess can contribute to innate immune response, which can in turn improve retinal ganglion cell survival after injury cause by an increase in intraocular pressure according to comprehensive review [165]	▲
36	*CPNE1* (8904)	within human disease cellular models using siRNA-based CPNE1-knockdown [166]: inhibited tumor growth and promoted cell apoptosis in breast cancer, a complication of which is tamoxifen-associated maculopathy in POAG [167]	▲	within human disease cellular models using human nasopharyngeal carcinoma cell lines NPC under different radiations up to 8 Gy [168]: CPNE1 excess along with improved radioresistance that can prevent glaucomagenesis following ionizing radiation exposure according to comprehensive biomedical review [169]	▲
37	*CXCR3* (2833)	within human disease models using mice subjected with artificial traumatic optic neuropathy [170]: CXCR3-antagonist medication reduced retinal inflammation that may relieve POAG	▲	within a biomedical cohort-based study [171]: CXCR3 excess in peripheral blood lymphocytes is a biomedical marker for accelerated progression of the earlier stages of POAG	▼
38	*CYP1A1* (1543)	within human disease models using senescence-accelerated OXYS rats as a commonly accepted animal model of human age-related macular degeneration compared with Wistar rats as a norm [172]: Cyp1a1-deficit in the retina may indicate the presence of oxidative stress contributing both retinopathy and age-related macular degeneration, which are both comorbid with POAG, at least as age-related diseases [96]	▼	within models of the effect of smoking on human health using rats [173]: *Cyp1a1*-upregulation in the iris/ciliary body and retina together with age-related macular degeneration with choroidal neovascularization as the most severe complication	▼
39	*CYP1B1* (1545)	within a biomedical cohort-based study [174]: both reduced catalytic activity and decreased protein stability can elevate risks of POAG	▼	according to a biomedical radiotherapy review [169]: ultraviolet radiation can upregulate CYP1B1 that may often manifest in glaucomagenesis as side effect following ionizing radiotherapy	▼
40	*CYP2C19* (1557)	within a biomedical cohort study [175]: reduced efficiency of CYP2C19 as a metabolizer may increase risks of side effects of timolol as an anti-POAG drug	▼	within a biomedical cohort study [175]: increased efficiency of CYP2C19 as a metabolizer may reduce risks of side effects of timolol as an anti-POAG drug	▲
41	*CYP46A1* (10858)	within human POAG models using rats [176]: voriconazole, as CYP46A1-inhibitor, plays a retinotoxic role under glaucomatous conditions	▼	within human POAG models using Cyp46a1-deficient mice [177]: pharmacologic activation of CYP46A1 may be a therapy for dyslipidemia-induced retinal damage and thereby slow the progression of POAG	▲

Table S2. *Cont.*

No	NCBI Gene *Symbol* (ID)	Effect of changes in human gene expression on the development of primary open-angle glaucoma (POAG, ☼: "▼" aggravation, "▲" alleviation) [Reference]			
		Deficit (↓)	☼ ▼ ▲	Excess (↑)	☼ ▼ ▲
42	*DEFB4A* (1673)	within a biomedical cohort-based study [178]: DEFB4A-deficit leads to corneal damage in thyroid eye disease, which is comorbid with POAG among eye diseases that may be aggravated by tobacco smoking [179]	▼	within a biomedical cohort study [180]: DEFB4A-excess is a biomedical molecular marker for POAG regardless gender, local antihypertensive therapy duration, disease stage, ophthalmotonus level as well as it may be increase after surgery against POAG	▼
43	*DGCR8* (54487)	within human age-related macular degeneration models using Dgcr8-deficient mice [181]: higher risks of mature retinal pigmented epithelium cell death and vision loss that is positively genetically correlating POAG according to a biomedical cohort study [182]	▼	within human disease cellular models using mouse lung epithelial cell line LM2 transfected with lentiviral vector overexpressing Dgcr8 under radiation exposure 8 Gy [183]: DGCR8 excess along with improved radioresistance that can prevent glaucomagenesis following ionizing radiation exposure according to comprehensive biomedical review [169]	▲
44	*EDN1* (1906)	within a biomedical cohort-based study [184]: EDN1-deficit is a risk factor of post-trabeculectomy low intraocular pressure, which is sight threatening because of bleb infection and hypotony maculopathy as postoperative complications in POAG	▼	within human POAG models using rats subjected with intravitreal injection of exogenous recombinant EDN1 [185]: both optic nerve axon degeneration and retinal ganglion cell apoptosis were accelerated, aggravating POAG	▼
45	*EFEMP1* (2202)	within human disease models using Efemp1-knockout mice [186]: corneal dysfunction, which is comorbid to POAG at least within exfoliation syndrome as the most common identifiable cause of POAG in the world [187]	▼	within a biomedical cohort-based study [188]: fibulin-3 overexpression holds future potential as new promising intraocular pressure lowering therapy for POAG	▲
46	*EGFR* (1956)	within a cohort-based biomedical pharmacological study [189]: anti-cancer medications inhibiting EGFR may elevate risks of dry eye, which is comorbid to POAG at least as a side effect of antihypertensive medications in POAG [104]	▼	within human disease models using chicken [190]: low molecular weight medication brimonidine treats POAG due to trans-activation of Egrf	▲
47	*ELN* (2006)	within a biomedical cohort-based proteome study of glaucomatous optic neuropathy [191]: elastin-deficit in the optic nerve head from Caucasian Americans patients compared with African Americans patients as a population-specific biomedical molecular marker for POAG	▼	within a cohort biomedical qPCR study [192]: *ELN* upregulation is a biomedical molecular marker for POAG	▼
48	*ENO4* (387712)	within a cohort-based biomedical studies [193]: ENO4-deficit is a biomedical molecular marker for autoimmune thyroid disease, a risk-factor of which is ophthalmic surgery against POAG [194]	▼	within a cohort-based biomedical studies [193]: ENO4-excess is a biomedical molecular marker for resistance against the autoimmune thyroid disease, a risk-factor of which is ophthalmic surgery against POAG [194]	▲
49	*EPO* (2056)	within human POAG models using DBA/2J mice strain pups of subjected with adenoviral vector carrying mutant *Epo* gene with reduced erythropoietic [195]: this neuroprotective gene therapy preserved vision due to decreasing infiltration of peripheral immune cells, modulating microglial reactivity, and decreasing oxidative stress	▲	within a biomedical cohort-based study [196]: EPO excess in plasma is a biomedical molecular marker for POAG	▼
50	*ESR1* (2099)	within an ethnopharmacological study of anti-diabetic Gegen Qinlian Decoction (GQD) within Chinese traditional medicine [197]: ESR1-deficit is a biomedical molecular marker for diabetes mellitus, which is comorbid with POAG, at least due to their mutual comorbidity with diabetic retinopathy [198]	▼	within a human neuroprotection cellular models using primary neocortical cell cultures established from mouse embryos on the 15th day of gestation, which were first exposed at either hypoxia or ischemic conditions and, next, treated with ospemifene as both new promising neuroprotective medication and novel selective estrogen receptor modulator simultaneously [199]: four-fold ESR1 protein excess compared to the normal condition along with neuroprotective effects by means of both proapoptotic proteins suppression and antiapoptotic proteins upregulation that altogether may slow the progression of POAG in terms of neurodegeneration of both the optic nerve and retinal ganglion cells	▲

Table S2. *Cont.*

No	NCBI Gene *Symbol* (ID)	Effect of changes in human gene expression on the development of primary open-angle glaucoma (POAG, ☼: "▼" aggravation, "▲" alleviation) [Reference]			
		Deficit (↓)	☼ ▼ ▲	Excess (↑)	☼ ▼ ▲
51	*ESR2* *(2100)*	according to a biomedical case report [167]: a complication of tamoxifen therapy for ESR2-deficient pT1 breast benign tumor to prophylaxis carcinogenesis may be ocular toxicity that may cause POAG, retinopathy and loss of visual acuity	▼	within a human neuroprotection cellular models using primary neocortical cell cultures established from mouse embryos on the 15th day of gestation, which were first exposed at either hypoxia or ischemic conditions and, next, treated with ospemifene as both new promising neuroprotective medication and novel selective estrogen receptor modulator simultaneously [199]: nine-fold ESR2 protein excess compared to the ischemic condition along with neuroprotective effects by means of both proapoptotic proteins suppression and antiapoptotic proteins upregulation that altogether may slow the progression of POAG in terms of neurodegeneration of both the optic nerve and retinal ganglion cells	▲
52	*FAS* (355)	within human disease model using Fas-deficient mice subjected with an single intracameral injection of microbeads elevating intraocular pressure [200]: resistance to POAG, which was then confirmed in an independent experiment using ONL1204 as a low molecular weight peptide inhibitor against Fas	▲	within human disease model using mice [200]: Fas-activation induced retinal ganglion cell apoptosis, glial activation, and inflammation as a complication of POAG	▼
53	*FASLG* (356)	within human neuroprotection models using Faslg-knockout mice [201]: reduced death of retinal ganglion cells that may slow the progression of POAG in terms of neurodegeneration of retinal ganglion cells	▲	within human POAG models using a hybrid mice line expressing only full-length Faslg responsible for accelerated retinal ganglion cell death, which was injected soluble short-length Faslg [201]: reduced death of retinal ganglion cells that may slow the progression of POAG in terms of neurodegeneration of retinal ganglion cells	▲
54	*FNDC3B* (64778)	within a cohort-based biomedical RNA-Seq study [202]: *FNDC3B*-downregulation can be a biomedical molecular marker for stable state in POAG	▲	within a cohort-based biomedical RNA-Seq study [202]: *FNDC3B*-upregulation can be a biomedical molecular marker for progression in POAG	▼
55	*FOXC1* (2296)	within human POAG cellular models using cultured human trabecular meshwork cells transfected with plasmids carrying either normal or mutant *FOXC1* with several variants of reduced activity [203]: the viability of human trabecular meshwork cells decreases with a decrease in FOXC1 activity in POAG	▼	within human sepsis-associated neurodegeneration encephalopathy models using 8-10 month aged male C57BL/6J mice subjected with artificially caused post-surgery sepsis, which both downregulated *Foxc1* and caused cognitive disorders as detected experimentally, and, next, transfected with adenoviral vector carrying the mouse *Foxc1* gene [204]: Foxc1 overexpression along with improved cognitive abilities that seems to be something like post-traumatic neuroregeneration alleviating POAG	▲
56	*GAS7* (8522)	within a biomedical cohort-based RNA-Seq study [205]: GAS7-downregulation can be a biomedical molecular biomarker of POAG	▼	according to the very first study focused on the mice *Gas7* gene localization, sequencing and phenotypically characterization [206]: *Gas7* overexpression can manifest in termination in neuron differentiation followed neurite-like outgrowth, which can improve post-traumatic retinal neuroregeneration according to human POAG models using mouse retinal neuronal precursor cell line RGC5 differentiating into either cone photoreceptor or ganglion cells due to treatment with the histone deacetylase inhibitor Trichostatin A (TSA) as a new promising regenerative medication extracted and purified from the bacterium *Streptomyces hygroscopicus* [207]	▲
57	*GLIS3* (169792)	within human disease models using Glis3-deficient mice [208]: susceptibility to neonatal diabetes, which is comorbid with POAG at list within diabetic retinopathy [198]	▼	according to a comprehensive pharmaceutical review [209]: enhancing GLIS3 activity may help patients with many GLIS3-insufficiency related diseases including glaucomas	▲

Table S2. *Cont.*

No	NCBI Gene *Symbol* (ID)	Effect of changes in human gene expression on the development of primary open-angle glaucoma (POAG, ☼: "▼" aggravation, "▲" alleviation) [Reference]			
		Deficit (↓)	☼ ▼ ▲	Excess (↑)	☼ ▼ ▲
58	*GMDS* (2762)	within human POAG models using zebrafish [210]: Gmds deficit can reduce fucosylation that may lead to defects in neuronal differentiation and maintenance	▼	within a biomedical cohort-based study [211]: GMDS excess is a biomedical molecular marker for carcinogenesis, which is comorbid to POAG at least in their commonly accepted general sensitivity to ultraviolet radiation-inducible oxidative stress [212]	▼
59	*GPX1* (2876)	within human disease model using elderly mice subjected to artificial weakening of neuroprotective abilities [213]: in POAG, Gpx1 deficit may be a biomedical molecular marker for both reduced retinal ganglion cell survival and retinal ganglion cell loss in the elderly because of their hypersensitivity to oxidative stress	▼	according to a comprehensive molecular medicine review [214]: medication-derived Gpx1 upregulation might open potential future therapies to harness the beneficial effects of this major ubiquitous antioxidant enzyme, which must counteract the inevitable damaging effects of free radicals on the optic nerve head when perceiving light flux as a vital function of the eyes in line with nonhuman primate POAG models using male juvenile cynomolgus monkeys (*Macaca fascicularis*) treated with an argon laser to scar the trabecular meshwork in one eye until an intraocular pressure increased indicating glaucomatous eye [161]	▲
60	*GRIN2B* (2904)	within human neurodegenerative disease cellular models using human induced pluripotent stem cells carrying either GRIN2B-deletion or GRIN2B loss-of-function mutation [215]: impaired both calcium influx and membrane depolarization that can aggravate POAG [216]	▼	within human disease models using mice [217]: Grin2b excess can enhance glutamatergic signaling, which can elevate excitotoxicity accelerating neurodegeneration in POAG	▼
61	*GSTM1* (2944)	within an exhaustive meta-analysis of the occurrence of single-nucleotide polymorphisms (SNPs) in human subpopulations with different regional and ethnic specificities [218]: GSTM1-null genotypes are associated with increased POAG risk in Asian populations	▼	within human disease model using rats subjected with corneal wounds, which was then regularly irradiated with blue light to slow down its healing and, at the same time, treated orally with the *Peucedanum japonicum* Thunb. leaf extract to repair these wounds [219]: extract-dose-dependent acceleration in wound healing due to Gstm1-upregulation that can alleviate POAG that can improve post-surgery recovery in POAG	▲
62	*GSTO1* (9446)	within human disease models using Gsto1-knockout mice subjected with an artificial photo-oxidative damage of retina [220]: decreased photoreceptor cell death, inflammation and oxidative stress as well as improved retinal function	▲	within human disease models using mice subjected with an artificial acute hypoglycemia [221]: Gsto1-upregulation together with accelerated retinal cell death as a POAG symptom [222]	▼
63	*GSTO2* (119391)	within human disease models using neuron-specific Gsto2-knockdown Drosophila line [223]: decreased the solubility of proteins homologous to human *FUS* in neurons in an age-dependent manner that can contribute to neurodegenerative proteinopathies, such as amyotrophic lateral sclerosis and dementia as well as POAG [224]	▼	within human disease models using Gsto2-overexpressing Drosophila line [225]: improved both locomotive activities and neuromuscular junctions that can prevent neurodegenerative proteinopathies, such as amyotrophic lateral sclerosis and dementia as well as POAG [224]	▲
64	*GSTP1* (2950)	within a biomedical cohort-based proteomic study [226]: GSTP1 downregulation in aqueous humour is a biomedical molecular marker for POAG	▼	within a phytopharmacological dietary wolfberry study [227]: GSTP1 upregulation can improve retinal mitochondrial biogenesis that may alleviate POAG	▲
65	*GSTT1* (2952)	in a cohort-based biomedical study [228]: GSTT1 is one of the major differentially hypermethylated genes, hence one of the lowest differential expressions in African Americans with metabolic syndrome compared to those without it, while metabolic syndrome is a risk factor for high intraocular pressure exacerbating POAG [229]	▼	within a comprehensive meta-analysis of transcriptomes from patients with and without glaucoma, verified using qPCR [230]: GSTT1 excess is a biomedical molecular marker for POAG	▼
66	*HAMP* (57817)	within human POAG cellular models using human the trabecular meshwork cells cultivated with HAMP-antagonists [231]: beneficial therapeutic effects in POAG	▲	within a biomedical cohort-based study [232]: *HAMP* excess in the serum and aqueous humor of patients is a biomedical molecular marker for POAG	▼

Table S2. *Cont.*

No	NCBI Gene *Symbol* (ID)	Effect of changes in human gene expression on the development of primary open-angle glaucoma (POAG, ☼: "▼" aggravation, "▲" alleviation) [Reference]			
		Deficit (↓)	☼ ▼ ▲	Excess (↑)	☼ ▼ ▲
67	*HDAC6* (10013)	within human disease models using rats subjected with an artificial retinal ischaemia/reperfusion injury [233]: HDAC6-inhibitor tubacin elevated survival of the retinal ganglion cell in POAG	▲	according to comprehensive review [234]: HDAC6 excess can improve microtubule dynamics beneficial for axonal growth, as well as supervise and coordinate stress response including stress granule formation, chaperone upregulation, inflammatory tolerance increase and facilitates the autophagy of both misfolded proteins and protein misaggregates leading to neurodegeneration, that altogether can alleviate POAG	▲
68	*HES1* (3280)	within human POAG cellular models using primary human trabecular meshwork cells subjected with oxidative stress along with a transfection of plasmid carrying shRNA against human *HES1* gene [235]: rescued POAG-related defects	▲	within human POAG cellular models using primary human trabecular meshwork cells subjected with oxidative stress [235]: *HES1* upregulation leading POAG-related defects	▼
69	*HSPA4* (3308)	within human diseases models using *Hspa4*-deficient mice [236]: enhanced apoptosis in inflammatory bowel disease comorbid with POAG [237]	▼	within human disease cellular models using murine cells transfected with plasmid carrying an additional *Hspa4* gene copy [238]: Hspa4 excess can protect cells against oxidative damage that may prevent an important pathogenetic step in POAG [239]	▲
70	*HSPA5* (3309)	within a pharmaceutical study on the dexamethasone side effects during anti-inflammatory treatment using human trabecular meshwork cells [240]: HSPA5 deficit can increase both outflow resistance and intraocular pressure that may aggravate POAG	▼	within a pharmaceutical study using rats subjected with valproate [241]: Hspa5 upregulation, which can protect retina against ischemia-reperfusion injury and, thereby, retard POAG development	▲
71	*IGF1R* (3480)	within human POAG cellular models using rat retinal ganglion cell line RGC-5 transfected with lentiviral vector carrying mircoRNA 100 (miR-100) targeted to *Igf1r* silencing under H2O2-caused oxidative stress [242]: Igf1r-deficit kept retinal ganglion cell against apoptosis	▲	within human POAG cellular models using rat neonatal retinal ganglion cells [243]: etidronate upregulates *Igf1r* promoting neurite growth and retinal ganglion cell survival	▲
72	*IL10* (3586)	within a biomedical cohort-based study [244]: valproic acid downregulated IL10 that can prevent surgery-induced conjunctival inflammation, which is a post-trabeculectomy complication in POAG [245]	▲	within human POAG models using mice subjected with spinal cord injury, which downregulated Il10 according to a proper measurement in vivo, and, next, treated with nanoparticles of curcumin as a new promising natural phytomedication with neuroregenerative activity under moder nanopharmacutical study [246]: excess IL10 along with successful neuroregeneration, which has been confirmed through several independent behavioral tests, and may alleviate POAG according to a comprehensive biomedical review [247]	▲
73	*IL1A* (3552)	according to a comprehensive pharmaceutical review [248]: phytopharmaceuticals containing carotenoids and polyphenols can downregulate *IL1A* due to their antioxidant and anti-inflammatory activities for adjuvant and preventive therapy in the elderly against POAG and other eye diseases	▲	within human POAG cellular models using human trabecular meshwork cell line TM-1 transfected with plasmid vector carrying mutant glaucomatous variants of *MYOC* gene [249]: *IL1A* upregulation as a candidate molecular marker for POAG	▼
74	*IL1B* (3553)	within human eye disease models using mice [250]: Il1b-downregulation can reduce apoptosis of retinal ganglion cells that may relieve POAG	▲	within human POAG cellular models using human trabecular meshwork cell line TM-1 transfected with plasmid vector carrying mutant glaucomatous variants of *MYOC* gene [249]: *IL1B* upregulation as a candidate molecular marker for POAG	▼
75	*IL1RN* (3557)	within human disease models using *Il1rn*-knockout mice retinal pigment epithelial cells [251]: reduced suppression of antigen-presenting mature dendritic cells, that can alleviate autoimmune response in POAG [252]	▲	within human disease models using transgenic mice carrying an additional *Il1*rn gene copy [253]: resistant to herpetic stromal keratitis as a complication of POAG treatment with drugs that can suppress the immune system [254]	▼
76	*IL2* (3558)	within human disease models using mice subjected with artificially induce ocular hypertension and, next, treated with the Tempol medication [255]: relieved POAG along with Il2-downregulation	▲	within a biomedical cohort-based study [256]: IL2 excess in lacrimal fluid is a biomedical molecular marker for POAG	▼

Table S2. *Cont.*

No	NCBI Gene *Symbol* (ID)	Effect of changes in human gene expression on the development of primary open-angle glaucoma (POAG, ☼: "▼" aggravation, "▲" alleviation) [Reference]			
		Deficit (↓)	☼ ▼ ▲	Excess (↑)	☼ ▼ ▲
77	*IL6* (3569)	within a biomedical cohort study [257]: IL6 deficit in trabecular meshwork is a biomedical molecular marker for trabecular meshwork fibrosis in POAG	▼	within a biomedical cohort-based study [258]: IL6 excess is a biomedical molecular marker for POAG	▼
78	*IL6R* (3570)	according to an exhaustive review [259]: the anti-IL6R humanized monoclonal antibody tocilizumab is a new promising drug for rheumatoid arthritis, a risk factor for which is POAG [194]	▲	within a cohort-based biomedical study [257]: IL6R excess in trabecular meshwork is a biomedical molecular marker for trabecular meshwork fibrosis in POAG	▼
79	*ITIH1* (3697)	according to an exhaustive retrospective analysis of tumor-related transcriptomes [260]: ITIH1-deficit may be a biomedical molecular marker for breast tumors, a side effect of treatment of which using tamoxifen may be the tamoxifen-associated maculopathy aggravating POAG [167]	▼	within human disease models using mice [261]: Itih1-excess can exacerbate systemic insulin resistance, which may contribute to POAG development [262]	▼
80	*LDLR* (3949)	within human disease models using Ldlr-knockout mice [263]: susceptibility to accelerated atherogenesis, which can aggravate POAG [143]	▼	within a biomedical cohort-based study [264]: LDLR excess is a biomedically proven molecular marker for pterygium occurrence and progression, which blocks light exposure from the cornea to the retina and, thus, may reduce retinal activity and metabolic demands leading to a decreased vascular density in fundus that altogether can aggravate POAG [265]	▼
81	*LOXL1* (4016)	according to an exhaustive review [266]: LOXL1 deficit can promote elastotic processes predisposing to extracellular matrix defects in the late stages of POAG	▼	within a cohort biomedical pharmaceutical study on the medication treatment of pseudoexfoliation syndrome as an age-related POAG complication [267]: in comparison with conventionally healthy volunteers, patients had hypermethylation of the *LOXL1* gene and, thus, its underexpression, whereas *LOXL1* upregulation due to its hypomethylation resulted from treatment of these patients with azacitidine as a promising candidate adjuvant medication that may alleviate POAG in old age	▲
82	*LRRC27* (80313)	within a biomedical cohort-based study [268]: LRRC27 hypermethylation reducing its expression is a biomedical molecular marker for resistance to low-fat/low-carbohydrate Mediterranean diet together with physical activity as a treatment for obesity, which is positively correlated with POAG [115]	▼	within a biomedical cohort-based study of patients with preeclampsia in comparison with patients with gestational hypertension [269]: LRRC27 excess is a biomedical molecular marker for preeclampsia with thrombosis, vascular and endothelial dysfunction, the last of which is successfully treated using Tanakan in POAG as its relief [270]	▼
83	*LTBP2* (4053)	within biomedical studies of Pakistani families and Gypsy patients [271]: *LTBP2*-null is a biomedical molecular marker for POAG	▼	within human glaucoma tissue models using mice with the interference RNA-impaired ciliary zonule formation leading lens luxation as POAG symptom and, next, treated with exogenous recombinant purified Ltbp2 within mice eye tissue level ex vivo [272]: restored unfragmented and bundled ciliary zonules to almost their norms	▲
84	*MFN1* (55669)	within human disease models using Mfn1/Mfn2-double-knockout mice subjected with artificial acute cardiac ischemia-reperfusion [273]: mitochondrial dysfunction leading post-ischemic cardiac arrhythmogenesis, which may also occur as a complication of POAG treatment with latanoprost [274]	▼	within human ocular disease models using mice [275]: Mfn1 upregulation can protect against mitochondrial damage in lens epithelial cells under light-induced oxidative stress inevitable at the eye vital function, that may alleviate POAG	▲
85	*MFN2* (9927)	within a human disease model using Mnf2-knockout mice subjected to artificial ischemia/reperfusion injury of the retina [276]: resistance to metformin-based treatment of retinal ischemia/reperfusion injury, which can exacerbate POAG	▼	within a human POAG model using mice [277]: during glaucomatous neurodegeneration, retinal ganglion cells can selectively accumulate Mfn2-excess in a phosphorylated form	▼
86	*MLIP* (90523)	within human age-related disease models using *Mlip*-knockout mice [278]: impaired cardiac adaptation, which is comorbid with POAG, at least within the framework of age-related disorders	▼	within human health models using proteomics analysis of the high versus low body mass male Hu sheep of [279]: Mlip excess is an proven economically valuable molecular marker for fat-tailed sheep breeding, while obesity may aggravate POAG [115]	▼

Table S2. *Cont.*

No	NCBI Gene *Symbol* (ID)	Effect of changes in human gene expression on the development of primary open-angle glaucoma (POAG, ☼: "▼" aggravation, "▲" alleviation) [Reference]			
		**Deficit (↓)**	☼ ▼ ▲	**Excess (↑)**	☼ ▼ ▲
87	*MMP1* (4312)	within a cohort-based biomedical study [280]: pilocarpine-based treatment in POAG can accelerate with a decrease in MMP1 level	▲	within a cohort-based biomedical study [281]: MMP1 excess in aqueous humor can be a biomedical molecular marker for susceptibility to POAG	▼
88	*MMP2* (4313)	within human glaucoma models using Mmp2-null mice [282]: resistance to retinal neovascularization aggravating POAG as well as attenuated retinal ganglion cell death and suppressed tumor necrosis factor, which may protect against neurodegeneration and neuroinflammation, respectively	▲	within a cohort-based study cohorts: in both aqueous humor [283] and conjunctival stroma [280], MMP2 excess is a biomedical molecular marker for POAG	▼
89	*MMP9* (4318)	within human glaucoma models using transgenic Mmp9-deficient mice [284]: elevated intraocular pressure aggravating POAG	▼	within an observational pharmaceutical study using tear sample collection [285]: latanoprost, being a commonly accepted medication against POAG, can simultaneously both upregulate MMP9 and reduce intraocular pressure that can alleviate POAG	▲
90	*MMP12* (4321)	within human glaucoma models using Mmp12-knockout mice [286]: decreased levels of both adhesion molecule and inflammatory cytokines along with reduced vascular leakage in oxygen-induced retinopathy, as well as markedly reduced macrophage content in the retina with impaired macrophage migratory capacity, attenuated retinal capillary dropout and mitigated pathological retinal neovascularization that all improvements to POAG were reproduced using normal mice subjected with MMP408 as pharmacological inhibitor of MMP12	▲	within a cohort-based biomedical study [281]: MMP12 excess in aqueous humor can be a biomedical molecular marker for susceptibility to POAG	▼
91	*MPP7* (143098)	within cohort-based study together with POAG models using human trabecular meshwork cells that was verified using murine eye subjected with mechanical stress mimicking the POAG development scenario [287]: MPP7 deficit can be a biomedical molecular marker for susceptibility to POAG	▼	within POAG models using human trabecular meshwork cells [112] along with an observations within cancer-derived xenograft mice [288]: MPP7 excess can stimulate autophagy that can reduce ocular hypertension as a relief in POAG	▲
92	*MTHFR* (4524)	according to an exhaustive review [289]: Mthfr-deficient mice are an animal model for human hyperhomocysteinemia as a risk factor for both endothelial dysfunction and POAG [290]	▼	within a cohort-based study [291]: 3 weeks of yoga practice resulted in fivefold upregulation in the expression of MTHFR gene, whereas MTHFR-deficit may be a molecular marker for ocular defects according to human ocular defect models using zebrafish larvae [292]	▲
93	*MUTYH* (4595)	within human age-related disease models using mice aged 5 to 22 months [293]: Mutyh levels decrease with increasing age, which may contribute to the progression of age-related diseases, including POAG	▼	within comparative human disease models using Mutyh-deficient mice versus normal mice [294]: oxidative activation of microglia increases with increasing levels of Mutyh, leading to retinal degeneration as a progression of POAG [295]	▼
94	*MYOC* (4653)	within a pharmaceutical research [296]: small molecule MYOC-inhibitors (e.g., GW5074 and apigenin) reduced pathogenic MYOC aggregation that can enhance normogenic MYOC secretion and thus alleviate POAG	▲	within human cellular POAG models using either human retinal pigment epithelium cell line RPE8319, human embryonic kidney cell line HEK293, and human trabecular meshworkcell line HTM [297]: 17β estradiol-inducible MYOC upregulation might have a potential implication POAG pathogenesis	▼
95	*NCKAP5* (344148)	according to the case report of a 69-year-old man [298]: suprachoroidal effusion aggravated surgery for POAG, most likely accompanied by NCKAP5 deficit, which is a biomedical molecular marker for non-small cell lung cancer as a well-known risk factor of suprachoroidal effusion [299], which, indeed, was in the above-mentioned 69-year-old man	▼	according to the case report of a 68-year-old woman [300]: after surgery for POAG, Staphylococcus aureus caused infective necrotizing scleritis, most likely accompanied by NCKAP5 upregulation because of hypo-methylation of the Nckap5 mouse gene was found in Staphylococcus aureus-induced mastitis as human disease models using mice [301]	▼
96	*NOS2* (4843)	within a phytopharmaceutical study [302]: resveratrol as natural phytoantioxidant with antibacterial and antifungal protective properties can suppress NOS2 to medicate POAG	▲	within cohort-based iris sample postmortem study [303]: both excess and hyperactivity of NOS2 can be a biomedical molecular marker for susceptibility to POAG	▼

Table S2. *Cont.*

No	NCBI Gene *Symbol* (ID)	Effect of changes in human gene expression on the development of primary open-angle glaucoma (POAG, ☼: "▼" aggravation, "▲" alleviation) [Reference]			
		**Deficit (↓)**	☼ ▼ ▲	**Excess (↑)**	☼ ▼ ▲
97	*NOS3* (4846)	within human disease models using NOS3 knockout mice [304]: higher risks of both hypertension and atherosclerosis, while both atherosclerosis [143] and hypertension [9] can aggravate POAG	▼	within a cohort-based biomedical study [305]: positive therapeutic effects of mindfulness meditation on intraocular pressure and trabecular meshwork in patients in India with medically uncontrolled primary open angle glaucoma was accompanied by NOS3 upregulation	▲
98	*NRG2* (9542)	within human disease models using Nrg2-knockout mice [306]: reduced reproductive capacity and early growth retardation, while in dogs artificial selection for short stature may have contributed to increased prevalence of POAG [307]	▼	within human neurodegenerative disease models using rat pups subjected with an interference-RNA-caused delaying neurogenesis, an, next, treated with retrovirus carrying an additional copy of the rat Nrg2 gene [308]: Nrg2 excess along rescued the impairments of dendritic arborization, whereas this repairative neurogenesis can alleviate the post-COVID-infection effects on POAG [309]	▲
99	*NTF4* (4909)	according to comprehensive review [310]: many POAG-related NTF4-mutation experiments *in vitro* have indicated that neurotrophin-4 failure can contribute to POAG pathogenesis	▼	within human cellular POAG-related post-traumatic wound healing models using telomerase-immortalized human corneal epithelial cell line hTCEpi between passages 20 and 28 [311]: activated stromal fibroblast conditioned medium can elevate NTF4 level to repair eye injuries	▲
100	*NTM* (50863)	within a cohort-based biomedical microarray study [312]: NTM-deficit seems to be a biomedical molecular marker for ischemic diseases, which occur significantly more often in patients with POAG than without it [313]	▼	within women disease models using adult virgin female Spague-Dawley rats [314]: estrogen upregulates *Ntm* along with neuroprotection for retinal ganglion cells within the optic nerve that can alleviate POAG as an additional benefit during maintenance postmenopausal estrogen-based therapy according to comprehensive review [315]	▲
101	*NTRK2* (4915)	according to exhaustive review [316]: compared to normal, Ntrk2 knockout mice have accelerated photoreceptor degeneration and loss of retinal ganglion cells, as well as more severe damage to the optic nerve	▼	within a cohort-based retrospective biomedical study [317]: total healing effect of drugs used to treat glaucoma associated with retinal TrkB upregulation as neuroprotector	▲
102	*OGG1* (4968)	within a cohort-based biomedical study [137]: OGG1-deficit is a biomedical molecular marker of POAG because of the corresponding decrease in the level of excision repair of somatic pre-mutational DNA damage caused by oxidative stress	▼	within POAG cellular models using conjunctival fibroblasts under short-term cold atmospheric pressure plasma as a promising effective rapid pre-operative disinfectant for eye surfaces without damages [318]: within the day after such disinfection, OGG1 excess induced excision repair of pre-mutational DNA damages following oxidative stress	▲
103	*OPA1* (4976)	according to a cohort-based biomedical study using qPCR [319]: OPA1-deficiency is a biomedical molecular marker for susceptibility to POAG	▼	within human POAG models using rats [320]: *Opa1* upregulation may protect retinal ganglion cells by ways of enhancing mitochondria fusion, which may provide a useful strategy to battle against POAG	▲
104	*OPTC* (26254)	within cohort-based biomedical POAG study using RT-qPCR [321]: OPTC deficiency occurs with reduced visual acuity, visual field defects near fixation point in the infero-temporal quadrant and tubular vision as POAG symptoms	▼	within human POAG models using mice subjected with recombinant opticin injection into eyes [322]: inhibited pathologic preretinal neovascularization within the normally avascular mature vitreous that can alleviate POAG	▲
105	*OPTN* (10133)	according to an exhaustive review [323]: OPTN-dysfunction can lead mitochondrial degradation, accumulation of damaged mitochondria and defective mitophagy in the axons, which taken altogether can aggravate POAG	▼	within an exhaustive review [323]: OPTN excess in retinal ganglion cells may enhance mitochondrial biogenesis, fission, and volume density that may alleviate POAG	▲
106	*PADI2* (11240)	according to comprehensive review [324]: in the contrary to age-related neurodegeneration, both retinal deimination and PADI2 expression decrease with age during healthy aging	▲	within cohort-based post-mortem studies [325]: PADI2 excess in the optic nerve can be a biomedical molecular marker for susceptibility to POAG	▼
107	*PARP1* (142)	within human POAG models using Parp1-knockout mice [137]: increases resistance to retinal neurodegeneration without affecting retinal function	▲	within cohort-based elderly women biomedical study [326]: smoking elevates PARP1 level that can speed up both apoptosis and POAG	▼

Table S2. *Cont.*

No	NCBI Gene *Symbol* (ID)	Effect of changes in human gene expression on the development of primary open-angle glaucoma (POAG, ☼: "▼" aggravation, "▲" alleviation) [Reference]			
		Deficit (↓)	☼ ▼ ▲	Excess (↑)	☼ ▼ ▲
108	*PLXDC2* (84898)	within a cohort-based biomedical studies [327]: PLXDC2 deficiency is a biomedical molecular marker for lesser risks of ischemic stroke, which is comorbid to POAG [328]	▲	within a cohort-based biomedical studies [327]: PLXDC2 excess is a biomedical molecular marker for susceptibility to ischemic stroke, which is comorbid to POAG [328]	▼
109	*PMM2* (5373)	within a cohort-based biomedical study [329]: PMM2 deficiency is a biomedical molecular marker for congenital glycosylation disorders aggravating POAG [330]	▼	exogenous recombinant insulin injections can both upregulate *PMM2* within human disease cellular models using COS-7 cells [331] and relieve POAG according to an exhaustive review [332]	▲
110	*PON1* (5444)	according to an exhaustive review [333]: fluoride contributes to many eye diseases including POAG through reducing PON1 and antioxidant levels	▼	within a cohort-based biomedical study [334]: PON1 excess in blood is a biomedical molecular marker for susceptibility to POAG	▼
111	*PPARG* (5468)	within a cohort-based biomedical study [335]: difference between intraocular and intracranial pressures is among main risk factors of both primary open-angle glaucoma and Graves' orbitopathy, while PPARG-deficit is a biomedical molecular marker for Graves' orbitopathy [336]	▼	within human disease models using mice [337]: magnolol as principal ingredient of *Magnoliae officinalis* cortex, has anti-inflammatory and neuroprotective effects and relieves retinal damages and inflammation due to, mainly, Pparg-upregulation, which altogether can alleviate POAG	▲
112	*PRNP (5621)*	within human disease models using *Prnp*-null mice [338]: increased intraocular pressure aggravating POAG	▼	within human eye disease models using proteome of neuroretina from both mouse and rat [339]: Prnp overexpression modulated by heat shock proteins as an attribute of a protective role of Prnp in the neuroretinal cellular stress response that may alleviate POAG	▲
113	*PTGFR* (5737)	within a cohort-based biomedical study [340]: the effectiveness of the response to latanoprost as a drug against POAG decreases with a decrease in the transcriptional activity of the PTGFR gene as measured experimentally [341]	▼	according to an exhaustive pharmaceutical review [342]: artificial PTGFR-agonists are drugs against POAG	▲
114	*PTGS1* (5742)	within human disease models using Ptgs1-knockout mice [343]: prostaglandin deficit in the corneal tissue, uveoscleral tissue, lens, retina and optic nerve, whereas exogenous recombinant prostaglandins and their synthetic analogues (e.g., latanoprost) are drugs against POAG [344]	▼	within human POAG pharmaceutical cellular models using human embryonic kidney cell line HEK-293 treated with an artificial low molecular weight analog AGN008 of the human prostaglandin E receptor 4, PTGER4, as a new promising anti-POAG medication [345]: *PTGS1*-upregulation along with reduced intraocular pressure	▲
115	*RHOA* (387)	according to an exhaustive review [346]: drug-induced therapeutic inhibition of RHOA can decrease aqueous humor drainage via the trabecular meshwork in the drainage pathway that can alleviate POAG	▲	within human neuroregeneration models using rats subjected with artificial sciatic transection injuries [347]: post-injury activated GTP-bound RhoA dramatically upregulated in both neurons and axons, which shown a RhoA-dose-related increase in the initiation of neurite outgrowth, and in the proportion with long neurites in the extent and distance of axons, and partnered Schwann cell regrowth	▲
116	*ROCK1* (6093)	within human POAG cellular models using spontaneously arising retinal pigment epithelial cell line ARPE-19 [348]: miR-136-5p inhibits ROCK1-expression that relieves inflammatory retina injury in POAG	▲	according to an exhaustive pharmaceutical review [349]: netarsudil as a low molecular weight ROCK1-antagonist is an eye drop medication against POAG	▼
117	*ROCK2* (9475)	within a pharmaceutical study using mice [350]: fasudil as a low molecular weight ROCK2-inhibitor is medication against POAG as well as against hypertension, heart failure, stroke, brain and spinal cord injuries	▲	within a cohort-based biomedical study [351]: ROCK2 excess in the human lens epithelial cells seems to be a biomedically proven molecular marker for increased protein aggregates aggravating POAG	▼
118	*SEC14L2* (23541)	within human disease cellular models using human SEC14L2-knockout COS-7 cell line generated based on CRISPR–Cas9 technology [352]: impaired endosome fission and accumulation that can cause age-related exfoliation syndrome aggravating POAG in old age [353]	▼	within the pharmaceutical search for promising cell cultures as an *ex vivo* test-system for the design of vaccines against the hepatitis C virus [354]: *SEC14L2* overexpression can increase vitamin E levels, which may alleviate POAG [355]	▲

Table S2. *Cont.*

No	NCBI Gene *Symbol* (ID)	Effect of changes in human gene expression on the development of primary open-angle glaucoma (POAG, ☼: "▼" aggravation, "▲" alleviation) [Reference]			
		**Deficit (↓)**	☼ ▼ ▲	**Excess (↑)**	☼ ▼ ▲
119	*SERPINE1* (5054)	within human POAG cellular model using human trabecular meshwork cells HTM cultivated and treated with TGF-beta2 relieving POAG [356]: increased fibrillar extracellular matrix aggravating POAG along with serpin E1 deficit relatively to those at BMP7 treatment alleviating POAG	▼	according to a cohort-based biomedical study [357]: serpin E1 excess in intraocular aqueous humor can be a biomedical molecular marker for susceptibility to POAG	▲
120	*SIRT1* (23411)	within cohort-based biomedical trabeculectomy study using RT-qPCR [358]: Sirtuin 1 deficiency in the trabecular meshwork tissue can be a biomedical molecular marker for susceptibility to POAG	▼	within human neuroprotection models using C57BL/6 mice infected with a neurotropic strain of mouse hepatitis virus MHV-A59 inducing a multiple-sclerosis-like neuronal loss and, next, treated orally with an artificial compound SRTAW04 as a new promising neuroprotector [359]: Sirt1 upregulation along with prevented neuronal loss during inflammatory demyelinating optic nerve lesion that can neuroprotectantly alleviate POAG	▲
121	*SIX1* (6495)	within human POAG models using transgenic mice [360]: Six1 deficiency in eye can be a biomedical molecular marker for susceptibility to POAG	▼	according to an exhaustive biomedical review [361]: *SIX1* overexpression can slow down the apoptosis as the most important event in retinal ganglion cells during therapy against POAG in line with a cohort-based biomedical study [362]	▲
122	*SIX6* (4990)	within a cohort-based biomedical gene copy number variation study of using microarrays of blood [30]: the only SIX6-deletion case happens in POAG	▼	within human neuroregeneration models using embryonic chick retinal pigmented epithelium subjected with an artificial injury [363]: SIX6 excess along with transient dedifferentiation of retinal pigmented epithelium reprogrammed to neuroepithelium, which can differentiate to neural retina cells as neuroregeneration alleviating POAG	▲
123	*SLC4A10* (57282)	within human disease models using Slc4a10-deficient mice [364]: impaired visual function in retina that can aggravate POAG	▼	according to a comprehensive biomedical review [365]: a contrast sensitivity level decreases with a decrease Slc4a10 level within a mice model of POAG as well as a visual acuity level increases with an increase in contrast sensitivity level within a cohort-based biomedical study [366]	▲
124	*SLC23A2* (9962)	according to a cohort-based biomedical study [367]: SLC23A2 deficit in tears, aqueous humor, and blood is a biomedical molecular marker for susceptibility to POAG	▼	within human POAG cellular models using an immortalized human lens epithelial cell line HLE-B3 under exposure to the chemical oxidant tert-butylhydroperoxide [368]: an immediate SLC23A2 up-regulation improving Na+-dependent transport of vitamin C (i.e., ascorbic acid) as the most important antioxidant into the normal lens epithelium in order to prevent eye damages under oxidant stressors	▲
125	*SLCO2A1* (6578)	within human health care models using rat type 1 alveolar epithelial cell-like cell line AT1-L subjected with cigarette smoke extract [369]: Slco2a1-deficit can elevate susceptibility to oxidant stress, which can lead to detrimental effects on ocular health, namely: POAG, cataract, and age-related macular degeneration as three most often causes of vision loss [370]	▼	within a cohort-based biomedical study of latanoprost therapy efficiency, finding of which were verified by means of human POAG cellular models using human embryonic kidney cell line HEK293 overexpressing *SLCO2A1* due to an additional gene copy transfected [371]: improved latanoprost therapeutic effect against POAG	▲
126	*SPARC* (6678)	within human disease models using Sparc-knockout mice [372]: Sparc-null mice exhibit lower intraocular pressures, while the best way to manage POAG is by lowering the intraocular pressure [373]	▲	within cohort-based biomedical RNA-Seq study using postmortem donor ocular tissue within and without POAG in donor's anamnesis [374]: SPARC-excess seems to be a biomedically proven molecular marker for POAG	▼
127	*SPP1* (6696)	within human Alzheimer's disease models using transgenic mice carrying microglia-specific miR-155 knockdown [375]: Spp1 downregulation mitigated retinal inflammation and vasculopathy as protective effects against vascular damages during Alzheimer's disease and associated neurodegenerative ocular diseases, including POAG	▲	within a cohort-based biomedical RNA-Seq analysis of human corneoscleral tissues taken from postmortem human donor eyes with versus without glaucoma [376]: SPP1-excess can be a biomedical molecular biomarker of POAG	▼

Table S2. *Cont.*

No	NCBI Gene *Symbol* (ID)	Effect of changes in human gene expression on the development of primary open-angle glaucoma (POAG, ☼: "▼" aggravation, "▲" alleviation) [Reference] Deficit (↓)	☼ ▼ ▲	Excess (↑)	☼ ▼ ▲
128	*STAT3* (6774)	according to an exhaustive mini-review letter [377]: *STAT3* gene silencing can contribute to both origin and development of POAG and its severity among patients with diabetes	▼	within human POAG model using rats subjected with a chronic artificially elevated intraocular pressure [378]: STAT3 overexpression in the optic nerve head seems to be a candidate molecular marker for the very earlier stages of the POAG origin and development	▼
129	*TAP1* (6890)	within human disease cellular models using the human melanoma cells cultured with the synthetic nanomeric peptide IT9302 homologous to the C-terminal of the human IL-10 (i.e., AYMTMKIRN) [379]: TAP1 inhibited along with a IT9302-dose-dependent increase in sensitivity to lysis by natural killer cells of anticancer innate immune response to melanoma, which is comorbid to POAG in line with a comprehensive review [380]	▲	within human diabetes mellitus models using mice [381]: Tap1 overexpression may be a candidate molecular marker for diabetes mellitus, which is comorbid with POAG, at least due to their mutual comorbidity with diabetic retinopathy [198]	▼
130	*TAP2* (6891)	within human disease cellular models using the human melanoma cells cultured with the synthetic nanomeric peptide IT9302 homologous to the C-terminal of the human IL-10 (i.e., AYMTMKIRN) [379]: TAP2 inhibited along with a IT9302-dose-dependent increase in sensitivity to lysis by natural killer cells of anticancer innate immune response to melanoma, which is comorbid to POAG in line with a comprehensive review [380]	▲	within human transcriptome meta-analysis related to inflammatory bowel disease, most frequent among which are Crohn's disease and ulcerative colitis [382]: TAP2 excess along with abnormal immune response to intestinal tract microbiota that can provoke through microbiota-gut-retina axis autoimmunity impact to POAG pathogenesis [383]	▼
131	*TBK1* (29110)	according to an exhaustive retrospective review [384]: TBK1-inhibitors are considered as promising drugs for adjuvant therapy in POAG	▲	within human antiviral innate immune response models using duck embryonic fibroblasts transfected with a recombinant plasmid with or without another copy of the Tbk1 gene and 24 hours later incubated with duck enteritis virus [385]: excess Tbk1 may enhance the antiviral innate immune response, which may prevent viral conjunctivitis as a dangerous complication after glaucoma surgery [386]	▲
132	*TCF4* (6925)	within human disease models using neuron-specific *Tcf4*-knockdown adult mice (because of Tcf4-knockout animals are perinatally lethal) [387]: higher risks of both hyperexcitability and increased dendritic complexity of neurons contributing schizophrenia, which is comorbid to POAG as age-related neurodegenerative diseases [388]	▼	according to a cohort-based biomedical study using postmortem donor corneas obtained from the eye bank of Transplant Services [389]: higher risks of Fuchs' endothelial corneal dystrophy comorbid to POAG [124]	▼
133	*TGFB2* (7042)	within human POAG treatment models using mice subjected with the ocular eye drops containing astragaloside IV, which is a novel saponin isolated from the roots of Astragalus membranaceus as a promising medication against POAG [390]: the Tgfbr2 inhibition reduces intraocular pressure that can alleviate POAG	▲	within human POAG models using mice injected with adenoviral vector carrying an human TGFBR2 gene in addition to their own mice Tgfbr2 gene [390]: TGFBR2-excess can elevate intraocular pressure that can aggravate POAG	▼
134	*TGFBR3* (7049)	within a cohort-based biomedical RNA-Seq analysis of human Schlemm's canal endothelial cells isolated from eight different postmortem human eyes with versus without glaucoma [391]: TGFBR3-deficit can be a biomedical molecular marker of POAG	▼	within a cohort-based biomedical cellular study using orbital fibroblasts isolated from specimens obtained intraoperatively from patients with versus without thyroid-eye disease, who underwent decompressive surgery [392]: TGFBR3 excess can prevent fibrosis, which is a complication of POAG [393]	▲
135	*TIMP1* (7076)	within a cohort-based biomedical qPCR-analysis of specimens of Tenon capsule obtained intraoperatively from patients with versus without POAG [394]: TIMP1-deficit as a biomedical molecular marker of POAG	▼	within human neuroprotection cellular models using mice BV2 microglial cell culture subjected with ursolic acid as a promising neuroprotector being intensively studied pharmaceutically now, that resulted in reliably detected anti-apoptotic neuroprotective changes, which were next reversed back by a TIMP1 neutralizing antibody (anti-TIMP1) [395]: TIMP1 excess has anti-apoptotic neuroprotective abilities that may slow the progression of POAG	▲

Table S2. *Cont.*

No	NCBI Gene *Symbol* (ID)	Effect of changes in human gene expression on the development of primary open-angle glaucoma (POAG, ☼: "▼" aggravation, "▲" alleviation) [Reference]			
		Deficit (↓)	☼ ▼ ▲	Excess (↑)	☼ ▼ ▲
136	*TIMP2* (7077)	within human POAG model using rats, who were first subjected to artificial pathogenesis of POAG after which they were treated recombinant human decorin [396]: Timp2 downregulation along with both reduced intraocular pressure and retarded retinal ganglion cell loss that all can relieve POAG	▲	according to comprehensive review [397]: overexpression of TIMP2 in optic nerve using a lentiviral vector carrying another TIMP2 gene copy is a promising gene therapy strategy for axonal regeneration being intensively studied pharmaceutically to retard the progression of POAG	▲
137	*TIMP3* (7078)	within a cohort-based biomedical study [283]: TIMP3 deficiency is a biomedical molecular marker of POAG	▼	within a cohort-based pharmaceutical tissue study using trabecular meshwork, ciliary muscle, and scleral fibroblast separated from cadaver eyes within 6 hours postmortem [398]: both bimatoprost and latanoprost are commonly accepted eye medications against POAG, which can simultaneously both upregulate *TIMP3* and reduce the increased intraocular pressure	▲
138	*TIMP4* (7079)	within a cohort-based biomedical study [399]: sulfur mustard downregulates TIMP4 along with systemic eye damages aggravating POAG	▼	within a cohort-based biomedical study [283]: TIMP4 excess is a biomedical molecular marker of POAG	▼
139	*TLR2* (7097)	within human disease models using Tlr2-knockout mice [400]: susceptibility to experimental autoimmune diseases, while POAG is biomedically well-known risk-factor of autoimmune diseases [194]	▼	within human eye disease models using mice subjected with intravitreal injection of a Gram-positive spherically shaped bacterium Staphylococcus aureus [401]: *Tlr2*-upregulation in retinal Muller glia contributing to retinal innate defense via production of inflammatory mediators and antimicrobial peptides opposing infectious endophthalmitis following POAG filtration surgery, especially during the coronavirus disease (COVID-19) pandemic period [402]	▲
140	*TLR4* (7099)	within human POAG models using Tlr4-null mice [403]: resistance to both artificially induced POAG and elevated intraocular pressure	▲	according to a retrospective review [404]: TLR4-excess can elevate both trabecular meshwork cell fibrosis and retinal ganglion cell death that can aggravate POAG	▼
141	*TMCO1* (54499)	within a cohort-based biomedical study of gliomas and others TMCO1-related diseases [405]: TMCO1 dysfunction is closely related to a variety of human diseases, including glaucomas	▼	within a cohort-based biomedical micro-RNA comparative transcriptome study [406]: excessive hsa-miR-122-5p reducing TMCO1-target protein level in glaucomatous patients relatively its more higher norm in conventionally healthy volunteers can statistically significantly be a molecular marker for the exfoliation glaucoma, which is comorbide to POAG at least within their common biomedical associations with both decreased conventional aqueous humor outflow and increased intraocular pressure	▲
142	*TMTC2* (160335)	within a comprehensive biochemical study using RNA-interference targeted to mRNA transcribed from the human TMTC2 gene [407]: TMTC2 -knockdown elevates intracellular calcium release just like lanoprost does as a widely used anti-POAG medication [393]	▲	within a comprehensive biochemical study [407]: redox stress upregulates the human TMTC2 gene along with an acceleration in cellular senescence, which can contribute to POAG as an age-related disease in line with an exhaustive retrospective biomedical review [408]	▼
143	*TNF* (7124)	within human POAG models using Sprague-Dawley rats injected with hyaluronic acid into the anterior chamber of the eye during six-weeks and, next after POAG symptom detection, treated with myricetin as a promising phytomedication against POAG [409]: Tnf downregulation along with lowered levels in reactive oxidative species (ROS), lipid peroxidation products, pro-inflammatory cytokines and intraocular pressure that altogether can relieve POAG	▲	within human POAG emergence models using Sprague-Dawley rats injected streptozotocin in citric buffer and, next after diabetic symptom detection, rat retinal tissue samples were prepared and studied using RT-qPCR [410]: accelerated apoptosis of retinal ganglion cells along with Tnf excess, both of which can correspond to something like POAG emergence together with a promising molecular marker for early POAG	▼

Table S2. *Cont.*

No	NCBI Gene *Symbol* (ID)	Effect of changes in human gene expression on the development of primary open-angle glaucoma (POAG, ☼: "▼" aggravation, "▲" alleviation) [Reference]			
		Deficit (↓)	☼ ▼ ▲	Excess (↑)	☼ ▼ ▲
144	*TP53* (7157)	according to complex biomedical analysis of association between SNP rs4938723:C (i.e., ancestral allele "tttgacctatTacagctctca" => minor allele "tttgacctatCacagctctca") and POAG in Chinese population using luciferase assays, in-silicon analyses, real-time PCR, Western Blot, and flow cytometry and many other methods [411]: minor allele "C" can downregulate *TP53* expression, thus leading to suppressed cell apoptosis and enhanced cell proliferation that could reduce susceptibility to POAG	▲	according to a cohort-based biomedical study in Poland [412]: *TP53* upregulation can promote cell death through apoptosis during neurodegenerative processes aggravating POAG	▼
145	*TRPC6* (7225)	within human POAG cellular models using the human lamina cribrosa cells subjected with hydrogen peroxide as an oxidative stressor inducing POAG along with either small interference RNA targeted to *TRPC6* or low molecular weight TRPC6-inhibitor SKF96365 as a new promising medications against POAG [413]: *TRPC6* downregulation along with slowing down cell proliferation that can prevent fibrosis causing optic neuropathy in POAG	▲	within human eye disease models using adult male rats subjected to retinal ischemia induced by ophthalmic vessel ligation for 60 minutes, and after 5 minutes complete restored perfusion was found [414]: Trpc6 excess before ischemia-reperfusion injury has an early neuroprotective effect on retinal ganglion cells promoting their survival, which may alleviate POAG	▲
146	*TRPM5* (29850)	within human disease models using Trpm5-deficient mice [415]: increased susceptibility to bacterial infections that can aggravate POAG according to a biomedical case report [150]	▼	according to a nutritional study of natural herbal products that can stimulate people to a healthy lifestyle [416]: bitter melon extract upregulates *TRPM5* contributing to aversion to high salt concentrations that can prevent high salt provoking cardiovascular diseases, especially hypertension as a risk factor of POAG [417]	▲
147	*TXNRD2* (10587)	according to a comprehensive molecular biochemical review [418]: TXNRD2 deficiency can lead to an increase in mitochondrial reactive oxygen species (ROS) playing a key role in the pathogenesis of POAG [419]	▼	within human lifespan models using transgenic fruit flies overexpressing Txnrd2 verified with many independent transcriptome profiling data on long-lived primate and rodent species [420]: Txnrd2 excess is a biomedical molecular marker for susceptibility to longevity that seems to be fitting the intraocular pressure decrease with age increase in the human POAG model using transgenic mice [421]	▲
148	*VAV2* (7410)	within human POAG models using Vav2-deficient mice [422]: early onset of iridocorneal angle changes and elevated intraocular pressure, with subsequent selective loss of retinal ganglion cells and optic nerve head cupping, which are the hallmarks of POAG	▼	within human neurodevelopment models using transgenic Vav2 gain-of-function chicken (*Gallus gallus domesticus*) [423]: Vav2 excess in the chick spinal cord along with an improved limb trajectory of motor axon growth that might contribute to both neuroprotection and neuroregeneration during POAG according to an exhaustive biomedical review [424]	▲
149	*VAV3* (10451)	within the human disease models using *Vav3*-knockout mice [422]: a glaucoma-like phenotype that corresponds to SNP in Japanese patients with POAG	▼	according to an exhaustive review [425]: Vav3 activation may promote granule cell survival in the cerebellum that may slow the progression of POAG accompanied by neurodegeneration throughout the whole brain, especially severe in the cerebellum, oculomotor regions, visual cortices and frontal lobe, in line with a biomedical cohort-based study using magnetic resonance imaging [426]	▲
150	*VDR* (7421)	according to an exhaustive review [427]: Vdr-knockout mice are susceptible to hypertension aggravating POAG [9]	▼	within human disease cellular models using a fast-growing mice neuroblastoma cell line Neuro2a transfected with lentiviral vector carrying another Vdr gene copy [428]: amyloid precursor protein gene expression decreases with an increase in Vdr level that can reduce regional beta-amyloid peptide accumulations, which can block axonal transport as an initial step in retinal ganglion cell degeneration aggravating POAG according to human POAG models using mice injected with microbeads to increase intraocular pressure [429]	▲

Table S2. *Cont.*

No	NCBI Gene *Symbol* (ID)	Effect of changes in human gene expression on the development of primary open-angle glaucoma (POAG, ☼: "▼" aggravation, "▲" alleviation) [Reference]			
		Deficit (↓)	☼ ▼ ▲	Excess (↑)	☼ ▼ ▲
151	*VEGFA* (7422)	within human POAG models using rats injected with streptozotocin injection until diabetic symptoms observed, and, next, transfected with microRNA-15b targeted *Vegfa* into retinal capillary endothelial cells [430]: *Vegfa* downregulation along with reduced hyperproliferation of retinal capillary endothelial cells that can alleviate retinopathy in POAG [198]	▲	within human hypoxia eye adaptation models using mice exposed to either short- (6 hours), long- (48 hours) terms or chronic (11 weeks) normobaric hypoxia at 400 m or hypobaric hypoxia at 3450 m above sea level [431]: *Vegfa* upregulation at 5 of 6 statistically significant cases, with the only exception of a near-threshold trend in the case of chronic normobaric hypoxia, which overall indicates an attempt to improve oxygen delivery to both the optic nerve and retinal ganglion cells by increasing choroidal circulation, which may be something like neuroprotection supporting their survival as the alleviation of POAG	▲
152	*WDR36* (134430)	within human POAG cellular models using yeast expressing test-system with yeast *WDR38*-homologous gene *Utp21* carrying *WDR38*-allele associated with POAG [432]: WDR38 deficiency seems to be a promising candidate biomedical molecular marker for POAG in human	▼	within human POAG models using transgenic heterozygotous Wdr36-deficient glaucomatous mice overexpressing *Wdr36* [433]: Wdr36 excess along absence of POAG symptoms, which may argue for rather than against the protective properties of excess WDR36 against POAG	▲
153	*XRCC1* (7515)	within human disease models using murine neural-specific inactivation of Xrcc1 [434]: susceptibility to neurodegenerative diseases, one of which is POAG [224]	▼	within a biomedical cohort-based study using the tissue microarray technology [435]: XRCC1 excess is a biomedical molecular marker of the healthy glia cells adjacent the glioma cells because it exerts potent inhibitory effects on neovascularization, suppresses the tumorigenic behavior of glioma cells, and protects neural cells just as tetramethylpyrazine does, being herbal medication from *Ligusticum striatum* (Chuan Xiong) root within traditional Chinese medicine against both glioma and glaucoma [436]	▲

Note: Hereinafter, "biomedical" should be understood as "clinical" for POAG patients and as "experimental" for laboratory POAG models using human or animal cells, tissues and/or organs.

Table S3. Candidate SNP markers in the 90-bp proximal promoters of 153 human genes associated with POAG according to our *in silico* analysis.

#	Human Gene: *NCBI Gene Symbol* (NCBI Gene ID)	Candidate SNP marker: dbSNP ID:min [437]	5′ flank, 10 bp	WT →min	3 flank, 10 bp	K_D, nM, *in silico*: WT MEAN	± SEM	K_D, nM, *in silico*: min MEAN	± SEM	Significance: Z	p	ϱ	Δ	Effect of changes in human gene expression on the development of primary open-angle glaucoma (POAG, ☼: "▼" aggravation, "▲" alleviation) [Reference]	☼ ▲▼
1	*ABCA1* (19)	rs1457394998:C	cagccgaatc	T→C	tgcgctcggt	2.23	0.22	6.31	0.54	15.78	10^{-6}	A	↓	within human disease models using Abca1-null mice [76]: accelerated vision loss can worsen POAG	▼
		rs1826500736:C	gccgaatcta	T→C	cgctcggtgc	2.23	0.22	6.12	0.59	14.5	10^{-6}	A			
		rs886063317:C	agccgaatct	A→C	gcgctcggtg	2.23	0.22	9.87	0.90	21.9	10^{-6}	A			
		rs886063317:G	agccgaatct	A→G	gcgctcggtg	2.23	0.22	7.05	0.66	16.69	10^{-6}	A			
		rs1008606492:G	gcagccgaat	C→G	ctgcgctcgg	2.23	0.22	1.80	0.19	2.96	10^{-2}	C	↑	in a cohort-based biomedical study [77]: ABCA1 excess is a biomedical molecular marker for POAG	
		rs960653280:A	tgcagccgaa	T→A	tctgcgctcg	2.23	0.22	1.71	0.17	3.76	10^{-3}	B			
2	*ABCB1* (5243)	rs1160560450:C	tctttgccac	A→C	ccgcttcgct	56.75	3.86	81.24	5.94	7.19	10^{-6}	A	↓	within a biomedical pharmacological study using radiolabeled verapamil [78]: for this adjuvant anti-glaucoma medication [79], an ABCB1 inhibitor increased influx to and decreased outflow from the retina across the blood-retinal barrier	▲
		rs1160560450:G	tctttgccac	A→G	ccgcttcgct	56.75	3.86	81.24	5.98	7.16	10^{-6}	A			
		rs1160560450:T	tctttgccac	A→T	ccgcttcgct	56.75	3.86	72.51	6.00	4.58	10^{-3}	B			
		rs1819428060:C	cgctctcttt	G→C	cacagccgct	56.75	3.86	65.08	4.76	2.74	10^{-2}	C			
		rs1830258786:C	taaacacttg	T→C	tggtactggg	7.06	0.75	8.32	0.95	2.11	0.05	D			
		rs1830259026:A	ggataaacac	T→A	ctctggtact	7.06	0.75	8.19	0.73	2.14	0.05	D			
		rs1830261354:C	ccttagttca	T→C	aagcctgcct	8.32	0.95	14.63	1.00	8.50	10^{-6}	A			
		rs200311057:C	cttcgctctc	T→C	gagcacagcc	56.75	3.86	62.7	4.66	1.98	0.05	D			
		rs201808019:A	gccacaggaa	G→A	tcgctctctt	56.75	3.86	70.03	4.52	4.49	10^{-3}	B			
		rs922964124:G	gccttagttc	A→G	caagcctgcc	8.32	0.95	14.28	1.10	7.85	10^{-6}	A			
		rs952525996:G	tctctttgcc	A→G	agccgcttcg	56.75	3.86	78.50	6.17	6.24	10^{-6}	A			
		rs1055006926:T	tgccttagtt	C→T	tcaagcctgc	8.32	0.95	3.32	0.26	13.3	10^{-6}	A	↑	within a cohort-based biomedical study [80]: the increasing level of ABCB1 expression with an increase in the duration of drug treatment may contribute to the molecular mechanisms of the emergence and progression of multidrug resistance, which may delay POAG treatment	▼
		rs1333197116:C	aacacttgta	T→C	gtactgggat	7.06	0.75	4.90	0.54	4.80	10^{-3}	B			
		rs1333197116:G	aacacttgta	T→G	gtactgggat	7.06	0.75	4.94	0.54	4.67	10^{-3}	B			
		rs1377882204:A	tattaccatt	T→A	ataaacactt	7.06	0.75	5.16	0.44	4.63	10^{-3}	B			
		rs1584915287:A	ggctgggcag	G→A	tgaggctgat	56.75	3.86	33.96	2.58	10.08	10^{-6}	A			
		rs1584915309:A	gaggctgatt	G→A	tcatctgtgg	56.75	3.86	42.34	3.15	5.81	10^{-6}	A			
		rs1819430203:A	tggctgggca	G→A	gtgaggctga	56.75	3.86	44.81	3.10	4.87	10^{-3}	B			
		rs1830259533:T	ctgggataaa	C→T	ctcctctggt	7.06	0.75	2.86	0.42	10.03	10^{-6}	A			
		rs200558234:A	tggtgaggct	G→A	gcagtcatct	56.75	3.86	30.79	2.54	11.45	10^{-6}	A			
		rs528747108:T	gggataaaca	C→T	cctctggtac	7.06	0.75	4.70	0.54	5.22	10^{-6}	A			
		rs564911658:T	ggttttcact	G→T	acttatcctt	8.32	0.95	5.38	0.59	5.54	10^{-6}	A			
		rs894975326:A	agcctgcctg	C→A	tagtgctttc	8.32	0.95	6.29	0.69	3.53	10^{-3}	B			

Table S3. Cont.

#	Human Gene: *NCBI Gene Symbol* (NCBI Gene ID)	Candidate SNP marker: dbSNP ID:min [437]	5′ flank, 10 bp	WT →min	3 flank, 10 bp	K_D, nM, *in silico*: WT MEAN	± SEM	min MEAN	± SEM	Significance: Z	p	ϱ	Δ	Effect of changes in human gene expression on the development of primary open-angle glaucoma (POAG, ☼: "▼" aggravation, "▲" alleviation) [Reference]	☼ ▲▼
3	*ABCC4* (10257)	rs1330141361:C	aagcggctgc	T→C	ccggctcccg	43.84	3.61	83.96	6.8	11.24	10^{-6}	A	↓	within human POAG models using Dutch-Belted rabbits subjected with topical application of MK571 as the low molecular weight Abcc4 inhibitor [81]: reduced intraocular pressure alleviating POAG	▲
		rs895616841:C	cgggcctgag	A→C	ccccgaggcg	121.25	9.48	257.05	19.58	13.77	10^{-6}	A			
		rs924514487:C	gctgcttcac	A→C	tcccggaagc	43.84	3.61	88.55	7.02	12.29	10^{-6}	A			
		rs924514487:G	gctgcttcac	A→G	tcccggaagc	43.84	3.61	75.07	5.84	9.50	10^{-6}	A			
		rs1054444461:A	gcggcgggcc	T→A	gcgcccccga	121.25	9.48	102.07	8.12	3.09	10^{-2}	C	↑	within a comparative study of human pre- versus post-chemotherapy retinoblastoma cell transcriptome normalized using adjacent non-cancer cells [82]: anticancer chemotherapy increased ABCC4 expression by 33% along with an alleviation of retinoblastoma, which is comorbid to POAG according to a biomedical review [83]	
		rs113655070:T	ccaccgccgc	C→T	ggagcccgcg	71.87	5.12	60.37	4.14	3.53	10^{-3}	B			
		rs1278513141:A	ggcgcgtagc	G→A	ctccaggcgg	94.58	9.78	61.56	6.33	5.89	10^{-6}	A			
		rs1278513141:T	ggcgcgtagc	G→T	ctccaggcgg	94.58	9.78	61.64	6.46	5.82	10^{-6}	A			
		rs1406172327:T	gccggagccc	C→T	caggcgtggc	71.87	5.12	63.94	4.98	2.22	0.05	D			
		rs1428212996:T	ccccagcatc	C→T	ggcggccgga	71.87	5.12	44.69	3.47	9.02	10^{-6}	A			
		rs1441254999:C	gcggcgcgta	G→C	ggctccaggc	94.58	9.78	75.43	8.53	2.95	10^{-2}	C			
		rs1594471127:T	gcttgaggtc	C→T	ccagcatccc	71.87	5.12	32.32	3.14	13.28	10^{-6}	A			
		rs1594471361:T	caggcggcgg	C→T	ctcccgggct	94.58	9.78	54.66	5.49	7.61	10^{-6}	A			
		rs2041679445:A	cctgcttgag	G→A	gccccagcat	71.87	5.12	58.69	4.39	3.92	10^{-3}	B			
		rs2041681402:T	gcttcacagg	C→T	cggaagcggc	43.84	3.61	38.05	3.01	2.48	0.05	D			
		rs2041681699:T	ggctgcttca	C→T	ctcccggaag	43.84	3.61	13.22	1.23	19.32	10^{-6}	A			
		rs2041683404:T	ggcggcgggc	C→T	ggcgcccccg	94.58	9.78	61.52	4.24	6.92	10^{-6}	A			
		rs2041683474:A	gaggcggcgg	G→A	cgggcgcccc	121.25	9.48	90.12	6.72	5.49	10^{-6}	A			
		rs2041683554:T	ccgaggcggc	G→T	agcgggcgcc	121.25	9.48	100.75	7.80	3.37	10^{-3}	B			
		rs2041684341:T	gcgcgtagcg	G→T	tccaggcggc	94.58	9.78	71.91	7.27	3.79	10^{-3}	B			
		rs2041684572:A	gcggcggcgc	G→A	ccgggctcca	94.58	9.78	51.60	5.04	8.52	10^{-6}	A			
		rs72557965:T	gatcagcgcg	A→T	caccgccgcc	71.87	5.12	63.53	4.27	2.52	0.05	D			
		rs761238493:A	tgatcagcgc	G→A	ccaccgccgc	71.87	5.12	64.45	4.38	2.22	0.05	D			
		rs929533777:A	tgcttgaggt	C→A	cccagcatcc	71.87	5.12	17.60	1.88	21.96	10^{-6}	A			
		rs929533777:T	tgcttgaggt	C→T	cccagcatcc	71.87	5.12	59.49	3.85	3.93	10^{-3}	B			
		rs949866238:A	tgcttcacag	G→A	ccggaagcgg	43.84	3.61	35.21	2.86	3.79	10^{-3}	B			
		rs973797350:A	tccaggcggc	G→A	agctcccggg	94.58	9.78	77.6	7.63	2.77	10^{-2}	C			
		rs981312112:C	ctgcttcaca	G→C	cccggaagcg	43.84	3.61	32.68	2.64	5.10	10^{-6}	A			
4	*ACE* (1636)	rs1165773630:A	tcctcctgca	G→A	tacaacaaga	9.75	0.98	14.06	1.12	5.70	10^{-6}	A	↓	according to a comprehensive pharmacological mini-review [84]: ACE-inhibitors may be promising medications against POAG due to reducing angiotensin II levels that may have beneficial effects via lowering vascular superoxide anion production	▲
		rs1377273896:C	tgtcgggttt	T→C	ataacccgca	2.84	0.33	10.74	0.99	17.88	10^{-6}	A			
		rs2029992189:G	cctgcagtac	A→G	acaagatcct	9.75	0.98	19.13	1.85	9.66	10^{-6}	A			
		rs2029992650:G	gcagtacaac	A→G	agatcctgtt	9.75	0.98	14.03	1.35	5.22	10^{-6}	A			
		rs2049625286:T	cgggttttat	A→T	acccgcaggg	2.84	0.33	5.63	0.47	9.54	10^{-6}	A			
		rs766503941:C	tccgctctta	T→C	tggccagggg	10.8	0.99	16.94	1.80	6.41	10^{-6}	A			
		rs961011546:G	gtgtgtcggg	T→G	tttataaccc	2.84	0.33	3.77	0.50	3.19	10^{-2}	C			
		rs992062827:T	ttgtggggcg	G→T	gcaggctggc	73.11	5.16	80.91	6.00	1.98	0.05	D			
		rs1160198167:C	gggttttata	A→C	cccgcagggc	2.84	0.33	2.33	0.24	2.52	0.05	D	↑	within a biomedical pharmaceutical study [85]: captopril (SQ 14225, ACE-inhibitor) reduced intraocular pressure, with no effects on heart rate and pupil diameter as a new promising anti-glaucomatous medication	▼
		rs1391800646:A	gcggccggct	T→A	gtggggcggg	73.11	5.16	62.16	7.24	2.38	0.05	D			
		rs1418567921:T	cttcctcctg	C→T	agtacaacaa	9.75	0.98	4.06	0.49	11.10	10^{-6}	A			
		rs1430095199:A	gcacgcggcc	G→A	gcttgtgggg	73.11	5.16	52.88	3.86	6.38	10^{-6}	A			
		rs1430095199:T	gcacgcggcc	G→T	gcttgtgggg	73.11	5.16	56.31	5.01	4.59	10^{-3}	B			
		rs2049635348:C	cacgcggccg	G→C	cttgtggggc	73.11	5.16	59.59	5.20	3.64	10^{-3}	B			
		rs538952820:C	ggtgtgtcgg	G→C	ttttataacc	2.84	0.33	2.44	0.25	1.96	0.05	D			
		rs71375895:A	tgcgggctcc	G→A	ctcttattgg	10.80	0.99	9.20	0.83	2.49	0.05	D			
		rs763413849:T	gggctccgct	C→T	ttattggcca	10.80	0.99	5.90	0.55	9.24	10^{-6}	A			
		rs767906072:A	gagaccaagt	G→A	caaaggagta	9.75	0.98	5.41	0.55	8.22	10^{-6}	A			
		rs779433192:A	tgcagtacaa	C→A	aagatcctgt	9.75	0.98	5.14	0.53	8.89	10^{-6}	A			
		rs959531460:A	cggcttgtgg	G→A	gcgggcaggc	73.11	5.16	63.68	5.04	2.60	10^{-2}	C			

Table S3. Cont.

Human Gene		Candidate SNP marker				K_D, nM, *in silico*				Significance				Effect of changes in human gene expression on the development of primary open-angle glaucoma (POAG, ☼: "▼" aggravation, "▲" alleviation) [Reference]	☼
#	*NCBI Gene Symbol* (NCBI Gene ID)	dbSNP ID:min [437]	5′ flank, 10 bp	WT →min	3 flank, 10 bp	WT MEAN	± SEM	min MEAN	± SEM	Z	p	ϱ	Δ		▲ ▼
5	*ACVR1* (90)	rs1296957714:G	tcggaagttt	A→G	gcaagggtct	6.61	0.62	9.79	0.91	5.97	10^{-6}	A	↓	within a Saudi cohort-based biomedical study [86]: minor allele "G" of the biomedical SNP marker rs12997 of POAG can upregulate ACVR1 through the target-site damage for miR-330-3p that may result in ACVR1 downregulation by this miRNA as norm	▲
		rs1297621806:C	acagtaccaa	T→C	cttctaggaa	8.36	0.82	17.75	1.70	10.99	10^{-6}	A			
		rs1325936579:C	cagacactca	T→C	aatcacagct	26.87	1.86	43.48	2.77	10.23	10^{-6}	A			
		rs1462678334:G	cgcgccgcgc	C→G	ccgcgccgcc	147.09	12.28	175.04	14.36	2.97	10^{-2}	C			
		rs1480212417:C	ccggccccgc	A→C	gcccccggct	110.98	8.16	161.97	12.10	7.21	10^{-6}	A			
		rs1480212417:G	ccggccccgc	A→G	gcccccggct	110.98	8.16	173.44	12.83	8.56	10^{-6}	A			
		rs1490820984:G	gccgcgccga	C→G	cgccgccccg	147.09	12.28	225.39	18.58	7.27	10^{-6}	A			
		rs1687574938:C	gctggaatgt	T→C	catctgttac	3.06	0.30	6.73	0.72	10.96	10^{-6}	A			
		rs1688861598:G	atcggaagtt	T→G	agcaagggtc	6.61	0.62	9.79	0.91	5.97	10^{-6}	A			
		rs1688865166:G	aaacagtacc	A→G	cccttctagg	8.36	0.82	17.00	1.48	10.82	10^{-6}	A			
		rs1688865391:G	ctaggaaaac	A→G	ggacttccct	8.36	0.82	11.34	1.10	4.41	10^{-3}	B			
		rs1688865490:G	ttctaggaaa	A→G	caggacttcc	8.36	0.82	11.00	0.82	4.45	10^{-3}	B			
		rs1690242948:C	ctttgccaag	T→C	gttgcacggt	5.55	0.46	9.43	0.83	8.79	10^{-6}	A			
		rs1690272111:G	gccgacctgc	A→G	ccccgcgccg	147.09	12.28	171.62	13.76	2.66	10^{-2}	C			
		rs1690272455:C	cgcgccgacc	T→C	ccgccccgcg	147.09	12.28	257.36	21.82	9.40	10^{-6}	A			
		rs1690272687:C	cgccgcgccg	A→C	gcgccgcccc	147.09	12.28	257.36	21.82	9.40	10^{-6}	A			
		rs1690307150:C	agacactcat	T→C	atcacagctc	26.87	1.86	29.72	2.07	2.05	0.05	D			
		rs1690307354:C	agctccagac	A→C	tggttaatca	26.87	1.86	69.15	4.70	19.50	10^{-6}	A			
		rs1690307354:G	agctccagac	A→G	tggttaatca	26.87	1.86	60.83	3.94	17.24	10^{-6}	A			
		rs576786850:G	tgccaagttt	A→G	gcacggtact	5.55	0.46	10.39	0.94	10.26	10^{-6}	A			
		rs748440812:A	actttgccaa	G→A	tgttgcacgg	5.55	0.46	7.35	0.73	4.36	10^{-3}	B			
		rs928264330:G	ggaaaacagt	A→G	cttcccttct	8.36	0.82	18.79	1.65	12.31	10^{-6}	A			
		rs933769569:A	cacagctcca	G→A	gcctggttaa	26.87	1.86	32.43	2.62	3.54	10^{-3}	B			
		rs937794314:G	aacagtacca	A→G	ccttctagga	8.36	0.82	12.56	1.29	5.72	10^{-6}	A			
		rs1026671986:A	aacctcgggc	G→A	agtgaggcta	17.34	1.62	12.12	0.83	6.19	10^{-6}	A	↑	according to comprehensive review [87]: ACVR1 upregulation follows 1 hour after an increase in intraocular pressure aggravating POAG	▼
		rs1033044615:T	gccggctgca	C→T	cgggctccct	147.09	12.28	127.87	10.75	2.36	0.05	D			
		rs1036724933:A	gcgcccggct	G→A	gccgacctgc	147.09	12.28	55.6	5.25	15.44	10^{-6}	A			
		rs1248710567:T	ccgcgccgcg	C→T	cccgcgccgc	147.09	12.28	117.92	9.21	3.87	10^{-3}	B			
		rs1276478393:T	ctcattggcc	G→T	agctccagac	26.87	1.86	24.07	1.76	2.19	0.05	D			
		rs1281995406:T	cgacctgcag	C→T	ccgcgccgcg	147.09	12.28	119.46	9.93	3.53	10^{-3}	B			
		rs1284099979:T	gctcccggcc	C→T	ctcggccccc	110.98	8.16	98.22	8.58	2.14	0.05	D			
		rs1292176173:T	taccaataag	C→T	taggaaaaca	8.36	0.82	5.70	0.52	5.70	10^{-6}	A			
		rs1296702895:T	gctttcattc	C→T	gcctccccct	21.42	1.53	17.57	1.16	4.07	10^{-3}	B			
		rs1307884220:A	caagcttgat	C→A	cctttctccc	21.42	1.53	16.72	1.61	4.14	10^{-3}	B			
		rs1336887683:T	ctgcctcgca	C→T	gcagcgcccg	147.09	12.28	117.70	8.57	4.02	10^{-3}	B			
		rs1365308623:A	gccccgcaga	G→A	cccggctccc	110.98	8.16	66.19	4.63	10.19	10^{-6}	A			
		rs1397685642:A	gcccggctgc	C→A	cgacctgcag	147.09	12.28	76.98	5.24	12.02	10^{-6}	A			
		rs1417859192:A	cgggctccct	C→A	ccgcagagtt	147.09	12.28	128.42	13.08	2.06	0.05	D			
		rs1462678334:A	cgcgccgcgc	C→A	ccgcgccgcc	147.09	12.28	90.56	7.26	8.38	10^{-6}	A			
		rs1490820984:A	gccgcgccga	C→A	cgccgccccg	147.09	12.28	91.83	6.74	8.47	10^{-6}	A			
		rs1688862462:A	cccacctctt	G→A	tttttttttc	6.61	0.62	3.10	0.31	11.12	10^{-6}	A			
		rs1690239126:T	ccgctggatt	C→T	ggcctctggg	17.34	1.62	4.67	0.52	18.02	10^{-6}	A			
		rs1690271057:A	cactccgcct	C→A	cggctgcctc	147.09	12.28	130.26	11.72	1.98	0.05	D			
		rs1690271143:A	ctcgcactcc	G→A	cgcccggctg	147.09	12.28	96.06	6.39	7.98	10^{-6}	A			
		rs1690271311:T	gcctcgcact	C→T	agcgcccggc	147.09	12.28	96.05	6.73	7.82	10^{-6}	A			
		rs1690276773:T	cccggccccg	C→T	ggcccccggc	110.98	8.16	30.85	3.59	18.58	10^{-6}	A			
		rs956007103:T	tcgccggctg	C→T	tccgggctcc	110.98	8.16	44.43	5.69	12.40	10^{-6}	A			
		rs988922806:T	ggctccctcg	C→T	gcagagttcc	147.09	12.28	115.76	8.26	4.36	10^{-3}	B			

Table S3. Cont.

Human Gene		Candidate SNP marker				K_D, nM, *in silico*				Significance				Effect of changes in human gene expression on the development of primary open-angle glaucoma (POAG, ☼: "▼" aggravation, "▲" alleviation) [Reference]	☼ ▲ ▼
						WT		min							
#	*NCBI Gene Symbol* (NCBI Gene ID)	dbSNP ID:min [437]	5′ flank, 10 bp	WT →min	3 flank, 10 bp	MEAN±	SEM	MEAN±	SEM	Z	p	ϱ	Δ		
6	*ADIPOQ* (9370)	rs1026210588:A	gcccccgtga	G→A	taccaggctg	30.38	1.75	18.26	2.84	6.13	10^{-6}	A	↑	within human disease cellular models using AdipoQ-deficient mice pre-adipocyte cell culture processed with thapsigargin as a new promising phytochemical medication inducing integrated stress response [88]: threefold increase in Adipoq protein level through improved translation as a protection against oxidative stress, obesity, insulin-resistance, and diabetes that altogether can relieve metabolic syndrome and reduce risks of POAG [89]	▲
		rs1234356305:T	tgggaaatga	C→T	aattgtgagg	30.38	1.75	10.86	0.89	20.59	10^{-6}	A			
		rs1402472817:A	tgacaattgt	G→A	aggtggggac	30.38	1.75	13.48	1.23	15.07	10^{-6}	A			
		rs1452297273:C	gaaatgacaa	T→C	tgtgaggtgg	30.38	1.75	26.49	1.59	3.29	10^{-2}	C			
		rs1723033906:T	gtaccaggct	G→T	ttgaggctgg	30.38	1.75	27.58	1.76	2.24	0.05	D			
		rs201665933:T	cccgtgagta	C→T	caggctgttg	30.38	1.75	18.76	1.67	9.10	10^{-6}	A			
		rs539178974:A	gcctgccccc	G→A	tgagtaccag	30.38	1.75	17.11	1.34	11.79	10^{-6}	A			
		rs76288006:T	tgcctgcccc	C→T	gtgagtacca	30.38	1.75	19.09	1.67	8.88	10^{-6}	A			
		rs906028479:T	tgagtaccag	G→T	ctgttgaggc	30.38	1.75	23.52	2.37	4.41	10^{-3}	B			
7	*ADRB2* (154)	rs1008147293:C	agtcctgtgc	A→C	cataacgggc	13.92	1.09	20.54	2.36	5.58	10^{-6}	A	↓	within an in vitro autoradiographic study of beta-adrenergic specific binding sites on the human retinal vessels [90]: ADRB2-blockers are drugs in the POAG treatment to reduce intraocular pressure	▲
		rs1008147293:G	agtcctgtgc	A→G	cataacgggc	13.92	1.09	20.54	2.36	5.58	10^{-6}	A			
		rs1302798886:G	tgtgcacata	A→G	cgggcagaac	13.92	1.09	17.75	1.57	4.10	10^{-3}	B			
		rs1756545774:C	taaagtcctg	T→C	gcacataacg	13.92	1.09	16.28	1.25	2.85	10^{-2}	C			
		rs35913731:C	tcctgtgcac	A→C	taacgggcag	13.92	1.09	20.54	2.36	5.58	10^{-6}	A			
		rs35913731:G	tcctgtgcac	A→G	taacgggcag	13.92	1.09	20.54	2.36	5.58	10^{-6}	A			
		rs759570402:A	cctgtgcaca	T→A	aacgggcaga	13.92	1.09	20.49	1.60	6.97	10^{-6}	A			
		rs759570402:C	cctgtgcaca	T→C	aacgggcaga	13.92	1.09	20.54	2.36	5.58	10^{-6}	A			
		rs759570402:G	cctgtgcaca	T→G	aacgggcaga	13.92	1.09	20.54	2.36	5.58	10^{-6}	A			
		rs1756545520:T	gggcagttcc	C→T	ctaaagtcct	13.92	1.09	12.06	1.15	2.33	0.05	D	↑	within a biomedical cohort-based study [91]: ADRB2-excess along with its agonistic autoantibodies are biomedical molecular markers for any POAG development state	▼
		rs1756546300:T	gtgcacataa	C→T	gggcagaacg	13.92	1.09	11.84	0.86	3.03	10^{-2}	C			
		rs35913731:T	tcctgtgcac	A→T	taacgggcag	13.92	1.09	11.33	1.04	3.42	10^{-3}	B			
8	*AFAP1* (60312)	rs1200852193:G	ccaacctccc	T→G	caccgccccg	28.02	1.84	46.92	3.20	10.87	10^{-6}	A	↓	within human disease cellular models using human AFAP1-knockdown cells [92]: reduced actin stress fiber cross-linking and decreased adhesion to fibronectin, each of which may slow a progression of POAG [93]	▲
		rs1205161380:C	ctgtaagaat	A→C	ccaagtgacc	10.27	0.68	14.76	1.42	6.21	10^{-6}	A			
		rs1235322756:G	cggggccgtg	T→G	cgggcggcgg	88.43	7.65	397.16	30.45	25.98	10^{-6}	A			
		rs1250876431:T	agaataactt	A→T	tgaccactgt	10.27	0.68	12.22	0.52	4.43	10^{-3}	B			
		rs1355794636:G	ttgtcacacc	A→G	aggcctttgg	27.51	2.14	37.17	2.79	5.56	10^{-6}	A			
		rs1355794636:T	ttgtcacacc	A→T	aggcctttgg	27.51	2.14	37.17	2.79	5.56	10^{-6}	A			
		rs1371935149:G	caacctccct	T→G	accgccccgc	28.02	1.84	49.31	3.74	11.26	10^{-6}	A			
		rs1378620290:C	tgggtctaat	A→C	ttgcaaatca	3.41	0.31	4.55	0.43	4.43	10^{-3}	B			
		rs1717105734:C	ctaataatac	T→C	aatcactggg	3.41	0.31	4.25	0.34	3.65	10^{-3}	B			
		rs751324719:G	cactgggtct	A→G	catttgcaaa	3.41	0.31	4.55	0.43	4.43	10^{-3}	B			
		rs75661638:C	acaccatctg	T→C	tttgggttgt	27.51	2.14	36.44	2.94	5.01	10^{-6}	A			
		rs75661638:G	acaccatctg	T→G	tttgggttgt	27.51	2.14	33.84	2.63	3.77	10^{-3}	B			
		rs759263927:G	ataacttaat	C→G	ccactgtaag	10.27	0.68	11.25	0.60	2.15	0.05	D			
		rs765174764:G	ccactgtaag	A→G	ggtccaagtg	10.27	0.68	11.18	0.62	1.98	0.05	D			
		rs77245474:C	tcacaccatc	T→C	cctttgggtt	27.51	2.14	36.44	2.94	5.01	10^{-6}	A			
		rs867215528:G	cgggcggggc	C→G	acggcgggcg	88.43	7.65	117.75	10.48	4.61	10^{-3}	B			
		rs879288495:C	ggcggggccg	T→C	ggcgggcggc	88.43	7.65	285.12	24.10	19.35	10^{-6}	A			
		rs906509959:A	tctaataata	C→A	aaatcactgg	3.41	0.31	4.46	0.41	4.16	10^{-3}	B			
		rs907198295:G	tgggttgtca	C→G	cgtcaggcct	27.51	2.14	35.21	2.65	4.56	10^{-3}	B			

Table S3. Cont.

Human Gene		Candidate SNP marker				K_D, nM, *in silico*				Significance				Effect of changes in human gene expression on the development of primary open-angle glaucoma (POAG, ☼: "▼" aggravation, "▲" alleviation) [Reference]	☼ ▲ ▼
#	*NCBI Gene Symbol* (NCBI Gene ID)	dbSNP ID:min [437]	5′ flank, 10 bp	WT →min	3 flank, 10 bp	WT MEAN±	WT SEM	min MEAN±	min SEM	Z	p	ϱ	Δ		
		rs1014045492:T	tttctcctta	C→T	aacttaatct	10.27	0.68	4.56	0.52	12.31	10^{-6}	A			
		rs112138036:T	cctttgggtt	G→T	atgacgtcag	27.51	2.14	20.76	1.66	5.04	10^{-6}	A			
		rs1173323532:A	gcgggcgggg	C→A	gacggcgggc	88.43	7.65	43.33	4.14	11.07	10^{-6}	A			
		rs1173323532:T	gcgggcgggg	C→T	gacggcgggc	88.43	7.65	50.21	4.43	9.16	10^{-6}	A			
		rs1228181484:C	aataacttaa	T→C	accactgtaa	10.27	0.68	9.36	0.62	1.98	0.05	D			
		rs1233824980:T	tctttttctc	C→T	gaataactta	10.27	0.68	7.88	0.81	4.35	10^{-3}	B			
		rs1251586978:T	acctcccttt	G→T	cgccccgccc	28.02	1.84	23.82	1.56	3.5	10^{-3}	B			
		rs1472254139:G	actgggtcta	A→G	atttgcaaat	3.41	0.31	2.94	0.33	2.09	0.05	D			
		rs1479636830:A	ggccgtgtgc	G→A	gcggcgggcg	88.43	7.65	75.64	6.89	2.48	0.05	D			
		rs1479636830:T	ggccgtgtgc	G→T	gcggcgggcg	88.43	7.65	75.31	6.34	2.66	10^{-2}	C			
		rs1488357847:T	gggcggggcc	G→T	cggcgggcgg	88.43	7.65	49.85	3.90	9.83	10^{-6}	A			
		rs1577377422:A	gggccgtgtg	C→A	ggcggcgggc	88.43	7.65	64.09	5.38	5.34	10^{-6}	A			
		rs1717110947:T	actcagtatt	C→T	cttcgagtaa	3.41	0.31	1.26	0.13	14.36	10^{-6}	A			
8	*AFAP1* (60312)	rs1717124562:T	gttgtcacac	C→T	caggcctttg	27.51	2.14	9.28	0.85	18.11	10^{-6}	A	↑	according to an exhaustive retrospective review of POAG-related bioinformatic prioritization together with functional annotation of transcriptome-wide association studies [94]: AFAP1 upregulation might potentially protect against POAG	▲
		rs1720455390:A	accactgtaa	G→A	gggtccaagt	10.27	0.68	7.14	0.55	7.18	10^{-6}	A			
		rs752935459:A	ccctttgttc	C→A	ccgcccaacc	28.02	1.84	21.86	1.50	5.22	10^{-6}	A			
		rs752935459:T	ccctttgttc	C→T	ccgcccaacc	28.02	1.84	22.46	1.50	4.72	10^{-3}	B			
		rs75442622:A	gtcacaccat	C→A	gcctttgggt	27.51	2.14	6.48	0.65	22.84	10^{-6}	A			
		rs759263927:A	ataacttaat	C→A	ccactgtaag	10.27	0.68	6.57	0.54	8.42	10^{-6}	A			
		rs765174764:T	ccactgtaag	A→T	ggtccaagtg	10.27	0.68	3.24	0.34	18.64	10^{-6}	A			
		rs76741708:A	cacaccatct	G→A	ctttgggttg	27.51	2.14	15.21	1.40	9.83	10^{-6}	A			
		rs867215528:A	cgggcggggc	C→A	acggcgggcg	88.43	7.65	73.47	7.17	2.84	10^{-2}	C			
		rs867215528:T	cgggcggggc	C→T	acggcgggcg	88.43	7.65	46.74	4.13	10.31	10^{-6}	A			
		rs907198295:T	tgggttgtca	C→T	cgtcaggcct	27.51	2.14	11.70	1.14	13.67	10^{-6}	A			
		rs933580362:A	ggcgggcggg	G→A	ggacggcggg	88.43	7.65	66.08	5.91	4.68	10^{-3}	B			
		rs962452815:T	gcttctccca	G→T	ctggctttgg	27.51	2.14	23.25	1.57	3.26	10^{-2}	C			
		rs1005073824:T	ccctgtgaca	A→T	ttcctagcat	28.42	1.76	31.07	1.98	2.00	0.05	D			
		rs1252059021:A	gaaagatggg	G→A	acaagacgac	28.42	1.76	31.84	1.93	2.62	10^{-2}	C			
9	*AGER* (177)	rs1418451350:T	tgtgacaaga	C→T	ctagcattcc	28.42	1.76	32.39	2.03	2.97	10^{-2}	C	↓	within human disease models using Ager-knockout mice subjected with an artificial diabetes [95]: protection against many retinopathic lesions, especially those related to innate immune responses as a prevention against diabetic retinopathy, which is comorbid with POAG, at least as age-related disease [96]	▲
		rs1786829887:C	attatttctc	T→C	gtggggtgat	5.97	0.61	9.31	0.72	6.91	10^{-6}	A			
		rs755330804:C	agacgactga	A→C	tccctgtgac	28.42	1.76	31.46	2.40	2.07	0.05	D			
		rs772843309:G	ctgtgacaag	A→G	cctagcattc	28.42	1.76	38.47	2.69	6.48	10^{-6}	A			

Table S3. Cont.

#	Human Gene: *NCBI Gene Symbol* (NCBI Gene ID)	Candidate SNP marker: dbSNP ID:min [437]	5′ flank, 10 bp	WT →min	3 flank, 10 bp	K_D, nM, *in silico*: WT MEAN	± SEM	min MEAN	± SEM	Significance: Z	p	ϱ	Δ	Effect of changes in human gene expression on the development of primary open-angle glaucoma (POAG, ☼: "▼" aggravation, "▲" alleviation) [Reference]	☼ ▲▼
9	*AGER* (177)	rs1027793598:T	gggtgcaggc	C→T	ggctggagag	53.08	5.28	46.16	4.67	1.97	0.05	D	↑	within human disease models using diabetic rats treated with Zerumbone as a bioactive compound extracted from the rhizomes of Zingiber zerumbet [97]: Zerumbone has reversed back Ager-upregulation accompanied by both disarrangement and thickness reduction in retinal layers as symptoms of retinopathy, which is comorbid with POAG, at least as an age-related disease [96]	▼
		rs1225433624:G	aattatttct	C→G	ggtggggtga	5.97	0.61	4.98	0.42	2.72	10^{-2}	C			
		rs1248593697:T	gaggggcaga	C→T	cacagcaggg	53.08	5.28	28.57	2.52	9.31	10^{-6}	A			
		rs1261275476:A	ccccacctag	G→A	gagggtgcag	53.08	5.28	33.08	4.70	5.45	10^{-6}	A			
		rs1297297405:T	ggtgcaggcc	C→T	gctggagaga	53.08	5.28	33.36	3.52	6.40	10^{-6}	A			
		rs1345393602:G	tctctagtac	C→G	tgataattat	5.97	0.61	5.15	0.56	1.98	0.05	D			
		rs1426184391:A	ttccctgtga	C→A	aattcctagc	28.42	1.76	23.85	1.79	3.61	10^{-3}	B			
		rs1786811930:A	gccacagcag	G→A	ctagggcgga	53.08	5.28	44.97	3.32	2.68	10^{-2}	C			
		rs1786817204:T	agagagggtg	C→T	atgggggctg	53.08	5.28	33.9	3.28	6.46	10^{-6}	A			
		rs1786820421:A	gatgggggct	G→A	gacgactgaa	53.08	5.28	31.47	2.99	7.60	10^{-6}	A			
		rs372257952:T	gaggccacag	C→T	cacctagggc	53.08	5.28	28.14	2.33	9.80	10^{-6}	A			
		rs557568056:A	ataattattt	C→A	ggggtggggt	5.97	0.61	3.23	0.27	9.28	10^{-6}	A			
		rs748643759:A	gtgacaagac	G→A	tagcattccc	28.42	1.76	24.92	1.44	3.12	10^{-2}	C			
		rs750572313:T	gcaggcccca	C→T	ggagagaggg	53.08	5.28	30.22	3.02	7.98	10^{-6}	A			
		rs752659917:T	gggcggaggc	C→T	ggccccacct	53.08	5.28	33.63	3.40	6.44	10^{-6}	A			
		rs755330804:C	agacgactga	A→C	tccctgtgac	53.08	5.28	44.74	3.58	2.68	10^{-2}	C			
		rs756266085:A	gagagagggt	G→A	gatgggggct	53.08	5.28	35.37	3.76	5.57	10^{-6}	A			
		rs759261188:A	ccacagcagg	G→A	tagggcggag	53.08	5.28	46.94	3.17	2.05	0.05	D			
		rs760391029:T	gggagagggg	C→T	aggccacagc	53.08	5.28	18.11	1.79	15.32	10^{-6}	A			
		rs765169643:T	ggccacagca	G→T	cctagggcgg	53.08	5.28	39.46	2.87	4.81	10^{-6}	A			
		rs779376259:A	caagacgact	G→A	attccctgtg	28.42	1.76	11.13	0.77	20.23	10^{-6}	A			
		rs779376259:C	caagacgact	G→C	attccctgtg	28.42	1.76	23.85	1.54	3.92	10^{-3}	B			
10	*APBB2* (323)	rs1013141854:G	tctggtctca	A→G	ccggagcccg	15.4	1.37	24.9	2.43	7.28	10^{-6}	A	↓	within human disease models using Apbb2-knockout mice [98]: cortical cataracts, reduced lens transparency, and ocular muscle dysfunction that altogether may aggravate POAG [99]	▼
		rs1051366639:G	agcgggagca	C→G	cgggagcacc	54.39	4.14	65.41	5.16	3.36	10^{-3}	B			
		rs1186162346:C	ggagcacttc	A→C	agcacctagc	54.39	4.14	132.33	9.81	16.73	10^{-6}	A			
		rs1186162346:G	ggagcacttc	A→G	agcacctagc	54.39	4.14	91.15	6.98	9.56	10^{-6}	A			
		rs1188791744:G	cacactgtat	A→G	aaatatttac	2.24	0.28	7.48	0.81	14.52	10^{-6}	A			
		rs1188791744:T	cacactgtat	A→T	aaatatttac	2.24	0.28	6.55	0.64	13.53	10^{-6}	A			
		rs1269688048:C	cggccccgcc	T→C	cccccacccc	43.71	3.23	87.75	6.04	13.82	10^{-6}	A			
		rs1269688048:G	cggccccgcc	T→G	cccccacccc	43.71	3.23	73.42	5.56	9.81	10^{-6}	A			
		rs1408507830:G	gttctggtct	C→G	tgccggagcc	15.4	1.37	20.40	1.73	4.58	10^{-3}	B			
		rs1490274532:C	gcgggagcac	T→C	gggagcacct	54.39	4.14	107.26	7.07	13.48	10^{-6}	A			
		rs1756191385:A	cctaaaataa	G→A	gatacagttg	8.26	0.64	9.34	0.78	2.17	0.05	D			
		rs1756192494:G	cagttgacct	A→G	tctgggtgat	8.26	0.64	9.34	0.78	2.17	0.05	D			
		rs1761539013:C	ccagcagcca	A→C	cacgcgcctc	132.33	9.81	166.18	12.88	4.25	10^{-3}	B			
		rs1761540776:C	gcgcctcccc	A→C	ggcggggcca	132.33	9.81	165.69	13.51	4.08	10^{-3}	B			
		rs1761568402:C	cgggagcact	T→C	ggagcaccta	54.39	4.14	118.00	9.04	14.33	10^{-6}	A			
		rs1771672555:C	gggaaagaca	T→C	ttgtgtccca	10.26	1.06	13.31	1.75	3.10	10^{-2}	C			
		rs1772633358:T	cccggtaaaa	G→T	aggacctcgc	9.47	0.84	12.57	1.07	4.61	10^{-3}	B			
		rs1780182730:G	ggtctcaagt	A→G	gagcccgttc	15.4	1.37	46.35	3.21	19.58	10^{-6}	A			
		rs1780183232:A	tggtctcaag	T→A	ggagcccgtt	15.4	1.37	45.76	3.19	19.29	10^{-6}	A			
		rs1780183680:C	ctggtctcaa	G→C	cggagcccgt	15.4	1.37	18.53	1.65	2.94	10^{-2}	C			
		rs1780184606:C	ttctggtctc	A→C	gccggagccc	15.4	1.37	22.62	2.26	5.76	10^{-6}	A			
		rs1780205450:T	gccaaagcct	G→T	gcgcagccgc	40.26	3.07	46.26	3.29	2.66	10^{-2}	C			
		rs1809307689:G	ctcacactgt	A→G	aaaaatattt	2.24	0.28	5.31	0.32	12.39	10^{-6}	A			
		rs560266493:G	actgtatact	A→G	tatttactca	2.24	0.28	3.19	0.38	4.10	10^{-3}	B			
		rs573261813:C	tgccaaagcc	T→C	cgcgcagccg	40.26	3.07	47.88	3.56	3.25	10^{-2}	C			
		rs747702142:A	cctggagaag	T→A	cgctgccaaa	40.26	3.07	58.62	3.83	7.48	10^{-6}	A			
		rs994283474:A	aaataagcac	G→A	cagttgacct	8.26	0.64	9.25	0.78	1.99	0.05	D			

Table S3. Cont.

Human Gene		Candidate SNP marker				K$_D$, nM, *in silico*				Significance				Effect of changes in human gene expression on the development of primary open-angle glaucoma (POAG, ☼: "▼" aggravation, "▲" alleviation) [Reference]	☼ ▲ ▼
						WT		min							
#	*NCBI Gene Symbol* (NCBI Gene ID)	dbSNP ID:min [437]	5′ flank, 10 bp	WT →min	3 flank, 10 bp	MEAN	± SEM	MEAN	± SEM	Z	p	ϱ	Δ		
		rs1051366639:T	agcgggagca	C→T	cgggagcacc	54.39	4.14	39.24	2.94	6.11	10^{-6}	A			
		rs1054680462:A	tactcacact	G→A	ataaaaatat	2.24	0.28	0.89	0.11	10.67	10^{-6}	A			
		rs1182974271:T	tccccagcag	C→T	ggccacgcgc	54.39	4.14	37.98	2.86	6.71	10^{-6}	A			
		rs1186162346:T	ggagcacttc	A→T	agcacctagc	54.39	4.14	44.28	3.50	3.75	10^{-3}	B			
		rs1199814984:T	gctcagttgc	C→T	gcactgagca	82.87	5.61	67.58	4.47	4.31	10^{-3}	B			
		rs1229558901:A	gcggggccac	G→A	ggccgggtgg	132.33	9.81	96.34	8.51	5.51	10^{-6}	A			
		rs1241671736:A	tcatcataat	G→A	atctcttggc	8.26	0.64	5.55	0.68	5.52	10^{-6}	A			
		rs1305268996:A	agcacctagc	G→A	catctggcgg	54.39	4.14	48.60	3.98	2.01	0.05	D			
		rs1329240292:A	cagcgccgct	C→A	tggaatctcg	82.87	5.61	49.85	4.73	8.72	10^{-6}	A			
		rs1347599468:G	gcccccggta	A→G	aagaggacct	9.47	0.84	8.05	0.76	2.50	0.05	D			
		rs1362670462:A	cacttcacgg	G→A	cctagcggga	54.39	4.14	41.51	3.35	4.87	10^{-3}	B			
		rs1408507830:A	gttctggtct	C→A	tgccggagcc	15.40	1.37	8.62	0.71	9.60	10^{-6}	A			
		rs1408507830:T	gttctggtct	C→T	tgccggagcc	15.40	1.37	5.16	0.49	16.92	10^{-6}	A			
		rs1412248980:A	gtggaatctc	G→A	agcctggaga	82.87	5.61	58.48	3.84	7.39	10^{-6}	A			
		rs1469610604:T	ctagcgggag	C→T	ggcgggagca	54.39	4.14	17.31	1.79	17.81	10^{-6}	A			
		rs1472214937:C	agcatgctca	G→C	gaacggcact	82.87	5.61	69.10	4.85	3.72	10^{-3}	B			
		rs1580896815:A	ccccggcccc	G→A	cggcccccac	43.71	3.23	37.23	2.75	3.07	10^{-2}	C			
		rs1756193527:T	tacagttgac	C→T	cttctgggtg	8.26	0.64	6.44	0.41	4.95	10^{-6}	A			
		rs1756195802:T	tgatacagtt	G→T	acacttctgg	8.26	0.64	6.50	0.5	4.42	10^{-3}	B			
		rs1761526370:T	agggagggcg	C→T	gcgggcgggg	132.33	9.81	70.12	7.19	10.04	10^{-6}	A			
		rs1761547079:T	ggggcggggc	C→T	tgcggccggg	132.33	9.81	93.39	9.29	5.62	10^{-6}	A			
		rs1780192441:T	tgctcagttg	C→T	ggcactgagc	82.87	5.61	32.81	2.41	18.56	10^{-6}	A			
10	*APBB2* (323)	rs1780197198:A	cgcgggactc	G→A	gccgctccct	82.87	5.61	62.67	4.82	5.45	10^{-6}	A	↑	within biomedical cohort-based genome-wide APBB2 SNP association study of a population of Polish centenarians [100]: APBB2 overexpressing/beta-amyloid accumulating alleles fit in with longevity along with the very first phenotypic manifestations of cognitive impairment only in the elderly, as something like neuroprotection against POAG	▲
		rs1780202999:T	agtggaatct	C→T	aagcctggag	82.87	5.61	49.21	3.02	11.40	10^{-6}	A			
		rs1780207117:T	gcagccgctg	C→T	ccttccctcg	40.26	3.07	31.71	2.74	4.14	10^{-3}	B			
		rs1780218573:T	cccggccccg	C→T	ggcccccacc	43.71	3.23	34.92	2.61	4.27	10^{-3}	B			
		rs35178640:A	ctcgcccccg	G→A	cagaagagga	9.47	0.84	6.68	0.57	5.68	10^{-6}	A			
		rs376389662:A	cctcgccccc	G→A	gcagaagagg	9.47	0.84	6.68	0.52	5.88	10^{-6}	A			
		rs537547371:T	agtgcggccg	G→T	cacttcacgg	132.33	9.81	98.66	8.10	5.31	10^{-6}	A			
		rs571084310:A	gcagccaatg	G→A	gcgcctcccc	132.33	9.81	80.93	5.93	9.43	10^{-6}	A			
		rs577356623:T	cggaacggca	C→T	cgcgggactc	82.87	5.61	60.94	4.05	6.48	10^{-6}	A			
		rs748261156:A	gtcaacaact	G→A	gtagtgttgc	9.47	0.84	6.34	0.73	5.52	10^{-6}	A			
		rs764392277:A	acatgtacct	G→A	ccaggggaaa	10.26	1.06	7.86	0.93	3.41	10^{-3}	B			
		rs768273451:T	tttactcaca	C→T	atataaaaat	2.24	0.28	1.78	0.22	2.64	10^{-2}	C			
		rs779184199:T	tttcctactg	C→T	catcaggcaa	9.47	0.84	3.59	0.37	14.31	10^{-6}	A			
		rs921141759:A	gagcacttca	C→A	gcacctagcg	54.39	4.14	37.58	2.85	6.88	10^{-6}	A			
		rs921141759:T	gagcacttca	C→T	gcacctagcg	54.39	4.14	24.21	1.77	15.35	10^{-6}	A			
		rs929353208:A	ctccagcctt	C→A	ccgccgggtc	43.71	3.23	29.7	2.57	6.79	10^{-6}	A			
		rs932618325:A	gggagcactt	C→A	gagcacctag	54.39	4.14	12.93	1.28	23.01	10^{-6}	A			
		rs932618325:T	gggagcactt	C→T	gagcacctag	54.39	4.14	11.98	1.20	24.07	10^{-6}	A			
		rs954026778:A	tcgcggaacg	G→A	ctgcgcggga	82.87	5.61	50.05	3.62	10.17	10^{-6}	A			
		rs966765615:A	gcggggcagg	G→A	ggccgcggcg	132.33	9.81	111.9	8.37	3.19	10^{-2}	C			
		rs977604859:C (transcript APBB2-216)	ggagaagtgg	A→C	tgccaaagcc	82.87	5.61	74.72	5.53	2.07	0.05	D			
		rs977604859:T (transcript APBB2-214)	ggagaagtgg	A→T	tgccaaagcc	40.26	3.07	20.31	2.01	10.94	10^{-6}	A			
		rs979190450:T	gcacttcacg	G→T	acctagcggg	54.39	4.14	33.31	2.81	8.63	10^{-6}	A			

Table S3. Cont.

#	Human Gene: *NCBI Gene Symbol* (NCBI Gene ID)	Candidate SNP marker: dbSNP ID:min [437]	5′ flank, 10 bp	WT →min	3 flank, 10 bp	K$_D$, nM, *in silico*: WT MEAN	WT ± SEM	min MEAN	min ± SEM	Significance: Z	p	ϱ	Δ	Effect of changes in human gene expression on the development of primary open-angle glaucoma (POAG, ☼: "▼" aggravation, "▲" alleviation) [Reference]	☼ ▲ ▼
11	*APEX1* (328)	rs1036767971:T	gacaagcgcg	C→T	ctctgatcac	26.41	1.81	29.57	1.92	2.39	0.05	D	↓	within human disease models using mice subjected with an artificially induced retinal angiogenesis, and, next, treated with APX3330 as a small molecule inhibitor of Apex1 redox activity [101]: blocked retinal neovascularization, which can provoke severe peripheral retinal hemorrhages aggravating POAG [102]	▲
		rs1195571375:C	cccttctttg	T→C	gctcgggtta	19.45	1.44	26.41	1.81	6.06	10^{-6}	A			
		rs1195571375:G	cccttctttg	T→G	gctcgggtta	19.45	1.44	26.41	1.81	6.06	10^{-6}	A			
		rs1228269913:T	cctctgatca	C→T	gtgaccaggt	26.41	1.81	29.91	1.77	2.74	10^{-2}	C			
		rs1267686857:C	acccttcttt	G→C	tgctcgggtt	19.45	1.44	26.41	1.81	6.06	10^{-6}	A			
		rs1434543521:C	tgcacccttc	T→C	ttgtgctcgg	19.45	1.44	26.41	1.81	6.06	10^{-6}	A			
		rs1881248354:C	ctccgtcacg	T→C	ggtgtcagac	37.23	3.24	44.75	3.23	3.25	10^{-2}	C			
		rs1881248695:A	tcacgtggtg	T→A	cagacagacc	37.23	3.24	44.58	3.39	3.12	10^{-2}	C			
		rs1881248695:C	tcacgtggtg	T→C	cagacagacc	37.23	3.24	44.58	3.61	3.03	10^{-2}	C			
		rs1881252293:C	caagcgcgcc	T→C	ctgatcacgt	26.41	1.81	37.23	3.24	6.19	10^{-6}	A			
		rs1020366414:A	gtgcaccctt	C→A	tttgtgctcg	26.41	1.81	17.32	1.71	7.00	10^{-6}	A	↑	within a cohort-based biomedical study using liquid chromatography-mass spectrometry [103]: APEX1 upregulation in tear fluid of patients with primary Sjogren's syndrome is a biomedical molecular marker for ocular dryness, which may result in dry eye disease, which is comorbid to POAG at least as a side effect of antihypertensive medications in POAG [104]	▼
		rs1020366414:T	gtgcaccctt	C→T	tttgtgctcg	19.45	1.44	12.84	0.85	8.34	10^{-6}	A			
		rs1045457325:T	gtccgctacc	C→T	acgtgggggc	26.41	1.81	12.62	1.68	9.88	10^{-6}	A			
		rs104894986:A	gtgtcagaca	G→A	accaatcacg	37.23	3.24	29.23	2.23	4.18	10^{-3}	B			
		rs11622131:A	cttcggccac	G→A	acaagcgcgc	37.23	3.24	22.51	1.69	8.76	10^{-6}	A			
		rs11622131:T	cttcggccac	G→T	acaagcgcgc	37.23	3.24	15.04	1.58	13.29	10^{-6}	A			
		rs1184609076:A	aaagaaaaat	G→A	acaaagaggc	13.46	1.29	10.46	0.62	4.48	10^{-3}	B			
		rs1300114948:A	ccacgacaag	C→A	gcgcctctga	37.23	3.24	23.41	1.79	8.00	10^{-6}	A			
		rs1300114948:T	ccacgacaag	C→T	gcgcctctga	37.23	3.24	20.32	1.59	10.35	10^{-6}	A			
		rs1337142602:A	cctgtatgag	G→A	acccccaga	13.46	1.29	9.38	0.89	5.36	10^{-6}	A			
		rs1361507648:A	gccaagaaga	G→A	taagacggcc	13.46	1.29	9.76	0.83	5.02	10^{-6}	A			
		rs1881248211:A	tctccgtcac	G→A	tggtgtcaga	37.23	3.24	25.30	1.90	6.71	10^{-6}	A			
		rs1881248565:A	gtcacgtggt	G→A	tcagacagac	37.23	3.24	11.42	1.17	17.58	10^{-6}	A			
		rs1881250021:T	caatcacgcg	C→T	attcttcggc	37.23	3.24	26.44	2.15	5.75	10^{-6}	A			
		rs1881250752:T	tcttcggcca	C→T	gacaagcgcg	37.23	3.24	31.07	2.37	3.12	10^{-2}	C			
		rs1881257123:T	cttctttgtg	C→T	tcgggttagg	19.45	1.44	16.09	1.13	3.72	10^{-3}	B			
		rs1881314123:A	gcccagccct	G→A	tatgaggacc	13.46	1.29	3.76	0.45	16.62	10^{-6}	A			
		rs1881315093:A	ccctgtatga	G→A	gacccccag	13.46	1.29	7.66	0.77	8.11	10^{-6}	A			
		rs375359278:G	cacgtggtgt	C→G	agacagacca	37.23	3.24	29.66	2.38	3.84	10^{-6}	A			
		rs749166265:A	agccctgtat	G→A	aggaccccc	13.46	1.29	3.62	0.39	18.17	10^{-6}	A			
		rs754170368:A	agaagagtaa	G→A	acggccgcaa	13.46	1.29	8.37	0.79	7.08	10^{-6}	A			
		rs977106649:A	ctacccacgt	G→A	ggggctcagc	26.41	1.81	22.84	2.57	2.21	0.05	D			
12	*APOE* (348)	rs1391781909:C	gggagcccta	T→C	aattggacaa	4.86	0.52	13.32	1.21	14.42	10^{-6}	A	↓	within human disease models using ApoE-deficient mice [105]: neuroprotection against axonal damage-induced retinal ganglion cell death	▲
		rs1406267920:C	ccctataatt	G→C	gacaagtctg	4.86	0.52	6.29	0.70	3.34	10^{-3}	B			
		rs758379972:G	ggggagccct	A→G	taattggaca	4.86	0.52	15.81	1.49	16.60	10^{-6}	A			
		rs1199509478:A	cttagaatgt	G→A	aagggagaat	39.69	3.29	10.28	0.98	21.36	10^{-6}	A	↑	in cohort-based biomedical study using quantitative real-time polymerase chain reaction (qPCR) and enzyme immunosorbent assays (ELISA test) [106]: excess of both APOE mRNA and APOE protein are biomedical molecular markers for POAG	▼
		rs1373159353:A	aagggagaat	G→A	aggaatgcga	39.69	3.29	16.54	1.29	15.39	10^{-6}	A			
		rs1406267920:A	ccctataatt	G→A	gacaagtctg	4.86	0.52	3.43	0.36	4.69	10^{-3}	B			
		rs1568615131:T	atgaggaatg	C→T	gagactggga	39.69	3.29	32.28	2.46	3.67	10^{-3}	B			
13	*AQP1* (358)	rs1012082384:C	gaggaagaag	A→C	gagaaggaga	49.13	3.29	67.54	4.37	6.84	10^{-6}	A	↓	within human POAG models using mice lacking Aqp1 [107]: reduced both aqueous fluid production and intraocular pressure that might alleviate POAG	▲
		rs1015997187:G	ggccctataa	A→G	taggcccagc	1.58	0.19	1.92	0.23	2.30	0.05	D			
		rs1314294921:G	gcggtccagg	A→G	caacgtgaag	28.06	1.71	41.40	2.78	8.56	10^{-6}	A			
		rs1340576534:A	cggtccagga	C→A	aacgtgaagg	28.06	1.71	37.63	2.59	6.38	10^{-6}	A			
		rs1791169581:C	cccccggccc	T→C	ataaataggc	1.58	0.19	4.03	0.41	11.87	10^{-6}	A			
		rs28362735:G	ttcacctatg	A→G	ctctctgcct	13.31	1.41	18.66	1.52	5.06	10^{-6}	A			
		rs377506522:G	acggcggtcc	A→G	ggacaacgtg	28.06	1.71	33.09	2.27	3.59	10^{-3}	B			
		rs770124156:G	gtccaggaca	A→G	cgtgaaggtg	28.06	1.71	45.54	3.17	10.46	10^{-6}	A			
		rs772232668:C	tctcttcacc	T→C	atgactctct	13.31	1.41	29.89	2.07	12.82	10^{-6}	A			
		rs778081147:G	ctcttcacct	A→G	tgactctctg	13.31	1.41	29.89	2.07	12.82	10^{-6}	A			

Table S3. Cont.

Human Gene		Candidate SNP marker				K$_D$, nM, *in silico*				Significance				Effect of changes in human gene expression on the development of primary open-angle glaucoma (POAG, ☼: "▼" aggravation, "▲" alleviation) [Reference]	☼
						WT		min							
#	*NCBI Gene Symbol* (NCBI Gene ID)	dbSNP ID:min [437]	5′ flank, 10 bp	WT → min	3 flank, 10 bp	MEAN	± SEM	MEAN	± SEM	Z	p	ϱ	Δ		▲▼
13	*AQP1* (358)	rs1031109348:A	cagaagcaat	G→A	ggagacaggc	67.02	4.78	27.02	2.20	16.76	10^{-6}	A	↑	according to a comprehensive biomedical review [108]: within human post-injury trabecular meshwork regeneration cellular models the using adipose-derived stem cells, AQP1 excess along with improved wound healing in that might slow down optic nerve head damage as the alleviation of POAG	▲
		rs1162794255:T	acaggccttg	G→T	accaaggaaa	67.02	4.78	14.33	1.69	22.42	10^{-6}	A			
		rs1293843400:A	gagagacagg	G→A	agggcagggc	67.02	4.78	55.69	4.23	3.55	10^{-3}	B			
		rs1462565715:G	acctatgact	C→G	tctgccttcg	13.31	1.41	11.03	1.07	2.62	10^{-2}	C			
		rs1562579833:T	tctggccagg	C→T	agagggaggg	49.13	3.29	36.37	3.22	5.42	10^{-6}	A			
		rs1562579886:T	aagagacggg	G→T	tggagaagga	67.02	4.78	56.07	3.74	3.65	10^{-3}	B			
		rs1791169647:T	cggccctata	A→T	ataggcccag	1.58	0.19	0.90	0.15	5.29	10^{-6}	A			
		rs1791494279:T	caggcagagg	G→T	agggtgaggc	49.13	3.29	33.75	3.23	6.43	10^{-6}	A			
		rs1791495935:A	aggaagaaga	G→A	agaaggagag	49.13	3.29	34.78	2.24	7.43	10^{-6}	A			
		rs1791496127:T	agagaaggag	A→T	gacagggagg	67.02	4.78	58.7	4.09	2.65	10^{-2}	B			
		rs1791497128:A	gacaggcctt	G→A	gaccaaggaa	67.02	4.78	26.32	2.37	16.25	10^{-6}	A			
		rs1791497253:A	cttggaccaa	G→A	gaaagagacg	67.02	4.78	38.73	2.59	11.21	10^{-6}	A			
		rs1791546017:A	actctctctt	C→A	acctatgact	13.31	1.41	7.59	0.58	8.61	10^{-6}	A			
		rs200496523:A	ccaggacaac	G→A	tgaaggtgtc	28.06	1.71	16.95	1.09	11.34	10^{-6}	A			
		rs28362735:T	ttcacctatg	A→T	ctctctgcct	13.31	1.41	11.06	0.96	2.70	10^{-3}	B			
		rs561208577:A	gagacggggt	G→A	gagaaggaga	67.02	4.78	23.35	2.24	17.65	10^{-6}	A			
		rs563058618:A	tggggaggaa	G→A	aagagagaag	49.13	3.29	39.34	2.61	4.71	10^{-3}	B			
		rs563058618:T	tggggaggaa	G→T	aagagagaag	49.13	3.29	10.70	0.90	28.36	10^{-6}	A			
		rs563326561:T	caatgggaga	C→T	aggccttgga	67.02	4.78	28.64	2.56	14.86	10^{-6}	A			
		rs763470248:A	cctcacagat	T→A	gactacactg	13.31	1.41	9.97	0.92	4.12	10^{-3}	B			
		rs764683412:T	aggctgaggc	C→T	agcagtgggg	49.13	3.29	35.10	3.39	5.73	10^{-6}	A			
		rs779776693:T	tccaggacaa	C→T	gtgaaggtgt	28.06	1.71	19.81	1.43	7.36	10^{-6}	A			
		rs970653755:A	agggccagaa	G→A	caatgggaga	67.02	4.78	38.51	3.32	9.90	10^{-6}	A			
14	*ARHGEF12* (23365)	rs1173346124:A	cctcctccgc	T→A	gtctgcgcgc	77.68	6.28	127.73	9.57	9.02	10^{-6}	A	↓	within a retrospective literature meta-analysis [109]: ARHGEF12 deficit might be a candidate molecular marker for reduced organism viability	▼
		rs1173346124:C	cctcctccgc	T→C	gtctgcgcgc	77.68	6.28	153.72	12.75	11.79	10^{-6}	A			
		rs1292907888:T	ccgccggcct	C→T	ctccgctgtc	77.68	6.28	87.03	6.91	2.01	0.05	D			
		rs1943995573:G	tgatgatggt	A→G	gatgattggg	14.17	1.28	24.48	2.25	8.48	10^{-6}	A			
		rs998090054:C	ctccgctgtc	T→C	gcgcgcgcgc	77.68	6.28	167.89	11.70	14.44	10^{-6}	A			
		rs1019476090:A	gccgacaccc	G→A	tccgtgagct	99.70	7.72	80.91	5.95	3.91	10^{-3}	B	↑	according to a retrospective literature meta-analysis [109]: improved eye development	▲
		rs1029543993:A	tccgctgtct	G→A	cgcgcgcgcc	77.68	6.28	20.44	2.22	19.71	10^{-6}	A			
		rs1039367036:T	cggcctcctc	C→T	gctgtctgcg	77.68	6.28	64.06	4.72	3.53	10^{-3}	B			
		rs1052142680:A	ctttccatta	G→A	cagcgcagtg	14.17	1.28	9.83	1.01	5.35	10^{-6}	A			
		rs1216004864:T	ggcctcctcc	G→T	ctgtctgcgc	77.68	6.28	57.08	4.03	5.75	10^{-6}	A			
		rs1238263034:A	acacccgtcc	G→A	tgagctgatc	99.70	7.72	44.71	3.67	14.21	10^{-6}	A			
		rs1238263034:T	acacccgtcc	G→T	tgagctgatc	99.70	7.72	70.32	5.06	6.61	10^{-6}	A			
		rs1402559254:C	gggagttcga	G→C	gccccgagac	99.70	7.72	84.15	7.58	2.86	10^{-2}	C			
		rs1453144630:G	cctccgctgt	C→G	tgcgcgcgcg	77.68	6.28	47.19	4.24	8.25	10^{-6}	A			
		rs1467879297:T	cgacacccgt	C→T	cgtgagctga	99.70	7.72	48.62	3.86	12.95	10^{-6}	A			
		rs1942340264:T	ccccgcggttg	C→T	gcggccccca	77.68	6.28	63.96	5.48	3.30	10^{-3}	B			
		rs73580205:G	gagttcgagg	C→G	cccgagactc	99.70	7.72	83.82	5.73	3.36	10^{-3}	B			
		rs896859361:A	ggagttcgag	G→A	ccccgagact	99.70	7.72	70.50	5.46	6.33	10^{-6}	A			
		rs899492910:T	gcctcctccg	C→T	tgtctgcgcg	77.68	6.28	44.38	3.44	10.00	10^{-6}	A			
		rs915141318:C	cgaatgatga	T→C	ggtagatgat	14.17	1.28	11.91	1.14	2.64	10^{-2}	C			
		rs965355453:A	ccgtccgtga	G→A	ctgatcccgc	99.70	7.72	54.13	3.84	11.64	10^{-6}	A			

Table S3. Cont.

#	Human Gene: *NCBI Gene Symbol* (NCBI Gene ID)	Candidate SNP marker: dbSNP ID:min [437]	5′ flank, 10 bp	WT →min	3 flank, 10 bp	K_D, nM, *in silico*: WT MEAN	± SEM	min MEAN	± SEM	Significance: Z	p	ϱ	Δ	Effect of changes in human gene expression on the development of primary open-angle glaucoma (POAG, ☼: "▼" aggravation, "▲" alleviation) [Reference]	☼ ▲ ▼
15	*ASB10* (136371)	rs116644649:T	agggctattt	A→T	ctgggccagt	3.45	0.34	15.44	1.07	25.02	10^{-6}	A			
		rs1225221113:C	cacagtcatg	T→C	tggcccaaga	19.07	1.35	27.55	3.38	5.19	10^{-6}	A			
		rs1261141771:G	gaacacagtc	A→G	ccttggccca	19.07	1.35	27.55	3.38	5.19	10^{-6}	A			
		rs1400603986:C	gggctattta	T→C	tgggccagtc	3.45	0.34	6.59	0.68	9.14	10^{-6}	A			
		rs1584820920:A	gccagtcagg	G→A	ctgacagctg	3.45	0.34	4.12	0.34	2.79	10^{-6}	A			
		rs1801619607:T	gtcatgtgga	G→T	ccaagaacac	19.07	1.35	27.16	1.84	7.23	10^{-6}	A	↓	according to comprehensive review [110]: ASB10 mutational loss-of-function may impair an adequate response to inflammatory signals in the eye that could lead to glaucomatous vision loss	▼
		rs532487798:C	aacacagtca	T→C	cttggcccaa	19.07	1.35	27.55	3.38	5.19	10^{-6}	A			
		rs896059451:A	caagaacaca	G→A	acaccttggc	19.07	1.35	27.55	3.38	5.19	10^{-6}	A			
		rs952133738:A	ccagtcaggg	C→A	tgacagctgg	3.45	0.34	3.98	0.4	2.07	10^{-6}	A			
		rs952133738:T	ccagtcaggg	C→T	tgacagctgg	3.45	0.34	3.93	0.35	1.99	10^{-6}	A			
		rs977066049:T	tcatgtggag	C→T	caagaacaca	19.07	1.35	25.96	1.69	6.42	10^{-6}	A			
		rs1007719469:T	gccaccagag	G→T	tcgctggagc	78.32	5.73	68.78	4.86	2.55	10^{-6}	A			
		rs1157789686:A	gggcatctgg	G→A	agaggggtga	78.32	5.73	68.92	5.16	2.44	10^{-6}	A			
		rs1246375583:A	acacagtcat	G→A	ttggcccaag	19.07	1.35	9.62	0.68	13.74	10^{-6}	A			
		rs1260332398:T	gggggctggg	C→T	gctgagaacc	78.32	5.73	47.81	3.77	9.18	10^{-6}	A			
		rs1267297728:T	tgagaaccag	G→T	ctgggggttg	78.32	5.73	58.15	4.68	5.48	10^{-6}	A			
		rs1321826450:T	gctatttatg	G→T	ggccagtcag	3.45	0.34	2.75	0.29	3.14	10^{-6}	A			
		rs1398418077:A	ggcatctggg	G→A	gaggggtgag	78.32	5.73	66.49	4.76	3.2	10^{-6}	A			
		rs1426232242:T	ccagaggggt	G→T	tggagcagcc	78.32	5.73	63.07	5.21	3.93	10^{-6}	A			
		rs1459275284:A	ggctatttat	G→A	gggccagtca	3.45	0.34	1.35	0.13	13.44	10^{-6}	A			
		rs1801619873:T	cccaagaaca	C→T	ctacaccttg	19.07	1.35	11.63	0.78	10.19	10^{-6}	A	↑	within cellular POAG models using human and mice cells [111]: improved autophagy, which may alleviate POAG associated with increased intraocular pressure [112]	▲
		rs1801620732:T	aaagcttgcc	C→T	tccccgacct	19.07	1.35	9.64	0.91	11.57	10^{-6}	A			
		rs1801622686:A	tgctgagaac	C→A	catctggggg	78.32	5.73	41.44	2.79	12.81	10^{-6}	A			
		rs1801622822:A	tgggggttgc	T→A	tgaggggcat	78.32	5.73	53.43	4.12	7.2	10^{-6}	A			
		rs1801623006:A	ggggcatctg	G→A	cagaggggtg	78.32	5.73	62.14	4.37	4.56	10^{-6}	A			
		rs527249641:A	agccaccaga	G→A	ctcgctggag	78.32	5.73	50.4	3.76	8.44	10^{-6}	A			
		rs547090299:A	gagcctcgct	G→A	gccgggccgc	78.32	5.73	52.97	5.27	6.33	10^{-6}	A			
		rs889250439:A	aggggcatct	G→A	ccagaggggt	78.32	5.73	31.63	2.87	15.56	10^{-6}	A			
		rs957245156:A	atagcctaca	C→A	aagcttgccc	19.07	1.35	10.26	1.01	10.23	10^{-6}	A			
16	*ATOH7* (220202)	rs1415730244:G	tctttgctat	A→G	cctccttcag	2.46	0.26	9.3	0.85	19.03	10^{-6}	A	↓	within a biomedical cohort-based study of gene copy number variation using microarrays of blood samples [30]: only ATOH7 deletion was significantly frequent in POAG	▼

Table S3. Cont.

#	Human Gene: NCBI Gene Symbol (NCBI Gene ID)	Candidate SNP marker: dbSNP ID:min [437]	5′ flank, 10 bp	WT →min	3 flank, 10 bp	K_D, nM, *in silico*: WT MEAN	WT ± SEM	min MEAN	min ± SEM	Significance: Z	p	ϱ	Δ	Effect of changes in human gene expression on the development of primary open-angle glaucoma (POAG, ☼: "▼" aggravation, "▲" alleviation) [Reference]	☼ ▲ ▼
17	*ATXN2* (6311)	rs1158857350:T	ccaaatatgc	C→T	tttctgcagt	8.22	0.70	9.83	0.78	3.07	10^{-2}	C	↓	within human disease models using Atxn2-knockout mice [114]: dyslipidemia and insulin resistance as well as progressive obesity, which are positively correlating with POAG [115]	▼
		rs1206647618:T	aacacgcagt	C→T	cacctgcctc	24.87	2.71	31.05	2.14	3.44	10^{-3}	B			
		rs1298891805:C	agcagcaact	G→C	ttgaaaatag	11.59	1.13	16.01	1.46	4.83	10^{-3}	B			
		rs1323871955:C	ggcgtctcct	T→C	cgccgcgttc	121.58	8.32	134.49	10.32	1.96	0.05	D			
		rs1365150175:G	agcagcagca	A→G	tcattgaaaa	11.59	1.13	14.61	1.63	3.12	10^{-2}	C			
		rs1381816563:T	gcgggcctcc	C→T	ccttggtctc	42.17	3.16	47.24	3.45	2.17	0.05	D			
		rs140262591:G	tttctgcagt	A→G	gtctgctttt	8.22	0.70	12.72	1.12	7.11	10^{-6}	A			
		rs1455830722:C	gtaccaaata	T→C	tttttctgc	8.22	0.70	10.03	0.82	3.39	10^{-3}	B			
		rs1875071434:G	gccacagaat	A→G	aacacgcagt	24.87	2.71	36.66	2.89	5.76	10^{-6}	A			
		rs1876354615:G	agtaccaaat	A→G	tttttttctg	8.22	0.70	10.01	0.81	3.34	10^{-3}	B			
		rs1877803515:C	gcagcaactg	T→C	tgaaaatagc	11.59	1.13	17.11	0.97	6.89	10^{-6}	A			
		rs1882167743:G	attttgttca	A→G	taatggagag	12.19	0.79	13.72	1.14	2.24	0.05	D			
		rs1885148088:G	cggccccggg	A→G	cggcggtggc	26.73	2.64	39.27	3.99	5.43	10^{-6}	A			
		rs749796720:A	tctatttgca	G→A	gtctgcttgg	6.85	0.59	8.68	0.71	4.00	10^{-3}	B			
		rs749796720:T	tctatttgca	G→T	gtctgcttgg	6.85	0.59	8.68	0.71	4.00	10^{-3}	B			
		rs755551719:A	cgggcctccc	C→A	cttggtctcg	62.03	4.38	68.64	4.75	2.05	0.05	D			
		rs765011609:C	tattttgttc	A→C	ataatggaga	12.19	0.79	13.72	1.14	2.24	0.05	D			
		rs767312980:G	gcagtaccaa	A→G	ctttttttc	8.22	0.70	10.03	0.82	3.39	10^{-3}	B			
		rs776360703:A	acacgcagtc	G→A	acctgcctcc	24.87	2.71	31.05	2.20	3.41	10^{-3}	B			
		rs930223966:C	tttgttcaaa	T→C	atggagagta	12.19	0.79	13.72	1.14	2.24	0.05	D			
		rs938322131:C	ccgccccttc	G→C	ggcgggcctc	42.17	3.16	78.31	5.32	12.25	10^{-6}	A			
		rs1014384481:A	aagggcagac	C→A	cgccctcccc	97.72	5.91	50.55	3.48	14.39	10^{-6}	A	↑	within human neurodegenerative disease models using Atxn2-overexpressing mice [116]: neurodegeneration, which can aggravate POAG [117]	
		rs1014384481:G	aagggcagac	C→G	cgccctcccc	97.72	5.91	78.21	4.99	5.07	10^{-6}	A			
		rs1023933190:T	gcagacccgc	C→T	ctccccgaag	97.72	5.91	53.47	3.53	13.48	10^{-6}	A			
		rs1026711400:A	gacccgcctt	G→A	cccgaagggc	97.72	5.91	25.89	2.37	24.19	10^{-6}	A			
		rs1198823870:A	atccgggtag	G→A	gaggaaggcg	97.72	5.91	41.50	3.93	15.25	10^{-6}	A			
		rs1199255755:T	cttctactac	C→T	gtagccaaag	6.87	0.62	5.91	0.71	2.01	0.05	D			
		rs1202618139:T	ggaccgtatc	C→T	gcgcggcccc	26.73	2.64	17.76	1.62	6.08	10^{-6}	A			
		rs1203661416:T	gcttcatttt	C→T	ttgaaaaaca	7.57	0.77	5.24	0.44	5.56	10^{-6}	A			
		rs1222354979:A	cccgccttga	G→A	cgaagggcag	97.72	5.91	40.53	2.73	19.46	10^{-6}	A			
		rs1222354979:T	cccgccttga	G→T	cgaagggcag	97.72	5.91	41.01	2.85	18.83	10^{-6}	A			
		rs1267268861:A	ccttgaggaa	G→A	gggcagaccc	97.72	5.91	84.9	6.09	3.00	10^{-2}	C			
		rs1308996073:A	acccgcgcct	C→A	cggccgcgcc	121.58	8.32	106.71	10.06	2.24	0.05	D			
		rs1318202779:A	gccccgggac	C→A	gcggtggcgc	26.73	2.64	21.50	2.49	2.86	10^{-2}	C			
		rs1355615874:A	cgtctccttg	G→A	ccgcgttccg	121.58	8.32	74.23	5.42	9.87	10^{-6}	A			
		rs1371744210:T	ggccccggga	C→T	ggcggtggcg	26.73	2.64	21.83	2.11	2.93	10^{-2}	C			
		rs1371815160:T	cgcgttccgg	C→T	cgcgtccccg	121.58	8.32	95.26	7.86	4.55	10^{-3}	B			

Table S3. Cont.

#	Human Gene	Candidate SNP marker				K_D, nM, *in silico*				Significance				Effect of changes in human gene expression on the development of primary open-angle glaucoma (POAG, ☼: "▼" aggravation, "▲" alleviation) [Reference]	☼ ▲ ▼
	NCBI Gene Symbol (NCBI Gene ID)	dbSNP ID:min [437]	5′ flank, 10 bp	WT →min	3 flank, 10 bp	WT		min		Z	p	ϱ	Δ		
						MEAN±	SEM	MEAN±	SEM						
		rs1372850074:A	ctgtaccagt	G→A	agcagcagca	11.59	1.13	9.95	0.99	2.19	0.05	D			
		rs1379912207:A	ctccggccgc	G→A	cccgggcgcc	121.58	8.32	108.45	8.02	2.27	0.05	D			
		rs1380962636:A	gcggatccgg	G→A	ccttgaggaa	97.72	5.91	48.96	4.77	12.06	10^{-6}	A			
		rs1381286001:T	ctccttggcg	C→T	cgttccggcg	121.58	8.32	105.38	7.21	2.96	10^{-2}	C			
		rs1421533492:T	agcaactgta	C→T	aaaatagcag	11.59	1.13	6.20	0.59	9.19	10^{-6}	A			
		rs1457844733:A	gaccgtatcc	C→A	cgcggccccg	26.73	2.64	19.75	1.89	4.41	10^{-3}	B			
		rs1465955829:T	gaccgacacg	C→T	ccagaggctg	97.72	5.91	50.17	4.09	13.14	10^{-6}	A			
		rs1472170516:T	cagtcgccac	A→T	cctccaacac	24.87	2.71	11.99	1.07	10.34	10^{-6}	A			
		rs1489670698:T	cttgaggaag	G→T	ggcagacccg	97.72	5.91	71.88	5.73	6.14	10^{-6}	A			
		rs1875080509:T	cagccattta	C→T	acagcagcag	24.87	2.71	15.56	1.17	7.09	10^{-6}	A			
		rs1876319138:A	cagtgttaat	G→A	ggctctattt	6.85	0.59	5.39	0.60	3.39	10^{-3}	B			
		rs1876319791:A	ggctctattt	G→A	aaagtctgct	6.85	0.59	3.20	0.32	11.57	10^{-6}	A			
		rs1882168185:A	aagaaataat	G→A	gccgaaacgt	12.19	0.79	7.13	0.62	9.90	10^{-6}	A			
		rs1885131706:T	ctccccgccc	C→T	tctcggcggg	42.17	3.16	31.09	2.29	5.81	10^{-6}	A			
		rs1885147780:A	ccccgggacc	G→A	cggtggcgcg	26.73	2.64	15.20	1.40	8.35	10^{-6}	A			
		rs1885181667:T	aaggcggatc	C→T	ccgccttgag	97.72	5.91	76.59	7.61	4.19	10^{-3}	B			
		rs1885183887:A	agaccgacac	G→A	cccagaggct	97.72	5.91	70.20	5.76	6.49	10^{-6}	A			
		rs1885184133:T	ctgagaccga	C→T	ggccccagag	97.72	5.91	43.80	4.14	14.30	10^{-6}	A			
17	*ATXN2* (6311)	rs200655762:T	tttttttctg	C→T	ggcagtctgc	8.22	0.70	3.84	0.32	12.76	10^{-6}	A	↑	within human neurodegenerative disease models using Atxn2-overexpressing mice [116]: neurodegeneration, which can aggravate POAG [117]	▼
		rs367736419:C	ttcagacttt	G→C	ttgttcaaat	12.19	0.79	10.45	0.86	2.94	10^{-2}	C			
		rs695871:T	cttcgtcgtc	C→T	cctccccgcc	62.03	4.38	49.31	3.39	4.66	10^{-3}	B			
		rs695871:G	cttcgtcgtc	C→G	cctccccgcc	62.03	4.38	38.80	2.69	9.48	10^{-6}	A			
		rs750577637:A	ccgccgcgtt	C→A	cccgcgcgtc	121.58	8.32	32.10	3.32	21.47	10^{-6}	A			
		rs760686060:A	tgttgttttt	G→A	attgaattta	6.87	0.62	2.55	0.25	14.81	10^{-6}	A			
		rs765424170:T	tccggcgtct	C→T	ccccgccgcg	121.58	8.32	50.15	3.51	18.09	10^{-6}	A			
		rs767937872:T	tttttgtagc	C→T	atttattgtt	6.87	0.62	4.16	0.39	7.72	10^{-6}	A			
		rs768040755:A	tgcgccaact	C→A	cacgcggggc	24.87	2.71	20.41	2.03	2.68	10^{-2}	C			
		rs770331673:A	gggcagaccc	G→A	ccctccccga	97.72	5.91	57.65	4.19	11.16	10^{-6}	A			
		rs778156916:A	caccctccat	G→A	tgcgccaact	24.87	2.71	11.03	1.18	10.66	10^{-6}	A			
		rs894326790:G	ccggcgtctc	C→G	cccgccgcgt	121.58	8.32	65.48	4.93	12.16	10^{-6}	A			
		rs896783209:C	ggcggatccg	G→C	gccttgagga	97.72	5.91	77.05	7.82	4.02	10^{-3}	B			
		rs896783209:T	ggcggatccg	G→T	gccttgagga	97.72	5.91	29.27	2.94	20.56	10^{-6}	A			
		rs915310331:A	ggctgagacc	G→A	caggccccag	97.72	5.91	63.41	4.50	9.28	10^{-6}	A			
		rs918544130:A	cagactttgt	T→A	gttcaaatgt	12.19	0.79	6.20	0.58	11.95	10^{-6}	A			
		rs923951686:A	gaagggcaga	C→A	acgccctccc	97.72	5.91	71.48	4.76	6.96	10^{-6}	A			
		rs924300242:T	ccttcgtcgt	C→T	gcctccccgc	62.03	4.38	48.47	3.04	5.22	10^{-6}	A			
		rs938322131:A	ccgcccttc	G→A	ggcgggcctc	42.17	3.16	27.09	2.01	8.40	10^{-6}	A			

Table S3. Cont.

#	Human Gene: *NCBI Gene Symbol* (NCBI Gene ID)	Candidate SNP marker: dbSNP ID:min [437]	Candidate SNP marker: 5′ flank, 10 bp	Candidate SNP marker: WT →min	Candidate SNP marker: 3 flank, 10 bp	K_D, nM, *in silico*: WT MEAN	K_D, nM, *in silico*: WT ± SEM	K_D, nM, *in silico*: min MEAN	K_D, nM, *in silico*: min ± SEM	Significance: Z	Significance: p	Significance: ϱ	Δ	Effect of changes in human gene expression on the development of primary open-angle glaucoma (POAG, ☼: "▼" aggravation, "▲" alleviation) [Reference]	☼ ▲▼
18	*B4GALT3* (8703)	rs1013081497:C	accgggtgcc	T→C	cggtgtctgg	41.22	4.11	65.72	4.78	7.56	10^{-6}	A	↓	within a cohort-based biomedical study [118] along with human disease models using Chinese hamster ovary cells [119]: B4GALT3 deficit can lead glycan deficiency in the trabecular meshwork that can contribute to POAG	▼
		rs1043288232:C	ccgggtgcct	A→C	ggtgtctggg	41.22	4.11	65.72	4.78	7.56	10^{-6}	A			
		rs1043288232:G	ccgggtgcct	A→G	ggtgtctggg	41.22	4.11	65.72	4.78	7.56	10^{-6}	A			
		rs1164858268:C	tcattgtttt	A→C	ccttcaatgt	4.15	0.28	5.25	0.44	4.37	10^{-3}	B			
		rs946098592:G	gtgcctagtc	A→G	tctgggaccg	41.22	4.11	52.88	4.58	3.77	10^{-3}	B			
		rs1170763687:A	tgcctagtca	G→A	ctgggaccgg	41.22	4.11	27.6	2.87	5.57	10^{-6}	A	↑	within a retrospective comparative cohort-based biomedical data meta-analysis [120] along with human neoplasia models using a large variety of human cancer cell lines [121]: B4GALT3 excess can elevate risk of neoplasia, which can reduce lifespan in POAG compared to lifespan in other glaucomas	
		rs1258212166:A	gggaccgggt	G→A	acgcggtgtc	41.22	4.11	13.07	1.29	16.35	10^{-6}	A			
		rs1376683142:T	gcctagtcag	C→T	tgggaccggg	41.22	4.11	32.81	3.14	3.30	10^{-3}	B			
		rs1664095727:A	cgcggtgtct	G→A	gcccccggcc	41.22	4.11	28.34	2.81	5.33	10^{-6}	A			
		rs1664154531:T	tcaatgtttc	A→T	tccctccacc	4.15	0.28	3.71	0.26	2.34	0.05	D			
		rs904682878:A	ggaccgggtg	C→A	cgcggtgtct	41.22	4.11	25.24	2.52	6.96	10^{-6}	A			
19	*BMP4* (652)	rs1046130830:A	ggggcctcgg	C→A	ctgccgcggg	36.41	2.82	42.95	2.92	3.20	10^{-2}	C	↓	within human disease models using mice subjected with an artificial glaucoma induction and, next, treated with recombinant adeno-associated virus carrying the human CHRDL1 gene as BMP4-blocker [122]: BMP4-blockade decreases the number of surviving retinal ganglion cells	▼
		rs1239576697:A	taaaacaaca	G→A	tcgttaatga	7.34	0.53	8.22	0.6	2.20	0.05	D			
		rs1328550373:C	ggaaagaaag	A→C	aggaggagga	41.83	2.99	48.25	3.25	2.91	10^{-2}	C			
		rs1334203311:A	gaactgtagg	T→A	agtaactgct	10.93	1.05	15.47	1.56	4.99	10^{-6}	A			
		rs1334203311:C	gaactgtagg	T→C	agtaactgct	10.93	1.05	15.31	1.27	5.33	10^{-6}	A			
		rs1344050675:G	ggtcttgagt	A→G	tccacagcac	20.26	1.76	40.47	2.91	12.27	10^{-6}	A			
		rs1456141352:C	tggtcttgag	T→C	atccacagca	20.26	1.76	54.82	4.23	17.12	10^{-6}	A			
		rs1594795032:G	tggtcgtttt	A→G	ccgaatgctg	5.57	0.42	8.00	0.67	6.42	10^{-6}	A			
		rs1895554953:A	gaaaaacaga	G→A	aggggctgga	21.12	1.41	23.66	1.59	2.41	0.05	D			
		rs544117627:G	cgttaatgat	T→G	gaaccacatt	7.34	0.53	8.71	0.81	2.90	10^{-2}	C			
		rs759247214:G	gcactggtct	T→G	gcagatccac	20.26	1.76	33.68	3.86	7.06	10^{-6}	A			
		rs770567411:C	ttgagtatcc	T→C	cagcactggt	20.26	1.76	22.91	1.9	2.04	0.05	D			
		rs916241282:G	gggctggaag	A→G	gtgactccga	21.12	1.41	36.41	2.82	10.67	10^{-6}	A			
		rs1001159899:A	tgaactgtag	G→A	cagtaactgc	10.93	1.05	6.61	0.64	7.38	10^{-6}	A	↑	according to a cohort-based biomedical study [123]: BMP4 excess is a biomedically proven molecular marker for corneal dystrophy comorbid to POAG [124]	
		rs1032255181:T	gtaactgctt	G→T	aggacatccc	10.93	1.05	8.24	0.66	4.52	10^{-3}	B			
		rs1041688888:T	tcggcgctca	C→T	cgggaggggc	36.41	2.82	20.64	1.66	10.16	10^{-6}	A			
		rs1177418822:A	ccacagcact	G→A	gaagagcaga	20.26	1.76	16.64	1.52	3.12	10^{-2}	C			
		rs1271619816:T	ggggctggaa	G→T	cgtgactccg	21.12	1.41	4.59	0.4	27.87	10^{-6}	A			
		rs1277842500:C	gtcttgagta	T→C	ccacagcact	20.26	1.76	17.78	1.63	2.07	0.05	D			
		rs1278053591:A	cggcgctcac	G→A	gggaggggcc	36.41	2.82	23.39	1.66	8.41	10^{-6}	A			
		rs1278495533:A	gaggaggaag	G→A	ccggggaaga	41.83	2.99	27.52	1.72	8.83	10^{-6}	A			
		rs1322581101:A	cactggtctt	G→A	cagatccaca	20.26	1.76	5.39	0.5	20.81	10^{-6}	A			
		rs1339577528:T	aggaaagaaa	G→T	gaggaggagg	41.83	2.99	10.91	0.86	25.22	10^{-6}	A			
		rs1397454661:A	aggaaggaaa	G→A	ggaagaggag	41.83	2.99	22.49	1.5	12.70	10^{-6}	A			
		rs1465533523:T	ggagtatttg	C→T	aaattcttga	10.93	1.05	7.64	0.66	5.57	10^{-6}	A			
		rs1566585899:A	agaaagaaag	C→A	ggaggaagga	41.83	2.99	36.13	2.41	3.00	10^{-2}	C			
		rs1566587007:C	atttcgttaa	T→C	ttaagaacca	7.34	0.53	5.86	0.41	4.46	10^{-3}	B			
		rs1594795035:T	gctgatggtc	G→T	ggtaaccgaa	5.57	0.42	4.22	0.33	5.14	10^{-6}	A			
		rs1895634484:A	aggagtattt	G→A	aaaattcttg	10.93	1.05	4.77	0.51	11.57	10^{-6}	A			
		rs1895635817:C	ttgaactgta	G→C	ccagtaactg	10.93	1.05	6.83	0.68	6.80	10^{-6}	A			
		rs1895741370:G	aggaggaagg	A→G	cggggaagag	41.83	2.99	37.48	2.39	2.29	0.05	D			
		rs746551997:C	cgaatgctga	T→C	ttcctggtaa	5.57	0.42	4.97	0.38	2.10	0.05	D			
		rs774069849:A	ctggtcttga	G→A	gatccacagc	20.26	1.76	14.62	1.23	5.39	10^{-6}	A			
		rs779603940:A	gttttattat	G→A	tgctgatggt	5.57	0.42	1.80	0.18	17.91	10^{-6}	A			
		rs868713706:T	ttaccggctt	C→T	atgcgggatc	20.26	1.76	12.80	1.09	7.56	10^{-6}	A			
		rs902431707:A	actgtaggta	G→A	taactgcttg	10.93	1.05	8.56	0.78	3.69	10^{-3}	B			
		rs947763057:A	ccgaggggct	G→A	ctcacgtgac	21.12	1.41	16.08	1.24	5.35	10^{-6}	A			

Table S3. Cont.

Human Gene		Candidate SNP marker				K_D, nM, *in silico*				Significance				Effect of changes in human gene expression on the development of primary open-angle glaucoma (POAG, ☼: "▼" aggravation, "▲" alleviation) [Reference]	☼
						WT		min							▲
#	*NCBI Gene Symbol* (NCBI Gene ID)	dbSNP ID:min [437]	5′ flank, 10 bp	WT→min	3 flank, 10 bp	MEAN	± SEM	MEAN	± SEM	Z	p	ϱ	Δ		▼
20	*CACNA2D1* (781)	rs1226247325:G	ccctcttatt	A→G	gccatttttt	1.71	0.17	3.97	0.35	12.73	10^{-6}	A	↓	within cellular models of human eye injuries [125]: CACNA2D1-blockers improve eye injury repair that can relieve POAG	▲
		rs1350974248:C	ggggaataaa	T→C	ctcggcaatc	8.83	0.72	14.14	1.63	6.67	10^{-6}	A			
		rs1407725831:C	aatcggggga	A→C	ttcgcctcgg	8.83	0.72	13.47	1.30	6.68	10^{-6}	A			
		rs1407725831:G	aatcggggga	A→G	ttcgcctcgg	8.83	0.72	12.65	1.31	5.45	10^{-6}	A			
		rs1830689267:C	gtctgtttcc	A→C	ggctcgccga	41.46	3.03	57.33	4.21	6.26	10^{-6}	A			
		rs369068443:C	ttgccctctt	A→C	aatgccattt	1.71	0.17	2.71	0.28	6.39	10^{-6}	A			
		rs532460440:C	tgccctctta	T→C	atgccatttt	1.71	0.17	2.27	0.29	3.50	10^{-3}	B			
		rs552469490:C	ttttgccctc	T→C	aaaatgccat	1.71	0.17	2.02	0.22	2.24	0.05	D			
		rs775251843:G	tttgccctct	T→G	aaatgccatt	1.71	0.17	3.97	0.35	12.73	10^{-6}	A			
		rs896670849:C	gggggaataa	A→C	cctcggcaat	8.83	0.72	14.12	1.36	7.42	10^{-6}	A			
		rs1012262350:T	gcaatcgggg	G→T	ccttcgcctc	8.83	0.72	5.12	0.40	9.68	10^{-6}	A	↑	within cellular models of human diseases using transgenic mice overexpressing Cacna2d1 [126]: pain hypersensitivity that can worsen POAG	▼
		rs1230138813:T	ggaggaaacc	G→T	gaataaatca	41.46	3.03	34.55	3.03	3.20	10^{-2}	C			
		rs1314431952:T	tttccaaagt	C→T	gccgaggtct	41.46	3.03	33.37	2.36	4.27	10^{-3}	B			
		rs1339559275:T	tccaaagtcg	C→T	cgaggtctgt	41.46	3.03	30.51	2.14	6.06	10^{-6}	A			
		rs1396066282:T	ggtctgtttc	C→T	gggctcgccg	41.46	3.03	10.26	0.85	25.26	10^{-6}	A			
		rs1406026667:A	aatcagggag	G→A	tcgggggaat	41.46	3.03	29.35	2.18	6.64	10^{-6}	A			
		rs1563560923:A	ttccaaagtc	G→A	ccgaggtctg	41.46	3.03	35.82	2.56	2.86	10^{-2}	C			
		rs1830689775:A	ggctcgccga	G→A	gagcgccggc	41.46	3.03	37.02	2.68	2.21	0.05	D			
		rs935535188:A	cggcaatcgg	G→A	cgccttcgcc	8.83	0.72	7.67	0.64	2.41	0.05	D			
21	*CAT* (847)	rs567620272:G	ccgagcagcc	A→G	atcagaaggc	43.29	2.97	47.89	3.40	2.04	0.05	D	↓	within a cohort-based blood serum and tear study [127]: catalase insufficiency in lacrimal fluid can be a biomedical molecular marker of free radical oxidation activation, antioxidant defense system suppression and endothelium dysfunction that altogether can worsen POAG	▼
		rs1055680029:A	agcctgaagt	C→A	gccacggact	43.29	2.97	25.56	2.45	8.93	10^{-6}	A	↑	within in vitro cellular POAG models using human trabecular meshwork cell line subjected with extracellular vesicles derived from the non-pigmented ciliary epithelium [128]: catalase upregulation reduced oxidative stress and thus alleviated POAG	▲
		rs1191617083:A	agaaggcagt	C→A	ctcccgaggg	43.29	2.97	29.13	3.09	6.27	10^{-6}	A			
		rs1590295799:T	ttggctgagc	C→T	tgaagtcgcc	43.29	2.97	35.66	2.64	3.84	10^{-3}	B			
		rs958064048:T	agcagccaat	C→T	agaaggcagt	43.29	2.97	13.51	1.06	22.35	10^{-6}	A			
22	*CAV1* (857)	rs1387525753:G	ttccccccat	A→G	caatacaaga	8.28	0.62	14.25	1.33	9.11	10^{-6}	A	↓	within human disease models using Cav1-knockout mice [129]: blood-retinal barrier breakdown, mural cell alteration, and venous enlargement with branch veins being frequent sites of breakdown that altogether may contribute to retinal disorders with vascular pathologies, including retinopathy, uveoretinitis, and POAG	▼
		rs1406325411:A	cgcgggcggg	G→A	gccttcggac	126.18	10.34	143.49	11.81	2.21	0.05	D			
		rs1441495858:G	tccccccata	C→G	aatacaagat	8.28	0.62	12.28	0.92	7.48	10^{-6}	A			
		rs1664923757:C	gtggagggac	A→C	agagagggcc	32.02	2.24	79.32	6.02	17.56	10^{-6}	A			
		rs1793523406:C	tttcccccca	T→C	acaatacaag	8.28	0.62	10.48	0.79	4.44	10^{-3}	B			
		rs1793547267:G	gagggacaag	A→G	gagggccgag	32.02	2.24	56.59	4.39	10.9	10^{-6}	A			
		rs913921938:C	gggggccttc	G→C	gaccgcgcgg	126.18	10.34	142.13	9.86	2.22	0.05	D			
		rs971191431:T	acaagagagg	G→T	ccgaggcagg	32.02	2.24	35.43	2.54	2.02	0.05	D			

Table S3. Cont.

Human Gene		Candidate SNP marker				K_D, nM, *in silico*				Significance				Effect of changes in human gene expression on the development of primary open-angle glaucoma (POAG, ☼: "▼" aggravation, "▲" alleviation) [Reference]	☼ ▲ ▼
#	*NCBI Gene Symbol* (NCBI Gene ID)	dbSNP ID:min [437]	5′ flank, 10 bp	WT→min	3 flank, 10 bp	WT MEAN	± SEM	min MEAN	± SEM	Z	p	ϱ	Δ		
22	*CAV1* (857)	rs1020021753:T	tttttccccc	C→T	atacaataca	8.28	0.62	4.29	0.38	11.36	10^{-6}	A	↑	within human POAG cellular models using human embryonic kidney cell line HEK293 transfected by vector with one more mice Cav1 gene [130]: caveolin-1 excess along with protection against lipotoxicity, which is risk factor for elevated intraocular pressure aggravating POAG [131]	▲
		rs1042958593:A	ctttttttcc	C→A	cccatacaat	10.96	0.66	9.38	0.63	3.43	10^{-3}	B			
		rs1042958593:T	ctttttttcc	C→T	cccatacaat	10.96	0.66	9.76	0.65	2.57	0.05	D			
		rs1046527816:A	ggcggggcct	G→A	ccctgacccc	126.18	10.34	109.36	9.77	2.36	0.05	D			
		rs113695532:A	ggcgggggag	G→A	caggcgcgcc	126.18	10.34	98.21	8.04	4.33	10^{-3}	B			
		rs1156581949:G	ttttcccccc	A→G	tacaatacaa	8.28	0.62	6.85	0.55	3.46	10^{-3}	B			
		rs1193135483:T	acaagatctt	C→T	cttcctcagt	8.28	0.62	7.18	0.57	2.63	10^{-2}	C			
		rs1235829774:A	gccttcggac	C→A	gcgcggcggg	126.18	10.34	99.96	7.13	4.29	10^{-3}	B			
		rs1356098194:T	gcgggggagg	C→T	aggcgcgccc	126.18	10.34	59.4	5.69	11.96	10^{-6}	A			
		rs1387525753:T	ttccccccat	A→T	caatacaaga	10.96	0.66	8.72	0.74	4.37	10^{-3}	B			
		rs1472403378:C	ggggccttcg	G→C	accgcgcggc	126.18	10.34	102.42	8.09	3.67	10^{-3}	B			
		rs1584766232:A	ttcggaccgc	G→A	cggcggggcc	126.18	10.34	107.55	7.90	2.90	10^{-2}	C			
		rs1793523530:A	ccatacaata	C→A	aagatcttcc	8.28	0.62	4.21	0.37	11.81	10^{-6}	A			
		rs1793523568:G	acaatacaag	A→G	tcttccttcc	8.28	0.62	6.41	0.50	4.74	10^{-3}	B			
		rs1793523672:C	atacaagatc	T→C	tccttcctca	8.28	0.62	6.68	0.52	3.97	10^{-3}	B			
		rs1793523717:G	tacaagatct	T→G	ccttcctcag	8.28	0.62	6.63	0.53	4.10	10^{-3}	B			
		rs1793554542:A	gccggggcgt	G→A	cgcgggcggg	126.18	10.34	64.46	7.55	9.40	10^{-6}	A			
		rs529037774:A	gcgccttttt	T→A	tccccccata	8.28	0.62	6.02	0.52	5.61	10^{-6}	A			
		rs548665630:T	tcccttaaag	C→T	acagcccagg	8.28	0.62	5.89	0.83	4.29	10^{-3}	B			
		rs905272582:T	ggggcctgcc	C→T	tgaccccctgg	126.18	10.34	85.52	6.39	7.01	10^{-6}	A			
		rs913921938:A	gggggccttc	G→A	gaccgcgcgg	126.18	10.34	49.87	3.99	16.22	10^{-6}	A			
		rs949374493:T	tcggaccgcg	C→T	ggcggggcct	126.18	10.34	98.96	7.08	4.47	10^{-3}	B			
		rs968099566:T	gtgttattta	C→T	ccgagtcctg	5.84	0.59	4.83	0.41	2.88	10^{-2}	C			
		rs986253282:A	cgggtcctgc	G→A	tgctgagccg	126.18	10.34	100.03	6.93	4.33	10^{-3}	B			
		rs988770352:A	cgggggcctt	C→A	ggaccgcgcg	126.18	10.34	44.93	4.09	16.86	10^{-6}	A			
23	*CAV2* (858)	rs1253583599:G	ccgggcgcta	C→G	gggaagggga	66.84	5.51	99.06	9.15	6.35	10^{-6}	A	↓	within a biomedical cohort-based comparative study of minor G versus ancestral T alleles of the known biomedical SNP-marker rs17588172 for POAG using Korean participants [132]: minor G allele downregulates CAV2 that elevates intraocular pressure worsening POAG	▼
		rs1793039672:C	gccgggcgct	A→C	cgggaagggg	66.84	5.51	99.06	9.15	6.35	10^{-6}	A			
		rs1793039804:C	cgggcgctac	G→C	ggaaggggag	66.84	5.51	75.53	6.42	2.06	0.05	D			
		rs1001897642:C	cgcagggcta	G→C	gcgaggcgag	66.84	5.51	53.35	5.3	3.49	10^{-3}	B	↑	within cellular POAG models using differentiated rat PC12 cell line [133]: caveolin-2 is up-regulated in response to the mechanical injury of differentiated PC12 cells that can improve post-traumatic neuron repair retarding POAG development	▲
		rs1187801213:A	ggcgctacgg	G→A	aaggggaggc	66.84	5.51	36.52	3.26	9.95	10^{-6}	A			
		rs1253583599:A	ccgggcgcta	C→A	gggaagggga	66.84	5.51	57.26	4.57	2.7	10^{-2}	C			
		rs1273674888:T	cagggctagg	C→T	gaggcgaggg	66.84	5.51	28.39	2.77	13.4	10^{-6}	A			
		rs1386620414:A	gctacgggaa	G→A	gggaggccgc	66.84	5.51	49.29	3.93	5.31	10^{-6}	A			
		rs1478850025:A	cggggccggg	C→A	gctacgggaa	66.84	5.51	57.88	5.01	2.41	0.05	D			
		rs1793037769:A	ggcctcgcag	G→A	gctaggcgag	66.84	5.51	53.37	4.95	3.63	10^{-3}	B			
		rs1793037837:A	gcctcgcagg	G→A	ctaggcgagg	66.84	5.51	40.48	3.51	8.39	10^{-6}	A			
		rs1793039804:A	cgggcgctac	G→A	ggaaggggag	66.84	5.51	26.79	2.38	15.11	10^{-6}	A			
		rs1793039870:A	gggcgctacg	G→A	gaaggggagg	66.84	5.51	21.54	1.84	19.06	10^{-6}	A			

Table S3. Cont.

#	Human Gene: *NCBI Gene Symbol* (NCBI Gene ID)	Candidate SNP marker: dbSNP ID:min [437]	5′ flank, 10 bp	WT →min	3 flank, 10 bp	K_D, nM, *in silico*: WT MEAN±	SEM	min MEAN±	SEM	Significance: Z	p	ϱ	Δ	Effect of changes in human gene expression on the development of primary open-angle glaucoma (POAG, ☼: "▼" aggravation, "▲" alleviation) [Reference]	☼ ▲ ▼
24	*CDC7* (8317)	rs1001284914:C	gtgcagttcc	T→C	tttcccgccc	93.48	6.30	116.35	8.54	4.39	10^{-3}	B	↓	within a cohort-based study [248]: CDC7-blockers within a gene-target therapy can retard atherogenesis as a risk-factor of POAG [135]	▲
		rs1160843404:G	cgagggcttt	A→G	gtgtgcgcat	10.16	1.01	40.62	3.27	21.62	10^{-6}	A			
		rs1160843404:T	cgagggcttt	A→T	gtgtgcgcat	10.16	1.01	13.35	1.08	4.25	10^{-3}	B			
		rs1246335464:C	gccgtgcagt	T→C	ccttttcccg	93.48	6.30	113.41	7.83	4.01	10^{-3}	B			
		rs1246335464:G	gccgtgcagt	T→G	ccttttcccg	93.48	6.30	113.87	9.03	3.79	10^{-3}	B			
		rs1557582324:G	gcctgcgcac	T→G	aagcatctgg	26.76	2.11	58.57	4.78	13.81	10^{-6}	A			
		rs1666637539:G	gcgcctgcgc	A→G	ctaagcatct	26.76	2.11	38.57	4.15	5.48	10^{-6}	A			
		rs1666638024:G	cctgcgcact	A→G	agcatctggt	26.76	2.11	64.25	4.37	16.83	10^{-6}	A			
		rs934631771:G	agggctttag	T→G	gtgcgcatgt	10.16	1.01	28.98	2.63	15.53	10^{-6}	A			
		rs1026663566:T	ttttctcgcc	G→T	tgcagttcct	93.48	6.30	80.62	6.03	2.94	10^{-2}	C	↑	within a cohort-based biomedical study [136]: Cdc7 overexpression contributes to DNA-damage resistance that can alleviate POAG [137]	
		rs1035805520:A	cccttcggat	C→A	cctccgacct	93.48	6.30	31.93	3.53	16.60	10^{-6}	A			
		rs1051529163:G	cagttccttt	T→G	cccgcccccc	93.48	6.30	79.14	5.53	3.43	10^{-3}	B			
		rs1223142871:T	cgcccccctt	C→T	ggatccctcc	93.48	6.30	69.37	5.44	5.77	10^{-6}	A			
		rs1242010226:G	cactaagcat	C→G	tggttcggtc	26.76	2.11	23.90	1.66	2.15	0.05	D			
		rs1242010226:T	cactaagcat	C→T	tggttcggtc	26.76	2.11	23.95	1.96	1.96	0.05	D			
		rs1281030771:T	cgcctgcgca	C→T	taagcatctg	26.76	2.11	19.31	1.46	5.99	10^{-6}	A			
		rs1303380094:T	cgtgcagttc	C→T	ttttcccgcc	93.48	6.30	46.24	3.25	14.46	10^{-6}	A			
		rs1359312864:T	tctcgccgtg	C→T	agttcctttt	93.48	6.30	24.00	2.21	23.86	10^{-6}	A			
		rs1408091950:A	gtcgagggct	T→A	tagtgtgcgc	10.16	1.01	7.93	1.00	3.08	10^{-2}	C			
		rs1666610802:T	gggtcgaggg	C→T	tttagtgtgc	10.16	1.01	8.89	0.81	1.98	0.05	D			
		rs1666629801:T	ggttttctcg	C→T	cgtgcagttc	93.48	6.30	44.45	2.99	15.62	10^{-6}	A			
		rs1666631026:T	ccgtgcagtt	C→T	cttttcccgc	93.48	6.30	54.81	3.88	10.93	10^{-6}	A			
		rs1666632077:T	ttccttttcc	C→T	gcccccccttc	93.48	6.30	58.35	4.29	9.45	10^{-6}	A			
		rs1666632268:A	tccttttccc	G→A	cccccccttcg	93.48	6.30	84.50	5.80	2.10	0.05	D			
		rs1666632268:T	tccttttccc	G→T	cccccccttcg	93.48	6.30	81.22	5.70	2.89	10^{-2}	C			
		rs1666634902:T	ccttcggatc	C→T	ctccgacctg	93.48	6.30	83.64	6.47	2.17	0.05	D			
		rs1666636468:A	acctgcggcg	G→A	gaaagtcgcg	93.48	6.30	40.93	2.93	16.78	10^{-6}	A			
		rs184644681:A	agttcctttt	C→A	ccgccccccct	93.48	6.30	15.55	1.35	32.69	10^{-6}	A			
		rs184644681:T	agttcctttt	C→T	ccgccccccct	93.48	6.30	59.06	4.03	9.57	10^{-6}	A			
		rs371046438:A	cggttttctc	G→A	ccgtgcagtt	93.48	6.30	53.66	3.52	11.80	10^{-6}	A			
		rs371046438:T	cggttttctc	G→T	ccgtgcagtt	93.48	6.30	66.30	5.05	6.75	10^{-6}	A			
		rs547148264:T	gcgcactaag	C→T	atctggttcg	26.76	2.11	5.84	0.67	21.98	10^{-6}	A			
		rs905713669:A	ttctcgccgt	G→A	cagttccttt	93.48	6.30	29.10	3.22	18.03	10^{-6}	A			
		rs970216851:T	ccccccttcg	G→T	atccctccga	93.48	6.30	16.21	1.54	30.10	10^{-6}	A			
		rs992203228:T	gcggcgggaa	A→T	gtcgcgcgcc	93.48	6.30	77.12	6.33	3.62	10^{-3}	B			
25	*CDH1* (999)	rs1457876601:T	caggtgaacc	C→T	tcagccaatc	44.81	5.39	55.40	5.53	2.72	10^{-2}	C	↓	within human disease cellular models [138]: CDH1 insufficiency as a molecular marker for differentiation to epithelial cells can elevate risks of epithelial to mesenchymal transition and, thus, fibrosis as a post-POAG-surgery complication	▼
		rs1962411937:G	agccaatcag	C→G	ggtacggggg	44.81	5.39	63.30	6.74	4.30	10^{-3}	B			
		rs1962412008:G	aatcagcggt	A→G	cgggggcgg	44.81	5.39	74.01	5.96	6.94	10^{-6}	A			
		rs34500817:G	atcagcggta	C→G	ggggggcggt	44.81	5.39	52.84	5.72	2.04	0.05	D			
		rs557942201:T	ggtgaaccct	C→T	agccaatcag	44.81	5.39	55.40	5.53	2.72	10^{-2}	C			

Table S3. Cont.

Human Gene		Candidate SNP marker				K_D, nM, *in silico*				Significance				Effect of changes in human gene expression on the development of primary open-angle glaucoma (POAG, ☼: "▼" aggravation, "▲" alleviation) [Reference]	☼
#	*NCBI Gene Symbol* (NCBI Gene ID)	dbSNP ID:min [437]	5′ flank, 10 bp	WT →min	3 flank, 10 bp	WT MEAN±	WT SEM	min MEAN±	min SEM	Z	p	ϱ	Δ		▲ ▼
25	*CDH1* (999)	rs1157212868:A	agcggtacgg	G→A	gggcggtgcc	44.81	5.39	37.17	3.69	2.40	0.05	D	↑	within human disease cellular models [138]: CDH1 excess as a molecular marker for differentiation to epithelial cells can prevent epithelial to mesenchymal transition and, thus, fibrosis as a post-POAG-surgery complication	▲
		rs1177347058:G	tcagttcaga	C→G	tccagcccgc	48.03	3.76	30.52	2.29	8.36	10^{-6}	A			
		rs1208427103:A	tcagtggcgt	C→A	ggaactgcaa	48.03	3.76	35.09	3.27	5.16	10^{-6}	A			
		rs1213481634:A	gaactgcaaa	G→A	cacctgtgag	48.03	3.76	37.85	2.75	4.46	10^{-3}	B			
		rs1336812842:G	tgcaaagcac	C→G	tgtgagcttg	48.03	3.76	31.74	2.51	7.44	10^{-6}	A			
		rs1336812842:T	tgcaaagcac	C→T	tgtgagcttg	48.03	3.76	28.96	2.00	9.68	10^{-6}	A			
		rs1354393862:A	gcacctgtga	G→A	cttgcggaag	48.03	3.76	33.47	2.89	6.19	10^{-6}	A			
		rs1382294724:T	cagcggtacg	G→T	ggggcggtgc	44.81	5.39	18.13	1.95	11.23	10^{-6}	A			
		rs1429353892:T	tgcagccacg	C→T	accccctctc	44.81	5.39	24.07	2.69	7.58	10^{-6}	A			
		rs1481940627:A	gtgagcttgc	G→A	gaagtcagtt	48.03	3.76	40.13	2.95	3.35	10^{-3}	B			
		rs1824814858:T	cacctgtgag	C→T	ttgcggaagt	48.03	3.76	32.62	2.96	6.46	10^{-6}	A			
		rs1962416724:T	cgtcggaact	G→T	caaagcacct	48.03	3.76	26.83	2.18	10.33	10^{-6}	A			
		rs1962416981:T	ctgcaaagca	C→T	ctgtgagctt	48.03	3.76	35.03	2.66	5.78	10^{-6}	A			
		rs1962417789:A	cttgcggaag	T→A	cagttcagac	48.03	3.76	42.94	2.90	2.17	0.05	D			
		rs34500817:A	atcagcggta	C→A	ggggggcggt	44.81	5.39	31.23	2.73	4.86	10^{-3}	B			
		rs34500817:T	atcagcggta	C→T	ggggggcggt	44.81	5.39	30.22	2.90	5.13	10^{-6}	A			
		rs566244764:A	gccggcaggt	G→A	aaccctcagc	44.81	5.39	19.87	2.58	9.20	10^{-6}	A			
		rs777789988:T	aactgcaaag	C→T	acctgtgagc	48.03	3.76	17.11	1.82	15.60	10^{-6}	A			
		rs893677325:T	ctctcagtgg	C→T	gtcggaactg	48.03	3.76	34.64	2.20	6.48	10^{-6}	A			
26	*CDKN1A* (1026)	rs1582561578:T	gcggttgtat	A→T	tcagggccgc	2.71	0.32	7.76	0.76	13.79	10^{-6}	A	↓	according to a comprehensive retrospective biomedical review [139]: CDKNA1A-deficiency can complicate wound healing after glaucoma filtration surgery that may be prevented due to an adjunctive ocular gene therapy using an adenovirus vector containing an additional copy of the human CDKN1A gene	▼
		rs1761775879:C	ggcggttgta	T→C	atcagggccg	2.71	0.32	6.50	0.76	10.52	10^{-6}	A			
		rs1761775879:G	ggcggttgta	T→G	atcagggccg	2.71	0.32	9.94	1.02	16.65	10^{-6}	A			
		rs934486987:C	gttgtatatc	A→C	gggccgcgct	2.71	0.32	4.51	0.60	5.75	10^{-6}	A			
		rs1173187137:A	tgcttcccag	G→A	aacatgcttg	60.41	4.40	39.33	2.82	8.40	10^{-6}	A	↑	within human disease models using human lung fibroblast strain CRL-1262 under UV-irradiation [140]: UV dose-dependent overexpression of CDKN1A along with either cell survival due to this dose-dependent excess of CDKN1A arresting the cell cycle until DNA damage caused by UV radiation is successfully repaired, or CDKN1A-induced cell death if DNA damage in question appears to be too great and unrepairable	▲
		rs1209621011:A	tcaggaacat	G→A	tcccaacatg	22.48	2.04	9.74	1.02	12.07	10^{-6}	A			
		rs124232928:A	tggccgtcag	G→A	aacatgtccc	60.41	4.40	42.88	3.45	6.32	10^{-6}	A			
		rs1373855119:T	cctatgctgc	C→T	tgcttcccag	60.41	4.40	51.87	3.84	2.94	10^{-6}	A			
		rs1420544594:T	caggctgtgg	C→T	tctgattggc	60.41	4.40	51.45	3.51	3.22	10^{-6}	A			
		rs1444333768:T	tggctttctg	G→T	ccgtcaggaa	60.41	4.40	35.49	2.54	10.41	10^{-6}	A			
		rs149655211:A	gctttctggc	C→A	gtcaggaaca	60.41	4.40	50.19	3.73	3.56	10^{-6}	A			
		rs149655211:T	gctttctggc	C→T	gtcaggaaca	60.41	4.40	48.24	3.58	4.32	10^{-6}	A			
		rs1562034779:A	ttctggccgt	C→A	aggaacatgt	60.41	4.40	40.04	3.30	7.48	10^{-6}	A			
		rs1582557039:G	aggaacatgc	T→G	tgggcagcag	60.41	4.40	48.21	3.79	4.21	10^{-6}	A			
		rs1582557105:A	ctggccgtca	G→A	gaacatgtcc	60.41	4.40	49.53	3.24	4.06	10^{-6}	A			
		rs1761695842:T	ccgtcaggaa	C→T	atgtcccaac	22.48	2.04	9.08	0.89	13.6	10^{-6}	A			
		rs54292311:C	ggcagcaggc	T→C	gtggctctga	60.41	4.40	54.07	3.61	2.24	10^{-6}	A			
		rs891719643:T	ggttgtatat	C→T	agggccgcgc	2.71	0.32	1.96	0.20	4.14	10^{-6}	A			
27	*CDKN2A* (1029)	rs1189898349:C	gctcagatgc	T→C	gaggaagaag	52.84	3.97	62.39	4.59	3.16	10^{-2}	C	↓	within a retrospective comprehensive complex GWAS and QTL meta-analysis [141]: three biomedically proven SNP-markers rs1063192, rs7865618 and rs2157719 for POAG are associated with CDKN2A decreased expression gene in the cerebral cortex	▼
		rs1198105973:G	cccctttgct	A→G	gtctgtccct	15.68	1.43	38.70	2.88	15.34	10^{-6}	A			
		rs1232186316:A	tcccagtctg	C→A	cggtgcgtgg	14.89	1.22	18.31	1.44	3.64	10^{-3}	B			
		rs1819741705:G	agttcggagg	A→G	agctccgcag	4.01	0.41	5.83	0.65	4.93	10^{-6}	A			
		rs1819745495:C	tttgctattt	T→C	gtccctgccc	15.68	1.43	20.04	2.05	3.58	10^{-3}	B			
		rs1820598663:T	gaaaataaaa	A→T	aggttaaaac	6.76	0.56	8.40	0.79	3.49	10^{-3}	B			
		rs1820598964:G	aaaccgaaaa	T→G	cgtgaaggtt	6.76	0.56	9.30	0.73	5.63	10^{-6}	A			

Table S3. Cont.

#	Human Gene: *NCBI Gene Symbol* (NCBI Gene ID)	Candidate SNP marker: dbSNP ID:min [437]	5′ flank, 10 bp	WT →min	3 flank, 10 bp	K_D, nM, *in silico*: WT MEAN	± SEM	min MEAN	± SEM	Significance: Z	p	ϱ	Δ	Effect of changes in human gene expression on the development of primary open-angle glaucoma (POAG, ☼: "▼" aggravation, "▲" alleviation) [Reference]	☼ ▲ ▼
		rs1001094833:T	ggaagaagcg	C→T	aagaggaaag	52.84	3.97	36.14	2.58	7.33	10^{-6}	A			
		rs1016480088:C	accggaggaa	G→C	cttccgccag	31.43	2.21	19.10	1.36	9.98	10^{-6}	A			
		rs1016480088:T	accggaggaa	G→T	cttccgccag	31.43	2.21	6.83	0.58	27.81	10^{-6}	A			
		rs1212618363:T	gcgcgctcag	G→T	gtgggggcg	89.08	6.74	53.92	4.03	9.44	10^{-6}	A			
		rs1232186316:A	tcccagtctg	C→A	cggtgcgtgg	89.08	6.74	44.38	3.36	13.02	10^{-6}	A			
		rs1234805040:A	ccagtctgca	G→A	gtgcgtgggt	89.08	6.74	79.15	5.60	2.28	0.05	D			
		rs1252803244:A	tggaattcat	T→A	ggaggatata	4.01	0.41	3.34	0.31	2.63	10^{-2}	C			
		rs1317459567:C	aagttcggag	G→C	cagctccgca	4.01	0.41	3.30	0.37	2.57	0.05	D			
		rs1333586229:T	gtgggtccca	G→T	gggggcggtg	89.08	6.74	54.74	4.28	8.95	10^{-6}	A			
		rs1343260418:A	cctgcggggc	G→A	gcgggtgcgc	89.08	6.74	67.85	5.04	5.14	10^{-6}	A			
		rs1359374454:A	agtctgcagt	T→A	gcgtgggtcc	14.89	1.22	12.34	0.96	3.33	10^{-3}	B			
		rs1417624311:T	gggggcggtg	C→T	ggagatgggc	89.08	6.74	49.03	4.21	10.44	10^{-6}	A			
		rs1587359061:T	gcctgcgggg	C→T	ggcgggtgcg	89.08	6.74	66.58	5.28	5.31	10^{-6}	A			
		rs1819745897:G	tgccccttttg	C→G	cagtctgtcc	15.68	1.43	12.81	1.49	2.73	10^{-2}	C			
		rs1819746025:A	ctgcccccttt	G→A	tcagtctgtc	15.68	1.43	5.22	0.51	16.46	10^{-6}	A			
		rs1819975655:A	gaggaagaaa	G→A	cgccagcacc	31.43	2.21	20.75	1.40	8.52	10^{-6}	A			
		rs1819976200:T	agcaccggag	G→T	gctcttccgc	31.43	2.21	13.36	1.05	16.23	10^{-6}	A			
27	*CDKN2A* (1029)	rs1820600916:T	tcagatgctc	C→T	ggaagaagcg	52.84	3.97	44.94	3.4	3.04	10^{-2}	C		according to comprehensive biomedical review [142]: CDKN2A excess can prevent atherosclerosis aggravating POAG [143]	▲
		rs1820601308:A	gcgctcagat	G→A	aagaggaaga	52.84	3.97	13.25	1.37	21.63	10^{-6}	A	↑		
		rs1820601788:A	aggaagaagc	G→A	gaagaggaaa	52.84	3.97	45.41	3.24	2.92	10^{-2}	C			
		rs1820602507:T	gaaagaggaa	G→T	cgggggaaga	52.84	3.97	26.67	2.36	11.77	10^{-6}	A			
		rs1820603714:T	gcgggggaag	A→T	gggaggggag	52.84	3.97	39.66	3.22	5.19	10^{-6}	A			
		rs372430243:A	aagaagcgct	C→A	gaggaaagag	52.84	3.97	33.11	2.48	8.82	10^{-6}	A			
		rs565075759:T	ggctggtcac	C→T	gaggaggggc	31.43	2.21	20.48	1.87	7.44	10^{-6}	A			
		rs753059687:A	gcggagatgg	G→A	gcgcctgcgg	89.08	6.74	79.65	6.02	2.09	0.05	D			
		rs766025515:T	cggagatggg	C→T	cgcctgcggg	89.08	6.74	62.35	5.12	6.39	10^{-6}	A			
		rs768642051:T	agggggcggt	G→T	cggagatggg	89.08	6.74	70.69	6.56	3.86	10^{-3}	B			
		rs770015235:A	gggtcccagt	C→A	gggcggtgcg	89.08	6.74	22.08	2.14	22.69	10^{-6}	A			
		rs770015235:G	gggtcccagt	C→G	gggcggtgcg	89.08	6.74	59.87	4.97	7.08	10^{-6}	A			
		rs771139743:A	tgcagttaag	G→A	gggtcccagt	14.89	1.22	11.51	1.01	4.29	10^{-3}	B			
		rs775186150:T	gcgtgggtcc	C→T	caggggggcgg	89.08	6.74	31.39	2.78	17.90	10^{-6}	A			
		rs781158752:T	cgggcgcgct	C→T	aaggtggggg	89.08	6.74	57.54	4.85	7.72	10^{-6}	A			
		rs781358597:A	ctgcagttaa	G→A	tgggtcccag	14.89	1.22	9.97	1.39	4.97	10^{-6}	A			
		rs906120178:A	cgccagcacc	G→A	gggggctctt	31.43	2.21	28.09	1.99	2.26	0.05	D			
		rs931838144:A	cgggggaaga	G→A	ggaggggagc	52.84	3.97	32.31	2.25	9.60	10^{-6}	A			
		rs940346959:T	cgaaaataaa	A→T	aaggttaaaa	6.76	0.56	5.10	0.45	4.63	10^{-3}	B			
		rs1821376108:G	ggcccagctg	A→G	gccggcccct	35.71	2.41	45.68	3.99	4.46	10^{-3}	B	↓	within human POAG models using Cdkn2b-knockout mice [144]: more vulnerable to retinal ganglion cell loss in response to elevated intraocular pressure that can aggravate POAG	▼
		rs1234675805:A	cggagcctaa	G→A	cggacagggg	35.71	2.41	24.11	2.06	7.21	10^{-6}	A			
		rs1466837558:A	tggcccagct	G→A	cgccggcccc	35.71	2.41	13.18	1.19	17.65	10^{-6}	A			
		rs1466837558:C	tggcccagct	G→C	cgccggcccc	35.71	2.41	29.69	1.85	4.01	10^{-3}	B			
28	*CDKN2B* (1030)	rs1821376565:T	ccccttggcc	C→T	ggagacgccg	35.71	2.41	30.05	2.07	3.57	10^{-3}	B		within a phytopharmaceutical microarray-based transcriptome study using murine microglial cells treated with 15-methoxypinusolidic acid as a modern promising anti-POAG phytomedication extracted from Biota orientalis [145]: Cdkn2b excess causing microglia cell cycle arrest, which can inhibit proliferation of microglia cells and induce apoptosis of these cells that may slow down neurodegeneration of both optic nerve and retina	▲
		rs1821379948:A	ggagcctaag	G→A	ggacaggggg	35.71	2.41	27.58	2.37	4.73	10^{-3}	B			
		rs1821379948:T	ggagcctaag	G→T	ggacaggggg	35.71	2.41	18.94	1.82	10.81	10^{-6}	A	↑		
		rs191200920:T	gctgaaaacg	G→T	cccttggccc	35.71	2.41	14.82	1.35	15.51	10^{-6}	A			
		rs2069419:A	cttggcccag	C→A	gacgccggcc	35.71	2.41	22.23	1.44	10.12	10^{-6}	A			
		rs2069419:T	cttggcccag	C→T	gacgccggcc	35.71	2.41	21.86	1.54	10.07	10^{-6}	A			
		rs920584435:A	tccccactct	G→A	tgccggctgg	35.71	2.41	17.95	1.81	11.32	10^{-6}	A			

Table S3. Cont.

#	Human Gene: *NCBI Gene Symbol* (NCBI Gene ID)	Candidate SNP marker: dbSNP ID:min [437]	5′ flank, 10 bp	WT →min	3 flank, 10 bp	K_D, nM, *in silico*: WT MEAN	± SEM	min MEAN	± SEM	Significance: Z	p	ϱ	Δ	Effect of changes in human gene expression on the development of primary open-angle glaucoma (POAG, ☼: "▼" aggravation, "▲" alleviation) [Reference]	☼ ▲ ▼
29	*CNTNAP4* (85445)	rs1026016066:C	tgcttcctca	T→C	tttctcttgc	27.96	1.83	31.00	2.11	2.18	0.05	D	↓	within a cohort-based biomedical study [146]: CNTNAP4-deficit is a biomedical molecular marker for major depressive disorder, which is comorbid with POAG at least as an age-related developmental neurodegenerative disorder [147]	▼
		rs1204751656:G	tggcagagct	A→G	ctgagaagag	19.16	1.73	22.09	1.52	2.50	0.05	D			
		rs1451577024:G	ttgctgcttc	C→G	tcattttctc	27.96	1.83	30.72	2.01	2.03	0.05	D			
		rs1958508079:C	tattggaaaa	T→C	agaagagaag	13.66	1.17	15.37	1.22	2.03	0.05	D			
		rs1958523937:G	gcagagctac	T→G	gagaagagga	19.16	1.73	22.09	1.52	2.50	0.05	D			
		rs1961712450:C	tgtgaatctt	T→C	tataatgagg	3.06	0.38	4.10	0.42	3.64	10^{-3}	B			
		rs537619036:T	ctactgagaa	G→T	aggactggag	19.16	1.73	22.09	1.52	2.50	0.05	D			
		rs890321242:G	tgaatctttt	A→G	taatgaggta	3.06	0.38	4.16	0.36	4.05	10^{-3}	B			
		rs1028883570:A	agctggcaga	G→A	ctactgagaa	19.16	1.73	12.18	2.08	4.69	10^{-3}	B	↑	within a biomedical cohort-based transcriptome study of neurodegenerative diseases systematized using five non-overlapping clusters by means of multiple sclerosis severity degrees according to magnetic resonance imaging scans [148]: CNTNAP4 upregulation was detected in the only inactive form of the mildest smallest neurodegenerations, which can themselves heal over time, that may correspond the slowest neurodegeneration of optic nerve alleviating POAG	▲
		rs1044625613:T	ttttctcttg	C→T	agccgccatg	27.96	1.83	11.43	0.86	17.88	10^{-6}	A			
		rs1055095141:A	atgcttgtta	G→A	gaaagatgct	19.50	1.51	15.18	1.08	4.75	10^{-3}	B			
		rs1189699255:T	aattttcata	C→T	agttaaattt	3.06	0.38	1.93	0.24	5.25	10^{-6}	A			
		rs1193748391:A	gctgcttcct	C→A	attttctctt	27.96	1.83	25.02	1.72	2.34	0.05	D			
		rs1193748391:T	gctgcttcct	C→T	attttctctt	27.96	1.83	12.37	0.99	15.79	10^{-6}	A			
		rs1309677971:T	ggcagagcta	C→T	tgagaagagg	19.16	1.73	13.87	1.33	4.91	10^{-6}	A			
		rs1333517561:T	tctaccttaa	G→T	atattgtttt	3.06	0.38	2.37	0.22	3.26	10^{-2}	C			
		rs1355133691:G	atcttttata	A→G	tgaggtataa	3.06	0.38	2.33	0.26	3.22	10^{-2}	C			
		rs1398263987:A	ccgccatgat	T→A	ctgaggcctt	27.96	1.83	13.60	1.61	10.63	10^{-6}	A			
		rs1443711872:T	tggggagcc	C→T	aataaatgtg	19.16	1.73	8.89	1.41	8.43	10^{-6}	A			
		rs1958507999:A	ttaaggatta	T→A	tggaaaatag	13.66	1.17	8.38	0.64	8.53	10^{-6}	A			
		rs1958512133:T	atgctgctgc	C→T	aatttgtggg	19.50	1.51	17.57	1.15	2.05	0.05	D			
		rs1958512404:T	ccaatttgtg	G→T	gaccgctgct	19.50	1.51	14.25	1.13	5.66	10^{-6}	A			
		rs1958523750:G	gctggcagag	C→G	tactgagaag	19.16	1.73	15.35	1.57	3.26	10^{-2}	C			
		rs1958527057:T	tttgggggag	C→T	ccaataaatg	19.16	1.73	13.39	0.97	6.20	10^{-6}	A			
		rs1960905875:T	ttgcagccgc	C→T	atgattctga	27.96	1.83	16.89	1.46	9.30	10^{-6}	A			
		rs1961713262:A	cttttataat	G→A	aggtataatc	3.06	0.38	2.19	0.24	4.05	10^{-3}	B			
		rs368397887:T	cagagctact	G→T	agaagaggac	19.16	1.73	5.41	0.52	19.23	10^{-6}	A			
		rs372798746:T	tccgcttttg	C→T	tgcttcctca	27.96	1.83	18.15	1.23	9.17	10^{-6}	A			
		rs754138831:A	cgccatgatt	C→A	tgaggccttc	27.96	1.83	14.53	1.05	13.41	10^{-6}	A			
		rs914726851:T	ctgcttcctc	A→T	tttctcttg	27.96	1.83	22.73	1.48	4.49	10^{-3}	B			
		rs951736272:G	tcctcatttt	C→G	tcttgcagcc	27.96	1.83	16.99	1.12	10.72	10^{-6}	A			
30	*COCH* (1690)	rs1011026634:C	cctgtctgtc	G→C	tcgttttggc	41.49	2.72	51.91	4.38	4.19	10^{-3}	B	↓	within human anti-bacterial humoral innate immune response models using Coch-knockout mice [149]: less immune response after infection with different bacteria as susceptibility to bacterial infections that can aggravate POAG according to a biomedical case report [150]	▼
		rs1171679416:G	cggcccagcc	T→G	ttatagcggc	3.38	0.37	5.65	0.78	5.87	10^{-6}	A			
		rs1180655203:G	ggggttgcac	A→G	ccgatcctgg	67.42	6.03	85.6	6.68	4.02	10^{-3}	B			
		rs1220632080:T	ggagctggaa	G→T	tcgccgccgg	66.84	5.42	100.62	7.64	7.36	10^{-6}	A			
		rs1281623170:G	gcgaaggcgg	A→G	gctggaagtc	66.84	5.42	76.93	6.21	2.46	0.05	D			
		rs1324773370:C	ccagccttta	T→C	agcggccgcg	3.38	0.37	11.33	1.34	15.02	10^{-6}	A			
		rs1324773370:G	ccagccttta	T→G	agcggccgcg	3.38	0.37	16.89	1.81	21.04	10^{-6}	A			
		rs1345542947:A	gagctggaag	T→A	cgccgccggg	66.84	5.42	78.95	6.24	2.94	10^{-2}	C			
		rs1390836479:A	gtctgtcgtc	G→A	ttttggcgcc	41.49	2.72	45.50	2.60	2.12	0.05	D			
		rs1895286407:A	tcgctcccag	C→A	ctgtctgtcg	41.49	2.72	49.16	3.86	3.31	10^{-3}	B			
		rs1895286698:G	gctcccagcc	T→G	gtctgtcgtc	41.49	2.72	47.00	3.45	2.53	0.05	D			
		rs1895287203:G	tctgtcgtcg	T→G	tttggcgccc	41.49	2.72	47.44	3.71	2.62	10^{-2}	C			
		rs1895537347:G	gagactgcta	A→G	tgaggggact	20.57	1.50	23.93	1.72	2.96	10^{-2}	C			
		rs1895537467:C	agactgctaa	T→C	gaggggactg	20.57	1.50	23.63	1.83	2.61	10^{-2}	C			
		rs528387564:C	ctctgaggag	G→C	tgacgcgcgg	82.86	6.27	108.00	9.37	4.60	10^{-3}	B			
		rs577009320:G	tgagactgct	A→G	atgaggggac	20.57	1.50	32.69	2.55	8.68	10^{-6}	A			
		rs748500996:A	ctaatgaggg	G→A	actggtttgg	20.57	1.50	23.04	1.68	2.21	0.05	D			
		rs751651997:G	ctcttgagac	T→G	gctaatgagg	20.57	1.50	32.06	2.49	8.33	10^{-6}	A			
		rs941492932:C	ggtgcggggt	T→C	gcacaccgat	67.42	6.03	85.60	6.68	4.02	10^{-3}	B			

Table S3. Cont.

#	Human Gene: NCBI Gene Symbol (NCBI Gene ID)	Candidate SNP marker: dbSNP ID:min [437]	5′ flank, 10 bp	WT →min	3 flank, 10 bp	K_D, nM, *in silico*: WT MEAN	± SEM	min MEAN	± SEM	Significance: Z	p	ϱ	Δ	Effect of changes in human gene expression on the development of primary open-angle glaucoma (POAG, ☼: "▼" aggravation, "▲" alleviation) [Reference]	☼ ▲▼
30	*COCH* (1690)	rs1016133322:A	ctcgatttgc	C→A	gccgaggcgc	67.42	6.03	42.38	3.26	7.87	10^{-6}	A	↑	according to a comprehensive review of human disease models using young DBA/2J mice susceptible to POAG [151]: Coch-excess may be a biomedical molecular marker for elevated intraocular pressure, retinal ganglion cell degeneration, and optic nerve damage in older ages	▼
		rs1052186777:T	ctctcctctg	C→T	gccgcgcccg	82.86	6.27	66.36	4.86	4.22	10^{-3}	B			
		rs1225527080:T	cctttatagc	G→T	gccgcggggg	3.38	0.37	2.70	0.27	3.02	10^{-2}	C			
		rs1307781228:T	cgctcccagc	C→T	tgtctgtcgt	41.49	2.72	30.59	2.12	6.38	10^{-6}	A			
		rs1323866782:T	tcccgcgctt	C→T	ctcccgcgcc	66.84	5.42	53.60	3.99	4.01	10^{-3}	B			
		rs1447321198:T	gtcctctctc	C→T	tctgcgccgc	82.86	6.27	49.21	3.55	9.98	10^{-6}	A			
		rs1555310259:A	agctggaagt	C→A	gccgccgggc	66.84	5.42	33.69	2.85	11.70	10^{-6}	A			
		rs1566404136:T	ggcggagctg	G→T	aagtcgccgc	66.84	5.42	9.16	0.9	31.20	10^{-6}	A			
		rs1895286792:A	ctcccagcct	G→A	tctgtcgtcg	41.49	2.72	16.93	1.25	18.17	10^{-6}	A			
		rs1895286993:T	tgtctgtcgt	C→T	gttttggcgc	41.49	2.72	34.23	2.47	3.95	10^{-3}	B			
		rs1895287305:T	gtcgttttgg	C→T	gcccccgcct	41.49	2.72	36.68	2.38	2.67	10^{-2}	C			
		rs1895288901:A	gttgcacacc	G→A	atcctgggct	67.42	6.03	43.56	3.65	7.13	10^{-6}	A			
		rs1895293794:T	cccggggatc	C→T	gaagggtgcg	82.86	6.27	49.26	3.60	9.89	10^{-6}	A			
		rs1895538611:T	ggactggttt	G→T	gttgttcgca	20.57	1.50	17.36	1.13	3.47	10^{-3}	B			
		rs755127637:A	gactgctaat	G→A	aggggactgg	20.57	1.50	9.23	1.10	11.49	10^{-6}	A			
		rs771415523:T	ctcctctgcg	C→T	cgcgcccggg	82.86	6.27	74.87	5.16	1.98	0.05	D			
		rs771771730:T	gggtgcgggg	C→T	tctgaggagg	82.86	6.27	70.41	5.02	3.13	10^{-2}	C			
		rs775266701:G	gtgcggggct	C→G	tgaggaggtg	82.86	6.27	72.81	5.56	2.41	0.05	D			
		rs866110012:A	aggcggagct	G→A	gaagtcgccg	66.84	5.42	14.34	1.34	24.90	10^{-6}	A			
		rs967507373:T	ggcgaaggcg	G→T	agctggaagt	66.84	5.42	37.98	2.86	10.22	10^{-6}	A			
31	*COL1A1* (1277)	rs1276176600:G	aggacagtat	A→G	tgctctccat	1.36	0.14	4.25	0.39	16.39	10^{-6}	A	↓	within human POAG filtration surgery models using mice [152]: auxiliary treatment with mitomycin-C during surgery downregulates Col1a1 and simultaneously prevents fibrosis as post-surgery	▲
		rs553852405:A	caccctgtgg	T→A	cctcccccctc	6.45	0.56	8.81	0.82	4.88	10^{-3}	B			
		rs573537477:A	tcaccctgtg	G→A	ccctcccccct	6.45	0.56	7.51	0.62	2.54	0.05	D			
		rs933521482:G	tgtggttttt	A→G	ccctctcacc	6.45	0.56	18.04	1.21	18.68	10^{-6}	A			
		rs975122507:G	ccatcaggac	A→G	ctccctgctc	1.36	0.14	1.84	0.19	4.11	10^{-3}	B			
32	*COL8A1* (1295)	rs1302666883:C	tcctccgttg	T→C	gaccgcgctc	44.03	3.37	84.68	6.79	11.79	10^{-6}	A	↓	within human disease models using Col8a1-knockout mice [154]: thinning of both corneal stroma and of Descemet's membrane as symptoms of POAG [155]	▼
		rs142663006:G	cccacctact	A→G	tgaggagcag	4.90	0.45	5.63	0.48	2.22	10^{-6}	A			
		rs1576407275:C	ctccgttgtg	A→C	ccgcgctccc	44.03	3.37	83.34	6.42	11.74	10^{-6}	A			
		rs189264986:T	ccataaaagc	C→T	aagtgcatgt	5.63	0.48	6.58	0.55	2.60	10^{-6}	A			
		rs1937435199:C	cactccttcc	T→C	ccgttgtgac	44.03	3.37	49.43	3.96	2.09	10^{-6}	A			
		rs1937435277:C	cttcctccgt	T→C	gtgaccgcgc	44.03	3.37	84.68	6.79	11.79	10^{-6}	A			
		rs1937435320:C	ttcctccgtt	G→C	tgaccgcgct	44.03	3.37	80.87	6.04	11.36	10^{-6}	A			
		rs1937440763:G	tgcccataaa	A→G	gccaagtgca	5.63	0.48	9.02	0.78	7.75	10^{-6}	A			
		rs1937440802:T	gcccataaaa	G→T	ccaagtgcat	5.63	0.48	6.50	0.57	2.33	10^{-6}	A			
		rs573260456:C	ccacctacta	T→C	gaggagcagc	4.90	0.45	5.63	0.48	2.22	10^{-6}	A			
		rs970152339:A	gcccacctac	T→A	atgaggagca	4.90	0.45	5.63	0.48	2.22	10^{-6}	A			
		rs1007998668:T	tccttcctcc	G→T	ttgtgaccgc	44.03	3.37	30.89	2.38	6.53	10^{-6}	A	↑	within a biomedical cohort-based transcriptome study of microneedling therapy [156]: COL8A1 upregulation is a biomedical molecular marker for wound healing, which is the major problem of glaucoma surgery according to a comprehensive retrospective review [157]	▲
		rs1226165956:A	cctccgcctt	G→A	cgggctccag	44.03	3.37	37.81	3.82	2.40	10^{-6}	A			
		rs1402575524:A	tcccacctgc	C→A	cataaaagcc	4.90	0.45	4.08	0.36	2.88	10^{-6}	A			
		rs1413286706:A	ttgcgggctc	C→A	agtctctgcc	44.03	3.37	39.30	2.93	2.13	10^{-6}	A			
		rs1413286706:T	ttgcgggctc	C→T	agtctctgcc	44.03	3.37	19.37	1.73	13.94	10^{-6}	A			
		rs1937436822:A	cccgccccgt	C→A	gtctctcctc	44.03	3.37	30.00	2.61	6.62	10^{-6}	A			
		rs1937436982:A	cgtctctcct	C→A	cgccttgcgg	44.03	3.37	35.69	3.23	3.54	10^{-6}	A			
		rs1937440314:A	ctactatgag	G→A	agcagctcct	4.90	0.45	3.45	0.37	4.94	10^{-6}	A			
		rs755916456:A	cacctactat	G→A	aggagcagct	4.90	0.45	1.78	0.19	14.31	10^{-6}	A			
		rs916013768:A	tccctgccca	C→A	ctactatgag	4.90	0.45	3.87	0.40	3.38	10^{-6}	A			

Table S3. Cont.

#	Human Gene: *NCBI Gene Symbol* (NCBI Gene ID)	Candidate SNP marker: dbSNP ID:min [437]	5′ flank, 10 bp	WT →min	3 flank, 10 bp	K_D, nM, *in silico*: WT MEAN	± SEM	min MEAN	± SEM	Significance: Z	p	ϱ	Δ	Effect of changes in human gene expression on the development of primary open-angle glaucoma (POAG, ☼: "▼" aggravation, "▲" alleviation) [Reference]	☼ ▲ ▼
33	*COL8A2* (1296)	rs1159765578:C	gctgtggaaa	G→C	ccttggccgt	40.86	3.35	51.15	4.06	3.94	10^{-3}	B	↓	within human POAG models using Col8a2-defective mutant mice [158]: retinal ganglion cell damage resistance, which can alleviate POAG	▲
		rs1441636841:A	tgtggaaaga	G→A	ttggccgtgg	40.86	3.35	48.44	3.48	3.12	10^{-2}	C			
		rs1643944724:A	gaaagaggag	G→A	ccgtggctgt	40.86	3.35	45.77	3.74	1.96	0.05	D			
		rs1643944776:C	tggctgtgga	A→C	ggccttggcc	40.86	3.35	48.61	3.86	3.05	10^{-2}	C			
		rs1257216977:T	gaccccgggc	C→T	gatcttttgg	32.47	2.17	16.40	1.41	12.52	10^{-6}	A	↑	within both biomedical cohort-based transcriptome study of post-surgical corneal wound healing in glaucoma patients and biomedical glaucoma surgery models using mice [159]: COL8A2 upregulation is one among three most vital gene expression changes during the very first week of wound healing immediately after glaucoma surgery until its fibrotic complication may begin that can require auxiliary therapy suppressing collagen-encoding genes	
		rs1278108123:G	tttctctgtt	C→G	ggcttgatga	32.47	2.17	27.87	1.77	3.32	10^{-3}	B			
		rs1278108123:T	tttctctgtt	C→T	ggcttgatga	32.47	2.17	24.45	1.62	6.02	10^{-6}	A			
		rs1336518767:A	gttcttcttt	G→A	tgactttctc	32.47	2.17	8.87	0.88	21.68	10^{-6}	A			
		rs1570031394:G	gcttgatgac	T→G	acccagggct	32.47	2.17	27.39	1.82	3.61	10^{-3}	B			
		rs1643658365:A	ctggcttgat	G→A	accacccagg	32.47	2.17	11.64	0.84	20.85	10^{-6}	A			
		rs1643658422:T	ccagggctgg	C→T	tgccaaacca	32.47	2.17	25.79	1.87	4.66	10^{-3}	B			
		rs1643659426:T	ggcctagtgc	C→T	tggtgacccc	32.47	2.17	19.10	1.66	9.67	10^{-6}	A			
		rs1643659464:T	gggcctagtg	C→T	ttggtgaccc	32.47	2.17	16.43	1.51	11.98	10^{-6}	A			
		rs1643944811:A	ccttggccgt	G→A	cggggcgcgg	40.86	3.35	24.47	1.79	9.34	10^{-6}	A			
		rs562985985:A	aaattctgat	C→A	gtgtaatctc	32.47	2.17	16.12	1.19	14.04	10^{-6}	A			
		rs766700848:T	ttctttgcag	C→T	tttctctgtt	32.47	2.17	21.50	1.76	7.79	10^{-6}	A			
		rs917813569:T	cccagggctg	G→T	gtgccaaacc	32.47	2.17	25.76	1.89	4.66	10^{-3}	B			
34	*CP* (1356)	rs1212304646:C	ctcacttacg	T→C	taggtgaaac	4.37	0.52	7.51	0.64	7.38	10^{-6}	A	↓	within a biomedical cohort comparison study of patients with cataracts with and without POAG [160]: low serum ceruloplasmin levels are molecular markers of POAG risk in cataract	▼
		rs1559957827:C	gctaaaatag	T→C	agtttagtct	3.71	0.35	4.67	0.40	3.61	10^{-3}	B			
		rs554237003:T	aaaatagtaa	C→T	ttagtcttgc	3.71	0.35	4.29	0.40	2.21	0.05	D			
		rs571401035:A	aagaaagtct	T→A	agcaaataag	1.72	0.17	2.45	0.25	4.96	10^{-6}	A			
		rs757258903:A	tgctaaaata	G→A	aagtttagtc	3.71	0.35	4.67	0.40	3.61	10^{-3}	B			
		rs1284740324:A	cttacgtatg	G→A	tgaaactctc	4.37	0.52	2.61	0.30	6.24	10^{-6}	A	↑	within nonhuman primate POAG models using male juvenile cynomolgus monkeys (*Macaca fascicularis*) treated with an argon laser to scar the trabecular meshwork in one eye until an intraocular pressure increased indicating glaucomatous eye [161]: ceruloplasmin excess in the retina that may be needed as part of antioxidant system, which must counteract the inevitable damaging effects of free radicals on the head of the optic nerve when perceiving light flux as a vital function of the eyes	▲
		rs1338118501:A	acttacgtat	G→A	gtgaaactct	4.37	0.52	2.24	0.27	7.88	10^{-6}	A			
		rs1373417354:G	cttatttata	A→G	aggaagaaag	1.72	0.17	1.43	0.14	2.66	10^{-2}	C			
		rs151304828:T	ctctcactta	C→T	tttaggtgaa	4.37	0.52	2.69	0.27	6.22	10^{-6}	A			
		rs1727958716:C	agattctcta	G→C	actttaactc	3.71	0.35	2.84	0.26	4.03	10^{-3}	B			
		rs566097592:A	tctcacttac	G→A	ttaggtgaaa	4.37	0.52	2.81	0.32	5.36	10^{-6}	A			
35	*CPAMD8* (27151)	rs1224346602:C	ccggggcctt	T→C	cttctgtggc	8.80	0.84	33.32	2.80	20.96	10^{-6}	A	↓	within human disease models using zebrafish embryos carrying the CRISPR/Cas9-disruped Cpamd8 gene [162]: anterior segment dysgenesis as microphthalmia, which is comorbid with POAG [163]	▼
		rs1279924346:A	ccccggggcc	T→A	ggcttctgtg	8.80	0.84	14.26	2.14	5.44	10^{-6}	A			
		rs1334327230:G	gtcccggaga	C→G	cgagcggggc	100.13	7.38	151.64	11.36	7.90	10^{-6}	A			
		rs1433747250:C	tcccggagac	G→C	gagcggggcg	100.13	7.38	144.77	11.28	6.88	10^{-6}	A			
		rs1434332277:C	ctcctttggc	T→C	aagtggactg	40.79	3.55	48.93	4.30	2.94	10^{-2}	C			
		rs1599948931:G	cccggagacg	A→G	agcggggcgg	100.13	7.38	164.87	13.19	9.17	10^{-6}	A			
		rs1599948979:G	gcggtcccgg	A→G	gggcgagcgg	100.13	7.38	165.69	12.79	9.44	10^{-6}	A			
		rs2051979579:T	cggggccttt	A→T	ttctgtggcc	8.80	0.84	34.96	2.39	23.51	10^{-6}	A			
		rs2052166971:C	ggaagcaagt	A→C	gggatgggag	21.44	2.05	41.00	3.13	10.60	10^{-6}	A			
		rs759848196:A	gtggccccgg	G→A	acctggcttc	8.80	0.84	10.52	1.10	2.53	0.05	D			
		rs764656970:G	gcaagtagag	A→G	tgggagagga	21.44	2.05	25.94	2.15	3.01	10^{-2}	C			
		rs771405368:C	gggcctttac	A→C	ctgtggcccc	8.80	0.84	17.77	1.67	10.51	10^{-6}	A			
		rs771405368:T	gggcctttac	A→T	ctgtggcccc	8.80	0.84	12.61	1.45	4.83	10^{-3}	B			
		rs774879992:C	aggaagcaag	T→C	ggggatggga	21.44	2.05	41.00	3.13	10.60	10^{-6}	A			

Table S3. Cont.

Human Gene		Candidate SNP marker				K_D, nM, *in silico*				Significance				Effect of changes in human gene expression on the development of primary open-angle glaucoma (POAG, ☼: "▼" aggravation, "▲" alleviation) [Reference]	☼
#	*NCBI Gene Symbol* (NCBI Gene ID)	dbSNP ID:min [437]	5′ flank, 10 bp	WT →min	3 flank, 10 bp	WT MEAN	± SEM	min MEAN	± SEM	Z	p	ϱ	Δ		▲ ▼
		rs1198660139:T	ggggccttta	C→T	tctgtggccc	8.80	0.84	2.62	0.26	17.42	10^{-6}	A			
		rs1217638498:T	aggacggccg	C→T	gcggcggcgc	288.79	22.46	248.29	19.39	2.74	10^{-2}	C			
		rs1270350764:A	gcgcgaggac	G→A	ccccggcggc	288.79	22.46	251.02	20.39	2.49	0.05	D			
		rs1299008978:T	gaggaagcaa	G→T	aggggatggg	21.44	2.05	18.47	1.45	2.41	0.05	D			
		rs1308452173:A	ggaccctggc	C→A	cggccgcgct	288.79	22.46	208.51	17.19	5.75	10^{-6}	A			
		rs1312765862:A	cctggccctg	G→A	cgcgctcgga	288.79	22.46	166.15	13.63	9.78	10^{-6}	A			
		rs1332974558:C	agcaagtaga	G→C	atgggagagg	21.44	2.05	16.13	1.84	3.83	10^{-3}	B			
		rs1339331904:T	ccagccctgg	C→T	ctggccctgg	288.79	22.46	189.32	14.99	7.61	10^{-6}	A			
		rs1372308239:A	tttggcttag	G→A	ggactgtctc	40.79	3.55	17.29	1.50	13.96	10^{-6}	A			
		rs1373324158:T	cggccgcgct	C→T	cggcgcgagg	288.79	22.46	135.70	11.97	12.85	10^{-6}	A			
		rs1377238720:A	gaagcaagta	G→A	ggatgggaga	21.44	2.05	16.80	1.32	3.94	10^{-3}	B			
		rs1377327305:A	ggcttagggt	G→A	ctgtctcctt	40.79	3.55	25.86	2.64	6.80	10^{-6}	A			
		rs1433747250:A	tcccggagac	G→A	gagcggggcg	100.13	7.38	54.10	4.08	11.69	10^{-6}	A			
		rs1433747250:T	tcccggagac	G→T	gagcggggcg	100.13	7.38	52.32	4.51	11.45	10^{-6}	A			
		rs1443292142:T	cctccccagg	C→T	tggcccggcc	288.79	22.46	136.21	10.71	13.59	10^{-6}	A			
		rs1452314526:T	ccccaggagc	C→T	ggcgcggcgc	288.79	22.46	227.76	17.86	4.30	10^{-3}	B			
		rs1460928662:T	cgcccaggag	C→T	ccgggagggt	100.13	7.38	84.16	6.75	3.19	10^{-2}	C			
35	*CPAMD8* (27151)	rs1599687513:A	ggtgaggcct	G→A	ctttggctta	40.79	3.55	15.87	1.55	14.43	10^{-6}	A	↑	within human disease cellular models using both human glioblastoma U251 and rhabdomyosarcoma RD cell lines [164]: CPAMD8 excess can contribute to innate immune response, which can in turn improve retinal ganglion cell survival after injury cause by an increase in intraocular pressure according to comprehensive review [165]	▲
		rs1599948170:T	gcccggcccc	C→T	gcccagccct	288.79	22.46	257.06	21.18	2.05	0.05	D			
		rs2052861492:A	gtctcctttg	G→A	ggaagtggac	40.79	3.55	23.26	1.77	9.71	10^{-6}	A			
		rs2052861492:T	gtctcctttg	G→T	ggaagtggac	40.79	3.55	17.47	1.49	13.89	10^{-6}	A			
		rs2057092344:A	cccctcccca	G→A	cctggcccgg	288.79	22.46	215.28	17.33	5.25	10^{-6}	A			
		rs2057092638:A	ccggcccccct	C→A	ccagccctgg	288.79	22.46	76.74	9.83	17.69	10^{-6}	A			
		rs2057094191:A	tcggaccctg	G→A	gacggccgcg	288.79	22.46	171.70	13.01	9.58	10^{-6}	A			
		rs2057094378:T	cgctcggacc	C→T	gaggacggcc	288.79	22.46	188.15	12.82	8.29	10^{-6}	A			
		rs2057094931:A	ggacggccgc	G→A	cggcggcgcg	288.79	22.46	104.77	8.64	17.89	10^{-6}	A			
		rs2057102916:A	cggagacgag	G→A	cggggcggtc	100.13	7.38	77.56	5.75	4.89	10^{-3}	B			
		rs2057103014:A	ccggagacga	G→A	gcggggcggt	100.13	7.38	74.19	5.74	5.61	10^{-6}	A			
		rs2057103629:T	ggggcggtcc	C→T	gcggggcgag	100.13	7.38	84.19	6.25	3.32	10^{-3}	B			
		rs45485597:T	agtccccttg	C→T	tgaggcctgt	40.79	3.55	17.99	1.98	11.65	10^{-6}	A			
		rs576043929:A	ggccgcgctc	G→A	ggcgcgagga	288.79	22.46	99.37	8.05	19.00	10^{-6}	A			
		rs749944979:T	tgtctccttt	G→T	gggaagtgga	40.79	3.55	35.70	2.72	2.30	0.05	D			
		rs767178412:A	ttggcttagg	G→A	gactgtctcc	40.79	3.55	34.33	2.43	3.08	10^{-2}	C			
		rs928016376:T	ctggccctgg	C→T	gcgctcggac	288.79	22.46	144.50	12.07	12.13	10^{-6}	A			
		rs960961995:T	gccgcgctcg	G→T	gcgcgaggac	288.79	22.46	36.23	3.72	32.22	10^{-6}	A			
		rs1188510691:T	ggggaaagaa	C→T	gcagaggtca	14.06	0.93	15.75	0.98	2.50	0.05	D			
		rs1197290591:C	cacaaaaccc	A→C	ccccctctta	18.66	2.13	22.29	2.53	2.21	0.05	D			
		rs1197290591:G	cacaaaaccc	A→G	ccccctctta	18.66	2.13	26.29	2.78	4.41	10^{-3}	B			
		rs1276599004:A	atccccctct	T→A	agggactgaa	10.11	0.88	12.64	0.96	3.87	10^{-3}	B			
		rs1276599004:C	atccccctct	T→C	agggactgaa	10.11	0.88	12.81	0.98	4.08	10^{-3}	B			
36	*CPNE1* (8904)	rs1342578153:C	ctcttagcac	A→C	tgaatatccc	10.11	0.88	18.66	2.13	8.55	10^{-6}	A	↓	within human disease cellular models using siRNA-based CPNE1-knockdown [166]: inhibited tumor growth and promoted cell apoptosis in breast cancer, a complication of which is tamoxifen-associated maculopathy in POAG [167]	▲
		rs1342578153:G	ctcttagcac	A→G	tgaatatccc	10.11	0.88	18.66	2.13	8.55	10^{-6}	A			
		rs1410043143:T	actaaatcca	G→T	agggaaatga	4.93	0.50	5.99	0.62	2.70	10^{-2}	C			
		rs1601508483:C	cttgacgtca	A→C	ttgcgcggcc	41.23	2.79	46.82	3.37	2.57	0.05	D			
		rs1601508580:C	ttagcacaaa	A→C	atatccccct	10.11	0.88	18.66	2.52	7.64	10^{-6}	A			

Table S3. Cont.

Human Gene		Candidate SNP marker				K_D, nM, *in silico*				Significance				Effect of changes in human gene expression on the development of primary open-angle glaucoma (POAG, ☼: "▼" aggravation, "▲" alleviation) [Reference]	☼ ▲ ▼
#	*NCBI Gene Symbol* (NCBI Gene ID)	dbSNP ID:min [437]	5′ flank, 10 bp	WT →min	3 flank, 10 bp	WT MEAN	± SEM	min MEAN	± SEM	Z	p	ϱ	Δ		
36	*CPNE1* (8904)	rs2032051334:A	attttcattc	T→A	gaaagaacct	14.06	0.93	16.18	1.13	2.91	10^{-2}	C			▲
		rs2032051571:C	cctcattttc	A→C	tggggaaaga	14.06	0.93	26.06	1.93	12.43	10^{-6}	A			
		rs2034461773:C	cgacggccat	T→C	gctgagcggg	16.63	1.27	30.43	2.41	11.00	10^{-6}	A			
		rs2034467782:C	cgcggcccct	T→C	cagcgcgctt	41.23	2.79	49.09	3.67	3.46	10^{-3}	B			
		rs2034467927:C	gcgcggcccc	T→C	tcagcgcgct	41.23	2.79	49.09	3.67	3.46	10^{-3}	B			
		rs2034469701:C	caaaacccag	T→C	ccctcttagc	18.66	2.13	41.23	2.79	11.98	10^{-6}	A			
		rs2034470518:C	ccctcttagc	A→C	actgaatatc	10.11	0.88	18.66	2.13	8.55	10^{-6}	A			
		rs2034470518:G	ccctcttagc	A→G	actgaatatc	10.11	0.88	18.66	2.13	8.55	10^{-6}	A			
		rs2034470655:G	tccccctctt	A→G	gggactgaat	10.11	0.88	13.49	1.03	4.98	10^{-6}	A	↓	within human disease cellular models using siRNA-based CPNE1-knockdown [166]: inhibited tumor growth and promoted cell apoptosis in breast cancer, a complication of which is tamoxifen-associated maculopathy in POAG [167]	
		rs555080174:C	cggccccttg	A→C	gcgcgcttgc	41.23	2.79	49.09	3.67	3.46	10^{-3}	B			
		rs555080174:G	cggccccttg	A→G	gcgcgcttgc	41.23	2.79	49.09	3.67	3.46	10^{-3}	B			
		rs573649329:G	tgcgcggccc	C→G	gtcagcgcgc	41.23	2.79	49.09	3.67	3.46	10^{-3}	B			
		rs751753039:C	acggccattt	T→C	tgagcgggcc	16.63	1.27	35.74	2.36	15.18	10^{-6}	A			
		rs769708366:G	ggaaatgata	C→G	tatgaccaga	4.93	0.50	5.93	0.54	2.71	10^{-2}	C			
		rs778007118:T	gggaaatgat	A→T	ctatgaccag	4.93	0.50	14.41	1.41	15.23	10^{-6}	A			
		rs958027236:T	agcgggccga	C→T	cggagccgct	16.63	1.27	18.63	1.43	2.10	0.05	D			
		rs960966945:C	ccccttgacg	T→C	cgcttgcgcg	41.23	2.79	49.09	3.67	3.46	10^{-6}	A			
		rs1025243919:T	ttttgtgaag	C→T	gccgacggcc	16.63	1.27	14.71	1.14	2.25	0.05	D			
		rs1164053864:A	ggagtgggat	G→A	gggctgcagg	24.95	2.37	15.67	1.29	7.41	10^{-6}	A			
		rs1165571608:A	ttgacgtcaa	C→A	tgcgcggccc	41.23	2.79	31.66	2.40	5.21	10^{-6}	A			
		rs1180580510:T	ccagagggaa	A→T	ttcagctatg	4.93	0.5	3.93	0.45	2.96	10^{-2}	C			
		rs1183902178:T	agtgggatgc	C→T	gctgcagggg	24.95	2.37	8.17	0.85	15.81	10^{-6}	A			
		rs1197290591:T	cacaaaaccc	A→T	cccccctctta	18.66	2.13	13.02	1.27	4.80	10^{-3}	B			
		rs1218975287:A	gccccttgac	G→A	gcgcttgcgc	41.23	2.79	26.98	2.02	8.41	10^{-6}	A			
		rs1237724361:T	gttcctcccc	C→T	tcaaccacgc	41.23	2.79	30.55	2.18	6.10	10^{-6}	A			
		rs1240237540:A	tgccacttag	G→A	ggggagtggg	24.95	2.37	10.94	1.19	11.40	10^{-6}	A			
		rs1337934428:T	gccgacggcc	A→T	ccgctgagcg	16.63	1.27	13.52	0.98	3.94	10^{-3}	B			
		rs1345389207:T	ccacttaggc	C→T	ggagtgggat	24.95	2.37	20.13	2.09	3.05	10^{-2}	C			
		rs1398436609:A	atgccactta	G→A	gggggagtgg	24.95	2.37	10.60	1.06	12.38	10^{-6}	A			
		rs1446258117:T	tgggatgcca	C→T	tgcaggggga	24.95	2.37	18.00	1.57	5.07	10^{-6}	A			
		rs1864606873:T	ggccgacggc	C→T	gccgctgagc	41.23	2.79	20.82	1.44	14.12	10^{-6}	A			
		rs2034432464:T	tgagaggatg	C→T	cctctgagat	24.95	2.37	10.36	0.93	13.46	10^{-6}	A	↑	within human disease cellular models using human nasopharyngeal carcinoma cell lines NPC under different radiations up to 8 Gy [168]: CPNE1 excess along with improved radioresistance that can prevent glaucomagenesis following ionizing radiation exposure according to comprehensive biomedical review [169]	
		rs2034461523:A	cggccatttt	G→A	gagcgggccg	16.63	1.27	5.95	0.60	16.25	10^{-6}	A			
		rs2034462019:T	gggccgacgg	C→T	agccgctgag	41.23	2.79	36.50	2.40	2.59	10^{-2}	C			
		rs2034466661:T	ccttgacgtc	A→T	cttgcgcggc	41.23	2.79	25.50	2.00	9.28	10^{-6}	A			
		rs2034467305:T	ggccccttga	C→T	cgcgcttgcg	41.23	2.79	19.42	1.40	15.26	10^{-6}	A			
		rs2295356:G	cctccccctt	C→G	accacgccgt	41.23	2.79	34.10	2.85	3.54	10^{-3}	B			
		rs555080174:T	cggccccttg	A→T	gcgcgcttgc	41.23	2.79	32.42	2.47	4.72	10^{-3}	B			
		rs745378738:A	ggtctcgcat	G→A	tcttgtctcc	14.06	0.93	6.75	0.64	12.69	10^{-6}	A			
		rs745378738:T	ggtctcgcat	G→T	tcttgtctcc	14.06	0.93	10.21	0.98	5.47	10^{-6}	A			
		rs886228806:T	cgttcctccc	C→T	gtcaaccacg	41.23	2.79	32.55	2.35	4.78	10^{-3}	B			
		rs930190615:A	ccccccttctg	G→A	acgccgttcc	41.23	2.79	31.13	2.43	5.44	10^{-6}	A			
		rs973227037:T	acctcatttt	C→T	atggggaaag	14.06	0.93	4.15	0.34	23.05	10^{-6}	A			
		rs978567579:A	gccattttgt	G→A	gcgggccgac	16.63	1.27	6.13	0.73	14.17	10^{-6}	A			
		rs984689510:C	ggggagtggg	A→C	cagggctgca	24.95	2.37	17.24	1.98	4.96	10^{-6}	A			
		rs984689510:G	ggggagtggg	A→G	cagggctgca	24.95	2.37	19.14	2.00	3.76	10^{-3}	B			

Table S3. Cont.

Human Gene		Candidate SNP marker				K_D, nM, *in silico*				Significance				Effect of changes in human gene expression on the development of primary open-angle glaucoma (POAG, ☼: "▼" aggravation, "▲" alleviation) [Reference]	☼ ▲ ▼
#	*NCBI Gene Symbol* (NCBI Gene ID)	dbSNP ID:min [437]	5′ flank, 10 bp	WT →min	3 flank, 10 bp	WT MEAN	WT ± SEM	min MEAN	min ± SEM	Z	p	ϱ	Δ		
37	*CXCR3* (2833)	rs2040873500:C	ctggtagagg	T→C	tttagggagg	21.39	2.60	35.47	3.05	6.79	10^{-6}	A	↓	within human disease models using mice subjected with artificial traumatic optic neuropathy [170]: CXCR3-antagonist medication reduced retinal inflammation that may relieve POAG	▲
		rs1004086764:A	gggaggtctg	G→A	cctgaggttt	21.39	2.60	13.79	1.60	5.22	10^{-6}	A	↑	within a biomedical cohort-based study [171]: CXCR3 excess in peripheral blood lymphocytes is a biomedical marker for accelerated progression of the earlier stages of POAG	▼
		rs2040873192:T	cttcctctgt	G→T	tgaaactccc	21.39	2.60	13.90	1.63	5.10	10^{-6}	A			
38	*CYP1A1* (1543)	rs1039570166:G	ccgcctataa	A→G	cacgtacaag	3.55	0.42	6.74	0.83	7.44	10^{-6}	A	↓	within human disease models using senescence-accelerated OXYS rats as a commonly accepted animal model of human age-related macular degeneration compared with Wistar rats as a norm [172]: Cyp1a1-deficit in the retina may indicate the presence of oxidative stress contributing both retinopathy and age-related macular degeneration, which are both comorbid with POAG, at least as age-related diseases [96]	▼
		rs1350087703:G	ttctctccca	A→G	tggcttgccc	6.65	0.58	10.43	1.02	6.89	10^{-6}	A			
		rs2063208870:G	aagcccgcct	A→G	gccacacgta	3.55	0.42	8.61	1.26	9.34	10^{-6}	A			
		rs900568803:G	gcccgcctat	A→G	cacacgtaca	3.55	0.42	8.61	1.26	9.34	10^{-6}	A			
		rs974842698:A	tctctcccaa	T→A	ggcttgccct	6.65	0.58	20.22	1.29	20.62	10^{-6}	A			
		rs1178010409:A	gtacaagccc	G→A	cgtggccaca	3.55	0.42	2.90	0.31	2.52	10^{-6}	A	↑	within models of the effect of smoking on human health using rats [173]: Cyp1a1-upregulation in the iris/ciliary body and retina together with age-related macular degeneration with choroidal neovascularization as the most severe complication	
		rs1596350590:A	cccaataaaa	G→A	gccctttctc	6.65	0.58	5.62	0.50	2.68	10^{-6}	A			
		rs923400379:T	ctttctctcc	C→T	tctggcttgc	6.65	0.58	4.88	0.45	4.85	10^{-6}	A			
39	*CYP1B1* (1545)	rs1023114728:C	tacccagtcc	T→C	tgccctcctt	9.21	0.94	18.09	1.82	9.42	10^{-6}	A	↓	within a biomedical cohort-based study [174]: both reduced catalytic activity and decreased protein stability can elevate risks of POAG	▼
		rs1023114728:G	tacccagtcc	T→G	tgccctcctt	9.21	0.94	11.07	1.13	2.56	0.05	D			
		rs1028331682:C	ctccagttgt	G→C	cgcttctgcg	27.90	2.02	32.73	2.31	3.16	10^{-2}	C			
		rs1400465721:C	cagttgtgag	A→C	ttctgcgact	27.90	2.02	31.33	2.22	2.29	0.05	D			
		rs1682535641:C	gcgactccag	T→C	cgagcgcttc	27.90	2.02	38.71	2.93	6.25	10^{-6}	A			
		rs1682536808:C	cagtccttaa	A→C	ctccttctac	9.21	0.94	14.83	1.27	7.16	10^{-6}	A			
		rs1682536840:C	acccagtcct	T→C	gccctccttc	9.21	0.94	19.18	1.86	10.44	10^{-6}	A			
		rs1683079689:C	acacagctta	T→C	ttattaacca	7.49	0.46	9.13	0.68	4.11	10^{-3}	B			
		rs1683079946:G	aacacagctt	A→G	cttattaacc	7.49	0.46	10.14	1.02	5.16	10^{-6}	A			
		rs1683079971:A	aaacacagct	T→A	gcttattaac	7.49	0.46	9.53	0.79	4.69	10^{-3}	B			
		rs1160839936:A	accaaacaca	G→A	gttgcttatt	7.49	0.46	5.46	0.68	4.59	10^{-3}	B	↑	according to a biomedical radiotherapy review [169]: ultraviolet radiation can upregulate CYP1B1 that may often manifest in glaucomagenesis as side effect following ionizing radiotherapy	
		rs1443337898:A	gactccagtt	G→A	agcgcttctg	27.90	2.02	9.67	1.04	16.31	10^{-6}	A			
		rs1573297353:T	taaccaaaca	C→T	atgttgctta	7.49	0.46	6.15	0.75	2.92	10^{-2}	C			
		rs1683079628:C	ttattaatca	T→C	ccaaacacag	7.49	0.46	4.92	0.51	6.97	10^{-6}	A			
		rs1683079628:G	ttattaatca	T→G	ccaaacacag	7.49	0.46	4.58	0.52	7.61	10^{-6}	A			
		rs1683079647:C	cttattaatc	A→C	accaaacaca	7.49	0.46	6.42	0.43	3.42	10^{-3}	B			
		rs1683079664:T	acagcttatt	A→T	attaaccaaa	7.49	0.46	4.95	0.42	7.92	10^{-6}	A			
		rs1683080079:A	tgcttattaa	C→A	gagctgaatg	7.49	0.46	6.07	0.37	4.89	10^{-3}	B			
		rs990198177:A	ccagttgtga	G→A	cttctgcgac	27.90	2.02	21.32	1.58	5.19	10^{-6}	A			
		rs996067465:T	gcttattaac	C→T	agctgaatgt	7.49	0.46	4.62	0.29	11.07	10^{-6}	A			
40	*CYP2C19* (1557)	rs1414185783:G	gagtgttata	A→G	aaagcttgga	2.02	0.21	3.45	0.4	6.93	10^{-6}	A	↓	within a biomedical cohort study [175]: reduced efficiency of CYP2C19 as a metabolizer may increase risks of side effects of timolol as an anti-POAG drug	▼
		rs1848185282:G	tagcatggag	T→G	gttataaaaa	2.02	0.21	2.41	0.24	2.42	0.05	D			
		rs1848185341:C	tggagtgtta	T→C	aaaaagcttg	2.02	0.21	5.13	0.49	13.42	10^{-6}	A			
		rs1848185312:A	agcatggagt	G→A	ttataaaaag	2.02	0.21	1.47	0.14	4.58	10^{-3}	B	↑	within a biomedical cohort study [175]: increased efficiency of CYP2C19 as a metabolizer may reduce risks of side effects of timolol as an anti-POAG drug	▲
41	*CYP46A1* (10858)	rs1350698478:T	ttgtttagtt	C→T	ctgccaggga	10.98	0.88	15.04	1.13	5.74	10^{-6}	A	↓	within human POAG models using rats [176]: voriconazole, as CYP46A1-inhibitor, plays a retinotoxic role under glaucomatous conditions	▼
		rs1402540378:T	aggcggagac	G→T	tggttggcgg	54.24	4.75	78.63	5.57	6.59	10^{-6}	A			
		rs1462581750:G	gctaactatt	C→G	ttagtaacaa	5.39	0.48	6.10	0.51	2.03	0.05	D			
		rs1566823600:C	ctattcttag	T→C	aacaaattac	5.39	0.48	9.66	0.94	8.87	10^{-6}	A			
		rs1595179524:G	ggcggagacg	T→G	ggttggcggg	54.24	4.75	118.21	9.39	13.18	10^{-6}	A			
		rs1595179529:G	gagacgtggt	T→G	ggcggggact	54.24	4.75	73.52	5.41	5.32	10^{-6}	A			
		rs1595201483:G	tggagctgaa	A→G	ctcttgttta	10.98	0.88	13.38	1.22	3.26	10^{-2}	C			
		rs2056474855:A	gggcgctaac	T→A	attcttagta	5.39	0.48	6.39	0.68	2.46	0.05	D			
		rs2056783238:T	agctgaaact	C→T	ttgtttagtt	10.98	0.88	15.02	1.02	5.97	10^{-6}	A			

Table S3. Cont.

#	Human Gene: NCBI Gene Symbol (NCBI Gene ID)	Candidate SNP marker: dbSNP ID:min [437]	5′ flank, 10 bp	WT →min	3 flank, 10 bp	KD, nM, *in silico*: WT MEAN	± SEM	min MEAN	± SEM	Significance: Z	p	ϱ	Δ	Effect of changes in human gene expression on the development of primary open-angle glaucoma (POAG, ☼: "▼" aggravation, "▲" alleviation) [Reference]	☼ ▲▼
41	*CYP46A1* (10858)	rs1269176855:T	gaggcggaga	C→T	gtggttggcg	54.24	4.75	30.75	2.90	8.82	10^{-6}	A	↑	within human POAG models using Cyp46a1-deficient mice [177]: pharmacologic activation of CYP46A1 may be a therapy for dyslipidemia-induced retinal damage and thereby slow the progression of POAG	▲
		rs1413683878:A	tgaaactctt	G→A	tttagttcct	10.98	0.88	5.60	0.56	10.49	10^{-6}	A			
		rs1462581750:T	gctaactatt	C→T	ttagtaacaa	5.39	0.48	3.53	0.30	6.91	10^{-6}	A			
		rs1595179561:T	ggggactgtg	C→T	gccctgggag	54.24	4.75	31.18	2.78	8.86	10^{-6}	A			
		rs2056468893:T	ggaggggtcg	G→T	agtcggcggg	54.24	4.75	32.71	3.24	7.65	10^{-6}	A			
		rs2056474837:C	gaggggcgct	A→C	actattctta	5.39	0.48	4.71	0.37	2.31	0.05	D			
		rs747411166:A	ctcttgttta	G→A	ttcctgccag	10.98	0.88	7.92	0.64	5.73	10^{-6}	A			
		rs915725791:A	cgctaactat	T→A	cttagtaaca	5.39	0.48	3.00	0.32	8.43	10^{-6}	A			
		rs948560295:A	ctaactattc	T→A	tagtaacaaa	5.39	0.48	2.22	0.22	13.39	10^{-6}	A			
42	*DEFB4A* (1673)	rs1472226511:G	caggaccttt	A→G	taaggtggaa	3.48	0.48	9.52	1.05	11.39	10^{-6}	A	↓	within a biomedical cohort-based study [178]: DEFB4A-deficit leads to corneal damage in thyroid eye disease, which is comorbid with POAG among eye diseases that may be aggravated by tobacco smoking [179]	▼
		rs1472226511:T	caggaccttt	A→T	taaggtggaa	3.48	0.48	6.96	0.53	8.79	10^{-6}	A			
		rs1818968330:C	ggacctttat	A→C	aggtggaagg	3.48	0.48	10.17	0.96	12.8	10^{-6}	A			
		rs1818968330:G	ggacctttat	A→G	aggtggaagg	3.48	0.48	8.67	0.89	10.63	10^{-6}	A			
43	*DGCR8* (54487)	rs1199028358:G	tgcggctccc	C→G	caatgcgggg	120.48	9.29	144.18	10.54	3.38	10^{-3}	B	↓	within human age-related macular degeneration models using Dgcr8-deficient mice [181]: higher risks of mature retinal pigmented epithelium cell death and vision loss that is positively genetically correlating POAG according to a biomedical cohort study [182]	▼
		rs1488075923:G	ggctcccca	A→G	tgcggggcgg	120.48	9.29	158.53	10.53	5.39	10^{-6}	A			
		rs2049405171:C	agcccggcct	T→C	tgtgaggcaa	23.93	1.90	56.52	5.34	13.93	10^{-6}	A			
		rs2049405195:G	gcccggcctt	T→G	gtgaggcaac	23.93	1.90	56.73	3.77	16.68	10^{-6}	A			
		rs761877586:G	agctgtctac	A→G	ttaatgaaaa	3.85	0.34	5.32	0.39	5.65	10^{-6}	A			
		rs1017434348:A	cggggattgg	C→A	cgcacgccgc	120.48	9.29	78.8	5.65	8.07	10^{-6}	A	↑	within human disease cellular models using mouse lung epithelial cell line LM2 transfected with lentiviral vector overexpressing Dgcr8 under radiation exposure 8 Gy [183]: DGCR8 excess along with improved radioresistance that can prevent glaucomagenesis following ionizing radiation exposure according to comprehensive biomedical review [169]	▲
		rs1017434348:T	cggggattgg	C→T	cgcacgccgc	120.48	9.29	62.37	4.69	12.23	10^{-6}	A			
		rs1239697693:T	ctgcggctcc	C→T	ccaatgcggg	120.48	9.29	79.88	5.85	7.73	10^{-6}	A			
		rs1263875705:A	tcccccaatg	C→A	ggggcggccg	120.48	9.29	70.27	4.76	10.51	10^{-6}	A			
		rs1263875705:T	tcccccaatg	C→T	ggggcggccg	120.48	9.29	53.77	4.26	14.59	10^{-6}	A			
		rs1415627940:A	ggcggcctcc	G→A	cagcgcgctc	120.48	9.29	100.4	7.68	3.36	10^{-3}	B			
		rs1456407963:T	tctgcggctc	C→T	cccaatgcgg	120.48	9.29	104.94	8.32	2.50	0.05	D			
		rs1459140213:A	cgcacgccgc	G→A	aaggcccgcc	120.48	9.29	82.83	5.37	7.44	10^{-6}	A			
		rs1459140213:T	cgcacgccgc	G→T	aaggcccgcc	120.48	9.29	89.80	6.03	5.75	10^{-6}	A			
		rs2049400221:T	gcggctcccc	C→T	aatgcggggc	120.48	9.29	26.20	2.12	27.31	10^{-6}	A			
		rs2049400350:T	ccaatgcggg	G→T	cggccgcgcg	120.48	9.29	104.37	7.21	2.77	10^{-2}	C			
		rs2049400707:A	ttggccgcac	G→A	ccgcgaaggc	120.48	9.29	94.48	6.24	4.79	10^{-3}	B			
		rs2049400759:T	ccgcacgccg	C→T	gaaggcccgc	120.48	9.29	54.66	3.70	15.40	10^{-6}	A			
		rs533656822:A	ccgcagcgcg	C→A	tctgcggctc	120.48	9.29	105.90	8.02	2.39	0.05	D			
		rs547062594:T	tggccgcacg	C→T	cgcgaaggcc	120.48	9.29	104.96	8.23	2.51	0.05	D			
		rs924072318:T	ggagcccggc	C→T	tttgtgaggc	23.93	1.90	15.19	1.16	8.26	10^{-6}	A			
44	*EDN1* (1906)	rs1348890016:G	ggggttcaat	A→G	taaaaagccg	1.50	0.15	4.73	0.45	16.63	10^{-6}	A	↓	within a biomedical cohort-based study [184]: EDN1-deficit is a risk factor of post-trabeculectomy low intraocular pressure, which is sight threatening because of bleb infection and hypotony maculopathy as postoperative complications in POAG	▼
		rs1762638489:T	aatataaaaa	G→T	ccggcagaga	1.50	0.15	1.76	0.17	2.25	0.05	D			
		rs1762638567:T	atataaaaag	C→T	cggcagagag	1.50	0.15	1.80	0.18	2.51	0.05	D			
45	*EFEMP1* (2202)	rs1259000100:C	tttccctccc	T→C	cgccctcccc	40.43	3.05	75.87	5.77	11.75	10^{-6}	A	↓	within human disease models using Efemp1-knockout mice [186]: corneal dysfunction, which is comorbid to POAG at least within exfoliation syndrome as the most common identifiable cause of POAG in the world [187]	▼
		rs1261756209:C	ttccctccct	T→C	gccctccccc	40.43	3.05	95.50	7.62	15.64	10^{-6}	A			
		rs1463449413:C	ggggtggttt	T→C	agtgttgccc	18.50	1.33	38.90	2.46	15.50	10^{-6}	A			
		rs1052751621:T	ttattgggaa	A→T	ctagcattca	7.35	0.67	6.35	0.52	2.40	0.05	D			
		rs1188366176:T	ccctgaaatg	C→T	gcagtcgcag	18.5	1.33	11.96	1.24	6.89	10^{-6}	A	↑	within a biomedical cohort-based study [188]: fibulin-3 overexpression holds future potential as new promising intraocular pressure lowering therapy for POAG	▲
		rs1208494233:G	ctccctttct	C→G	tccccctttc	40.43	3.05	32.55	2.42	4.09	10^{-3}	B			
		rs1218194697:T	ctttccctcc	C→T	tcgccctccc	40.43	3.05	25.52	1.88	8.72	10^{-6}	A			
		rs1311806771:T	cacttattgg	G→T	ctcctagcat	7.35	0.67	6.35	0.56	2.32	0.05	D			
		rs1671006580:A	tgccggactt	C→A	gccgcggccg	40.43	3.05	29.41	3.56	4.46	10^{-3}	B			
		rs777092611:A	gtggttttgt	G→A	gttgcccggg	18.50	1.33	7.06	0.68	16.02	10^{-6}	A			

Table S3. Cont.

#	Human Gene: *NCBI Gene Symbol* (NCBI Gene ID)	Candidate SNP marker: dbSNP ID:min [437]	5′ flank, 10 bp	WT →min	3 flank, 10 bp	KD, nM, *in silico*: WT MEAN	± SEM	min MEAN	± SEM	Significance: Z	p	ϱ	Δ	Effect of changes in human gene expression on the development of primary open-angle glaucoma (POAG, ☼: "▼" aggravation, "▲" alleviation) [Reference]	☼ ▲ ▼
46	*EGFR* (1956)	rs1562773786:G	tcctgcaata	A→G	tgtctcaggg	10.41	0.92	15.86	1.28	7.06	10^{-6}	A	↓	within a cohort-based biomedical pharmacological study [189]: anti-cancer medications inhibiting EGFR may elevate risks of dry eye, which is comorbid to POAG at least as a side effect of antihypertensive medications in POAG [104]	▼
		rs1786335900:C	cctcctcctc	T→C	gctcctcccg	124.51	9.29	177.85	14.02	6.57	10^{-6}	A			
		rs1786337144:C	cgccgcctgg	T→C	ccctcctcct	177.85	14.02	204.28	16.53	2.45	0.05	D			
		rs1786337304:G	gcctggtccc	T→G	cctcctcccg	177.85	14.02	204.7	15.75	2.55	0.05	D			
		rs1786340276:T	cgagctagac	G→T	tccgggcagc	25.87	2.81	34.42	3.74	3.71	10^{-3}	B			
		rs372961750:A	tgtgttcctg	C→A	aataatgtct	10.41	0.92	13.08	1.00	3.91	10^{-3}	B			
		rs1220736440:T	ctggtccctc	C→T	tcctcccgcc	124.51	9.29	108.79	7.64	2.63	10^{-2}	C	↑	within human disease models using chicken [190]: low molecular weight medication brimonidine treats POAG due to trans-activation of Egrf	▲
		rs1268446807:G	ggtataaact	T→G	ttgactcaca	3.80	0.45	2.87	0.32	3.49	10^{-3}	B			
		rs1317202224:A	tcctccgccg	C→A	ctggtccctc	124.51	9.29	77.87	6.25	8.56	10^{-6}	A			
		rs1392699234:T	cccgatccct	C→T	ctccgccgcc	177.85	14.02	123.70	9.16	6.71	10^{-6}	A			
		rs17288924:A	ccgccgcctg	G→A	tccctcctcc	124.51	9.29	101.27	7.82	3.85	10^{-3}	B			
		rs1785816124:A	ttgtgttcct	G→A	caataatgtc	10.41	0.92	6.47	0.41	8.80	10^{-6}	A			
		rs1786336357:A	cctcccgatc	C→A	ctcctccgcc	124.51	9.29	77.51	6.09	8.75	10^{-6}	A			
		rs1786336944:T	tccgccgcct	G→T	gtccctcctc	124.51	9.29	58.43	4.51	14.10	10^{-6}	A			
		rs1786337606:T	ggtccctcct	C→T	ctcccgccct	124.51	9.29	90.70	6.74	6.01	10^{-6}	A			
		rs1786338071:A	ctcccgccct	G→A	cctccccgcg	124.51	9.29	52.10	4.92	14.48	10^{-6}	A			
		rs1786338153:A	tcccgccctg	C→A	ctccccgcgc	177.85	14.02	122.85	8.82	6.94	10^{-6}	A			
		rs1786340050:C	gcgcgagcta	G→C	acgtccgggc	39.48	3.51	23.99	2.18	7.83	10^{-6}	A			
		rs1786340170:T	gcgagctaga	C→T	gtccgggcag	39.48	3.51	18.79	1.62	11.98	10^{-6}	A			
		rs529539316:A	ctcctcctct	G→A	ctcctcccga	124.51	9.29	38.56	3.09	21.39	10^{-6}	A			
		rs940570715:A	tgctcctccc	G→A	atccctcctc	177.85	14.02	118.39	8.79	7.51	10^{-6}	A			
		rs995883443:T	ctcctcctcc	C→T	gccctgcctc	177.85	14.02	140.62	9.91	4.44	10^{-3}	B			
47	*ELN* (2006)	rs1222495699:G	ttggataagt	A→G	gtagatggat	4.84	0.52	9.11	0.81	9.05	10^{-6}	A	↓	within a biomedical cohort-based proteome study of glaucomatous optic neuropathy [191]: elastin-deficit in the optic nerve head from Caucasian Americans patients compared with African Americans patients as a population-specific biomedical molecular marker for POAG	▼
		rs1281291637:G	ccgggccttt	C→G	gtaattgtcc	10.19	0.93	13.76	1.18	4.81	10^{-3}	B			
		rs1554659636:C	ttcgtaattg	T→C	cccctccccg	78.02	5.81	100.98	8.06	4.73	10^{-3}	B			
		rs1584502289:C	gttggataag	T→C	agtagatgga	4.84	0.52	13.26	1.14	14.61	10^{-6}	A			
		rs1787811752:A	agccgggcct	T→A	tcgtaattgt	10.19	0.93	15.78	1.21	7.36	10^{-6}	A			
		rs45569239:G	ttggggataa	A→G	acgaggtgcg	6.05	0.53	11.08	0.98	9.76	10^{-6}	A			
		rs868987958:A	tgtgagccgg	G→A	cctttcgtaa	10.19	0.93	12.19	1.08	2.82	10^{-2}	C			
		rs1048169039:C (transcript ELN-216)	cgggcctttc	G→C	taattgtccc	10.19	0.93	15.96	1.27	7.41	10^{-6}	A			

Table S3. Cont.

#	Human Gene: *NCBI Gene Symbol* (NCBI Gene ID)	Candidate SNP marker: dbSNP ID:min [437]	5′ flank, 10 bp	WT →min	3 flank, 10 bp	K_D, nM, *in silico*: WT MEAN	± SEM	min MEAN	± SEM	Significance: Z	p	ϱ	Δ	Effect of changes in human gene expression on the development of primary open-angle glaucoma (POAG, ☼: "▼" aggravation, "▲" alleviation) [Reference]	☼ ▲▼
		rs1048169039:C (transcript ELN-203)	cgggcctttc	G→C	taattgtccc	53.39	6.16	36.17	2.58	5.74	10^{-6}	A			
		rs1048169039:A (transcript ELN-203)	cgggcctttc	G→A	taattgtccc	53.39	6.16	9.43	1.05	21.65	10^{-6}	A			
		rs1248584261:A	taggctttgg	G→A	gataaaacga	21.68	1.72	16.75	1.24	4.76	10^{-3}	B			
		rs1250660687:A	gccctctctc	C→A	ctccctcttt	78.15	5.41	70.73	4.87	2.04	0.05	D			
		rs1281291637:T	ccgggccttt	C→T	gtaattgtcc	53.39	6.16	11.68	1.55	17.31	10^{-6}	A			
		rs1361315738:T	ctccctcttt	C→T	cctcacagcc	78.15	5.41	46.79	3.20	10.53	10^{-6}	A			
		rs1383907662:T	cgtaattgtc	C→T	cctccccgcg	53.39	6.16	37.48	3.34	4.85	10^{-3}	B			
		rs1460153097:T	cgcccgccct	C→T	tctccctccc	53.39	6.16	42.75	3.15	3.25	10^{-2}	C			
		rs1554659636:C	ttcgtaattg	T→C	cccctccccg	53.39	6.16	19.45	3.02	10.44	10^{-6}	A			
		rs1554659770:C	tctccctccc	T→C	ctttccctca	78.15	5.41	69.75	4.84	2.32	0.05	D			
		rs1554659782:T	ccctctttcc	C→T	tcacagccga	78.15	5.41	44.00	2.92	11.97	10^{-6}	A			
		rs1563736738:A	cagccgacga	G→A	gcaacaatta	47.57	3.27	35.72	2.23	6.17	10^{-6}	A			
		rs1787812943:C	gcctttcgta	A→C	ttgtcccctc	53.39	6.16	41.33	5.33	2.96	10^{-2}	C			
		rs1787824417:A	tcccccctct	C→A	ccgccctccc	78.02	5.81	50.58	4.92	7.07	10^{-6}	A			
47	*ELN* (2006)	rs1787834618:T	ctccctccct	C→T	tttccctcac	78.15	5.41	41.44	2.78	13.16	10^{-6}	A	↑	within a cohort biomedical qPCR study [192]: ELN upregulation is a biomedical molecular marker for POAG	▼
		rs1787837222:T	ctttccctca	C→T	agccgacgag	47.57	3.27	23.54	1.98	12.96	10^{-6}	A			
		rs1787838678:A	tccctcacag	C→A	cgacgaggca	47.57	3.27	38.30	2.88	4.25	10^{-3}	B			
		rs1787842811:T	gacgaggcaa	C→T	aattaggctt	18.25	1.51	8.23	0.79	12.58	10^{-6}	A			
		rs41410045:G	ggggataaaa	C→G	gaggtgcgga	21.68	1.72	16.92	1.34	4.41	10^{-3}	B			
		rs41410045:T	ggggataaaa	C→T	gaggtgcgga	21.68	1.72	18.43	1.46	2.89	10^{-2}	C			
		rs537200597:A (transcript ELN-217)	cctcacagcc	G→A	acgaggcaac	78.15	5.41	66.54	5.15	3.10	10^{-2}	C			
		rs537200597:T (transcript ELN-206)	cctcacagcc	G→T	acgaggcaac	47.57	3.27	41.15	3.37	2.71	10^{-2}	C			
		rs540977629:A	ggttggataa	G→A	tagtagatgg	4.84	0.52	3.30	0.32	5.30	10^{-6}	A			
		rs559112365:A	aggctttggg	G→A	ataaaacgag	21.68	1.72	18.26	1.31	3.20	10^{-2}	C			
		rs576349806:T	ccctcacagc	C→T	gacgaggcaa	78.15	5.41	58.34	3.93	6.05	10^{-6}	A			
		rs782217420:T	taagtagtag	A→T	tggataagct	4.84	0.52	3.59	0.42	3.77	10^{-2}	B			
		rs782528515:A	cattcactat	C→A	ttctcttccc	4.84	0.52	4.04	0.42	2.41	0.05	D			
		rs972033864:A	cacagccgac	G→A	aggcaacaat	47.57	3.27	41.24	2.74	2.99	10^{-2}	C			
		rs972033864:T	cacagccgac	G→T	aggcaacaat	47.57	3.27	26.75	1.85	11.79	10^{-6}	A			
		rs1182889998:C	ctagctccaa	T→C	aaaagggaac	5.94	0.55	12.07	1.08	10.99	10^{-6}	A			
		rs1230104759:C	caagtaaagt	A→C	caagaagata	4.48	0.42	5.79	0.52	3.89	10^{-3}	B			
		rs1230104759:G	caagtaaagt	A→G	caagaagata	4.48	0.42	5.79	0.52	3.89	10^{-3}	B			
		rs1237371285:G	tagctccaat	A→G	aaagggaacg	5.94	0.55	12.07	1.08	10.99	10^{-6}	A			
		rs1246463977:A	aaaagggaac	G→A	acttttaccg	17.76	1.58	20.92	1.83	2.62	10^{-2}	C			
		rs1322460165:G	gggaacgact	T→G	ttaccgcagg	17.76	1.58	30.20	2.51	8.72	10^{-6}	A			
48	*ENO4* (387712)	rs1564840584:C	ggaacgactt	T→C	taccgcagga	17.76	1.58	25.57	2.31	5.74	10^{-6}	A	↓	within a cohort-based biomedical studies [193]: ENO4-deficit is a biomedical molecular marker for autoimmune thyroid disease, a risk-factor of which is ophthalmic surgery against POAG [194]	▼
		rs1589741921:G	aacgactttt	A→G	ccgcaggaag	17.76	1.58	39.49	3.57	12.59	10^{-6}	A			
		rs1846341271:G	cttcgcaagt	A→G	aagtacaaga	4.48	0.42	5.79	0.52	3.89	10^{-3}	B			
		rs187849003:G	aagtaaagta	C→G	aagaagataa	4.48	0.42	5.79	0.52	3.89	10^{-3}	B			
		rs553138860:T	caataaaagg	G→T	aacgactttt	5.94	0.55	6.84	0.55	2.30	0.05	D			
		rs940939057:A	gaacgacttt	T→A	accgcaggaa	17.76	1.58	19.97	1.52	2.00	0.05	D			
		rs940939057:C	gaacgacttt	T→C	accgcaggaa	17.76	1.58	39.49	3.57	12.59	10^{-6}	A			

Table S3. Cont.

Human Gene		Candidate SNP marker				K_D, nM, *in silico*				Significance				Effect of changes in human gene expression on the development of primary open-angle glaucoma (POAG, ☼: "▼" aggravation, "▲" alleviation) [Reference]	☼ ▲▼
#	*NCBI Gene Symbol* (NCBI Gene ID)	dbSNP ID:min [437]	5′ flank, 10 bp	WT →min	3 flank, 10 bp	WT MEAN±	WT SEM	min MEAN±	min SEM	Z	p	ϱ	Δ		
48	*ENO4* (387712)	rs1409027980:T	agccacgcga	G→T	atctgggggt	17.76	1.58	13.44	1.22	4.40	10^{-3}	B	↑	within a cohort-based biomedical studies [193]: ENO4-excess is a biomedical molecular marker for resistance against the autoimmune thyroid disease, a risk-factor of which is ophthalmic surgery against POAG [194]	▲
		rs1589751539:A	atattttctt	G→A	ctattaagga	4.48	0.42	2.95	0.32	5.87	10^{-6}	A			
		rs17095337:A	tccaataaaa	G→A	ggaacgactt	5.94	0.55	5.02	0.41	2.70	10^{-2}	C			
		rs1845983050:A	catcctagct	C→A	caataaaagg	5.94	0.55	4.58	0.38	4.20	10^{-3}	B			
		rs1845984414:T	acgactttta	C→T	cgcaggaaga	17.76	1.58	8.80	0.75	11.44	10^{-6}	A			
		rs1845984497:T	cgacttttac	C→T	gcaggaagaa	17.76	1.58	12.85	0.99	5.51	10^{-6}	A			
		rs1846340242:A	aaagccatat	T→A	ttcttgctat	4.48	0.42	3.76	0.39	2.50	0.05	D			
		rs368340847:T	agtacaagaa	G→T	ataaggggag	4.48	0.42	2.72	0.27	7.34	10^{-6}	A			
		rs771141549:A	atatcccact	G→A	taaagccata	4.48	0.42	3.14	0.40	4.48	10^{-3}	B			
49	*EPO* (2056)	rs1426138669:G	caacccaggc	A→G	tctctgagtc	43.44	3.32	57.44	4.27	5.24	10^{-6}	A	↓	within human POAG models using DBA/2J mice strain pups of subjected with adenoviral vector carrying mutant Epo gene with reduced erythropoietic [195]: this neuroprotective gene therapy preserved vision due to decreasing infiltration of peripheral immune cells, modulating microglial reactivity, and decreasing oxidative stress	▲
		rs1806720480:G	ggcatctctg	A→G	gtctccgccc	43.44	3.32	52.46	4.21	3.40	10^{-3}	B			
		rs927602488:C	catctctgag	T→C	ctccgcccaa	43.44	3.32	57.88	4.43	5.31	10^{-6}	A			
		rs982385184:C	caggcatctc	T→C	gagtctccgc	43.44	3.32	72.80	5.12	9.94	10^{-6}	A			
		rs1206140349:T	ttgcggaact	C→T	agcaacccag	43.44	3.32	28.85	2.19	7.60	10^{-6}	A	↑	within a biomedical cohort-based study [196]: EPO excess in plasma is a biomedical molecular marker for POAG	▼
		rs1454542091:T	cccccccaggg	G→T	aggtgtccgg	43.44	3.32	25.28	2.66	8.32	10^{-6}	A			
		rs1806720296:A	agcaacccag	G→A	catctctgag	43.44	3.32	35.86	2.52	3.69	10^{-3}	B			
		rs374690554:A	acccaggcat	C→A	tctgagtctc	43.44	3.32	12.66	1.07	21.60	10^{-6}	A			
		rs965329692:T	agctactttg	C→T	ggaactcagc	43.44	3.32	25.73	1.87	9.94	10^{-6}	A			
		rs976950831:A	gctactttgc	G→A	gaactcagca	43.44	3.32	37.40	2.60	2.90	10^{-2}	C			
50	*ESR1* (2099)	rs1162822573:T	tccctgaata	C→T	tcttgctttt	10.51	0.96	15.15	1.23	5.99	10^{-6}	A	↓	within an ethnopharmacological study of anti-diabetic Gegen Qinlian Decoction (GQD) within Chinese traditional medicine [197]: ESR1-deficit is a biomedical molecular marker for diabetes mellitus, which is comorbid with POAG, at least due to their mutual comorbidity with diabetic retinopathy [198]	▼
		rs1168394747:G	tgctaaatat	A→G	gctgtctgtg	2.40	0.27	4.74	0.60	8.08	10^{-6}	A			
		rs1186493569:T	gtttaaaaca	A→T	gccatatgga	4.72	0.41	5.65	0.52	2.84	10^{-2}	C			
		rs1308632189:G	cacactacgt	A→G	tttctagcca	3.75	0.35	4.87	0.53	3.61	10^{-3}	B			
		rs1346221194:C	tctttatttt	T→C	gagttactgc	8.35	0.70	9.88	0.81	2.86	10^{-2}	C			
		rs1398584072:G	gctgctaaat	A→G	tagctgtctg	2.40	0.27	6.70	0.54	14.82	10^{-6}	A			
		rs1562507288:G	aataatatta	A→G	ttctgtcctc	3.68	0.39	5.22	0.49	4.89	10^{-3}	B			
		rs1583378648:C	ctgctaaata	T→C	agctgtctgt	2.40	0.27	4.42	0.47	7.92	10^{-6}	A			
		rs1583379282:T	ctacgtattt	C→T	tagccaacga	3.75	0.35	4.42	0.43	2.40	0.05	D			
		rs1778008760:A	tcccccagct	G→A	ctaaatatag	2.40	0.27	2.80	0.18	2.40	0.05	D			
		rs1778021122:T	gtatttctag	C→T	caacgaggag	3.75	0.35	4.49	0.42	2.70	10^{-2}	C			
		rs1778095222:G	gaccagtact	T→G	aaagttggag	4.23	0.41	8.03	0.75	9.61	10^{-6}	A			
		rs1778370614:C	ccgcgagttt	A→C	aaacaagcca	4.72	0.41	6.64	0.64	5.29	10^{-6}	A			
		rs1792746014:G	aaataatatt	A→G	attctgtcct	3.68	0.39	7.03	0.56	9.69	10^{-6}	A			
		rs370499721:G	ctgaaataat	A→G	ttaattctgt	3.68	0.39	8.64	0.72	12.58	10^{-6}	A			
		rs748537766:G	ttcctgaaat	A→G	atattaattc	3.68	0.39	4.46	0.46	2.58	10^{-2}	C	↑	within a human neuroprotection cellular models using primary neocortical cell cultures established from mouse embryos on the 15th day of gestation, which were first exposed at either hypoxia or ischemic conditions and, next, treated with ospemifene as both new promising neuroprotective medication and novel selective estrogen receptor modulator simultaneously [199]: four-fold ESR1 protein excess compared to the normal condition along with neuroprotective effects by means of both proapoptotic proteins suppression and antiapoptotic proteins upregulation that altogether may slow the progression of POAG in terms of neurodegeneration of both the optic nerve and retinal ganglion cells	▲
		rs1473184683:T	ctcctccctc	C→T	ctgaatactc	10.51	0.96	9.15	0.83	2.16	0.05	D			
		rs1562423474:T	ctcgggagac	C→T	agtacttaaa	4.23	0.41	3.09	0.33	4.35	10^{-3}	B			
		rs1777575174:T	ctccctccct	G→T	aatactcttg	10.51	0.96	2.63	0.26	20.67	10^{-6}	A			
		rs1778020016:T	atcctatgta	C→T	acactacgta	3.75	0.35	2.22	0.22	7.77	10^{-6}	A			
		rs1778087238:A	taatgcatat	G→A	agctcgggag	4.23	0.41	3.08	0.31	4.56	10^{-3}	B			
		rs1778097239:C	gtacttaaag	T→C	tggaggcccg	4.23	0.41	2.94	0.39	4.48	10^{-3}	B			
		rs1778370039:T	ggtgtcccgc	G→T	agtttaaaac	4.72	0.41	3.13	0.28	6.62	10^{-6}	A			
		rs1778859762:T	ctttattttt	G→T	agttactgca	8.35	0.70	4.10	0.36	11.71	10^{-6}	A			
		rs1792743742:T	gtttcctgaa	A→T	taatattaat	3.68	0.39	2.98	0.32	2.77	10^{-2}	C			

Table S3. Cont.

#	Human Gene: *NCBI Gene Symbol* (NCBI Gene ID)	Candidate SNP marker: dbSNP ID:min [437]	5′ flank, 10 bp	WT →min	3 flank, 10 bp	K$_D$, nM, *in silico*: WT MEAN	± SEM	min MEAN	± SEM	Significance: Z	p	ϱ	Δ	Effect of changes in human gene expression on the development of primary open-angle glaucoma (POAG, ☼: "▼" aggravation, "▲" alleviation) [Reference]	☼ ▲▼
51	*ESR2* (2100)	rs1012562006:C	gggctcaggc	A→C	ctcggtcacg	23.04	2.45	52.61	4.54	12.06	10^{-6}	A	↓	according to a biomedical case report [167]: a complication of tamoxifen therapy for ESR2-deficient pT1 breast benign tumor to prophylaxis carcinogenesis may be ocular toxicity that may cause POAG, retinopathy and loss of visual acuity	▼
		rs1181520534:G	gcgggcttca	T→G	ggagaatcca	6.82	0.63	27.48	2.31	22.39	10^{-6}	A			
		rs1236249379:G	tgagaacatt	A→G	ccacgaatct	4.73	0.39	9.49	0.89	11.16	10^{-6}	A			
		rs142119488:G	ttcataagct	A→G	tccacgcggg	6.82	0.63	11.06	1.04	7.35	10^{-6}	A			
		rs2076715464:C	tatttaaatt	T→C	tttaatgaaa	3.86	0.44	5.58	0.82	3.94	10^{-3}	B			
		rs2076925482:C	tctcggtctt	T→C	ctctaccctc	5.98	0.60	21.53	1.51	21.03	10^{-6}	A			
		rs2077554417:G	gggcttcata	A→G	agaatccacg	6.82	0.63	9.95	0.88	5.91	10^{-6}	A			
		rs35036378:G	cctctcggtc	T→G	ccctctaccc	5.98	0.60	8.19	0.67	4.88	10^{-3}	B			
		rs766797386:T	ttaaaaggaa	G→T	cctctcggtc	5.98	0.60	7.31	0.61	3.08	10^{-2}	C			
		rs894576775:G	aatatttaaa	T→G	tttttaatga	3.86	0.44	4.61	0.56	2.11	0.05	D			
		rs1426058466:T	tttaaatttt	C→T	taatgaaaat	3.86	0.44	1.80	0.17	10.28	10^{-6}	A	↑	within a human neuroprotection cellular models using primary neocortical cell cultures established from mouse embryos on the 15th day of gestation, which were first exposed at either hypoxia or ischemic conditions and, next, treated with ospemifene as both new promising neuroprotective medication and novel selective estrogen receptor modulator simultaneously [199]: nine-fold ESR2 protein excess compared to the ischemic condition along with neuroprotective effects by means of both proapoptotic proteins suppression and antiapoptotic proteins upregulation that altogether may slow the progression of POAG in terms of neurodegeneration of both the optic nerve and retinal ganglion cells	▲
		rs1460728522:T	attttgtcac	C→T	tcgtagtctg	26.03	1.87	22.24	1.58	3.12	10^{-2}	C			
		rs2076926117:T	tgggctcagg	C→T	gctcggtcac	23.04	2.45	6.38	1.08	12.84	10^{-6}	A			
		rs2077555436:A	cagggagggt	C→A	ttgccagggc	26.03	1.87	8.71	1.25	13.66	10^{-6}	A			
		rs746385847:A	acattataat	G→A	aatctttgag	4.73	0.39	2.60	0.28	8.78	10^{-6}	A			
		rs749310099:T	acgcgggctt	C→T	ttggagaatc	6.82	0.63	3.20	0.42	9.41	10^{-6}	A			
		rs754023746:A	cagctgttat	C→A	aggtgttttc	4.73	0.39	3.53	0.37	4.41	10^{-e}	B			
		rs910482422:T	ggattttgtc	A→T	ggtcgtagtc	26.03	1.87	21.42	1.61	3.75	10^{-3}	B			
		rs923592432:T	ttttgtcacc	C→T	cgtagtctgg	26.03	1.87	23.03	1.72	2.37	0.05	D			
52	*FAS* (355)	rs1026417557:G	agtgacacac	A→G	ggtgttcaaa	18.00	1.20	22.47	1.46	4.77	10^{-3}	B	↓	within human disease model using Fas-deficient mice subjected with an single intracameral injection of microbeads elevating intraocular pressure [200]: resistance to POAG, which was then confirmed in an independent experiment using ONL1204 as a low molecular weight peptide inhibitor against Fas	▲
		rs1057289230:C	gacccgctca	G→C	tacggagttg	25.52	2.96	30.69	2.99	2.44	0.05	D			
		rs1196351048:C	gaacacaccc	T→C	gaggccagcc	48.22	3.84	61.14	4.65	4.31	10^{-3}	B			
		rs1203247594:C	atgcccattt	G→C	tgcaacgaac	15.31	1.22	21.84	1.58	6.59	10^{-6}	A			
		rs1223335656:T	ggcactggca	C→T	ggaacacacc	48.22	3.84	56.71	4.59	2.86	10^{-2}	C			
		rs12264245:G	gaaaggaagt	A→G	atacaggaag	9.55	1.07	14.29	1.43	5.36	10^{-6}	A			
		rs1318126055:C	agggaagcgg	T→C	ttacgagtga	16.96	1.53	29.06	2.51	8.61	10^{-6}	A			
		rs1336742597:G	acaggtgttc	A→G	aagacgcttc	18.00	1.20	31.27	2.19	11.43	10^{-6}	A			
		rs1353528671:C	ctcctacctc	T→C	ggtgagccct	41.25	3.48	48.14	4.93	2.33	0.05	D			
		rs1450417581:G	accccgtctt	A→G	gtcccgggga	18.09	1.42	59.99	4.69	21.66	10^{-6}	A			
		rs1564669941:G	cccgctcagt	A→G	cggagttggg	25.52	2.96	57.95	4.32	11.9	10^{-6}	A			
		rs1589439153:C	cacggaacac	A→C	ccctgaggcc	48.22	3.84	65.61	4.41	5.91	10^{-6}	A			
		rs1846895973:C	gaagtaatac	A→C	ggaaggaagt	9.55	1.07	14.29	1.43	5.36	10^{-6}	A			
		rs1847115762:C	ggtgttcaaa	G→C	acgcttctgg	18.00	1.20	26.26	1.78	7.95	10^{-6}	A			
		rs1847119197:A	ggaagcggtt	T→A	acgagtgact	16.96	1.53	20.86	1.60	3.49	10^{-3}	B			
		rs1847138768:C	ccctcctacc	T→C	ctggtgagcc	41.25	3.48	50.87	3.65	3.79	10^{-3}	B			
		rs1847207382:G	ttcagaacag	A→G	tattgctcat	15.53	1.38	23.20	2.02	6.43	10^{-6}	A			
		rs371701570:C	taccccgtct	T→C	agtcccgggg	18.09	1.42	59.99	4.69	21.66	10^{-6}	A			
		rs557366318:C	agcggtttac	G→C	agtgacttgg	16.96	1.53	23.49	2.15	5.07	10^{-6}	A			
		rs557982780:G	aggaagtaat	A→G	caggaaggaa	9.55	1.07	14.29	1.43	5.36	10^{-6}	A			
		rs748639752:G	ggacccgctc	A→G	gtacggagtt	25.52	2.96	41.19	3.99	6.34	10^{-6}	A			
		rs760596246:G	ggaccctcct	A→G	cctctggtga	41.25	3.48	50.87	3.65	3.79	10^{-3}	B			
		rs769961396:C	aggtgttcaa	A→C	gacgcttctg	18.00	1.20	31.27	2.19	11.43	10^{-6}	A			
		rs942198129:G	ccttcagaac	A→G	gatattgctc	15.53	1.38	23.44	2.02	6.64	10^{-6}	A			
		rs965461197:T	gtgttcaaag	A→T	cgcttctggg	18.00	1.20	20.90	1.34	3.23	10^{-2}	C			

Table S3. Cont.

Human Gene		Candidate SNP marker				K_D, nM, *in silico*				Significance				Effect of changes in human gene expression on the development of primary open-angle glaucoma (POAG, ☼: "▼" aggravation, "▲" alleviation) [Reference]	☼ ▲ ▼
#	*NCBI Gene Symbol* (NCBI Gene ID)	dbSNP ID:min [437]	5′ flank, 10 bp	WT →min	3 flank, 10 bp	WT MEAN	± SEM	min MEAN	± SEM	Z	p	ϱ	Δ		
		rs1032283712:A	gtgacacaca	G→A	gtgttcaaag	18.00	1.20	14.31	0.97	4.83	10^{-3}	B			
		rs111675393:A	aggaagtagt	G→A	caggaaggaa	9.55	1.07	5.86	0.76	5.69	10^{-6}	A			
		rs1159155936:G	gatattgctc	A→G	ttttctggca	15.53	1.38	13.23	1.12	2.61	10^{-2}	C			
		rs1190761605:G	ccgtcttagt	C→G	ccggggatag	18.09	1.42	14.90	1.20	3.47	10^{-3}	B			
		rs1192530320:T	aagagtgaca	C→T	acaggtgttc	18.00	1.20	11.55	1.18	7.29	10^{-6}	A			
		rs1229890773:A	cccgcgggtt	G→A	gtggacccgc	48.22	3.84	25.51	3.03	8.91	10^{-6}	A			
		rs1276191966:A	tggtggaccc	G→A	ctcagtacgg	25.52	2.96	20.97	2.22	2.50	0.05	D			
		rs1276191966:T	tggtggaccc	G→T	ctcagtacgg	25.52	2.96	18.69	2.40	3.6	10^{-6}	A			
		rs1312703118:T	tctggaccct	C→T	ctacctctgg	41.25	3.48	17.74	1.73	13.11	10^{-6}	A			
		rs1387233818:C	cagatattgc	T→C	cattttctgg	15.53	1.38	13.51	1.23	2.20	0.05	D			
		rs1399455393:T	tgagggaagc	G→T	gtttacgagt	16.96	1.53	13.68	1.29	3.30	10^{-3}	B			
		rs1419652980:A	aagtaataca	G→A	gaaggaagta	9.55	1.07	3.86	0.47	10.95	10^{-6}	A			
		rs1439962148:A	gctgcctctt	C→A	tcccgcgggt	48.22	3.84	12.59	1.10	22.72	10^{-6}	A			
		rs1464418725:T	ggaagtggta	C→T	aggaaggaag	9.55	1.07	6.92	0.68	4.30	10^{-3}	B			
		rs1475203128:T	ttagtcccgg	G→T	gataggcaaa	18.09	1.42	16.16	1.31	2.00	0.05	D			
		rs1490084330:T	ggaacacacc	C→T	tgaggccagc	48.22	3.84	37.48	2.52	4.83	10^{-3}	B			
		rs1589439843:T	cttagtcccg	G→T	ggataggcaa	18.09	1.42	16.00	1.28	2.19	0.05	D			
		rs1846895061:T	cagaaaggaa	G→T	taatacagga	9.55	1.07	8.11	0.73	2.26	0.05	D			
		rs1847109981:T	cggggctttt	C→T	gtgagctcgt	18.00	1.20	14.55	1.05	4.33	10^{-3}	B			
		rs1847113684:A	gcgcaagagt	G→A	acacacaggt	18.00	1.20	13.95	1.22	4.63	10^{-3}	B			
		rs1847114138:T	gagtgacaca	C→T	aggtgttcaa	18.00	1.20	10.99	1.06	8.40	10^{-6}	A			
		rs1847114850:A	acacacaggt	G→A	ttcaaagacg	18.00	1.20	7.74	0.70	15.07	10^{-6}	A			
		rs1847115059:A	cacaggtgtt	C→A	aaagacgctt	18.00	1.20	6.31	0.49	20.59	10^{-6}	A			
		rs1847119430:A	aagcggttta	C→A	gagtgacttg	16.96	1.53	9.56	0.77	9.47	10^{-6}	A			
52	*FAS* (355)	rs1847123715:G	cggaacacac	C→G	ctgaggccag	48.22	3.84	37.95	3.19	4.13	10^{-3}	B	↑	within human disease model using mice [200]: Fas-activation induced retinal ganglion cell apoptosis, glial activation, and inflammation as a complication of POAG	▼
		rs1847138496:T	gaccctccta	C→T	ctctggtgag	41.25	3.48	23.36	1.91	9.67	10^{-6}	A			
		rs1847143134:G	cttaccccgt	C→G	ttagtcccgg	18.09	1.42	14.35	1.70	3.26	10^{-2}	C			
		rs1847144814:T	gtcttagtcc	C→T	ggggataggc	18.09	1.42	14.27	1.15	4.23	10^{-3}	B			
		rs1847207382:T	ttcagaacag	A→T	tattgctcat	15.53	1.38	11.21	1.05	5.03	10^{-6}	A			
		rs367885573:A	ggcttacccc	G→A	tcttagtccc	41.25	3.48	30.04	2.70	5.15	10^{-6}	A			
		rs367885573:T	ggcttacccc	G→T	tcttagtccc	41.25	3.48	34.44	2.41	3.29	10^{-3}	B			
		rs376591116:T	tagtcccggg	G→T	ataggcaaag	18.09	1.42	12.98	1.43	4.90	10^{-6}	A			
		rs535323064:G	aaaggaagta	A→G	tacaggaagg	9.55	1.07	5.86	0.74	5.76	10^{-6}	A			
		rs557160851:A	ggaaggaagt	G→A	gtacaggaag	9.55	1.07	5.86	0.76	5.69	10^{-6}	A			
		rs557366318:A	agcggtttac	G→A	agtgacttgg	16.96	1.53	9.65	0.87	8.83	10^{-6}	A			
		rs558072404:A	tgagccctct	C→A	ctgcccgggt	41.25	3.48	29.95	2.70	5.19	10^{-6}	A			
		rs575570390:G	ttacgagtga	C→G	ttggctggag	48.22	3.84	42.26	2.95	2.49	0.05	D			
		rs61852593:A	aggaagtagt	G→A	caggaaggaa	9.55	1.07	5.86	0.76	5.69	10^{-6}	A			
		rs61852594:A	ggaaggaagt	G→A	gtacaggaag	9.55	1.07	5.86	0.76	5.69	10^{-6}	A			
		rs750066201:T	cgggtggagg	C→T	ttaccccgtc	41.25	3.48	29.73	2.92	5.06	10^{-6}	A			
		rs757128589:G	acagaaagga	A→G	gtaatacagg	9.55	1.07	7.74	1.13	2.28	0.05	D			
		rs759174021:T	tcagtacgga	G→T	ttggggaagc	25.52	2.96	19.52	1.54	3.83	10^{-3}	B			
		rs767394348:A	acggaacaca	C→A	cctgaggcca	48.22	3.84	33.62	2.50	6.61	10^{-6}	A			
		rs775316075:T	catctggacc	C→T	tcctacctct	41.25	3.48	24.49	2.44	7.98	10^{-6}	A			
		rs778357057:A	tcttagtccc	G→A	gggataggca	18.09	1.42	15.03	1.21	3.30	10^{-3}	B			
		rs781677949:A	gaggcttacc	C→A	cgtcttagtc	41.25	3.48	24.26	1.82	9.40	10^{-6}	A			
		rs781677949:T	gaggcttacc	C→T	cgtcttagtc	41.25	3.48	27.58	2.51	6.49	10^{-6}	A			
		rs981359669:A	agtaatacag	G→A	aaggaagtag	9.55	1.07	6.92	0.61	4.49	10^{-3}	B			
		rs992028982:T	cccatttgtg	C→T	aacgaaccct	15.31	1.22	5.65	0.71	13.40	10^{-6}	A			

Table S3. Cont.

Human Gene		Candidate SNP marker				K_D, nM, *in silico*				Significance				Effect of changes in human gene expression on the development of primary open-angle glaucoma (POAG, ☼: "▼" aggravation, "▲" alleviation) [Reference]	☼ ▲ ▼
#	*NCBI Gene Symbol* (NCBI Gene ID)	dbSNP ID:min [437]	5′ flank, 10 bp	WT →min	3 flank, 10 bp	WT MEAN	WT ± SEM	min MEAN	min ± SEM	Z	p	ϱ	Δ		
53	*FASLG* (356)	rs1659076289:G	tggaaactct	A→G	taagagagat	3.91	0.38	11.06	1.15	14.57	10^{-6}	A	↓	within human neuroprotection models using Faslg-knockout mice [201]: reduced death of retinal ganglion cells that may slow the progression of POAG in terms of neurodegeneration of retinal ganglion cells	▲
		rs920544506:T	ctataagaga	G→T	atccagcttg	3.91	0.38	4.64	0.46	2.44	0.05	D			
		rs971983536:C	ctatggaaac	T→C	ctataagaga	3.91	0.38	4.64	0.47	2.43	0.05	D			
		rs971983536:G	ctatggaaac	T→G	ctataagaga	3.91	0.38	4.48	0.44	1.96	0.05	D			
		rs971983536:A	ctatggaaac	T→A	ctataagaga	3.91	0.38	2.56	0.25	6.06	10^{-6}	A	↑	within human POAG models using a hybrid mice line expressing only full-length Faslg responsible for accelerated retinal ganglion cell death, which was injected soluble short-length Faslg [201]: reduced death of retinal ganglion cells that may slow the progression of POAG in terms of neurodegeneration of retinal ganglion cells	
54	*FNDC3B* (64778)	rs111903995:C	ggcggtaacc	G→C	gcggccgcgg	55.17	6.10	69.72	9.22	2.72	10^{-2}	C	↓	within a cohort-based biomedical RNA-Seq study [202]: FNDC3B-downregulation can be a biomedical molecular marker for stable state in POAG	▲
		rs1241068850:C	attttgtaga	T→C	gtaaagggct	6.71	0.67	10.79	1.23	6.25	10^{-6}	A			
		rs1286512664:C	caaaccctcc	T→C	ggtagttatt	17.15	1.58	22.07	2.01	3.90	10^{-3}	B			
		rs1303619076:G	cctggtagtt	A→G	tttgagcggc	17.15	1.58	24.50	2.41	5.30	10^{-6}	A			
		rs1341836337:G	cggccggtat	A→G	ttcggcggtt	7.20	1.12	10.94	1.00	4.64	10^{-3}	B			
		rs1445814021:G	ttgtagatgt	A→G	aagggcttag	6.71	0.67	10.79	1.23	6.25	10^{-6}	A			
		rs1715899806:G	ggcggccggt	A→G	tattcggcgg	7.20	1.12	10.94	1.00	4.64	10^{-3}	B			
		rs1715910824:G	ccctcctggt	A→G	gttatttgag	17.15	1.58	35.31	2.65	12.14	10^{-6}	A			
		rs171592920:T	gtagttattt	G→T	agcggcggcg	17.15	1.58	21.12	1.99	3.15	10^{-2}	C			
		rs1715977708:G	tgggggcggt	A→G	accggcggcc	55.17	6.10	107.68	7.93	10.07	10^{-6}	A			
		rs971501866:C	tgtaaagggc	T→C	tagatttcag	6.71	0.67	9.84	0.79	5.94	10^{-6}	A			
		rs111903995:A	ggcggtaacc	G→A	gcggccgcgg	55.17	6.10	47.35	4.73	2.05	0.05	D	↑	within a cohort-based biomedical RNA-Seq study [202]: FNDC3B-upregulation can be a biomedical molecular marker for progression in POAG	▼
		rs1177557208:A	ttggaatctt	G→A	tatagagaac	9.31	1.09	3.40	0.44	11.49	10^{-6}	A			
		rs1398272625:A	ccggtatatt	C→A	ggcggtttac	7.20	1.12	2.49	0.27	11.27	10^{-6}	A			
		rs1576807161:A	gcggtttacg	G→A	taatgggcgg	7.20	1.12	5.42	0.48	3.18	10^{-2}	C			
		rs1576888854:T	tgaatggatt	C→T	ctataggagt	6.71	0.67	5.38	0.57	3.05	10^{-2}	C			
		rs1715910679:A	aaccctcctg	G→A	tagttatttg	17.15	1.58	7.60	0.82	11.42	10^{-6}	A			
		rs1715910947:A	ctcctggtag	T→A	tatttgagcg	17.15	1.58	11.14	1.12	6.31	10^{-6}	A			
		rs1715977545:T	gctgggggcg	G→T	taaccggcgg	55.17	6.10	27.34	3.23	8.67	10^{-6}	A			
		rs1715977898:A	gggcggtaac	C→A	ggcggccgcg	55.17	6.10	24.03	2.18	11.62	10^{-6}	A			
		rs567974546:A	tcctggtagt	T→A	atttgagcgg	17.15	1.58	14.77	1.44	2.22	0.05	D			
		rs933332049:A	tgtggcggcc	G→A	gtatattcgg	7.20	1.12	5.21	0.79	2.99	10^{-2}	C			
		rs946030142:A	ggccccaaat	C→A	cccatgtggt	17.15	1.58	12.64	1.15	4.71	10^{-3}	B			
		rs971672019:T	tcaaaccctc	C→T	tggtagttat	17.15	1.58	12.95	1.27	4.17	10^{-3}	B			
		rs973015746:T	cggaatcccc	C→T	atcaaaccct	17.15	1.58	13.66	1.26	3.49	10^{-3}	B			
		rs981795347:A	gtcccgacat	C→A	attgacatgg	17.15	1.58	10.44	0.83	8.14	10^{-6}	A			
		rs981795347:T	gtcccgacat	C→T	attgacatgg	17.15	1.58	12.20	1.39	4.64	10^{-3}	B			
		rs997478002:T	gatgtttttg	G→T	aatcttgtat	9.31	1.09	6.90	0.66	3.96	10^{-3}	B			
55	*FOXC1* (2296)	rs1019856198:A	gtgtaactgc	G→A	taaaaaagtc	8.42	0.71	4.37	0.37	10.94	10^{-6}	A	↑	within human sepsis-associated neurodegeneration encephalopathy models using 8-10 month aged male C57BL/6J mice subjected with artificially caused post-surgery sepsis, which both downregulated Foxc1 and caused cognitive disorders as detected experimentally, and, next, transfected with adenoviral vector carrying the mouse Foxc1 gene [204]: Foxc1 overexpression along with improved cognitive abilities that seems to be something like post-traumatic neuroregeneration alleviating POAG	▲
		rs1581372897:T	gctgcccgga	A→T	aaaagtgtaa	8.42	0.71	7.29	0.62	2.42	0.05	D			
		rs986925320:A	ggaaaaaagt	G→A	taactgcgta	8.42	0.71	2.87	0.31	15.77	10^{-6}	A			
56	*GAS7* (8522)	rs1180966217:G	tggggtgccc	A→G	ggaacctccg	46.55	3.35	75.01	6.67	8.34	10^{-6}	A	↓	within a biomedical cohort-based RNA-Seq study [205]: GAS7-downregulation can be a biomedical molecular biomarker of POAG	▼
		rs1184356732:C	acaagttgtt	A→C	gaaagtccag	14.06	1.09	24.77	1.82	10.63	10^{-6}	A			
		rs1323721247:A	gtttgtggga	G→A	cccctggcgc	51.15	3.81	57.81	3.89	2.44	0.05	D			
		rs1418075392:C	tgcccagaac	A→C	ctccgctggg	46.55	3.35	75.01	6.67	8.34	10^{-6}	A			
		rs1597734458:T	tgacctcagc	C→T	attgtttgtt	9.33	0.67	10.45	0.68	2.32	0.05	D			
		rs1597763424:C	gagtcatggg	T→C	gctgtttgtg	51.15	3.81	59.75	4.22	3.03	10^{-2}	C			
		rs1597763424:G	gagtcatggg	T→G	gctgtttgtg	51.15	3.81	59.75	4.22	3.03	10^{-2}	C			
		rs1597850817:G	ccccccagcc	C→G	cctgagcccc	61.05	6.11	78.94	7.19	3.80	10^{-3}	B			

Table S3. Cont.

#	Human Gene: *NCBI Gene Symbol* (NCBI Gene ID)	Candidate SNP marker: dbSNP ID:min [437]	5′ flank, 10 bp	WT →min	3 flank, 10 bp	K_D, nM, *in silico*: WT MEAN	± SEM	min MEAN	± SEM	Significance: Z	p	ϱ	Δ	Effect of changes in human gene expression on the development of primary open-angle glaucoma (POAG, ☼: "▼" aggravation, "▲" alleviation) [Reference]	☼ ▲ ▼
56	*GAS7* (8522)	rs17208387:T	tgttaatttc	C→T	ccagaacaag	14.06	1.09	16.47	1.03	3.18	10^{-2}	C	↓	within a biomedical cohort-based RNA-Seq study [205]: GAS7-downregulation can be a biomedical molecular biomarker of POAG	▼
		rs187919709:C	gcccagagct	A→C	cccgcccccc	61.05	6.11	128.23	9.42	11.96	10^{-6}	A			
		rs187919709:G	gcccagagct	A→G	cccgcccccc	61.05	6.11	128.23	9.42	11.96	10^{-6}	A			
		rs191755777:C	caacgttttt	T→C	gtgacctcag	9.33	0.67	22.65	1.66	17.22	10^{-6}	A			
		rs192411650:A	cttcagagct	G→A	taggctgaca	0.77	0.09	0.90	0.09	1.98	0.05	D			
		rs2069392838:C	actgagagaa	G→C	ccaaatcaca	32.57	2.04	39.37	2.88	3.94	10^{-3}	B			
		rs2069392964:G	gactgagaga	A→G	cccaaatcac	32.57	2.04	42.93	3.59	5.29	10^{-6}	A			
		rs2072211122:G	gtgattttct	A→G	gcaccccgtg	13.98	1.28	30.08	1.79	14.06	10^{-6}	A			
		rs2072760440:T	gttaatttcc	C→T	cagaacaagt	14.06	1.09	16.85	1.04	3.67	10^{-3}	B			
		rs2072762622:C	cagccaacgt	T→C	tgttgtgacc	9.33	0.67	16.57	1.25	11.01	10^{-6}	A			
		rs2073199171:C	tctattttaa	T→C	aacgtcaagt	5.73	0.38	7.31	0.43	5.52	10^{-6}	A			
		rs2073199214:C	gtctctattt	T→C	ggcaacgtca	5.73	0.38	11.34	0.77	14.33	10^{-6}	A			
		rs2073218897:C	gtgggagtca	T→C	tggcgctgtt	51.15	3.81	97.75	7.53	12.09	10^{-6}	A			
		rs2073218927:G	tgtgggagtc	A→G	ctggcgctgt	51.15	3.81	79.63	6.12	8.27	10^{-6}	A			
		rs2073842621:C	gtggtgtcca	T→C	ctggctggtg	13.91	1.05	15.55	1.15	2.12	0.05	D			
		rs377602878:T	ttgttaattt	C→T	tccagaacaa	14.06	1.09	19.22	1.31	6.07	10^{-6}	A			
		rs904619784:G	gtgtccatag	T→G	gctggtgtgt	13.91	1.05	21.04	1.68	7.52	10^{-6}	A			
		rs947045890:G	gagctgttat	A→G	tgacaacttc	0.77	0.09	2.36	0.24	14.39	10^{-6}	A			
		rs1036382144:C	ccagaacaag	T→C	atctggaaag	14.06	1.09	12.36	1.23	2.04	0.05	D	↑	according to the very first study focused on the mice Gas7 gene localization, sequencing and phenotypically characterization [206]: Gas7 overexpression can manifest in termination in neuron differentiation followed neurite-like outgrowth, which can improve post-traumatic retinal neuroregeneration according to human POAG models using mouse retinal neuronal precursor cell line RGC5 differentiating into either cone photoreceptor or ganglion cells due to treatment with the histone deacetylase inhibitor Trichostatin A (TSA) as a new promising regenerative medication extracted and purified from the bacterium *Streptomyces hygroscopicus* [207]	▲
		rs111406589:T	ccccaaatca	C→T	aggggttaac	32.57	2.04	13.69	1.19	16.17	10^{-6}	A			
		rs1205810866:A	ctgacctcag	G→A	gtgtgctgcc	12.82	1.22	10.45	0.94	3.13	10^{-2}	C			
		rs1259757669:T	gctccattag	C→T	gagaaagtct	13.98	1.28	10.94	1.32	3.23	10^{-2}	C			
		rs1272653890:C	ccagcccaga	G→C	agccccgccc	61.05	6.11	39.36	4.23	5.98	10^{-6}	A			
		rs1281515648:A	cattgcatct	G→A	gcctccagaa	14.06	1.09	11.59	1.06	3.23	10^{-2}	C			
		rs1341304818:T	gtgtggtgtc	C→T	tgctggctgg	13.91	1.05	6.92	0.75	10.55	10^{-6}	A			
		rs1390562062:T	tagcgacttg	C→T	tggccctggg	46.55	3.35	24.59	1.79	12.47	10^{-6}	A			
		rs1394024297:A	ctagcgactt	G→A	ctggccctgg	46.55	3.35	17.58	1.75	15.82	10^{-6}	A			
		rs150614175:T	gcccagaaca	C→T	tccgctgggg	46.55	3.35	40.48	3.00	2.71	10^{-2}	C			
		rs2069391150:A	cagagcctgt	G→A	agcagttctc	32.57	2.04	20.36	2.65	6.50	10^{-6}	A			
		rs2069393076:C	cagactgaga	G→C	tccccaaatc	32.57	2.04	24.61	1.69	6.04	10^{-6}	A			
		rs2072202142:T	gacctcaggg	G→T	gtgctgcctc	12.82	1.22	7.05	0.68	8.80	10^{-6}	A			
		rs2072210812:A	gattttctag	G→A	accccgtgag	13.98	1.28	12.03	1.11	2.31	0.05	D			
		rs2072211319:A	gagtgatttt	C→A	cagcaccccg	13.98	1.28	3.10	0.31	22.11	10^{-6}	A			
		rs2072756929:A	gccccatttc	C→A	gacttgcctc	46.55	3.35	31.35	2.44	7.46	10^{-6}	A			
		rs2072758228:A	gtgcccagaa	C→A	cctccgctgg	46.55	3.35	26.53	1.90	11.07	10^{-6}	A			
		rs2074559317:C	cccagagcta	G→C	ccgcccccca	61.05	6.11	42.97	4.38	4.92	10^{-6}	A			
		rs2074559396:C	ccccagccca	G→C	tgagccccgc	61.05	6.11	37.40	3.75	6.92	10^{-6}	A			
		rs531623714:A	cctgggctag	C→A	agaacactgg	46.55	3.35	31.05	3.32	6.27	10^{-6}	A			
		rs531623714:T	cctgggctag	C→T	agaacactgg	46.55	3.35	41.41	3.39	2.15	0.05	D			
		rs935055165:T	gcagttctct	C→T	cctggtttgg	32.57	2.04	19.34	1.61	10.03	10^{-6}	A			
		rs943711587:G	tcaagtctct	A→G	ccacggcaac	5.73	0.38	4.97	0.34	2.99	10^{-2}	C			
		rs975267129:A	cccagaacac	T→A	ccgctggggt	46.55	3.35	38.86	3.28	3.26	10^{-2}	C			
		rs988267760:T	ctcagccaac	G→T	tttgttgtga	9.33	0.67	7.59	0.60	3.85	10^{-3}	B			
		rs988354905:A	agtcatgggt	G→A	ctgtttgtgg	51.15	3.81	33.81	3.12	6.99	10^{-6}	A			

Table S3. Cont.

#	Human Gene: *NCBI Gene Symbol* (NCBI Gene ID)	Candidate SNP marker: dbSNP ID:min [437]	5′ flank, 10 bp	WT →min	3 flank, 10 bp	K_D, nM, *in silico*: WT MEAN	± SEM	min MEAN	± SEM	Significance: Z	p	ϱ	Δ	Effect of changes in human gene expression on the development of primary open-angle glaucoma (POAG, ☼: "▼" aggravation, "▲" alleviation) [Reference]	☼ ▲▼
57	*GLIS3* (169792)	rs1190965937:G	gacctgccca	A→G	ggcaagttaa	14.72	1.69	20.58	1.63	4.79	10^{-3}	B	↓	within human disease models using Glis3-deficient mice [208]: susceptibility to neonatal diabetes, which is comorbid with POAG at list within diabetic retinopathy [198]	▼
		rs1287292949:C	agccgcgtcc	A→C	cctctccctg	72.20	6.34	198.76	14.68	17.66	10^{-6}	A			
		rs1287292949:G	agccgcgtcc	A→G	cctctccctg	72.20	6.34	198.76	14.68	17.66	10^{-6}	A			
		rs1380775449:G	ccgcgtccac	A→G	tctccctgca	72.20	6.34	198.76	14.68	17.66	10^{-6}	A			
		rs141177375:C	tctgatttct	A→C	tcccagtcac	8.22	0.81	22.13	1.47	16.61	10^{-6}	A			
		rs141177375:G	tctgatttct	A→G	tcccagtcac	8.22	0.81	22.13	1.47	16.61	10^{-6}	A			
		rs1587360326:C	gctctctcct	A→C	gtgagccgcc	21.27	1.59	25.40	2.33	3.00	10^{-2}	C			
		rs1587364843:G	tggatggggg	T→G	gtccccccgt	22.02	1.57	27.98	2.25	4.46	10^{-3}	B			
		rs1816805254:G	tctcctagac	A→G	gccgccggct	21.27	1.59	27.24	2.39	4.29	10^{-3}	B			
		rs182162506:C	ttctgatttc	T→C	ttcccagtca	8.22	0.81	22.13	1.47	16.61	10^{-6}	A			
		rs1834337845:G	tgacctgccc	A→G	aggcaagtta	14.72	1.69	17.61	1.23	2.66	10^{-2}	C			
		rs557604466:T	agtgagccgc	C→T	tggaaagcgt	21.27	1.59	23.83	1.89	2.09	0.05	D			
		rs901338501:G	ccggctctct	C→G	tgagtgagcc	21.27	1.59	25.32	1.75	3.43	10^{-3}	B			
		rs955862342:G	ctgcccaagt	A→G	aagttaatga	14.72	1.69	31.66	2.68	10.72	10^{-6}	A			
		rs981115967:G	tgcagccgcg	T→G	tcccctctcc	72.20	6.34	86.35	7.63	2.87	10^{-2}	C			
		rs1054264214:C	ctctctccta	G→C	tgagccgccg	21.27	1.59	15.40	1.14	6.14	10^{-6}	A	↑	according to a comprehensive pharmaceutical review [209]: enhancing GLIS3 activity may help patients with many GLIS3-insufficiency related diseases including glaucomas	▲
		rs1054264214:T	ctctctccta	G→T	tgagccgccg	21.27	1.59	4.94	0.49	23.60	10^{-6}	A			
		rs1188874844:A	ccccactctt	C→A	cgcgagcccg	41.20	3.27	18.23	1.63	13.64	10^{-6}	A			
		rs1204510797:A	ctcctagaca	C→A	ccgccggctc	21.27	1.59	15.28	1.21	6.09	10^{-6}	A			
		rs1318056914:G	cggctctctc	C→G	gagtgagccg	21.27	1.59	14.84	1.46	5.82	10^{-6}	A			
		rs1318056914:T	cggctctctc	C→T	gagtgagccg	21.27	1.59	11.68	0.98	10.64	10^{-6}	A			
		rs1324223594:T	cgcgcacggg	C→T	ccagagcgcg	72.20	6.34	60.41	6.52	2.56	0.05	D			
		rs1381426684:A	ggttttcatt	T→A	gtttggatgg	22.02	1.57	13.77	0.97	9.35	10^{-6}	A			
		rs1381426684:G	ggttttcatt	T→G	gtttggatgg	22.02	1.57	18.84	1.32	3.11	10^{-2}	C			
		rs1395914378:A	cccccgtttg	G→A	cgcgctccgt	22.02	1.57	18.38	1.46	3.38	10^{-3}	B			
		rs1419918714:A	ggcaccggcg	G→A	cggcgcgcac	72.20	6.34	50.11	3.94	6.20	10^{-6}	A			
		rs1462747355:A	ctctcctaga	C→A	agccgccggc	21.27	1.59	14.14	1.22	7.15	10^{-6}	A			
		rs1462747355:T	ctctcctaga	C→T	agccgccggc	21.27	1.59	7.26	0.82	15.91	10^{-6}	A			
		rs1468490721:T	ctcacacatc	C→T	ccttcctgag	7.53	0.65	5.55	0.48	5.01	10^{-6}	A			
		rs1587360250:A	agacacacac	C→A	ggctctctcc	21.27	1.59	17.93	1.26	3.33	10^{-3}	B			
		rs1587364866:A	cccccgttt	G→A	ccgcgctccg	22.02	1.57	10.92	1.36	9.79	10^{-6}	A			
		rs1816804344:A	tagacacaca	C→A	cggctctctc	21.27	1.59	18.23	1.51	2.77	10^{-2}	C			
		rs1816811786:A	gtcgctctct	G→A	agtcggcccg	21.27	1.59	15.27	1.39	5.64	10^{-6}	A			
		rs1816814452:T	gtgtattttg	A→T	aatgaccctg	21.27	1.59	11.98	0.96	10.51	10^{-6}	A			
		rs1816935145:A	gttttcattt	C→A	tttggatggg	22.02	1.57	10.56	0.69	15.14	10^{-6}	A			
		rs1816935297:C	gggttttcat	T→C	cgtttggatg	22.02	1.57	19.21	1.38	2.70	10^{-2}	C			
		rs1816939933:T	gcacacacgg	C→T	cagacgcgcg	22.02	1.57	13.12	1.21	8.89	10^{-6}	A			
		rs1816985377:T	cgcgtccaca	C→T	ctccctgcag	72.20	6.34	45.85	3.58	7.73	10^{-6}	A			
		rs1816996830:T	gctggctaca	G→T	ctcaggaagc	41.20	3.27	19.06	1.95	11.9	10^{-6}	A			
		rs1816997002:G	aagcagctgg	C→G	tcacactcag	41.20	3.27	29.31	3.22	5.03	10^{-6}	A			
		rs1834126099:G	gcttaaaaac	C→G	ttgtagtatt	6.99	0.72	5.73	0.41	3.16	10^{-2}	C			
		rs1834330590:A	agtcattgtt	T→A	cccttgaagg	14.72	1.69	11.72	0.93	3.27	10^{-2}	C			
		rs1834735750:A	gctcacacat	C→A	tccttcctga	7.53	0.65	5.11	0.48	6.08	10^{-6}	A			
		rs1834738475:G	cttctgattt	C→G	gttcccagtc	8.22	0.81	5.48	0.53	5.87	10^{-6}	A			
		rs761083460:A	catcctttat	G→A	tgaggctcac	7.53	0.65	2.29	0.23	18.00	10^{-6}	A			
		rs901338501:T	ccggctctct	C→T	tgagtgagcc	21.27	1.59	11.04	0.89	11.97	10^{-6}	A			
		rs903360862:T	ttttcatttc	C→T	ttggatgggg	22.02	1.57	17.59	1.27	4.42	10^{-3}	B			
		rs921563609:A	ctaacaagga	G→A	aaaaacctta	6.99	0.72	4.45	0.45	6.19	10^{-6}	A			
		rs935144019:A	ggaagcagct	G→A	gctcacactc	41.20	3.27	19.43	2.12	11.15	10^{-6}	A			
		rs960662613:A	ccctgcagcc	G→A	ccttcccctc	72.20	6.34	63.59	4.68	2.22	0.05	D			
		rs969234038:T	gtccacactg	C→T	cctgcagccg	72.20	6.34	45.62	2.77	8.60	10^{-6}	A			
		rs986276590:A	cccctccggc	G→A	tcttccccct	41.20	3.27	24.35	2.08	9.03	10^{-6}	A			
		rs986276590:T	cccctccggc	G→T	tcttccccct	41.20	3.27	28.76	2.31	6.37	10^{-6}	A			

Table S3. Cont.

Human Gene		Candidate SNP marker				K$_D$, nM, *in silico*				Significance				Effect of changes in human gene expression on the development of primary open-angle glaucoma (POAG, ☼: "▼" aggravation, "▲" alleviation) [Reference]	☼ ▲ ▼
#	*NCBI Gene Symbol* (NCBI Gene ID)	dbSNP ID:min [437]	5′ flank, 10 bp	WT →min	3 flank, 10 bp	WT MEAN	± SEM	min MEAN	± SEM	Z	p	ϱ	Δ		
58	*GMDS* (2762)	rs1279990704:A	ctgacgcgaa	T→A	agcgtcgccg	66.02	4.85	96.03	6.81	7.34	10^{-6}	A		within human POAG models using zebrafish [210]: Gmds deficit can reduce fucosylation that may lead to defects in neuronal differentiation and maintenance	▼
		rs1781841839:G	gacgcgaatg	A→G	cgtcgccggc	66.02	4.85	111.12	9.07	9.48	10^{-6}	A	↓		
		rs1781842061:G	gctgacgcga	A→G	cagcgtcgcc	66.02	4.85	111.12	9.07	9.48	10^{-6}	A			
		rs1249302689:A	ccggctgacg	C→A	ctacagcgtc	66.02	4.85	56.23	4.19	3.07	10^{-2}	C		within a biomedical cohort-based study [211]: GMDS excess is a biomedical molecular marker for carcinogenesis, which is comorbid to POAG at least in their commonly accepted general sensitivity to ultraviolet radiation-inducible oxidative stress [212]	
		rs1289148349:T	gacagtaaag	C→T	ttcagcctct	10.77	0.78	6.09	0.67	8.62	10^{-6}	A			
		rs1450662342:A	cggctgacgc	G→A	tacagcgtcg	66.02	4.85	41.01	2.84	9.43	10^{-6}	A	↑		
		rs1581752469:A	cctcttgaca	G→A	gctgtcttca	10.77	0.78	9.53	0.81	2.19	0.05	D			
		rs1781841241:T	ctgcgccagt	C→T	cgcgaatgac	66.02	4.85	29.42	2.77	13.54	10^{-6}	A			
59	*GPX1* (2876)	rs1176414550:C	gccggccagt	T→C	cgtgacccgc	6.93	0.52	25.11	1.64	25.83	10^{-6}	A		within human disease model using elderly mice subjected to artificial weakening of neuroprotective abilities [213]: in POAG, Gpx1 deficit may be a biomedical molecular marker for both reduced retinal ganglion cell survival and retinal ganglion cell loss in the elderly because of their hypersensitivity to oxidative stress	▼
		rs1176414550:G	gccggccagt	T→G	cgtgacccgc	6.93	0.52	30.20	2.10	28.72	10^{-6}	A			
		rs1401819530:C	cgccggccag	T→C	acgtgacccg	6.93	0.52	17.80	1.39	17.39	10^{-6}	A	↓		
		rs1405579466:C	gccgccggcc	A→C	ccacgtgacc	6.93	0.52	8.29	0.61	3.39	10^{-3}	B			
		rs1160031325:A	acccgccgcc	G→A	tgtgccacgt	6.93	0.52	6.19	0.51	2.03	0.05	D		according to a comprehensive molecular medicine review [214]: medication-derived Gpx1 upregulation might open potential future therapies to harness the beneficial effects of this major ubiquitous antioxidant enzyme, which must counteract the inevitable damaging effects of free radicals on the optic nerve head when perceiving light flux as a vital function of the eyes in line with nonhuman primate POAG models using male juvenile cynomolgus monkeys (Macaca fascicularis) treated with an argon laser to scar the trabecular meshwork in one eye until an intraocular pressure increased indicating glaucomatous eye [161]	▲
		rs1291918247:A	cctcaggccc	G→A	tgaggcggga	45.94	4.03	24.86	1.77	10.88	10^{-6}	A			
		rs1349514546:A	tgtgccacgt	G→A	ggaaaactgc	45.94	4.03	14.33	1.62	16.26	10^{-6}	A			
		rs1361398637:A	ggggccggat	G→A	cgggtagggc	45.94	4.03	26.82	2.39	8.62	10^{-6}	A			
		rs1439316786:A	cctgtgccac	G→A	ccggaaaact	45.94	4.03	28.27	2.34	8.05	10^{-6}	A			
		rs1800668:T	cctcccctta	C→T	gcgcctgctg	6.93	0.52	3.59	0.35	10.72	10^{-6}	A	↑		
		rs2047893069:T	cgccgccggc	C→T	gccacgtgac	6.93	0.52	4.50	0.35	7.93	10^{-6}	A			
		rs2047893526:A	ccggaaaact	G→A	gaccctcagg	45.94	4.03	17.49	2.66	10.99	10^{-6}	A			
		rs2047895221:A	cgagccctcc	G→A	tgaggagggg	45.94	4.03	39.47	3.78	2.34	0.05	D			
		rs899533338:T	ccggccagtt	A→T	gtgacccgcc	6.93	0.52	5.31	0.47	4.59	10^{-3}	B			
		rs996535868:A	tgccacgtga	C→A	aaaactgcct	45.94	4.03	36.15	2.87	4.05	10^{-3}	B			
60	*GRIN2B* (2904)	rs1197799406:C	ccacctcttt	T→C	ctctccaccc	23.01	1.57	27.03	2.05	3.17	10^{-2}	C		within human neurodegenerative disease cellular models using human induced pluripotent stem cells carrying either GRIN2B-deletion or GRIN2B loss-of-function mutation [215]: impaired both calcium influx and membrane depolarization that can aggravate POAG [216]	▼
		rs1350017362:C	tccacccccc	A→C	gctcctgcct	23.01	1.57	25.95	2.21	2.21	0.05	D			
		rs1366930470:C	accccccacc	T→C	cctgcctctc	23.01	1.57	28.53	1.95	4.46	10^{-3}	B	↓		
		rs1591652529:C	cccccacctc	T→C	tgcctctcca	23.01	1.57	31.84	2.24	6.63	10^{-6}	A			
		rs1863148966:A	ggggtggggg	G→A	gggggtgggg	37.31	2.68	43.50	3.21	2.98	10^{-2}	C			
		rs1024745770:T	ggagaaagag	G→T	gtgggggtg	37.31	2.68	25.32	2.81	5.86	10^{-6}	A		within human disease models using mice [217]: Grin2b excess can enhance glutamatergic signaling, which can elevate excitotoxicity accelerating neurodegeneration in POAG	
		rs1197799406:G	ccacctcttt	T→G	ctctccaccc	23.01	1.57	16.42	1.29	6.47	10^{-6}	A			
		rs1213509668:T	ggccttcttt	G→T	gtgtcagtgt	26.41	2.61	19.00	1.33	5.44	10^{-6}	A			
		rs1300539525:G	caccccccac	C→G	tcctgcctct	23.01	1.57	16.40	1.24	6.65	10^{-6}	A			
		rs1414806408:A	tggggggtgt	G→A	gtggggtggg	25.37	2.94	13.74	1.17	8.53	10^{-6}	A			
		rs1429450386:G	gaaagaggac	A→G	gggggtgtgg	37.31	2.68	33.30	2.35	2.26	0.05	D			
		rs1555110824:T	gaccacctcc	A→T	aacccaaagg	26.41	2.61	23.09	2.11	1.99	0.05	D			
		rs1555110831:T	cgtacctgtg	C→T	tttaacaact	26.41	2.61	13.65	1.60	8.61	10^{-6}	A	↑		
		rs1863147997:T	ggagcagaag	C→T	acccgcccct	37.31	2.68	9.52	0.91	22.9	10^{-6}	A			
		rs1863148556:A	ggacacgcgc	G→A	gtggagaaag	37.31	2.68	32.38	2.74	2.55	0.05	D			
		rs375572662:T	ctccaccccc	C→T	ggctcctgcc	23.01	1.57	17.38	1.25	5.66	10^{-6}	A			
		rs748829220:T	cctccaagat	C→T	aaaggggacc	26.41	2.61	15.74	1.14	8.44	10^{-6}	A			
		rs767875110:A	ttgctgtcat	C→A	gtgggccttc	26.41	2.61	11.14	1.00	12.9	10^{-6}	A			
		rs797044930:T	gccttctttg	C→T	tgtcagtgtg	26.41	2.61	17.92	1.12	6.62	10^{-6}	A			
		rs998632225:T	cccctgggag	C→T	gctcacaccc	37.31	2.68	23.38	2.13	8.07	10^{-6}	A			

Table S3. Cont.

#	Human Gene: *NCBI Gene Symbol* (NCBI Gene ID)	Candidate SNP marker: dbSNP ID:min [437]	5′ flank, 10 bp	WT →min	3 flank, 10 bp	K_D, nM, *in silico*: WT MEAN	± SEM	min MEAN	± SEM	Significance: Z	p	ϱ	Δ	Effect of changes in human gene expression on the development of primary open-angle glaucoma (POAG, ☼: "▼" aggravation, "▲" alleviation) [Reference]	☼ ▲ ▼
61	*GSTM1* (2944)	rs1314884398:C	ggagctctta	T→C	actctgagcc	2.50	0.27	8.23	0.79	16.68	10^{-6}	A	↓	within an exhaustive meta-analysis of the occurrence of single-nucleotide polymorphisms (SNPs) in human subpopulations with different regional and ethnic specificities [218]: GSTM1-null genotypes are associated with increased POAG risk in Asian populations	▼
		rs142392008:A	agctcttata	C→A	tctgagccct	2.50	0.27	3.86	0.41	5.81	10^{-6}	A			
		rs1294403899:A	ctttcgggtt	G→A	tggcgggccg	70.81	4.68	25.71	2.05	19.55	10^{-6}	A	↑	within human disease model using rats subjected with corneal wounds, which was then regularly irradiated with blue light to slow down its healing and, at the same time, treated orally with the Peucedanum japonicum Thunb. leaf extract to repair these wounds [219]: extract-dose-dependent acceleration in wound healing due to Gstm1-upregulation that can alleviate POAG that can improve post-surgery recovery in POAG	▲
		rs1326634227:A	tcccgggcct	C→A	cagaatggcg	70.81	4.68	16.68	1.62	24.60	10^{-6}	A			
		rs1326634227:G	tcccgggcct	C→G	cagaatggcg	70.81	4.68	63.48	4.66	2.21	0.05	D			
		rs1441715196:A	gcctccagaa	T→A	ggcgcctttc	70.81	4.68	41.84	3.07	10.66	10^{-6}	A			
		rs1647984645:A	ctcccgggcc	T→A	ccagaatggc	70.81	4.68	52.85	3.78	6.01	10^{-6}	A			
		rs1647985730:G	tggcgccttt	C→G	gggttgtggc	70.81	4.68	62.22	4.55	2.62	10^{-2}	C			
62	*GSTO1* (9446)	rs1013027073:C	caagattaaa	T→C	atagggtaga	4.30	0.25	6.43	0.56	7.68	10^{-6}	A	↓	within human disease models using Gsto1-knockout mice subjected with an artificial photo-oxidative damage of retina [220]: decreased photoreceptor cell death, inflammation and oxidative stress as well as improved retinal function	▲
		rs1055504352:G	atgactgagc	A→G	tttataactt	2.27	0.22	4.56	0.71	7.69	10^{-6}	A			
		rs1314766886:G	tgcgctgcgc	C→G	acgatgtccg	53.46	3.75	58.90	3.94	2.00	0.05	D			
		rs1369565112:G	ctgcgccacg	A→G	tgtccgggga	53.46	3.75	103.58	8.11	12.59	10^{-6}	A			
		rs1383161386:G	aagattaaat	A→G	tagggtagag	4.30	0.25	5.54	0.42	5.28	10^{-6}	A			
		rs2091590079:G	acaagattaa	A→G	tatagggtag	4.30	0.25	5.24	0.38	4.27	10^{-3}	B			
		rs2091592259:A	ggatgactga	G→A	catttataac	2.27	0.22	2.61	0.26	2.04	0.05	D			
		rs2091592273:A	gatgactgag	C→A	atttataact	2.27	0.22	3.10	0.32	4.46	10^{-3}	B			
		rs2091592297:C	tgactgagca	T→C	ttataacttt	2.27	0.22	3.36	0.33	5.72	10^{-6}	A			
		rs2091592308:G	ctgagcattt	A→G	taacttttgt	2.27	0.22	5.90	0.55	14.38	10^{-6}	A			
		rs573809437:T	gcatttataa	C→T	ttttgtgtat	2.27	0.22	2.76	0.30	2.71	10^{-2}	C			
		rs751870821:C	tctctgggcc	G→C	taatcgcctt	28.05	2.56	31.78	2.42	2.10	0.05	D			
		rs768426683:C	tgcgccacga	T→C	gtccggggag	53.46	3.75	68.42	5.34	4.71	10^{-3}	B			
		rs768426683:G	tgcgccacga	T→G	gtccggggag	53.46	3.75	82.33	5.85	8.65	10^{-6}	A			
		rs984173238:C	gggcaggcac	T→C	tttgagctaa	16.23	1.23	22.01	1.80	5.46	10^{-6}	A			
		rs1003018292:G	gccacgatgt	C→G	cggggagtca	53.46	3.75	44.02	3.11	3.90	10^{-3}	V	↑	within human disease models using mice subjected with an artificial acute hypoglycemia [221]: Gsto1-upregulation together with accelerated retinal cell death as a POAG symptom [222]	▼
		rs1263636441:T	cacctctctg	G→T	gccgtaatcg	28.05	2.56	23.54	2.81	2.34	0.05	D			
		rs1295340987:A	cacctacttc	C→A	tgaatcccct	16.23	1.23	10.22	0.90	7.95	10^{-6}	A			
		rs1589843010:A	ggtgggatac	G→A	gggggtctcg	28.05	2.56	19.91	2.01	5.04	10^{-6}	A			
		rs2091593562:T	gacgcgccac	C→T	tacttcctga	16.23	1.23	12.30	1.16	4.58	10^{-3}	B			
		rs2091594169:T	gatgtccggg	G→T	agtcagccag	53.46	3.75	37.33	3.82	5.79	10^{-6}	A			
		rs2091595699:A	gggggcggtg	G→A	gatacggggg	28.05	2.56	23.62	2.61	2.40	0.05	D			
		rs2091595758:T	cggtgggata	C→T	gggggtctc	28.05	2.56	22.00	2.00	3.77	10^{-3}	B			
		rs541811664:A	ggtcggagct	G→A	cagtgggacg	28.05	2.56	22.54	2.11	3.35	10^{-6}	A			
		rs747892529:A	ccacgatgtc	C→A	ggggagtcag	53.46	3.75	30.90	2.43	10.41	10^{-6}	A			
		rs747892529:G	ccacgatgtc	C→G	ggggagtcag	53.46	3.75	45.25	3.89	3.01	10^{-2}	C			
		rs747892529:T	ccacgatgtc	C→T	ggggagtcag	53.46	3.75	29.05	2.52	10.95	10^{-6}	A			
		rs768426683:A	tgcgccacga	T→A	gtccggggag	53.46	3.75	40.73	2.96	5.39	10^{-6}	A			
		rs772396594:A	cacgatgtcc	G→A	gggagtcagc	53.46	3.75	44.95	3.28	3.43	10^{-3}	B			
63	*GSTO2* (119391)	rs1016399495:C	aaaagccaca	T→C	gcagcactgc	47.65	3.49	72.78	4.87	8.54	10^{-6}	A	↓	within human disease models using neuron-specific Gsto2-knockdown Drosophila line [223]: decreased the solubility of proteins homologous to human FUS in neurons in an age-dependent manner that can contribute to neurodegenerative proteinopathies, such as amyotrophic lateral sclerosis and dementia as well as POAG [224]	▼
		rs1247520584:C	gacttcacaa	A→C	ggcctcctgc	23.07	1.79	34.07	2.74	6.97	10^{-6}	A			
		rs1589856153:T	agccacatgc	A→T	gcactgccct	47.65	3.49	63.49	4.23	5.80	10^{-6}	A			
		rs2011242050:T	agccgactga	C→T	ttcacaaagg	23.07	1.79	25.99	2.03	2.17	0.05	D			
		rs2011563340:G	aagaaaagcc	A→G	catgcagcac	47.65	3.49	72.78	4.87	8.54	10^{-6}	A			
		rs2011563463:G	gaaaagccac	A→G	tgcagcactg	47.65	3.49	72.78	4.87	8.54	10^{-6}	A			

Table S3. Cont.

Human Gene		Candidate SNP marker				K				Significance					
#	*NCBI Gene Symbol* (NCBI Gene ID)	dbSNP ID:min [437]	5′ flank, 10 bp	WT →min	3 flank, 10 bp	WT MEAN	± SEM	min MEAN	± SEM	Z	p	ϱ	Δ	Effect of changes in human gene expression on the development of primary open-angle glaucoma (POAG, ☼: "▼" aggravation, "▲" alleviation) [Reference]	☼ ▲ ▼
		rs1172951544:A	aaagccacat	G→A	cagcactgcc	47.65	3.49	9.10	1.13	22.94	10^{-6}	A			
		rs1255434595:A	ggctagttgg	C→A	gggtaggatc	43.64	4.45	31.96	3.19	4.37	10^{-3}	B			
		rs1332211632:T	gggcaggccc	C→T	gtctaggccc	43.64	4.45	27.39	3.50	5.70	10^{-6}	A			
		rs1421156293:T	ggggcaggcc	C→T	cgtctaggcc	43.64	4.45	32.00	3.46	4.18	10^{-3}	B			
		rs1458996952:A	ttggcgggta	G→A	gatcacgtgc	43.64	4.45	30.53	2.17	5.75	10^{-6}	A			
		rs1458996952:C	ttggcgggta	G→C	gatcacgtgc	43.64	4.45	31.09	2.39	5.31	10^{-6}	A			
		rs1476458804:T	gcactgccct	C→T	tctgggactt	47.65	3.49	41.75	2.97	2.59	10^{-2}	C			
		rs2011253789:A	tgggcgccgg	G→A	gctagttggc	43.64	4.45	35.56	3.63	2.84	10^{-2}	C			
		rs2011254570:T	ggtaggatca	C→T	gtgcgagggg	43.64	4.45	23.03	1.78	10.00	10^{-6}	A			
		rs2011254654:T	gtaggatcac	G→T	tgcgagggc	43.64	4.45	36.97	2.98	2.56	0.05	D			
		rs2011564374:A	tgccctctct	G→A	ggacttggga	47.65	3.49	25.86	2.27	10.69	10^{-6}	A			
63	*GSTO2* (119391)	rs2011565430:T	ctcccacagc	C→T	atcttgggat	47.65	3.49	25.44	1.81	12.28	10^{-6}	A	↑	within human disease models using Gsto2-overexpressing Drosophila line [225]: improved both locomotive activities and neuromuscular junctions that can prevent neurodegenerative proteinopathies, such as amyotrophic lateral sclerosis and dementia as well as POAG [224]	▲
		rs376014202:A	actgacttca	C→A	aaaggcctcc	23.07	1.79	13.05	0.84	11.30	10^{-6}	A			
		rs376014202:T	actgacttca	C→T	aaaggcctcc	23.07	1.79	9.15	0.74	16.54	10^{-6}	A			
		rs780411004:A	aggatcacgt	G→A	cgaggggcag	43.64	4.45	13.88	1.84	13.68	10^{-6}	A			
		rs909881072:T	ggcaggcccc	G→T	tctaggcccc	43.64	4.45	29.51	3.05	5.39	10^{-6}	A			
		rs923193687:G	aagccacatg	C→G	agcactgccc	47.65	3.49	40.10	3.11	3.24	10^{-2}	C			
		rs923193687:T	aagccacatg	C→T	agcactgccc	47.65	3.49	8.21	0.95	25.72	10^{-6}	A			
		rs932699673:A	cgggtaggat	C→A	acgtgcgagg	43.64	4.45	10.06	0.94	21.19	10^{-6}	A			
		rs932699673:T	cgggtaggat	C→T	acgtgcgagg	43.64	4.45	15.21	1.58	14.48	10^{-6}	A			
		rs956054001:C	tcctgaagaa	A→C	agccacatgc	47.65	3.49	38.47	2.97	4.02	10^{-3}	B			
		rs977297354:A	gccgggggcta	G→A	ttggcgggta	43.64	4.45	35.64	3.13	3.01	10^{-2}	C			
		rs1409927950:A	gctcgcccat	C→A	ccccgctcct	54.99	5.27	44.23	4.45	3.13	10^{-2}	C			
		rs1028909908:G	gggaccctcc	A→G	gaagagcggc	55.67	4.33	148.55	10.30	18.84	10^{-6}	A			
		rs1220761444:G	gaccaccctt	A→G	taaggctcgg	4.77	0.58	15.01	1.43	14.86	10^{-6}	A			
		rs1250736317:G	ggatgataca	T→G	ggtggtgtct	12.31	1.07	17.63	1.41	6.09	10^{-6}	A			
		rs1459781916:G	aggatgatac	A→G	tggtggtgtc	12.31	1.07	15.89	1.59	3.86	10^{-3}	B			
		rs1867415754:G	cctccagaag	A→G	gcggccggcg	55.67	4.33	83.97	6.95	7.24	10^{-6}	A			
		rs1867417021:G	caccctttata	A→G	ggctcggagg	4.77	0.58	6.66	0.76	4.01	10^{-3}	B	↓	within a biomedical cohort-based proteomic study [226]: GSTP1 downregulation in aqueous humour is a biomedical molecular marker for POAG	▼
		rs71534226:G	gtaggatgat	A→G	catggtggtg	12.31	1.07	28.84	2.12	14.98	10^{-6}	A			
		rs763929334:A	ggaccaccct	T→A	ataaggctcg	4.77	0.58	9.49	0.86	9.09	10^{-6}	A			
		rs763929334:G	ggaccaccct	T→G	ataaggctcg	4.77	0.58	20.06	1.82	18.98	10^{-6}	A			
		rs774297523:C	accaccctta	T→C	aaggctcgga	4.77	0.58	13.92	1.31	13.96	10^{-6}	A			
		rs889185294:C	accctccaga	A→C	gagcggccgg	55.67	4.33	106.96	7.48	12.49	10^{-6}	A			
64	*GSTP1* (2950)	rs1051824512:T	tccgcgggac	C→T	ctccagaaga	55.67	4.33	40.91	2.95	5.81	10^{-6}	A			
		rs1194648427:T	ggctgggagg	G→T	atgagagtag	12.31	1.07	10.75	1.05	2.07	0.05	D			
		rs1233936065:T	cccttataag	G→T	ctcggaggcc	4.77	0.58	3.55	0.46	3.30	10^{-3}	B			
		rs1317401958:A	acccttataa	G→A	gctcggaggc	4.77	0.58	2.97	0.32	5.83	10^{-6}	A			
		rs1351989235:A	taggatgata	C→A	atggtggtgt	12.31	1.07	7.33	0.66	8.30	10^{-6}	A			
		rs1351989235:T	taggatgata	C→T	atggtggtgt	12.31	1.07	4.23	0.45	15.49	10^{-6}	A			
		rs1867414674:A	cgcccggggt	G→A	cagcggccgc	55.67	4.33	40.09	4.23	5.01	10^{-6}	A	↑	within a phytopharmacological dietary wolfberry study [227]: GSTP1 upregulation can improve retinal mitochondrial biogenesis that may alleviate POAG	▲
		rs369775780:A	atgatacatg	G→A	tggtgtctgg	12.31	1.07	10.00	0.97	3.19	10^{-2}	C			
		rs369775780:T	atgatacatg	G→T	tggtgtctgg	12.31	1.07	8.45	0.88	5.54	10^{-6}	A			
		rs755205239:C	gagagtagga	T→C	gatacatggt	12.31	1.07	9.14	0.90	4.54	10^{-3}	B			
		rs758809730:A	gatgatacat	G→A	gtggtgtctg	12.31	1.07	3.83	0.56	13.82	10^{-6}	A			
		rs762723269:T	cgggaccacc	C→T	ttataaggct	4.77	0.58	3.20	0.39	4.60	10^{-3}	B			
		rs996068510:T	ccgcgggacc	C→T	tccagaagag	55.67	4.33	40.73	2.92	5.91	10^{-6}	A			

Table S3. Cont.

#	Human Gene: *NCBI Gene Symbol* (NCBI Gene ID)	Candidate SNP marker: dbSNP ID:min [437]	5′ flank, 10 bp	WT→min	3 flank, 10 bp	K_D, nM, *in silico*: WT MEAN	± SEM	min MEAN	± SEM	Significance: Z	p	ϱ	Δ	Effect of changes in human gene expression on the development of primary open-angle glaucoma (POAG, ☼: "▼" aggravation, "▲" alleviation) [Reference]	☼ ▲ ▼
65	*GSTT1* (2952)	none SNP markers were found to significantly change the K_D-values in the proximal promoters of this human gene using the knowledge base Human_SNP_TATAdb [49], as accessed on March 20, 2024													
66	*HAMP* (57817)	rs1474900660:G	aagtttttgt	A→G	tagatggggt	2.75	0.40	7.48	0.68	11.71	10^{-6}	A		within human POAG cellular models using human the trabecular meshwork cells cultivated with HAMP-antagonists [231]: beneficial therapeutic effects in POAG	▲
		rs2066308148:G	aggccccata	A→G	aagcgactgt	6.98	0.59	16.48	1.57	13.47	10^{-6}	A	↓		
		rs2066308155:C	ggccccataa	A→C	agcgactgtc	6.98	0.59	12.24	1.15	8.86	10^{-6}	A			
		rs2066308133:T	cctggcaggc	C→T	ccataaaagc	6.98	0.59	6.07	0.52	2.32	0.05	D	↑	within a biomedical cohort-based study [232]: HAMP excess in the serum and aqueous humor of patients is a biomedical molecular marker for POAG	▼
		rs367646034:T	cccataaaag	C→T	gactgtcact	6.98	0.59	5.94	0.51	2.65	10^{-2}	C			
67	*HDAC6* (10013)	rs1165157031:C	tcaccacgcc	T→C	atttggccg	16.62	1.83	46.06	4.29	14.13	10^{-6}	A		within human disease models using rats subjected with an artificial retinal ischaemia/reperfusion injury [233]: HDAC6-inhibitor tubacin elevated survival of the retinal ganglion cell in POAG	▲
		rs1387218098:C	cccgccgctt	G→C	tgagctcgcg	49.81	3.74	66.87	4.76	5.69	10^{-6}	A			
		rs1420235592:T	cggtgagtac	A→T	gcgcgtcgac	30.74	2.98	37.05	3.16	2.89	10^{-6}	A			
		rs1422293290:C	ccacgcctat	T→C	ttggccgcca	16.62	1.83	25.45	2.19	6.09	10^{-6}	A			
		rs1425526769:C	gccccgccgc	T→C	tgtgagctcg	49.81	3.74	72.96	5.59	7.11	10^{-6}	A			
		rs1602226382:C	acgcctattt	T→C	ggccgccacc	16.62	1.83	21.25	2.22	3.23	10^{-6}	A			
		rs2062723198:C	accacgccta	T→C	tttggccgcc	16.62	1.83	23.04	2.99	3.84	10^{-6}	A	↓		
		rs2062727823:C	ggcgcctgcc	T→C	tttacgtagg	4.49	0.44	5.69	0.58	3.36	10^{-6}	A			
		rs2062731471:C	gagtttgaga	A→C	aggggctgcg	23.38	1.78	29.30	2.28	4.15	10^{-6}	A			
		rs2062733697:T	ggttagcgaa	A→T	cgttagcggt	37.79	2.98	54.86	4.97	6.20	10^{-6}	A			
		rs2062741935:C	cggggccggg	T→C	agaatggcca	21.11	2.12	43.11	3.23	11.41	10^{-6}	A			
		rs782373936:G	ccggcccgat	A→G	aggggtggag	24.80	2.24	31.57	2.35	4.12	10^{-6}	A			
		rs782426162:G	cactagcccc	C→G	tcacataccc	8.86	0.86	10.80	1.15	2.74	10^{-6}	A			
		rs1162889836:A	tgagtggagc	G→A	gtgagtacag	30.74	2.98	24.42	1.75	3.82	10^{-6}	A		according to comprehensive review [234]: HDAC6 excess can improve microtubule dynamics beneficial for axonal growth, as well as supervise and coordinate stress response including stress granule formation, chaperone upregulation, inflammatory tolerance increase and facilitates the autophagy of both misfolded proteins and protein misaggregates leading to neurodegeneration, that altogether can alleviate POAG	
		rs1292007317:C	gggttagcga	A→C	acgttagcgg	37.79	2.98	25.31	2.20	6.84	10^{-6}	A			
		rs1297954912:T	cacaggtcgc	C→T	agaaacttgg	23.38	1.78	19.28	1.60	3.42	10^{-6}	A			
		rs1319946793:A	gcggagtttg	G→A	aaggctgtgg	21.11	2.12	16.34	1.21	4.11	10^{-6}	A			
		rs1374290603:T	ccggtccggc	C→T	cgataagggg	24.80	2.24	20.20	1.71	3.32	10^{-6}	A			
		rs1436025484:A	gttagcgaaa	C→A	gttagcggtc	37.79	2.98	27.37	1.81	6.27	10^{-6}	A			
		rs1436025484:T	gttagcgaaa	C→T	gttagcggtc	37.79	2.98	24.28	1.80	8.18	10^{-6}	A			
		rs1444683380:A	cccgataagg	G→A	gtggagttaa	24.80	2.24	20.87	1.97	2.64	10^{-6}	A			
		rs1557022229:T	ctgcctttta	C→T	gtaggcgcag	4.49	0.44	2.45	0.24	8.79	10^{-6}	A			
		rs1557022530:A	ggggcgggat	C→A	tggcggagtg	31.57	2.35	25.88	2.47	3.28	10^{-6}	A			
		rs17281216:T	agagggttag	C→T	gaaacgttag	37.79	2.98	14.82	1.63	13.85	10^{-6}	A	↑		
		rs2062730029:A	cgcgagaggt	G→A	tggctcgcgc	49.81	3.74	28.08	3.39	8.07	10^{-6}	A			
		rs2062731852:A	gtggagcggt	G→A	agtacagcgc	30.74	2.98	8.91	0.80	18.78	10^{-6}	A			
		rs2062733319:A	gctagggact	G→A	gctgaagagg	37.79	2.98	27.24	2.17	5.83	10^{-6}	A			
		rs2062735108:A	tggttgaaac	G→A	ctagggcgg	31.57	2.35	14.91	1.48	12.09	10^{-6}	A			
		rs2062735334:T	ctagggcgg	G→T	atctggcgga	31.57	2.35	26.65	3.21	2.39	10^{-6}	A			
		rs2062736066:T	gcccgataag	G→T	ggtggagtta	24.80	2.24	10.13	1.00	13.40	10^{-6}	A			
		rs782564776:A	tggctgaaga	G→A	ggttagcgaa	37.79	2.98	30.08	2.77	3.76	10^{-6}	A			
		rs902921020:A	ctggttgaag	G→A	aacggggcag	37.79	2.98	31.66	2.36	3.26	10^{-6}	A			
		rs919312765:T	ggccttgagg	C→T	acggtcccct	49.81	3.74	38.47	3.63	4.29	10^{-6}	A			
		rs982230450:A	gggcggggcc	G→A	ggtagaatgg	21.11	2.12	15.81	1.61	4.04	10^{-6}	A			
68	*HES1* (3280)	rs921184111:G	ccagatatat	A→G	tagaggccgc	0.69	0.11	1.47	0.19	7.56	10^{-6}	A		within human POAG cellular models using primary human trabecular meshwork cells subjected with oxidative stress along with a transfection of plasmid carrying shRNA against human HES1 gene [235]: rescued POAG-related defects	▲
		rs931936858:C	cagatatata	T→C	agaggccgcc	0.69	0.11	1.04	0.14	4.02	10^{-6}	A	↓		
		rs931936858:G	cagatatata	T→G	agaggccgcc	0.69	0.11	1.24	0.21	5.17	10^{-6}	A			

Table S3. Cont.

Human Gene		Candidate SNP marker				K_D, nM, *in silico*				Significance				Effect of changes in human gene expression on the development of primary open-angle glaucoma (POAG, ☼: "▼" aggravation, "▲" alleviation) [Reference]	☼ ▲ ▼
#	*NCBI Gene Symbol* (NCBI Gene ID)	dbSNP ID:min [437]	5′ flank, 10 bp	WT →min	3 flank, 10 bp	WT MEAN	± SEM	min MEAN	± SEM	Z	p	ϱ	Δ		
69	*HSPA4* (3308)	rs1179371472:A	gaacatcccg	T→A	tctttcgtag	10.61	1.03	12.70	1.21	2.65	10^{-2}	C	↓	within human diseases models using Hspa4-deficient mice [236]: enhanced apoptosis in inflammatory bowel disease comorbid with POAG [237]	▼
		rs1180439878:C	ccgttctttc	G→C	tagctctggt	10.61	1.03	19.70	1.93	8.96	10^{-6}	A			
		rs1236593053:A	acatcccgtt	C→A	tttcgtagct	10.61	1.03	13.65	1.27	3.74	10^{-3}	B			
		rs1765080922:C	cgttctttcg	T→C	agctctggtg	10.61	1.03	32.56	3.23	16.14	10^{-6}	A			
		rs937387156:T	gttctttcgt	A→T	gctctggtgc	10.61	1.03	30.86	2.19	17.71	10^{-6}	A			
		rs1026921290:T	cccgttcttt	C→T	gtagctctgg	10.61	1.03	4.94	0.49	11.01	10^{-6}	A	↑	within human disease cellular models using murine cells transfected with plasmid carrying an additional Hspa4 gene copy [238]: Hspa4 excess can protect cells against oxidative damage that may prevent an important pathogenetic step in POAG [239]	▲
		rs1054631128:T	tctttcgtag	C→T	tctggtgctg	10.61	1.03	8.09	0.77	3.97	10^{-3}	B			
		rs1180439878:A	ccgttctttc	G→A	tagctctggt	10.61	1.03	6.81	0.62	6.64	10^{-6}	A			
		rs529032580:C	ttctttcgta	G→C	ctctggtgct	10.61	1.03	9.10	0.91	2.19	0.05	D			
70	*HSPA5* (3309)	rs1178835270:G	gggggagggt	A→G	acgacggggc	1.07	0.12	3.61	0.39	15.23	10^{-6}	A	↓	within a pharmaceutical study on the dexamethasone side effects during anti-inflammatory treatment using human trabecular meshwork cells [240]: HSPA5 deficit can increase both outflow resistance and intraocular pressure that may aggravate POAG	▼
		rs1178835270:T	gggggagggt	A→T	acgacggggc	1.07	0.12	3.03	0.32	13.24	10^{-6}	A			
		rs979231959:G	gcctgggcca	A→G	ccagcttggt	42.79	3.24	60.59	4.15	6.81	10^{-6}	A			
		rs1414599242:A	ccagcttggt	G→A	atcggcggcg	42.79	3.24	26.38	2.88	7.29	10^{-6}	A	↑	within a pharmaceutical study using rats subjected with valproate [241]: Hspa5 upregulation, which can protect retina against ischemia-reperfusion injury and, thereby, retard POAG development	▲
		rs1832567307:T	gcagggcctt	C→T	gcctccaacg	42.79	3.24	21.80	2.64	9.44	10^{-6}	A			
		rs1832567518:T	gaacggcctc	C→T	cctgggccaa	42.79	3.24	20.55	2.29	10.90	10^{-6}	A			
		rs1832567691:A	ttggtggcct	G→A	cggcggccag	42.79	3.24	38.32	3.08	1.99	0.05	D			
		rs1832567890:T	cggccagctt	G→T	cgaatcggcg	42.79	3.24	24.96	1.90	10.04	10^{-6}	A			
		rs959944493:T	cggcggcctc	C→T	ccttcaccaa	42.79	3.24	29.70	3.95	4.76	10^{-3}	B			
71	*IGF1R* (3480)	rs1174230319:C	ccgctcgagt	G→C	tgcgagcggg	69.32	5.76	96.82	8.05	5.69	10^{-6}	A	↓	within human POAG cellular models using rat retinal ganglion cell line RGC-5 transfected with lentiviral vector carrying mircoRNA 100 (miR-100) targeted to Igf1r silencing under H2O2-caused oxidative stress [242]: Igf1r-deficit kept retinal ganglion cell against apoptosis	▲
		rs1280597345:G	tggccgctcg	A→G	gtgtgcgagc	69.32	5.76	97.99	8.32	5.83	10^{-6}	A			
		rs1375108105:A	gccgctcgag	T→A	gtgcgagcgg	69.32	5.76	110.43	9.84	7.64	10^{-6}	A			
		rs1375108105:C	gccgctcgag	T→C	gtgcgagcgg	69.32	5.76	110.43	9.84	7.64	10^{-6}	A			
		rs1375108105:G	gccgctcgag	T→G	gtgcgagcgg	69.32	5.76	110.43	9.84	7.64	10^{-6}	A			
		rs1168709511:A	gtggccgctc	G→A	agtgtgcgag	69.32	5.76	27.81	2.47	15.00	10^{-6}	A	↑	within human POAG cellular models using rat neonatal retinal ganglion cells [243]: etidronate upregulates Igf1r promoting neurite growth and retinal ganglion cell survival	
		rs1174230319:A	ccgctcgagt	G→A	tgcgagcggg	69.32	5.76	21.49	2.00	18.75	10^{-6}	A			
		rs1216904496:T	ggtggccgct	C→T	gagtgtgcga	69.32	5.76	41.09	2.68	9.90	10^{-6}	A			
		rs1310383556:T	aaggtcagca	C→T	aaaatacagg	9.06	0.80	4.31	0.34	12.51	10^{-6}	A			
		rs1323884112:A	ggcggtggcc	G→A	ctcgagtgtg	69.32	5.76	54.62	3.49	4.55	10^{-3}	B			
		rs1378249511:T	ctcgagtgtg	C→T	gagcgggcgc	69.32	5.76	20.05	1.70	20.92	10^{-6}	A			
		rs1490517310:T	gtcagcacaa	A→T	atacaggtca	9.06	0.80	3.74	0.52	10.72	10^{-6}	A			
		rs1596401431:A	tctagcagat	G→A	cagttaaaga	9.06	0.80	5.93	0.40	7.63	10^{-6}	A			
		rs2052240035:A	tcgagtgtgc	G→A	agcgggcgcg	69.32	5.76	40.84	3.27	9.17	10^{-6}	A			
		rs2052241253:A	gggcgcgtgt	G→A	cgcgggccag	69.32	5.76	29.93	3.97	10.73	10^{-6}	A			
		rs369728300:T	gcacaaaata	C→T	aggtcataaa	9.06	0.80	5.71	0.52	7.28	10^{-6}	A			
		rs928387601:A	gcgcgccgct	G→A	ctgcggcggt	69.32	5.76	58.42	4.94	2.89	10^{-2}	C			
72	*IL10* (3586)	rs1388972984:C	taagcacaag	G→C	tcctagtgag	25.15	1.62	32.27	1.96	5.64	10^{-6}	A	↓	within a biomedical cohort-based study [244]: valproic acid downregulated IL10 that can prevent surgery-induced conjunctival inflammation, which is a post-trabeculectomy complication in POAG [245]	▲
		rs1391692624:C	cctagtgagg	T→C	gttgcaggtt	25.15	1.62	32.27	1.96	5.64	10^{-6}	A			
		rs1462125860:G	ctagtgaggt	A→G	ttgcaggttt	25.15	1.62	32.27	1.96	5.64	10^{-6}	A			
		rs188056661:C	gagcttacaa	T→C	gaggcctccc	1.65	0.16	3.10	0.24	9.99	10^{-6}	A			
		rs1163046221:A	gtaagcacaa	G→A	ttcctagtga	25.15	1.62	15.65	1.20	9.49	10^{-6}	A	↑	within human POAG models using mice subjected with spinal cord injury, which downregulated Il10 according to a proper measurement in vivo, and, next, treated with nanoparticles of curcumin as a new promising natural phytomedication with neuroregenerative activity under moder nanopharmacutical study [246]: excess IL10 along with successful neuroregeneration, which has been confirmed through several independent behavioral tests, and may alleviate POAG according to a comprehensive biomedical review [247]	
		rs1327219745:A	aaggcaagat	G→A	gaggtaagca	25.15	1.62	22.69	1.83	2.00	0.05	D			
		rs1406029918:T	ttgtttcctt	C→T	gggaattggg	16.88	1.98	14.73	1.00	2.02	0.05	D			
		rs1407597040:T	aagcacaagg	C→T	cctagtgagg	25.15	1.62	21.21	1.66	3.36	10^{-3}	B			
		rs1674826380:A	ctcagcacat	C→A	gaagatgtta	16.88	1.98	8.05	1.03	8.51	10^{-6}	A			
		rs1674826756:A	aagatgttaa	C→A	ggagaagtgg	16.88	1.98	10.70	0.75	6.67	10^{-6}	A			
		rs1674828368:A	gggtttgttt	C→A	tgcagggaat	16.88	1.98	11.70	1.15	4.79	10^{-3}	B			
		rs1674884284:G	tgagcttaca	A→G	agaggcctcc	1.65	0.16	1.16	0.12	5.01	10^{-6}	A			
		rs534191384:A	tgtttccttc	G→A	ggaattgggt	16.88	1.98	12.23	0.82	4.78	10^{-3}	B			
		rs938054219:A	gtttcctagt	G→A	tagggttgca	25.15	1.62	20.50	1.68	3.92	10^{-3}	B			

Table S3. Cont.

#	Human Gene: *NCBI Gene Symbol* (NCBI Gene ID)	Candidate SNP marker: dbSNP ID:min [437]	5′ flank, 10 bp	WT →min	3 flank, 10 bp	K_D, nM, *in silico*: WT MEAN	± SEM	min MEAN	± SEM	Significance: Z	p	ρ	Δ	Effect of changes in human gene expression on the development of primary open-angle glaucoma (POAG, ☼: "▼" aggravation, "▲" alleviation) [Reference]	☼ ▲ ▼
73	*IL1A* (3552)	rs1681267950:T	aagacaatta	C→T	cacgcctact	6.00	0.53	2.19	0.22	15.26	10^{-6}	A	↑	within human POAG cellular models using human trabecular meshwork cell line TM-1 transfected with plasmid vector carrying mutant glaucomatous variants of MYOC gene [249]: IL1A upregulation as a candidate molecular marker for POAG	▼
		rs1681268075:C	cttaagacaa	T→C	agccacgcct	6.00	0.53	4.02	0.38	6.21	10^{-6}	A			
		rs1681268246:T	gccacgccta	C→T	gtagccacgt	6.00	0.53	4.33	0.36	5.38	10^{-6}	A			
74	*IL1B* (3553)	rs1387100797:C	tggatactgc	T→C	tattctggga	5.87	0.59	6.81	0.69	2.09	10^{-6}	A	↓	within human eye disease models using mice [250]: Il1b-downregulation can reduce apoptosis of retinal ganglion cells that may relieve POAG	▲
		rs1682104638:C	ccctttcctt	T→C	atactctttt	9.56	0.81	11.95	1.16	3.44	10^{-6}	A			
		rs549858786:T	tgaaagccat	A→T	acttctgctt	4.50	0.39	6.81	0.45	7.63	10^{-6}	A			
		rs1143627:T	ttttgaaagc	C→T	cctacttctg	4.50	0.39	1.76	0.17	14.56	10^{-6}	A	↑	within human POAG cellular models using human trabecular meshwork cell line TM-1 transfected with plasmid vector carrying mutant glaucomatous variants of MYOC gene [249]: IL1B upregulation as a candidate molecular marker for POAG	▼
		rs1682102652:A	tggcagatac	C→A	gagggagaaa	22.81	2.20	7.49	0.73	16.21	10^{-6}	A			
		rs4986962:T	attcaacaga	G→T	tgaaatcagg	9.56	0.81	8.31	0.77	2.24	10^{-6}	A			
		rs529869449:A	ggagaaactg	G→A	aaaacagcga	22.81	2.20	14.71	1.46	6.35	10^{-6}	A			
		rs561088583:A	ctggcagata	C→A	cgagggagaa	22.81	2.20	10.14	0.95	12.08	10^{-6}	A			
75	*IL1RN* (3557)	rs1275462628:C	ggagggaatc	A→C	gttacaacac	10.80	1.26	13.52	1.64	2.67	10^{-2}	C	↓	within human disease models using Il1rn-knockout mice retinal pigment epithelial cells [251]: reduced suppression of antigen-presenting mature dendritic cells, that can alleviate autoimmune response in POAG [252]	▲
		rs1687004378:C	actatttctt	T→C	ataaaccaca	2.45	0.26	6.63	0.65	13.70	10^{-6}	A			
		rs761519699:G	ggaatcagtt	A→G	caacactcca	10.80	1.26	15.81	1.03	5.69	10^{-6}	A			
		rs906693964:G	tcctccttgt	A→G	ctctctgagg	8.24	0.83	23.84	1.84	16.70	10^{-6}	A			
		rs906693964:T	tcctccttgt	A→T	ctctctgagg	8.24	0.83	23.84	1.84	16.70	10^{-6}	A			
		rs1045348184:T	tgggctcctc	C→T	ttgtactctc	8.24	0.83	4.69	0.48	7.84	10^{-6}	A	↑	within human disease models using transgenic mice carrying an additional Il1rn gene copy [253]: resistant to herpetic stromal keratitis as a complication of POAG treatment with drugs that can suppress the immune system [254]	▼
		rs1372431138:T	ctgggctcct	C→T	cttgtactct	8.24	0.83	5.74	0.59	5.02	10^{-6}	A			
76	*IL2* (3558)	rs1281607562:A	atccccaccc	C→A	gttacttttg	6.88	0.61	5.46	0.49	3.67	10^{-3}	B	↑	within a biomedical cohort-based study [256]: IL2 excess in lacrimal fluid is a biomedical molecular marker for POAG	▼
		rs1797736596:T	ccccccttaaa	G→T	ttgtatcccc	6.88	0.61	3.64	0.40	8.99	10^{-6}	A			
		rs55739620:A	gtttcataca	G→A	gaggaaaaac	6.88	0.61	5.00	0.47	4.92	10^{-6}	A			
		rs55936127:T	cttttgtatc	C→T	tttctgagtt	6.88	0.61	5.98	0.53	2.24	0.05	D			
77	*IL6* (3569)	rs1206724976:C	gaggtcactg	T→C	tttatcgatc	6.61	0.57	9.27	1.01	4.87	10^{-3}	B	↓	within a biomedical cohort study [257]: IL6 deficit in trabecular meshwork is a biomedical molecular marker for trabecular meshwork fibrosis in POAG	▼
		rs1783994704:C	cactgtttta	T→C	cgatcttgaa	6.61	0.57	9.27	1.01	4.87	10^{-3}	B			
		rs1784019589:C	caaagattta	T→C	caaatgtggg	10.65	0.96	17.06	1.40	7.73	10^{-6}	A			
		rs201575115:A	agagtctcaa	C→A	ccccaataaa	2.87	0.48	4.89	0.30	5.99	10^{-6}	A			
		rs895939328:G	caataaatat	A→G	ggactggaga	2.87	0.48	4.57	0.45	4.79	10^{-3}	B			
		rs1389636465:G	ccctccaaca	A→G	agatttatca	10.65	0.96	8.00	0.75	4.41	10^{-3}	B	↑	within a biomedical cohort-based study [258]: IL6 excess is a biomedical molecular marker for POAG	
		rs1784019552:G	caacaaagat	T→G	tatcaaatgt	10.65	0.96	8.19	0.80	3.94	10^{-3}	B			
		rs2069827:T	ctgttttatc	G→T	atcttgaaga	6.61	0.57	5.44	0.44	3.28	10^{-2}	C			
		rs527770772:C	ccaacaaaga	T→C	ttatcaaatg	10.65	0.96	9.00	0.65	2.91	10^{-2}	C			
		rs899406081:T	ctccaacaaa	G→T	atttatcaaa	10.65	0.96	4.56	0.41	13.31	10^{-6}	A			
78	*IL6R* (3570)	rs1011958394:C	ttctacatag	T→C	gtccatgtgc	3.90	0.36	5.45	0.46	5.32	10^{-6}	A	↓	according to an exhaustive review [259]: the anti-IL6R humanized monoclonal antibody tocilizumab is a new promising drug for rheumatoid arthritis, a risk factor for which is POAG [194]	▲
		rs1266520720:C	cggttcccat	T→C	agcctgtccg	42.27	2.93	74.74	4.91	11.93	10^{-6}	A			
		rs1285855598:C	ccgctccggc	T→C	ttcgtaaccg	13.82	1.27	18.74	1.85	4.52	10^{-3}	B			
		rs1339656293:G	ccggttccca	T→G	tagcctgtcc	42.27	2.93	50.63	4.61	3.15	10^{-2}	C			
		rs1687629160:A	tcaacagaac	C→A	gggaggaagg	19.75	1.68	22.50	1.61	2.35	0.05	D			
		rs766792218:G	agctctttct	A→G	catagtgtcc	3.90	0.36	12.02	1.13	17.02	10^{-6}	A			
		rs938877359:G	gcccggttcc	C→G	attagcctgt	42.27	2.93	47.66	3.61	2.34	0.05	D			
		rs951837376:T	atcaacagaa	C→T	cgggaggaag	19.75	1.68	23.04	1.56	2.84	10^{-2}	C			
		rs986153559:C	gctccggctt	T→C	cgtaaccgca	13.82	1.27	34.77	3.33	13.90	10^{-6}	A			

Table S3. Cont.

Human Gene		Candidate SNP marker				K_D, nM, *in silico*				Significance				Effect of changes in human gene expression on the development of primary open-angle glaucoma (POAG, ☼: "▼" aggravation, "▲" alleviation) [Reference]	☼ ▲ ▼
#	*NCBI Gene Symbol* (NCBI Gene ID)	dbSNP ID:min [437]	5′ flank, 10 bp	WT →min	3 flank, 10 bp	WT MEAN±	SEM	min MEAN±	SEM	Z	p	ϱ	Δ		
78	*IL6R* (3570)	rs1019106367:T	actgggtgct	C→T	aggaagccgg	49.33	3.37	30.50	2.57	8.86	10^{-6}	A	↑	within a cohort-based biomedical study [257]: IL6R excess in trabecular meshwork is a biomedical molecular marker for trabecular meshwork fibrosis in POAG	▼
		rs1035138441:T	gtggctgata	C→T	gcccttttct	19.75	1.68	15.19	1.44	4.12	10^{-3}	B			
		rs1222019038:T	gcccttttct	C→T	atcaacagaa	19.75	1.68	9.29	0.62	13.95	10^{-6}	A			
		rs1262883973:A	aggctcccac	C→A	ccagcagatg	49.33	3.37	40.65	3.12	3.77	10^{-3}	B			
		rs1377208955:T	accaaaaatc	A→T	aaactaggcc	19.75	1.68	13.11	1.16	6.69	10^{-6}	A			
		rs1455492560:A	caatgccact	G→A	ttcactgggt	49.33	3.37	18.64	2.14	14.55	10^{-6}	A			
		rs1455492560:T	caatgccact	G→T	ttcactgggt	49.33	3.37	28.11	1.93	11.60	10^{-6}	A			
		rs1470654147:A	gtcccctgtt	C→A	tccccgctca	19.75	1.68	13.35	1.19	6.34	10^{-6}	A			
		rs1689159600:A	gggtagagcc	G→A	gaagacaatg	49.33	3.37	43.70	3.01	2.50	0.05	D			
		rs1689159825:T	agagccggaa	G→T	acaatgccac	49.33	3.37	12.72	1.28	22.32	10^{-6}	A			
		rs1689161079:G	ccactgttca	C→G	tgggtgctca	49.33	3.37	43.40	3.10	2.59	10^{-2}	C			
		rs1689502494:T	acagctcttt	C→T	tacatagtgt	3.90	0.36	2.42	0.26	6.75	10^{-6}	A			
		rs557148290:T	ctccggcttt	C→T	gtaaccgcac	13.82	1.27	6.66	0.64	10.97	10^{-6}	A			
		rs770930455:C	caccccagca	G→C	atgggctggc	49.33	3.37	38.47	3.03	4.77	10^{-3}	B			
		rs938877359:T	gcccggttcc	C→T	attagcctgt	42.27	2.93	15.86	1.37	17.73	10^{-6}	A			
		rs947543586:T	ctttcgtaac	C→T	gcaccctggg	13.82	1.27	10.12	1.05	4.49	10^{-3}	B			
79	*ITIH1* (3697)	rs138733525:T	tgtattgatt	A→T	ctggtctgac	7.86	0.76	9.24	0.87	2.39	0.05	D	↓	according to an exhaustive retrospective analysis of tumor-related transcriptomes [260]: ITIH1-deficit may be a biomedical molecular marker for breast tumors, a side effect of treatment of which using tamoxifen may be the tamoxifen-associated maculopathy aggravating POAG [167]	▼
		rs1490378042:G	aatgtggact	T→G	taactttctg	10.30	1.05	12.29	1.21	2.50	0.05	D			
		rs1559459214:C	cagcactgta	T→C	tgattactgg	7.86	0.76	9.77	1.06	3.00	10^{-2}	C			
		rs1698938551:T	ttatccagca	C→T	tgtattgatt	7.86	0.76	9.22	0.91	2.32	0.05	D			
		rs1698992699:C	ggtaattttg	T→C	aaactagaaa	4.93	0.45	9.21	0.83	9.70	10^{-6}	A			
		rs1698992748:T	attttgtaaa	C→T	tagaaagtcc	4.93	0.45	6.07	0.52	3.34	10^{-3}	B			
		rs564991103:C	actgtattga	T→C	tactggtctg	7.86	0.76	9.77	1.06	3.00	10^{-2}	C			
		rs779736713:G	cactgtattg	A→G	ttactggtct	7.86	0.76	9.77	1.06	3.00	10^{-2}	C			
		rs991345932:A	tccagcactg	T→A	attgattact	7.86	0.76	9.77	1.06	3.00	10^{-2}	C			
		rs1437359159:T	atgtccaggt	A→T	attttgtaaa	4.93	0.45	4.18	0.39	2.52	0.05	D	↑	within human disease models using mice [261]: Itih1-excess can exacerbate systemic insulin resistance, which may contribute to POAG development [262]	
		rs1464832323:A	cttgtctaat	G→A	tttaatccat	7.86	0.76	3.79	0.40	10.23	10^{-6}	A			
		rs1494317719:G	actttaactt	T→G	ctggaggtca	10.30	1.05	8.33	0.89	2.88	10^{-2}	C			
		rs1698938422:A	tttaatccat	G→A	tttatccagc	7.86	0.76	4.53	0.41	8.35	10^{-6}	A			
		rs1698938514:A	tgtttatcca	G→A	cactgtattg	7.86	0.76	6.79	0.65	2.15	0.05	D			
		rs368832013:A	atccagcact	G→A	tattgattac	7.86	0.76	3.35	0.37	11.66	10^{-6}	A			
		rs545105647:A	ccatgtttat	C→A	cagcactgta	7.86	0.76	2.84	0.36	12.84	10^{-6}	A			
80	*LDLR* (3949)	rs113750467:C	agttcgtctg	T→C	gactcagacc	37.32	2.63	53.51	4.50	6.58	10^{-6}	A	↓	within human disease models using Ldlr-knockout mice [263]: susceptibility to accelerated atherogenesis, which can aggravate POAG [143]	▼
		rs1357531646:G	aatgctgtaa	A→G	tgacgtgggc	7.43	0.70	9.05	0.89	2.90	10^{-2}	C			
		rs1568582310:C	attgaaatgc	T→C	gtaaatgacg	7.43	0.70	12.72	1.17	8.18	10^{-6}	A			
		rs747068848:C	atgctgtaaa	T→C	gacgtgggcc	7.43	0.70	11.55	1.14	6.47	10^{-6}	A			
		rs879254501:C	ctctcggcag	T→C	tcgtctgtga	37.32	2.63	46.22	3.55	4.11	10^{-3}	B			
		rs879254502:C	tctcggcagt	T→C	cgtctgtgac	37.32	2.63	52.42	4.05	6.50	10^{-6}	A			
		rs879254503:G	cggcagttcg	T→G	ctgtgactca	37.32	2.63	53.51	4.50	6.58	10^{-6}	A			
		rs879254505:C	gcagttcgtc	T→C	gtgactcaga	37.32	2.63	53.51	4.50	6.58	10^{-6}	A			
		rs879254506:C	cagttcgtct	G→C	tgactcagac	37.32	2.63	46.85	4.56	3.79	10^{-3}	B			
		rs879254507:G	ttcgtctgtg	A→G	ctcagaccgg	37.32	2.63	44.83	3.15	3.69	10^{-3}	B			
		rs879254511:C	ctgtgactca	G→C	accgggactg	37.32	2.63	41.42	3.00	2.06	0.05	D			
		rs969658891:A	ggggcttgtg	T→A	acgagcgggg	7.02	0.87	25.60	2.20	17.11	10^{-6}	A			

Table S3. Cont.

#	Human Gene: *NCBI Gene Symbol* (NCBI Gene ID)	Candidate SNP marker: dbSNP ID:min [437]	5′ flank, 10 bp	WT →min	3 flank, 10 bp	K_D, nM, *in silico*: WT MEAN	± SEM	min MEAN	± SEM	Significance: Z	p	ϱ	Δ	Effect of changes in human gene expression on the development of primary open-angle glaucoma (POAG, ☼: "▼" aggravation, "▲" alleviation) [Reference]	☼ ▲ ▼
80	*LDLR* (3949)	rs121908042:A	cccaagacgt	G→A	ctcccaggac	37.32	2.63	18.48	1.90	11.30	10^{-6}	A	↑	within a biomedical cohort-based study [264]: LDLR excess is a biomedically proven molecular marker for pterygium occurrence and progression, which blocks light exposure from the cornea to the retina and, thus, may reduce retinal activity and metabolic demands leading to a decreased vascular density in fundus that altogether can aggravate POAG [265]	▼
		rs193922571:A	gatgggaagt	G→A	catctctcgg	37.32	2.63	9.21	1.07	20.68	10^{-6}	A			
		rs201102461:A	gacgagtttc	G→A	ctgccacgat	37.32	2.63	30.01	2.06	4.44	10^{-3}	B			
		rs2077073153:T	gacggggctt	G→T	tgtacgagcg	7.02	0.87	4.19	0.42	6.46	10^{-6}	A			
		rs2077269298:A	gttcgtctgt	G→A	actcagaccg	37.32	2.63	13.17	1.41	16.30	10^{-6}	A			
		rs730882080:T	tcgctgccac	G→T	atgggaagtg	37.32	2.63	21.75	2.17	8.84	10^{-6}	A			
		rs730882081:G	tgtgactcag	A→G	ccgggactgc	37.32	2.63	33.31	2.41	2.25	0.05	D			
		rs762139262:T	ctcggcagtt	C→T	gtctgtgact	37.32	2.63	23.19	2.37	7.67	10^{-6}	A			
		rs769383881:A	ctcccaggac	G→A	agtttcgctg	37.32	2.63	26.34	1.99	6.76	10^{-6}	A			
		rs769383881:T	ctcccaggac	G→T	agtttcgctg	37.32	2.63	19.74	1.89	10.73	10^{-6}	A			
		rs869320648:A	ctgccacgat	G→A	ggaagtgcat	37.32	2.63	21.22	1.73	10.50	10^{-6}	A			
		rs875989899:T	catctctcgg	C→T	agttcgtctg	37.32	2.63	26.69	2.05	6.44	10^{-6}	A			
		rs879254486:T	gtttcgctgc	C→T	acgatgggaa	37.32	2.63	25.82	2.32	6.47	10^{-6}	A			
		rs879254502:A	tctcggcagt	T→A	cgtctgtgac	37.32	2.63	13.23	1.33	16.89	10^{-6}	A			
		rs879254506:A	cagttcgtct	G→A	tgactcagac	37.32	2.63	14.87	1.30	16.39	10^{-6}	A			
		rs980837425:T	gcttgtgtac	G→T	agcggggcgg	7.02	0.87	4.01	0.49	6.42	10^{-6}	A			
81	*LOXL1* (4016)	rs2068574970:G	ccggcgggcc	A→G	tagggccccg	63.68	7.17	116.91	13.07	7.66	10^{-6}	A	↓	according to an exhaustive review [266]: LOXL1 deficit can promote elastotic processes predisposing to extracellular matrix defects in the late stages of POAG	▼
		rs2068574994:C	cggcgggcca	T→C	agggccccgc	63.68	7.17	124.45	8.38	10.22	10^{-6}	A			
		rs1156950085:A	ctcccagcct	G→A	ttgcttattc	63.68	7.17	49.22	4.46	3.57	10^{-3}	B	↑	within a cohort biomedical pharmaceutical study on the medication treatment of pseudoexfoliation syndrome as an age-related POAG complication [267]: in comparison with conventionally healthy volunteers, patients had hypermethylation of the LOXL1 gene and, thus, its underexpression, whereas LOXL1 upregulation due to its hypomethylation resulted from treatment of these patients with azacitidine as a promising candidate adjuvant medication that may alleviate POAG in old age	▲
		rs1179605174:A	gcaggcggac	C→A	aggaggccga	63.68	7.17	44.90	3.52	5.09	10^{-6}	A			
		rs1309066213:A	aaagccggcg	G→A	gccatagggc	63.68	7.17	51.82	4.70	2.85	10^{-2}	C			
		rs2068574948:T	gccggcgggc	C→T	atagggcccc	63.68	7.17	18.58	1.98	15.91	10^{-6}	A			
		rs2068575346:T	ggcggaccag	G→T	aggccgaagc	63.68	7.17	41.05	3.17	6.43	10^{-6}	A			
		rs772065686:T	acgctcccag	C→T	ctgttgctta	63.68	7.17	54.25	3.84	2.41	0.05	D			
		rs930023705:A	ccagcctgtt	G→A	cttattcatt	63.68	7.17	47.96	4.52	3.86	10^{-3}	B			
		rs964130523:A	aagccggcgg	G→A	ccatagggcc	63.68	7.17	38.25	3.46	7.06	10^{-6}	A			
82	*LRRC27* (80313)	rs1032328739:C	ggggctcgcc	A→C	gcgcttcagt	48.10	3.75	54.38	4.23	2.23	0.05	D	↓	within a biomedical cohort-based study [268]: LRRC27 hypermethylation reducing its expression is a biomedical molecular marker for resistance to low-fat/low-carbohydrate Mediterranean diet together with physical activity as a treatment for obesity, which is positively correlated with POAG [115]	▼
		rs1032328739:G	ggggctcgcc	A→G	gcgcttcagt	48.10	3.75	54.38	4.10	2.26	0.05	D			
		rs1032542800:A	agcgcttcag	T→A	gggcggggac	48.10	3.75	61.70	5.10	4.39	10^{-3}	B			
		rs1032542800:C	agcgcttcag	T→C	gggcggggac	48.10	3.75	83.55	6.13	10.32	10^{-6}	A			
		rs1230234372:G	tcgccagcgc	T→G	tcagtgggcg	48.10	3.75	57.86	4.61	3.32	10^{-3}	B			
		rs1722709701:A	tcagtgggcg	G→A	ggacgcggca	48.10	3.75	53.89	4.25	2.05	0.05	D			
		rs2066797247:G	caggcgcatc	A→G	cagacacact	26.80	1.84	31.07	2.83	2.59	10^{-2}	C			
		rs2066797580:T	ggcgcatcac	A→T	gacacactcg	26.80	1.84	32.59	2.56	3.75	10^{-3}	B			
		rs2066797868:G	catcacagac	A→G	cactcgcgct	26.80	1.84	40.27	2.97	8.09	10^{-6}	A			
		rs557922665:G	ccagcgcttc	A→G	gtgggcgggg	48.10	3.75	83.55	6.13	10.32	10^{-6}	A			
		rs920186951:C	acagacacac	T→C	cgcgctcagc	26.80	1.84	30.78	2.16	2.82	10^{-2}	C			

Table S3. Cont.

Human Gene		Candidate SNP marker				K_D, nM, *in silico*				Significance				Effect of changes in human gene expression on the development of primary open-angle glaucoma (POAG, ☼: "▼" aggravation, "▲" alleviation) [Reference]	☼ ▲ ▼
#	*NCBI Gene Symbol* (NCBI Gene ID)	dbSNP ID:min [437]	5′ flank, 10 bp	WT →min	3 flank, 10 bp	WT MEAN	± SEM	min MEAN	± SEM	Z	p	ϱ	Δ		
82	*LRRC27* (80313)	rs1218908254:T	ctcccaggac	C→T	taataggaaa	6.31	0.64	3.32	0.32	9.15	10^{-6}	A	↑	within a biomedical cohort-based study of patients with preeclampsia in comparison with patients with gestational hypertension [269]: LRRC27 excess is a biomedical molecular marker for preeclampsia with thrombosis, vascular and endothelial dysfunction, the last of which is successfully treated using Tanakan in POAG as its relief [270]	▼
		rs1275495339:T	ctcgccagcg	C→T	ttcagtgggc	48.10	3.75	26.26	2.32	10.27	10^{-6}	A			
		rs1306327580:G	gccagcgctt	C→G	agtgggcggg	48.10	3.75	38.32	2.76	4.29	10^{-3}	B			
		rs1319368690:C	ggacctaata	G→C	gaaagatgga	6.31	0.64	5.02	0.56	3.01	10^{-2}	C			
		rs1379933866:T	gattccgtac	C→T	ggcgtctcca	33.82	2.63	26.04	2.91	3.84	10^{-3}	B			
		rs1425819684:A	ggctcgccag	C→A	gcttcagtgg	48.10	3.75	41.11	3.29	2.81	10^{-2}	C			
		rs2066797781:T	gcatcacaga	C→T	acactcgcgc	26.80	1.84	9.87	0.92	17.23	10^{-6}	A			
		rs2066808640:A	cgcggcaggt	G→A	agacggggcg	48.10	3.75	21.64	2.11	12.80	10^{-6}	A			
		rs2067048113:A	gatggaattt	G→A	tattgtgaca	6.31	0.64	2.51	0.26	12.70	10^{-6}	A			
		rs2067048699:A	aatttgtatt	G→A	tgacatagcc	6.31	0.64	4.81	0.47	3.85	10^{-3}	B			
		rs543275155:A	cagacacact	C→A	gcgctcagcg	26.80	1.84	17.20	1.62	7.60	10^{-6}	A			
		rs550119797:C	ggaatttgta	T→C	tgtgacatag	6.31	0.64	5.06	0.51	3.09	10^{-2}	C			
		rs557922665:T	ccagcgcttc	A→T	gtgggcgggg	48.10	3.75	43.21	3.24	1.98	0.05	D			
		rs934308654:T	tccgtaccgg	C→T	gtctccatgg	33.82	2.63	29.47	2.94	2.17	0.05	D			
		rs975721881:T	aggcgcatca	C→T	agacacactc	26.80	1.84	11.14	0.97	15.85	10^{-6}	A			
		rs991218838:T	ctgcgggatt	C→T	cgtaccggcg	33.82	2.63	12.85	1.30	15.19	10^{-6}	A			
83	*LTBP2* (4053)	rs1307270399:G	cagctggagt	A→G	ccgtgcgagc	33.32	2.90	97.51	7.09	18.92	10^{-6}	A	↓	within biomedical studies of Pakistani families and Gypsy patients [271]: LTBP2-null is a biomedical molecular marker for POAG	▼
		rs1348464563:C	ccagctggag	T→C	gccgtgcgag	33.32	2.90	112.51	7.41	22.28	10^{-6}	A			
		rs1348464563:G	ccagctggag	T→G	gccgtgcgag	33.32	2.90	112.51	7.41	22.28	10^{-6}	A			
		rs1385818690:C	tgtggataaa	A→C	agggggcctg	6.88	0.59	11.04	1.00	7.59	10^{-6}	A			
		rs1385818690:G	tgtggataaa	A→G	agggggcctg	6.88	0.59	11.01	0.96	7.69	10^{-6}	A			
		rs1201338234:A	acacagaatg	C→A	tgcagagtcc	20.16	1.50	14.63	0.95	6.49	10^{-6}	A	↑	within human glaucoma tissue models using mice with the interference RNA-impaired ciliary zonule formation leading lens luxation as POAG symptom and, next, treated with exogenous recombinant purified Ltbp2 within mice eye tissue level ex vivo [272]: restored unfragmented and bundled ciliary zonules to almost their norms	▲
		rs1407978288:A	agatgtggat	G→A	tgccctattt	20.16	1.50	7.35	0.66	17.23	10^{-6}	A			
		rs1419502657:A	ggcctggtgt	G→A	tccgaggagg	6.88	0.59	3.96	0.34	9.13	10^{-6}	A			
		rs2087047756:T	aatgcaatgc	C→T	gtcccacaca	20.16	1.50	16.94	1.54	2.96	10^{-2}	C			
		rs2087047792:A	agaatgcaat	G→A	gagtcccaca	20.16	1.50	12.92	1.18	7.56	10^{-6}	A			
		rs2087048045:T	cagagtccca	C→T	tgtctgtcct	20.16	1.50	14.87	1.37	5.13	10^{-6}	A			
		rs2088625595:A	ggagtaggag	C→A	cgagcccagc	33.32	2.90	25.90	2.58	3.81	10^{-3}	B			
		rs565718367:A	gtggataaaa	G→A	gggggcctgg	6.88	0.59	5.99	0.51	2.30	0.05	D			
		rs991812434:T	gtcccacaca	G→T	tgtcctgcag	20.16	1.50	8.19	0.71	15.74	10^{-6}	A			
84	*MFN1* (55669)	rs1288173219:T	gaattttatt	A→T	caagtggagt	4.49	0.50	6.54	0.54	5.44	10^{-6}	A	↓	within human disease models using Mfn1/Mfn2-double-knockout mice subjected with artificial acute cardiac ischemia-reperfusion [273]: mitochondrial dysfunction leading post-ischemic cardiac arrhythmogenesis, which may also occur as a complication of POAG treatment with latanoprost [274]	▼
		rs1302035114:G	aatttatttc	A→G	ttggggagtt	7.48	0.49	8.27	0.50	2.25	0.05	D			
		rs1365229909:C	ggggatccgg	A→C	cctagacggt	119.09	9.67	135.77	10.42	2.35	0.05	D			
		rs1455351340:C	cgcctggcat	A→C	gatgcgcttc	13.60	1.41	30.56	2.15	12.91	10^{-6}	A			
		rs1711958815:C	cctggcatag	A→C	tgcgcttccg	13.60	1.41	30.56	2.15	12.91	10^{-6}	A			
		rs1711982747:C	gatccggacc	T→C	agacggtcgt	119.09	9.67	135.77	10.42	2.35	0.05	D			
		rs1712020204:C	gcataattta	T→C	ttcattgggg	7.48	0.49	11.49	0.58	10.33	10^{-6}	A			
		rs756528332:G	attttattac	A→G	agtggagttt	4.49	0.50	5.20	0.48	2.04	0.05	D			
		rs762897527:G	agcataattt	A→G	tttcattggg	7.48	0.49	14.36	1.07	13.15	10^{-6}	A			
		rs959587664:C	ccggacctag	A→C	cggtcgtgcg	119.09	9.67	135.77	10.42	2.35	0.05	D			

Table S3. Cont.

#	Human Gene: *NCBI Gene Symbol* (NCBI Gene ID)	Candidate SNP marker: dbSNP ID:min [437]	5′ flank, 10 bp	WT →min	3 flank, 10 bp	K_D, nM, *in silico*: WT MEAN	± SEM	min MEAN	± SEM	Significance: Z	p	ϱ	Δ	Effect of changes in human gene expression on the development of primary open-angle glaucoma (POAG, ☼: "▼" aggravation, "▲" alleviation) [Reference]	☼ ▲ ▼
84	*MFN1* (55669)	rs1043939606:T	gcggccgagt	C→T	gagcccacgg	119.09	9.67	67.99	4.86	10.36	10^{-6}	A	↑	within human ocular disease models using mice [275]: Mfn1 upregulation can protect against mitochondrial damage in lens epithelial cells under light-induced oxidative stress inevitable at the eye vital function, that may alleviate POAG	▲
		rs1232796414:T	gggtgggatt	G→T	ggggatccgg	119.09	9.67	72.07	5.06	9.35	10^{-6}	A			
		rs1378323268:G	gtcttgaact	C→G	ttgacctcag	26.58	1.81	17.92	1.22	8.19	10^{-6}	A			
		rs1392953505:T	ggccaggctg	G→T	tcttgaactc	26.58	1.81	20.78	1.39	5.15	10^{-6}	A			
		rs1466621742:T	cggccgagtc	G→T	agcccacgga	119.09	9.67	54.10	5.30	12.39	10^{-6}	A			
		rs147133928:T	ggatccggac	C→T	tagacggtcg	119.09	9.67	69.87	4.77	10.05	10^{-6}	A			
		rs1576998575:G	aaagaattac	C→G	taaaaacatt	7.48	0.49	5.99	0.55	3.91	10^{-3}	B			
		rs1576998575:T	aaagaattac	C→T	taaaaacatt	7.48	0.49	4.96	0.53	6.53	10^{-6}	A			
		rs1577003845:T	ccaggctggt	C→T	ttgaactctt	26.58	1.81	16.04	1.15	10.22	10^{-6}	A			
		rs1577003849:T	ggctggtctt	G→T	aactcttgac	26.58	1.81	8.42	0.61	23.10	10^{-6}	A			
		rs1711958701:A	gcctggcata	G→A	atgcgcttcc	13.60	1.41	5.76	0.55	12.23	10^{-6}	A			
		rs1711980993:T	gcccacggag	C→T	ccacgaggcc	119.09	9.67	89.15	7.79	4.85	10^{-3}	B			
		rs1711981413:T	gccgtgctcg	G→T	gggtgggatt	119.09	9.67	103.90	8.23	2.40	0.05	D			
		rs1711982280:A	ggtgggattg	G→A	gggatccgga	119.09	9.67	79.32	5.33	7.71	10^{-6}	A			
		rs1712019783:T	attttgccag	C→T	ataatttatt	7.48	0.49	5.21	0.44	6.74	10^{-6}	A			
		rs1712495663:T	gctggtcttg	A→T	actcttgacc	26.58	1.81	7.37	0.74	21.07	10^{-6}	A			
		rs549641321:A	tggcatagat	G→A	cgcttccggc	13.60	1.41	4.54	0.50	14.49	10^{-6}	A			
		rs772715781:C	ttttgccagc	A→C	taatttattt	7.48	0.49	6.35	0.52	3.10	10^{-2}	C			
		rs772715781:T	ttttgccagc	A→T	taatttattt	7.48	0.49	6.24	0.47	3.60	10^{-3}	B			
		rs957872634:T	ggcatagatg	C→T	gcttccggcc	13.60	1.41	11.58	1.03	2.34	0.05	D			
		rs977402966:T	tgtctgacta	G→T	aacactccca	7.48	0.49	3.22	0.39	12.26	10^{-6}	A			
85	*MFN2* (9927)	rs1645952041:C	ccttgacagc	T→C	tgaggctccg	43.83	3.25	49.36	4.09	2.14	0.05	D	↓	within a human disease model using Mnf2-knockout mice subjected to artificial ischemia/reperfusion injury of the retina [276]: resistance to metformin-based treatment of retinal ischemia/reperfusion injury, which can exacerbate POAG	▼
		rs1645953898:G	caccggaact	A→G	cagcccccat	21.45	2.62	62.13	4.00	15.41	10^{-6}	A			
		rs1645953967:G	ccggaactac	A→G	gcccccatga	21.45	2.62	32.85	3.74	5.11	10^{-6}	A			
		rs1645959452:G	tgacgcgctt	A→G	tccacttccc	21.74	2.00	57.25	4.99	15.28	10^{-6}	A			
		rs1645959483:C	gacgcgctta	T→C	ccacttccct	21.74	2.00	39.85	3.89	9.03	10^{-6}	A			
		rs1645959529:T	gcgcttatcc	A→T	cttccctcct	21.74	2.00	27.41	2.47	3.61	10^{-3}	B			
		rs1645959567:T	cgcttatcca	C→T	ttccctcctc	21.74	2.00	29.23	2.64	4.59	10^{-3}	B			
		rs933337636:G	gtttttcgcc	C→G	cttgacagct	43.83	3.25	51.83	4.41	2.97	10^{-2}	C			
		rs1041955870:T	ccggcggagg	A→T	gtggcgcgcg	95.27	7.68	72.90	5.60	4.81	10^{-3}	B	↑	within a human POAG model using mice [277]: during glaucomatous neurodegeneration, retinal ganglion cells can selectively accumulate Mfn2-excess in a phosphorylated form	
		rs1057237656:A	tcgtccgctt	G→A	ggacgcgccg	95.27	7.68	46.61	4.21	11.81	10^{-6}	A			
		rs1234999126:A	gcgctgagac	G→A	ccgctcgaag	95.27	7.68	59.72	4.96	8.07	10^{-6}	A			
		rs1254242797:T	acgccgctcg	A→T	agcgccgagt	95.27	7.68	50.87	6.14	8.64	10^{-6}	A			
		rs1318782800:T	acgcccaccg	G→T	aactacagcc	43.83	3.25	16.95	1.58	15.96	10^{-6}	A			
		rs1327921121:T	gtggcgcgcg	G→T	aggagtggcg	95.27	7.68	24.15	2.41	21.41	10^{-6}	A			
		rs1348390203:T	ctgcgtcgtc	C→T	gcttgggacg	95.27	7.68	70.08	4.95	5.73	10^{-6}	A			
		rs1365524434:A	gagcctctgc	G→A	tcgtccgctt	95.27	7.68	64.78	4.30	7.39	10^{-6}	A			
		rs1411886443:A	ctgagacgcc	G→A	ctcgaagcgc	95.27	7.68	66.50	4.48	6.85	10^{-6}	A			
		rs1450668544:A	cagcccccat	G→A	atgcagtggg	21.45	2.62	18.46	1.59	2.01	0.05	D			
		rs1454216455:T	ccgctcgaag	C→T	gccgagtcgc	95.27	7.68	62.29	4.78	7.63	10^{-6}	A			
		rs1470702496:A	cgctgagacg	C→A	cgctcgaagc	95.27	7.68	78.12	5.01	3.86	10^{-3}	B			
		rs1470702496:T	cgctgagacg	C→T	cgctcgaagc	95.27	7.68	81.50	5.88	2.89	10^{-2}	C			
		rs1569763080:T	tggtgacgcg	C→T	ttatccactt	21.74	2.00	12.09	1.09	9.09	10^{-6}	A			
		rs1645953750:T	cgacgcccac	C→T	ggaactacag	43.83	3.25	35.50	4.08	3.08	10^{-2}	C			
		rs1645954824:A	cgtccgcttg	G→A	gacgcgccgg	95.27	7.68	64.83	4.18	7.46	10^{-6}	A			

Table S3. Cont.

Human Gene		Candidate SNP marker				K_D, nM, *in silico*				Significance				Effect of changes in human gene expression on the development of primary open-angle glaucoma (POAG, ☼: "▼" aggravation, "▲" alleviation) [Reference]	☼ ▲ ▼
						WT		min							
#	*NCBI Gene Symbol* (NCBI Gene ID)	dbSNP ID:min [437]	5′ flank, 10 bp	WT →min	3 flank, 10 bp	MEAN±	SEM	MEAN±	SEM	Z	p	ϱ	Δ		
		rs1645954856:T	gtccgcttgg	G→T	acgcgccggc	95.27	7.68	25.24	2.73	19.67	10^{-6}	A			
		rs1645955407:T	ggagtggcgc	G→T	cggaggagtg	95.27	7.68	84.86	6.86	2.03	10^{-6}	A			
		rs1645959503:T	acgcgcttat	C→T	cacttccctc	21.74	2.00	16.54	1.44	4.32	10^{-6}	A			
		rs534796178:A	ccgagcctct	G→A	cgtcgtccgc	95.27	7.68	21.09	1.97	24.43	10^{-6}	A			
		rs537683714:A	ggcgcgctga	G→A	acgccgctcg	95.27	7.68	68.51	4.77	6.19	10^{-6}	A			
		rs537683714:C	ggcgcgctga	G→C	acgccgctcg	95.27	7.68	77.09	6.33	3.68	10^{-6}	A			
		rs568548916:A	gcgcgcggag	G→A	agtggcgcgc	95.27	7.68	39.30	3.05	15.82	10^{-6}	A			
85	*MFN2* (9927)	rs886045216:T	cgccgagtcg	C→T	ggggcagcag	95.27	7.68	69.23	5.83	5.48	10^{-6}	A	↑	within a human POAG model using mice [277]: during glaucomatous neurodegeneration, retinal ganglion cells can selectively accumulate Mfn2-excess in a phosphorylated form	▼
		rs899316973:T	gagtggcgcg	C→T	tgagacgccg	95.27	7.68	69.97	5.26	5.61	10^{-6}	A			
		rs904417540:A	gaggagtggc	G→A	cgcggaggag	95.27	7.68	79.57	5.74	3.33	10^{-6}	A			
		rs933337636:T	gtttttcgcc	C→T	cttgacagct	43.83	3.25	38.08	2.98	2.61	10^{-6}	A			
		rs940925759:A	gcgcggagga	G→A	tggcgcgctg	95.27	7.68	78.20	5.94	3.57	10^{-6}	A			
		rs945868855:A	gccggcggag	G→A	agtggcgcgc	95.27	7.68	39.30	3.05	15.83	10^{-6}	A			
		rs945868855:T	gccggcggag	G→T	agtggcgcgc	95.27	7.68	21.52	2.22	22.76	10^{-6}	A			
		rs973376897:A	gacgcccacc	G→A	gaactacagc	21.45	2.62	16.91	1.95	2.83	10^{-6}	A			
		rs1013008693:G	gctttacctc	T→G	ttttagaatt	9.83	0.85	11.58	1.07	2.58	10^{-2}	C			
		rs1024186563:G	cagctcatca	A→G	tatcagcagc	11.67	1.15	16.06	1.51	4.71	10^{-3}	B			
		rs1028568119:C	catctataaa	T→C	ggaaacacta	1.82	0.18	3.20	0.40	7.05	10^{-6}	A			
		rs1189317675:G	aaaaaaatat	A→G	tagctgtgca	2.04	0.28	5.84	0.73	11.29	10^{-6}	A			
		rs1189317675:T	aaaaaaatat	A→T	tagctgtgca	2.04	0.28	6.33	0.61	13.43	10^{-6}	A			
		rs1227401381:G	ttgaacatct	A→G	taaatggaaa	1.82	0.18	5.97	0.56	17.20	10^{-6}	A			
		rs1303232260:G	tcgctccctt	A→G	tgtgatgaat	8.10	0.68	19.57	1.54	15.27	10^{-6}	A			
		rs1376440189:C	tgaacatcta	T→C	aaatggaaac	1.82	0.18	4.98	0.48	14.41	10^{-6}	A			
		rs1433047521:C	aaaatatata	G→C	ctgtgcatca	2.04	0.28	3.01	0.28	4.69	10^{-3}	B			
		rs1435532095:G	cgctccctta	T→G	gtgatgaatc	8.10	0.68	16.70	1.58	11.40	10^{-6}	A	↓	within human age-related disease models using Mlip-knockout mice [278]: impaired cardiac adaptation, which is comorbid with POAG, at least within the framework of age-related disorders	
		rs1469467801:G	aaaaaaaaat	A→G	tatagctgtg	2.04	0.28	6.87	0.83	13.24	10^{-6}	A			
		rs1582166772:T	tgttgtttta	G→T	tatgaggctc	4.00	0.41	4.83	0.36	3.01	10^{-2}	C			
		rs1768483302:C	aaaaaatata	T→C	agctgtgcat	2.04	0.28	6.20	0.83	11.56	10^{-6}	A			
		rs1769451774:G	ttgttttagt	A→G	tgaggctccc	4.00	0.41	7.27	0.64	8.92	10^{-6}	A			
		rs1769456379:A	ctcccttatg	T→A	gatgaatcaa	8.10	0.68	17.91	1.38	13.85	10^{-6}	A			
86	*MLIP* (90523)	rs528035721:G	gctcatcaat	A→G	tcagcagctg	11.67	1.15	16.06	1.51	4.71	10^{-3}	B			▼
		rs556504680:C	tcctgcaata	T→C	ctaacatgta	5.24	0.61	6.17	0.53	2.27	0.05	D			
		rs750862540:C	aaaaaaaata	T→C	atagctgtgc	2.04	0.28	5.60	0.66	11.09	10^{-6}	A			
		rs750862540:G	aaaaaaaata	T→G	atagctgtgc	2.04	0.28	9.22	0.97	17.39	10^{-6}	A			
		rs939065662:A	aaaaaaaaaa	T→A	atatagctgt	2.04	0.28	7.02	0.75	14.14	10^{-6}	A			
		rs1007099868:T	gggcaagttt	C→T	ggtgtgagtt	31.80	3.39	27.55	2.43	2.08	0.05	D			
		rs1010048418:A	tcccttatgt	G→A	atgaatcaat	8.10	0.68	2.90	0.32	14.68	10^{-6}	A			
		rs1015364932:T	ggaaaaaaaa	A→T	atatatagct	2.04	0.28	0.99	0.11	8.24	10^{-6}	A			
		rs1017061965:T	tgggaagttt	C→T	gagaagacgc	31.80	3.39	24.16	1.84	4.20	10^{-3}	B			
		rs1025013403:A	gaggcggaat	G→A	ggtggggcaa	31.80	3.39	24.55	2.43	3.56	10^{-3}	B			
		rs1044926262:A	tgcagctcat	C→A	aatatcagca	11.67	1.15	4.01	0.36	16.03	10^{-6}	A	↑	within human health models using proteomics analysis of the high versus low body mass male Hu sheep of [279]: Mlip excess is an proven economically valuable molecular marker for fat-tailed sheep breeding, while obesity may aggravate POAG [115]	
		rs1044926262:T	tgcagctcat	C→T	aatatcagca	11.67	1.15	2.89	0.29	19.85	10^{-6}	A			
		rs1172367208:A	cctgcaatat	C→A	taacatgtaa	5.24	0.61	1.16	0.16	16.92	10^{-6}	A			
		rs1179230386:A	tcgagaagac	G→A	ctatgggctg	31.80	3.39	19.17	1.40	7.85	10^{-6}	A			
		rs1322646061:T	ttaatatatt	C→T	agatgggatt	6.97	0.58	2.44	0.23	16.91	10^{-6}	A			

Table S3. Cont.

#	Human Gene: NCBI Gene Symbol (NCBI Gene ID)	Candidate SNP marker: dbSNP ID:min [722]	5′ flank, 10 bp	WT →min	3 flank, 10 bp	K_D, nM, *in silico*: WT MEAN	WT ± SEM	min MEAN	min ± SEM	Significance: Z	p	ϱ	Δ	Effect of changes in human gene expression on the development of primary open-angle glaucoma (POAG, ☼: "▼" aggravation, "▲" alleviation) [Reference]	☼ ▲▼
86	*MLIP* (90523)	rs1350670646:G	gaaaaaaaaa	A→G	tatatagctg	2.04	0.28	1.50	0.18	3.37	10^{-3}	B	↑	within human health models using proteomics analysis of the high versus low body mass male Hu sheep of [279]: Mlip excess is an proven economically valuable molecular marker for fat-tailed sheep breeding, while obesity may aggravate POAG [115]	▼
		rs1383570270:G	agataggtta	A→G	tatattcaga	6.97	0.58	2.78	0.36	11.96	10^{-6}	A			
		rs1439244057:A	ttcgagaaga	C→A	gctatgggct	31.80	3.39	27.76	2.01	2.11	0.05	D			
		rs1581962081:T	caatatctaa	C→T	atgtaagcac	5.24	0.61	3.01	0.32	7.04	10^{-6}	A			
		rs1582166772:A	tgttgtttta	G→A	tatgaggctc	4.00	0.41	2.88	0.25	4.92	10^{-6}	A			
		rs1767283126:A	aatatattca	G→A	atgggattga	6.97	0.58	5.83	0.46	3.13	10^{-2}	C			
		rs1767285423:T	ttgctatttc	C→T	agtccagagt	6.97	0.58	5.79	0.53	3.01	10^{-2}	C			
		rs1768924778:A	ggcaagtttc	G→A	gtgtgagttt	31.80	3.39	22.79	1.85	4.97	10^{-6}	A			
		rs1768924943:A	tgtgagtttg	G→A	gaagtttcga	31.80	3.39	24.52	1.54	4.21	10^{-3}	B			
		rs1768925606:A	tgggctgggt	C→A	tgttaggttg	31.80	3.39	16.32	1.41	9.72	10^{-6}	A			
		rs1768925699:A	ggctgggtct	G→A	ttaggttggg	31.80	3.39	19.58	1.48	7.43	10^{-6}	A			
		rs533267071:A	cgagaagacg	C→A	tatgggctgg	31.80	3.39	11.94	1.05	14.17	10^{-6}	A			
		rs533267071:T	cgagaagacg	C→T	tatgggctgg	31.80	3.39	13.25	1.33	11.96	10^{-6}	A			
		rs547655005:T	tcaatatcag	C→T	agctgacaca	11.67	1.15	8.11	0.83	5.13	10^{-6}	A			
		rs570695011:A	gggaagtttc	G→A	agaagacgct	31.80	3.39	24.16	1.67	4.33	10^{-3}	B			
		rs570695011:T	gggaagtttc	G→T	agaagacgct	31.80	3.39	14.27	1.21	11.76	10^{-6}	A			
		rs772020715:T	tatgggctgg	G→T	tctgttaggt	31.80	3.39	25.01	2.23	3.46	10^{-3}	B			
		rs998979195:A	gtgtgagttt	G→A	ggaagtttcg	31.80	3.39	24.15	2.20	3.93	10^{-3}	B			
87	*MMP1* (4312)	rs1015706782:G	aggactctat	A→G	ctggaagggc	0.75	0.1	2.19	0.2	13.52	10^{6}	A	↓	within a cohort-based biomedical study [280]: pilocarpine-based treatment in POAG can accelerate with a decrease in MMP1 level	▲
		rs1353697700:C	ggactctata	T→C	tggaagggca	0.75	0.1	1.97	0.21	11.63	10^{6}	A			
		rs1443577762:G	caaggactct	A→G	ttctggaagg	0.75	0.1	1.18	0.18	4.64	10^{3}	B			
		rs983798242:C	actctatata	T→C	gaagggcaag	0.75	0.1	1.38	0.16	7.18	10^{6}	A			
88	*MMP2* (4313)	rs1250869995:G	ccagccggct	A→G	catctggcgg	12.71	1.52	84.78	6.96	26.14	10^{-6}	A	↓	within human glaucoma models using Mmp2-null mice [282]: resistance to retinal neovascularization aggravating POAG as well as attenuated retinal ganglion cell death and suppressed tumor necrosis factor, which may protect against neurodegeneration and neuroinflammation, respectively	▲
		rs1251356680:C	tgacccgaac	T→C	gtatatggtg	3.45	0.40	4.12	0.47	2.18	0.05	D			
		rs1269475894:C	cccagccggc	T→C	acatctggcg	12.71	1.52	34.33	2.68	13.90	10^{-6}	A			
		rs1281249278:A	gggactctta	T→A	gtaaatagcg	2.65	0.24	4.99	0.51	9.36	10^{-6}	A			
		rs1281249278:G	gggactctta	T→G	gtaaatagcg	2.65	0.24	5.17	0.49	10.24	10^{-6}	A			
		rs1396763374:G	cgaactgtat	A→G	tggtgaaatc	3.45	0.40	7.66	0.56	11.60	10^{-6}	A			
		rs1453770023:G	agccggctac	A→G	tctggcggct	12.71	1.52	20.66	2.46	5.75	10^{-6}	A			
		rs1487126432:A	tatgtaaata	G→A	cggggacatc	2.65	0.24	3.06	0.27	2.29	0.05	D			
		rs17859831:T	ggctacatct	G→T	gcggctgccc	12.71	1.52	15.13	1.75	2.10	0.05	D			
		rs1962054460:C	cttatgtaaa	T→C	agcggggaca	2.65	0.24	3.20	0.34	2.75	10^{-2}	C			
		rs1962071507:C	agagccctcc	T→C	tcctggctgg	89.48	6.55	103.18	8.26	2.63	10^{-2}	C			
		rs1962071624:C	ccctccttcc	T→C	ggctgggctc	89.48	6.55	103.18	8.26	2.63	10^{-2}	C			
		rs1962071624:G	ccctccttcc	T→G	ggctgggctc	89.48	6.55	103.18	8.26	2.63	10^{-2}	C			
		rs758455676:G	ggcacatcct	A→G	tgacagctgc	14.12	1.36	46.85	3.96	18.68	10^{-6}	A			

Table S3. Cont.

#	Human Gene: *NCBI Gene Symbol* (NCBI Gene ID)	Candidate SNP marker: dbSNP ID:min [437]	5′ flank, 10 bp	WT →min	3 flank, 10 bp	K$_D$, nM, *in silico*: WT MEAN	± SEM	min MEAN	± SEM	Significance: Z	p	ρ	Δ	Effect of changes in human gene expression on the development of primary open-angle glaucoma (POAG, ☼: "▼" aggravation, "▲" alleviation) [Reference]	☼ ▲▼
88	*MMP2* (4313)	rs1216060612:A	ccagggcaca	T→A	cctatgacag	14.12	1.36	12.16	1.09	2.27	0.05	D	↑	within a cohort-based study cohorts: in both aqueous humor [283] and conjunctival stroma [280], MMP2 excess is a biomedical molecular marker for POAG	▼
		rs12445370:T	agagcggcca	G→T	agagccctcc	89.48	6.55	30.13	3.29	16.55	10^{-6}	A			
		rs1259653896:A	gccggctaca	T→A	ctggcggctg	12.71	1.52	9.18	0.91	4.18	10^{-3}	B			
		rs1260539139:T	tgtctctgac	C→T	atctatcatt	15.94	1.32	6.37	0.69	13.50	10^{-6}	A			
		rs1262700604:A	ctgtctctga	C→A	catctatcat	15.94	1.32	13.52	1.34	2.55	0.05	D			
		rs1284747456:G	ccggctacat	C→G	tggcggctgc	12.71	1.52	7.79	0.84	6.07	10^{-6}	A			
		rs1332782015:T	gaaagggact	C→T	ttatgtaaat	2.65	0.24	2.25	0.20	2.56	0.05	D			
		rs1386045583:T	gagagccctc	C→T	ttcctggctg	89.48	6.55	55.92	4.03	9.16	10^{-6}	A			
		rs1390226555:T	gccctccttc	C→T	tggctgggct	89.48	6.55	68.58	4.67	5.32	10^{-6}	A			
		rs1422147463:T	cctgttttat	C→T	atagggtct	3.45	0.40	2.69	0.21	3.61	10^{-3}	B			
		rs1455241409:A	ctctgaccat	C→A	tatcattgtg	15.94	1.32	4.15	0.43	20.44	10^{-6}	A			
		rs1467245725:G	gcagcacttc	T→G	tagccagcat	21.44	1.81	15.04	1.49	5.45	10^{-6}	A			
		rs1468281902:T	catcctatga	C→T	agctgcacca	14.12	1.36	9.60	0.78	6.12	10^{-6}	A			
		rs1567371625:T	ggagagcggc	C→T	agagagccct	89.48	6.55	45.16	4.25	11.48	10^{-6}	A			
		rs1596802388:A	ctgatcatct	G→A	tttctgacca	15.94	1.32	14.15	1.22	2.00	0.05	D			
		rs17859827:A	catctatcat	T→A	gtggctgatc	15.94	1.32	8.45	0.66	11.12	10^{-6}	A			
		rs1961146234:T	agcaaagcag	C→T	acttcttagc	21.44	1.81	13.42	1.15	7.78	10^{-6}	A			
		rs1961146540:T	cagcacattt	C→T	aaggatcaga	21.44	1.81	9.37	0.68	14.88	10^{-6}	A			
		rs1962030567:A	cagcccagcc	G→A	gctacatctg	12.71	1.52	9.87	1.07	3.13	10^{-2}	C			
		rs1962030770:T	cagccggcta	C→T	atctggcggc	12.71	1.52	4.64	0.61	11.29	10^{-6}	A			
		rs1962070433:A	ccgctcgggt	C→A	ggagagcggc	89.48	6.55	32.88	3.27	16.21	10^{-6}	A			
		rs1962072156:T	gctcccaaac	C→T	gcggttcaga	89.48	6.55	74.63	6.08	3.31	10^{-3}	B			
		rs1962099425:A	gacccgaact	G→A	tatatggtga	3.45	0.40	1.17	0.15	12.43	10^{-6}	A			
		rs552587550:A	tctctgacca	T→A	ctatcattgt	15.94	1.32	11.04	0.96	6.14	10^{-6}	A			
		rs566831986:A	tcgtggccat	G→A	caaggggcat	21.44	1.81	11.83	1.31	8.52	10^{-6}	A			
		rs761682477:T	gctgggctcc	C→T	aaaccgcggt	89.48	6.55	22.55	2.07	23.51	10^{-6}	A			
		rs866688535:G	accatctatc	A→G	ttgtggctga	15.94	1.32	10.06	0.95	7.36	10^{-6}	A			
		rs897550290:G	gcccagccgg	C→G	tacatctggc	12.71	1.52	10.42	1.19	2.41	0.05	D			
		rs911452729:T	tccttcctgg	C→T	tgggctccca	89.48	6.55	65.37	4.59	6.19	10^{-6}	A			
		rs923187631:C	ccatctatca	T→C	tgtggctgat	15.94	1.32	12.87	1.15	3.53	10^{-3}	B			
89	*MMP9* (4318)	rs1720400719:T	ttaaagcccc	C→T	acaacagcag	15.00	1.48	10.52	1.10	4.96	10^{-6}	A	↑	within an observational pharmaceutical study using tear sample collection [285]: latanoprost, being a commonly accepted medication against POAG, can simultaneously both upregulate MMP9 and reduce intraocular pressure that can alleviate POAG	▲
		rs2084257509:T	tccttaaagc	C→T	cccacaacag	15.00	1.48	12.38	0.98	3.05	10^{-2}	C			
		rs2084257560:T	aaagcccccca	C→T	aacagcagct	15.00	1.48	11.18	1.10	4.24	10^{-3}	B			
90	*MMP12* (4321)	rs1294826446:G	taagggctat	A→G	agaacccgga	1.22	0.14	3.49	0.36	13.83	10^{-6}	A	↓	within human glaucoma models using Mmp12-knockout mice [286]: decreased levels of both adhesion molecule and inflammatory cytokines along with reduced vascular leakage in oxygen-induced retinopathy, as well as markedly reduced macrophage content in the retina with impaired macrophage migratory capacity, attenuated retinal capillary dropout and mitigated pathological retinal neovascularization that all improvements to POAG were reproduced using normal mice subjected with MMP408 as pharmacological inhibitor of MMP12	▲
		rs1859573534:G	agggctatat	A→G	aacccggact	1.22	0.14	3.65	0.38	14.37	10^{-6}	A			
		rs1859573681:C	ctaagggcta	T→C	cagaacccgg	1.22	0.14	3.35	0.29	14.19	10^{-6}	A			
		rs782146210:C	aagggctata	T→C	gaacccggac	1.22	0.14	3.70	0.40	14.33	10^{-6}	A			
		rs1555009916:A	cggactaagg	G→A	ttcacagaac	1.22	0.14	0.93	0.10	3.42	10^{-6}	A	↑	within a cohort-based biomedical study [281]: MMP12 excess in aqueous humor can be a biomedical molecular marker for susceptibility to POAG	▼

Table S3. Cont.

Human Gene		Candidate SNP marker				K_D, nM, *in silico*				Significance				Effect of changes in human gene expression on the development of primary open-angle glaucoma (POAG, ☼: "▼" aggravation, "▲" alleviation) [Reference]	☼ ▲ ▼
						WT		min							
#	*NCBI Gene Symbol* (NCBI Gene ID)	dbSNP ID:min [437]	5′ flank, 10 bp	WT →min	3 flank, 10 bp	MEAN±	SEM	MEAN±	SEM	Z	p	ϱ	Δ		
91	*MPP7* (143098)	rs1215625405:G	tttatatcgt	A→G	tgtataactt	2.06	0.21	2.50	0.25	2.70	10^{-2}	C	↓	within cohort-based study together with POAG models using human trabecular meshwork cells that was verified using murine eye subjected with mechanical stress mimicking the POAG development scenario [287]: MPP7 deficit can be a biomedical molecular marker for susceptibility to POAG	▼
		rs1351680556:G	gcccctctta	C→G	cctatcagca	26.30	2.41	37.03	3.05	5.55	10^{-6}	A			
		rs1834498574:G	acttgagtat	A→G	caagcacatt	2.19	0.24	8.26	0.85	17.68	10^{-6}	A			
		rs1834498574:T	acttgagtat	A→T	caagcacatt	2.19	0.24	3.29	0.35	5.35	10^{-6}	A			
		rs1834498597:C	gacttgagta	T→C	tcaagcacat	2.19	0.24	5.99	0.62	13.37	10^{-6}	A			
		rs1834498617:G	cattgacttg	A→G	aacgtcaagc	2.19	0.24	2.68	0.29	2.66	10^{-2}	C			
		rs1840627167:G	acttctttat	A→G	acgactgtat	2.06	0.21	5.00	0.63	10.83	10^{-6}	A			
		rs1840627261:G	taacttcttt	A→G	gaacgactgt	2.06	0.21	4.43	0.52	9.78	10^{-6}	A			
		rs1840627459:A	gactgtataa	C→A	tacagaggaa	2.06	0.21	2.49	0.26	2.54	0.05	D			
		rs1840691851:T	ttccttttac	A→T	cacctgtgca	5.52	0.55	7.91	0.80	5.10	10^{-6}	A			
		rs1840691944:G	acttcctttt	A→G	accacctgtg	5.52	0.55	14.13	1.23	14.27	10^{-6}	A			
		rs1840692025:A	cacttccttt	T→A	aaccacctgt	5.52	0.55	13.52	1.07	14.12	10^{-6}	A			
		rs928482229:C	ggcccctctt	A→C	gcctatcagc	26.30	2.41	100.38	6.47	23.91	10^{-6}	A			
		rs996366869:A	tctttatatc	G→A	actgtataac	2.06	0.21	3.17	0.32	5.96	10^{-6}	A			
		rs1026714565:A	agcaggcccc	T→A	cgcagcctat	26.30	2.41	18.06	1.72	5.68	10^{-6}	A	↑	within POAG models using human trabecular meshwork cells [112] along with an observations within cancer-derived xenograft mice [288]: MPP7 excess can stimulate autophagy that can reduce ocular hypertension as a relief in POAG	▲
		rs1045520229:T	ctcttacccg	C→T	tcagcaggcc	26.30	2.41	20.05	1.81	4.22	10^{-3}	B			
		rs1324073309:G	gtctgtgata	A→G	ggcacacagc	14.13	1.23	10.82	1.01	4.20	10^{-3}	B			
		rs1347213152:T	cctcttaccc	G→T	atcagcaggc	26.30	2.41	22.48	2.08	2.42	0.05	D			
		rs1451817170:T	cccctcttac	C→T	ctatcagcag	26.30	2.41	18.22	1.61	5.78	10^{-6}	A			
		rs1840692112:T	tgtgcacttc	C→T	caggaaccac	5.52	0.55	4.67	0.45	2.44	0.05	D			
		rs1840693932:A	acagctgtct	G→A	ctgtgaggca	14.13	1.23	8.93	0.57	8.50	10^{-6}	A			
		rs1840694944:A	gtagttttct	C→A	tatactgagg	14.13	1.23	11.82	1.04	2.89	10^{-2}	C			
		rs1841206989:A	ccccgcccct	C→A	gtgcagcccc	26.30	2.41	20.40	1.86	3.93	10^{-3}	B			
		rs560335233:A	ttctttatat	C→A	gactgtataa	2.06	0.21	0.62	0.07	15.65	10^{-6}	A			
		rs764399463:A	atatactgag	G→A	gaaaagagac	14.13	1.23	9.17	1.13	5.75	10^{-6}	A			
		rs764399463:T	atatactgag	G→T	gaaaagagac	14.13	1.23	10.34	1.19	4.32	10^{-3}	B			
		rs897221194:A	atactgaggt	G→A	aaagagacaa	14.13	1.23	7.68	0.96	8.02	10^{-6}	A			
92	*MTHFR* (4524)	rs1185939452:T	atatgggcct	C→T	cctcgctagg	12.84	0.89	14.33	0.97	2.26	0.05	D	↓	according to an exhaustive review [289]: Mthfr-deficient mice are an animal model for human hyperhomocysteinemia as a risk factor for both endothelial dysfunction and POAG [290]	▼
		rs13306559:C	gctaggaata	T→C	tactgcccct	12.84	0.89	15.62	1.25	3.70	10^{-3}	B			
		rs1375587605:C	tgacgataaa	G→C	ctcgcgtcac	7.34	0.63	9.05	0.81	3.38	10^{-3}	B			
		rs1399506999:T	aagcatatca	G→T	tctggggtca	11.01	1.00	14.51	1.12	4.63	10^{-3}	B			
		rs1463108966:G	tgcccctcgc	T→G	ctgggcacta	12.84	0.89	17.79	1.57	5.81	10^{-6}	A			
		rs1644264482:G	ggtcagaagc	A→G	gtcatctctg	11.01	1.00	16.03	1.14	6.49	10^{-6}	A			
		rs1644438619:T	gaatatgggc	C→T	cccctcgcta	12.84	0.89	15.92	1.05	4.49	10^{-3}	B			
		rs1644438857:C	tcgctaggaa	T→C	actactgccc	12.84	0.89	24.11	2.21	10.96	10^{-6}	A			
		rs1644442707:A	cagctccgcc	C→A	ttcctttgtc	20.93	1.50	26.39	2.00	4.44	10^{-3}	B			
		rs1644442827:A	cgcagctccg	C→A	tcttcctttg	20.93	1.50	24.69	1.83	3.20	10^{-2}	C			
		rs1644443009:T	tgtcgcagct	C→T	gcctcttcct	20.93	1.50	23.45	1.73	2.21	0.05	D			
		rs1644483334:T	cagtaagtgg	C→T	gactagttgt	10.31	1.04	12.04	1.12	2.27	0.05	D			
		rs1644486651:T	ggagaagagc	G→T	ggcgatcacc	46.35	3.32	54.67	3.88	3.27	10^{-2}	C			
		rs1644486787:G	cacctggaga	A→G	tccccggcga	46.35	3.32	86.38	6.33	12.15	10^{-6}	A			
		rs1644486929:C	gatcacctgg	A→C	gcctccccgg	46.35	3.32	91.04	6.84	13.00	10^{-6}	A			
		rs1644500738:G	tccgccctgt	A→G	cgccggtggt	46.96	5.36	66.22	5.02	5.01	10^{-6}	A			
		rs1644502153:T	cgtcacatga	C→T	gcgaggactc	7.34	0.63	10.27	0.86	5.60	10^{-6}	A			

Table S3. Cont.

Human Gene		Candidate SNP marker				K_D, nM, *in silico*				Significance				Effect of changes in human gene expression on the development of primary open-angle glaucoma (POAG, ☼: "▼" aggravation, "▲" alleviation) [Reference]	☼ ▲ ▼
#	*NCBI Gene Symbol* (NCBI Gene ID)	dbSNP ID:min [437]	5′ flank, 10 bp	WT →min	3 flank, 10 bp	WT MEAN	± SEM	min MEAN	± SEM	Z	p	ϱ	Δ		
92	*MTHFR* (4524)	rs1644502182:G	gcgtcacatg	A→G	ggcgaggact	7.34	0.63	10.21	0.88	5.43	10^{-6}	A			
		rs751044729:G	ctcgctagga	A→G	cactactgcc	12.84	0.89	20.36	1.57	8.89	10^{-6}	A			
		rs765899301:G	cgctaggaat	A→G	ctactgcccc	12.84	0.89	21.45	1.86	9.24	10^{-6}	A	↓	according to an exhaustive review [289]: Mthfr-deficient mice are an animal model for human hyperhomocysteinemia as a risk factor for both endothelial dysfunction and POAG [290]	▼
		rs907404476:G	tcacctggag	A→G	ctccccggcg	46.35	3.32	86.32	6.31	12.15	10^{-6}	A			
		rs956422292:G	cagttgaaca	C→G	gcagctccgc	20.93	1.50	23.68	2.02	2.22	0.05	D			
		rs1036129117:T	ctccagcaac	C→T	taggcccgcc	46.96	5.36	32.76	2.45	5.28	10^{-6}	A			
		rs1189620891:T	ggtttccgcc	C→T	cccgcgccgg	46.96	5.36	23.91	2.96	8.02	10^{-6}	A			
		rs1371242457:A	ccctcgctag	G→A	ggcactactg	12.84	0.89	6.89	0.57	11.57	10^{-6}	A			
		rs1375587605:A	tgacgataaa	G→A	ctcgcgtcac	7.34	0.63	5.33	0.46	5.26	10^{-6}	A			
		rs1447135183:T	tggagaagag	C→T	cggcgatcac	46.35	3.32	40.47	2.93	2.67	10^{-2}	C			
		rs1570504770:A	cagactagtt	G→A	agtgcttggc	10.31	1.04	5.72	0.56	8.41	10^{-6}	A			
		rs1570504770:T	cagactagtt	G→T	agtgcttggc	10.31	1.04	8.69	0.82	2.48	0.05	D			
		rs1570506681:A	caacctgaca	C→A	cgcctctcca	46.96	5.36	29.77	2.45	6.48	10^{-6}	A			
		rs1570506681:T	caacctgaca	C→T	cgcctctcca	46.96	5.36	34.17	2.70	4.58	10^{-3}	B			
		rs1644442470:G	ttgaacacac	C→G	gctccgccca	20.93	1.50	18.29	1.31	2.66	10^{-2}	C			
		rs1644483477:A	tgttcagtaa	G→A	cgcagactag	10.31	1.04	8.46	0.87	2.75	10^{-2}	C	↑	within a cohort-based study [291]: 3 weeks of yoga practice resulted in fivefold upregulation in the expression of MTHFR gene, whereas MTHFR-deficit may be a molecular marker for ocular defects according to human ocular defect models using zebrafish larvae [292]	▲
		rs1644486991:A	gcgatcacct	G→A	ctgcctcccc	46.35	3.32	18.01	1.44	17.61	10^{-6}	A			
		rs1644499583:G	cgccccttca	C→G	cacctgcgcc	43.54	2.87	35.06	2.26	4.70	10^{-6}	A			
		rs1644500504:A	cgcctctcca	G→A	ccctgtaggc	46.96	5.36	39.00	2.65	2.80	10^{-2}	C			
		rs1644500539:T	cccgcctctc	C→T	cgccctgtag	46.96	5.36	23.61	1.99	9.69	10^{-6}	A			
		rs1644500809:A	tggtttccgc	C→A	acccgcgccg	46.96	5.36	26.09	2.58	7.79	10^{-6}	A			
		rs1644500850:A	cggtggtttc	C→A	gtcacccgcg	46.96	5.36	37.51	3.93	2.90	10^{-2}	C			
		rs1644502225:A	cgcgtcacat	G→A	gggcgaggac	7.34	0.63	5.16	0.44	5.78	10^{-6}	A			
		rs956422292:T	cagttgaaca	C→T	gcagctccgc	20.93	1.50	14.46	1.02	7.34	10^{-6}	A			
		rs965866122:T	cccttcactg	C→T	ctgcgccgcg	43.54	2.87	25.95	1.54	11.67	10^{-6}	A			
		rs970393032:A	ccccttcact	G→A	cctgcgccgc	43.54	2.87	11.99	1.01	24.17	10^{-6}	A			
93	*MUTYH* (4595)	rs1012424747:C	agactacatc	T→C	ttatgagctc	9.40	1.12	22.11	2.08	11.27	10^{-6}	A			
		rs1294688351:C	acttccgttc	A→C	cgatgggtcg	48.21	3.91	99.42	8.09	12.59	10^{-6}	A			
		rs1302501876:G	cgggcgcgct	A→G	cgccggaaag	60.40	5.77	129.43	8.57	13.11	10^{-6}	A			
		rs1361833225:G	aacttccgtt	C→G	ccgatgggtc	48.21	3.91	54.82	3.76	2.42	0.05	D			
		rs1364717156:C	cgcggtgtac	A→C	gcagccggag	22.02	1.95	36.30	2.71	8.63	10^{-6}	A			
		rs1459137516:C	cggagccgcg	G→C	gcttttgcag	22.02	1.95	27.49	2.38	3.58	10^{-3}	B			
		rs1465744124:G	tatgagctcc	A→G	ctactaaccg	9.40	1.12	11.30	1.46	2.09	0.05	D			
		rs1570583528:G	tcgcgggagg	T→G	cgcgctagag	37.53	3.97	50.05	3.49	4.54	10^{-3}	B			
		rs1645941886:G	attagttctt	A→G	gtggaattat	4.13	0.41	9.60	0.90	12.41	10^{-6}				
		rs1645942445:G	tcattagttc	T→G	tggtggaatt	4.13	0.41	7.86	0.80	9.08	10^{-6}	A	↓	within human age-related disease models using mice aged 5 to 22 months [293]: Mutyh levels decrease with increasing age, which may contribute to the progression of age-related diseases, including POAG	▼
		rs1646749729:C	ccgggcgcgc	T→C	gcgccggaaa	60.40	5.77	129.43	8.57	13.11	10^{-6}	A			
		rs1646771781:G	gcgcgcgcta	A→G	aaggcagcct	29.46	2.21	36.89	3.07	4.02	10^{-3}	A			
		rs1646782494:T	ttccgttcag	A→T	atgggtcgaa	48.21	3.91	58.30	4.08	3.55	10^{-3}	B			
		rs1646859766:G	gccgcggtgt	A→G	ttgcagccgg	22.02	1.95	36.30	2.71	8.63	10^{-6}	B			
		rs1646873020:C	caactattac	T→C	gaactgaatc	4.07	0.39	9.40	1.12	11.01	10^{-6}	A			
		rs1646874467:G	gaatcacaac	T→G	tccactgaac	4.07	0.39	6.14	0.54	6.38	10^{-6}	A			
		rs3219477:C	ttagttctta	T→C	tggaattatc	4.13	0.41	13.31	1.22	17.40	10^{-6}	A			
		rs776814505:A	tagagctcgc	G→A	gccgggcgcg	37.53	3.97	44.14	3.69	2.40	0.05	D			
		rs914037253:A	tggcgcgcgc	T→A	ggaaggcagc	29.46	2.21	47.97	4.38	8.25	10^{-6}	A			

Table S3. Cont.

#	Human Gene: *NCBI Gene Symbol* (NCBI Gene ID)	Candidate SNP marker: dbSNP ID:min [437]	5′ flank, 10 bp	WT →min	3 flank, 10 bp	K_D, nM, *in silico*: WT MEAN	± SEM	min MEAN	± SEM	Significance: Z	p	ϱ	Δ	Effect of changes in human gene expression on the development of primary open-angle glaucoma (POAG, ☼: "▼" aggravation, "▲" alleviation) [Reference]	☼ ▲ ▼
93	*MUTYH* (4595)	rs988198038:G	ctaattgcct	A→G	cctggcgcgc	38.42	2.67	47.61	3.06	4.53	10^{-3}	B		within human age-related disease models using mice aged 5 to 22 months [293]: Mutyh levels decrease with increasing age, which may contribute to the progression of age-related diseases, including POAG	▼
		rs1645057147:C	cccttctttt	A→C	acagcagtgt	17.07	1.48	27.20	2.12	7.99	10^{-6}	A	↓		
		rs752665489:G	ctgagtcgtc	T→G	tcgtctcccg	54.79	3.95	64.66	4.90	3.17	10^{-2}	B			
		rs1009511367:A	agccggagcc	G→A	caggcttttg	22.02	1.95	15.16	1.41	5.82	10^{-6}	A			
		rs1032778925:A	attggcctgt	G→A	gctaattgcc	38.42	2.67	29.64	3.10	4.13	10^{-3}	B			
		rs1038028727:A	gcggttcagg	C→A	cgtctcctgg	99.42	8.27	68.14	5.33	6.61	10^{-6}	A			
		rs1048565967:A	gggcgcgcta	G→A	gccggaaagc	60.40	5.77	32.90	2.93	9.30	10^{-6}	A			
		rs1060504202:A	tgggtacgct	G→A	tgagtcgtct	54.79	3.95	21.09	1.79	17.17	10^{-6}	A			
		rs1064795596:A	agtcgtctgt	G→A	tctcccgcct	54.79	3.95	14.72	1.60	20.18	10^{-6}	A			
		rs1161871796:T	gcgcggttca	G→T	tccgtctcct	54.79	3.95	37.08	2.98	7.23	10^{-6}	A			
		rs1164643036:T	gggaggtaat	C→T	ctagagctcg	37.53	3.97	30.18	1.95	3.51	10^{-3}	B			
		rs1205641254:G	gcgggaggta	A→G	cgctagagct	37.53	3.97	27.85	3.06	3.91	10^{-3}	B			
		rs1246360154:A	gcggggtctc	G→A	tcggggatgg	99.42	8.27	75.72	5.86	4.79	10^{-3}	B			
		rs1246376784:A	agtcggggat	G→A	gcggttcagg	99.42	8.27	71.47	6.97	5.15	10^{-6}	A			
		rs1248252264:A	gttcagacgt	C→A	gtcgaacttc	48.21	3.91	17.19	1.67	16.27	10^{-6}	A			
		rs1273207352:A	gtcgccgatg	G→A	ctcgccgtcg	99.42	8.27	46.32	3.19	14.15	10^{-6}	A			
		rs1293817589:A	gagccgcggt	G→A	ttttgcagcc	22.02	1.95	6.93	0.68	17.43	10^{-6}	A			
		rs1307006679:A	aaagccgggc	G→A	gagtgcgccg	60.40	5.77	41.33	3.81	5.71	10^{-6}	A			
		rs1311829327:A	tgagtcgtct	G→A	cgtctcccgc	54.79	3.95	18.90	1.69	18.55	10^{-6}	A			
		rs1338038953:A	cttcttttag	G→A	agcagtgttc	17.07	1.48	8.19	0.77	11.49	10^{-6}	A			
		rs1362031886:C	gagctcgcgg	G→C	cgggcgcgct	37.53	3.97	30.06	3.15	2.98	10^{-2}	C			
		rs1362031886:T	gagctcgcgg	G→T	cgggcgcgct	37.53	3.97	16.17	1.42	12.25	10^{-6}	A			
		rs1370783231:T	ctccagacta	C→T	accgttatga	9.40	1.12	3.51	0.41	11.80	10^{-6}	A			
		rs1384587477:A	cgctagagct	C→A	aaagccgggc	60.40	5.77	35.57	3.13	8.15	10^{-6}	A	↑	within comparative human disease models using Mutyh-deficient mice versus normal mice [294]: oxidative activation of microglia increases with increasing levels of Mutyh, leading to retinal degeneration as a progression of POAG [295]	
		rs1384587477:G	cgctagagct	C→G	aaagccgggc	60.40	5.77	47.14	4.85	3.53	10^{-3}	B			
		rs1436893723:T	ggtcgaactt	C→T	ggtcgccgat	99.42	8.27	77.74	5.85	4.38	10^{-3}	B			
		rs1456848545:A	ctcgcgggag	G→A	gcgcgctaga	37.53	3.97	18.91	1.66	9.96	10^{-6}	A			
		rs1456848545:T	ctcgcgggag	G→T	gcgcgctaga	37.53	3.97	15.26	1.09	14.10	10^{-6}	A			
		rs1553127879:T	gtgttccctt	C→T	gcttcacagc	17.07	1.48	9.97	0.83	8.97	10^{-6}	A			
		rs1553136984:A	tcgtctgtgg	G→A	tcccgcctga	54.79	3.95	37.90	3.53	6.26	10^{-6}	A			
		rs1553137062:A	caccgctcgt	C→A	ggcggccatg	54.79	3.95	34.42	3.20	7.90	10^{-6}	A			
		rs1557517332:A	tcctggcgcg	G→A	tgcggtccgt	99.42	8.27	61.51	4.52	8.65	10^{-6}	A			
		rs1570584160:A	gcgcgctaga	G→A	cggaaagccg	60.40	5.77	18.71	1.85	17.03	10^{-6}	A			
		rs1570591700:A	ccgcctgagt	C→A	ccgctcgtct	54.79	3.95	17.22	1.74	18.62	10^{-6}	A			
		rs1570591700:T	ccgcctgagt	C→T	ccgctcgtct	54.79	3.95	27.89	1.99	13.32	10^{-6}	A			
		rs1570591736:A	tcccgcctga	G→A	caccgctcgt	54.79	3.95	37.40	2.27	8.10	10^{-6}	A			
		rs1645059514:T	tgcttcacag	C→T	caaaggagct	17.07	1.48	9.21	0.73	10.53	10^{-6}	A			
		rs1646739460:G	ggtaatcttt	C→G	agctcgcggg	37.53	3.97	30.59	2.72	2.96	10^{-2}	C			
		rs1646782056:T	tccgttcaga	C→T	tgggtcgaac	48.21	3.91	27.92	2.09	9.89	10^{-6}	A			
		rs1646783847:T	tcgaacttcc	G→T	tcgccgatgg	48.21	3.91	23.56	1.91	12.50	10^{-6}	A			
		rs1646788230:T	ctcgccgtcg	G→T	tggggcgggg	99.42	8.27	68.13	5.60	6.46	10^{-6}	A			
		rs1646796858:A	ttcaggcagt	C→A	tcctggcgcg	99.42	8.27	35.23	3.09	17.16	10^{-6}	A			
		rs1646798945:T	tggcgcggtt	C→T	ggtccgtctc	54.79	3.95	29.98	2.98	9.83	10^{-6}	A			
		rs2275602:T	gtacgctgga	C→T	gtcgtctgtg	54.79	3.95	41.49	3.62	4.91	10^{-6}	A			

Table S3. Cont.

#	Human Gene: *NCBI Gene Symbol* (NCBI Gene ID)	Candidate SNP marker: dbSNP ID:min [437]	5′ flank, 10 bp	WT →min	3 flank, 10 bp	K_D, nM, *in silico*: WT MEAN	± SEM	min MEAN	± SEM	Significance: Z	p	ϱ	Δ	Effect of changes in human gene expression on the development of primary open-angle glaucoma (POAG, ☼: "▼" aggravation, "▲" alleviation) [Reference]	☼ ▲ ▼
93	*MUTYH* (4595)	rs368538538:A	cgcggttcag	G→A	ccgtctcctg	54.79	3.95	35.01	3.04	7.93	10^{-6}	A	↑	within comparative human disease models using Mutyh-deficient mice versus normal mice [294]: oxidative activation of microglia increases with increasing levels of Mutyh, leading to retinal degeneration as a progression of POAG [295]	▼
		rs375770248:A	aggcagtcgg	G→A	tggcgcggtt	99.42	8.27	53.53	3.92	11.17	10^{-6}	A			
		rs545112449:A	ggcggggtct	C→A	gtcggggatg	99.42	8.27	82.34	7.24	3.11	10^{-2}	C			
		rs587788237:A	ccgctcgtct	C→A	cggccatgac	54.79	3.95	30.73	2.96	9.61	10^{-6}	A			
		rs747567943:T	ggggtgtctg	C→T	agggtgcttc	17.07	1.48	10.91	1.04	6.96	10^{-6}	A			
		rs753502884:T	cgtctcccgc	C→T	atgacaccgc	54.79	3.95	41.76	2.42	5.87	10^{-6}	A			
		rs755914972:A	ctcctggcgc	G→A	ttgcggtccg	99.42	8.27	68.48	5.20	6.62	10^{-6}	A			
		rs755914972:T	ctcctggcgc	G→T	ttgcggtccg	99.42	8.27	67.65	4.97	6.94	10^{-6}	A			
		rs755928199:A	cgcctgagtc	G→A	cgctcgtctc	54.79	3.95	19.48	1.88	17.14	10^{-6}	A			
		rs755928199:C	cgcctgagtc	G→C	cgctcgtctc	54.79	3.95	32.71	3.75	7.61	10^{-6}	A			
		rs755928199:T	cgcctgagtc	G→T	cgctcgtctc	54.79	3.95	21.96	1.87	16.40	10^{-6}	A			
		rs758246147:A	agtgttccct	T→A	tgcttcacag	17.07	1.48	9.45	0.94	8.97	10^{-6}	A			
		rs766566103:T	ggcagtcggg	G→T	ggcgcggttc	99.42	8.27	30.82	2.97	18.40	10^{-6}	A			
		rs766584437:A	gggtacgctg	G→A	gagtcgtctg	54.79	3.95	38.16	3.14	6.61	10^{-6}	A			
		rs766584437:T	gggtacgctg	G→T	gagtcgtctg	54.79	3.95	25.68	2.36	12.96	10^{-6}	A			
		rs767402084:A	gtcgtctgtg	G→A	ctcccgcctg	54.79	3.95	20.61	1.87	16.88	10^{-6}	A			
		rs767402084:C	gtcgtctgtg	G→C	ctcccgcctg	54.79	3.95	37.84	4.24	5.55	10^{-6}	A			
		rs769313208:A	aggggtgtct	G→A	aagggtgctt	17.07	1.48	8.07	0.80	11.39	10^{-6}	A			
		rs770735105:A	ccgcggtgta	C→A	tgcagccgga	22.02	1.95	12.58	1.08	9.05	10^{-6}	A			
		rs774530388:T	tctgtgggta	C→T	cgcctgagtc	54.79	3.95	45.59	4.12	3.18	10^{-2}	C			
		rs778337668:A	ggtccgtctc	C→A	gctggacttg	99.42	8.27	60.36	4.17	9.23	10^{-6}	A			
		rs779818394:T	ctctgcttca	C→T	ctgcaaagga	17.07	1.48	8.71	0.72	11.21	10^{-6}	A			
		rs876658588:A	ctgtgggtac	G→A	gcctgagtcg	54.79	3.95	26.38	2.74	11.55	10^{-6}	A			
		rs878854188:G	cctgagtcgt	C→G	ctcgtctccc	54.79	3.95	36.43	2.66	7.95	10^{-6}	A			
		rs888185369:A	aagccgggcg	C→A	agtgcgccgg	60.40	5.77	52.30	5.43	2.04	0.05	D			
		rs888185369:T	aagccgggcg	C→T	agtgcgccgg	60.40	5.77	45.89	4.34	4.08	10^{-3}	B			
		rs929692534:G	cggtgtacaa	C→G	agccggagcc	22.02	1.95	17.18	1.50	3.99	10^{-3}	B			
		rs929692534:T	cggtgtacaa	C→T	agccggagcc	22.02	1.95	16.95	1.41	4.31	10^{-3}	B			
94	*MYOC* (4653)	rs1269839734:C	ggctccccag	T→C	catgaagggc	0.84	0.08	1.09	0.16	2.99	10^{-2}	C	↓	within a pharmaceutical research [296]: small molecule MYOC-inhibitors (e.g., GW5074 and apigenin) reduced pathogenic MYOC aggregation that can enhance normogenic MYOC secretion and thus alleviate POAG	▲
		rs1653388728:C	tccccagtat	A→C	gaagggctgg	0.84	0.08	4.14	0.52	20.08	10^{-6}	A			
		rs1653388728:G	tccccagtat	A→G	gaagggctgg	0.84	0.08	2.15	0.23	12.94	10^{-6}	A			
		rs945704121:C	ctccccagta	T→C	tgaagggctg	0.84	0.08	1.66	0.19	9.27	10^{-6}	A			
95	*NCKAP5* (344148)	rs12616987:G	ggggattctt	A→G	ggaaacagac	3.38	0.32	6.77	0.57	11.01	10^{-6}	A	↓	according to the case report of a 69-year-old man [298]: suprachoroidal effusion aggravated surgery for POAG, most likely accompanied by NCKAP5 deficit, which is a biomedical molecular marker for non-small cell lung cancer as a well-known risk factor of suprachoroidal effusion [299], which, indeed, was in the above-mentioned 69-year-old man	▼
		rs1318674727:G	tccttttatg	T→G	tgattctttt	5.01	0.45	7.16	0.61	5.77	10^{-6}	A			
		rs1575108213:C	acatcccttt	T→C	attttcaaaa	5.46	0.41	6.56	0.41	3.74	10^{-3}	B			
		rs1684108651:T	ggattcttat	A→T	aaacagacgg	3.38	0.32	5.99	0.49	9.21	10^{-6}	A			
		rs1684108911:C	gggattctta	T→C	gaaacagacg	3.38	0.32	6.77	0.57	11.01	10^{-6}	A			
		rs1684996145:A	tccctttca	T→A	ttcaaaaaac	5.46	0.41	6.56	0.41	3.74	10^{-3}	B			
		rs1688729112:C	tttttgacag	T→C	cccctccttt	5.08	0.45	5.79	0.52	2.08	0.05	D			
		rs370087547:C	ttatcctttt	A→C	ccatgattct	5.01	0.45	8.19	0.70	7.95	10^{-6}	A			
		rs370087547:G	ttatcctttt	A→G	ccatgattct	5.01	0.45	7.50	0.65	6.49	10^{-6}	A			
		rs755331260:C	tttatccttt	T→C	tccatgattc	5.01	0.45	8.10	0.70	7.77	10^{-6}	A			
		rs918859537:C	tatcctttta	T→C	catgattctt	5.01	0.45	7.52	0.66	6.53	10^{-6}	A			

Table S3. Cont.

Human Gene		Candidate SNP marker				K_D, nM, *in silico*				Significance				Effect of changes in human gene expression on the development of primary open-angle glaucoma (POAG, ☼: "▼" aggravation, "▲" alleviation) [Reference]	☼ ▲ ▼
#	*NCBI Gene Symbol* (NCBI Gene ID)	dbSNP ID:min [437]	5′ flank, 10 bp	WT →min	3 flank, 10 bp	WT MEAN	WT ± SEM	min MEAN	min ± SEM	Z	p	ϱ	Δ		
		rs1182347665:T	aaagactcaa	C→T	agtcagttaa	5.08	0.45	4.45	0.39	2.15	0.05	D			
		rs1241326202:T	ttgacagtca	G→T	ctcctttctt	5.08	0.45	3.93	0.33	4.23	10^{-3}	B			
		rs1355019500:C	gattcttata	A→C	aacagacggg	3.38	0.32	2.37	0.25	5.06	10^{-6}	A			
		rs1381497852:A	gacgggggat	T→A	ccctggaaac	3.38	0.32	2.32	0.30	4.78	10^{-3}	B			
95	*NCKAP5* (344148)	rs1684107457:T	cacaaaagaa	A→T	ttcttataag	3.38	0.32	2.19	0.22	6.35	10^{-6}	A	↑	according to the case report of a 68-year-old woman [300]: after surgery for POAG, Staphylococcus aureus caused infective necrotizing scleritis, most likely accompanied by NCKAP5 upregulation because of hypo-methylation of the Nckap5 mouse gene was found in Staphylococcus aureus-induced mastitis as human disease models using mice [301]	▼
		rs1688729012:T	ttttgacagt	C→T	ccctcctttc	5.08	0.45	3.92	0.27	4.64	10^{-3}	B			
		rs1691078475:A	atcctttttat	G→A	atgattcttt	5.01	0.45	2.23	0.23	12.00	10^{-6}	A			
		rs920895087:A	gagcttgtat	T→A	ctttgtccac	5.08	0.45	2.65	0.38	7.76	10^{-6}	A			
		rs920895087:G	gagcttgtat	T→G	ctttgtccac	5.08	0.45	4.27	0.55	2.21	0.05	D			
		rs1015191291:C	cgcgtgggtg	A→C	gcccgggaca	55.26	3.93	83.09	5.77	8.21	10^{-6}	A			
		rs1051667903:A	gggtgaagag	C→A	ggacatcgcg	55.26	3.93	61.65	4.39	2.17	0.05	D			
		rs1339255364:G	gcatggggtg	A→G	ctctctggat	1.74	0.18	2.03	0.20	2.16	0.05	D			
		rs1597560537:C	gcgtgggtga	A→C	cccgggacat	55.26	3.93	83.09	5.77	8.21	10^{-6}	A			
		rs1597560537:G	gcgtgggtga	A→G	cccgggacat	55.26	3.93	83.09	5.69	8.26	10^{-6}	A			
		rs1909267196:C	ccgagcgata	A→C	ggctgcaggc	9.94	0.86	17.49	1.76	8.51	10^{-6}	A	↓	within a phytopharmaceutical study [302]: resveratrol as natural phytoantioxidant with antibacterial and antifungal protective properties can suppress NOS2 to medicate POAG	▲
		rs1909267317:G	tccgagcgat	A→G	gggctgcagg	9.94	0.86	38.37	2.88	23.61	10^{-6}	A			
96	*NOS2* (4843)	rs1909267435:A	ctccgagcga	T→A	ggggctgcag	9.94	0.86	44.27	4.04	23.76	10^{-6}	A			
		rs1909497090:G	gtgagtataa	A→G	gatggcatgg	1.74	0.18	2.12	0.24	2.59	10^{-2}	C			
		rs986613855:C	tgggtgaaga	G→C	gggacatcgc	55.26	3.93	61.96	4.56	2.24	0.05	D			
		rs1010042298:T	tgcaggctcc	G→T	ggggcggggg	9.94	0.86	8.45	0.73	2.66	10^{-2}	C			
		rs1261808037:T	acatcgcgtg	G→T	ctcggcccgg	55.26	3.93	18.32	1.43	20.89	10^{-6}	A			
		rs1299794842:A	tcgcgtgggt	G→A	ggcccgggac	55.26	3.93	16.67	1.44	21.43	10^{-6}	A	↑	within cohort-based iris sample postmortem study [303]: both excess and hyperactivity of NOS2 can be a biomedical molecular marker for susceptibility to POAG	▼
		rs1663071985:T	cgggacatcg	C→T	gcagctcggc	55.26	3.93	48.09	3.59	2.70	10^{-2}	C			
		rs976252980:T	ctcggcccgg	G→T	aggaggggca	55.26	3.93	19.09	2.33	15.08	10^{-6}	A			
		rs1028318654:C	attggcaggg	T→C	aagtagtttg	12.74	1.07	16.63	1.89	3.76	10^{-3}	B			
		rs1028318654:G	attggcaggg	T→G	aagtagtttg	12.74	1.07	18.09	1.94	5.13	10^{-6}	A			
		rs1177079858:C	tgtttactga	A→C	actaggggca	11.08	1.17	13.19	1.50	2.24	0.05	D	↓	within human disease models using NOS3 knockout mice [304]: higher risks of both hypertension and atherosclerosis, while both atherosclerosis [143] and hypertension [9] can aggravate POAG	▼
		rs1432795270:G	caggcgcctc	A→G	ctagggcgac	38.89	4.03	110.90	10.05	15.22	10^{-6}	A			
		rs1008295323:T	tcccctccct	C→T	ttcctaagga	28.18	2.66	20.13	1.90	5.04	10^{-6}	A			
		rs1168973228:T	ccaggcgcct	C→T	actagggcga	38.89	4.03	8.83	1.04	18.88	10^{-6}	A			
		rs1223238088:T	agacgaagag	A→T	acatgaaagt	11.08	1.17	7.90	0.65	5.08	10^{-6}	A			
		rs1393289189:A	cgcctcacta	G→A	ggcgaccccct	38.89	4.03	23.03	2.04	7.69	10^{-6}	A			
		rs1397768771:T	ctcactaggg	C→T	gacccctggt	38.89	4.03	33.90	2.74	2.09	0.05	D			
97	*NOS3* (4846)	rs145555620:T	cctcactagg	G→T	cgacccctgg	38.89	4.03	29.34	2.31	4.33	10^{-3}	B			
		rs1802244456:A	ggattggcag	G→A	gtaagtagtt	12.74	1.07	9.19	0.84	5.27	10^{-6}	A			
		rs1802305214:G	ctccctcttc	C→G	taaggaaaag	28.18	2.66	15.18	1.73	8.37	10^{-6}	A	↑	within a cohort-based biomedical study [305]: positive therapeutic effects of mindfulness meditation on intraocular pressure and trabecular meshwork in patients in India with medically uncontrolled primary open angle glaucoma was accompanied by NOS3 upregulation	▲
		rs1802305214:T	ctccctcttc	C→T	taaggaaaag	28.18	2.66	9.58	0.81	17.01	10^{-6}	A			
		rs545164063:G	gtaagtagtt	T→G	ggcaggaagg	12.74	1.07	10.29	0.88	3.55	10^{-3}	B			
		rs546143732:T	ctaggagggg	G→T	ttgtttactg	11.08	1.17	8.96	0.85	3.01	10^{-2}	C			
		rs567664983:A	aggcgcctca	C→A	tagggcgacc	38.89	4.03	23.33	2.17	7.34	10^{-6}	A			
		rs752962923:A	gcctcactag	G→A	gcgacccctg	38.89	4.03	26.35	2.21	5.85	10^{-6}	A			
		rs769213623:A	tcttcctaag	G→A	aaaaggccag	28.18	2.66	14.94	1.08	10.67	10^{-6}	A			
		rs964032879:A	aagtagtttg	G→A	caggaaggac	12.74	1.07	8.49	0.60	7.37	10^{-6}	A			

Table S3. Cont.

Human Gene		Candidate SNP marker				K_D, nM, *in silico*				Significance				Effect of changes in human gene expression on the development of primary open-angle glaucoma (POAG, ☼: "▼" aggravation, "▲" alleviation) [Reference]	☼ ▲ ▼
						WT		min							
#	*NCBI Gene Symbol* (NCBI Gene ID)	dbSNP ID:min [437]	5′ flank, 10 bp	WT →min	3 flank, 10 bp	MEAN	± SEM	MEAN	± SEM	Z	p	ϱ	Δ		
98	*NRG2* (9542)	rs1036285416:C	ggcgctaacg	T→C	gagggctaag	19.04	1.39	33.26	2.90	9.82	10^{-6}	A	↓	within human disease models using Nrg2-knockout mice [306]: reduced reproductive capacity and early growth retardation, while in dogs artificial selection for short stature may have contributed to increased prevalence of POAG [307]	▼
		rs1284121842:C	ctaacgttac	G→C	gctaagaggc	19.04	1.39	24.19	1.65	4.79	10^{-3}	B			
		rs1669833888:G	gtgaccgcct	A→G	ccccggaggg	3.58	0.38	10.87	0.94	16.19	10^{-6}	A			
		rs1762107606:C	aacgttacgc	T→C	taagaggcgc	19.04	1.39	24.14	1.58	4.84	10^{-3}	B			
		rs927542613:G	cgcgctccct	A→G	cctgcagaag	44.48	3.88	56.53	3.98	4.28	10^{-3}	B			
		rs964956302:A	gaggcgctaa	C→A	tggagggcta	19.04	1.39	27.46	2.09	6.95	10^{-6}	A			
		rs1199244862:A	gctccctact	C→A	gcagaagccg	44.48	3.88	10.57	1.36	18.52	10^{-6}	A	↑	within human neurodegenerative disease models using rat pups subjected with an interference-RNA-caused delaying neurogenesis, an, next, treated with retrovirus carrying an additional copy of the rat Nrg2 gene [308]: Nrg2 excess along rescued the impairments of dendritic arborization, whereas this repairative neurogenesis can alleviate the post-COVID-infection effects on POAG [309]	▲
		rs1199244862:T	gctccctact	C→T	gcagaagccg	44.48	3.88	22.00	2.39	10.10	10^{-6}	A			
		rs1212994481:T	gctgtttccg	G→T	gctaacgtta	57.75	4.27	44.59	3.11	5.10	10^{-6}	A			
		rs1334921794:A	gcggagcccc	C→A	gcggctgagc	57.75	4.27	19.72	1.44	20.72	10^{-6}	A			
		rs1361448898:T	ttccggtttt	C→T	cgttacgctg	57.75	4.27	30.46	2.60	11.33	10^{-6}	A			
		rs1427855432:A	ctccctactc	G→A	cagaagccgc	44.48	3.88	25.02	2.79	8.14	10^{-6}	A			
		rs1469860963:A	ggcggagccc	C→A	ggcggctgag	57.75	4.27	43.00	3.13	5.69	10^{-6}	A			
		rs1469860963:T	ggcggagccc	C→T	ggcggctgag	57.75	4.27	50.46	4.07	2.47	0.05	D			
		rs1762119592:A	gcgcgagaga	G→A	gggcagaggg	44.48	3.88	18.16	1.35	15.62	10^{-6}	A			
		rs1762121768:T	gccgcgctcc	C→T	cccctgcaga	44.48	3.88	22.22	1.78	11.72	10^{-6}	A			
		rs369753660:T	cggagccccc	C→T	cggctgagcg	57.75	4.27	16.85	1.43	21.90	10^{-6}	A			
		rs752263293:T	ccagcgggct	C→T	tttccggttt	57.75	4.27	36.71	2.60	8.85	10^{-6}	A			
99	*NTF4* (4909)	rs1023226050:T	ggtcccggca	C→T	ctggcttcca	72.65	5.27	83.10	6.56	2.51	0.05	D	↓	according to comprehensive review [310]: many POAG-related NTF4-mutation experiments in vitro have indicated that neurotrophin-4 failure can contribute to POAG pathogenesis	▼
		rs1215958394:C	cggcccctcc	T→C	gcaggatccc	137.47	10.34	218.43	16.69	8.64	10^{-6}	A			
		rs1305292458:C	ggcacgcgag	T→C	tccaaggtcc	72.65	5.27	137.47	10.34	12.20	10^{-6}	A			
		rs1600252827:C	cggcgtcctg	G→C	cctcgggatt	133.23	10.29	154.94	10.84	2.90	10^{-2}	C			
		rs2040168261:T	tcaagtgatt	G→T	acctcctggg	31.72	2.28	39.28	2.87	4.17	10^{-3}	B			
		rs1017358588:G (transcript NTF4-203)	cccggcccct	C→G	aggcaggatc	137.47	10.34	159.07	11.85	2.76	10^{-2}	C			
		rs1017358588:A (transcript NTF4-207)	cccggcccct	C→A	aggcaggatc	72.65	5.27	41.50	3.25	10.49	10^{-6}	A	↑	within human cellular POAG-related post-traumatic wound healing models using telomerase-immortalized human corneal epithelial cell line hTCEpi between passages 20 and 28 [311]: activated stromal fibroblast conditioned medium can elevate NTF4 level to repair eye injuries	▲
		rs1018712710:T	gatctccact	C→T	gccccaggta	133.23	10.29	80.39	5.77	9.59	10^{-6}	A			
		rs1026630873:A	cccaccacaa	C→A	ttcaatctca	31.72	2.28	25.56	2.09	3.97	10^{-3}	B			
		rs1031777123:A	tcctctgccg	C→A	cccccggccc	137.47	10.34	104.94	7.53	5.19	10^{-6}	A			
		rs1031777123:T	tcctctgccg	C→T	cccccggccc	137.47	10.34	98.97	7.51	6.15	10^{-6}	A			
		rs1268413255:A	cccctcctct	G→A	ggatcccccg	72.65	5.27	35.73	3.41	11.84	10^{-6}	A			
		rs1268961095:T	ccctcgggtg	G→T	tggtcgccct	133.23	10.29	86.62	6.99	7.71	10^{-6}	A			
		rs1301631025:A	ggaggaaact	G→A	cggggcttgg	62.87	4.31	22.90	1.82	19.23	10^{-6}	A			
		rs1332019193:A	cttccaaggt	C→A	gccaggcctg	72.65	5.27	49.23	4.33	6.83	10^{-6}	A			
		rs1350272835:A	cctcctccct	C→A	agatctccac	133.23	10.29	81.45	7.28	8.33	10^{-6}	A			
		rs1370006783:A	aggaaactga	G→A	gggcttggcg	62.87	4.31	56.50	4.25	2.10	0.05	D			
		rs1394061779:A	cacctcctgg	G→A	accacaacct	31.72	2.28	27.01	1.77	3.30	10^{-3}	B			
		rs1440668027:A	gcttggcgga	G→A	accggggcgg	62.87	4.31	44.91	2.82	7.23	10^{-6}	A			
		rs1447581466:A	gcgagtgcaa	G→A	ggtcccggca	72.65	5.27	45.54	3.18	9.27	10^{-6}	A			
		rs1467489020:T	tcccccggcc	C→T	gcaaggcagg	137.47	10.34	113.30	8.49	3.64	10^{-3}	B			
		rs2040168297:G	ggttcaagtg	A→G	tccacctcct	31.72	2.28	28.33	2.01	2.23	0.05	D			
		rs2040198021:A	gccctcgggt	G→A	ctggtcgccc	133.23	10.29	72.43	6.89	9.95	10^{-6}	A			
		rs2040198043:A	ccctgccctc	G→A	cgtcctggtc	133.23	10.29	95.76	6.87	6.26	10^{-6}	A			
		rs2040198080:T	gtcctggtcg	C→T	gggattccgg	133.23	10.29	86.90	6.30	8.07	10^{-6}	A			
		rs2040198135:A	gcgtcctggt	C→A	tcgggattcc	133.23	10.29	42.20	4.23	18.18	10^{-6}	A			

Table S3. Cont.

Human Gene		Candidate SNP marker				K$_D$, nM, *in silico*				Significance				Effect of changes in human gene expression on the development of primary open-angle glaucoma (POAG, ☼: "▼" aggravation, "▲" alleviation) [Reference]	☼
#	*NCBI Gene Symbol* (NCBI Gene ID)	dbSNP ID:min [437]	5′ flank, 10 bp	WT →min	3 flank, 10 bp	WT MEAN	WT ± SEM	min MEAN	min ± SEM	Z	p	ϱ	Δ		▲▼
99	*NTF4* (4909)	rs2040200710:A	ctcctctgcc	G→A	tcccccggcc	137.47	10.34	107.24	8.41	4.57	10^{-3}	B	↑	within human cellular POAG-related post-traumatic wound healing models using telomerase-immortalized human corneal epithelial cell line hTCEpi between passages 20 and 28 [311]: activated stromal fibroblast conditioned medium can elevate NTF4 level to repair eye injuries	▲
		rs2040200710:T	ctcctctgcc	G→T	tcccccggcc	137.47	10.34	119.44	8.91	2.65	10^{-2}	C			
		rs2040200769:A	cctcctctgc	C→A	atcccccggc	137.47	10.34	112.64	8.71	3.69	10^{-3}	B			
		rs2040200834:T	ccggcccctc	C→T	ggcaggatcc	72.65	5.27	49.87	3.69	7.26	10^{-6}	A			
		rs2040200882:T	cccccggccc	C→T	caaggcagga	137.47	10.34	98.07	7.22	6.41	10^{-6}	A			
		rs2040201118:A	tgcaaggcag	G→A	cggcacgcga	137.47	10.34	103.58	7.54	5.41	10^{-6}	A			
		rs2040201146:A	cacgcgagtg	C→A	caaggtcccg	72.65	5.27	32.36	2.22	16.18	10^{-6}	A			
		rs2040201146:T	cacgcgagtg	C→T	caaggtcccg	72.65	5.27	15.53	1.41	26.58	10^{-6}	A			
		rs2040201244:A	cccggcacgc	G→A	gcttccaagg	72.65	5.27	53.52	3.62	6.16	10^{-6}	A			
		rs560217335:A	tccacctcct	G→A	ccaccacaac	31.72	2.28	20.52	1.66	8.04	10^{-6}	A			
		rs573996923:T	cctcgggatt	C→T	cactcctcct	133.23	10.29	85.30	6.35	8.31	10^{-6}	A			
		rs910245578:A	ccggcgtcct	G→A	ccctcgggat	133.23	10.29	32.82	2.94	23.68	10^{-6}	A			
		rs910245578:T	ccggcgtcct	G→T	ccctcgggat	133.23	10.29	43.77	3.41	20.29	10^{-6}	A			
		rs922917315:T	gcacgcgagt	G→T	ccaaggtccc	72.65	5.27	43.00	2.84	10.70	10^{-6}	A			
		rs943037252:A	ctcgggattc	C→A	actcctcctc	133.23	10.29	76.18	5.48	10.59	10^{-6}	A			
		rs975733124:A	gagtgcaagg	C→A	tcccggcacg	72.65	5.27	59.28	4.15	4.03	10^{-3}	B			
		rs975733124:T	gagtgcaagg	C→T	tcccggcacg	137.47	10.34	56.11	5.01	15.35	10^{-6}	A			
100	*NTM* (50863)	rs1327147045:A	ggcaaagtgg	C→A	atttatcatt	7.15	0.61	8.74	0.79	3.24	10^{-2}	C	↓	within a cohort-based biomedical microarray study [312]: NTM-deficit seems to be a biomedical molecular marker for ischemic diseases, which occur significantly more often in patients with POAG than without it [313]	▼
		rs1480913173:G	cccatctgct	A→G	ttgtttccga	23.03	1.59	31.78	2.59	6.04	10^{-6}	A			
		rs1490573003:C	ggccagattg	T→C	acaatgtgtc	11.09	0.83	13.84	1.00	4.26	10^{-3}	B			
		rs181300802:G	tggctttcct	A→G	atagttctaa	4.67	0.44	10.14	0.72	13.05	10^{-6}	A			
		rs2054787657:C	tcccatctgc	T→C	attgtttccg	23.03	1.59	50.83	3.64	15.93	10^{-6}	A			
		rs2458764:C	ctttcctaat	A→C	gttctaattt	4.67	0.44	17.22	1.06	23.06	10^{-6}	A			
		rs768742863:C	agatccacca	T→C	acatttcaga	10.61	1.02	18.01	1.85	7.52	10^{-6}	A			
		rs920674456:A	tttcctaata	G→A	ttctaatttc	4.67	0.44	6.58	0.59	5.24	10^{-6}	A			
		rs962828988:A	gctttcctaa	T→A	agttctaatt	4.67	0.44	12.89	1.07	16.06	10^{-6}	A			
		rs998127238:C	cccacttcct	G→C	tgctcgcccg	60.02	4.26	69.75	5.25	2.91	10^{-2}	C			
		rs1234482347:T	tattgtttcc	G→T	attgttttcc	23.03	1.59	20.48	1.41	2.42	0.05	D	↑	within women disease models using adult virgin female Spague-Dawley rats [314]: estrogen upregulates Ntm along with neuroprotection for retinal ganglion cells within the optic nerve that can alleviate POAG as an additional benefit during maintenance postmenopausal estrogen-based therapy according to comprehensive review [315]	▲
		rs1269751113:A	caagggtaca	G→A	gtgtccccgt	10.61	1.02	6.53	0.66	6.93	10^{-6}	A			
		rs1271083527:G	agggagaaag	A→G	caagatattc	12.21	0.97	10.21	0.86	3.08	10^{-2}	C			
		rs1375989187:T	gctcctgtct	C→T	tttaatcagc	7.15	0.61	6.32	0.43	2.27	0.05	D			
		rs1430372743:A	caaaggagta	G→A	aacgatgaat	20.95	1.62	17.81	1.46	2.89	10^{-2}	C			
		rs1446828103:A	cggggggcgt	G→A	tgccgtgcgg	60.02	4.26	46.59	5.90	3.49	10^{-3}	B			
		rs1477700828:A	gaccacaaag	G→A	agtagaacga	20.95	1.62	18.83	1.19	2.14	10^{-6}	A			
		rs1487945593:T	accccaccca	C→T	ttcctgtgct	60.02	4.26	50.37	3.24	3.66	10^{-3}	B			
		rs1565518438:A	ggcttcaagc	G→A	ctaaaacctt	20.95	1.62	8.97	0.82	14.16	10^{-6}	A			
		rs1565518438:T	ggcttcaagc	G→T	ctaaaacctt	20.95	1.62	12.40	1.32	7.99	10^{-6}	A			
		rs1566293472:C	tcagtccctt	G→C	acgtacctgt	12.21	0.97	10.02	1.01	3.07	10^{-2}	C			
		rs1592772722:T	cacccacttc	C→T	tgtgctcgcc	60.02	4.26	22.31	1.56	19.86	10^{-6}	A			
		rs1941024641:C	ggcatttatc	A→C	ttctatctct	7.15	0.61	6.13	0.53	2.54	0.05	D			
		rs1941036781:T	ccagattgta	C→T	aatgtgtctc	11.09	0.83	3.99	0.55	13.05	10^{-6}	A			
		rs1950801237:A	tcagaccact	G→A	agctcaagtt	20.95	1.62	17.18	1.39	3.55	10^{-3}	B			
		rs200094059:A	aggagtagaa	C→A	gatgaatcag	20.95	1.62	12.82	1.20	8.09	10^{-6}	A			
		rs2054788814:C	ccatctgcta	T→C	tgtttccgat	23.03	1.59	19.20	1.53	3.46	10^{-3}	B			

Table S3. Cont.

#	Human Gene: *NCBI Gene Symbol* (NCBI Gene ID)	Candidate SNP marker: dbSNP ID:min [437]	5′ flank, 10 bp	WT →min	3 flank, 10 bp	K_D, nM, *in silico*: WT MEAN	± SEM	min MEAN	± SEM	Significance: Z	p	ϱ	Δ	Effect of changes in human gene expression on the development of primary open-angle glaucoma (POAG, ☼: "▼" aggravation, "▲" alleviation) [Reference]	☼ ▲ ▼
100	*NTM* (50863)	rs2054789367:A	tgctattgtt	T→A	ccgattgttt	23.03	1.59	11.60	0.74	14.61	10^{-6}	A	↑	within women disease models using adult virgin female Spague-Dawley rats [314]: estrogen upregulates Ntm along with neuroprotection for retinal ganglion cells within the optic nerve that can alleviate POAG as an additional benefit during maintenance postmenopausal estrogen-based therapy according to comprehensive review [315]	▲
		rs2054789367:C	tgctattgtt	T→C	ccgattgttt	23.03	1.59	20.38	1.30	2.61	10^{-2}	C			
		rs2054790170:T	ctattgtttc	C→T	gattgttttc	23.03	1.59	18.40	1.27	4.62	10^{-3}	B			
		rs2054952200:A	ccacccactt	C→A	ctgtgctcgc	60.02	4.26	9.15	0.81	33.23	10^{-6}	A			
		rs2054959130:T	cctgtgctcg	C→T	ccggggggcg	60.02	4.26	51.33	3.82	3.04	10^{-2}	C			
		rs2070378807:T	acctccctct	G→T	atggctgctg	12.21	0.97	8.51	0.74	6.13	10^{-6}	A			
		rs2095237677:T	tttgcagatc	C→T	accatacatt	10.61	1.02	8.20	0.77	3.83	10^{-3}	B			
		rs373752573:A	gggggcgtgt	G→A	ccgtgcggct	60.02	4.26	31.87	5.67	6.61	10^{-6}	A			
		rs527873479:A	ttcctgtgct	C→A	gcccgggggg	60.02	4.26	27.91	2.45	13.58	10^{-6}	A			
		rs557114085:A	cccacccact	T→A	cctgtgctcg	60.02	4.26	20.48	2.65	14.59	10^{-6}	A			
		rs574253398:T	agaaagacaa	G→T	atattctagc	12.21	0.97	4.57	0.40	16.58	10^{-6}	A			
		rs745482971:A	cgcccggggg	G→A	cgtgtgccgt	60.02	4.26	42.47	3.56	6.30	10^{-6}	A			
		rs747602602:T	agtcccttga	C→T	gtacctgtct	12.21	0.97	7.57	0.71	7.79	10^{-6}	A			
		rs749199928:A	cttgacgtac	C→A	tgtctggtct	12.21	0.97	6.02	0.68	10.23	10^{-6}	A			
		rs755702161:A	gtcccttgac	G→A	tacctgtctg	12.21	0.97	8.52	0.91	5.41	10^{-6}	A			
		rs758141533:T	tggcttcaag	C→T	gctaaaacct	20.95	1.62	13.88	1.54	6.09	10^{-6}	A			
		rs765911985:T	ccccgtggga	C→T	aaaaggggac	10.61	1.02	6.88	0.59	6.74	10^{-6}	A			
		rs771015385:A	tgacgtacct	G→A	tctggtcttc	12.21	0.97	10.29	1.04	2.67	10^{-2}	C			
		rs776703734:T	atccaccata	C→T	atttcagaag	10.61	1.02	4.02	0.43	13.58	10^{-6}	A			
		rs908232193:T	gctccacttc	C→T	catctgctat	23.03	1.59	15.69	1.15	7.64	10^{-6}	A			
		rs916496220:T	caagcgctaa	A→T	accttcctga	20.95	1.62	11.61	1.24	8.97	10^{-6}	A			
		rs923273034:A	agcgctaaaa	C→A	cttcctgagg	20.95	1.62	14.97	1.28	5.85	10^{-6}	A			
		rs935196608:T	ggagtagaac	G→T	atgaatcagt	20.95	1.62	17.37	1.62	3.10	10^{-2}	C			
		rs949099349:T	cttcccatct	G→T	ctattgtttc	23.03	1.59	11.02	0.93	13.57	10^{-6}	A			
		rs976101415:A	gttctggctt	C→A	aagcgctaaa	20.95	1.62	14.74	1.12	6.50	10^{-6}	A			
		rs979380883:A	gagggcctga	C→A	cacaaaggag	20.95	1.62	16.60	1.30	4.23	10^{-3}	B			
		rs998127238:A	cccacttcct	G→A	tgctcgcccg	60.02	4.26	21.99	2.07	17.05	10^{-6}	A			
101	*NTRK2* (4915)	rs1183656665:C	gttgcgcatc	T→C	ggcgccagag	70.15	5.45	115.91	8.92	9.19	10^{-6}	A	↓	according to exhaustive review [316]: compared to normal, Ntrk2 knockout mice have accelerated photoreceptor degeneration and loss of retinal ganglion cells, as well as more severe damage to the optic nerve	▼
		rs1269866360:C	tgtgcgcgcg	T→C	gtgtgaactc	31.09	2.56	37.70	3.43	3.14	10^{-2}	C			
		rs1306374803:A	cggagctggg	C→A	actcatccat	29.65	2.33	36.25	2.76	3.67	10^{-3}	И			
		rs1366256995:C	gatccccaaa	T→C	aggtggcccg	19.23	1.95	32.21	2.03	8.62	10^{-6}	A			
		rs1373953788:A	ctcttttaaa	T→A	gactgcggaa	5.24	0.53	12.29	1.09	12.69	10^{-6}	A			
		rs1383559621:A	tctggcgcca	G→A	agcgcgcccc	70.15	5.45	78.59	6.24	2.05	0.05	D			
		rs1412954523:C	tgtttgatac	T→C	gagataggca	9.71	0.88	13.59	1.14	5.44	10^{-6}	A			
		rs1424473615:C	cgctggttgc	G→C	catctggcgc	70.15	5.45	92.16	6.94	5.04	10^{-6}	A			
		rs1430233146:G	ccgatcccca	A→G	ataggtggcc	19.23	1.95	32.21	2.03	8.62	10^{-6}	A			
		rs1456088592:A	gagctgggca	C→A	tcatccatcc	29.65	2.33	42.26	3.49	6.22	10^{-6}	A			
		rs1564009527:G	tgctgctgtc	T→G	gcttctggcc	79.45	5.49	102.07	7.47	4.98	10^{-6}	A			
		rs1587798811:G	agccctcgga	A→G	gtgtcaggca	60.58	5.47	73.45	5.81	3.21	10^{-2}	C			
		rs1587801367:G	tcccgtcagg	T→G	taaaggatga	12.49	0.98	17.82	1.34	6.54	10^{-6}	A			
		rs2058496516:G	ccgctggttg	C→G	gcatctggcg	70.15	5.45	87.66	6.65	4.10	10^{-3}	B			
		rs2058503905:T	caccagagcc	C→T	tcggaagtgt	99.15	7.52	111.58	7.99	2.27	0.05	D			
		rs2058504864:G	ccccgttctc	T→G	tttaaatgac	5.24	0.53	8.15	0.57	7.17	10^{-6}	A			
		rs2058504932:C	cccgttctct	T→C	ttaaatgact	5.24	0.53	9.59	0.96	8.47	10^{-6}	A			
		rs2058564110:G	catgctgctg	C→G	tgtctgcttc	79.45	5.49	88.20	6.31	2.10	0.05	D			

Table S3. Cont.

#	Human Gene: NCBI Gene Symbol (NCBI Gene ID)	Candidate SNP marker: dbSNP ID:min [437]	5′ flank, 10 bp	WT →min	3 flank, 10 bp	K_D, nM, *in silico*: WT MEAN	± SEM	min MEAN	± SEM	Significance: Z	p	ϱ	Δ	Effect of changes in human gene expression on the development of primary open-angle glaucoma (POAG, ☼: "▼" aggravation, "▲" alleviation) [Reference]	☼ ▲▼
		rs374004883:G	ttgtgtgtgt	A→G	tgagcgtgtg	8.23	0.78	13.15	0.95	7.87	10^{-6}	A			
		rs544237847:T	gtgtttgata	C→T	tgagataggc	9.71	0.88	14.00	1.04	6.24	10^{-6}	A			
		rs747979248:C	tttgtgtgtg	T→C	atgagcgtgt	8.23	0.78	13.15	0.95	7.87	10^{-6}	A	↓	according to exhaustive review [316]: c ompared to normal, Ntrk2 knockout mice have accelerated photoreceptor degeneration and loss of retinal ganglion cells, as well as more severe damage to the optic nerve	▼
		rs776804640:G	tccccgttct	C→G	ttttaaatga	5.24	0.53	8.54	0.65	7.72	10^{-6}	A			
		rs957681584:C	ctggttgcgc	A→C	tctggcgcca	70.15	5.45	115.91	8.92	9.19	10^{-6}	A			
		rs1023302684:T	ggcgctctct	C→T	cccgcgcggg	129.77	10.33	112.95	8.58	2.52	0.05	D			
		rs1031980326:A	gggcgccgct	G→A	gttgcgcatc	70.15	5.45	46.84	4.23	6.78	10^{-6}	A			
		rs1050261680:T	tgctgctgct	G→T	tctgcttctg	79.45	5.49	41.78	2.98	12.95	10^{-6}	A			
		rs1169329027:A	tcaggttaaa	G→A	gatgagagaa	12.49	0.98	8.43	0.58	7.55	10^{-6}	A			
		rs1223218256:T	ccctcccctc	C→T	ctggtgcttt	79.45	5.49	62.85	4.79	4.56	10^{-3}	B			
		rs1292980866:A	tcaggcactg	C→A	ggtgtatttt	60.58	5.47	29.93	3.15	10.18	10^{-6}	A			
		rs1318230450:T	ctgtctgctt	C→T	tggccagtgg	79.45	5.49	68.20	5.45	2.89	10^{-2}	C			
		rs1343516146:T	tctgcttctg	G→T	ccagtggcac	79.45	5.49	34.77	2.66	16.02	10^{-6}	A			
		rs1359091145:A	aagtgtcagg	C→A	actgcggtgt	60.58	5.47	49.07	3.64	3.61	10^{-3}	B			
		rs1365970412:T	ttttggattt	C→T	agactaattt	8.23	0.78	6.04	0.59	4.57	10^{-3}	B			
		rs1383425332:T	ttggatttca	G→T	actaattttc	8.23	0.78	3.15	0.32	13.86	10^{-6}	A			
		rs1383559621:T	tctggcgcca	G→T	agcgcgcccc	70.15	5.45	60.65	7.10	2.07	0.05	D			
		rs1424473615:T	cgctggttgc	G→T	catctggcgc	70.15	5.45	47.18	3.23	7.67	10^{-6}	A			
		rs1450231231:A	ctggccagtg	G→A	caccgatgcc	79.45	5.49	42.59	4.14	10.46	10^{-6}	A			
		rs1481230871:T	gctctctccc	C→T	gcgcgggggc	129.77	10.33	103.64	8.40	3.96	10^{-3}	B			
		rs151306123:A	gccctcggaa	G→A	tgtcaggcac	99.15	7.52	78.68	6.49	4.13	10^{-3}	B			
101	*NTRK2* (4915)	rs1587807413:T	tgcgtgtgtg	C→T	gcgcgtgtgt	31.09	2.56	22.80	1.92	5.26	10^{-6}	A			
		rs2058498497:T	agcgcgcccc	C→T	agcgctgtgc	129.77	10.33	105.43	7.01	4.00	10^{-3}	B			
		rs2058498693:T	cgccccccagc	G→T	ctgtgcccgc	129.77	10.33	59.32	4.53	14.20	10^{-6}	A			
		rs2058500865:A	gccatgggct	G→A	gtgctccgga	129.77	10.33	52.96	4.78	14.89	10^{-6}	A	↑	within a cohort-based retrospective biomedical study [317]: total healing effect of drugs used to treat glaucoma associated with retinal TrkB upregulation as neuroprotector	▲
		rs2058501481:A	tccggagcgt	G→A	ccgggcgctc	129.77	10.33	66.86	6.99	10.09	10^{-6}	A			
		rs2058503534:A	ccgcgcgggg	G→A	caccagagcc	129.77	10.33	97.40	7.34	5.23	10^{-6}	A			
		rs2058503829:A	gggcaccaga	G→A	ccctcggaag	99.15	7.52	83.69	6.16	3.21	10^{-2}	C			
		rs2058503975:A	cagagccctc	G→A	gaagtgtcag	99.15	7.52	47.42	3.24	14.46	10^{-6}	A			
		rs2058503975:C	cagagccctc	G→C	gaagtgtcag	99.15	7.52	53.55	4.75	10.56	10^{-6}	A			
		rs2058504864:G	ccccgttctc	T→G	tttaaatgac	5.23	0.47	4.29	0.36	3.22	10^{-2}	C			
		rs2058523359:T	ggaatggcta	C→T	atcccgtcag	12.49	0.98	9.89	1.09	3.47	10^{-3}	B			
		rs2058523731:A	gctacatccc	G→A	tcaggttaaa	12.49	0.98	11.16	0.91	1.99	0.05	D			
		rs2058541443:A	accaccgatc	C→A	ccaaatagg t	19.23	1.95	16.58	1.57	2.14	0.05	D			
		rs2058563459:A	gtgtgaactc	C→A	cacatgctgc	31.09	2.56	19.47	1.54	8.18	10^{-6}	A			
		rs2058565219:A	tctggccagt	G→A	gcaccgatgc	79.45	5.49	37.12	4.01	11.87	10^{-6}	A			
		rs2058565550:T	gccagtggca	C→T	cgatgcctcc	79.45	5.49	57.55	4.54	6.15	10^{-6}	A			
		rs2058568622:T	ctgctcgccc	C→T	cagattcccc	79.45	5.49	62.80	4.50	4.72	10^{-3}	B			
		rs2058570254:A	tcccctccct	G→A	gtgcttttgt	79.45	5.49	31.04	2.73	16.80	10^{-6}	A			
		rs2058572224:A	gtttgtgtgt	G→A	tatgagcgtg	8.23	0.78	2.50	0.26	17.03	10^{-6}	A			
		rs536882405:T	tcggggtggg	G→T	atgacgcgag	29.65	2.33	22.10	2.23	4.60	10^{-3}	B			
		rs541777997:A	gcgctctctc	C→A	ccgcgcgggg	129.77	10.33	97.56	6.32	5.56	10^{-6}	A			
		rs541777997:T	gcgctctctc	C→T	ccgcgcgggg	129.77	10.33	98.77	7.41	4.99	10^{-6}	A			
		rs561541551:A	ctccccgcgc	G→A	ggggcaccag	99.15	7.52	88.59	6.75	2.10	0.05	D			

Table S3. Cont.

Human Gene		Candidate SNP marker				K_D, nM, *in silico*				Significance				Effect of changes in human gene expression on the development of primary open-angle glaucoma (POAG, ☼: "▼" aggravation, "▲" alleviation) [Reference]	☼ ▲ ▼
#	*NCBI Gene Symbol* (NCBI Gene ID)	dbSNP ID:min [437]	5′ flank, 10 bp	WT →min	3 flank, 10 bp	WT MEAN±	WT SEM	min MEAN±	min SEM	Z	p	ϱ	Δ		
101	*NTRK2* (4915)	rs768395947:G	gccgggcgct	C→G	tctccccgcg	129.77	10.33	96.66	7.70	5.23	10^{-6}	Φ	↑	within a cohort-based retrospective biomedical study [317]: total healing effect of drugs used to treat glaucoma associated with retinal TrkB upregulation as neuroprotector	▲
		rs768395947:T	gccgggcgct	C→T	tctccccgcg	129.77	10.33	54.93	4.09	15.77	10^{-6}	A			
		rs776804640:A	tccccgttct	C→A	ttttaaatga	5.23	0.47	3.81	0.37	4.79	10^{-3}	B			
		rs887499235:T	ggcaccgatg	C→T	ctccctcctc	79.45	5.49	50.63	4.20	8.34	10^{-6}	A			
		rs901798750:T	cgtgtgtgaa	C→T	tcccacatgc	31.09	2.56	21.38	1.83	6.30	10^{-6}	A			
		rs909829325:T	cgcgcggggg	C→T	accagagccc	129.77	10.33	41.41	4.04	18.14	10^{-6}	A			
		rs928170147:A	cctcctccct	G→A	ctcgccccca	79.45	5.49	55.81	4.88	6.34	10^{-6}	A			
		rs932815578:A	tttccttatt	G→A	tgtttgatac	8.01	0.72	4.49	0.33	9.96	10^{-6}	A			
		rs937920127:T	cgtgccgggc	G→T	ctctctcccc	129.77	10.33	95.34	7.15	5.64	10^{-6}	A			
		rs970489808:T	gcgacccggg	C→T	catgggctgg	129.77	10.33	97.23	7.00	5.38	10^{-6}	A			
102	*OGG1* (4968)	rs1052618606:G	gctccactgc	A→G	ctgtgtaccg	10.70	1.18	13.14	1.47	2.62	10^{-2}	C	↓	within a cohort-based biomedical study [137]: OGG1-deficit is a biomedical molecular marker of POAG because of the corresponding decrease in the level of excision repair of somatic pre-mutational DNA damage caused by oxidative stress	▼
		rs1324064169:C	catcgtactc	T→C	agcctccact	17.21	1.63	23.05	2.51	4.04	10^{-3}	B			
		rs144091991:C	ggcatcgtac	T→C	ctagcctcca	17.21	1.63	25.17	2.49	5.55	10^{-6}	A			
		rs2077221182:A	ggcgggagaa	G→A	ataagtcgca	9.78	0.87	11.21	1.06	2.10	0.05	D			
		rs2077221385:C	gggagaagat	A→C	agtcgcaagg	9.78	0.87	22.68	2.21	12.73	10^{-6}	A			
		rs2077221456:A	agaagataag	T→A	cgcaaggagg	9.78	0.87	11.55	1.01	2.67	10^{-2}	C			
		rs2077221632:T	agataagtcg	C→T	aaggaggggg	9.78	0.87	11.42	0.96	2.54	0.05	D			
		rs2077277363:C	cactgcactg	T→C	gtaccgagga	10.70	1.18	29.27	2.22	15.05	10^{-6}	A			
		rs371621260:G	tgcactgtgt	A→G	ccgaggagac	10.70	1.18	29.27	2.22	15.05	10^{-6}	A			
		rs558448106:C	attggctacc	T→C	ctaggtgaaa	17.23	1.33	19.16	1.45	1.97	0.05	D			
		rs573657900:T	gcgggagaag	A→T	taagtcgcaa	9.78	0.87	12.00	1.10	3.21	10^{-2}	C			
		rs752330449:G	tgttcctcca	A→G	caacaacatc	24.91	1.91	28.84	1.85	2.92	10^{-2}	C			
		rs764308935:G	tccaacaaca	A→G	catcgcccgc	24.91	1.91	31.49	2.25	4.46	10^{-3}	B			
		rs765272012:G	tccactgcac	T→G	gtgtaccgag	10.70	1.18	27.32	2.90	12.26	10^{-6}	A			
		rs776099709:G	cgcatggggc	A→G	tcgtactcta	17.21	1.63	23.58	2.68	4.25	10^{-3}	B			
		rs1161275896:T	gcgggaccta	C→T	acctcaggaa	9.78	0.87	6.89	0.75	4.99	10^{-6}	A	↑	within POAG cellular models using conjunctival fibroblasts under short-term cold atmospheric pressure plasma as a promising effective rapid pre-operative disinfectant for eye surfaces without damages [318]: within the day after such disinfection, OGG1 excess induced excision repair of pre-mutational DNA damages following oxidative stress	▲
		rs1231108714:T	catggggcat	C→T	gtactctagc	17.21	1.63	8.99	0.88	9.55	10^{-6}	A			
		rs1313488677:A	atggggcatc	G→A	tactctagcc	17.21	1.63	9.37	0.88	9.12	10^{-6}	A			
		rs1313488677:T	atggggcatc	G→T	tactctagcc	17.21	1.63	10.05	0.96	8.01	10^{-6}	A			
		rs1313556641:T	gttcctccaa	C→T	aacaacatcg	24.91	1.91	9.50	0.84	16.42	10^{-6}	A			
		rs143170381:A	acaacaacat	C→A	gcccgcatca	24.91	1.91	13.69	1.20	10.28	10^{-6}	A			
		rs201580660:T	cactgtgtac	C→T	gaggagacaa	10.70	1.18	7.92	0.88	3.84	10^{-3}	B			
		rs2077214880:A	ccaccctgat	T→A	tctcattggc	17.23	1.33	12.19	1.13	5.75	10^{-6}	A			
		rs2077214956:A	cctgatttct	C→A	attggcgcct	17.23	1.33	10.85	1.03	7.57	10^{-6}	A			
		rs2077215832:T	gattggctac	C→T	tctaggtgaa	17.23	1.33	7.77	0.75	12.94	10^{-6}	A			
		rs2077215971:A	gctacctcta	G→A	gtgaaatgag	17.23	1.33	11.64	0.87	7.31	10^{-6}	A			
		rs2077238463:C	ggcgcatggg	G→C	catcgtactc	17.21	1.63	14.47	1.31	2.65	10^{-2}	C			
		rs2077277753:T	actgtgtacc	G→T	aggagacaag	10.70	1.18	8.28	0.91	3.29	10^{-3}	B			
		rs2077314196:T	tctgttcctc	C→T	aacaacaaca	24.91	1.91	15.51	1.16	8.84	10^{-6}	A			
		rs2077315980:T	acatcgcccg	C→T	atcactggca	24.91	1.91	14.65	1.61	7.90	10^{-6}	A			
		rs2077317027:A	tcactggcat	G→A	gtggagcggc	24.91	1.91	19.30	2.02	3.94	10^{-3}	B			
		rs2077318942:A	tgccaggctt	T→A	tggacctcgg	24.91	1.91	20.40	1.81	3.41	10^{-3}	B			
		rs757236953:A	ccactgcact	G→A	tgtaccgagg	10.70	1.18	3.53	0.41	13.93	10^{-6}	A			
		rs761699627:T	ctccactgca	C→T	tgtgtaccga	10.70	1.18	8.77	0.91	2.63	10^{-2}	C			
		rs763044440:A	ggcggtgcct	G→A	ctgtggaaat	17.21	1.63	12.73	1.25	4.42	10^{-3}	B			

Table S3. Cont.

Human Gene		Candidate SNP marker				K_D, nM, *in silico*				Significance				Effect of changes in human gene expression on the development of primary open-angle glaucoma (POAG, ☼: "▼" aggravation, "▲" alleviation) [Reference]	☼ ▲ ▼
#	*NCBI Gene Symbol* (NCBI Gene ID)	dbSNP ID:min [437]	5′ flank, 10 bp	WT →min	3 flank, 10 bp	WT MEAN	± SEM	min MEAN	± SEM	Z	p	ϱ	Δ		
102	*OGG1* (4968)	rs777493798:T	tcgcccgcat	C→T	actggcatgg	24.91	1.91	16.34	1.61	6.75	10^{-6}	A	↑	within POAG cellular models using conjunctival fibroblasts under short-term cold atmospheric pressure plasma as a promising effective rapid pre-operative disinfectant for eye surfaces without damages [318]: within the day after such disinfection, OGG1 excess induced excision repair of pre-mutational DNA damages following oxidative stress	▲
		rs909688286:A	gaagataagt	C→A	gcaaggaggg	9.78	0.87	4.93	0.50	10.16	10^{-6}	A			
		rs915196024:A	taggtgaaat	G→A	agcggtggtt	17.23	1.33	15.27	1.16	2.24	0.05	D			
		rs922604565:G	ttggctacct	C→G	taggtgaaat	17.23	1.33	13.01	1.39	4.26	10^{-3}	B			
103	*OPA1* (4976)	rs1408107340:T	ccatatatat	A→T	ggttaatttt	1.30	0.13	2.05	0.20	6.46	10^{-6}	A	↓	according to a cohort-based biomedical study using qPCR [319]: OPA1-deficiency is a biomedical molecular marker for susceptibility to POAG	▼
		rs1445003186:C	gctgcccgcc	T→C	agaaagggtg	18.46	1.73	51.27	4.06	16.63	10^{-6}	A			
		rs1716741428:A	actctccata	T→A	atataggtta	1.30	0.13	2.05	0.20	6.46	10^{-6}	A			
		rs1716741428:C	actctccata	T→C	atataggtta	1.30	0.13	2.05	0.20	6.46	10^{-6}	A			
		rs1716743068:T	tatataggtt	A→T	atttatcat	1.30	0.13	1.60	0.16	2.96	10^{-2}	C			
		rs1724911639:G	tgacggactg	A→G	gtacgggtgc	22.43	2.51	46.84	4.91	9.60	10^{-6}	A			
		rs1724912200:T	cggactgagt	A→T	cgggtgcctg	22.43	2.51	51.92	3.96	12.39	10^{-6}	A			
		rs1724924981:C	cggccgcggc	T→C	ctgtgccctt	61.92	5.19	75.56	5.20	3.67	10^{-3}	B			
		rs1724925548:C	gccgcggctc	T→C	gtgcccttgc	61.92	5.19	75.56	5.20	3.67	10^{-3}	B			
		rs908070414:C	gcggctctgt	G→C	cccttgctgc	61.92	5.19	75.20	6.29	3.28	10^{-2}	C			
		rs968211001:C	cattttaaag	T→C	gtgttcacaa	6.13	0.46	7.62	0.45	4.53	10^{-3}	B			
		rs972416371:G	ctgcccgcct	A→G	gaaagggtga	18.46	1.73	51.27	4.06	16.63	10^{-6}	A			
		rs972416371:T	ctgcccgcct	A→T	gaaagggtga	18.46	1.73	36.07	2.52	11.44	10^{-6}	A			
		rs1035848869:T	cagtccgttc	C→T	cgacgcactg	81.92	5.62	68.93	4.44	3.67	10^{-3}	B	↑	within human POAG models using rats [320]: Opa1 upregulation may protect retinal ganglion cells by ways of enhancing mitochondria fusion, which may provide a useful strategy to battle against POAG	▲
		rs1171895507:A	aggctcttgc	G→A	gaagtccatg	61.92	5.19	34.05	2.73	10.31	10^{-6}	A			
		rs1198278699:A	cccgtgagag	G→A	cgatggattg	81.92	5.62	58.03	4.05	7.05	10^{-6}	A			
		rs1198278699:T	cccgtgagag	G→T	cgatggattg	81.92	5.62	61.86	4.70	5.48	10^{-6}	A			
		rs1228223981:T	gcgatggatt	G→T	ctccagtccg	81.92	5.62	41.31	2.89	13.97	10^{-6}	A			
		rs1302113197:T	cgatggattg	C→T	tccagtccgt	81.92	5.62	70.07	5.11	3.12	10^{-2}	C			
		rs1331047563:A	agtccgttcc	C→A	gacgcactgt	81.92	5.62	68.96	5.80	3.17	10^{-2}	C			
		rs1331047563:T	agtccgttcc	C→T	gacgcactgt	81.92	5.62	72.47	5.69	2.35	0.05	D			
		rs1353303633:T	tgcggaagtc	C→T	atgcgccatt	61.92	5.19	24.39	2.39	14.43	10^{-6}	A			
		rs1415935265:G	ggccgcggct	C→G	tgtgcccttg	61.92	5.19	46.12	3.96	4.91	10^{-6}	A			
		rs1438053247:T	ggcggggccc	C→T	gtgagaggcg	81.92	5.62	53.18	4.21	8.25	10^{-6}	A			
		rs1475453155:T	tgtgcgcatg	C→T	gctggtcctc	81.92	5.62	51.83	3.99	8.88	10^{-6}	A			
		rs1477694300:A	gtgcctgtca	G→A	gctcttgcgg	61.92	5.19	48.72	3.67	4.25	10^{-3}	B			
		rs1486042414:T	cggctctgtg	C→T	ccttgctgct	61.92	5.19	34.20	2.71	10.29	10^{-6}	A			
		rs1553876585:A	tttgttttgt	G→A	ggaaggttgt	17.26	1.26	5.75	0.60	17.31	10^{-6}	A			
		rs1560296419:A	ttgcggaagt	C→A	catgcgccat	61.92	5.19	14.88	1.97	18.19	10^{-6}	A			
		rs1577095173:T	gcccgcctag	A→T	aagggtgaag	18.46	1.73	10.27	1.11	8.20	10^{-6}	A			
		rs1703730899:A	gcactgtgcg	C→A	atgcgctggt	81.92	5.62	40.06	2.97	14.16	10^{-6}	A			
		rs1724891697:A	gagggcgggg	C→A	cccgtgagag	81.92	5.62	72.20	5.44	2.48	0.05	D			
		rs1724892161:A	ggggccccgt	G→A	agaggcgatg	81.92	5.62	23.80	2.44	20.06	10^{-6}	A			
		rs1724893344:T	agaggcgatg	G→T	attgctccag	81.92	5.62	14.25	1.43	28.77	10^{-6}	A			
		rs1724895720:A	cgttcccgac	G→A	cactgtgcgc	81.92	5.62	34.36	2.89	16.01	10^{-6}	A			
		rs1724895880:A	ccgacgcact	G→A	tgcgcatgcg	81.92	5.62	29.29	2.56	18.51	10^{-6}	A			
		rs1724906489:T	tgctgcccgc	C→T	tagaaagggt	18.46	1.73	15.54	1.56	2.51	0.05	D			
		rs1724907756:T	tagaaagggt	G→T	aagtggttgt	18.46	1.73	13.43	1.15	5.02	10^{-6}	A			
		rs1724908450:A	agggtgaagt	G→A	gttgtttccg	22.43	2.51	14.94	1.48	5.44	10^{-6}	A			
		rs1724913095:A	gagtacgggt	G→A	cctgtcaggc	61.92	5.19	25.17	3.14	11.99	10^{-6}	A			
		rs1724913594:T	agtacgggtg	C→T	ctgtcaggct	61.92	5.19	50.24	4.28	3.50	10^{-3}	B			

Table S3. Cont.

Human Gene #	NCBI Gene Symbol (NCBI Gene ID)	Candidate SNP marker: dbSNP ID:min [437]	5′ flank, 10 bp	WT →min	3 flank, 10 bp	K_D, nM, *in silico*: WT MEAN	± SEM	min MEAN	± SEM	Significance: Z	p	P	Δ	Effect of changes in human gene expression on the development of primary open-angle glaucoma (POAG, ☼: "▼" aggravation, "▲" alleviation) [Reference]	☼ ▲ ▼
103	*OPA1* (4976)	rs1724914451:T	gggtgcctgt	C→T	aggctcttgc	61.92	5.19	26.28	2.31	14.10	10^{-6}	A	↑	within human POAG models using rats [320]: Opa1 upregulation may protect retinal ganglion cells by ways of enhancing mitochondria fusion, which may provide a useful strategy to battle against POAG	▲
		rs1724916503:A	tcaggctctt	G→A	cggaagtcca	61.92	5.19	17.90	1.66	19.84	10^{-6}	A			
		rs1724927984:A	tgcccttgct	G→A	ctgagggcca	61.92	5.19	24.90	2.64	13.48	10^{-6}	A			
		rs1733198507:C	gactagtgtg	T→C	tggaaatgat	17.26	1.26	15.34	1.28	2.13	0.05	D			
		rs532439820:T	gctctgtgcc	C→T	ttgctgctga	61.92	5.19	53.81	4.31	2.42	0.05	D			
		rs543322961:G	tccgttcccg	A→G	cgcactgtgc	81.92	5.62	68.51	5.97	3.22	10^{-2}	C			
		rs551297694:A	agactagtgt	G→A	ttggaaatga	17.26	1.26	7.26	0.80	13.16	10^{-6}	A			
		rs755377312:A	aggttgttgt	G→A	gttggagatc	17.26	1.26	8.23	0.81	12.09	10^{-6}	A			
		rs755794042:T	gtgtgttgga	A→T	atgattgccc	17.26	1.26	12.59	1.11	5.50	10^{-6}	A			
		rs895958718:T	ctcggccgcg	G→T	ctctgtgccc	61.92	5.19	42.16	3.48	6.53	10^{-6}	A			
		rs902875056:A	tgcctgtcag	G→A	ctcttgcgga	61.92	5.19	45.83	3.87	5.06	10^{-6}	A			
		rs905603873:T	tgtgcccttg	C→T	tgctgagggc	61.92	5.19	40.48	2.58	8.07	10^{-6}	A			
104	*OPTC* (26254)	rs1453279693:G	tgcgagccat	T→G	cccacaacac	23.05	1.89	25.88	2.08	2.02	0.05	D	↓	within cohort-based biomedical POAG study using RT-qPCR [321]: OPTC deficiency occurs with reduced visual acuity, visual field defects near fixation point in the infero-temporal quadrant and tubular vision as POAG symptoms	▼
		rs1661240776:T	accactgtct	A→T	acccaagccc	15.50	1.75	19.34	1.57	3.18	10^{-2}	C			
		rs765345117:G	tctgcgagcc	A→G	ttcccacaac	23.05	1.89	25.80	2.05	1.98	0.05	D			
		rs868268305:G	cattcccaca	A→G	cactctggag	23.05	1.89	56.90	3.71	17.27	10^{-6}	A			
		rs891547924:C	ggcaccactg	T→C	ctaacccaag	15.50	1.75	47.36	3.99	15.85	10^{-6}	A			
		rs1661240696:A	gggcaccact	G→A	tctaacccaa	15.50	1.75	4.61	0.53	15.10	10^{-6}	A	↑	within human POAG models using mice subjected with recombinant opticin injection into eyes [322]: inhibited pathologic preretinal neovascularization within the normally avascular mature vitreous that can alleviate POAG	▲
		rs1661240848:G	cactgtctaa	C→G	ccaagcccca	15.50	1.75	13.31	1.28	2.05	0.05	D			
		rs1661270238:T	attcccacaa	C→T	actctggaga	23.05	1.89	13.47	1.01	9.68	10^{-6}	A			
		rs1661270467:T	gcctgtccct	C→T	agatcgctgc	23.05	1.89	18.88	1.58	3.40	10^{-3}	B			
		rs1661270503:T	ctgtccctca	G→T	atcgctgccc	23.05	1.89	11.84	1.02	11.22	10^{-6}	A			
		rs965158486:A	gtgcccactt	G→A	ccaggaccag	23.05	1.89	20.17	2.15	1.99	0.05	D			
105	*OPTN* (10133)	rs1053520071:G	agacatattg	T→G	gttaaatccc	7.43	0.60	9.54	0.84	4.18	10^{-6}	A	↓	according to an exhaustive review [323]: OPTN-dysfunction can lead mitochondrial degradation, accumulation of damaged mitochondria and defective mitophagy in the axons, which taken altogether can aggravate POAG	▼
		rs1833205412:T	atattgtgtt	A→T	aatcccttgc	7.43	0.60	8.82	0.69	3.05	10^{-6}	A			
		rs755458443:G	tgttcattca	C→G	tttactcctt	10.88	0.83	13.64	0.98	4.33	10^{-6}	A			
		rs879161543:C	cttgtagaca	T→C	attgtgttaa	7.43	0.60	11.22	1.09	6.54	10^{-6}	A			
		rs1309777507:C	tagacatatt	G→C	tgttaaatcc	7.43	0.60	9.00	0.70	3.41	10^{-6}	A			
		rs1309777507:A	tagacatatt	G→A	tgttaaatcc	7.43	0.60	5.38	0.32	6.49	10^{-6}	A	↑	within an exhaustive review [323]: OPTN excess in retinal ganglion cells may enhance mitochondrial biogenesis, fission, and volume density that may alleviate POAG	▲
		rs1307411426:T	ccaggccgcg	C→T	atcagcccta	91.61	7.57	31.40	3.38	15.77	10^{-6}	A			
		rs1360919961:T	ctctccctcc	C→T	tctctccctc	76.94	5.64	61.30	4.55	4.36	10^{-6}	A			
		rs1364103323:T	ttccctcctc	C→T	acaccagcag	23.02	1.47	18.49	2.03	3.45	10^{-6}	A			
		rs1390458091:T	ctattcttca	G→T	caacaagaat	23.02	1.47	18.70	2.08	3.24	10^{-6}	A			
		rs1401824386:A	gacatattgt	G→A	ttaaatccct	7.43	0.60	2.78	0.29	14.81	10^{-6}	A			
		rs1413337853:G	ctccctctct	C→G	cgtccctccc	76.94	5.64	66.24	4.77	2.92	10^{-6}	A			
		rs1413337853:T	ctccctctct	C→T	cgtccctccc	76.94	5.64	44.51	2.96	11.06	10^{-6}	A			
		rs1426377456:T	tccctctctc	C→T	gtccctccct	76.94	5.64	48.93	3.52	8.82	10^{-6}	A			
		rs1481317881:T	attcacttta	C→T	tccttgtcat	10.88	0.83	6.93	0.58	8.00	10^{-6}	A			
		rs1487522292:A	gccttagttt	G→A	atctgttcat	10.88	0.83	8.07	0.56	5.80	10^{-6}	A			
		rs1588425821:G	ccctccctct	C→G	tccctctctc	76.94	5.64	59.59	4.76	4.72	10^{-6}	A			
		rs1588425838:T	ccgtccctcc	C→T	tctctctctc	76.94	5.64	60.16	3.99	4.98	10^{-6}	A			
		rs1743842825:T	ccgcgcatca	G→T	ccctaggcac	91.61	7.57	41.18	3.24	14.03	10^{-6}	A			
		rs1832686819:A	ctctctccct	C→A	cctctctccc	76.94	5.64	46.84	3.91	8.94	10^{-6}	A			
		rs1832688083:G	ccctccctct	C→G	tctctctctc	76.94	5.64	49.57	3.85	8.24	10^{-6}	A			
		rs1832688174:T	ccctctctct	C→T	tctctccccc	76.94	5.64	38.23	2.60	14.00	10^{-6}	A			

Table S3. Cont.

Human Gene		Candidate SNP marker				K$_D$, nM, *in silico*				Significance				Effect of changes in human gene expression on the development of primary open-angle glaucoma (POAG, ☼: "▼" aggravation, "▲" alleviation) [Reference]	☼ ▲ ▼
#	*NCBI Gene Symbol* (NCBI Gene ID)	dbSNP ID:min [437]	5′ flank, 10 bp	WT →min	3 flank, 10 bp	WT MEAN±	WT SEM	min MEAN±	min SEM	Z	p	ϱ	Δ		
105	*OPTN* (10133)	rs1832688359:A	ctctctctct	C→A	cccctccctc	76.94	5.64	23.82	2.19	19.97	10^{-6}	A	↑	within an exhaustive review [323]: OPTN excess in retinal ganglion cells may enhance mitochondrial biogenesis, fission, and volume density that may alleviate POAG	▲
		rs1832690408:A	gctgtctaat	C→A	cgggggcagc	23.02	1.47	9.72	0.79	16.72	10^{-6}	A			
		rs1832691073:T	tcctccacac	C→T	agcagaggct	23.02	1.47	20.16	1.81	2.40	0.05	D			
		rs1832691524:A	aacaagaata	G→A	ccgagcctat	23.02	1.47	20.21	1.39	2.78	10^{-2}	C			
		rs1832704288:T	cgcgggcagg	G→T	agcggctggc	91.61	7.57	52.84	5.05	8.71	10^{-6}	A			
		rs1832706304:T	caggccgcgc	A→T	tcagccctag	91.61	7.57	76.80	5.82	3.15	10^{-2}	C			
		rs1833204517:C	agaatttgtc	T→C	tgtagacata	7.43	0.60	6.63	0.50	2.07	0.05	D			
		rs369971287:G	cgcatcagcc	C→G	taggcacccc	91.61	7.57	58.22	6.32	6.65	10^{-6}	A			
		rs369971287:T	cgcatcagcc	C→T	taggcacccc	91.10	8.95	57.52	5.30	6.83	10^{-6}	A			
		rs754983786:C	tgtagacata	T→C	tgtgttaaat	7.43	0.60	6.18	0.58	2.97	10^{-2}	C			
		rs766197705:T	gtcttgtaga	C→T	atattgtgtt	7.43	0.60	2.90	0.37	12.44	10^{-6}	A			
		rs909544362:T	gctggctgtc	A→T	gctgagccgc	91.61	7.57	62.43	5.15	6.57	10^{-6}	A			
		rs921462437:T	cgcgcatcag	C→T	cctaggcacc	91.61	7.57	50.75	4.77	9.44	10^{-6}	A			
		rs934177393:T	gcgcatcagc	C→T	ctaggcaccc	91.61	7.57	63.65	5.13	6.31	10^{-6}	A			
		rs950760673:T	ctaggcaccc	C→T	agtcccggct	91.10	8.95	59.92	5.35	6.31	10^{-6}	A			
106	*PADI2* (11240)	rs1242550498:G	gccgctggat	A→G	cccgcccacc	19.50	1.74	73.44	5.66	22.45	10^{-6}	A	↓	according to comprehensive review [324]: in the contrary to age-related neurodegeneration, both retinal deimination and PADI2 expression decrease with age during healthy aging	▲
		rs909512842:G	caccggccgc	T→G	gccgccccgc	19.50	1.74	23.19	2.07	2.74	10^{-2}	C			
		rs979744989:C	tggataaggc	T→C	ccaccggccg	19.50	1.74	22.60	2.02	2.33	0.05	D			
		rs1400283492:A	accggccgct	G→A	ccgccccgcc	19.50	1.74	5.71	0.56	18.44	10^{-6}	A	↑	within cohort-based post-mortem studies [325]: PADI2 excess in the optic nerve can be a biomedical molecular marker for susceptibility to POAG	▼
		rs771116097:A	gctggataag	G→A	gcccaccggc	19.50	1.74	10.09	0.89	10.47	10^{-6}	A			
		rs776875955:A	cccaccggcc	G→A	gggccgcccc	19.50	1.74	16.94	1.48	2.26	0.05	D			
		rs933837152:A	gcagcccact	G→A	cggacaggcc	19.50	1.74	15.15	1.41	3.91	10^{-3}	B			
107	*PARP1* (142)	rs1406776870:T	gccccgtgga	C→T	ccgccggccc	110.13	8.96	123.38	9.99	1.98	0.05	D	↓	within human POAG models using Parp1-knockout mice [137]: increases resistance to retinal neurodegeneration without affecting retinal function	▲
		rs1665190766:T	cgcgggttcc	G→T	cgccccgtgg	110.13	8.96	154.18	11.67	6.05	10^{-6}	A			
		rs1447006640:T	gacgcgggtt	C→T	cccgccccgt	110.13	8.96	37.93	2.87	19.17	10^{-6}	A	↑	within cohort-based elderly women biomedical study [326]: smoking elevates PARP1 level that can speed up both apoptosis and POAG	▼
		rs1665190174:A	gttccgtggg	C→A	cgtggacgcg	110.13	8.96	98.09	7.98	2.01	0.05	D			
108	*PLXDC2* (84898)	rs1185738497:A	tttttttaaa	C→A	acattttttt	4.66	0.38	5.81	0.50	3.71	10^{-3}	B	↓	within a cohort-based biomedical studies [327]: PLXDC2 deficiency is a biomedical molecular marker for lesser risks of ischemic stroke, which is comorbid to POAG [328]	▲
		rs1376111939:A	tttttttttt	T→A	aaacacattt	4.66	0.38	5.62	0.43	3.37	10^{-3}	B			
		rs1376111939:C	tttttttttt	T→C	aaacacattt	4.66	0.38	12.70	0.83	19.20	10^{-6}	A			
		rs1486671376:C	tttttttttt	A→C	aacacatttt	4.66	0.38	15.50	1.01	23.04	10^{-6}	A			
		rs1486671376:T	tttttttttt	A→T	aacacatttt	4.66	0.38	8.94	0.60	12.31	10^{-6}	A			
		rs1836336211:G	ttccctcctc	A→G	aagtgtggag	22.01	1.72	39.95	2.95	11.08	10^{-6}	A			
		rs1836336255:A	cctcctcaaa	G→A	tgtggagaag	22.01	1.72	33.36	2.28	8.01	10^{-6}	A			
		rs1836336303:A	cctcaaagtg	T→A	ggagaaggag	22.01	1.72	31.65	2.81	6.14	10^{-6}	A			
		rs1836340590:C	ttttttttaa	A→C	cacatttttt	4.66	0.38	8.25	0.56	10.79	10^{-6}	A			
		rs1836340590:T	ttttttttaa	A→T	cacatttttt	4.66	0.38	8.06	0.59	10.01	10^{-6}	A			
		rs944753048:C	ctgccttccc	T→C	cctcaaagtg	22.01	1.72	25.27	1.91	2.54	0.05	D			
		rs1040768437:T	tgccttccct	C→T	ctcaaagtgt	22.01	1.72	15.49	1.15	6.53	10^{-6}	A	↑	within a cohort-based biomedical studies [327]: PLXDC2 excess is a biomedical molecular marker for susceptibility to ischemic stroke, which is comorbid to POAG [328]	▼
		rs1173342083:A	tcctcaaagt	G→A	tggagaagga	22.01	1.72	13.74	1.31	7.64	10^{-6}	A			
		rs1293332198:A	ggcggcagat	C→A	tggcaaattg	22.01	1.72	10.69	1.08	11.28	10^{-6}	A			
		rs1836336172:T	cttccctcct	C→T	aaagtgtgga	22.01	1.72	7.58	0.73	17.23	10^{-6}	A			
		rs1836336331:T	tcaaagtgtg	G→T	agaaggagcg	22.01	1.72	9.92	0.81	14.06	10^{-6}	A			

Table S3. Cont.

#	Human Gene: *NCBI Gene Symbol* (NCBI Gene ID)	Candidate SNP marker: dbSNP ID:min [437]	5′ flank, 10 bp	WT→min	3 flank, 10 bp	K_D, nM, *in silico*: WT MEAN	± SEM	min MEAN	± SEM	Significance: Z	p	ϱ	Δ	Effect of changes in human gene expression on the development of primary open-angle glaucoma (POAG, ☼: "▼" aggravation, "▲" alleviation) [Reference]	☼ ▲ ▼
109	*PMM2* (5373)	rs1015882031:C	tcgaatgtac	A→C	aggcgggcgt	11.98	1.23	16.16	1.60	4.20	10^{-3}	B	↓	within a cohort-based biomedical study [329]: PMM2 deficiency is a biomedical molecular marker for congenital glycosylation disorders aggravating POAG [330]	▼
		rs1015882031:G	tcgaatgtac	A→G	aggcgggcgt	11.98	1.23	15.53	1.59	3.59	10^{-3}	B			
		rs1397914439:A	tgctcgaatg	T→A	acaaggcggg	11.98	1.23	16.16	1.60	4.20	10^{-3}	B			
		rs1567324564:A	tcaaaacaag	G→A	ccattaaaag	6.38	0.66	7.38	0.47	2.41	0.05	D			
		rs1567327778:C	caacgtgtct	T→C	gtaaggtgcg	15.27	1.13	32.81	2.38	14.78	10^{-6}	A			
		rs1596481707:G	ctcccaggcg	T→G	tatgggaacg	30.31	2.44	34.68	3.16	2.22	0.05	D			
		rs2060587973:C	acgtgtcttg	T→C	aaggtgcggc	15.27	1.13	41.43	3.79	16.97	10^{-6}	A			
		rs2060587981:G	cgtgtcttgt	A→G	aggtgcggct	15.27	1.13	35.18	2.27	17.01	10^{-6}	A			
		rs377441458:G	gctcgaatgt	A→G	caaggcgggc	11.98	1.23	16.16	1.60	4.20	10^{-3}	B			
		rs749166324:C	ccaacgtgtc	T→C	tgtaaggtgc	15.27	1.13	31.75	2.52	13.52	10^{-6}	A			
		rs749166324:G	ccaacgtgtc	T→G	tgtaaggtgc	15.27	1.13	23.54	1.77	8.21	10^{-6}	A			
		rs751782324:A	ctcgtgccaa	C→A	gtgtcttgta	15.27	1.13	19.37	1.23	4.88	10^{-3}	B			
		rs888275252:G	cattaaaagt	T→G	ggactctgcc	6.38	0.66	7.29	0.47	2.19	0.05	D			
		rs1002344814:T	tcctcttccc	G→T	acgtgccctg	30.31	2.44	22.50	2.05	4.90	10^{-6}	A	↑	exogenous recombinant insulin injections can both upregulate PMM2 within human disease cellular models using COS-7 cells [331] and relieve POAG according to an exhaustive review [332]	▲
		rs1047959332:A	ctcgaatgta	C→A	aaggcgggcg	11.98	1.23	5.94	0.57	9.98	10^{-6}	A			
		rs1191382478:T	atgtacaagg	C→T	gggcgtgatc	11.98	1.23	10.18	1.05	2.23	0.05	D			
		rs1220452675:T	gtgccaacgt	G→T	tcttgtaagg	15.27	1.13	13.59	1.10	2.13	0.05	D			
		rs1220464161:A	gcgcatgctc	G→A	aatgtacaag	11.98	1.23	9.16	0.94	3.70	10^{-3}	B			
		rs1238829249:T	ccctgcgact	C→T	agcggccgaa	30.31	2.44	24.99	2.42	3.06	10^{-2}	C			
		rs1315989776:A	gccaacgtgt	C→A	ttgtaaggtg	15.27	1.13	8.16	0.47	13.32	10^{-6}	A			
		rs1349387453:T	cctcctcttc	C→T	cgacgtgccc	30.31	2.44	25.31	1.76	3.38	10^{-3}	B			
		rs1596481707:A	ctcccaggcg	T→A	tatgggaacg	30.31	2.44	19.30	1.69	7.59	10^{-6}	A			
		rs2060586031:A	atgctcgaat	G→A	tacaaggcgg	11.98	1.23	3.11	0.36	17.38	10^{-6}	A			
		rs2060586031:T	atgctcgaat	G→T	tacaaggcgg	11.98	1.23	8.76	1.02	4.03	10^{-3}	B			
		rs7359468:A	gaatgtacaa	G→A	gcgggcgtga	11.98	1.23	6.90	0.71	7.61	10^{-6}	A			
		rs745432642:A	ggaagtaaat	C→A	ccaccggccc	11.98	1.23	6.06	0.70	8.78	10^{-6}	A			
		rs763287830:A	ggcgttatgg	G→A	aacggagtcc	30.31	2.44	23.11	1.78	4.87	10^{-3}	B			
		rs949011351:C	aggcgttatg	G→C	gaacggagtc	30.31	2.44	23.24	1.93	4.60	10^{-3}	B			
110	*PON1* (5444)	rs1408703340:G	cggcccagct	A→G	gtgcacctgg	61.82	6.50	203.13	15.44	18.34	10^{-6}	A	↓	according to an exhaustive review [333]: fluoride contributes to many eye diseases including POAG through reducing PON1 and antioxidant levels	▼
		rs976861427:G	tcggcccagc	T→G	tgtgcacctg	61.82	6.50	230.98	18.49	19.96	10^{-6}	A			
		rs1237732763:T	gctgccccag	C→T	ggccaatcgg	61.82	6.50	48.23	4.38	3.58	10^{-3}	B	↑	within a cohort-based biomedical study [334]: PON1 excess in blood is a biomedical molecular marker for susceptibility to POAG	
		rs1807976172:T	cagctagctg	C→T	cctggtcggc	61.82	6.50	41.91	3.72	5.66	10^{-6}	A			
		rs553285883:T	cctggtcggc	C→T	ccttctgtgc	61.82	6.50	40.99	3.86	5.82	10^{-6}	A			
111	*PPARG* (5468)	rs1158612015:T	agaaatctca	G→T	gtcccttggt	8.69	0.57	10.49	0.60	4.32	10^{-3}	B	↓	within a cohort-based biomedical study [335]: difference between intraocular and intracranial pressures is among main risk factors of both primary open-angle glaucoma and Graves' orbitopathy, while PPARG-deficit is a biomedical molecular marker for Graves' orbitopathy [336]	▼
		rs1192457451:C	ggctggaaag	G→C	cgaagtcgct	79.65	6.19	93.47	7.31	2.90	10^{-2}	C			
		rs1199047527:C	ctgcggcttt	T→C	atataaggtc	1.21	0.13	3.84	0.31	17.51	10^{-6}	A			
		rs1218356709:C	gtctcttttt	T→C	attgttaaga	3.28	0.27	3.96	0.46	2.66	10^{-2}	C			
		rs1220607751:C	ttcctatttt	A→C	tgtattgttt	2.86	0.26	3.74	0.39	3.91	10^{-3}	B			
		rs1363652767:G	ataacttttt	A→G	gactaacatt	5.42	0.52	10.77	1.03	10.17	10^{-6}	A			
		rs1437299483:C	ttaacagaaa	T→C	ctcaggtccc	8.69	0.57	11.87	0.81	6.60	10^{-6}	A			
		rs1574928464:G	gcagcagagg	T→G	taacagaaat	8.69	0.57	11.18	0.96	4.67	10^{-3}	B			
		rs183181489:C	tccctcccag	T→C	cgcgcgccgc	314.57	24.61	475.82	47.90	6.49	10^{-6}	A			
		rs2046522165:G	aatgattaaa	A→G	gtcagtgact	6.17	0.45	7.77	0.63	4.21	10^{-3}	B			
		rs2046538832:C	gctccctccc	A→C	gtcgcgcgcc	314.57	24.61	475.82	47.90	6.49	10^{-6}	A			
		rs2046538914:G	ccctcccagt	C→G	gcgcgccgcc	314.57	24.61	409.46	28.46	5.04	10^{-6}	A			

Table S3. Cont.

Human Gene		Candidate SNP marker				K_D, nM, *in silico*				Significance				Effect of changes in human gene expression on the development of primary open-angle glaucoma (POAG, ☼: "▼" aggravation, "▲" alleviation) [Reference]	☼
#	*NCBI Gene Symbol* (NCBI Gene ID)	dbSNP ID:min [437]	5′ flank, 10 bp	WT →min	3 flank, 10 bp	WT MEAN	± SEM	min MEAN	± SEM	Z	p	ϱ	Δ		▲▼
111	*PPARG* (5468)	rs2046538966:C	cctcccagtc	G→C	cgcgccgccg	314.57	24.61	406.47	27.67	4.94	10⁻⁶	A	↓	within a cohort-based biomedical study [335]: difference between intraocular and intracranial pressures is among main risk factors of both primary open-angle glaucoma and Graves' orbitopathy, while PPARG-deficit is a biomedical molecular marker for Graves' orbitopathy [336]	▼
		rs2046548612:G	ggcccagcgc	A→G	ctcggagccc	133.57	10.82	329.78	25.65	16.09	10⁻⁶	A			
		rs2046578043:A	gaggttaaca	G→A	aaatctcagg	8.69	0.57	10.58	0.62	4.47	10⁻³	B			
		rs2046581316:G	tgcggctttt	A→G	tataaggtca	1.21	0.13	3.25	0.33	13.51	10⁻⁶	A			
		rs2046912088:A	attcctattt	T→A	atgtattgtt	2.86	0.26	3.52	0.30	3.35	10⁻³	B			
		rs2047045287:G	tttttagact	A→G	acattactca	5.42	0.52	8.95	0.77	7.79	10⁻⁶	A			
		rs2047270857:A	aaaatcttgt	C→A	tctttttttat	3.28	0.27	3.96	0.46	2.66	10⁻²	C			
		rs572181473:C	gttaacagaa	A→C	tctcaggtcc	8.69	0.57	13.18	0.87	8.96	10⁻⁶	A			
		rs572181473:G	gttaacagaa	A→G	tctcaggtcc	8.69	0.57	12.00	0.69	7.40	10⁻⁶	A			
		rs919315050:G	aggttaacag	A→G	aatctcaggt	8.69	0.57	12.55	0.82	7.96	10⁻⁶	A			
		rs934011901:A	ggcagcagag	G→A	ttaacagaaa	8.69	0.57	10.31	1.14	2.66	10⁻²	C			
		rs934011901:C	ggcagcagag	G→C	ttaacagaaa	8.69	0.57	10.34	0.69	3.70	10⁻³	B			
		rs1052854589:A	accccggggg	G→A	ctggaaaggc	79.65	6.19	55.40	4.39	6.54	10⁻⁶	A	↑	within human disease models using mice [337]: magnolol as principal ingredient of *Magnoliae officinalis* cortex, has anti-inflammatory and neuroprotective effects and relieves retinal damages and inflammation due to, mainly, Pparg-upregulation, which altogether can alleviate POAG	▲
		rs1184525317:A	cccaccccca	C→A	ccccaccccc	475.82	47.90	240.57	19.26	10.61	10⁻⁶	A			
		rs1184525317:T	cccaccccca	C→T	ccccaccccc	475.82	47.90	205.87	17.75	12.64	10⁻⁶	A			
		rs1192457451:A	ggctggaaag	G→A	cgaagtcgct	79.65	6.19	64.87	4.56	3.92	10⁻³	B			
		rs1193705856:A	ctgtgtacat	G→A	tgtacacatt	3.28	0.27	2.82	0.21	2.77	10⁻²	C			
		rs1213136843:T	cacccccacc	C→T	ccagccggcg	314.57	24.61	145.18	12.27	13.43	10⁻⁶	A			
		rs1222421344:T	tcgcgcgccg	C→T	cgcccccgcc	314.57	24.61	249.50	15.94	4.59	10⁻³	B			
		rs1244804516:T	tgggtgcctc	C→T	gtgcctccgc	79.65	6.19	58.37	4.86	5.46	10⁻⁶	A			
		rs1252548019:T	cccccacccc	C→T	acccccagcc	314.57	24.61	94.17	9.39	19.04	10⁻⁶	A			
		rs1272976323:A	cccaccccca	C→A	ccccagccgg	475.82	47.90	263.55	20.05	9.36	10⁻⁶	A			
		rs1274706735:A	cacccccagc	C→A	ggcgcccgcg	314.57	24.61	234.87	16.98	5.49	10⁻⁶	A			
		rs1274706735:T	cacccccagc	C→T	ggcgcccgcg	314.57	24.61	234.68	17.00	5.50	10⁻⁶	A			
		rs1306266855:A	cccaccccca	C→A	ccccaccccc	475.82	47.90	240.57	19.31	10.59	10⁻⁶	A			
		rs1306266855:T	cccaccccca	C→T	ccccaccccc	475.82	47.90	205.87	17.72	12.65	10⁻⁶	A			
		rs1311328381:A	gcccccaccc	C→A	cacccccacc	314.57	24.61	105.23	9.26	18.60	10⁻⁶	A			
		rs1311328381:T	gcccccaccc	C→T	cacccccacc	314.57	24.61	143.16	10.88	14.44	10⁻⁶	A			
		rs1338989043:T	ccaccccccac	C→T	cccaccccca	314.57	24.61	148.16	12.57	13.05	10⁻⁶	A			
		rs1346034321:A	cggggcccag	C→A	gcactcggag	133.57	10.82	95.84	7.88	5.75	10⁻⁶	A			
		rs1362070177:G	acccccaccc	C→G	cagccggcgc	475.82	47.90	362.20	30.11	4.18	10⁻³	B			
		rs1362070177:T	acccccaccc	C→T	cagccggcgc	475.82	47.90	201.52	15.01	13.72	10⁻⁶	A			
		rs1386887028:A	agcgcactcg	G→A	agcccgagcc	133.57	10.82	64.71	4.43	13.67	10⁻⁶	A			
		rs1386887028:T	agcgcactcg	G→T	agcccgagcc	133.57	10.82	18.05	1.79	31.29	10⁻⁶	A			
		rs1390824474:T	gcccgagccg	C→T	agccgccgcc	133.57	10.82	111.88	12.77	2.53	0.05	D			
		rs1418068807:T	ttttgtgata	C→T	ataatgatat	2.86	0.26	1.37	0.17	9.52	10⁻⁶	A			
		rs1429124873:T	cccgcccccg	C→T	ccccaccccc	475.82	47.90	387.86	35.95	2.99	10⁻²	C			
		rs1452201064:G	cccccacccc	C→G	agccggcgcc	475.82	47.90	407.45	35.45	2.33	0.05	D			
		rs1469312525:A	cgcccccacc	C→A	ccaccccccac	314.57	24.61	152.42	12.64	12.71	10⁻⁶	A			
		rs1469312525:T	cgcccccacc	C→T	ccaccccccac	314.57	24.61	142.17	12.21	13.67	10⁻⁶	A			
		rs1559479356:T	actgctaaga	A→T	atttaagaaa	6.17	0.45	3.66	0.36	8.52	10⁻⁶	A			
		rs1574926139:G	ccgcccccac	C→G	cccaccccca	475.82	47.90	306.76	26.30	6.64	10⁻⁶	A			
		rs1574928152:T	ctgggtgcct	C→T	cgtgcctccg	79.65	6.19	53.11	4.11	7.39	10⁻⁶	A			
		rs1574928184:A	ctccgcggat	G→A	gagcgcgcag	79.65	6.19	31.34	3.04	15.02	10⁻⁶	A			
		rs183181489:A	tccctcccag	T→A	cgcgcgccgc	314.57	24.61	215.53	16.67	6.87	10⁻⁶	A			

Table S3. Cont.

Human Gene		Candidate SNP marker				K_D, nM, *in silico*				Significance				Effect of changes in human gene expression on the development of primary open-angle glaucoma (POAG, ☼: "▼" aggravation, "▲" alleviation) [Reference]	☼
						WT		min							▲
#	*NCBI Gene Symbol* (NCBI Gene ID)	dbSNP ID:min [437]	5′ flank, 10 bp	WT →min	3 flank, 10 bp	MEAN	± SEM	MEAN	± SEM	Z	p	ϱ	Δ		▼
		rs2046523159:T	aacttacaaa	C→T	gcaaggaact	6.17	0.45	3.05	0.30	11.43	10^{-6}	A			
		rs2046538999:G	ctcccagtcg	C→G	gcgccgccgc	314.57	24.61	201.35	15.04	8.25	10^{-6}	A			
		rs2046539239:T	ccagtcgcgc	G→T	ccgccgcccc	314.57	24.61	268.82	19.71	2.93	10^{-2}	C			
		rs2046541957:T	cccccgcccc	C→T	acccccaccc	314.57	24.61	120.01	12.29	14.95	10^{-6}	A			
		rs2046542067:A	cccgcccccа	C→A	ccccaccccc	475.82	47.90	244.01	19.91	10.31	10^{-6}	A			
		rs2046542510:T	acccccaccc	C→T	cacccccacc	314.57	24.61	143.16	11.04	14.33	10^{-6}	A			
		rs2046542756:T	cacccccacc	C→T	ccacccccag	314.57	24.61	142.96	12.20	13.63	10^{-6}	A			
		rs2046543098:T	ccacccccac	C→T	cccagccggc	314.57	24.61	159.08	13.49	11.82	10^{-6}	A			
		rs2046543533:A	cccccagccg	G→A	cgcccgcgcc	475.82	47.90	358.77	26.74	4.51	10^{-3}	B			
		rs2046544407:T	gcgcccgccc	C→T	cgcgccgggc	475.82	47.90	397.38	28.26	2.92	10^{-2}	C			
		rs2046544539:T	cgcccccgcg	C→T	cgggcccggc	475.82	47.90	324.17	24.37	6.11	10^{-6}	A			
		rs2046548465:A	gcggggccca	G→A	cgcactcgga	133.57	10.82	73.55	6.99	9.55	10^{-6}	A			
		rs2046548567:A	ggggcccagc	G→A	cactcggagc	133.57	10.82	90.06	6.30	7.36	10^{-6}	A			
		rs2046548708:A	cagcgcactc	G→A	gagcccgagc	133.57	10.82	52.79	4.12	16.49	10^{-6}	A			
		rs2046548918:T	gcactcggag	C→T	ccgagcccga	133.57	10.82	113.24	9.11	2.89	10^{-2}	C			
111	*PPARG* (5468)	rs2046575944:A	caccccgggg	G→A	gctggaaagg	79.65	6.19	69.13	5.40	2.57	0.05	D	↑	within human disease models using mice [337]: magnolol as principal ingredient of *Magnoliae officinalis* cortex, has anti-inflammatory and neuroprotective effects and relieves retinal damages and inflammation due to, mainly, Pparg-upregulation, which altogether can alleviate POAG	▲
		rs2046576183:T	gctggaaagg	C→T	gaagtcgctg	79.65	6.19	69.15	4.96	2.67	10^{-2}	C			
		rs2046877773:A	tgaatccttt	G→A	ctaataagtt	5.76	0.37	3.17	0.33	9.85	10^{-6}	A			
		rs2046912300:A	cctattttat	G→A	tattgttttt	2.86	0.26	1.23	0.12	12.65	10^{-6}	A			
		rs2047270080:A	gaacatgtgt	G→A	tatattgaaa	3.28	0.27	1.14	0.15	13.48	10^{-6}	A			
		rs2048482861:T	tgtttattcc	C→T	atctctccca	9.82	0.76	7.91	0.68	3.76	10^{-3}	B			
		rs2049567606:A	cctttgatat	C→A	aagcccttca	7.50	0.74	3.23	0.43	10.17	10^{-6}	A			
		rs2049572135:T	atttctactc	C→T	acattacgaa	7.50	0.74	4.96	0.46	6.12	10^{-6}	A			
		rs369867457:A	tttcaaatct	G→A	tagtgtcatt	5.42	0.52	4.35	0.47	3.06	10^{-2}	C			
		rs560460829:A	actcatgggt	G→A	tattcacaaa	9.82	0.76	6.65	0.79	5.51	10^{-6}	A			
		rs569533856:A	ccggggggct	G→A	gaaaggcgaa	79.65	6.19	14.94	1.37	27.87	10^{-6}	A			
		rs573670123:T	acttctccag	C→T	atttctactc	7.50	0.74	6.49	0.56	2.21	0.05	D			
		rs748150309:A	atgtaactta	C→A	aaacgcaagg	6.17	0.45	4.68	0.34	5.39	10^{-6}	A			
		rs867674907:T	cccccacccc	C→T	acccccaccc	314.57	24.61	90.48	9.07	19.59	10^{-6}	A			
		rs892089016:A	ctgaacatgt	G→A	tgtatattga	3.28	0.27	2.38	0.29	4.41	10^{-3}	B			
		rs990889498:C	cagttaaata	T→C	ttaccagcat	3.84	0.35	2.78	0.26	4.90	10^{-6}	A			
		rs1264926024:C	ctgcaccctt	T→C	aacttaaacc	5.66	0.35	6.35	0.61	2.05	0.05	D			
		rs1921412562:G	cgctgcaccc	T→G	ttaacttaaa	5.66	0.35	6.30	0.48	2.20	0.05	D			
		rs1921437524:A	gtaaacgccc	G→A	gggtgggagg	17.58	2.75	21.20	1.85	2.09	0.05	D	↓	within human disease models using Prnp-null mice [338]: increased intraocular pressure aggravating POAG	▼
		rs559032383:A	ggtaaacgcc	C→A	ggggtgggag	17.58	2.75	22.75	1.98	2.88	10^{-2}	C			
		rs879171850:C	tttaacttaa	A→C	cctcggccgg	5.66	0.35	6.99	0.77	3.35	10^{-3}	B			
		rs1034696966:A	ggcaggtaaa	C→A	gcccggggtg	17.58	2.75	10.96	0.95	5.29	10^{-6}	A			
112	*PRNP* (5621)	rs1051176596:T	ttccccgccc	C→T	gcgtccctcc	203.76	15.82	147.34	12.24	5.70	10^{-6}	A			
		rs113117765:T	cgcccgcgag	C→T	gccgccgctt	203.76	15.82	175.91	12.81	2.76	10^{-2}	C			
		rs1156237865:T	ttcccttccc	C→T	gccccgcgtc	203.76	15.82	180.25	13.92	2.24	0.05	D			
		rs1219803625:T	ttaacttaaa	C→T	ctcggccggc	5.66	0.35	4.09	0.50	4.76	10^{-3}	B	↑	within human eye disease models using proteome of neuroretina from both mouse and rat [339]: Prnp overexpression modulated by heat shock proteins as an attribute of a protective role of Prnp in the neuroretinal cellular stress response that may alleviate POAG	▲
		rs1356029546:A	acctccgccc	G→A	cgagcgccgc	203.76	15.82	143.22	11.97	6.18	10^{-6}	A			
		rs1380444454:A	ccccgccccg	C→A	gtccctcccc	203.76	15.82	178.42	14.11	2.40	0.05	D			
		rs1380444454:T	ccccgccccg	C→T	gtccctcccc	203.76	15.82	92.17	7.75	13.86	10^{-6}	A			
		rs1568527603:T	cgcgcgtcgc	C→T	tgtcctccga	192.38	14.89	69.79	5.54	18.28	10^{-6}	A			

Table S3. Cont.

#	Human Gene: *NCBI Gene Symbol* (NCBI Gene ID)	Candidate SNP marker: dbSNP ID:min [437]	5′ flank, 10 bp	WT →min	3 flank, 10 bp	K$_D$, nM, *in silico*: WT MEAN±	SEM	min MEAN±	SEM	Significance: Z	p	ϱ	Δ	Effect of changes in human gene expression on the development of primary open-angle glaucoma (POAG, ☼: "▼" aggravation, "▲" alleviation) [Reference]	☼
112	*PRNP* (5621)	rs1921412207:A	gcgctgcacc	C→A	tttaacttaa	5.66	0.35	4.80	0.53	2.59	10^{-2}	C	↑	within human eye disease models using proteome of neuroretina from both mouse and rat [339]: Prnp overexpression modulated by heat shock proteins as an attribute of a protective role of Prnp in the neuroretinal cellular stress response that may alleviate POAG	▲
		rs1921425963:T	cgccgcttcc	C→T	ttccccgccc	203.76	15.82	133.21	10.36	7.74	10^{-6}	A			
		rs1921427743:A	ccgcgtccct	C→A	cccctcggcc	203.76	15.82	83.95	8.59	13.81	10^{-6}	A			
		rs1921428864:A	cctcggcccc	G→A	cgcgtcgcct	203.76	15.82	126.01	10.37	8.49	10^{-6}	A			
		rs1921436500:T	acgaccgagg	C→T	aggtaaacgc	17.58	2.75	7.97	0.85	8.36	10^{-6}	A			
		rs935434196:A	gagcgccgcc	G→A	cttcccttcc	203.76	15.82	118.38	9.14	9.92	10^{-6}	A			
		rs940329839:T	gcctgtcctc	C→T	gagccagtcg	192.38	14.89	170.18	13.63	2.20	0.05	D			
		rs983151267:T	cttaaacctc	G→T	gccggccgcc	5.66	0.35	5.06	0.35	2.43	0.05	D			
113	*PTGFR* (5737)	rs1274007274:G	ctgccagcgc	A→G	gctaggtgca	37.07	3.55	42.97	4.19	2.16	0.05	D	↓	within a cohort-based biomedical study [340]: the effectiveness of the response to latanoprost as a drug against POAG decreases with a decrease in the transcriptional activity of the PTGFR gene as measured experimentally [341]	▼
		rs1648623406:G	tctgttagtg	T→G	gaatcattcc	9.75	0.85	11.51	1.26	2.37	0.05	D			
		rs1649374202:A	tctctcccag	T→A	actgctctgt	19.37	2.07	45.99	3.47	13.22	10^{-6}	A			
		rs1649377681:C	cgcagctagg	T→C	gcagagggat	37.07	3.55	63.68	5.71	8.24	10^{-6}	A			
		rs763340496:G	ctcccagtac	T→G	gctctgtgcc	19.37	2.07	35.42	2.83	9.05	10^{-6}	A			
		rs1170716646:A	cctcttctct	C→A	ccagtactgc	19.37	2.07	8.79	0.90	10.70	10^{-6}	A	↑	according to an exhaustive pharmaceutical review [342]: artificial PTGFR-agonists are drugs against POAG	▲
		rs1353522241:A	ctcttctctc	C→A	cagtactgct	19.37	2.07	10.13	1.24	7.97	10^{-6}	A			
		rs1557644643:T	cagctaggtg	C→T	agagggatcc	37.07	3.55	14.84	1.21	14.54	10^{-6}	A			
		rs1649374287:G	tctcccagta	C→G	tgctctgtgc	19.37	2.07	16.11	1.96	2.28	0.05	D			
		rs1649379113:T	ccgcagtttc	C→T	gcgctaaggg	37.07	3.55	31.54	2.47	2.61	10^{-2}	C			
		rs747836486:A	gtgccctctt	C→A	tctcccagta	19.37	2.07	11.64	1.01	7.41	10^{-6}	A			
114	*PTGS1* (5742)	rs1214320224:C	ctctcttggc	A→C	gggagggtgt	83.48	5.73	100.46	6.69	3.87	10^{-3}	B	↓	within human disease models using Ptgs1-knockout mice [343]: prostaglandin deficit in the corneal tissue, uveoscleral tissue, lens, retina and optic nerve, whereas exogenous recombinant prostaglandins and their synthetic analogues (e.g., latanoprost) are drugs against POAG [344]	▼
		rs1286709163:C	gcacgcacag	G→C	agcctgcact	44.99	3.85	55.70	4.88	3.49	10^{-3}	B			
		rs1328701749:G	gcgcacgcac	A→G	ggagcctgca	44.99	3.85	76.78	6.53	8.87	10^{-6}	A			
		rs1328701749:T	gcgcacgcac	A→T	ggagcctgca	44.99	3.85	61.04	5.18	5.07	10^{-6}	A			
		rs1836757120:G	agccgaggtg	A→G	cagctggagg	84.80	6.24	149.13	10.66	11.01	10^{-6}	A			
		rs1836764216:T	gcacaggagc	C→T	tgcactctgc	44.99	3.85	55.78	4.94	3.49	10^{-3}	B			
		rs1837058612:C	cagcctctct	T→C	ggcagggagg	83.48	5.73	103.16	8.37	3.98	10^{-3}	B			
		rs755810343:T	gcgaggcgca	C→T	gcacaggagc	44.99	3.85	59.42	4.66	4.79	10^{-3}	B			
		rs761911171:C	aagtccttcc	T→C	gttagatctg	12.84	1.36	16.48	1.43	3.65	10^{-3}	B			
		rs1006705764:T	ctgcactctg	C→T	gtcccgcacc	44.99	3.85	34.89	3.10	4.12	10^{-3}	B	↑	within human POAG pharmaceutical cellular models using human embryonic kidney cell line HEK-293 treated with an artificial low molecular weight analog AGN008 of the human prostaglandin E receptor 4, PTGER4, as a new promising anti-POAG medication [345]: PTGS1-upregulation along with reduced intraocular pressure	▲
		rs1038480670:T	tcttggcagg	G→T	agggtgtggg	83.48	5.73	39.67	3.71	12.82	10^{-6}	A			
		rs1193614506:A	agtccttcct	G→A	ttagatctgt	12.84	1.36	6.54	0.60	9.65	10^{-6}	A			
		rs1200525505:A	acaggagcct	G→A	cactctgcgt	44.99	3.85	25.65	2.41	8.85	10^{-6}	A			
		rs1222444121:A	cgcacgcaca	G→A	gagcctgcac	44.99	3.85	20.28	1.59	13.73	10^{-6}	A			
		rs1231909301:A	tggcagggag	G→A	gtgtgggcag	83.48	5.73	68.36	4.92	4.02	10^{-3}	B			
		rs1283681848:A	tgtgcgaggc	G→A	cacgcacagg	44.99	3.85	34.73	3.37	4.01	10^{-3}	B			
		rs1286709163:A	gcacgcacag	G→A	agcctgcact	44.99	3.85	26.50	2.18	8.91	10^{-6}	A			
		rs1300377967:A	ggccggactg	G→A	gagggaggag	83.48	5.73	74.76	5.56	2.18	0.05	D			
		rs1431476771:A	cagccgaggt	G→A	acagctggag	84.80	6.24	25.90	2.14	21.44	10^{-6}	A			
		rs1564126738:A	gggatgggct	G→A	gagctccggg	78.00	7.25	66.38	6.67	2.36	0.05	D			
		rs1836756745:A	gcgggcagcc	G→A	aggtgacagc	84.80	6.24	75.18	5.35	2.35	0.05	D			
		rs1836757240:T	gaggtgacag	C→T	tggagggagg	84.80	6.24	43.76	3.52	12.15	10^{-6}	A			
		rs1836760621:A	gggaggggat	G→A	ggctggagct	84.80	6.24	68.55	6.66	3.49	10^{-3}	B			
		rs1836761822:T	gagctccggg	C→T	agtgtgcgag	78.00	7.25	28.70	2.71	15.09	10^{-6}	A			
		rs1836763387:T	ggcgcacgca	C→T	aggagcctgc	44.99	3.85	18.70	1.81	13.59	10^{-6}	A			
		rs1837057755:T	agctcccgca	C→T	agcctctctt	83.48	5.73	65.61	6.85	3.85	10^{-3}	B			

Table S3. Cont.

Human Gene		Candidate SNP marker				K$_D$, nM, *in silico*				Significance				Effect of changes in human gene expression on the development of primary open-angle glaucoma (POAG, ☼: "▼" aggravation, "▲" alleviation) [Reference]	☼
						WT		min							▲
#	*NCBI Gene Symbol* (NCBI Gene ID)	dbSNP ID:min [437]	5′ flank, 10 bp	WT →min	3 flank, 10 bp	MEAN±	SEM	MEAN±	SEM	Z	p	ϱ	Δ		▼
114	*PTGS1* (5742)	rs1837058429:G	cacagcctct	C→G	ttggcaggga	83.48	5.73	68.15	5.00	4.04	10^{-3}	B	↑	within human POAG pharmaceutical cellular models using human embryonic kidney cell line HEK-293 treated with an artificial low molecular weight analog AGN008 of the human prostaglandin E receptor 4, PTGER4, as a new promising anti-POAG medication [345]: PTGS1-upregulation along with reduced intraocular pressure	▲
		rs1837063620:A	cgttccagct	T→A	cagcgtctgg	83.48	5.73	37.49	3.74	13.23	10^{-6}	A			
		rs374663959:A	gtgggcagcc	G→A	ttccagcttc	83.48	5.73	70.97	4.56	3.45	10^{-3}	B			
		rs374663959:T	gtgggcagcc	G→T	ttccagcttc	83.48	5.73	75.03	4.58	2.32	0.05	D			
		rs762034320:A	ggcagggagg	G→A	tgtgggcagc	83.48	5.73	51.08	4.02	9.41	10^{-6}	A			
		rs777103314:A	cgaggcgcac	G→A	cacaggagcc	44.99	3.85	24.10	2.39	9.55	10^{-6}	A			
		rs777103314:T	cgaggcgcac	G→T	cacaggagcc	44.99	3.85	31.50	2.64	5.95	10^{-6}	A			
		rs796300296:T	cgcacagcct	C→T	tcttggcagg	83.48	5.73	47.49	3.34	11.48	10^{-6}	A			
		rs889811433:A	ctcagctccc	G→A	cacagcctct	83.48	5.73	59.62	5.29	6.00	10^{-6}	A			
		rs907355047:A	ccagcttcag	C→A	gtctggagct	83.48	5.73	59.65	4.28	6.77	10^{-6}	A			
		rs907355047:T	ccagcttcag	C→T	gtctggagct	83.48	5.73	58.65	4.25	7.07	10^{-6}	A			
		rs908536503:T	gttccagctt	C→T	agcgtctgga	83.48	5.73	58.83	4.83	6.54	10^{-6}	A			
		rs941338247:A	cccgcacagc	C→A	tctcttggca	83.48	5.73	46.29	3.41	11.71	10^{-6}	A			
		rs943540469:A	ggcagtgtgc	G→A	aggcgcacgc	78.00	7.25	65.14	4.84	3.03	10^{-2}	C			
		rs943540469:T	ggcagtgtgc	G→T	aggcgcacgc	78.00	7.25	49.17	5.34	6.45	10^{-6}	A			
		rs973565118:T	gtgcgaggcg	C→T	acgcacagga	44.99	3.85	23.73	2.25	10.02	10^{-6}	A			
		rs991383048:A	agcctctctt	G→A	gcagggaggg	83.48	5.73	27.09	2.57	19.23	10^{-6}	A			
115	*RHOA* (387)	rs1004903505:G	ccttgacctg	A→G	tggcccctga	58.15	3.78	67.91	4.73	3.26	10^{-2}	C	↓	according to an exhaustive review [346]: drug-induced therapeutic inhibition of RHOA can decrease aqueous humor drainage via the trabecular meshwork in the drainage pathway that can alleviate POAG	▲
		rs1005606653:A	gcgcacgtcg	T→A	cactcggagg	72.64	5.27	92.09	7.93	4.21	10^{-3}	B			
		rs1005606653:G	gcgcacgtcg	T→G	cactcggagg	72.64	5.27	91.72	7.73	4.19	10^{-3}	B			
		rs1014786227:G	ctctcgcgct	A→G	ccgccctcgc	71.94	5.12	110.74	8.32	8.34	10^{-6}	A			
		rs1020367859:G	cgctctctcg	C→G	ccgcccgccc	71.94	5.12	89.89	6.67	4.33	10^{-3}	B			
		rs1254326303:T	cactcggagg	C→T	agcccgctgg	72.64	5.27	81.38	6.71	2.07	0.05	D			
		rs1313674504:G	atgagctgtg	A→G	agcaatagtg	29.03	2.44	68.29	5.20	15.10	10^{-6}	A			
		rs1350951076:G	cccgccctcg	C→G	tcccgccgcc	71.94	5.12	96.51	7.66	5.51	10^{-6}	A			
		rs1415116171:G	ccgccctcgc	T→G	cccgccgccg	71.94	5.12	88.09	6.69	3.89	10^{-3}	B			
		rs1487322740:G	ggaggcgcgc	A→G	gctgggcact	72.64	5.27	109.81	8.37	7.85	10^{-6}	A			
		rs1488183218:C	tctctcgcgc	T→C	cccgccctcg	71.94	5.12	123.94	8.97	10.72	10^{-6}	A			
		rs1575297520:T	gcccctggcc	C→T	gtggccgagc	58.15	3.78	65.14	4.15	2.50	0.05	D			
		rs2048939736:C	cctgaccctt	G→C	cgcccctggc	58.15	3.78	69.30	5.03	3.60	10^{-3}	B			
		rs2048948700:C	tcgctctctc	G→C	gccgcccgcc	71.94	5.12	86.11	6.48	3.47	10^{-3}	B			
		rs2048948930:G	gccctcgctc	T→G	cgccgccgcc	71.94	5.12	112.65	10.96	7.44	10^{-6}	A			
		rs2048949538:T	ccgccgcccg	C→T	ccgccctccc	71.94	5.12	80.60	5.68	2.27	0.05	D			
		rs2048953669:C	gtggatgagc	T→C	cctgagcaat	29.03	2.44	49.71	4.01	9.24	10^{-6}	A			
		rs2048953739:G	atagtggatg	A→G	gctcctgagc	29.03	2.44	33.41	2.80	2.37	0.05	D			
		rs543411304:A	ggatgagctg	T→A	tgagcaatag	29.03	2.44	68.29	5.20	15.10	10^{-6}	A			
		rs554642123:C	cgcgcacgtc	G→C	gcactcggag	72.64	5.27	108.64	9.33	7.16	10^{-6}	A			
		rs903240544:A	gagctgtgag	T→A	caatagtgga	29.03	2.44	42.27	3.38	6.49	10^{-6}	A			
		rs972865053:T	cttgacctga	C→T	ggcccctgac	58.15	3.78	67.91	4.78	3.24	10^{-2}	C			
		rs978554175:G	cctcgctctc	T→G	ccgccgcccg	71.94	5.12	100.54	7.22	6.62	10^{-6}	A			

Table S3. Cont.

Human Gene		Candidate SNP marker				K_D, nM, *in silico*				Significance				Effect of changes in human gene expression on the development of primary open-angle glaucoma (POAG, ☼: "▼" aggravation, "▲" alleviation) [Reference]	☼ ▲ ▼
#	*NCBI Gene Symbol* (NCBI Gene ID)	dbSNP ID:min [437]	5′ flank, 10 bp	WT→min	3 flank, 10 bp	WT MEAN	± SEM	min MEAN	± SEM	Z	p	ϱ	Δ		
		rs1016234448:T	tgacccttga	C→T	cccctggccc	58.15	3.78	40.85	2.58	7.80	10^{-6}	A			
		rs1020367859:T	cgctctctcg	C→T	ccgcccgccc	71.94	5.12	26.12	2.16	18.59	10^{-6}	A			
		rs1032906032:T	cgccctcgct	C→T	ccgccgccgc	71.94	5.12	38.57	2.59	12.76	10^{-6}	A			
		rs1165834463:T	cgtcgttccc	C→T	ggaggcgcgc	72.64	5.27	64.26	4.38	2.46	0.05	D			
		rs1190248210:A	aggcgcgcac	G→A	tgggcactcg	68.29	5.20	51.54	3.72	5.36	10^{-6}	A			
		rs1239733403:T	tctcgcgcta	C→T	cgccctcgct	71.94	5.12	59.64	5.16	3.35	10^{-3}	B			
		rs1271281906:T	gacttaacac	C→T	gatgcccagg	12.42	1.23	8.72	0.69	5.57	10^{-6}	A			
		rs1284387246:G	ctctctcgcg	C→G	gcccgccctc	71.94	5.12	56.23	4.12	4.83	10^{-3}	B			
		rs1284387246:T	ctctctcgcg	C→T	gcccgccctc	71.94	5.12	35.84	3.32	11.94	10^{-6}	A			
		rs1350951076:T	cccgccctcg	C→T	tcccgccgcc	71.94	5.12	57.15	4.12	4.55	10^{-3}	B			
		rs1398686711:A	gacctgtgcc	G→A	cgcggggccg	68.29	5.20	56.74	4.48	3.38	10^{-6}	A			
		rs1411188108:T	gcgcgcacgt	C→T	ggcactcgga	68.29	5.20	33.80	2.42	13.45	10^{-6}	A			
		rs1442866204:A	cccggtcact	G→A	cggtgataca	12.42	1.23	4.08	0.41	15.79	10^{-6}	A			
		rs1459168076:A	ccgcggtcct	C→A	cctcccgccg	71.94	5.12	30.99	2.94	14.19	10^{-6}	A			
		rs185825300:A	gcccgccctc	G→A	ctcccgccgc	71.94	5.12	61.16	4.44	3.19	10^{-2}	C			
		rs185825300:C	gcccgccctc	G→C	ctcccgccgc	71.94	5.12	60.48	4.33	3.44	10^{-3}	B			
		rs185825300:T	gcccgccctc	G→T	ctcccgccgc	71.94	5.12	52.68	3.82	6.14	10^{-6}	A			
		rs2048939736:A	cctgaccctt	G→A	cgcccctggc	58.15	3.78	19.04	1.45	22.30	10^{-6}	A			
		rs2048940694:T	accggctgct	C→T	ggccgcgccc	58.15	3.78	50.05	4.37	2.76	10^{-2}	C			
115	*RHOA* (387)	rs2048948591:T	gctctctcgc	G→T	cgcccgccct	71.94	5.12	50.40	4.87	5.93	10^{-6}	A	↑	within human neuroregeneration models using rats subjected with artificial sciatic transection injuries [347]: post-injury activated GTP-bound RhoA dramatically upregulated in both neurons and axons, which shown a RhoA-dose-related increase in the initiation of neurite outgrowth, and in the proportion with long neurites in the extent and distance of axons, and partnered Schwann cell regrowth	▲
		rs2048948876:G	ccctcgctct	C→G	gccgccgccc	71.94	5.12	54.37	3.99	5.48	10^{-6}	A			
		rs2048950835:G	cgcacgtcgt	T→G	actcggaggc	72.64	5.27	65.13	4.65	2.14	0.05	D			
		rs2048951265:C	tcggaggcgc	G→C	ccgctgggca	72.64	5.27	54.23	4.07	5.60	10^{-6}	A			
		rs2048951757:A	tgggcactcg	G→A	ctcgagcccg	72.64	5.27	62.08	4.50	3.06	10^{-2}	C			
		rs2048952561:T	gcgacctgtg	C→T	tgcgcggggc	68.29	5.20	46.69	3.53	7.09	10^{-6}	A			
		rs2048952631:A	gccgcgacct	G→A	gcgtgcgcgg	68.29	5.20	24.42	2.44	16.36	10^{-6}	A			
		rs2048953515:A	tgagctgtga	G→A	gcaatagtgg	29.03	2.44	23.83	1.91	3.40	10^{-3}	B			
		rs554642123:A	cgcgcacgtc	G→A	gcactcggag	68.29	5.20	46.66	3.42	7.20	10^{-6}	A			
		rs574275589:A	ctcgcgctac	C→A	gccctcgctc	71.94	5.12	30.46	3.07	13.94	10^{-6}	A			
		rs574275589:T	ctcgcgctac	C→T	gccctcgctc	71.94	5.12	60.17	4.79	3.35	10^{-3}	B			
		rs576523908:T	gctgggcact	C→T	ggctcgagcc	72.64	5.27	59.63	4.91	3.60	10^{-3}	B			
		rs750468556:T	ggagacttaa	C→T	ctggatgccc	12.42	1.23	4.11	0.40	15.90	10^{-6}	A			
		rs917298149:G	gacccttgac	C→G	ccctggcccc	58.15	3.78	39.27	2.35	8.90	10^{-6}	A			
		rs927327466:T	tcgcgctacc	C→T	ccctcgctct	71.94	5.12	56.11	5.15	4.28	10^{-3}	B			
		rs967641817:A	ctcgctctct	C→A	cgccgcccgc	71.94	5.12	26.68	2.12	18.59	10^{-6}	A			
		rs967641817:T	ctcgctctct	C→T	cgccgcccgc	71.94	5.12	43.32	3.21	9.87	10^{-6}	A			
		rs978554175:A	cctcgctctc	T→A	ccgccgcccg	71.94	5.12	38.49	2.90	12.08	10^{-6}	A			
		rs982961993:T	acgtcgttcc	C→T	cggaggcgcg	72.64	5.27	63.12	4.29	2.83	10^{-2}	C			
		rs990437260:A	cgcccgccct	C→A	cctcccgccg	71.94	5.12	62.60	5.48	2.47	0.05	D			

Table S3. Cont.

Human Gene		Candidate SNP marker				K$_D$, nM, *in silico*				Significance				Effect of changes in human gene expression on the development of primary open-angle glaucoma (POAG, ☼: "▼" aggravation, "▲" alleviation) [Reference]	☼
#	*NCBI Gene Symbol* (NCBI Gene ID)	dbSNP ID:min [437]	5′ flank, 10 bp	WT →min	3 flank, 10 bp	WT MEAN	WT ± SEM	min MEAN	min ± SEM	Z	p	ϱ	Δ		▲ ▼
116	*ROCK1* (6093)	rs1191127678:A	cagagtgcgc	G>A	tgcggaggcg	49.04	3.86	54.94	4.19	2.07	0.05	D	↓	within human POAG cellular models using spontaneously arising retinal pigment epithelial cell line ARPE-19 [348]: miR-136-5p inhibits ROCK1-expression that relieves inflammatory retina injury in POAG	▲
		rs2036759593:C	ggcgacagag	T>C	caccctgcgg	49.04	3.86	74.13	6.03	7.30	10^{-6}	A			
		rs893571953:G	cggaggcgac	A>G	accccaccct	49.04	3.86	129.53	9.60	17.97	10^{-6}	A			
		rs995850037:T	cctgcggagg	C>T	ccccacccca	49.04	3.86	56.30	3.81	2.66	10^{-2}	C			
		rs1568365779:A	aaggccatga	G>A	ccaaaatcac	9.16	1.03	6.82	0.74	3.79	10^{-3}	B	↑	according to an exhaustive pharmaceutical review [349]: netarsudil as a low molecular weight ROCK1-antagonist is an eye drop medication against POAG	▼
		rs1598568012:A	ggaggcgaca	G>A	ccccaccctg	49.04	3.86	20.76	1.52	16.00	10^{-6}	A			
		rs2036757899:A	ggttcccctt	C>A	gggtcccggc	49.04	3.86	32.68	3.00	6.71	10^{-6}	A			
117	*ROCK2* (9475)	rs1018336255:G	atcattcttt	A→G	tgacctgact	6.37	0.48	7.29	0.70	2.20	0.05	D	↓	within a pharmaceutical study using mice [350]: fasudil as a low molecular weight ROCK2-inhibitor is medication against POAG as well as against hypertension, heart failure, stroke, brain and spinal cord injuries	▲
		rs1396002909:G	aatgacataa	A→G	ttcctggtga	3.65	0.25	4.45	0.36	3.72	10^{-3}	B			
		rs1665143976:C	gatcattctt	T→C	ctgacctgac	6.37	0.48	7.29	0.70	2.20	0.05	D			
		rs1667245449:G	tgaaaatgac	A→G	cattttcctg	3.65	0.25	4.62	0.39	4.36	10^{-3}	B			
		rs540292547:C	ttttcattac	A→C	acattgactt	2.56	0.28	5.33	0.65	9.05	10^{-6}	A			
		rs540292547:G	ttttcattac	A→G	acattgactt	2.56	0.28	3.42	0.44	3.46	10^{-3}	B			
		rs965991626:A	ttattttcat	T→A	aatacattga	2.56	0.28	3.73	0.32	5.49	10^{-6}	A			
		rs1016980121:T	ctcacatagt	C→T	tccataattt	6.37	0.48	4.52	0.44	5.54	10^{-6}	A	↑	within a cohort-based biomedical study [351]: ROCK2 excess in the human lens epithelial cells seems to be a biomedically proven molecular marker for increased protein aggregates aggravating POAG	▼
		rs1046328237:A	gggcttgtgg	G→A	tgcgggcgga	60.93	6.07	33.24	3.16	8.80	10^{-6}	A			
		rs1188873019:C	ctgcgggcgg	A→C	cgcgccggcg	60.93	6.07	42.40	4.27	5.12	10^{-6}	A			
		rs1409437347:T	ttgtgggtag	C→T	ggcggagggg	60.93	6.07	39.53	3.85	6.21	10^{-6}	A			
		rs1426530957:A	attctttaat	G→A	cctgactgat	6.37	0.48	2.84	0.28	13.02	10^{-6}	A			
		rs371053193:T	attttcatta	C→T	tacattgact	2.56	0.28	0.89	0.11	12.79	10^{-6}	A			
		rs927590809:A	gggcggaggg	G→A	ccggcggctg	60.93	6.07	23.34	2.06	14.43	10^{-6}	A			
		rs927590809:T	gggcggaggg	G→T	ccggcggctg	60.93	6.07	23.74	2.00	14.46	10^{-6}	A			
118	*SEC14L2* (23541)	rs1474967438:C	aggttggagg	T→C	aggtgtggct	18.37	1.86	39.36	3.16	11.79	10^{-6}	A	↓	within human disease cellular models using human SEC14L2-knockout COS-7 cell line generated based on CRISPR–Cas9 technology [352]: impaired endosome fission and accumulation that can cause age-related exfoliation syndrome aggravating POAG in old age [353]	▼
		rs1933824632:C	ttggaggtag	G→C	tgtggctgtc	18.37	1.86	28.51	2.79	6.23	10^{-6}	A			
		rs1239168970:A	aggtaggtgt	G→A	gctgtccatt	18.37	1.86	13.47	1.18	4.63	10^{-3}	B	↑	within the pharmaceutical search for promising cell cultures as an ex vivo test-system for the design of vaccines against the hepatitis C virus [354]: SEC14L2 overexpression can increase vitamin E levels, which may alleviate POAG [355]	▲
		rs1320979275:T	agaggttgga	G→T	gtaggtgtgg	18.37	1.86	13.18	1.20	4.88	10^{-3}	B			
		rs1933760899:T	ggcacacttg	C→T	tcgcctcctg	51.72	3.24	37.28	2.54	7.07	10^{-6}	A			
		rs1933761550:T	gcctgggtct	C→T	tgacttcagg	51.72	3.24	34.88	2.43	8.40	10^{-6}	A			
		rs1933761626:A	ctgggtctct	G→A	acttcaggag	51.72	3.24	20.41	1.79	17.23	10^{-6}	A			
		rs1933763530:C	gcccgtgtca	G→C	agccttccgc	51.72	3.24	45.98	3.62	2.33	0.05	D			
		rs1933786716:A	gccgctgagg	G→A	cacaaaggca	26.18	1.91	21.15	1.60	4.05	10^{-3}	B			
		rs1933787097:A	agggcacaaa	G→A	gcaacattca	26.18	1.91	18.02	1.28	7.34	10^{-6}	A			
		rs542014248:A	tcagagcctt	C→A	cgcgccccag	51.72	3.24	40.73	4.23	3.94	10^{-3}	B			
		rs908906126:A	gggcccgtgt	C→A	agagccttcc	51.72	3.24	11.83	1.24	24.14	10^{-6}	A			
		rs911430490:A	ctctcgggat	C→A	ctttaagagg	9.42	0.79	3.68	0.33	15.36	10^{-6}	A			
		rs953251137:A	gcggaggggg	C→A	ccgtgtcaga	51.72	3.24	43.51	3.69	3.28	10^{-2}	C			
		rs956569686:A	ggtctctgac	T→A	tcaggagggc	51.72	3.24	34.88	2.10	9.06	10^{-6}	A			
		rs978496680:A	gggggcccgt	G→A	tcagagcctt	51.72	3.24	16.79	1.85	17.76	10^{-6}	A			
119	*SERPINE1* (5054)	rs1385468370:T	cccacatctg	G→T	tataaaagga	1.53	0.16	1.86	0.20	2.63	10^{-2}	C	↓	within human POAG cellular model using human trabecular meshwork cells HTM cultivated and treated with TGF-beta2 relieving POAG [356]: increased fibrillar extracellular matrix aggravating POAG along with serpin E1 deficit relatively to those at BMP7 treatment alleviating POAG	▼

Table S3. Cont.

#	Human Gene: *NCBI Gene Symbol* (NCBI Gene ID)	Candidate SNP marker: dbSNP ID:min [437]	5′ flank, 10 bp	WT →min	3 flank, 10 bp	K_D, nM, *in silico*: WT MEAN	± SEM	min MEAN	± SEM	Significance: Z	p	ϱ	Δ	Effect of changes in human gene expression on the development of primary open-angle glaucoma (POAG, ☼: "▼" aggravation, "▲" alleviation) [Reference]	☼ ▲ ▼
120	*SIRT1* (23411)	rs1038103295:C	cgtgatgggg	T→C	ttaaatctcc	6.57	0.61	11.40	1.23	7.75	10^{-6}	A	↓	within cohort-based biomedical trabeculectomy study using RT-qPCR [358]: Sirtuin 1 deficiency in the trabecular meshwork tissue can be a biomedical molecular marker for susceptibility to POAG	▼
		rs1038103295:G	cgtgatgggg	T→G	ttaaatctcc	6.57	0.61	11.23	1.07	8.05	10^{-6}	A			
		rs1292411190:C	agtaagtaga	T→C	gatagcacta	4.76	0.44	5.69	0.70	2.30	0.05	D			
		rs1396067593:A	gcggcggcgg	C→A	gattgggtac	43.88	4.48	50.58	4.74	2.05	0.05	D			
		rs1589070016:G	aagggatggt	A→G	tttatgctcg	3.70	0.34	7.99	0.78	11.51	10^{-6}	A			
		rs1842443743:T	gtttaaatct	C→T	ccgcagccgg	6.57	0.61	8.44	0.82	3.73	10^{-3}	B			
		rs1842572302:C	atggtattta	T→C	gctcgccttg	3.70	0.34	7.07	0.68	9.78	10^{-6}	A			
		rs1842815212:G	ggtaaggaag	T→G	aagtagatga	4.76	0.44	5.69	0.70	2.30	0.05	D			
		rs200894138:C	tggggtttaa	A→C	tctcccgcag	6.57	0.61	10.36	1.09	6.52	10^{-6}	A			
		rs562769017:G	gtaaggaagt	A→G	agtagatgat	4.76	0.44	5.69	0.70	2.30	0.05	D			
		rs92924702:T	aagtaagtag	A→T	tgatagcact	4.76	0.44	5.69	0.70	2.30	0.05	D			
		rs985627974:C	aggaagtaag	T→C	agatgatagc	4.76	0.44	5.69	0.70	2.30	0.05	D			
		rs995058732:C	gtgatggggt	T→C	taaatctccc	6.57	0.61	11.76	1.36	7.87	10^{-6}	A			
		rs1198565310:A	cgaggaggag	G→A	aagaggcggc	43.88	4.48	33.52	2.53	4.24	10^{-3}	B	↑	within human neuroprotection models using C57BL/6 mice infected with a neurotropic strain of mouse hepatitis virus MHV-A59 inducing a multiple-sclerosis-like neuronal loss and, next, treated orally with an artificial compound SRTAW04 as a new promising neuroprotector [359]: Sirt1 upregulation along with prevented neuronal loss during inflammatory demyelinating optic nerve lesion that can neuroprotectantly alleviate POAG	▲
		rs1456604162:C	taaggaagta	A→C	gtagatgata	4.76	0.44	4.16	0.39	2.07	0.05	D			
		rs1842443491:A	tcacgtgatg	G→A	ggtttaaatc	6.57	0.61	5.46	0.44	3.00	10^{-2}	C			
		rs1842572334:A	tggtatttat	G→A	ctcgccttgc	3.70	0.34	1.43	0.16	13.46	10^{-6}	A			
		rs561608453:A	ggggtttaaa	T→A	ctcccgcagc	6.57	0.61	5.51	0.52	2.66	10^{-2}	C			
		rs865826245:A	cgacgacgac	G→A	agggcgagga	43.88	4.48	34.65	2.63	3.71	10^{-3}	B			
		rs970556821:A	gtaccgaggt	G→A	cgcagggtgc	43.88	4.48	35.09	4.13	2.87	10^{-2}	C			
121	*SIX1* (6495)	rs1188829108:A	gaaattcaaa	T→A	agacttcaca	15.13	0.95	19.76	1.32	5.83	10^{-6}	A	↓	within human POAG models using transgenic mice [360]: Six1 deficiency in eye can be a biomedical molecular marker for susceptibility to POAG	▼
		rs1217093977:C	cgctgcgcgc	T→C	cgtgccgggg	18.36	1.79	37.12	4.08	9.58	10^{-6}	A			
		rs1217093977:G	cgctgcgcgc	T→G	cgtgccgggg	18.36	1.79	26.80	2.44	5.67	10^{-6}	A			
		rs1486921548:T	aaattcaaat	A→T	gacttcacag	15.13	0.95	25.47	1.60	11.73	10^{-6}	A			
		rs1002436790:A	gacgctgcgc	G→A	agcgtgccgg	18.36	1.79	12.15	1.16	6.06	10^{-6}	A	↑	according to an exhaustive biomedical review [361]: SIX1 overexpression can slow down the apoptosis as the most important event in retinal ganglion cells during therapy against POAG in line with a cohort-based biomedical study [362]	▲
		rs1257709075:T	cgctttaagg	C→T	gggacgctgc	18.36	1.79	15.08	1.12	3.21	10^{-2}	C			
		rs1260277899:T	ttcaaataga	G→T	ttcacaggaa	15.13	0.95	7.92	0.69	12.11	10^{-6}	A			
		rs1464999993:A	gctttaaggc	C→A	ggacgctgcg	18.36	1.79	15.26	1.51	2.67	10^{-2}	C			
		rs1464999993:T	gctttaaggc	C→T	ggacgctgcg	18.36	1.79	14.67	1.39	3.30	10^{-3}	B			
		rs1895170243:A	acaggaaatt	C→A	gtcaagactt	15.13	0.95	9.74	0.64	9.69	10^{-6}	A			
		rs906759092:A	gcgctttaag	G→A	ggggacgctg	18.36	1.79	12.72	1.25	5.32	10^{-6}	A			
122	*SIX6* (4990)	rs1181105707:A	cccggtgact	G→A	acagggggtc	33.91	2.15	16.63	1.67	12.00	10^{-6}	A	↑	within human neuroregeneration models using embryonic chick retinal pigmented epithelium subjected with an artificial injury [363]: SIX6 excess along with transient dedifferentiation of retinal pigmented epithelium reprogrammed to neuroepithelium, which can differentiate to neural retina cells as neuroregeneration alleviating POAG	▲
		rs1893248031:A	ttcaagcttt	G→A	attggcagag	33.91	2.15	9.58	0.81	23.96	10^{-6}	A			
		rs1893248031:T	ttcaagcttt	G→T	attggcagag	33.91	2.15	7.61	0.72	26.13	10^{-6}	A			
		rs950670025:G	tgctgattca	A→G	gctttgattg	33.91	2.15	29.76	2.16	2.71	10^{-2}	C			
123	*SLC4A10* (57282)	rs1039408113:C	ctgtatttta	T→C	actagtagcc	0.88	0.09	2.90	0.25	17.79	10^{-6}	A	↓	within human disease models using Slc4a10-deficient mice [364]: impaired visual function in retina that can aggravate POAG	▼
		rs1221268801:G	gcttgctgta	T→G	tttatactag	0.88	0.09	1.61	0.19	7.64	10^{-6}	A			
		rs1332908599:G	attttatact	A→G	gtagccaggt	0.88	0.09	1.73	0.18	9.35	10^{-6}	A			
		rs2031815610:C	tgctgtattt	T→C	atactagtag	0.88	0.09	1.70	0.22	7.96	10^{-6}	A			
		rs2031836957:G	tgatccgaat	A→G	ctaagcagag	7.01	0.87	27.15	2.15	18.40	10^{-6}	A			
		rs922309254:T	tgtattttat	A→T	ctagtagcca	0.88	0.09	3.37	0.27	20.73	10^{-6}	A			

Table S3. Cont.

Human Gene		Candidate SNP marker				K_D, nM, *in silico*				Significance				Effect of changes in human gene expression on the development of primary open-angle glaucoma (POAG, ☼: "▼" aggravation, "▲" alleviation) [Reference]	☼
#	*NCBI Gene Symbol* (NCBI Gene ID)	dbSNP ID:min [437]	5′ flank, 10 bp	WT →min	3 flank, 10 bp	WT MEAN±	WT SEM	min MEAN±	min SEM	Z	p	ρ	Δ		▲▼
123	*SLC4A10* (57282)	rs1284123096:T	agcccgagtg	A→T	gtaggagcca	27.72	2.12	14.04	1.46	10.55	10^{-6}	A	↑	according to a comprehensive biomedical review [365]: a contrast sensitivity level decreases with a decrease Slc4a10 level within a mice model of POAG as well as a visual acuity level increases with an increase in contrast sensitivity level within a cohort-based biomedical study [366]	▲
		rs1342961165:T	gaatactaag	C→T	agagcgagtg	7.01	0.87	5.84	0.56	2.33	0.05	D			
		rs1476941545:A	agcagcccga	G→A	tgagtaggag	27.72	2.12	19.23	1.52	6.66	10^{-6}	A			
		rs2031828643:A	cagcccgagt	G→A	agtaggagcc	27.72	2.12	7.82	0.77	20.34	10^{-6}	A			
		rs2031830045:A	gagtgagtag	G→A	agccatgtga	27.72	2.12	23.01	2.01	3.22	10^{-2}	C			
		rs2031833313:A	ctggtgagtt	G→A	gagtgtgcaa	27.72	2.12	14.43	1.27	11.22	10^{-6}	A			
		rs2031835251:T	cctcctgctt	C→T	agagctacct	27.72	2.12	14.84	1.54	9.72	10^{-6}	A			
		rs899759983:A	gatccgaata	C→A	taagcagagc	7.01	0.87	5.73	0.49	2.67	10^{-2}	C			
124	*SLC23A2* (9962)	rs1199795748:G	cagaacaaaa	A→G	gtgggtccag	12.14	0.79	14.21	1.03	3.22	10^{-2}	C	↓	according to a cohort-based biomedical study [367]: SLC23A2 deficit in tears, aqueous humor, and blood is a biomedical molecular marker for susceptibility to POAG	▼
		rs1220551043:G	gggagggagg	T→G	ggtcgggggc	175.52	14.02	196.28	14.03	2.09	0.05	D			
		rs1256739176:G	gggggcgggg	A→G	gggcaggggt	175.52	14.02	196.28	15.78	1.97	0.05	D			
		rs1292971478:C	gagggaggtg	G→C	tcgggggcgg	175.52	14.02	196.28	15.81	1.97	0.05	D			
		rs1314700493:G	gggtccagac	A→G	caatccactg	12.14	0.79	15.39	0.99	5.19	10^{-6}	A			
		rs1338662964:A	ccagacagaa	C→A	ccactgtggg	12.14	0.79	19.14	1.31	9.63	10^{-6}	A			
		rs1479631834:G	gcggggaggg	A→G	aggggtcggg	175.52	14.02	196.28	15.78	1.97	0.05	D			
		rs755738686:G	tgcccacagc	A→G	gaccctttc	25.57	2.29	32.11	2.44	3.88	10^{-3}	B			
		rs1171851016:A	ggcggggagg	G→A	caggggtcgg	175.52	14.02	106.91	7.45	9.36	10^{-6}	A	↑	within human POAG cellular models using an immortalized human lens epithelial cell line HLE-B3 under exposure to the chemical oxidant tert-butylhydroperoxide [368]: a n immediate SLC23A2 up-regulation improving Na+-dependent transport of vitamin C (i.e., ascorbic acid) as the most important antioxidant into the normal lens epithelium in order to prevent eye damages under oxidant stressors	▲
		rs1256382525:T	cacagcacta	C→T	cttttcctgc	25.57	2.29	14.56	1.44	8.46	10^{-6}	A			
		rs1292971478:A	gagggaggtg	G→A	tcgggggcgg	175.52	14.02	103.52	7.96	9.52	10^{-6}	A			
		rs1305920455:T	cccagaccgg	C→T	gggaggtggg	175.52	14.02	134.07	10.68	4.78	10^{-3}	B			
		rs1310196028:A	aggggaggtgg	G→A	cgggggcggg	175.52	14.02	146.20	11.70	3.23	10^{-2}	C			
		rs1371676666:T	gggccccaga	C→T	gggagggagg	175.52	14.02	101.31	6.81	10.53	10^{-6}	A			
		rs1394981906:T	ctccgctgca	C→T	ggggcgggcg	175.52	14.02	123.93	10.29	6.04	10^{-6}	A			
		rs1467709361:A	gggcggggag	G→A	gcaggggtcg	175.52	14.02	104.17	7.48	9.71	10^{-6}	A			
		rs1600222498:A	ggagggaggt	G→A	gtcgggggcg	175.52	14.02	51.67	4.91	19.70	10^{-6}	A			
		rs2088126716:A	ggctccgctg	C→A	cgggggcggg	175.52	14.02	146.19	12.86	3.08	10^{-2}	C			
		rs2088127322:A	ggccccagac	C→A	ggagggaggt	175.52	14.02	76.61	6.23	14.54	10^{-6}	A			
		rs914394177:A	gccccagacc	G→A	gagggaggtg	175.52	14.02	128.12	10.16	5.59	10^{-6}	A			
		rs926590713:T	cggctccgct	G→T	gcgggggcgg	175.52	14.02	93.84	7.38	11.17	10^{-6}	A			
125	*SLCO2A1* (6578)	rs1204991953:G	ggccacttat	A→G	tcatcggcgg	2.53	0.29	6.69	0.64	13.03	10^{-6}	A	↓	within human health care models using rat type 1 alveolar epithelial cell-like cell line AT1-L subjected with cigarette smoke extract [369]: Slco2a1-deficit can elevate susceptibility to oxidant stress, which can lead to detrimental effects on ocular health, namely: POAG, cataract, and age-related macular degeneration as three most often causes of vision loss [370]	▼
		rs1236477144:C	ggcggccact	T→C	tcatcatcgg	2.53	0.29	6.53	0.52	13.59	10^{-6}	A			
		rs1576467252:A	cggccactta	T→A	atcatcggcg	2.53	0.29	7.13	0.83	12.70	10^{-6}	A			
		rs1576467252:C	cggccactta	T→C	atcatcggcg	2.53	0.29	7.40	0.61	15.23	10^{-6}	A			
		rs535245455:C	gcggccactt	A→C	catcatcggc	2.53	0.29	8.58	0.67	17.67	10^{-6}	A			
		rs535245455:G	gcggccactt	A→G	catcatcggc	2.53	0.29	6.62	0.63	12.90	10^{-6}	A			
126	*SPARC* (6678)	rs1333469468:G	ggggagatag	A→G	cacggggtgg	23.62	2.12	29.56	2.63	3.55	10^{-3}	B	↓	within human disease models using Sparc-knockout mice [372]: Sparc-null mice exhibit lower intraocular pressures, while the best way to manage POAG is by lowering the intraocular pressure [373]	▲
		rs1336361494:G	gaggggagat	A→G	accacggggt	23.62	2.12	82.24	6.04	21.52	10^{-6}	A			
		rs1761154985:G	gttgcctgtc	T→G	agtggtttcc	15.68	1.39	23.62	2.12	6.50	10^{-6}	A			
		rs528241179:G	aggactggta	A→G	gggatggagt	10.03	0.98	11.47	1.06	1.99	0.05	D			
		rs1761156441:C	gggtggaggg	G→C	gagggaccac	23.62	2.12	19.45	1.74	3.07	10^{-2}	C	↑	within cohort-based biomedical RNA-Seq study using postmortem donor ocular tissue within and without POAG in donor's anamnesis [374]: SPARC-excess seems to be a biomedically proven molecular marker for POAG	▼
		rs939776406:T	gggagataga	C→T	acggggtgga	23.62	2.12	20.49	2.16	2.06	0.05	D			

Table S3. Cont.

Human Gene		Candidate SNP marker				K_D, nM, *in silico*				Significance				Effect of changes in human gene expression on the development of primary open-angle glaucoma (POAG, ☼: "▼" aggravation, "▲" alleviation) [Reference]	☼ ▲ ▼
						WT		min							
#	*NCBI Gene Symbol* (NCBI Gene ID)	dbSNP ID:min [437]	5′ flank, 10 bp	WT →min	3 flank, 10 bp	MEAN±	SEM	MEAN±	SEM	Z	p	ϱ	Δ		
		rs1029850854:C	agtttgctac	T→C	gggttgtgca	18.83	1.83	25.46	2.15	4.68	10^{-3}	B			
		rs1161490546:G	tcagtttgct	A→G	ctgggttgtg	18.83	1.83	30.70	4.37	5.68	10^{-6}	A			
		rs1414052335:C	agcttcagtt	T→C	gctactgggt	18.83	1.83	30.70	4.34	5.69	10^{-6}	A			
		rs1414052335:G	agcttcagtt	T→G	gctactgggt	18.83	1.83	30.53	2.26	7.91	10^{-6}	A	↓	within human Alzheimer's disease models using transgenic mice carrying microglia-specific miR-155 knockdown [375]: Spp1 downregulation mitigated retinal inflammation and vasculopathy as protective effects against vascular damages during Alzheimer's disease and associated neurodegenerative ocular diseases, including POAG	▲
		rs1725350535:G	gcagcagcat	T→G	taaattctgg	5.79	0.63	7.24	0.94	2.65	10^{-2}	C			
		rs548703562:C	gtggcagaaa	A→C	cctcatgaca	30.73	2.24	39.57	2.73	5.04	10^{-6}	A			
		rs979149978:C	cagcttcagt	T→C	tgctactggg	18.83	1.83	29.50	3.94	5.43	10^{-6}	A			
		rs1194170753:A	aaaacctcat	G→A	acacaatctc	30.73	2.24	13.84	1.19	14.14	10^{-6}	A			
		rs1309305039:A	ccgcctccct	G→A	tgttggtgga	30.73	2.24	24.32	2.58	3.63	10^{-3}	B			
127	*SPP1* (6696)	rs1450820031:T	cctcatgaca	C→T	aatctctccg	30.73	2.24	14.52	1.22	13.46	10^{-6}	A			
		rs1725344522:T	cagaggggga	A→T	gtgtgggagc	30.73	2.24	25.24	2.11	3.54	10^{-3}	B			
		rs1725344725:A	agggggaagt	G→A	tgggagcagg	30.73	2.24	22.86	2.21	4.88	10^{-3}	B			
		rs1725350435:T	tctgcagcag	C→T	atttaaattc	5.79	0.63	3.95	0.39	5.25	10^{-6}	A	↑	within a cohort-based biomedical RNA-Seq analysis of human corneoscleral tissues taken from postmortem human donor eyes with versus without glaucoma [376]: SPP1-excess can be a biomedical molecular biomarker of POAG	▼
		rs1725350816:A	gcatttaaat	T→A	ctgggagggc	5.79	0.63	2.61	0.28	10.44	10^{-6}	A			
		rs1725355749:T	gccgaccaag	G→T	tacagcttca	18.83	1.83	12.12	1.33	6.02	10^{-6}	A			
		rs563729787:G	gcagaaaacc	T→G	catgacacaa	30.73	2.24	26.06	1.92	3.18	10^{-2}	C			
		rs947906819:T	ctccgcctcc	C→T	tgtgttggtg	30.73	2.24	24.17	1.84	4.56	10^{-3}	B			
		rs968846642:T	gaccaaggta	C→T	agcttcagtt	18.83	1.83	10.52	1.32	7.33	10^{-6}	A			
		rs1016436357:T	gccgaacgag	C→T	acctctgggt	12.18	0.90	13.64	1.01	2.17	0.05	D	↓	according to an exhaustive mini-review letter [377]: STAT3 gene silencing can contribute to both origin and development of POAG and its severity among patients with diabetes	
		rs1016658693:A	agccccaacc	G→A	ccgagggaac	89.52	6.34	67.82	5.39	5.22	10^{-6}	A			
		rs1049643066:A	aattatgcat	G→A	ggcctttcat	15.15	1.78	11.37	1.00	3.91	10^{-3}	B			
		rs1179457149:A	cagactggga	G→A	ctgcggcggc	105.37	7.43	88.97	5.81	3.52	10^{-3}	B			
		rs1193030559:A	gctggccttt	C→A	tggccgaacg	12.18	0.90	8.70	0.59	6.74	10^{-6}	A			
		rs1229962633:A	ccgggggttc	C→A	gggaggggga	105.37	7.43	51.31	3.91	13.86	10^{-6}	A			
		rs1229962633:T	ccgggggttc	C→T	gggaggggga	105.37	7.43	57.95	4.23	11.78	10^{-6}	A			
		rs1242441519:A	actgggactt	G→A	tgtttacccc	9.27	1.43	7.76	0.70	1.99	0.05	D			
		rs1415033362:A	gtaattcagc	G→A	ctgaaggggc	105.37	7.43	92.56	7.99	2.33	0.05	D			
		rs1460196765:A	cgcagccgag	G→A	ggttccgacg	105.37	7.43	53.74	3.62	13.80	10^{-6}	A			
		rs1460196765:T	cgcagccgag	G→T	ggttccgacg	105.37	7.43	55.23	4.19	12.47	10^{-6}	A			
		rs2082800161:A	tgggacttgt	G→A	tttacccctа	9.27	1.43	6.67	0.66	3.60	10^{-3}	B			
		rs2085235237:T	cgtcgcagcc	G→T	gggggttccg	105.37	7.43	77.10	6.59	5.64	10^{-6}	A			
128	*STAT3* (6774)	rs2085235511:T	tccgacgtcg	C→T	gagccggggg	105.37	7.43	31.75	3.36	18.87	10^{-6}	A			
		rs2085237283:A	cgcagactgg	G→A	agctgcggcg	105.37	7.43	74.18	5.09	7.13	10^{-6}	A			
		rs2085237685:C	cggcggcgca	G→C	ttccggagct	105.37	7.43	65.57	4.94	9.19	10^{-6}	A	↑	within human POAG model using rats subjected with a chronic artificially elevated intraocular pressure [378]: STAT3 overexpression in the optic nerve head seems to be a candidate molecular marker for the very earlier stages of the POAG origin and development	▼
		rs2085244476:A	agctggcctt	T→A	gtggccgaac	12.18	0.90	9.69	0.88	3.90	10^{-3}	B			
		rs566151688:A	agactgggag	G→A	tgcggcggcg	105.37	7.43	94.78	6.82	2.10	0.05	D			
		rs566151688:T	agactgggag	G→T	tgcggcggcg	105.37	7.43	93.20	6.91	2.40	0.05	D			
		rs753416867:T	gctgaagggg	C→T	ggcgaggatt	15.15	1.78	12.79	1.28	2.19	0.05	D			
		rs765882801:G	tcagcggttt	C→G	ggggctgtaa	105.37	7.43	94.79	7.33	2.02	0.05	D			
		rs771250401:A	gtgaacatat	G→A	tgggacttgt	9.27	1.43	3.23	0.36	11.06	10^{-6}	A			
		rs778835967:A	tgcacacttt	G→A	tggtgaacat	9.27	1.43	6.03	0.63	4.63	10^{-3}	B			
		rs780393027:A	ggagccgggg	G→A	gactgggagg	105.37	7.43	72.66	4.84	7.66	10^{-6}	A			
		rs902564848:T	gcgcagactg	G→T	gagctgcggc	105.37	7.43	37.34	2.77	20.27	10^{-6}	A			
		rs903725268:A	agcggtttcc	G→A	ggctgtaatt	105.37	7.43	65.70	5.37	8.75	10^{-6}	A			
		rs928113019:T	gttccgacgt	C→T	gggagccggg	105.37	7.43	86.19	6.19	3.99	10^{-3}	B			
		rs935406613:A	ctgcggcggc	G→A	ggtttccgga	105.37	7.43	60.64	4.32	11.02	10^{-6}	A			
		rs939563262:T	ggggttccga	C→T	agggggagcc	105.37	7.43	64.84	4.26	10.07	10^{-6}	A			

Table S3. Cont.

#	Human Gene: NCBI Gene Symbol (NCBI Gene ID)	Candidate SNP marker: dbSNP ID:min [437]	5′ flank, 10 bp	WT →min	3 flank, 10 bp	K_D, nM, *in silico*: WT MEAN	± SEM	min MEAN	± SEM	Significance: Z	p	ρ	Δ	Effect of changes in human gene expression on the development of primary open-angle glaucoma (POAG, ☼: "▼" aggravation, "▲" alleviation) [Reference]	☼ ▲▼
		rs1329303922:C	cagccgccag	T→C	gttccctccc	62.32	5.04	94.69	6.94	7.67	10^{-6}	A			
		rs1411984966:A	ttggaagtag	G→A	gcaggccact	5.11	0.54	6.11	0.60	2.49	0.05	D			
		rs1420665270:A	gcgttatcta	G→A	tttggaagta	5.11	0.54	6.71	0.69	3.70	10^{-3}	B	↓	within human disease cellular models using the human melanoma cells cultured with the synthetic nanomeric peptide IT9302 homologous to the C-terminal of the human IL-10 (i.e., AYMTMKIRN) [379]: TAP1 inhibited along with a IT9302-dose-dependent increase in sensitivity to lysis by natural killer cells of anticancer innate immune response to melanoma, which is comorbid to POAG in line with a comprehensive review [380]	▲
		rs1770972460:G	cccagccgcc	A→G	tcgttccctc	62.32	5.04	94.69	6.94	7.67	10^{-6}	A			
		rs775610295:C	cgccagtagg	G→C	cctcccccag	62.32	5.04	74.12	6.25	2.97	10^{-2}	C			
		rs1310587111:T	gccccgccct	C→T	actttccgag	62.32	5.04	50.14	3.30	4.17	10^{-3}	B			
		rs1408055208:T	gcaggatcag	C→T	ttctcgccag	62.32	5.04	48.34	3.27	4.82	10^{-3}	И			
		rs1435614741:T	tcccccagcc	G→T	ccctcgttcc	62.32	5.04	35.59	4.96	6.95	10^{-6}	A			
129	*TAP1* (6890)	rs202053684:T	tcagcctgtt	C→T	ccagcgcagg	62.32	5.04	40.43	3.02	7.86	10^{-6}	A			
		rs746658627:T	aggactcggc	G→T	gccagtaggg	51.81	4.45	43.72	5.09	2.35	0.05	D			
		rs762944665:A	ctcccccagc	C→A	gccctcgttc	62.32	5.04	48.50	3.81	4.44	10^{-3}	B			
		rs763963785:T	gccagtaggg	G→T	ctcccccagc	51.81	4.45	27.62	2.44	10.21	10^{-6}	A	↑	within human diabetes mellitus models using mice [381]: Tap1 overexpression may be a candidate molecular marker for diabetes mellitus, which is comorbid with POAG, at least due to their mutual comorbidity with diabetic retinopathy [198]	▼
		rs775610295:A	cgccagtagg	G→A	cctcccccag	62.32	5.04	37.24	3.18	8.76	10^{-6}	A			
		rs777221320:T	ggactcggcg	G→T	ccagtagggg	51.81	4.45	41.73	4.35	3.20	10^{-2}	C			
		rs866383207:T	ccgccagtag	G→T	ccctccccca	62.32	5.04	18.27	2.05	17.74	10^{-6}	A			
		rs866625088:A	gccgccagta	G→A	tccctccccc	62.32	5.04	38.31	2.85	8.85	10^{-6}	A			
		rs866625088:T	gccgccagta	G→T	tccctccccc	62.32	5.04	26.73	3.11	11.94	10^{-6}	A			
		rs866782773:A	agcgcaggat	C→A	agcttctcgc	62.32	5.04	31.70	2.95	10.96	10^{-6}	A			
		rs1180760909:C	gtagctggcg	G→C	cctggccgag	53.34	5.52	63.29	6.39	2.37	0.05	D			
		rs1307282028:T	tttttttttt	A→T	ggattttttt	7.67	0.52	9.39	0.72	3.93	10^{-3}	B			
		rs146973617:C	ctggccgagc	G→C	tgacgcccgc	53.34	5.52	84.36	7.39	6.76	10^{-6}	A			
		rs1769589543:G	tttaagcacg	A→G	tttttttttt	7.67	0.52	8.65	0.63	2.40	0.05	D	↓	within human disease cellular models using the human melanoma cells cultured with the synthetic nanomeric peptide IT9302 homologous to the C-terminal of the human IL-10 (i.e., AYMTMKIRN) [379]: TAP2 inhibited along with a IT9302-dose-dependent increase in sensitivity to lysis by natural killer cells of anticancer innate immune response to melanoma, which is comorbid to POAG in line with a comprehensive review [380]	▲
		rs1769622925:C	tggccgagcg	T→C	gacgcccgcc	53.34	5.52	103.73	7.85	10.37	10^{-6}	A			
		rs1769623820:G	gccctggccg	A→G	ccctgacgcc	53.34	5.52	64.60	7.87	2.40	0.05	D			
		rs1161486415:T	gaccagagcc	G→T	cgtagctggc	53.34	5.52	41.28	4.00	3.61	10^{-3}	B			
		rs1170481549:T	agccggtagc	G→T	tggcggacca	53.34	5.52	24.83	2.38	10.83	10^{-6}	A			
		rs1283883289:A	tttttttttt	T→A	ggggattttt	7.67	0.52	2.53	0.26	17.99	10^{-6}	A			
130	*TAP2* (6891)	rs146973617:A	ctggccgagc	G→A	tgacgcccgc	53.34	5.52	28.89	3.06	8.27	10^{-6}	A			
		rs1582595134:A	tttttttttt	T→A	cggggatttt	7.67	0.52	4.28	0.36	10.84	10^{-6}	A			
		rs1769590318:A	tttttttttt	T→A	gggattttttt	7.67	0.52	5.48	0.44	6.36	10^{-6}	A			
		rs1769617135:T	gagcggacct	C→T	tgggagagac	53.34	5.52	38.36	3.33	4.88	10^{-3}	B	↑	within human transcriptome meta-analysis related to inflammatory bowel disease, most frequent among which are Crohn's disease and ulcerative colitis [382]: TAP2 excess along with abnormal immune response to intestinal tract microbiota that can provoke through microbiota-gut-retina axis autoimmunity impact to POAG pathogenesis [383]	▼
		rs1769618751:A	ggttgggaga	G→A	gccggtagcg	53.34	5.52	41.55	3.23	3.86	10^{-3}	B			
		rs1769623556:T	cctggccgag	C→T	ctgacgcccg	53.34	5.52	24.48	2.59	10.53	10^{-6}	A			
		rs1769630331:C	atgaaggcct	T→C	cccagcccag	34.66	2.52	31.06	2.27	2.13	0.05	D			
		rs907426345:T	cggcccagcc	C→T	ccccctcccg	34.66	2.52	29.37	2.15	3.21	10^{-2}	C			
		rs942611607:A	gagccggtag	C→A	ctggcggacc	53.34	5.52	31.33	3.06	7.48	10^{-6}	A			
		rs942611607:T	gagccggtag	C→T	ctggcggacc	53.34	5.52	32.49	3.04	7.10	10^{-6}	A			

Table S3. Cont.

Human Gene		Candidate SNP marker				K_D, nM, *in silico*				Significance				Effect of changes in human gene expression on the development of primary open-angle glaucoma (POAG, ☼: "▼" aggravation, "▲" alleviation) [Reference]	☼ ▲ ▼
						WT		min							
#	*NCBI Gene Symbol* (NCBI Gene ID)	dbSNP ID:min [437]	5′ flank, 10 bp	WT →min	3 flank, 10 bp	MEAN	± SEM	MEAN	± SEM	Z	p	ϱ	Δ		
131	*TBK1* (29110)	rs1002561866:C	ccctgttacc	T→C	gcatccccgc	20.82	2.03	24.78	2.22	2.63	10^{-2}	C			▲
		rs1156432910:C	gcgcgggccc	T→C	gttacctgca	20.82	2.03	23.98	2.09	2.16	0.05	D			
		rs1159191611:C	agacgggcca	T→C	aggcgtgcgc	45.05	4.70	55.92	4.38	3.31	10^{-3}	B			
		rs1159191611:G	agacgggcca	T→G	aggcgtgcgc	45.05	4.70	55.92	4.38	3.31	10^{-3}	B			
		rs1195947053:C	cggccgtcgg	T→C	cacgtggcct	55.92	4.38	106.08	8.62	11.35	10^{-6}	A			
		rs1236811069:G	ggggtgtgtg	T→G	cgggtggccg	28.35	2.65	37.75	3.33	4.46	10^{-3}	B			
		rs1239997964:G	agataagtac	T→G	ggcatataat	3.21	0.33	3.87	0.41	2.49	0.05	D			
		rs1278490004:G	cattaataag	T→G	catgcaaaaa	3.38	0.37	4.01	0.53	2.01	0.05	D			
		rs1290797902:C	tctgctctta	T→C	taatgtcctt	3.81	0.39	5.28	0.65	4.09	10^{-3}	B			
		rs1358649560:C	ctcttattaa	T→C	gtcctttttc	3.81	0.39	5.75	0.44	6.50	10^{-6}	A			
		rs1404740198:C	ctgtggtgac	T→C	accagacggg	24.37	2.47	45.05	4.70	8.45	10^{-6}	A			
		rs1592347528:G	tgactaccag	A→G	cgggccatag	28.16	2.83	40.66	4.07	5.18	10^{-6}	A			
		rs1592347662:G	gccggaagtg	T→G	cctgagtctc	85.73	5.79	99.98	7.18	3.12	10^{-2}	C	↓	according to an exhaustive retrospective review [384]: TBK1-inhibitors are considered as promising drugs for adjuvant therapy in POAG	
		rs2040429995:G	ggctgtggtg	A→G	ctaccagacg	24.37	2.47	36.88	3.52	5.95	10^{-6}	A			
		rs2040430124:G	ggtgactacc	A→G	gacgggccat	24.37	2.47	38.66	3.80	6.54	10^{-6}	A			
		rs2040436127:C	ctggggtgtg	T→C	gtcgggtggc	28.35	2.65	74.96	5.06	16.87	10^{-6}	A			
		rs2040946291:C	attgaccagt	T→C	aaaagaaaat	7.62	0.54	8.90	0.81	2.68	10^{-2}	C			
		rs573225756:T	gctgtggtga	C→T	taccagacgg	24.37	2.47	36.97	4.10	5.55	10^{-6}	A			
		rs61933192:C	gggccctgtt	A→C	cctgcatccc	20.82	2.03	28.35	2.65	4.57	10^{-3}	B			
		rs756395249:C	tttctgctct	T→C	attaatgtcc	3.81	0.39	6.89	0.56	9.09	10^{-6}	A			
		rs777807444:G	gctcttatta	A→G	tgtccttttt	3.81	0.39	4.89	0.53	3.36	10^{-3}	B			
		rs777807444:T	gctcttatta	A→T	tgtccttttt	3.81	0.39	5.16	0.33	5.06	10^{-6}	A			
		rs892981786:C	ggccataggc	G→C	tgcgcacgcg	45.05	4.70	55.92	4.38	3.31	10^{-3}	B			
		rs906970894:T	cgtcggtcac	G→T	tggcctccgg	55.92	4.38	79.73	5.28	6.92	10^{-6}	A			
		rs916286526:G	aagcccatta	A→G	taagtcatgc	3.38	0.37	4.68	0.68	3.58	10^{-3}	B			
		rs1021125988:C	taccagacgg	G→C	ccataggcgt	45.05	4.70	37.88	3.59	2.46	0.05	D			
		rs1021125988:T	taccagacgg	G→T	ccataggcgt	45.05	4.70	35.70	3.50	3.25	10^{-2}	C			
		rs1187318445:A	cttgtttatt	T→A	tattagtatg	7.62	0.54	6.23	0.69	3.07	10^{-2}	C			
		rs1279452194:A	cttattttat	T→A	ctgcttttgt	3.38	0.37	1.67	0.17	9.28	10^{-6}	A			
		rs1281546005:A	cggctgtggt	G→A	actaccagac	24.37	2.47	10.40	1.21	11.04	10^{-6}	A			
		rs1334135665:T	ggccctgtta	C→T	ctgcatcccc	20.82	2.03	10.41	1.00	10.10	10^{-6}	A			
		rs1344148724:T	ccagacgggc	C→T	ataggcgtgc	28.16	2.83	17.87	1.90	6.21	10^{-6}	A			
		rs1401880607:A	acgggccata	G→A	gcgtgcgcac	33.59	3.19	20.39	2.01	7.28	10^{-6}	A			
		rs1401880607:C	acgggccata	G→C	gcgtgcgcac	45.05	4.70	25.96	2.52	7.74	10^{-6}	A			
		rs1403802271:A	cgcgggccct	G→A	ttacctgcat	20.82	2.03	11.38	1.02	9.13	10^{-6}	A			
		rs141593283:T	cctgagtctc	G→T	aggaggccgc	85.73	5.79	34.99	3.36	15.26	10^{-6}	A	↑	within human antiviral innate immune response models using duck embryonic fibroblasts transfected with a recombinant plasmid with or without another copy of the Tbk1 gene and 24 hours later incubated with duck enteritis virus [385]: excess Tbk1 may enhance the antiviral innate immune response, which may prevent viral conjunctivitis as a dangerous complication after glaucoma surgery [386]	
		rs1416550043:A	cgtggacact	G→A	cctctggggt	28.35	2.65	19.46	2.22	5.11	10^{-6}	A			
		rs1422844057:A	aaaggtgctt	G→A	tttattttat	7.62	0.54	4.13	0.37	10.70	10^{-6}	A			
		rs1424897528:G	agctgaaatt	A→G	ttatttataa	2.34	0.19	2.01	0.20	2.37	0.05	D			
		rs1452010143:T	ttacttgaga	C→T	taaaaacagc	7.78	0.71	5.63	0.43	5.43	10^{-6}	A			
		rs1476023795:T	tgcctctggg	G→T	tgtgtgtcgg	28.35	2.65	22.42	1.95	3.67	10^{-3}	B			
		rs1565808793:A	cagggcttgc	G→A	aagccggaag	85.73	5.79	70.70	5.54	3.73	10^{-3}	B			
		rs1592348042:T	gccctgttac	C→T	tgcatccccg	20.82	2.03	17.72	1.72	2.34	0.05	D			
		rs1592350694:A	tcttagctgt	G→A	ttactccctt	7.78	0.71	5.66	0.84	3.65	10^{-3}	B			
		rs1592377498:T	ttgaccagtt	A→T	aaagaaaata	7.62	0.54	6.02	0.50	4.35	10^{-3}	B			

Table S3. Cont.

Human Gene		Candidate SNP marker				K_D, nM, *in silico*				Significance				Effect of changes in human gene expression on the development of primary open-angle glaucoma (POAG, ☼: "▼" aggravation, "▲" alleviation) [Reference]	☼ ▲ ▼
						WT		min							
#	*NCBI Gene Symbol* (NCBI Gene ID)	dbSNP ID:min [437]	5′ flank, 10 bp	WT →min	3 flank, 10 bp	MEAN±	SEM	MEAN±	SEM	Z	p	ϱ	Δ		
131	*TBK1* (29110)	rs1691133937:A	gtggtgacta	C→A	cagacgggcc	24.37	2.47	17.61	1.69	4.66	10^{-3}	B	↑	within human antiviral innate immune response models using duck embryonic fibroblasts transfected with a recombinant plasmid with or without another copy of the Tbk1 gene and 24 hours later incubated with duck enteritis virus [385]: excess Tbk1 may enhance the antiviral innate immune response, which may prevent viral conjunctivitis as a dangerous complication after glaucoma surgery [386]	▲
		rs2040430434:A	accagacggg	C→A	cataggcgtg	28.16	2.83	17.76	1.58	6.86	10^{-6}	A			
		rs2040430618:A	gggccatagg	C→A	gtgcgcacgc	45.05	4.70	26.51	2.44	7.61	10^{-6}	A			
		rs2040431519:T	cggtcacgtg	G→T	cctccggcca	55.92	4.38	43.76	3.62	4.31	10^{-3}	B			
		rs2040431711:T	ccggccaggg	C→T	ttgcgaagcc	85.73	5.79	69.22	5.49	4.11	10^{-3}	B			
		rs2040431939:A	gaagccggaa	G→A	tgtcctgagt	85.73	5.79	42.21	3.68	12.85	10^{-6}	A			
		rs2040480414:A	taacatttgt	T→A	tcttagctgt	7.78	0.71	5.16	0.48	6.28	10^{-6}	A			
		rs2040480790:T	gtgttactcc	C→T	ttaaaacata	7.78	0.71	5.96	0.36	4.87	10^{-3}	B			
		rs2040579732:A	tggcatataa	T→A	caaaatttat	3.21	0.33	2.15	0.27	4.89	10^{-3}	B			
		rs2040918501:A	gttaatttat	T→A	tgagtttttc	3.81	0.39	2.28	0.21	7.43	10^{-6}	A			
		rs2040946490:T	aaagaaaata	A→T	aacataaaca	7.62	0.54	4.47	0.28	11.20	10^{-6}	A			
		rs2040946952:A	tttgcatact	G→A	tttttgagca	7.62	0.54	6.14	0.52	3.91	10^{-3}	B			
		rs544456975:T	gcttgcgaag	C→T	cggaagtgtc	85.73	5.79	69.27	5.85	3.94	10^{-3}	B			
		rs749409737:A	tcttattaat	G→A	tcctttttca	3.81	0.39	2.38	0.23	6.79	10^{-6}	A			
		rs768966851:A	cgcggctgtg	G→A	tgactaccag	24.37	2.47	20.07	1.98	2.75	10^{-2}	C			
		rs892768308:G	gactaccaga	C→G	gggccatagg	24.37	2.47	19.02	1.88	3.51	10^{-3}	B			
		rs892768308:T	gactaccaga	C→T	gggccatagg	24.37	2.47	20.72	2.20	2.21	0.05	D			
		rs906970894:A	cgtcggtcac	G→A	tggcctccgg	55.92	4.38	35.92	2.73	8.11	10^{-6}	A			
		rs914445468:A	cgtagccggt	G→A	gaatggtcgc	28.35	2.65	19.05	1.70	6.16	10^{-6}	A			
		rs921883897:T	ctgcctctgg	G→T	gtgtgtgtcg	28.35	2.65	15.75	1.43	9.01	10^{-6}	A			
		rs931043370:A	cagctaacat	T→A	tgtttcttag	7.78	0.71	6.59	0.57	2.62	10^{-2}	B			
		rs931770956:A	tggggtgtgt	G→A	tcgggtggcc	28.35	2.65	12.95	1.32	11.35	10^{-6}	A			
		rs962928306:T	tgtcctgagt	C→T	tcgaggaggc	85.73	5.79	63.10	3.85	6.74	10^{-6}	A			
132	*TCF4* (6925)	rs1002345389:T	tttaagttgg	A>T	tctcttttag	6.16	0.48	7.27	0.49	3.24	10^{-2}	C	↓	within human disease models using neuron-specific Tcf4-knockdown adult mice (because of Tcf4-knockout animals are perinatally lethal) [387]: higher risks of both hyperexcitability and increased dendritic complexity of neurons contributing schizophrenia, which is comorbid to POAG as age-related neurodegenerative diseases [388]	▼
		rs1010233657:T	ggatgaatca	G>T	ctcctccagt	28.46	2.14	34.49	3.29	3.17	10^{-2}	C			
		rs1011702706:T	ctttaaacca	C>T	agtctttaac	12.79	0.97	15.47	1.13	3.60	10^{-3}	B			
		rs1034028752:C	ctttaactct	T>C	tggtagggag	12.79	0.97	16.95	1.13	5.57	10^{-6}	A			
		rs1042114850:C	ctctaggcgc	T>C	cttggccgcc	15.33	1.37	17.60	1.54	2.21	0.05	D			
		rs1052794946:A	ttttaagttg	G>A	ttctctttta	6.16	0.48	6.98	0.48	2.42	0.05	D			
		rs1052794946:C	ttttaagttg	G>C	ttctctttta	6.16	0.48	7.07	0.50	2.65	10^{-2}	C			
		rs1158928479:C	catcatctaa	G>C	tcttttcatt	11.01	0.91	14.52	0.97	5.20	10^{-6}	A			
		rs1169286283:T	ttttaatatt	A>T	gcaaactttt	1.87	0.24	2.72	0.23	4.90	10^{-6}	A			
		rs1171337239:C	ttctctttat	A>C	gccgcagctc	2.54	0.33	8.80	0.87	15.20	10^{-6}	A			
		rs1171337239:G	ttctctttat	A>G	gccgcagctc	2.54	0.33	7.59	0.78	13.16	10^{-6}	A			
		rs1175495243:C	gtgaatagtg	T>C	gtattttgca	9.98	0.79	12.70	1.02	4.27	10^{-3}	B			
		rs1210880988:C	agaagagagt	T>C	cccctccccg	12.04	0.88	13.90	1.01	2.79	10^{-2}	C			
		rs1219428180:T	ttatttgaat	C>T	gtgccgtatt	6.80	0.58	10.76	0.73	8.47	10^{-6}	A			
		rs1233945994:C	ccagtgggat	G>C	ttggagctcc	28.46	2.14	32.14	2.99	2.04	0.05	D			
		rs1251404574:A	gagtctttaa	C>A	ttaatggtag	12.79	0.97	16.08	1.27	4.18	10^{-3}	B			
		rs1270465240:C	ctcgaataaa	T>C	ggggatgtaa	8.63	0.59	10.94	0.91	4.42	10^{-3}	B			
		rs1298713209:C	cgtattgtta	T>C	ttctcttgtg	6.80	0.58	8.63	0.65	4.20	10^{-3}	B			
		rs1338686525:G	tgctgggtct	A>G	atcccttgtc	23.10	1.72	39.11	3.32	9.32	10^{-6}	A			
		rs1358417684:G	gtctcatcct	A>G	ccctcctccc	28.08	2.14	41.87	2.93	7.73	10^{-6}	A			
		rs1359222017:C	cagccccctt	T>C	aggcagcttt	24.75	1.68	34.55	2.51	6.71	10^{-6}	A			
		rs1359222017:G	cagccccctt	T>G	aggcagcttt	24.75	1.68	34.55	2.51	6.71	10^{-6}	A			

Table S3. Cont.

**Human Gene**		**Candidate SNP marker**				**K_D, nM, *in silico***				**Significance**				**Effect of changes in human gene expression on the development of primary open-angle glaucoma (POAG, ☼: "▼" aggravation, "▲" alleviation) [Reference]**	☼ ▲ ▼
						**WT**		**min**							
#	*NCBI Gene Symbol* (NCBI Gene ID)	**dbSNP ID:min [437]**	**5′ flank, 10 bp**	**WT →min**	**3 flank, 10 bp**	**MEAN±**	**SEM**	**MEAN±**	**SEM**	**Z**	**p**	**ϱ**	**Δ**		
132	*TCF4* (6925)	rs1361983258:G	tcgaggaact	A>G	atcggcatta	3.85	0.28	6.30	0.54	8.73	10^{-6}	A	↓	within human disease models using neuron-specific Tcf4-knockdown adult mice (because of Tcf4-knockout animals are perinatally lethal) [387]: higher risks of both hyperexcitability and increased dendritic complexity of neurons contributing schizophrenia, which is comorbid to POAG as age-related neurodegenerative diseases [388]	▼
		rs1380739357:C	taaactcgaa	T>C	gtgaggggat	8.63	0.59	12.31	1.05	6.51	10^{-6}	A			
		rs1391010086:C	tccagtggga	T>C	cttggagctc	28.46	2.14	32.14	2.99	2.04	0.05	D			
		rs1426534379:C	atccactaga	T>C	aggtgtgcat	7.70	0.92	14.54	1.35	8.39	10^{-6}	A			
		rs1446172153:C	attttttttt	T>C	aactgaagtt	22.01	1.50	24.96	1.99	2.40	0.05	D			
		rs145207252:G	ctttcttact	A>G	accccctgtat	3.02	0.28	11.54	0.93	21.83	10^{-6}	A			
		rs1466000828:C	atttgcagcc	T>C	gcatcacgtg	2.43	0.32	3.40	0.40	3.78	10^{-3}	B			
		rs1478271220:G	atgtactact	A>G	gcaactcttt	1.54	0.15	3.06	0.38	8.71	10^{-6}	A			
		rs1568475643:A	tttaccactc	T>A	taggcgctct	18.05	1.54	26.77	2.12	6.78	10^{-6}	A			
		rs1569451403:T	acaacagttt	A>T	agattcacgc	10.86	0.97	21.20	1.41	12.00	10^{-6}	A			
		rs1598841696:G	aggggctccc	A>G	ccatcccagg	78.45	5.82	105.14	7.91	5.54	10^{-6}	A			
		rs1603624843:T	ttggttttat	A>T	ctttccctga	1.96	0.21	5.93	0.52	16.02	10^{-6}	A			
		rs1603624844:G	atttggtttt	A>G	ttctttccct	1.96	0.21	5.37	0.55	13.59	10^{-6}	A			
		rs1603624844:T	atttggtttt	A>T	ttctttccct	1.96	0.21	8.59	0.76	21.26	10^{-6}	A			
		rs1648309128:C	ctcctcctcc	T>C	tgctcctcct	66.34	4.71	81.40	6.95	3.68	10^{-3}	B			
		rs180908396:C	ggaaagaaat	T>C	agaattctgc	11.76	1.11	28.00	2.08	14.43	10^{-6}	A			
		rs2061384063:G	gagctcctcc	A>G	agtgaccctt	28.46	2.14	32.14	2.99	2.04	0.05	D			
		rs2075529520:G	ttgatgtact	A>G	gcggcaactc	1.54	0.15	3.90	0.50	11.51	10^{-6}	A			
		rs2075624580:A	ttcgaggaac	T>A	aatcggcatt	3.85	0.28	5.80	0.49	7.33	10^{-6}	A			
		rs2075793995:G	gctgcgtaca	A>G	gttctcaggc	11.17	1.23	16.24	1.91	4.64	10^{-3}	B			
		rs2092362768:G	cgtgccatct	A>G	ctgagtgtga	16.69	1.34	19.35	1.43	2.71	10^{-2}	C			
		rs2093791242:G	ccgtattgtt	A>G	tttctcttgt	6.80	0.58	12.62	1.11	10.13	10^{-6}	A			
		rs2093822397:G	tttttttttcc	A>G	agttaatttt	22.01	1.50	26.49	1.89	3.76	10^{-3}	B			
		rs2093834615:C	ttcttactaa	T>C	ccctgtatcc	3.02	0.28	6.01	0.47	11.32	10^{-6}	A			
		rs2093834795:G	tttcttacta	A>G	cccctgtatc	3.02	0.28	4.24	0.37	5.32	10^{-6}	A			
		rs2093853696:G	caggaaagaa	A>G	aaagaattct	11.76	1.11	14.27	1.67	2.58	10^{-2}	C			
		rs2093949278:C	cagtttatca	T>C	atattttttt	8.08	0.51	9.70	0.91	3.24	10^{-2}	C			
		rs2093955059:G	atgaaattgt	T>G	tgtaaataaa	7.71	0.64	8.80	0.83	2.10	0.05	D			
		rs2093965800:G	gggagttttt	A>G	ctcctgggaa	5.47	0.61	9.24	0.90	7.06	10^{-6}	A			
		rs2094800432:A	cacaacagtt	T>A	cagattcacg	10.86	0.97	16.43	0.99	7.69	10^{-6}	A			
		rs2094875996:T	gcgattatga	A>T	agaaagacag	12.57	1.00	19.29	1.21	8.45	10^{-6}	A			
		rs2096819871:C	actgatatat	A>C	cccttctact	1.09	0.14	4.32	0.42	17.50	10^{-6}	A			
		rs2096819926:C	tactgactga	T>C	atttcccctt	1.09	0.14	2.45	0.29	9.49	10^{-6}	A			
		rs2097633101:A	ttctctttta	G>A	tgtaatgttg	6.16	0.48	7.04	0.46	2.63	10^{-2}	C			
		rs2097640148:G	tagggtcgaa	A>G	gggaaaccaa	8.46	0.76	13.44	1.17	7.37	10^{-6}	A			
		rs2097642577:C	cctcctcttc	T>C	ctcctcctcc	20.16	1.75	66.34	4.71	21.26	10^{-6}	A			
		rs2097660768:C	cctgatttgg	T>C	tttttctttt	1.96	0.21	2.78	0.34	4.26	10^{-3}	B			
		rs2097671747:G	gttggtgtta	A>G	cggagaagag	12.04	0.88	21.14	1.71	10.36	10^{-6}	A			
		rs2097672666:G	atttctttgc	T>G	cccccccacc	56.65	3.73	64.22	4.89	2.49	0.05	D			
		rs2097680912:C	taccactcta	T>C	ggcgctctgt	18.05	1.54	26.77	2.12	6.78	10^{-6}	A			
		rs2097680975:G	ctaggcgctc	T>G	tggccgcctc	15.33	1.37	18.05	1.57	2.62	10^{-2}	C			
		rs2097681003:T	tctctaggcg	C>T	tcttggccgc	15.33	1.37	17.71	1.51	2.33	0.05	D			
		rs369858906:C	tcctcctcct	T>C	gctcctcctc	66.34	4.71	81.40	6.95	3.68	10^{-3}	B			
		rs369858906:G	tcctcctcct	T>G	gctcctcctc	66.34	4.71	81.40	6.95	3.68	10^{-3}	B			

Table S3. Cont.

Human Gene		Candidate SNP marker				K_D, nM, *in silico*				Significance				Effect of changes in human gene expression on the development of primary open-angle glaucoma (POAG, ☼: "▼" aggravation, "▲" alleviation) [Reference]	☼ ▲ ▼
#	*NCBI Gene Symbol* (NCBI Gene ID)	dbSNP ID:min [437]	5′ flank, 10 bp	WT →min	3 flank, 10 bp	WT MEAN	± SEM	min MEAN	± SEM	Z	p	ϱ	Δ		
		rs374584569:G	tcctcctcct	C>G	gctgctcctc	66.34	4.71	81.40	6.95	3.68	10^{-3}	B			
		rs529307709:T	attatgaaga	C>T	aagacagggc	12.57	1.00	15.59	1.12	4.01	10^{-2}	B			
		rs532948788:G	gcatgtgaat	A>G	tagagtattt	9.98	0.79	11.14	0.87	1.98	0.05	D			
		rs535885747:G	tttgcagcct	A>G	catcacgtgt	2.43	0.32	4.07	0.46	5.93	10^{-6}	A			
		rs561026084:G	gggctcccac	A>G	atcccaggaa	78.45	5.82	105.14	7.91	5.54	10^{-6}	A			
		rs569404813:C	atcttttcat	T>C	caacgtttgt	11.01	0.91	16.04	1.51	6.00	10^{-6}	A			
		rs745753021:C	ttccagttta	T>C	aggatatttt	8.08	0.51	12.24	0.84	8.94	10^{-6}	A			
		rs74796261:A	acttttttttt	T>A	atttctggca	1.87	0.24	2.39	0.30	2.76	10^{-2}	C			
		rs74796261:C	acttttttttt	T>C	atttctggca	1.87	0.24	2.41	0.28	2.92	10^{-2}	C			
		rs766727238:C	ctcctcctcc	T>C	tgctgctcct	66.34	4.71	76.17	5.79	2.66	10^{-2}	C			
		rs772627542:G	gaaagaaatt	T>G	gaattctgca	11.76	1.11	28.00	2.08	14.43	10^{-6}	A			
		rs915299303:C	gagcgtgcca	T>C	ctactgagtg	16.69	1.34	19.35	1.43	2.71	10^{-2}	C	↓	within human disease models using neuron-specific Tcf4-knockdown adult mice (because of Tcf4-knockout animals are perinatally lethal) [387]: higher risks of both hyperexcitability and increased dendritic complexity of neurons contributing schizophrenia, which is comorbid to POAG as age-related neurodegenerative diseases [388]	
		rs918317881:C	ccttctcttt	A>C	cggccgcagc	2.54	0.33	12.77	1.32	19.37	10^{-6}	A			
		rs920377605:C	ctgactgata	T>C	ttccccttct	1.09	0.14	3.13	0.35	12.62	10^{-6}	A			
		rs928878162:G	ttttccctgt	A>G	gtgtgtttgt	1.35	0.19	3.85	0.52	10.66	10^{-6}	A			
		rs929716234:C	gctccttctc	T>C	ccgcggccgc	2.54	0.33	4.08	0.53	5.13	10^{-6}	A			
		rs929716234:G	gctccttctc	T>G	ccgcggccgc	2.54	0.33	3.55	0.39	3.89	10^{-3}	B			
		rs937678681:T	tttaaatttt	G>T	aaatgaaatt	7.71	0.64	9.88	0.84	4.18	10^{-3}	B			
		rs975736648:G	tgcattatcc	A>G	cagggaaggt	7.70	0.92	9.56	1.22	2.48	0.05	D			
		rs984610689:C	tttgatgtac	T>C	ggcggcaact	1.54	0.15	2.97	0.35	8.59	10^{-6}	A			
		rs990288118:G	aaactcgaat	A>G	tgaggggatg	8.63	0.59	12.31	1.05	6.51	10^{-6}	A			
		rs9959527:A	ttatgaagac	G>A	agacagggcg	12.57	1.00	18.22	1.36	6.79	10^{-6}	A			
132	*TCF4* (6925)	rs9959527:C	ttatgaagac	G>C	agacagggcg	12.57	1.00	20.09	1.69	8.09	10^{-6}	A			▼
		rs1009104646:T	tattttttttc	C>T	ggaatgaagg	8.08	0.51	5.03	0.44	8.84	10^{-6}	A			
		rs1009404140:A	cagttggagt	G>A	tatgaagacg	12.57	1.00	7.65	0.86	7.22	10^{-6}	A			
		rs1009776532:A	gatcccttgt	C>A	tcacattcca	23.10	1.72	12.11	1.34	9.68	10^{-6}	A			
		rs1024158287:T	actgaagtta	A>T	cctcgggtga	21.66	1.40	16.96	1.39	4.68	10^{-3}	B			
		rs1026600165:T	cattcctttt	C>T	cacttgtgca	15.43	1.25	11.69	0.78	5.29	10^{-6}	A			
		rs1047403290:A	tagtgtgtta	G>A	ttgcatgtga	9.98	0.79	7.04	0.60	6.03	10^{-6}	A			
		rs1171445977:A	tgtttctctt	T>A	atctgtaatg	6.16	0.48	2.49	0.25	14.25	10^{-6}	A			
		rs1172855847:A	actctttgat	G>A	catgggcggc	1.54	0.15	1.33	0.14	2.03	0.05	D			
		rs1174397391:A	ccactagata	C>A	gtgtgcatta	7.70	0.92	4.97	0.46	5.80	10^{-6}	A			
		rs1174397391:T	ccactagata	C>T	gtgtgcatta	7.70	0.92	4.90	0.45	5.95	10^{-6}	A			
		rs1177248129:C	gatcttttca	T>C	ccaacgtttg	11.01	0.91	8.85	0.90	3.32	10^{-3}	B			
		rs1186666252:A	agtaaaacat	G>A	taaatgtaca	17.64	1.15	6.04	0.53	19.55	10^{-6}	A	↑	according to a cohort-based biomedical study using postmortem donor corneas obtained from the eye bank of Transplant Services [389]: higher risks of Fuchs' endothelial corneal dystrophy comorbid to POAG [124]	
		rs1187473268:G	agctccttct	C>G	gccgcggccg	2.54	0.33	2.13	0.21	2.19	0.05	D			
		rs1198150380:T	gtggggaata	C>T	tttctccgtt	21.66	1.40	19.24	1.89	2.02	0.05	D			
		rs1200792686:C	catgccctcc	T>C	ctccccttct	28.08	2.14	25.07	1.95	2.09	0.05	D			
		rs1204138797:T	ctctgtttac	C>T	ctctctaggc	15.33	1.37	3.70	0.39	20.44	10^{-6}	A			
		rs1208207231:A	ctgaacttgg	G>A	attggtggaa	23.10	1.72	17.11	2.11	4.18	10^{-3}	B			
		rs1224463683:T	agatggccat	C>T	ttgtccttta	28.46	2.14	16.51	1.22	10.35	10^{-6}	A			
		rs1233945994:A	ccagtgggat	G>A	ttggagctcc	28.46	2.14	7.72	0.67	22.68	10^{-6}	A			
		rs1234292871:T	tttttttttt	C>T	gaagttaatt	21.66	1.40	11.49	0.78	13.56	10^{-6}	A			
		rs1242325337:T	tcctcctcct	C>T	cctccttctc	66.34	4.71	46.23	3.73	6.72	10^{-6}	A			
		rs1245089781:A	caagactatt	T>A	cttttccctt	2.92	0.24	2.45	0.22	2.86	10^{-2}	C			

Table S3. Cont.

Human Gene		Candidate SNP marker				KD, nM, *in silico*				Significance				Effect of changes in human gene expression on the development of primary open-angle glaucoma (POAG, ☼: "▼" aggravation, "▲" alleviation) [Reference]	☼ ▲ ▼
#	*NCBI Gene Symbol* (NCBI Gene ID)	dbSNP ID:min [437]	5′ flank, 10 bp	WT →min	3 flank, 10 bp	WT		min		Z	p	ϱ	Δ		
						MEAN±	SEM	MEAN±	SEM						
132	*TCF4* (6925)	rs1250432831:A	ctttgctttt	T>A	tccattcgtt	24.75	1.68	20.18	1.52	4.03	10^{-3}	B	↑	according to a cohort-based biomedical study using postmortem donor corneas obtained from the eye bank of Transplant Services [389]: higher risks of Fuchs' endothelial corneal dystrophy comorbid to POAG [124]	▼
		rs1256410294:T	tttgtaaagc	A>T	tgtaagatgc	6.49	0.65	4.70	0.37	5.11	10^{-6}	A			
		rs1267308051:C	cccctccccg	G>C	ctccgtcccc	67.50	4.23	59.51	4.41	2.60	10^{-2}	C			
		rs1279579999:A	gcggcgggct	G>A	cccacagtgc	78.45	5.82	39.40	3.48	11.94	10^{-6}	A			
		rs1303281797:T	tttgcagccc	C>T	aggcaggcag	24.75	1.68	21.56	1.47	2.88	10^{-2}	C			
		rs1307006497:T	tggtgttaag	A>T	gagaagagag	12.04	0.88	10.17	1.09	2.61	10^{-2}	C			
		rs1311794739:T	agtttgtttg	G>T	cagttcatgg	11.76	1.11	7.38	0.70	6.97	10^{-6}	A			
		rs1312371300:A	ggccatcaca	G>A	cctttacaga	28.46	2.14	25.22	1.72	2.38	0.05	D			
		rs1312963766:T	cccctttctg	C>T	agctttgcag	24.75	1.68	17.99	0.99	7.30	10^{-6}	A			
		rs1313572134:T	tcctcctcct	C>T	ccttctcctc	66.34	4.71	46.15	3.74	6.74	10^{-6}	A			
		rs1313902034:A	cctcccctcc	C>A	tccctccgtc	67.50	4.23	51.85	3.72	5.54	10^{-6}	A			
		rs1315354083:T	gcactttctc	C>T	ttctgcttgt	24.75	1.68	15.96	1.43	7.82	10^{-6}	A			
		rs1317032630:T	tccccttctt	C>T	cttgtcccac	28.08	2.14	19.47	1.51	6.75	10^{-6}	A			
		rs1320937167:A	ttggtggaat	C>A	tgctgggtct	23.10	1.72	10.83	0.87	13.80	10^{-6}	A			
		rs1320937167:T	ttggtggaat	C>T	tgctgggtct	23.10	1.72	17.87	1.31	4.91	10^{-6}	A			
		rs1321614447:A	taaattttgt	G>A	atgaaattgt	7.71	0.64	5.48	0.66	4.65	10^{-3}	B			
		rs1322443236:A	tgagtttggt	G>A	ttaatgccag	6.49	0.65	3.21	0.33	9.79	10^{-6}	A			
		rs1349504648:T	catagtaaaa	C>T	atttaaatgt	17.64	1.15	7.92	0.72	14.30	10^{-6}	A			
		rs1360429849:A	caattagggt	C>A	aagagggaaa	8.46	0.76	6.14	0.56	5.00	10^{-6}	A			
		rs1360429849:T	caattagggt	C>T	aagagggaaa	8.46	0.76	7.03	0.63	2.90	10^{-2}	C			
		rs1373217052:A	gctttttaat	C>A	tcctgccact	5.92	0.41	3.01	0.23	13.21	10^{-6}	A			
		rs1377201600:A	gtttaaattt	T>A	aaaatgaaat	7.71	0.64	6.23	0.46	3.83	10^{-3}	B			
		rs1382040032:T	aattagggtc	G>T	agagggaaac	8.46	0.76	4.81	0.48	8.38	10^{-6}	A			
		rs1386256431:T	gaataaattt	C>T	gatgtaaact	8.63	0.59	7.67	0.64	2.19	0.05	D			
		rs1389806405:T	ccccttccct	C>T	tttccccctc	67.50	4.23	60.91	4.36	2.16	0.05	D			
		rs1390355504:G	ccagtttatc	A>G	gatatttttt	8.08	0.51	7.18	0.65	2.16	0.05	D			
		rs1418103880:A	atgcaggaat	G>A	tcagcctggc	28.46	2.14	16.37	1.40	9.70	10^{-6}	A			
		rs1433748221:T	catggtaaca	C>T	tccagctttg	15.43	1.25	10.51	0.86	6.67	10^{-6}	A			
		rs1436263382:T	ctgtttacca	C>T	ctctaggcgc	18.80	1.70	14.94	1.26	3.72	10^{-3}	B			
		rs1441335728:A	gtgccgtatt	G>A	cagtttctct	6.80	0.58	4.33	0.38	7.42	10^{-6}	A			
		rs1443729479:A	ttaatcaaat	T>A	ccactcgctt	5.92	0.41	5.30	0.39	2.18	0.05	D			
		rs1446172153:G	attttttttt	T>G	aactgaagtt	21.66	1.40	16.65	1.12	5.63	10^{-6}	A			
		rs1447072809:A	gggtctattg	G>A	cttgtcctgc	23.10	1.72	15.35	1.24	7.44	10^{-6}	A			
		rs1456052767:T	acagtttatt	C>T	ttcacgccac	10.86	0.97	4.41	0.32	15.71	10^{-6}	A			
		rs1462878717:T	atttgtaaag	C>T	gtgtaagatg	6.49	0.65	3.80	0.35	7.88	10^{-6}	A			
		rs1463601946:G	agttaatttt	T>G	ggtgaaactg	21.66	1.40	18.94	1.25	2.90	10^{-2}	C			
		rs1465539351:A	gcgcctcggc	G>A	tgcaattccg	67.50	4.23	45.72	4.48	6.70	10^{-6}	A			
		rs1555710523:T	atcaacgagg	C>T	gggtccgtga	35.86	2.45	31.76	2.16	2.52	10^{-2}	C			
		rs1555710544:T	tgacatcaac	G>T	ctgcgggtcc	35.86	2.45	17.82	1.48	13.00	10^{-6}	A			
		rs1555710943:T	gcgtgagaag	G>T	cagaaggcag	35.86	2.45	29.45	2.21	3.87	10^{-3}	B			
		rs1555710958:T	agagcgtgag	A>T	gagcagaagg	35.86	2.45	24.91	1.94	7.03	10^{-6}	A			
		rs1555710973:T	ggcagagcgt	G>T	ccagagcaga	35.86	2.45	14.61	1.19	16.91	10^{-6}	A			
		rs1555710988:T	gcagaaggca	G>T	ctgacaccag	35.86	2.45	24.73	1.86	7.32	10^{-6}	A			
		rs1555796915:A	gcaggaatgt	T>A	agcctggcta	28.46	2.14	17.36	1.50	8.63	10^{-6}	A			

Table S3. Cont.

Human Gene		Candidate SNP marker				K$_D$, nM, *in silico*				Significance				Effect of changes in human gene expression on the development of primary open-angle glaucoma (POAG, ☼: "▼" aggravation, "▲" alleviation) [Reference]	☼ ▲ ▼
#	*NCBI Gene Symbol* (NCBI Gene ID)	dbSNP ID:min [437]	5′ flank, 10 bp	WT →min	3 flank, 10 bp	WT MEAN	± SEM	min MEAN	± SEM	Z	p	ρ	Δ		
		rs1555796988:T	tggctatgca	G>T	atgaatcagc	28.46	2.14	13.77	1.08	13.37	10^{-6}	A			
		rs1555797121:T	tgggatgaat	C>T	agctcctcca	28.46	2.14	23.82	1.63	3.50	10^{-3}	B			
		rs1598841692:A	ctcccacagt	G>A	ccaggaaggg	78.45	5.82	27.16	2.19	19.36	10^{-6}	A			
		rs1603447075:A	ggtgagaagt	G>A	tccaaacaca	21.66	1.40	8.69	0.85	15.63	10^{-6}	A			
		rs1603448504:A	tttttttcca	G>A	aatgaaggat	8.08	0.51	6.83	0.54	3.34	10^{-3}	B			
		rs1603624692:T	tttagtttta	A>T	gttgtttctc	6.16	0.48	5.21	0.44	2.91	10^{-2}	C			
		rs1603624760:G	ttgtaaagca	A>G	gtaagatgca	6.49	0.65	5.07	0.43	3.77	10^{-3}	B			
		rs1603624845:T	ccctgatttg	G>T	ttttttttctt	1.96	0.21	1.66	0.17	2.22	0.05	D			
		rs1603624929:A	ccccggagaa	G>A	tcccctcccc	67.50	4.23	39.85	2.75	11.30	10^{-6}	A			
		rs1603624931:A	ccctcccctc	C>A	ttccctccgt	67.50	4.23	61.12	4.31	2.11	10^{-6}	A			
		rs1603624932:A	cccctcccct	C>A	cttccctccg	67.50	4.23	46.74	3.68	7.31	10^{-6}	A			
		rs17522826:T	tttatcatag	C>T	tttttttcca	8.08	0.51	6.73	0.41	4.20	10^{-3}	B			
		rs1865824503:T	tcatcctagg	C>T	tcctcccggt	28.08	2.14	24.45	1.79	2.62	10^{-2}	C			
		rs187598991:A	tgggtctatt	G>A	ccttgtcctg	23.10	1.72	4.74	0.51	24.09	10^{-6}	A			
		rs2047109965:T	tctgcgggtc	C>T	gcccgagagc	35.86	2.45	30.37	2.05	3.46	10^{-3}	B			
		rs2061377351:T	cctggctatg	C>T	ggatgaatca	28.46	2.14	4.82	0.60	24.44	10^{-6}	A			
		rs2061383201:A	tcctccagtg	G>A	acccttggag	28.46	2.14	21.98	1.64	4.87	10^{-3}	B			
		rs2068692993:T	tcacagcgga	C>T	agccacaggc	28.08	2.14	19.67	1.65	6.30	10^{-6}	A			
		rs2075792119:A	tgcgtacaat	G>A	tctcaggcag	11.17	1.23	5.83	0.60	8.61	10^{-6}	A			
		rs2082428881:A	tgtgaatagt	G>A	agtattttgc	9.98	0.79	4.36	0.42	13.37	10^{-6}	A			
		rs2082430879:T	gagtattttg	C>T	tggcctattt	9.98	0.79	5.88	0.43	9.78	10^{-6}	A			
		rs2092357705:T	tttgagtgaa	C>T	ctccttttca	16.69	1.34	11.20	1.08	6.35	10^{-6}	A			
132	*TCF4* (6925)	rs2092358427:A	ttcagtttga	G>A	cttttctcct	16.69	1.34	13.06	0.99	4.45	10^{-3}	B	↑	according to a cohort-based biomedical study using postmortem donor corneas obtained from the eye bank of Transplant Services [389]: higher risks of Fuchs' endothelial corneal dystrophy comorbid to POAG [124]	▼
		rs2093791973:C	ttgtgccgta	T>C	atcagtttct	6.80	0.58	5.52	0.52	3.28	10^{-2}	C			
		rs2093823399:A	gttaattttt	T>A	gtgaaactga	21.66	1.40	9.78	0.78	15.45	10^{-6}	A			
		rs2093825728:T	cgtttgtggg	G>T	cctcctttct	21.66	1.40	15.37	1.93	4.85	10^{-6}	A			
		rs2093852960:T	aagaaattta	C>T	attctgcagg	11.76	1.11	4.08	0.42	15.09	10^{-6}	A			
		rs2093853896:T	gcaggaaaga	A>T	gaaagaattc	11.76	1.11	6.53	0.49	9.74	10^{-6}	A			
		rs2093951715:A	gctaagaata	G>A	ggagcagttt	8.08	0.51	6.92	0.55	3.05	10^{-2}	C			
		rs2093969492:A	tttcccaaat	G>A	caaaagccct	5.47	0.61	2.78	0.29	8.88	10^{-6}	A			
		rs2093970767:A	cttgggttca	G>A	tggaatctga	23.10	1.72	19.80	1.27	3.14	10^{-2}	C			
		rs2093971931:A	gtctattggt	G>A	tgtcctgctg	23.10	1.72	12.06	0.90	12.32	10^{-6}	A			
		rs2094800940:G	tcacgccaca	A>G	ggctggcaga	10.86	0.97	8.76	0.83	3.31	10^{-2}	C			
		rs2094803735:A	ctatttaaat	G>A	ccctttcaag	2.92	0.24	2.56	0.22	2.20	0.05	D			
		rs2094810346:A	gttagcctaa	G>A	atgtgatgag	12.79	0.97	8.89	0.81	6.10	10^{-6}	A			
		rs2094813455:T	actctttaaa	C>T	gggagtcttt	8.67	0.72	6.49	0.53	4.97	10^{-6}	A			
		rs2097640109:A	cgaaataaat	G>A	ccaattaggg	8.46	0.76	3.52	0.50	10.44	10^{-6}	A			
		rs2097640734:T	gcattatcca	C>T	agggaaggtg	7.70	0.92	6.37	0.67	2.37	0.05	D			
		rs2097641406:T	tttctccatt	C>T	gcttgttgca	24.75	1.68	17.72	1.23	6.89	10^{-6}	A			
		rs2097656807:A	ttctgattgc	G>A	gggactggat	16.75	1.32	12.02	1.20	5.20	10^{-6}	A			
		rs2097660720:A	tgatttggtt	T>A	ttttctttcc	1.96	0.21	1.14	0.14	6.53	10^{-6}	A			
		rs2097671480:T	tctgtagtgg	C>T	gtgtctgcgg	11.66	1.31	9.72	1.08	2.31	0.05	D			
		rs2097671763:T	gaagagagtt	G>T	ccctccccgg	67.50	4.23	25.42	1.88	20.16	10^{-6}	A			
		rs2097672328:T	cggcgtgttt	C>T	tccgtgcgcc	67.50	4.23	56.92	4.04	3.61	10^{-3}	B			
		rs2097672558:G	aacttgcaat	T>G	tttctttgct	56.65	3.73	41.46	2.83	6.58	10^{-6}	A			

Table S3. Cont.

Human Gene		Candidate SNP marker				K_D, nM, *in silico*				Significance				Effect of changes in human gene expression on the development of primary open-angle glaucoma (POAG, ☼: "▼" aggravation, "▲" alleviation) [Reference]	☼ ▲ ▼
#	*NCBI Gene Symbol* (NCBI Gene ID)	dbSNP ID:min [437]	5′ flank, 10 bp	WT →min	3 flank, 10 bp	WT MEAN±	WT SEM	min MEAN±	min SEM	Z	p	ϱ	Δ		
132	*TCF4* (6925)	rs2097672658:A	tttctttgct	G>A	ccccccaccc	56.65	3.73	27.58	2.20	13.91	10^{-6}	A	↑	according to a cohort-based biomedical study using postmortem donor corneas obtained from the eye bank of Transplant Services [389]: higher risks of Fuchs' endothelial corneal dystrophy comorbid to POAG [124]	▼
		rs2097674892:T	tgtaaactcg	A>T	aagtgagggg	8.63	0.59	2.56	0.27	19.14	10^{-6}	A			
		rs2097674907:T	gatgtaaact	C>T	agaagtgagg	8.63	0.59	7.31	1.08	2.05	0.05	D			
		rs2097680925:A	gtttaccact	C>A	ctaggcgctc	15.33	1.37	3.65	0.40	20.17	10^{-6}	A			
		rs2097680939:A	gctctgttta	C>A	cctctctagg	15.33	1.37	7.23	0.71	11.32	10^{-6}	A			
		rs2097680961:G	ggcgctctgt	T>G	ccgcctctct	15.33	1.37	9.57	1.03	6.71	10^{-6}	A			
		rs2097736051:T	ctgggttaca	C>T	ccaacagcat	15.43	1.25	7.95	0.65	11.49	10^{-6}	A			
		rs2097744198:T	ccgcctcggc	C>T	ggagccggct	78.45	5.82	58.45	4.98	5.21	10^{-6}	A			
		rs370306229:A	tgctgctgct	C>A	gctgctgctg	66.34	4.71	48.12	4.31	5.62	10^{-6}	A			
		rs372659948:A	tgctgctgct	G>A	gctgctgctg	66.34	4.71	33.51	3.13	11.63	10^{-6}	A			
		rs373584993:A	tgctgctgct	G>A	gctgctgctg	66.34	4.71	39.62	3.58	8.98	10^{-6}	A			
		rs374584569:T	tcctcctcct	C>T	gctgctcctc	66.34	4.71	39.65	2.85	10.19	10^{-6}	A			
		rs533937377:A	gtcctgctgg	G>A	catgatccct	23.10	1.72	17.14	1.57	5.06	10^{-6}	A			
		rs535081998:T	cctcctcctt	C>T	ctcctcctcc	66.34	4.71	52.54	4.02	4.47	10^{-3}	B			
		rs542686368:T	cccacagtgc	C>T	aggaaggggc	78.45	5.82	67.68	5.65	2.65	10^{-2}	C			
		rs559539653:A	aatcagcctg	G>A	ccagtgggat	28.46	2.14	21.89	1.91	4.55	10^{-3}	B			
		rs559539653:T	aatcagcctg	G>T	ccagtgggat	28.46	2.14	15.59	1.64	9.31	10^{-6}	A			
		rs747361615:A	gcctggctat	G>A	gggatgaatc	28.46	2.14	5.80	0.94	17.88	10^{-6}	A			
		rs747361615:T	gcctggctat	G>T	gggatgaatc	28.46	2.14	23.29	2.14	3.37	10^{-3}	B			
		rs758812309:T	ggctatgcag	G>T	tgaatcagcc	28.46	2.14	11.19	1.23	14.05	10^{-6}	A			
		rs761863292:A	cccagtcatt	G>A	ccccgcccct	18.05	1.54	12.14	1.03	6.60	10^{-6}	A			
		rs763961841:A	tgctgctgct	G>A	gctgctgctg	66.34	4.71	33.51	3.13	11.63	10^{-6}	A			
		rs766054008:T	ttagaattta	G>T	tgacatggtg	9.98	0.79	2.31	0.26	21.16	10^{-6}	A			
		rs768573909:A	acagtttttg	G>A	ccattggaat	14.30	1.01	12.37	0.94	2.78	10^{-2}	C			
		rs780717189:T	ggagctcctc	C>T	cagtgaccct	28.46	2.14	19.89	1.53	6.66	10^{-6}	A			
		rs879108660:T	ctagtataaa	C>T	gtatttgcag	2.43	0.32	1.48	0.16	5.74	10^{-6}	A			
		rs889748935:T	cttttttttt	A>T	tttctggcaa	1.87	0.24	1.24	0.13	4.99	10^{-6}	A			
		rs891956494:T	tttaaaccac	C>T	gtctttaact	12.79	0.97	10.86	0.78	3.13	10^{-2}	C			
		rs910058600:T	ccaagcccca	G>T	tgttaggaca	17.64	1.15	14.60	0.93	4.14	10^{-3}	B			
		rs914388067:A	tgctcaatat	G>A	agtgcttaat	3.23	0.35	1.56	0.16	9.69	10^{-6}	A			
		rs914950266:A	gctcctcctc	C>A	ctgctgctgc	66.34	4.71	58.08	4.48	2.53	0.05	D			
		rs915761935:T	aagacagggc	G>T	aagcagatag	12.57	1.00	10.89	0.89	2.51	0.05	D			
		rs916184577:T	cctgctgggt	C>T	tgatcccttg	23.10	1.72	12.52	1.67	8.03	10^{-6}	A			
		rs919867632:T	aaggggctcc	C>T	gccatcccag	78.45	5.82	19.81	2.08	21.44	10^{-6}	A			
		rs925057882:T	ttgcagcccc	C>T	ggcaggcagc	24.75	1.68	18.25	1.23	6.37	10^{-6}	A			
		rs945101301:T	cgatttgttt	G>T	ataaggagtt	14.30	1.01	8.15	0.67	10.35	10^{-6}	A			
		rs947386507:T	actctatggt	C>T	ctctgtttac	18.80	1.70	15.51	1.41	3.01	10^{-2}	C			
		rs967091397:A	taggcgctct	G>A	ggccgcctct	15.33	1.37	5.38	0.56	15.24	10^{-6}	A			
		rs974899376:A	tttcattaca	T>A	gtttgtgatc	11.01	0.91	6.85	0.55	8.19	10^{-6}	A			
		rs976470976:T	ctttgccatg	G>T	gactcttcca	15.43	1.25	13.84	0.94	2.06	0.05	D			
		rs988393350:G	tcctccttct	C>G	cctcctcctc	66.34	4.71	52.69	3.82	4.54	10^{-3}	B			
		rs988393350:T	tcctccttct	C>T	cctcctcctc	66.34	4.71	39.65	2.85	10.19	10^{-6}	A			
		rs991000077:A	agcatgttgt	G>A	agccaagccc	17.64	1.15	8.43	0.84	12.35	10^{-6}	A			
		rs993733471:A	attccttttc	G>A	acttgtgcag	15.43	1.25	10.69	0.71	7.00	10^{-6}	A			

Table S3. Cont.

#	Human Gene: NCBI Gene Symbol (NCBI Gene ID)	Candidate SNP marker: dbSNP ID:min [437]	5′ flank, 10 bp	WT →min	3 flank, 10 bp	K_D, nM, *in silico*: WT MEAN	±SEM	min MEAN	±SEM	Significance: Z	p	ϱ	Δ	Effect of changes in human gene expression on the development of primary open-angle glaucoma (POAG, ☼: "▼" aggravation, "▲" alleviation) [Reference]	☼ ▲ ▼
133	*TGFB2* (7042)	rs1021988847:C	agagaactta	T→C	aaatctcccc	2.53	0.26	6.40	0.62	13.04	10^{-6}	A	↓	within human POAG treatment models using mice subjected with the ocular eye drops containing astragaloside IV, which is a novel saponin isolated from the roots of Astragalus membranaceus as a promising medication against POAG [390]: the Tgfbr2 inhibition reduces intraocular pressure that can alleviate POAG	▲
		rs1571818459:C	gagagaactt	A→C	taaatctccc	2.53	0.26	12.27	1.09	23.05	10^{-6}	A			
		rs1571819869:A	gctccttctt	T→A	aaatatataa	1.50	0.12	1.88	0.18	3.57	10^{-3}	B			
		rs546214861:C	tctttaaata	T→C	ataaatttca	1.50	0.12	1.86	0.19	3.22	10^{-2}	C			
134	*TGFBR3* (7049)	rs1287034148:C	gcccgcgtgt	G→C	tgcccgtccc	40.71	3.89	60.23	4.41	6.51	10^{-6}	A	↓	within a cohort-based biomedical RNA-Seq analysis of human Schlemm's canal endothelial cells isolated from eight different postmortem human eyes with versus without glaucoma [391]: TGFBR3-deficit can be a biomedical molecular marker of POAG	▼
		rs1361073518:G	cgcccgcgtg	T→G	ctgcccgtcc	40.71	3.89	60.23	4.41	6.51	10^{-6}	A			
		rs1048759362:G	agtgccccac	C→G	gagatgagca	28.86	2.87	15.54	1.59	8.66	10^{-6}	A	↑	within a cohort-based biomedical cellular study using orbital fibroblasts isolated from specimens obtained intraoperatively from patients with versus without thyroid-eye disease, who underwent decompressive surgery [392]: TGFBR3 excess can prevent fibrosis, which is a complication of POAG [393]	▲
		rs1213707430:T	ccctcccctg	G→T	tggggaatcg	28.86	2.87	23.93	1.88	2.96	10^{-2}	C			
		rs1252009134:A	ccctggccat	G→A	aatcgcccct	28.86	2.87	11.64	1.12	13.12	10^{-6}	A			
		rs1261800739:A	gctgattgct	G→A	tccccccgcg	40.71	3.89	30.01	2.48	4.83	10^{-3}	B			
		rs1281694730:T	tcggcctgat	G→T	agtgcgccgg	54.81	5.21	45.12	4.95	2.68	10^{-2}	C			
		rs1316756347:T	tccccgcccg	C→T	cgtcctgccc	40.71	3.89	20.83	1.95	10.03	10^{-6}	A			
		rs1330623006:A	gggggtaatc	G→A	gtcggcctga	54.81	5.21	36.10	2.89	6.72	10^{-6}	A			
		rs1330623006:T	gggggtaatc	G→T	gtcggcctga	40.71	3.89	14.75	1.43	14.91	10^{-6}	A			
		rs1678199271:T	tgcaaggagg	G→T	agtttgaaaa	7.65	0.67	5.49	0.61	4.71	10^{-3}	B			
		rs1678200422:A	ttttcttatt	G→A	tcttcattaa	7.65	0.67	3.88	0.38	10.40	10^{-6}	A			
		rs1679299906:T	cctgatgggg	G→T	cgccgggtcg	40.71	3.89	22.22	2.31	8.58	10^{-6}	A			
		rs1679302340:A	gtccccgccc	G→A	gcgtcctgcc	40.71	3.89	24.76	2.72	6.83	10^{-6}	A			
		rs1679302428:T	gcccgtcccc	G→T	tgtggcgtcc	40.71	3.89	35.56	3.29	2.03	0.05	D			
		rs1679304439:T	cccgcgcgct	G→T	gcgcccttcc	40.71	3.89	20.61	1.36	11.74	10^{-6}	A			
		rs1679851884:T	cacagtgccc	C→T	cgcgagatga	28.86	2.87	10.60	1.19	13.33	10^{-6}	A			
		rs1679852358:A	ctcgcgagat	G→A	gccatgtggt	28.86	2.87	18.75	1.48	6.80	10^{-6}	A			
		rs537116060:G	gatgggggta	A→G	cgggtcggcc	54.81	5.21	47.47	4.81	2.07	0.05	D			
		rs537116060:T	gatgggggta	A→T	cgggtcggcc	40.71	3.89	28.48	2.89	5.13	10^{-6}	A			
		rs905713144:T	gcgagatgag	C→T	atgtggttct	28.86	2.87	11.50	1.33	12.07	10^{-6}	A			
		rs929900640:A	gagcggcact	T→A	tcggggacgc	54.81	5.21	34.79	3.44	6.62	10^{-6}	A			
		rs947282172:G	tgggggtaat	C→G	ggtcggcctg	54.81	5.21	39.56	3.76	4.85	10^{-3}	B			
135	*TIMP1* (7076)	rs1379028848:G	accagaggta	A→G	gcagggcccg	23.97	2.23	49.65	3.58	12.39	10^{-6}	A	↓	within a cohort-based biomedical qPCR-analysis of specimens of Tenon capsule obtained intraoperatively from patients with versus without POAG [394]: TIMP1-deficit as a biomedical molecular marker of POAG	▼
		rs1053991841:G	tgagtaatgc	A→G	tccaggaagc	25.73	1.77	20.49	1.86	4.00	10^{-3}	B	↑	within human neuroprotection cellular models using mice BV2 microglial cell culture subjected with ursolic acid as a promising neuroprotector being intensively studied pharmaceutically now, that resulted in reliably detected anti-apoptotic neuroprotective changes, which were next reversed back by a TIMP1 neutralizing antibody (anti-TIMP1) [395]: TIMP1 excess has anti-apoptotic neuroprotective abilities that may slow the progression of POAG	▲
		rs1225338968:T	cccagagaga	C→T	accagaggta	23.97	2.23	16.81	2.17	4.46	10^{-3}	B			
		rs1226680303:A	cccccgccat	G→A	gagagtgtct	34.21	2.93	19.04	1.51	10.02	10^{-6}	A			
		rs1304469082:C	agagacacca	G→C	aggtaagcag	23.97	2.23	17.70	1.56	4.74	10^{-3}	B			
		rs1319834840:A	gagtgtctgc	G→A	gatacttcca	34.21	2.93	20.91	2.60	6.52	10^{-6}	A			
		rs1360628820:A	ctgcggatac	T→A	tccacaggtc	34.21	2.93	14.04	1.52	12.90	10^{-6}	A			
		rs1413685629:T	cggatacttc	C→T	acaggtccca	34.21	2.93	7.56	0.77	22.73	10^{-6}	A			
		rs200254858:A	ccgccatgga	G→A	agtgtctgcg	34.21	2.93	27.16	2.20	3.91	10^{-3}	B			
		rs2057801606:A	gtgggtggat	G→A	agtaatgcat	25.73	1.77	6.75	0.60	23.70	10^{-6}	A			
		rs2057801989:T	ctagcgtgga	C→T	atttatcctc	5.01	0.43	3.88	0.34	4.11	10^{-3}	B			
		rs2057802299:A	cgccgcagat	C→A	cagcgcccag	23.97	2.23	15.08	1.61	6.55	10^{-6}	A			
		rs2057802465:A	agacaccaga	G→A	gtaagcaggg	23.97	2.23	18.40	1.58	4.18	10^{-3}	B			
		rs754631765:T	gtcccacaac	C→T	gcagcgagga	34.21	2.93	26.20	2.10	4.54	10^{-3}	B			
		rs755568993:T	agagtgtctg	C→T	ggatacttcc	34.21	2.93	27.96	2.51	3.25	10^{-2}	C			

Table S3. Cont.

#	Human Gene: *NCBI Gene Symbol* (NCBI Gene ID)	Candidate SNP marker: dbSNP ID:min [437]	5′ flank, 10 bp	WT →min	3 flank, 10 bp	K_D, nM, *in silico*: WT MEAN	± SEM	min MEAN	± SEM	Significance: Z	p	ϱ	Δ	Effect of changes in human gene expression on the development of primary open-angle glaucoma (POAG, ☼: "▼" aggravation, "▲" alleviation) [Reference]	☼ ▲ ▼
136	*TIMP2* (7077)	rs1180340247:G	actgcctccc	A→G	tcaaatcaat	8.76	0.91	10.29	1.16	2.09	0.05	D	↓	within human POAG model using rats, who were first subjected to artificial pathogenesis of POAG after which they were treated recombinant human decorin [396]: Timp2 downregulation along with both reduced intraocular pressure and retarded retinal ganglion cell loss that all can relieve POAG	▲
		rs1180340247:T	actgcctccc	A→T	tcaaatcaat	8.76	0.91	10.54	1.05	2.56	0.05	D			
		rs1192042887:G	catgatgtca	C→G	ctcacccctg	29.17	1.94	36.70	2.39	4.93	10^{-6}	A			
		rs1200100857:G	ctcacccctg	C→G	ctgtccctga	29.17	1.94	35.92	2.34	4.47	10^{-3}	B			
		rs1307704258:G	gcccctgtca	A→G	ttctggcttg	21.10	1.34	24.20	1.59	2.99	10^{-2}	C			
		rs1397143037:C	atgatgtcac	A→C	tcacccctgc	29.17	1.94	33.94	2.45	3.08	10^{-2}	C			
		rs1419889516:C	gcgcaataaa	A→C	aaatcctccg	5.44	0.50	11.07	1.41	9.07	10^{-6}	A			
		rs1599180816:C	gtcatattca	T→C	ttccaaataa	6.45	0.52	8.19	0.88	3.54	10^{-3}	B			
		rs2069709617:G	cttgggcccc	T→G	tgggcttctg	21.10	1.34	23.85	1.66	2.59	10^{-2}	C			
		rs2069710018:C	cctcccactg	T→C	atcaatgact	8.76	0.91	10.91	1.02	3.13	10^{-2}	C			
		rs2070123572:C	ccatgatgtc	A→C	cctcacccct	29.17	1.94	43.16	2.95	8.22	10^{-6}	A			
		rs2070314783:G	aaggtcatat	T→G	tatttccaaa	6.45	0.52	8.19	0.88	3.54	10^{-3}	B			
		rs2070314805:T	ataaggtcat	A→T	tctatttcca	6.45	0.52	8.19	0.88	3.54	10^{-3}	B			
		rs2070345127:T	cgcaataaaa	C→T	aatcctccga	5.44	0.50	6.60	0.56	3.09	10^{-2}	C			
		rs542453668:G	tccgagcgca	A→G	gcagaaaatc	5.44	0.50	9.69	1.28	7.20	10^{-6}	A			
		rs556975052:A	cccctgtcaa	C→A	tctggcttgg	21.10	1.34	23.34	1.66	2.11	0.05	D			
		rs987299827:C	cctgccatga	T→C	ctgacctcac	29.17	1.94	46.96	2.94	10.42	10^{-6}	A			
		rs115467730:T	cacagctcaa	C→T	tgccatgatg	29.17	1.94	19.02	1.83	7.32	10^{-6}	A	↑	according to comprehensive review [397]: overexpression of TIMP2 in optic nerve using a lentiviral vector carrying another TIMP2 gene copy is a promising gene therapy strategy for axonal regeneration being intensively studied pharmaceutically to retard the progression of POAG	
		rs1274496614:A	cctcacccct	G→A	gctgtccctg	29.17	1.94	23.26	1.91	4.29	10^{-3}	B			
		rs1406838517:A	atcctccgag	C→A	aggagcagaa	5.44	0.50	4.72	0.42	2.20	0.05	D			
		rs2069709476:A	agggattctt	G→A	gtcaacaaag	21.10	1.34	8.65	0.73	16.83	10^{-6}	A			
		rs2070124230:T	ctggctggct	C→T	acgcttgccc	29.17	1.94	24.49	2.21	3.12	10^{-2}	C			
		rs2070345217:T	ctccgagcgc	A→T	agcagaaaat	5.44	0.50	2.09	0.20	14.38	10^{-6}	A			
		rs2070345239:A	tcctccgagc	G→A	ggagcagaaa	5.44	0.50	4.43	0.41	3.13	10^{-2}	C			
		rs556975052:T	cccctgtcaa	C→T	tctggcttgg	21.10	1.34	7.82	0.78	16.75	10^{-6}	A			
		rs565793754:A	gctcacagct	G→A	cccctggct	29.17	1.94	25.88	2.31	2.15	0.05	D			
		rs749406272:T	gcttgggccc	C→T	ctgggcttct	21.10	1.34	19.10	1.21	2.22	0.05	D			
		rs879479065:G	caaagaaggg	A→G	gcccctgtca	21.10	1.34	18.84	1.25	2.47	0.05	D			
		rs931491645:T	gccatgatgt	C→T	acctcacccc	29.17	1.94	11.45	0.98	17.23	10^{-6}	A			
		rs950603314:A	ctgccatgat	G→A	tgacctcacc	29.17	1.94	13.43	0.94	16.06	10^{-6}	A			
		rs975220030:G	aacaaatact	C→G	cttgatagat	6.45	0.52	5.64	0.53	2.17	0.05	D			
137	*TIMP3* (7078)	rs1450847914:G	ccagcgctat	A→G	tcactcggcc	5.16	0.63	14.81	1.53	13.19	10^{-6}	A	↓	within a cohort-based biomedical study [283]: TIMP3 deficiency is a biomedical molecular marker of POAG	▼
		rs2046583724:C	cccagcgcta	T→C	atcactcggc	5.16	0.63	14.19	1.43	12.80	10^{-6}	A			
		rs976489437:G	gcccagcgct	A→G	tatcactcgg	5.16	0.63	14.49	1.58	12.63	10^{-6}	A			
		rs976489437:T	gcccagcgct	A→T	tatcactcgg	5.16	0.63	10.31	0.86	9.39	10^{-6}	A			
		rs2046583503:T	gggcccagcg	C→T	tatatcactc	5.16	0.63	3.82	0.42	3.70	10^{-3}	B	↑	within a cohort-based pharmaceutical tissue study using trabecular meshwork, ciliary muscle, and scleral fibroblast separated from cadaver eyes within 6 hours postmortem [398]: both bimatoprost and latanoprost are commonly accepted eye medications against POAG, which can simultaneously both upregulate TIMP3 and reduce the increased intraocular pressure	▲
		rs2046583936:A	cagcgctata	T→A	cactcggccg	5.16	0.63	3.73	0.44	3.83	10^{-3}	B			
		rs765139767:A	ggggcccagc	G→A	ctatatcact	5.16	0.63	3.38	0.36	5.24	10^{-6}	A			
138	*TIMP4* (7079)	rs1408218046:C	ccacctcata	A→C	ggcccttagg	10.19	0.93	12.16	1.15	2.69	10^{-2}	C	↓	within a cohort-based biomedical study [399]: sulfur mustard downregulates TIMP4 along with systemic eye damages aggravating POAG	▼

Table S3. Cont.

Human Gene		Candidate SNP marker				K_D, nM, *in silico*				Significance				Effect of changes in human gene expression on the development of primary open-angle glaucoma (POAG, ☼: "▼" aggravation, "▲" alleviation) [Reference]	☼ ▲ ▼
#	*NCBI Gene Symbol* (NCBI Gene ID)	dbSNP ID:min [437]	5′ flank, 10 bp	WT →min	3 flank, 10 bp	WT MEAN	± SEM	min MEAN	± SEM	Z	p	ϱ	Δ		
139	*TLR2* (7097)	rs1021057878:C	cctccagagc	T→C	ataataaata	2.51	0.17	2.93	0.25	2.85	10^{-2}	C	↓	within human disease models using Tlr2-knockout mice [400]: susceptibility to experimental autoimmune diseases, while POAG is biomedically well-known risk-factor of autoimmune diseases [194]	▼
		rs1169024514:C	cagagctata	A→C	taaatacatt	2.51	0.17	2.93	0.25	2.85	10^{-2}	C			
		rs1189955365:C	aataaataca	T→C	ttctgttctt	2.51	0.17	2.93	0.25	2.85	10^{-2}	C			
		rs1194710459:C	tcccttttgc	T→C	tacttcctag	20.90	1.79	26.57	1.95	4.25	10^{-3}	B			
		rs1447693055:C	agagctataa	T→C	aaatacattt	2.51	0.17	2.93	0.25	2.85	10^{-2}	C			
		rs1447693055:G	agagctataa	T→G	aaatacattt	2.51	0.17	2.93	0.25	2.85	10^{-2}	C			
		rs1471609683:G	cccttttgct	T→G	acttcctagt	20.90	1.79	27.24	2.74	4.01	10^{-3}	B			
		rs1578950658:C	cccgcacccc	A→C	gaaggagggg	62.21	4.67	104.40	8.69	9.24	10^{-6}	A			
		rs1735505267:C	gcttacttcc	T→C	agtcccgggc	20.90	1.79	27.70	2.18	4.85	10^{-3}	B			
		rs1736879696:G	cccagcctcc	A→G	gagctataat	2.51	0.17	2.80	0.17	2.44	0.05	D			
		rs1737007146:T	tcttttattt	G→T	taggttgaag	4.71	0.41	6.32	0.52	4.91	10^{-6}	A			
		rs757468634:C	cttttatttg	T→C	aggttgaagc	4.71	0.41	7.32	0.61	7.33	10^{-6}	A			
		rs1034089979:A	gcgcagcggc	G→A	cacgtgcccc	62.21	4.67	31.32	3.87	9.49	10^{-6}	A	↑	within human eye disease models using mice subjected with intravitreal injection of a Gram-positive spherically shaped bacterium Staphylococcus aureus [401]: Tlr2-upregulation in retinal Muller glia contributing to retinal innate defense via production of inflammatory mediators and antimicrobial peptides opposing infectious endophthalmitis following POAG filtration surgery, especially during the coronavirus disease (COVID-19) pandemic period [402]	▲
		rs1034089979:T	gcgcagcggc	G→T	cacgtgcccc	62.21	4.67	50.85	4.04	3.69	10^{-3}	B			
		rs1298299020:A	cagctctctt	C→A	ttcgacccag	51.47	3.67	11.99	0.96	27.13	10^{-6}	A			
		rs1298299020:T	cagctctctt	C→T	ttcgacccag	51.47	3.67	38.18	2.87	5.77	10^{-6}	A			
		rs1326655755:A	aggggcggtt	C→A	ccgcacccca	62.21	4.67	52.88	7.49	2.03	0.05	D			
		rs1365451972:T	gcggactttc	C→T	cttttgctta	62.21	4.67	34.35	2.46	11.46	10^{-6}	A			
		rs1393402068:T	accccagaag	G→T	agggggcgcc	62.21	4.67	18.69	1.50	21.92	10^{-6}	A			
		rs1404758587:A	cacggggcag	C→A	tgtcggggca	51.47	3.67	41.84	3.36	3.86	10^{-3}	B			
		rs1470210555:T	cgcggacttt	C→T	ccttttgctt	62.21	4.67	42.48	3.07	7.32	10^{-6}	A			
		rs1560731404:T	cggactttcc	C→T	ttttgcttac	20.90	1.79	18.13	1.29	2.55	0.05	D			
		rs1735501306:A	ggttcccgca	C→A	cccagaagga	62.21	4.67	49.39	3.53	4.45	10^{-3}	B			
		rs1735501502:C	ccgcacccca	G→C	aaggaggggg	62.21	4.67	37.79	2.97	9.17	10^{-6}	A			
		rs1735504441:C	ggactttccc	T→C	tttgcttact	20.90	1.79	17.02	1.55	3.28	10^{-2}	C			
		rs1735504577:C	gactttccct	T→C	ttgcttactt	20.90	1.79	17.71	1.62	2.64	10^{-2}	C			
		rs1735505029:T	cttttgctta	C→T	ttcctagtcc	20.90	1.79	13.18	1.21	7.35	10^{-6}	A			
		rs1736881700:G	tataataaat	A→G	catttctgtt	2.51	0.17	2.19	0.22	2.27	0.05	D			
		rs529278130:T	cttcttcgac	C→T	cagccttgca	51.47	3.67	43.69	3.13	3.24	10^{-2}	C			
		rs754074952:C	tgaacctctt	T→C	tatttgtagg	4.71	0.41	3.96	0.34	2.85	10^{-2}	C			
		rs757238850:A	cttatttgaa	C→A	ctcttttatt	4.71	0.41	4.15	0.31	2.21	0.05	D			
		rs914509126:A	tttgtaggtt	G→A	aagcactgga	4.71	0.41	3.36	0.31	5.31	10^{-6}	A			
		rs927217196:A	tttgcttact	T→A	cctagtcccg	20.90	1.79	5.60	0.63	18.70	10^{-6}	A			
		rs929450367:A	ggcggttccc	G→A	caccccagaa	62.21	4.67	50.41	3.58	4.07	10^{-3}	B			
		rs964688725:T	ctctcttctt	C→T	gacccagcct	51.47	3.67	39.47	2.63	5.44	10^{-6}	A			
		rs974026459:A	cggcgcacgt	G→A	ccccgtggaa	62.21	4.67	30.75	3.98	9.41	10^{-6}	A			
		rs983946884:A	gtgccccgtg	G→A	aaggggcggt	62.21	4.67	44.27	3.19	6.54	10^{-6}	A			
		rs987615193:G	ttcccgcacc	C→G	cagaaggagg	62.21	4.67	49.66	3.44	4.41	10^{-3}	B			

Table S3. Cont.

Human Gene #	Human Gene: *NCBI Gene Symbol* (NCBI Gene ID)	Candidate SNP marker: dbSNP ID:min [437]	5′ flank, 10 bp	WT →min	3 flank, 10 bp	K_D, nM, *in silico*: WT MEAN	± SEM	min MEAN	± SEM	Significance: Z	p	ϱ	Δ	Effect of changes in human gene expression on the development of primary open-angle glaucoma (POAG, ☼: "▼" aggravation, "▲" alleviation) [Reference]	☼ ▲ ▼
140	*TLR4* (7099)	rs1025141514:C	aagggctatt	T→C	taatagaacc	3.00	0.25	5.13	0.45	8.85	10^{-6}	A	↓	within human POAG models using Tlr4-null mice [403]: resistance to both artificially induced POAG and elevated intraocular pressure	▲
		rs1025141514:G	aagggctatt	T→G	taatagaacc	3.00	0.25	4.96	0.42	8.51	10^{-6}	A			
		rs1260074819:C	taattgggat	A→C	aaagccaact	6.13	0.53	16.39	1.31	16.77	10^{-6}	A			
		rs1399098379:G	tgaatacacc	A→G	attgctgtgg	7.41	0.76	8.56	0.75	2.13	0.05	D			
		rs1432819281:T	tagcccagaa	C→T	tgctttgaat	7.41	0.76	8.59	0.77	2.16	0.05	D			
		rs1829094210:T	ggataaaagc	C→T	aactagcttc	6.13	0.53	7.16	0.62	2.56	0.05	D			
		rs746941811:T	cccagaactg	C→T	tttgaataca	7.41	0.76	8.56	0.79	2.09	0.05	D			
		rs921693463:A	gctttgaata	C→A	accaattgct	7.41	0.76	10.63	0.88	5.47	10^{-6}	A			
		rs1829093149:C	tacaagggct	A→C	ttttaataga	3.00	0.25	2.61	0.25	2.16	0.05	D	↑	according to a retrospective review [404]: TLR4-excess can elevate both trabecular meshwork cell fibrosis and retinal ganglion cell death that can aggravate POAG	▼
		rs1829093184:A	caagggctat	T→A	ttaatagaac	3.00	0.25	2.34	0.16	4.55	10^{-3}	B			
141	*TMCO1* (54499)	rs1296877359:G	ttctccgata	C→G	tggccacacc	23.65	2.56	36.63	3.71	5.90	10^{-6}	A	↓	within a cohort-based biomedical study of gliomas and others TMCO1-related diseases [405]: TMCO1 dysfunction is closely related to a variety of human diseases, including glaucomas	▼
		rs1328770842:A	cgttctccga	T→A	cgtggccaca	23.65	2.56	41.86	4.36	7.60	10^{-6}	A			
		rs1328770842:C	cgttctccga	T→C	cgtggccaca	23.65	2.56	41.86	4.36	7.60	10^{-6}	A			
		rs1330784511:C	agttggtctg	T→C	cgggctgtca	17.65	2.08	38.69	3.23	10.87	10^{-6}	A			
		rs1330784511:G	agttggtctg	T→G	cgggctgtca	17.65	2.08	27.89	2.61	6.08	10^{-6}	A			
		rs1460101097:T	cgatacgagc	C→T	acaccgttct	23.65	2.56	29.32	2.97	2.90	10^{-2}	C			
		rs1571237356:C	tctccgatac	G→C	ggccacaccg	23.65	2.56	34.19	3.75	4.78	10^{-3}	B			
		rs373051778:C	agagttggtc	T→C	ttcgggctgt	17.65	2.08	28.35	2.47	6.47	10^{-6}	A			
		rs373051778:G	agagttggtc	T→G	ttcgggctgt	17.65	2.08	35.65	3.47	9.20	10^{-6}	A			
		rs765958391:C	ttggtctgtt	A→C	ggctgtcaga	17.65	2.08	41.86	4.36	10.99	10^{-6}	A			
		rs1040731918:T	cccgcgcccg	C→T	cacgctcccc	111.23	8.72	94.74	7.75	2.83	10^{-2}	C	↑	within a cohort-based biomedical micro-RNA comparative transcriptome study [406]: excessive hsa-miR-122-5p reducing TMCO1-target protein level in glaucomatous patients relatively its more higher norm in conventionally healthy volunteers can statistically significantly be a molecular marker for the exfoliation glaucoma, which is comorbide to POAG at least within their common biomedical associations with both decreased conventional aqueous humor outflow and increased intraocular pressure	▲
		rs1190207746:A	gctgtccccg	G→A	agggtctgct	111.23	8.72	97.76	6.89	2.45	0.05	D			
		rs1234195104:A	gagattcggc	C→A	ccaattctcg	24.54	2.08	20.31	1.58	3.29	10^{-2}	C			
		rs1256841275:T	cccggccatg	G→T	tgctcgctgt	111.23	8.72	46.97	3.66	15.60	10^{-6}	A			
		rs1264007339:A	ggtctgttac	T→A	ctgtcagagt	17.65	2.08	11.15	1.11	5.96	10^{-6}	A			
		rs1296877359:A	ttctccgata	C→A	tggccacacc	23.65	2.56	13.29	1.16	8.30	10^{-6}	A			
		rs1296877359:T	ttctccgata	C→T	tggccacacc	23.65	2.56	13.45	1.28	7.84	10^{-6}	A			
		rs1334999630:A	acaccgttct	C→A	ctctcgtggc	23.65	2.56	10.53	1.04	11.06	10^{-6}	A			
		rs1334999630:T	acaccgttct	C→T	ctctcgtggc	23.65	2.56	18.98	1.94	2.95	10^{-2}	C			
		rs1342190627:A	ctgcctgtgc	G→A	ccctcctccc	111.23	8.72	51.76	4.35	13.32	10^{-6}	A			
		rs140642978:G	ttggtttcct	C→G	tgagttgcgg	24.54	2.08	20.86	1.77	2.71	10^{-2}	C			
		rs140642978:T	ttggtttcct	C→T	tgagttgcgg	24.54	2.08	11.10	1.06	12.40	10^{-6}	A			
		rs1411818361:G	tggtctgtta	C→G	gctgtcagag	17.65	2.08	13.45	1.36	3.50	10^{-3}	B			
		rs1411818361:T	tggtctgtta	C→T	gctgtcagag	17.65	2.08	12.40	1.06	4.85	10^{-3}	B			
		rs1432218976:A	gttggtctgt	T→A	gggctgtcag	17.65	2.08	11.67	1.30	5.11	10^{-6}	A			
		rs1462179478:A	cctttccggt	G→A	tccgatacga	23.65	2.56	8.88	1.42	10.14	10^{-6}	A			
		rs1557828965:T	cccctcctcc	C→T	ggttcgcgcc	111.23	8.72	94.78	6.53	3.07	10^{-2}	C			
		rs1652695359:T	gggttggttt	C→T	cagtgagttg	24.54	2.08	19.86	1.50	3.74	10^{-3}	B			
		rs1652696189:A	cagctccagt	G→A	taccagccct	24.54	2.08	11.49	1.21	11.24	10^{-6}	A			
		rs1653925545:A	gcgaacggac	G→A	ccgctgcctg	111.23	8.72	64.71	5.86	9.05	10^{-6}	A			
		rs1653926469:T	cgccgcccct	C→T	gccatggttc	111.23	8.72	91.82	6.91	3.53	10^{-3}	B			
		rs1653926877:T	ccatggttcg	C→T	gctgtccccg	111.23	8.72	82.93	5.93	5.53	10^{-6}	A			
		rs368846066:T	cctgtgcgaa	C→T	tcctcccgct	111.23	8.72	60.25	4.74	11.04	10^{-6}	A			
		rs373091415:A	cgctgtcccc	G→A	cagggtctgc	111.23	8.72	88.68	6.91	4.10	10^{-3}	B			
		rs73014658:G	cctctagact	C→G	cgggttggtt	24.54	2.08	20.32	2.06	2.86	10^{-2}	C			

Table S3. Cont.

	Human Gene	Candidate SNP marker				K_D, nM, *in silico*				Significance				Effect of changes in human gene expression on the development of primary open-angle glaucoma (POAG, ☼: "▼" aggravation, "▲" alleviation) [Reference]	☼ ▲ ▼
#	*NCBI Gene Symbol* (NCBI Gene ID)	dbSNP ID:min [437]	5′ flank, 10 bp	WT →min	3 flank, 10 bp	WT MEAN	± SEM	min MEAN	± SEM	Z	p	ϱ	Δ		
141	*TMCO1* (54499)	rs745928907:T	cggtgctatg	C→T	acgagccttt	23.65	2.56	18.91	1.70	3.18	10^{-2}	C	↑	within a cohort-based biomedical micro-RNA comparative transcriptome study [406]: excessive hsa-miR-122-5p reducing TMCO1-target protein level in glaucomatous patients relatively its more higher norm in conventionally healthy volunteers can statistically significantly be a molecular marker for the exfoliation glaucoma, which is comorbide to POAG at least within their common biomedical associations with both decreased conventional aqueous humor outflow and increased intraocular pressure	▲
		rs749733764:T	ttcgggctgt	C→T	gaagtgcgat	17.65	2.08	13.00	1.20	4.09	10^{-3}	B			
		rs762686850:A	cccgctgcct	G→A	ccgcccctcc	111.23	8.72	37.62	3.40	18.12	10^{-6}	A			
		rs762686850:C	cccgctgcct	G→C	ccgcccctcc	111.23	8.72	89.80	6.76	3.94	10^{-3}	B			
		rs766189675:A	ccccggccat	G→A	ctgctcgctg	111.23	8.72	24.06	2.30	24.78	10^{-6}	A			
		rs766189675:T	ccccggccat	G→T	ctgctcgctg	111.23	8.72	43.32	2.77	18.63	10^{-6}	A			
		rs772201071:T	ttcctctaga	C→T	tgcgggttgg	24.54	2.08	20.81	1.86	2.68	10^{-2}	C			
		rs905494489:T	accgttctcc	G→T	ctcgtggcca	23.65	2.56	6.75	0.88	14.83	10^{-6}	A			
		rs909732960:G	gtctgttact	C→G	tgtcagagtt	17.65	2.08	14.38	1.58	2.55	0.05	D			
		rs953589173:A	gacgcacgct	C→A	ctgtgcgaac	111.23	8.72	91.69	8.12	3.27	10^{-2}	C			
		rs987700392:T	gtgcgaacgg	A→T	tcccgctgcc	111.23	8.72	87.93	8.29	3.83	10^{-3}	B			
142	*TMTC2* (160335)	rs1055662016:T	ggataagttg	G→T	tataaaactt	2.61	0.28	3.17	0.35	2.59	10^{-2}	C	↓	within a comprehensive biochemical study using RNA-interference targeted to mRNA transcribed from the human TMTC2 gene [407]: TMTC2 -knockdown elevates intracellular calcium release just like lanoprost does as a widely used anti-POAG medication [393]	▲
		rs1235772871:G	ataagttggt	A→G	taaaactttg	2.61	0.28	7.60	0.77	14.62	10^{-6}	A			
		rs1876454047:C	taagttggta	T→C	aaaactttga	2.61	0.28	7.14	0.72	13.81	10^{-6}	A			
		rs997032679:C	ttctcacttc	A→C	ctggagaagg	43.83	3.00	63.09	4.36	7.49	10^{-6}	A			
		rs997032679:G	ttctcacttc	A→G	ctggagaagg	43.83	3.00	60.52	4.19	6.62	10^{-6}	A			
		rs1010404090:T	ctggagtctc	C→T	agccaccgct	43.83	3.00	36.55	3.71	2.97	10^{-2}	C	↑	within a comprehensive biochemical study [407]: redox stress upregulates the human TMTC2 gene along with an acceleration in cellular senescence, which can contribute to POAG as an age-related disease in line with an exhaustive retrospective biomedical review [408]	▼
		rs1157270554:A	tctccagcca	C→A	cgcttctcac	43.83	3.00	38.52	2.45	2.77	10^{-2}	C			
		rs1407024265:A	caccgcttct	C→A	acttcactgg	43.83	3.00	18.53	1.45	16.60	10^{-6}	A			
		rs1417076334:C	ccaccgcttc	T→C	cacttcactg	43.83	3.00	37.44	2.86	3.07	10^{-2}	C			
		rs1427337080:A	ggaggatctg	G→A	gtggagaaag	50.51	3.98	44.23	3.46	2.39	0.05	D			
		rs1872319591:A	gggaggatct	G→A	ggtggagaaa	50.51	3.98	24.46	2.12	12.37	10^{-6}	A			
		rs1872320090:A	tctgggtgga	G→A	aaagggggctg	50.51	3.98	36.24	2.81	6.01	10^{-6}	A			
		rs1872323077:T	gagggttggt	C→T	acgcaggcgc	50.51	3.98	38.82	3.62	4.31	10^{-3}	B			
		rs1872329391:A	ctccagccac	C→A	gcttctcact	43.83	3.00	38.52	2.38	2.80	10^{-2}	C			
		rs1872329391:T	ctccagccac	C→T	gcttctcact	43.83	3.00	38.52	2.42	2.78	10^{-2}	C			
		rs569911677:A	ggttggtcac	G→A	caggcgcggg	50.51	3.98	38.65	3.36	4.57	10^{-3}	A			
		rs74106267:A	tctcacttca	C→A	tggagaaggg	43.83	3.00	27.30	1.91	9.68	10^{-6}	A			
		rs74106267:T	tctcacttca	C→T	tggagaaggg	43.83	3.00	25.91	1.61	11.37	10^{-6}	A			
		rs930371334:C	gtataaaact	T→C	tgaagaacaa	2.61	0.28	1.96	0.20	3.85	10^{-3}	B			
143	*TNF* (7124)	rs1245601180:G	ttcctctcac	A→G	tactgaccca	7.17	0.78	11.16	1.18	5.85	10^{-6}	A	↓	within human POAG models using Sprague-Dawley rats injected with hyaluronic acid into the anterior chamber of the eye during six-weeks and, next after POAG symptom detection, treated with myricetin as a promising phytomedication against POAG [409]: Tnf downregulation along with lowered levels in reactive oxidative species (ROS), lipid peroxidation products, pro-inflammatory cytokines and intraocular pressure that altogether can relieve POAG	▲
		rs1473111053:A	cagggacata	T→A	aaaggcagtt	2.23	0.25	3.86	0.34	7.61	10^{-6}	A			
		rs1771139893:G	tcttcctctc	A→G	catactgacc	7.17	0.78	18.70	1.99	12.64	10^{-6}	A			
		rs372568141:A	tcctctcaca	T→A	actgacccac	7.17	0.78	19.77	1.56	15.13	10^{-6}	A			
		rs55978323:A	tataaaggca	G→A	ttgttggcac	2.23	0.25	3.07	0.32	4.14	10^{-3}	B			
		rs961336693:C	taaaggcagt	T→C	gttggcacac	2.23	0.25	2.74	0.24	2.87	10^{-2}	C			
		rs1771139729:T	ctcttcctct	C→T	acatactgac	7.17	0.78	2.73	0.31	12.34	10^{-6}	A	↑	within human POAG emergence models using Sprague-Dawley rats injected streptozotocin in citric buffer and, next after diabetic symptom detection, rat retinal tissue samples were prepared and studied using RT-qPCR [410]: accelerated apoptosis of retinal ganglion cells along with Tnf excess, both of which can correspond to something like POAG emergence together with a promising molecular marker for early POAG	▼

Table S3. Cont.

Human Gene		Candidate SNP marker				K$_D$, nM, *in silico*				Significance				Effect of changes in human gene expression on the development of primary open-angle glaucoma (POAG, ☼: "▼" aggravation, "▲" alleviation) [Reference]	☼ ▲ ▼
#	*NCBI Gene Symbol* (NCBI Gene ID)	dbSNP ID:min [437]	5′ flank, 10 bp	WT→min	3 flank, 10 bp	WT MEAN	± SEM	min MEAN	± SEM	Z	p	ϱ	Δ		
144	*TP53* (7157)	rs1189631200:A	ggattacttg	C→A	ctccggcagg	14.84	1.10	16.42	1.15	1.99	0.05	D	↓	according to complex biomedical analysis of association between SNP rs4938723:C (i.e., ancestral allele "tttgacctatTacagctctca" => minor allele "tttgacctatCacagctctca") and POAG in Chinese population using luciferase assays, in-silicon analyses, real-time PCR, Western Blot, and flow cytometry and many other methods [411]: minor allele "C" can downregulate TP53 expression, thus leading to suppressed cell apoptosis and enhanced cell proliferation that could reduce susceptibility to POAG	▲
		rs125729208:C	gcgctaaaag	T→C	gctcaagact	19.96	1.68	24.07	2.14	3.06	10^{-2}	C			
		rs1399459244:C	agagatgaga	T→C	agtggttgtt	8.59	0.59	14.22	1.23	9.15	10^{-6}	A			
		rs1469696496:C	ctaaaagttt	T→C	caagactggc	19.96	1.68	28.35	2.32	5.98	10^{-6}	A			
		rs1480412583:G	tccccacttt	T→G	ctcatgctgg	34.03	2.44	43.53	3.47	4.60	10^{-3}	B			
		rs1555527049:G	ggatccccac	T→G	tgtctcatgc	34.03	2.44	43.53	3.47	4.60	10^{-3}	B			
		rs1597372671:C	ggtggtgcac	A→C	tagccaggca	6.34	0.69	7.96	0.67	3.30	10^{-3}	B			
		rs1597378578:G	tgttaagaga	T→G	gagggagtgg	8.59	0.59	9.50	0.63	2.13	0.05	D			
		rs2073426331:G	acacctatag	T→G	catggtggtg	6.34	0.69	7.67	0.91	2.36	0.05	D			
		rs2073426460:G	gcacacctat	A→G	ggcatggtgg	6.34	0.69	7.96	0.67	3.30	10^{-3}	B			
		rs759055064:A	gctggatccc	C→A	aagtgtctca	34.03	2.44	37.54	2.57	1.98	0.05	D			
		rs866946974:C	gatgagatta	A→C	ggttgttaag	8.59	0.59	13.99	1.41	8.00	10^{-6}	A			
		rs892070762:T	tcaagactgg	C→T	ctcccatgtg	19.96	1.68	25.74	2.39	4.06	10^{-3}	B			
		rs927625987:A	ttaaagaagc	C→A	aagagatgag	8.59	0.59	10.36	0.77	3.70	10^{-3}	B			
		rs1054623097:T	gtccagggag	C→T	ctagagccac	19.48	1.54	12.39	1.47	6.34	10^{-6}	A	↑	according to a cohort-based biomedical study in Poland [412]: TP53 upregulation can promote cell death through apoptosis during neurodegenerative processes aggravating POAG	▼
		rs1175544880:T	ggaagtgtct	C→T	gacccagggt	34.03	2.44	11.28	1.00	19.41	10^{-6}	A			
		rs1238481862:A	ttttcctctt	G→A	tggatcccca	34.03	2.44	11.69	1.07	18.40	10^{-6}	A			
		rs1409841774:A	caagactggc	G→A	tcccatgtgc	19.96	1.68	8.42	0.73	14.26	10^{-6}	A			
		rs1409841774:C	caagactggc	G→C	tcccatgtgc	19.96	1.68	14.36	1.09	5.82	10^{-6}	A			
		rs1428882924:A	gcccttactt	G→A	gcggattact	14.84	1.10	5.31	0.52	16.77	10^{-6}	A			
		rs1457582183:T	tctttcctag	C→T	ctttccttgc	7.46	0.63	6.04	0.61	3.22	10^{-2}	C			
		rs1469696496:C	ctaaaagttt	T→C	caagactggc	19.48	1.54	16.67	1.45	2.65	10^{-2}	C			
		rs1597359927:C	cacttttatc	A→C	tgcctcagat	7.46	0.63	6.62	0.57	2.00	0.05	D			
		rs1597378558:A	gagatgagat	T→A	gtggttgtta	8.59	0.59	5.68	0.48	7.64	10^{-6}	A			
		rs1597400604:A	cagtcaggag	C→A	ttacccaatc	47.80	4.43	34.32	2.52	5.61	10^{-6}	A			
		rs185172744:T	acttttatca	C→T	gcctcagatt	7.46	0.63	6.60	0.56	2.07	0.05	D			
		rs2073428554:T	tctacaaaaa	A→T	gaaacctcgt	6.34	0.69	3.77	0.42	6.68	10^{-6}	A			
		rs2073527009:A	gtctcatgct	G→A	gggttggaag	34.03	2.44	25.76	2.31	4.85	10^{-3}	B			
		rs2074028341:A	taaaagtttt	G→A	aagactggcg	19.96	1.68	14.54	0.81	6.30	10^{-6}	A			
		rs2074029420:T	tttcccctcc	C→T	gggattgggg	19.96	1.68	10.36	0.92	10.71	10^{-6}	A			
		rs2074029490:A	ttggggtttt	C→A	ggttgatggg	19.96	1.68	14.79	1.41	4.72	10^{-3}	B			
		rs2074030159:T	cgcaggggtt	G→T	gccaggagcc	19.96	1.68	11.58	1.31	7.72	10^{-6}	A			
		rs34361146:A	ttgcctcttt	C→A	atcacctttc	7.46	0.63	4.96	0.51	6.17	10^{-6}	A			
		rs945036538:T	accaagggtg	C→T	cggtggagga	7.46	0.63	5.16	0.49	5.86	10^{-6}	A			
145	*TRPC6* (7225)	rs1184993479:C	gccaactaaa	T→C	ttactaaatg	3.89	0.44	4.65	0.46	2.37	0.05	D	↓	within human POAG cellular models using the human lamina cribrosa cells subjected with hydrogen peroxide as an oxidative stressor inducing POAG along with either small interference RNA targeted to TRPC6 or low molecular weight TRPC6-inhibitor SKF96365 as a new promising medications against POAG [413]: TRPC6 downregulation along with slowing down cell proliferation that can prevent fibrosis causing optic neuropathy in POAG	▲
		rs1184993479:G	gccaactaaa	T→G	ttactaaatg	3.89	0.44	4.65	0.46	2.37	0.05	D			
		rs1680919324:C	aactaaatag	T→C	ctaaatgagc	3.89	0.44	4.65	0.46	2.37	0.05	D			
		rs1862272870:C	atgagccaac	T→C	atccttacta	3.89	0.44	4.65	0.46	2.37	0.05	D			
		rs920676961:A	aatgagccaa	C→A	tatccttact	3.89	0.44	4.92	0.39	3.39	10^{-3}	B			
		rs980854362:C	tccccgctgg	A→C	tccgcaccgg	88.01	7.29	195.48	15.38	13.97	10^{-6}	A			
		rs980854362:G	tccccgctgg	A→G	tccgcaccgg	88.01	7.29	195.48	15.53	13.91	10^{-6}	A			

Table S3. Cont.

Human Gene		Candidate SNP marker				K_D, nM, *in silico*				Significance				Effect of changes in human gene expression on the development of primary open-angle glaucoma (POAG, ☼: “▼” aggravation, “▲” alleviation) [Reference]	☼
						WT		min							
#	*NCBI Gene Symbol* (NCBI Gene ID)	dbSNP ID:min [437]	5′ flank, 10 bp	WT→min	3 flank, 10 bp	MEAN	± SEM	MEAN	± SEM	Z	p	ϱ	Δ		▲ ▼
145	*TRPC6* (7225)	rs1354737331:A	accgggtccc	C→A	gaaccctccg	88.01	7.29	70.29	5.75	3.86	10^{-3}	B	↑	within human eye disease models using adult male rats subjected to retinal ischemia induced by ophthalmic vessel ligation for 60 minutes, and after 5 minutes complete restored perfusion was found [414]: Trpc6 excess before ischemia-reperfusion injury has an early neuroprotective effect on retinal ganglion cells promoting their survival, which may alleviate POAG	▲
		rs1369745868:T	gaaccctccg	C→T	gccgctgacg	88.01	7.29	55.07	6.00	6.85	10^{-6}	A			
		rs1862254427:A	cgctggaact	G→A	caccgggtcc	88.01	7.29	46.70	4.30	10.24	10^{-6}	A			
		rs1862254589:T	gtccccgctg	G→T	ctccgcaccg	88.01	7.29	12.06	1.33	28.78	10^{-6}	A			
		rs1862254627:A	ggtccccgct	G→A	cctccgcacc	88.01	7.29	16.51	1.61	26.13	10^{-6}	A			
		rs1862272686:A	actaaatagt	G→A	taaatgagcc	3.89	0.44	3.18	0.31	2.67	10^{-2}	C			
		rs1862272834:C	gagccaacta	A→C	ccttactaaa	3.89	0.44	2.89	0.34	3.65	10^{-3}	B			
		rs759326295:A	cttactaaat	G→A	cagttggtat	3.89	0.44	2.86	0.26	4.23	10^{-3}	B			
146	*TRPM5* (29850)	rs1374998726:C	cccgtctgca	T→C	cttggccccg	79.41	5.91	94.25	6.63	3.35	10^{-3}	B	↓	within human disease models using Trpm5-deficient mice [415]: increased susceptibility to bacterial infections that can aggravate POAG according to a biomedical case report [150]	▼
		rs1589883730:C	ccccgtctgc	A→C	gcttggcccc	79.41	5.91	94.25	6.63	3.35	10^{-3}	B			
		rs1589883730:T	ccccgtctgc	A→T	gcttggcccc	79.41	5.91	94.25	6.63	3.35	10^{-3}	B			
		rs1298825646:T	gccccgtctg	C→T	tgcttggccc	79.41	5.91	13.71	1.40	27.77	10^{-6}	A	↑	according to a nutritional study of natural herbal products that can stimulate people to a healthy lifestyle [416]: bitter melon extract upregulates TRPM5 contributing to aversion to high salt concentrations that can prevent high salt provoking cardiovascular diseases, especially hypertension as a risk factor of POAG [417]	▲
		rs1336754556:T	cgcccacctg	C→T	cgccccctcc	79.41	5.91	49.69	3.45	9.21	10^{-6}	A			
		rs1445939679:A	gccccgcccc	G→A	ccacctgctt	79.41	5.91	56.23	4.21	6.54	10^{-6}	A			
		rs1589883747:A	tcacccacct	G→A	tggccccgcc	79.41	5.91	21.42	2.17	20.84	10^{-6}	A			
		rs1845795168:A	cagctctaga	G→A	gggtatccct	31.86	3.06	20.75	2.18	6.03	10^{-6}	A			
		rs1845795675:A	gccttctcag	C→A	ggtggggatg	51.49	5.11	39.61	2.91	4.25	10^{-3}	B			
		rs1845997729:T	tctgcatctc	C→T	gccccgcccc	79.41	5.91	65.77	4.88	3.58	10^{-3}	B			
		rs1845998299:A	ccacctgctt	G→A	cccgccctca	79.41	5.91	54.78	4.79	6.47	10^{-6}	A			
		rs1845998461:G	cctcacccac	C→G	cttggccccg	79.41	5.91	49.80	3.87	8.68	10^{-6}	A			
		rs1845998573:T	cgccctcacc	C→T	ctgcttggcc	79.41	5.91	18.52	1.83	23.55	10^{-6}	A			
		rs1845998695:T	ttggccccgc	C→T	cgcccacctg	79.41	5.91	63.57	4.91	4.15	10^{-3}	B			
		rs747426352:T	agccctcaag	G→T	tgggccttct	51.49	5.11	37.28	2.79	5.20	10^{-6}	A			
		rs748671564:T	gccagggtat	C→T	agccctcaag	51.49	5.11	30.15	2.58	8.18	10^{-6}	A			
		rs755625794:T	caccatgcag	G→T	gctctagagg	31.86	3.06	15.19	1.49	10.79	10^{-6}	A			
		rs758760094:A	aagggccagg	G→A	tctcagccct	51.49	5.11	30.76	3.61	6.71	10^{-6}	A			
		rs777001650:T	tgggccttct	C→T	ggaggtgggg	51.49	5.11	25.13	2.00	11.29	10^{-6}	A			
		rs778050073:A	tcaagggcca	G→A	cttctcagcc	51.49	5.11	28.25	3.06	8.18	10^{-6}	A			
		rs779380739:T	ggccaccatg	C→T	gcagctctag	31.86	3.06	13.57	1.57	11.36	10^{-6}	A			
		rs995032702:A	tatccctgca	G→A	aagggccagg	31.86	3.06	21.00	2.24	5.81	10^{-6}	A			
147	*TXNRD2* (10587)	rs1044214796:T	tttggaattt	G→T	cgacattctg	15.26	1.25	23.85	2.24	7.16	10^{-6}	A	↓	according to a comprehensive molecular biochemical review [418]: TXNRD2 deficiency can lead to an increase in mitochondrial reactive oxygen species (ROS) playing a key role in the pathogenesis of POAG [419]	▼
		rs1245768697:G	caagtacttt	A→G	ttcagaaaag	5.27	0.49	8.43	0.70	7.49	10^{-6}	A			
		rs1318247389:G	cgggcagccc	T→G	aggcagcgcg	64.52	5.77	136.72	10.85	12.56	10^{-6}	A			
		rs1482381664:G	agataagcac	A→G	caagctgccc	10.33	1.05	12.06	1.27	2.12	0.05	D			
		rs1601501281:C	gggcagccct	A→C	ggcagcgcgg	64.52	5.77	136.72	10.95	12.50	10^{-6}	A			
		rs182857388:T	aagtcaagta	C→T	atctttcaga	5.27	0.49	6.35	0.64	2.70	10^{-2}	C			
		rs1829944708:C	gatgtttccg	T→C	ccgcccgccg	6.08	0.66	7.31	0.78	2.41	0.05	D			
		rs1938768615:C	tttccgtagt	T→C	ccgccgggat	6.08	0.66	7.31	0.78	2.41	0.05	D			
		rs1940614037:C	tcaagtactt	T→C	tttcagaaaa	5.27	0.49	8.43	0.70	7.49	10^{-6}	A			
		rs1941737286:C	tagccagcgc	T→C	cggccccggt	103.03	7.08	121.60	8.57	3.36	10^{-3}	B			
		rs555437903:G	ctagccagcg	C→G	tcggccccgg	103.03	7.08	132.03	9.36	5.02	10^{-6}	A			
		rs756742203:G	gtactttaac	A→G	agaaaagtca	5.27	0.49	8.43	0.70	7.49	10^{-6}	A			
		rs767066477:A	ggcagcccta	G→A	gcagcgcggc	121.43	12.45	151.40	11.41	3.47	10^{-3}	B			

Table S3. Cont.

#	Human Gene: NCBI Gene Symbol (NCBI Gene ID)	Candidate SNP marker: dbSNP ID:min [437]	5′ flank, 10 bp	WT →min	3 flank, 10 bp	K_D, nM, *in silico*: WT MEAN	± SEM	min MEAN	± SEM	Significance: Z	p	ϱ	Δ	Effect of changes in human gene expression on the development of primary open-angle glaucoma (POAG, ☼: "▼" aggravation, "▲" alleviation) [Reference]	☼
147	*TXNRD2* (10587)	rs778735410:C	ggatgtttcc	G→C	gccgcccgcc	6.08	0.66	7.31	0.78	2.41	0.05	D	↓	according to a comprehensive molecular biochemical review [418]: TXNRD2 deficiency can lead to an increase in mitochondrial reactive oxygen species (ROS) playing a key role in the pathogenesis of POAG [419]	▼
		rs886509891:T	aaagtcaagt	A→T	tatctttcag	5.27	0.49	6.82	0.51	4.30	10^{-3}	B			
		rs887410599:G	ttccgtagtt	A→G	cgccgggatg	6.08	0.66	7.31	0.78	2.41	0.05	D			
		rs986793121:C	acatccatac	A→C	ccaagataag	10.33	1.05	16.84	1.55	7.11	10^{-6}	A			
		rs986793121:G	acatccatac	A→G	ccaagataag	10.33	1.05	16.84	1.61	6.99	10^{-6}	A			
		rs1037729705:A	gggggctatt	G→A	gtgggcgggg	6.08	0.66	3.64	0.41	6.58	10^{-6}	A	↑	within human lifespan models using transgenic fruit flies overexpressing Txnrd2 verified with many independent transcriptome profiling data on long-lived primate and rodent species [420]: Txnrd2 excess is a biomedical molecular marker for susceptibility to longevity that seems to be fitting the intraocular pressure decrease with age increase in the human POAG model using transgenic mice [421]	▲
		rs1051864281:A	gcccgggact	G→A	cgcgacggcc	103.03	7.08	29.02	2.75	21.62	10^{-6}	A			
		rs1213698422:A	ggcgggcagc	C→A	gcaggcagcg	121.43	12.45	49.78	6.29	10.95	10^{-6}	A			
		rs1213698422:T	ggcgggcagc	C→T	gcaggcagcg	121.43	12.45	40.75	4.44	14.60	10^{-6}	A			
		rs1218905301:A	cacatccata	C→A	cccaagataa	10.33	1.05	5.87	0.51	8.41	10^{-6}	A			
		rs1229309666:T	cctagctgcc	C→T	cggcgggcag	64.52	5.77	56.06	5.04	2.22	0.05	D			
		rs1270432241:T	gcgcggcggg	C→T	cggcgcaggc	121.43	12.45	88.36	8.01	4.65	10^{-3}	B			
		rs1271770673:T	acctctcccg	C→T	ccagcgctct	121.43	12.45	98.21	8.00	3.24	10^{-2}	C			
		rs1325528256:G	gcgctctcac	C→G	cggtctagcc	103.03	7.08	58.78	3.72	12.01	10^{-6}	A			
		rs1325528256:T	gcgctctcac	C→T	cggtctagcc	103.03	7.08	49.15	3.42	15.14	10^{-6}	A			
		rs1348707202:G	tctcacctct	C→G	ctagccagcg	103.03	7.08	89.73	6.07	2.87	10^{-2}	C			
		rs1402974879:T	ccctagctgc	C→T	gcggcgggca	64.52	5.77	53.63	4.82	2.91	10^{-2}	C			
		rs1459413191:A	actttaacat	C→A	aaaagtcaag	5.27	0.49	2.20	0.19	13.69	10^{-6}	A			
		rs149615777:C	agtttacata	A→C	cagacaccat	4.77	0.47	3.77	0.34	3.49	10^{-3}	B			
		rs1941409095:A	tgctattatt	T→A	gactgatgac	6.79	0.59	3.53	0.32	10.37	10^{-6}	A			
		rs1941595493:A	agactagtat	G→A	cagaattaat	4.77	0.47	2.27	0.26	9.90	10^{-6}	A			
		rs1941596844:T	cataagttta	C→T	ttggcagaca	4.77	0.47	2.27	0.20	11.15	10^{-6}	A			
		rs1941732671:T	ctagctgccc	C→T	ggcgggcagc	64.52	5.77	41.37	3.80	6.93	10^{-6}	A			
		rs1941733329:A	cggcgggcag	C→A	cgcaggcagc	121.43	12.45	31.11	3.21	18.74	10^{-6}	A			
		rs1941734145:A	tccggcgcag	G→A	acccgccccg	121.43	12.45	81.07	6.41	6.24	10^{-6}	A			
		rs1941736950:T	ctcacctctc	C→T	tagccagcgc	121.43	12.45	85.07	6.17	5.67	10^{-6}	A			
		rs1941737126:G	gctctcacct	C→G	gtctagccag	103.03	7.08	74.90	5.27	6.48	10^{-6}	A			
		rs33997573:A	attgtttaat	G→A	gggcgggggc	6.08	0.66	3.82	0.42	6.04	10^{-6}	A			
		rs530504248:A	gggatgtttc	C→A	cgccgcccgc	6.08	0.66	4.78	0.49	3.22	10^{-2}	C			
		rs549728841:T	ccggcgcagg	C→T	cccgccccgg	103.03	7.08	48.79	4.74	12.57	10^{-6}	A			
		rs764956654:T	cctctcccgc	G→T	cagcgctctc	121.43	12.45	96.08	7.42	3.65	10^{-3}	B			
		rs767066477:A	ggcagcccta	G→A	gcagcgcggc	64.52	5.77	25.96	2.16	14.88	10^{-6}	A			
		rs767066477:C	ggcagcccta	G→C	gcagcgcggc	64.52	5.77	41.59	3.93	6.75	10^{-6}	A			
		rs958349283:T	tcacctctcc	C→T	agccagcgct	121.43	12.45	84.93	6.71	5.52	10^{-6}	A			
148	*VAV2* (7410)	rs1231994678:C	cgggggacct	G→C	cggcggcgca	196.36	15.58	225.63	17.02	2.54	0.05	D	↓	within human POAG models using Vav2-deficient mice [422]: early onset of iridocorneal angle changes and elevated intraocular pressure, with subsequent selective loss of retinal ganglion cells and optic nerve head cupping, which are the hallmarks of POAG	▼
		rs1237449790:C	ccgctgcact	T→C	atgccccgcg	69.04	5.47	131.98	10.64	11.47	10^{-6}	A			
		rs537388617:T	ctttggctcg	A→T	cggccgctgc	69.04	5.47	78.36	6.99	2.12	0.05	D			
		rs755632731:C	cgctgcactt	T→C	tgccccgcgg	69.04	5.47	81.43	6.12	3.02	10^{-2}	C			
		rs918726623:C	gcgggggacc	T→C	acggcggcgc	196.36	15.58	232.39	16.44	3.17	10^{-2}	C			
		rs918726623:G	gcgggggacc	T→G	acggcggcgc	196.36	15.58	232.39	16.44	3.17	10^{-2}	C			
		rs931870860:G	cggccgctgc	A→G	gggatgcccc	69.04	5.47	127.32	9.21	11.41	10^{-6}	A			

Table S3. Cont.

#	*NCBI Gene Symbol* (NCBI Gene ID)	dbSNP ID:min [437]	5′ flank, 10 bp	WT →min	3 flank, 10 bp	WT MEAN	WT ± SEM	min MEAN	min ± SEM	Z	p	ϱ	Δ	Effect of changes in human gene expression on the development of primary open-angle glaucoma (POAG, ☼: "▼" aggravation, "▲" alleviation) [Reference]	☼ ▲ ▼
	**Human Gene**	**Candidate SNP marker**				**K_D, nM, *in silico***				**Significance**					
		rs1001764582:T	cgccccggcc	C→T	gcgcgaagcc	196.36	15.58	115.95	8.35	9.84	10^{-6}	A			
		rs1161050328:A	cgggcgggat	G→A	ccgcgcgcgc	196.36	15.58	95.27	11.36	10.10	10^{-6}	A			
		rs1180927579:T	tttggctcga	G→T	ggccgctgca	69.04	5.47	50.57	4.14	5.47	10^{-6}	A			
		rs1253955850:T	ggccgctgca	C→T	ggatgccccg	69.04	5.47	58.38	4.70	2.97	10^{-2}	C			
		rs1351676886:T	cggccctgag	C→T	aagccccgcc	196.36	15.58	102.94	7.45	12.03	10^{-6}	A			
		rs1485226167:T	ccccgccggg	C→T	ggagcgagcg	26.92	2.03	21.65	1.65	4.05	10^{-3}	B			
		rs1650214877:A	ggggacctgg	G→A	gcggcgcagc	196.36	15.58	158.78	12.24	3.84	10^{-3}	B			
		rs1843054437:T	tgcactttgg	C→T	cccgcggccg	69.04	5.47	43.31	2.96	8.91	10^{-6}	A			
		rs1843056948:T	ccgccccggc	C→T	cgcgcgaagc	196.36	15.58	144.48	10.59	5.68	10^{-6}	A			
		rs1843057949:T	ccgcgcgaag	C→T	cctggggcag	196.36	15.58	118.69	8.35	9.49	10^{-6}	A			
		rs1843058055:T	cccgcgcgaa	G→T	acctggggca	196.36	15.58	170.53	12.06	2.65	10^{-2}	C			
		rs1843058168:T	gcagcccgcg	C→T	ggggacctgg	196.36	15.58	75.24	5.88	17.23	10^{-6}	A			
148	*VAV2* (7410)	rs1843058388:A	ggggcagccc	G→A	gcgggggacc	196.36	15.58	136.78	9.96	6.72	10^{-6}	A	↑	within human neurodevelopment models using transgenic Vav2 gain-of-function chicken (*Gallus gallus domesticus*) [423]: Vav2 excess in the chick spinal cord along with an improved limb trajectory of motor axon growth that might contribute to both neuroprotection and neuroregeneration during POAG according to an exhaustive biomedical review [424]	▲
		rs1843059598:A	agcgggggac	C→A	cacggcggcg	196.36	15.58	62.33	5.04	20.27	10^{-6}	A			
		rs1843073104:T	cccgagcgca	G→T	ccgcggggcg	26.92	2.03	16.69	1.66	7.66	10^{-6}	A			
		rs1843075407:T	gcctttgtct	C→T	gaccccgccg	26.92	2.03	23.33	1.73	2.70	10^{-2}	C			
		rs1843075521:G	gggcctttgt	C→G	gcgaccccgc	26.92	2.03	17.01	1.29	8.57	10^{-6}	A			
		rs368292098:A	ctgcactttg	G→A	ccccgcggcc	69.04	5.47	31.39	2.25	14.77	10^{-6}	A			
		rs575492766:A	cctttgtctc	G→A	accccgccgg	26.92	2.03	22.37	1.68	3.47	10^{-3}	B			
		rs761647218:A	cactttggct	C→A	cgcggccgct	69.04	5.47	50.39	4.76	5.11	10^{-6}	A			
		rs762885980:A	ctcgagcact	G→A	ctgcactttg	69.04	5.47	44.35	5.17	6.28	10^{-6}	A			
		rs913391452:T	cagcggggga	C→T	gcacggcggc	196.36	15.58	111.81	8.71	10.13	10^{-6}	A			
		rs936173791:T	ttggctcgag	C→T	gccgctgcac	69.04	5.47	23.30	3.64	12.42	10^{-6}	A			
		rs967453198:T	cgcagcgggg	G→T	acgcacggcg	196.36	15.58	39.63	4.42	23.40	10^{-6}	A			
		rs976584809:A	gcgcagcggg	G→A	gacgcacggc	196.36	15.58	140.46	12.45	5.63	10^{-6}	A			
		rs1367564813:A	tcccggagcc	G→A	gcccagccgc	101.00	7.64	122.98	9.29	3.68	10^{-3}	B			
		rs1418740607:G	cccggagccg	T→G	cccagccgcg	101.00	7.64	129.08	9.63	4.62	10^{-3}	B			
		rs142499589:T	gcatggagcc	G→T	ggagccgtcg	101.00	7.64	123.50	9.32	3.76	10^{-3}	B			
		rs1659194562:C	cgtggaaata	T→C	gcctgaggga	3.20	0.45	8.30	0.81	11.21	10^{-6}	A			
		rs1659194720:G	ccgtggaaat	A→G	agcctgaggg	3.20	0.45	5.41	0.55	6.08	10^{-6}	A	↓	within the human disease models using Vav3-knockout mice [422]: a glaucoma-like phenotype that corresponds to SNP in Japanese patients with POAG	▼
		rs1675352464:T	tcgggcatgg	A→T	tcccggagcc	101.00	7.64	151.04	11.04	7.65	10^{-6}	A			
		rs1675352693:C	cgtcgggcat	G→C	cgtcccggag	101.00	7.64	129.64	10.02	4.62	10^{-3}	B			
		rs1675379489:G	gcgtctgggg	A→G	ctcccacccg	104.20	8.16	164.20	13.46	8.02	10^{-6}	A			
		rs776864629:G	gccgtcgggc	A→G	cgcgtcccgg	101.00	7.64	151.04	11.04	7.65	10^{-6}	A			
149	*VAV3* (10451)	rs1024431185:A	gaccggagcc	G→A	acccgggccg	155.00	12.48	122.64	10.49	3.99	10^{-3}	B			
		rs1163949175:A	cggcgcgctc	C→A	gccccggccg	104.20	8.16	76.54	6.28	5.44	10^{-6}	A			
		rs1176550082:A	ggagccgtcg	G→A	agccgcgtcc	101.00	7.64	69.08	5.27	7.07	10^{-6}	A			
		rs1210629398:T	gcggcggccg	G→T	gactcgctgc	155.00	12.48	112.26	9.26	5.60	10^{-6}	A			
		rs1297785617:T	cccgggccgg	G→T	gtcgcggcgc	155.00	12.48	71.62	9.05	10.31	10^{-6}	A			
		rs1320833419:T	agaccggccg	G→T	ggaccgcagc	104.20	8.16	74.00	5.97	6.09	10^{-6}	A	↑	according to an exhaustive review [425]: Vav3 activation may promote granule cell survival in the cerebellum that may slow the progression of POAG accompanied by neurodegeneration throughout the whole brain, especially severe in the cerebellum, oculomotor regions, visual cortices and frontal lobe, in line with a biomedical cohort-based study using magnetic resonance imaging [426]	▲
		rs1328089001:A	cttgcggacc	G→A	gcgtctgggg	104.20	8.16	93.42	7.38	1.96	0.05	D			
		rs1333962758:T	cggaccgcag	C→T	ctggggactt	104.20	8.16	73.61	5.87	6.22	10^{-6}	A			
		rs1345275106:A	gccgagccta	G→A	ccgggaccgg	155.00	12.48	45.81	5.02	17.93	10^{-6}	A			
		rs1380192046:A	ggccgggacc	G→A	cggcgcaccc	155.00	12.48	111.34	8.49	5.97	10^{-6}	A			
		rs1392497699:A	agcctagcgc	G→A	gaccggagcc	155.00	12.48	133.97	12.43	2.37	0.05	D			

Table S3. Cont.

Human Gene		Candidate SNP marker				KK_D, nM, *in silico*				Significance				Effect of changes in human gene expression on the development of primary open-angle glaucoma (POAG, ☼: "▼" aggravation, "▲" alleviation) [Reference]	☼
#	*NCBI Gene Symbol* (NCBI Gene ID)	dbSNP ID:min [437]	5′ flank, 10 bp	WT →min	3 flank, 10 bp	WT MEAN	± SEM	min MEAN	± SEM	Z	p	ϱ	Δ		▲▼
149	*VAV3* (10451)	rs1405902313:T	cctagcgcgg	C→T	ccggagccga	155.00	12.48	137.12	10.89	2.17	0.05	D	↑	according to an exhaustive review [425]: Vav3 activation may promote granule cell survival in the cerebellum that may slow the progression of POAG accompanied by neurodegeneration throughout the whole brain, especially severe in the cerebellum, oculomotor regions, visual cortices and frontal lobe, in line with a biomedical cohort-based study using magnetic resonance imaging [426]	▲
		rs1414865161:A	ccggagccga	G→A	ccgggccggg	155.00	12.48	55.16	4.97	17.11	10^{-6}	A			
		rs1480410085:A	gcggccgcat	C→A	cggcggcggc	101.00	7.64	18.77	2.19	24.22	10^{-6}	A			
		rs1480410085:T	gcggccgcat	C→T	cggcggcggc	101.00	7.64	52.02	3.00	13.94	10^{-6}	A			
		rs1488946498:A	gcccgcgacc	C→A	cctagcgcgg	155.00	12.48	119.82	8.09	4.90	10^{-6}	A			
		rs1488946498:T	gcccgcgacc	C→T	cctagcgcgg	155.00	12.48	83.17	6.15	11.39	10^{-6}	A			
		rs1675352327:A	cgggcatgga	G→A	cccggagccg	101.00	7.64	80.23	5.99	4.33	10^{-3}	B			
		rs1675352693:A	cgtcgggcat	G→A	cgtcccggag	101.00	7.64	29.11	2.77	20.45	10^{-6}	A			
		rs1675365429:A	cgagcctagc	G→A	gggaccggag	155.00	12.48	67.71	6.58	13.13	10^{-6}	A			
		rs1675365429:T	cgagcctagc	G→T	gggaccggag	155.00	12.48	95.12	7.93	8.43	10^{-6}	A			
		rs1675366068:T	cggagccgag	C→T	cgggccggga	155.00	12.48	49.49	5.09	17.49	10^{-6}	A			
		rs1675379568:T	cgcgtctggg	G→T	gctcccaccc	104.20	8.16	27.19	3.50	17.85	10^{-6}	A			
		rs1675380129:A	gcgctcccac	C→A	cggccgccgg	104.20	8.16	92.63	7.55	2.08	0.05	D			
		rs1675380331:T	ggcgcgctcc	C→T	ccccggccgc	104.20	8.16	71.43	7.28	5.88	10^{-6}	A			
		rs189324691:A	ggggacttgc	G→A	cccgcgcgtc	104.20	8.16	60.85	4.90	9.58	10^{-6}	A			
		rs189324691:T	ggggacttgc	G→T	cccgcgcgtc	104.20	8.16	87.23	6.83	3.21	10^{-2}	C			
		rs369653302:T	gagccgtcgg	G→T	gccgcgtccc	101.00	7.64	51.04	3.53	13.32	10^{-6}	A			
		rs373204433:T	ccgcgtcccg	G→T	ccccggccca	101.00	7.64	77.02	7.10	4.54	10^{-3}	B			
		rs573555875:A	ccggagccgt	C→A	ccagccgcgt	101.00	7.64	53.19	4.41	11.43	10^{-6}	A			
		rs573555875:T	ccggagccgt	C→T	ccagccgcgt	101.00	7.64	86.00	7.30	2.83	10^{-2}	C			
		rs746005911:A	ggcggcggcc	G→A	gcggcggcgg	101.00	7.64	50.47	4.05	12.58	10^{-6}	A			
		rs746005911:T	ggcggcggcc	G→T	gcggcggcgg	101.00	7.64	62.35	4.30	9.42	10^{-6}	A			
		rs759626271:T	agccgtcggg	C→T	ccgcgtcccg	101.00	7.64	29.43	2.71	20.69	10^{-6}	A			
		rs769974059:T	cggcggcggc	C→T	cgcggcggcg	101.00	7.64	86.82	5.92	2.97	10^{-2}	C			
		rs886686401:A	ccgagcctag	C→A	cgggaccgga	155.00	12.48	64.42	6.45	13.67	10^{-6}	A			
		rs886686401:T	ccgagcctag	C→T	cgggaccgga	155.00	12.48	42.62	3.74	21.67	10^{-6}	A			
		rs959326115:A	gggactcgct	G→A	ggagagcgcg	155.00	12.48	46.36	5.10	17.71	10^{-6}	A			
150	*VDR* (7421)	rs1010157110:G	cctggcactg	A→G	tgccagctgg	45.36	3.97	65.05	4.84	6.28	10^{-6}	A	↓	according to an exhaustive review [427]: Vdr-knockout mice are susceptible to hypertension aggravating POAG [9]	▼
		rs1242305982:G	actggacctg	T→G	ggcaagagag	62.01	4.74	106.29	7.55	10.33	10^{-6}	A			
		rs1297198771:C	ccactgctcc	T→C	gccaccgctt	39.67	3.91	54.49	5.33	4.57	10^{-3}	B			
		rs1304891316:A	ttgtcaaaag	G→A	gctgtctctg	26.24	1.95	29.37	2.08	2.20	0.05	D			
		rs1308784880:C	ggtgctgggc	T→C	aggcggggcc	71.21	5.50	82.96	6.68	2.74	10^{-2}	C			
		rs1592140694:C	tgctcctgct	A→C	ccgcttccca	39.67	3.91	97.60	7.54	14.37	10^{-6}	A			
		rs1592176401:G	agggccaggg	T→G	tggacagcag	16.91	1.59	19.45	1.77	2.14	0.05	D			
		rs1937821528:G	gggtggtgta	A→G	cagcagggcc	16.91	1.59	25.42	3.02	5.38	10^{-6}	A			
		rs1946648015:C	gactggacct	G→C	tggcaagaga	62.01	4.74	91.18	6.94	7.15	10^{-6}	A			
		rs201110362:G	ggccctggca	C→G	ggatgccagc	45.36	3.97	57.10	5.58	3.51	10^{-3}	B			
		rs760012896:G	gccctggcac	T→G	gatgccagct	45.36	3.97	65.05	4.84	6.28	10^{-6}	A			
		rs764297575:T	ccagctggcc	C→T	caccaaggat	45.36	3.97	51.54	4.25	2.13	0.05	D			
		rs770238728:A	ctggccctgg	C→A	aaggatgcca	45.36	3.97	62.03	5.54	5.01	10^{-6}	A			
		rs770238728:G	ctggccctgg	C→G	aaggatgcca	45.36	3.97	59.98	5.57	4.38	10^{-3}	B			
		rs916756491:C	gccagggtgg	T→C	acagcagcag	16.91	1.59	38.14	3.28	12.79	10^{-6}	A			
		rs973674771:C	ctgcttgtca	A→C	ctgggctgtc	26.24	1.95	37.72	2.79	6.93	10^{-6}	A			
		rs982790742:A	ccgcttccca	C→A	ggttgccgcc	39.67	3.91	47.90	5.24	2.56	0.05	D			

Table S3. Cont.

#	Human Gene: NCBI Gene Symbol (NCBI Gene ID)	Candidate SNP marker: dbSNP ID:min [437]	5′ flank, 10 bp	WT →min	3 flank, 10 bp	K_D, nM, *in silico*: WT MEAN	± SEM	min MEAN	± SEM	Significance: Z	p	ϱ	Δ	Effect of changes in human gene expression on the development of primary open-angle glaucoma (POAG, ☼: "▼" aggravation, "▲" alleviation) [Reference]	☼ ▲ ▼
		rs1044509748:A	ggcaagagag	G→A	acggagctcc	62.01	4.74	45.77	3.53	5.59	10^{-6}	A			
		rs1044509748:T	ggcaagagag	G→T	acggagctcc	62.01	4.74	25.10	2.36	14.92	10^{-6}	A			
		rs11574010:A	cctggcaaga	G→A	ccaacggagc	62.01	4.74	45.00	3.06	6.27	10^{-6}	A			
		rs1173278324:T	ctggcactga	C→T	gccagctggc	45.36	3.97	28.90	2.23	7.73	10^{-6}	A			
		rs1183362719:T	ggcggggcct	G→T	ggcggggccg	62.01	4.74	38.40	3.68	7.82	10^{-6}	A			
		rs1229389678:T	cccactgctc	C→T	cgccaccgct	39.67	3.91	22.59	2.17	8.18	10^{-6}	A			
		rs1255133895:A	cactgactct	G→A	gctggccctg	45.36	3.97	31.30	2.15	6.67	10^{-6}	A			
		rs1313856892:T	ggcctgcttg	C→T	gctctgaccg	45.36	3.97	19.55	1.41	14.87	10^{-6}	A			
		rs1337495947:A	tcctgctact	C→A	cttcccactg	39.67	3.91	10.65	1.41	15.89	10^{-6}	A			
		rs1365743261:T	tcccactgct	C→T	ccgccaccgc	39.67	3.91	27.63	2.45	5.45	10^{-6}	A			
		rs1396130056:A	accgtggcct	G→A	ctctggctct	45.36	3.97	27.18	2.41	8.23	10^{-6}	A			
		rs1946083143:A	ccctggcact	G→A	atgccagctg	45.36	3.97	14.16	1.61	16.25	10^{-6}	A			
150	*VDR* (7421)	rs1946083857:A	caccaaggat	G→A	ggggtgggtg	45.36	3.97	27.64	2.79	7.42	10^{-6}	A	↑	within human disease cellular models using a fast-growing mice neuroblastoma cell line Neuro2a transfected with lentiviral vector carrying another Vdr gene copy [428]: amyloid precursor protein gene expression decreases with an increase in Vdr level that can reduce regional beta-amyloid peptide accumulations, which can block axonal transport as an initial step in retinal ganglion cell degeneration aggravating POAG according to human POAG models using mice injected with microbeads to increase intraocular pressure [429]	▲
		rs1946084094:A	gggggtgggt	G→A	gggatgaggc	45.36	3.97	36.82	3.07	3.46	10^{-3}	B			
		rs1946188633:A	cactgctcct	G→A	ccaccgcttc	39.67	3.91	8.07	1.20	17.80	10^{-6}	A			
		rs1946645709:T	gctgggctgt	C→T	cggggccagg	71.21	5.50	30.15	2.37	15.61	10^{-6}	A			
		rs1946645844:A	caggtgctgg	G→A	agaggcgggg	71.21	5.50	53.15	3.96	5.45	10^{-6}	A			
		rs1946648015:A	gactggacct	G→A	tggcaagaga	62.01	4.74	29.05	2.99	11.82	10^{-6}	A			
		rs770238728:T	ctggccctgg	C→T	aaggatgcca	45.36	3.97	23.84	2.17	10.20	10^{-6}	A			
		rs920458052:T	aggtgctggg	C→T	gaggcggggc	71.21	5.50	44.61	3.68	8.28	10^{-6}	A			
		rs954415361:A	ggggcctgac	C→A	ggggccgggg	71.21	5.50	53.96	4.31	4.99	10^{-6}	A			
		rs954415361:T	ggggcctgac	C→T	ggggccgggg	71.21	5.50	61.65	4.42	2.74	10^{-2}	C			
		rs973674771:T	ctgcttgtca	A→T	ctgggctgtc	26.24	1.95	13.90	1.46	9.88	10^{-6}	A			
		rs995966715:T	ctctgcttgt	C→T	tgctgggctg	26.24	1.95	7.24	0.56	23.99	10^{-6}	A			
		rs998478828:A	acccccacac	C→A	gcccagccgc	39.67	3.91	32.31	2.88	3.09	10^{-2}	C			
		rs1022494662:C	gccgggctca	T→C	ggacgggtga	40.80	3.21	64.84	5.09	8.34	10^{-6}	A			
		rs1022494662:G	gccgggctca	T→G	ggacgggtga	40.80	3.21	64.84	5.09	8.34	10^{-6}	A			
		rs1295263390:C	gggaggaaga	G→C	tagctcgccg	42.77	3.12	50.82	3.97	3.22	10^{-2}	C			
		rs1341479209:G	ggaggaagag	T→G	agctcgccga	42.77	3.12	99.70	7.18	16.51	10^{-6}	A			
		rs1365612606:T	tttttttttt	A→T	aaagtcggct	4.49	0.39	5.09	0.40	2.12	0.05	D			
		rs1426314405:A	gccgattaca	T→A	cagcccgggc	20.97	1.65	23.64	1.69	2.26	0.05	D			
		rs1460226948:G	agccgggctc	A→G	tggacgggtg	40.80	3.21	64.84	5.09	8.34	10^{-6}	A			
151	*VEGFA* (7422)	rs1582461911:G	gccggggagg	A→G	agagtagctc	42.77	3.12	57.45	4.59	5.45	10^{-6}	A	↓	within human POAG models using rats injected with streptozotocin injection until diabetic symptoms observed, and, next, transfected with microRNA-15b targeted Vegfa into retinal capillary endothelial cells [430]: *Vegfa* downregulation along with reduced hyperproliferation of retinal capillary endothelial cells that can alleviate retinopathy in POAG [198]	▲
		rs1582479196:G	ccttgctctc	T→G	ttctgtcctc	27.95	2.01	36.94	2.65	5.50	10^{-6}	A			
		rs1763181606:C	cctttttttt	T→C	taaaagtcgg	4.49	0.39	7.54	0.68	8.25	10^{-6}	A			
		rs529911267:G	ttttttttta	A→G	aagtcggctg	4.49	0.39	6.74	0.66	6.14	10^{-6}	A			
		rs562785377:C	cttttttttt	T→C	aaaagtcggc	4.49	0.39	16.05	1.20	22.04	10^{-6}	A			
		rs752674861:G	ggctcatgga	C→G	gggtgaggcg	40.80	3.21	63.65	4.81	8.16	10^{-6}	A			
		rs752674861:T	ggctcatgga	C→T	gggtgaggcg	40.80	3.21	53.63	3.46	5.38	10^{-6}	A			
		rs772365349:G	cgccgaggag	A→G	gcgggccgcc	141.01	10.67	162.72	13.19	2.58	10^{-2}	C			

Table S3. Cont.

Human Gene		Candidate SNP marker				K_D, nM, *in silico*				Significance				Effect of changes in human gene expression on the development of primary open-angle glaucoma (POAG, ☼: "▼" aggravation, "▲" alleviation) [Reference]	☼ ▲ ▼
						WT		min							
#	*NCBI Gene Symbol* (NCBI Gene ID)	dbSNP ID:min [437]	5′ flank, 10 bp	WT →min	3 flank, 10 bp	MEAN	± SEM	MEAN	± SEM	Z	p	ϱ	Δ		
151	*VEGFA* (7422)	rs1001797852:T	tttctgtcct	C→T	agtggtccca	27.95	2.01	18.53	1.42	7.84	10^{-6}	A	↑	within human hypoxia eye adaptation models using mice exposed to either short- (6 hours), long- (48 hours) terms or chronic (11 weeks) normobaric hypoxia at 400 m or hypobaric hypoxia at 3450 m above sea level [431]: *Vegfa* upregulation at 5 of 6 statistically significant cases, with the only exception of a near-threshold trend in the case of chronic normobaric hypoxia, which overall indicates an attempt to improve oxygen delivery to both the optic nerve and retinal ganglion cells by increasing choroidal circulation, which may be something like neuroprotection supporting their survival as the alleviation of POAG	▲
		rs1010547230:T	gagggggccg	C→T	agtggcgact	40.80	3.21	34.88	3.88	2.30	0.05	D			
		rs1026258194:T	ttacatcagc	C→T	cgggcctggc	20.97	1.65	14.60	1.47	5.67	10^{-6}	A			
		rs1027989270:T	cagacagaca	C→T	cgcccccagc	37.65	2.83	34.00	2.07	2.11	0.05	D			
		rs1056039372:T	gagccgatta	C→T	atcagcccgg	20.97	1.65	11.00	1.09	10.22	10^{-6}	A			
		rs1177711361:T	gccggcggcg	G→T	acagtggacg	37.65	2.83	20.16	2.05	9.90	10^{-6}	A			
		rs1203752241:T	tcgccgaggc	G→T	ccgaggagag	141.01	10.67	125.60	9.53	2.16	0.05	D			
		rs1333424932:T	gctcatggac	G→T	ggtgaggcgg	40.80	3.21	30.08	2.37	5.48	10^{-6}	A			
		rs1388776124:T	aggaagaaga	G→T	aaggaagagg	40.80	3.21	14.68	1.19	18.09	10^{-6}	A			
		rs1396039360:A	cgggctcatg	G→A	acgggtgagg	40.80	3.21	20.17	1.44	13.27	10^{-6}	A			
		rs1412348943:T	cgccccacag	C→T	ccgagccgga	141.01	10.67	85.32	5.50	10.11	10^{-6}	A			
		rs1482267742:T	tctttctgtc	C→T	tcagtggtcc	27.95	2.01	24.46	1.74	2.64	10^{-2}	C			
		rs1561980368:A	ctcatggacg	G→A	gtgaggcggc	40.80	3.21	29.08	2.16	6.25	10^{-6}	A			
		rs1561980368:C	ctcatggacg	G→C	gtgaggcggc	40.80	3.21	32.65	2.57	4.01	10^{-3}	B			
		rs1582460556:A	agaagagaag	G→A	aagaggagag	40.80	3.21	33.35	2.27	3.87	10^{-3}	B			
		rs1582461193:T	tcatggacgg	G→T	tgaggcggcg	40.80	3.21	32.43	2.49	4.17	10^{-3}	B			
		rs1582462158:A	gccgaggcgc	C→A	gaggagagcg	141.01	10.67	119.92	9.01	3.04	10^{-2}	C			
		rs1582462228:A	gaggcgccga	G→A	gagagcgggc	141.01	10.67	108.27	8.10	4.97	10^{-6}	A			
		rs1763269227:A	gacagacaga	C→A	agacaccgcc	37.65	2.83	21.11	1.44	11.42	10^{-6}	A			
		rs1763393050:C	ccggggagga	A→C	gagtagctcg	42.77	3.12	30.00	2.37	6.60	10^{-6}	A			
		rs1763758740:G	ccgattacat	C→G	agcccgggcc	20.97	1.65	15.98	1.22	4.96	10^{-6}	A			
		rs1764608860:G	ctctttctgt	C→G	ctcagtggtc	27.95	2.01	25.14	1.82	2.08	0.05	D			
		rs370995111:A	cctccccctt	G→A	ggatcccgca	59.87	3.87	52.63	5.05	2.23	0.05	D			
		rs372731987:A	agcccgagcc	G→A	gagagggagc	141.01	10.67	89.15	6.80	8.54	10^{-6}	A			
		rs372731987:T	agcccgagcc	G→T	gagagggagc	141.01	10.67	107.83	8.14	5.02	10^{-6}	A			
		rs6928818:A	tgagtgacct	G→A	cttttggggg	59.87	3.87	18.08	1.93	19.17	10^{-6}	A			
		rs745387465:T	aggaagagta	G→T	ctcgccgagg	42.77	3.12	23.46	2.17	10.20	10^{-6}	A			
		rs752729944:T	ccccacagcc	C→T	gagccggaga	141.01	10.67	117.69	9.29	3.31	10^{-3}	B			
		rs754042549:T	gcgggccgcc	C→T	cacagcccga	141.01	10.67	93.71	7.89	7.22	10^{-6}	A			
		rs759754009:T	ggccgcccca	C→T	agcccgagcc	141.01	10.67	57.80	5.36	14.90	10^{-6}	A			
		rs762362569:T	gcgccgagga	G→T	agcgggccgc	141.01	10.67	53.69	5.18	15.75	10^{-6}	A			
		rs765392517:A	ccgccccaca	G→A	cccgagccgg	141.01	10.67	64.38	4.60	15.07	10^{-6}	A			
		rs765392517:C	ccgccccaca	G→C	cccgagccgg	141.01	10.67	117.17	9.52	3.34	10^{-3}	B			
		rs772861763:T	ccccagctac	C→T	acctcctccc	37.65	2.83	13.64	1.64	14.33	10^{-6}	A			
		rs774898653:A	ccgaggcgcc	G→A	aggagagcgg	141.01	10.67	126.48	9.36	2.06	0.05	D			
		rs774898653:C	ccgaggcgcc	G→C	aggagagcgg	141.01	10.67	125.06	9.51	2.24	0.05	D			
		rs777531850:A	gccggagagg	G→A	agcgcgagcc	141.01	10.67	126.62	9.45	2.03	0.05	D			
		rs779923978:A	cagacagaca	G→A	acaccgcccc	37.65	2.83	18.95	1.52	12.48	10^{-6}	A			
		rs779923978:C	cagacagaca	G→C	acaccgcccc	37.65	2.83	31.55	2.24	3.42	10^{-3}	B			
		rs780766978:A	cggggaggaa	G→A	agtagctcgc	42.77	3.12	22.35	1.72	12.24	10^{-6}	A			
		rs781745747:G	tgctctcttt	C→G	tgtcctcagt	27.95	2.01	16.98	1.25	9.68	10^{-6}	A			
		rs897547135:T	cgccgaggcg	C→T	cgaggagagc	141.01	10.67	89.83	5.93	8.98	10^{-6}	A			
		rs904926409:A	ggcaaagtga	G→A	tgacctgctt	59.87	3.87	45.26	3.20	5.84	10^{-6}	A			
		rs942729487:A	cccagcccca	G→A	ctaccacctc	37.65	2.83	19.43	1.84	10.93	10^{-6}	A			
		rs992466179:A	gcggcgcgga	G→A	ccgattacat	20.97	1.65	15.46	1.17	5.58	10^{-6}	A			

Table S3. Cont.

Human Gene		Candidate SNP marker				K_D, nM, *in silico*				Significance				Effect of changes in human gene expression on the development of primary open-angle glaucoma (POAG, ☼: "▼" aggravation, "▲" alleviation) [Reference]	☼ ▲ ▼
						WT		min							
#	*NCBI Gene Symbol* (NCBI Gene ID)	dbSNP ID:min [437]	5′ flank, 10 bp	WT →min	3 flank, 10 bp	MEAN	± SEM	MEAN	± SEM	Z	p	ϱ	Δ		
152	*WDR36* (134430)	rs1004959524:G	agggagaact	T→G	tatgtgttgc	6.02	0.62	7.61	0.80	3.19	10^{-2}	C	↓	within human POAG cellular models using yeast expressing test-system with yeast WDR38-homologous gene Utp21 carrying WDR38-allele associated with POAG [432]: WDR38 deficiency seems to be a promising candidate biomedical molecular marker for POAG in human	▼
		rs1248054645:C	gagaacttta	T→C	gtgttgcact	6.02	0.62	11.51	1.45	7.98	10^{-6}	A			
		rs76232292:A	tctggaggga	G→A	aactttatgt	6.02	0.62	7.61	0.62	3.59	10^{-3}	B			
		rs765826586:G	ggagaacttt	A→G	tgtgttgcac	6.02	0.62	15.91	1.21	15.25	10^{-6}	A			
		rs765826586:T	ggagaacttt	A→T	tgtgttgcac	6.02	0.62	14.72	1.00	14.58	10^{-6}	A			
		rs1064796838:A	aactttatgt	G→A	ttgcactgag	6.02	0.62	3.91	0.35	6.34	10^{-6}	A	↑	within human POAG models using transgenic heterozygotous Wdr36-deficient glaucomatous mice overexpressing *Wdr36* [433]: Wdr36 excess along absence of POAG symptoms, which may argue for rather than against the protective properties of excess WDR36 against POAG	▲
		rs1328773508:A	agaactttat	G→A	tgttgcactg	6.02	0.62	2.92	0.40	8.49	10^{-6}	A			
		rs1752873484:A	tttatgtgtt	G→A	cactgagggc	6.02	0.62	5.15	0.39	2.46	0.05	D			
		rs1752873524:T	ttatgtgttg	C→T	actgagggca	6.02	0.62	5.21	0.44	2.18	0.05	D			
		rs370426254:T	ggagggagaa	C→T	tttatgtgtt	6.02	0.62	5.12	0.44	2.44	0.05	D			
		rs767268039:T	ctggagggag	A→T	actttatgtg	6.02	0.62	5.00	0.40	2.88	10^{-2}	C			
153	*XRCC1* (7515)	rs1404415707:C	cggggtttga	A→C	cgccggcgcg	19.30	1.39	31.98	2.83	8.84	10^{-6}	A	↓	within human disease models using murine neural-specific inactivation of Xrcc1 [434]: susceptibility to neurodegenerative diseases, one of which is POAG [224]	▼
		rs1972851333:G	cgcgccgggg	T→G	gccggcgccg	19.30	1.39	33.41	2.40	10.77	10^{-6}	A			
		rs1972851378:A	gcgcgccggg	G→A	cgccggcgcc	19.30	1.39	22.00	1.65	2.51	0.05	D			
		rs568542703:C	cgccggggtt	T→C	cggcgccggc	19.30	1.39	39.74	2.86	14.16	10^{-6}	A			
		rs1027871636:A	ccatgccttc	G→A	tatggccacc	31.25	2.77	25.60	1.71	3.59	10^{-3}	B	↑	within a biomedical cohort-based study using the tissue microarray technology [435]: XRCC1 excess is a biomedical molecular marker of the healthy glia cells adjacent the glioma cells because it exerts potent inhibitory effects on neovascularization, suppresses the tumorigenic behavior of glioma cells, and protects neural cells just as tetramethylpyrazine does, being herbal medication from *Ligusticum striatum* (Chuan Xiong) root within traditional Chinese medicine against both glioma and glaucoma [436]	▲
		rs1027871636:C	ccatgccttc	G→C	tatggccacc	31.25	2.77	27.74	1.83	2.16	0.05	D			
		rs1213741890:T	cgcgcttgcg	C→T	cccgagcctc	19.30	1.39	8.06	0.90	13.16	10^{-6}	A			
		rs1281862650:T	cgcttgcgca	C→T	cgagcctcgc	19.30	1.39	17.21	1.31	2.19	0.05	D			
		rs1349789446:A	ggcgcgccgg	G→A	acgccggcgc	19.30	1.39	15.21	1.07	4.72	10^{-3}	B			
		rs1421294424:T	ttccagcctc	C→T	gaaatggtct	31.25	2.77	27.42	2.33	2.13	0.05	D			
		rs1460939617:A	gtagtctctt	C→A	ccgcccaccc	31.25	2.77	12.68	1.03	14.99	10^{-6}	A			
		rs1972917120:A	cgcccacccc	G→A	ccgccaaccc	31.25	2.77	25.80	2.03	3.23	10^{-2}	C			
		rs529821257:A	ccggcgcgcc	G→A	cgacgccggc	19.30	1.39	17.07	1.17	2.47	0.05	D			
		rs568542703:A	cgccggggtt	T→A	cggcgccggc	19.30	1.39	15.19	1.41	4.07	10^{-3}	B			
		rs569891910:T	cccatgcctt	C→T	ctatggccac	31.25	2.77	22.33	1.68	5.78	10^{-6}	A			
		rs890174313:A	tccaatgaac	C→A	ccgtagtctc	31.25	2.77	27.78	2.00	2.06	0.05	D			

Table S4. Verification of the i*n silico* estimates of changes in TBP affinity for the minor (min) and the ancestral (WT) alleles of SNPs in human genes promoters taken from the Human_SNP_TATAdb knowledge base [49] against previously published experimental data on the same changes obtained *in vitro* by the electrophoretic mobility shift assay (EMSA), including rs1143627:T in the human gene IL1B, a clinically proven SNP marker for susceptibility to POAG in Brazil according to a cohort-based original research article [40].

**Human Gene**		**Candidate SNP marker**				**Verification**		
#	*NCBI Gene Symbol* (NCBI Gene ID)	**dbSNP ID:min [437]**	**5′ flank, 10 bp**	**WT→min**	**3 flank, 10 bp**	*in silico*	*in vitro*	
						**$K_{D;min}$/ $K_{D;WT}$**	**$K_{D;min}$/ $K_{D;WT}$**	**Reference**
1	*IL1B* (3553)	rs1143627:T	ttttgaaagc	C→T	ataaaaacag	0.39	0.24	[438]
2	*ABCA9* (438)	rs367781716:C	caattatttg	T→C	atatttctga	2.41	2.41	[439]
3	*ASMT* (438)	rs1402972626:A	gacctttttgt	G→A	cccagaatag	0.47	1.67	[440]
4	*CDY2A* (9426)	rs20067072:C	atgttccata	T→C	aatcgtcata	1.67	3.13	[440]
5	*F3* (2152)	rs563763767:T	ccctttatag	C→T	gcgcggggca	0.66	0.36	[438]
6	*F9* (2158)	rs750827465:A	tttggtacaa	C→A	taatcgacct	0.63	0.31	[441]
7	*GCG* (2641)	rs183433761:G	gctggagagt	A→G	tataaaagca	1.84	2.25	[442]
8	*GTPBP6* (8225)	rs1393008234:T	acgagcacgt	G→T	atgaggagcg	0.25	0.93	[440]
9	*MBL2* (4153)	rs72661131:C	tctatttcta	T→C	atagcctgca	2.39	1.60	[438]
10	*SHOX* (6473)	rs1452787381:G	gtcgccgcgt	A→G	taaatagtga	3.00	6.69	[440]
11	*ZFY* (7544)	rs1452787381:T	ggagggggcc	C→T	aactaccatc	0.60	2.01	[438]
**Criteria of correlation analysis (*statistical significance*)**		Pearson's linear correlation, r				0.76 ($p < 0.01$)		
		Spearman's rank correlation, R				0.67 ($p < 0.025$)		
		Kendall's rank correlation, τ				0.53 ($p < 0.025$)		
		Goodman–Kruskal generalized correlation, γ				0.53 ($p < 0.025$)		
		Pearson's chi-squared statistics, χ^2				5.24 ($p < 0.025$)		

Genes: *IL1B*, interleukin 1 beta; *ABCA9,* ATP binding cassette subfamily a member 9; *ASMT,* acetylserotonin O-methyltransferase; *CDY2A,* chromodomain Y-linked 2A; *F3,* coagulation factor III, tissue factor; *F9,* coagulation factor IX; *GCG,* Glucagon; *GTPBP6,* GTP Binding Protein 6; *MBL2,* mannose binding lectin 2; *SHOX,* SHOX homeobox; *ZFY,* zinc finger protein Y-linked.

Table S5. Comparison between the biomedically proven SNP markers for human diseases according to the ClinVar database [38] (access on 10 July 2024) and their annotation according our *in silico* analysis

Human Gene		Candidate SNP marker				K_D, nM, *in silico*				Significance				Effect of changes in human gene expression on the development of primary open-angle glaucoma (POAG, ☼: "▼" aggravation, "▲" alleviation) [Reference]	☼ ▲ ▼
						WT		min							
#	*NCBI Gene Symbol* (NCBI Gene ID)	dbSNP ID:min [437]	5′ flank, 10 bp	WT →min	3 flank, 10 bp	MEAN±	SEM	MEAN±	SEM	Z	p	ϱ	Δ		
1	*ABCA1* (19)	rs886063317:C	agccgaatct	A→C	gcgctcggtg	2.23	0.22	9.87	0.90	21.9	10^{-6}	A	↓	within human disease models using Abca1-null mice [76]: accelerated vision loss can worsen POAG	▼
		rs886063317:G	agccgaatct	A→G	gcgctcggtg	2.23	0.22	7.05	0.66	16.69	10^{-6}	A		The ClinVar database [38] documents that both minor alleles "C" and "G" of the SNP rs886063317 as two clinically proven molecular SNP markers for familial high density lipoprotein deficiency (Tangier disease, hypoalphalipoproteinemia), which is comorbid for POAG according to a comprehensive review [443]	
2	*ABCB1* *(5243)*	rs1584915287:A	ggctgggcag	G→A	tgaggctgat	56.75	3.86	33.96	2.58	10.08	10^{-6}	A	↑	within a cohort-based biomedical study [80]: the increasing level of ABCB1 expression with an increase in the duration of drug treatment may contribute to the molecular mechanisms of the emergence and progression of multidrug resistance, which may delay POAG treatment<br>The ClinVar database [38] documents that the only minor allele "A" of the SNP rs1584915287 as a clinically proven molecular SNP marker for Tramadol response that is consistent with a case report [444] about a healthy man without past ocular history, who, after administering Tramadol as a painkiller for a hip fracture, experienced an acute ocular hypertension attack with glaucomatous vision impairment in both eyes	▼
3	*ATXN2* (6311)	rs695871:G	cttcgtcgtc	C→G	cctccccgcc	62.03	4.38	38.80	2.69	9.48	10^{-6}	A	↑	within human neurodegenerative disease models using Atxn2-overexpressing mice [116]: neurodegeneration, which can aggravate POAG [117]<br>The ClinVar database [38] documents that the only minor allele "G" of the SNP rs695871 as a clinically proven molecular SNP marker for Spinocerebellar ataxia type 2 that is consistent with cohort-based clinical observations of a near-threshold trend toward glaucomatous changes in the architecture of both retina and optic nerve head in patients in Spinocerebellar ataxia type 2 compared with both normal and Spinocerebellar ataxia of other types [445]	▼
4	*BMP4* (652)	rs774069849:A	ctggtcttga	G→A	gatccacagc	20.26	1.76	14.62	1.23	5.39	10^{-6}	A	↑	according to a cohort-based biomedical study [123]: BMP4 excess is a biomedically proven molecular marker for corneal dystrophy comorbid to POAG [124]<br>the ClinVar database [38] documents that the only minor allele "A" of the SNP rs774069849 as a clinically proven molecular SNP marker for both orofacial cleft and microphthalmia with brain and digit anomalies as morpho-ontogenetic disorders of the human head development, which are inevitably leading to developmental disorders of the eyes as parts of the head colocalized along with all its other parts under stringent spatiotemporal control that can phenotypically manifest as congenital, early-onset, pediatric and juvenile forms of various glaucomas, including POAG [446]	▼

Table S5. Cont.

#	Human Gene: *NCBI Gene Symbol* (NCBI Gene ID)	Candidate SNP marker: dbSNP ID:min [437]	5′ flank, 10 bp	WT →min	3 flank, 10 bp	K_D, nM, *in silico*: WT MEAN	± SEM	min MEAN	± SEM	Significance: Z	p	ϱ	Δ	Effect of changes in human gene expression on the development of primary open-angle glaucoma (POAG, ☼: "▼" aggravation, "▲" alleviation) [Reference]	☼ ▲ ▼
5	*CP* (1356)	rs151304828:T	ctctcactta	C→T	tttaggtgaa	4.37	0.52	2.69	0.27	6.22	10^{-6}	A	↑	within nonhuman primate POAG models using male juvenile cynomolgus monkeys (*Macaca fascicularis*) treated with an argon laser to scar the trabecular meshwork in one eye until an intraocular pressure increased indicating glaucomatous eye [161]: ceruloplasmin excess in the retina that may be needed as part of antioxidant system, which must counteract the inevitable damaging effects of free radicals on the head of the optic nerve when perceiving light flux as a vital function of the eyes<br>the ClinVar database [38] documents that the only minor allele "T" of the SNP rs151304828 as a clinically observed molecular SNP marker of the "Conflicting Pathogenicity Classifications" type within the terms of the standards/guidelines for the interpretation of sequence variants made jointly consensusly by the American College of Medical Genetics and Genomics and the Association for Molecular Pathology [447], which characterizes ferroxidase (ceruloplasmin) deficiency within susceptibility screening in healthy volunteers, and after a comprehensive automated analysis of all freely available literature and factual sources of clinical information, this allele was classified as "variant of unknown significance for this disease", whereas using our public available Web service SNP_TATA_Comparator [44] we have in silico estimated in opposite that this allele may on cause an excess of ferroxidase (ceruloplasmin) contributing to a protective mechanism in the retina against neuridegenerative oxidative stress in POAG [448]	▲
6	*ELN* (2006)	rs41410045:G	ggggataaaa	C→G	gaggtgcgga	21.68	1.72	16.92	1.34	4.41	10^{-3}	B	↑	within a cohort biomedical qPCR study [192]: ELN upregulation is a biomedical molecular marker for POAG<br>the ClinVar database [38] documents that two minor alleles "G" and "T" of the SNP rs41410045 along with two minor alleles "A" and "T" of another SNP rs537200597 as four clinically proven molecular SNP marker sfor both supravalvular aortic stenosis and cutis laxa, which are hereditary connective tissue disorders capable to manifest in morpho-ontogenetic eye defects such as a subluxation lens leading to POAG [449]	▼
		rs41410045:T	ggggataaaa	C→T	gaggtgcgga	21.68	1.72	18.43	1.46	2.89	10^{-2}	C			
		rs537200597:A (transcript ELN-217)	cctcacagcc	G→A	acgaggcaac	78.15	5.41	66.54	5.15	3.10	10^{-2}	C			
		rs537200597:T (transcript ELN-206)	cctcacagcc	G→T	acgaggcaac	47.57	3.27	41.15	3.37	2.71	10^{-2}	C			
7	*FAS* (355)	rs558072404:A	tgagccctct	C→A	ctgcccgggt	41.25	3.48	29.95	2.70	5.19	10^{-6}	A	↑	within human disease model using mice [200]: Fas-activation induced retinal ganglion cell apoptosis, glial activation, and inflammation as a complication of POAG<br>the ClinVar database [38] documents that the only minor allele "A" of the SNP rs558072404 as a clinically proven molecular SNP marker for autoimmune lymphoproliferative syndrome type 1 as a form of the lymphoproliferative disorder, which can be infection-related post-traumatic and \or post-surgery complications leading morphological changes in eyes that can elevate intraocular pressure aggravating POAG [450]	▼
8	*GRIN2B* (2904)	rs797044930:T	gccttctttg	C→T	tgtcagtgtg	26.41	2.61	17.92	1.12	6.62	10^{-6}	A	↑	within human disease models using mice [217]: Grin2b excess can enhance glutamatergic signaling, which can elevate excitotoxicity accelerating neurodegeneration in POAG<br>the ClinVar database [38] documents that the only minor allele "T" of the SNP rs797044930 as a clinically proven molecular SNP marker for intellectual disability as inborn genetic disease GRIN2B encephalopathy that according to a cohort-based clinical study [451], along with previously known epilepsy, autism and intellectual disability, can also manifest with movement disorder, cortical development malformation and cortical visual impairment, which is comorbid to POAG within a comprehensive populational review on pediatric visual impairment diagnoses in Barbados [452]	▼

Table S5. Cont.

#	Human Gene: *NCBI Gene Symbol* (NCBI Gene ID)	Human Gene: dbSNP ID:min [437]	Candidate SNP marker: 5′ flank, 10 bp	Candidate SNP marker: WT→min	Candidate SNP marker: 3 flank, 10 bp	K_D, nM, *in silico*: WT MEAN	± SEM	K_D, nM, *in silico*: min MEAN	± SEM	Significance: Z	p	ϱ	Δ	Effect of changes in human gene expression on the development of primary open-angle glaucoma (POAG, ☼: "▼" aggravation, "▲" alleviation) [Reference]	☼ ▲ ▼
														within human POAG cellular models using human trabecular meshwork cell line TM-1 transfected with plasmid vector carrying mutant glaucomatous variants of MYOC gene [249]: IL1B upregulation as a candidate molecular marker for POAG	
9	*IL1B* (3553)	rs1143627:T	ttttgaaagc	C→T	cctacttctg	4.50	0.39	1.76	0.17	14.56	10^{-6}	A	↑	the ClinVar database [38] documents that the only minor allele "T" of the SNP rs1143627 as a clinically proven molecular SNP marker for resistance to (i) pharyngeal squamous cell carcinoma [453] increasing risks of POAG [454]; (ii) severe hemophilia A (within a haplotype) [455] elevating intraocular pressure in POAG [456]; (iii) low-dose aspirin-induced peptic ulcers [457] aggravating POAG [458]; (iv) colorectal cancer (within a haplotype) [459] worsening POAG [460]; (v) dengue shock syndrome [461] contributing POAG [462]; (vi) intracranial hemorrhage [463] increasing intraocular pressure in POAG [464], (vii) brain arteriovenous malformation [463] aggravating POAG [465], besides (viii) ulcerative colitis [466], (ix) cardiomyopathy [467], and (x) duodenal ulcer after Helicobacter pylori [468] elevating POAG risks according to a comprehensive review [469] along with the cohort-based clinical studies [470] and [471], respectively, as well as susceptibility to many other disorders, namely: (1) gastric cancer at Helicobacter pylori [472] comorbid with POAG [473]; (2) higher vulnerability to persistent Helicobacter pylori infection in smokers and who abuses alcohol or eats fruit less than four times a week [474], all of which contribute to POAG [12, 475]; (3) tuberculosis [476] comorbid with POAG [477]; (4) precancerous lesions along with their transformation into cervical [478], breast [479], lung [480] cancers, hepatocellular carcinoma [481], or glioma [483], also hypertension and preeclampsia [482], pulmonary fibrosis [484], inflammatory bowel disease [485], diabetes mellitus [486], ischemic stroke [487], atherosclerosis [488] and coronary artery disease [489], all of which along with POAG have therr common clinical molecular marker as long non-coding RNA CDKN2B antisense RNA 1[237]; (5) obesity and age-related diseases [490] correlating positively with POAG [115]; (6) COVID-19 infection disease [491] aggravating POAG [309]; (7) at Helicobacter pylori, both chronic gastritis and gastric ulcer [492], promising natural phytomedications against which and POAG is an extract from as the phytosymbiotic fungus Aspergillus nidulans separated from coral jasmine (Nyctanthes arbortristis) [493]; (8) depressions [494] diagnosed by glaucomatous retinal changes [495]; (9) Graves' disease [496] elevating intraocular pressure in POAG [496]; (10) preterm labor [497] elevating risks ofPOAG [498]; and (10) POAG [40]; - and, also, for treatment resistance in psoriasis [499] contribiuting POAG [500]; IgA nephropathy [501], treatment of which using steroids can contribute to POAG [502]; osteomyelitis [503], orbital form of which aggravates POAG [504]; Kawasaki disease [505] with glaucomatous effects [506]; keratoconus [507] comorbid with POAG [508]; rheumatoid arthritis [509] aggravating POAG [510]; pouchitis [511], for which infliximab is an anti-inflammatory drug in POAG [512]; dental caries and gingivitis [513] while the number of natural teeth can negatively correlate with POAG [514]; osteoporosis [515], which, along with POAG, can be alleviated due to postmenopausal hormonal therapy [516]; lymphoma [517], while methotrexate as lymphoma-modifying drug can cause ischemic optic neuropathy in POAG [518]; epilepsy [519], while the anti-epileptic drug topiramate contributes POAG [520]; influenza A virus infection [521], while *Aloe emodin* as a natural ingredient of Chinese herbs, such as *Cassia occidentalis*, *Rheum palmatum* L., *Aloe vera*, and *Polygonum multiflorum* Thunb, is a phytomedication against both influenza A virus and POAG [522]; periodontitis [523] contributing POAG [524]; osteonecrosis [525], high-dose corticosteroid therapy against which may complicate POAG [526]; ocular toxoplasmosis (within a haplotype) [527] contribute with POAG [528]; liver fibrosis at hepatitis B virus [529] aggravating POAG within liver-eye axis [530]; fatigue with long daytime napping [531] may indicate POAG [532]; schizophrenia [533], in which thinning of the retinal nerve fiber layer worsening POAG [534]; in smokers, high-normal hemoglobin A1C [535] can increased intraocular pressure in POAG [536]; sleep disturbances in Alzheimer's disease [537], while snoring, excessive daytime sleepiness, insomnia and sleep-disordered breathing are associated with POAG [538]; prostate cancer [539] comorbid with POAG [540]; longer survival in primary plasma cell leukemia [541], steroid therapy of which can provoke POAG [542]; a promising drug AC-1202 against Alzheimer's disease [543] can decrease intraocular pressure alleviating POAG [544]; cardiovascular health in African Americans [545], whose predisposed to POAG [546] in line with the clinical fact that individuals with POAG who lead an unhealthy lifestyle have the worst cardiovascular health [547]; sunburns up to basal cell skin carcinoma [548] contributing POAG [549]; severe inflammation in Caucasians [550], whose risks of POAG is minimal among all ethnicities [551], whereas POAG severity increases with an increase in inflammation [552]; ankylosing spondylitis [553] contributing to POAG [554]	▼

Table S5. Cont.

#	Human Gene: *NCBI Gene Symbol* (NCBI Gene ID)	Candidate SNP marker: dbSNP ID:min [437]	5′ flank, 10 bp	WT →min	3 flank, 10 bp	K_D, nM, *in silico*: WT MEAN	± SEM	min MEAN	± SEM	Significance: Z	p	ϱ	Δ	Effect of changes in human gene expression on the development of primary open-angle glaucoma (POAG, ☼: "▼" aggravation, "▲" alleviation) [Reference]	☼ ▲ ▼
10	*LDLR* (3949)	rs969658891:A	ggggcttgtg	T→A	acgagcgggg	7.02	0.87	25.60	2.20	17.11	10^{-6}	A		within human disease models using Ldlr-knockout mice [263]: susceptibility to accelerated atherogenesis, which can aggravate POAG [143]	▼
		rs1357531646:G	aatgctgtaa	A→G	tgacgtgggc	7.43	0.70	9.05	0.89	2.90	10^{-2}	C			
		rs1568582310:C	attgaaatgc	T→C	gtaaatgacg	7.43	0.70	12.72	1.17	8.18	10^{-6}	A			
		rs747068848:C	atgctgtaaa	T→C	gacgtgggcc	7.43	0.70	11.55	1.14	6.47	10^{-6}	A			
		rs879254501:C	ctctcggcag	T→C	tcgtctgtga	37.32	2.63	46.22	3.55	4.11	10^{-3}	B			
		rs879254502:C	tctcggcagt	T→C	cgtctgtgac	37.32	2.63	52.42	4.05	6.50	10^{-6}	A	↓	the ClinVar database [38] documents that the minor alleles of the SNPs rs969658891:A, rs1357531646:G, rs1568582310:C, rs747068848:C, rs879254501:C, rs879254502:C, rs879254503:G, rs879254505:C, rs879254506:C, rs879254507:G, and rs879254511:C as eleven clinically proven molecular SNP markers for susceptibility to familial hypercholesterolemia aggravating POAG [313]	
		rs879254503:G	cggcagttcg	T→G	ctgtgactca	37.32	2.63	53.51	4.50	6.58	10^{-6}	A			
		rs879254505:C	gcagttcgtc	T→C	gtgactcaga	37.32	2.63	53.51	4.50	6.58	10^{-6}	A			
		rs879254506:C	cagttcgtct	G→C	tgactcagac	37.32	2.63	46.85	4.56	3.79	10^{-3}	B			
		rs879254507:G	ttcgtctgtg	A→G	ctcagaccgg	37.32	2.63	44.83	3.15	3.69	10^{-3}	B			
		rs879254511:C	ctgtgactca	G→C	accgggactg	37.32	2.63	41.42	3.00	2.06	0.05	D			
		rs121908042:A	cccaagacgt	G→A	ctcccaggac	37.32	2.63	18.48	1.90	11.30	10^{-6}	A		within a biomedical cohort-based study [264]: LDLR excess is a biomedically proven molecular marker for pterygium occurrence and progression, which blocks light exposure from the cornea to the retina and, thus, may reduce retinal activity and metabolic demands leading to a decreased vascular density in fundus that altogether can aggravate POAG [265]	
		rs193922571:A	gatgggaagt	G→A	catctctcgg	37.32	2.63	9.21	1.07	20.68	10^{-6}	A			
		rs201102461:A	gacgagtttc	G→A	ctgccacgat	37.32	2.63	30.01	2.06	4.44	10^{-3}	B			
		rs2077073153:T	gacggggctt	G→T	tgtacgagcg	7.02	0.87	4.19	0.42	6.46	10^{-6}	A			
		rs2077269298:A	gttcgtctgt	G→A	actcagaccg	37.32	2.63	13.17	1.41	16.30	10^{-6}	A			
		rs730882080:T	tcgctgccac	G→T	atgggaagtg	37.32	2.63	21.75	2.17	8.84	10^{-6}	A			
		rs730882081:G	tgtgactcag	A→G	ccgggactgc	37.32	2.63	33.31	2.41	2.25	0.05	D		the ClinVar database [38] documents that the minor alleles of the SNPs rs121908042:A, .rs193922571:A, rs201102461:A, rs2077073153:T, rs2077269298:A, rs730882080:T, rs730882081:G, ,rs762139262:T, rs769383881:A, rs769383881:T, rs869320648:A, rs875989899:T, rs879254486:T, rs879254502:A, and rs879254506:A as fifteen clinically proven molecular SNP markers for susceptibility to both familial hypercholesterolemia and pathogenic cardiovascular phenotype, which can aggravate POAG [555] and occur in those with POAG more often than without glaucoma [313], respectively	
		rs762139262:T	ctcggcagtt	C→T	gtctgtgact	37.32	2.63	23.19	2.37	7.67	10^{-6}	A	↑		
		rs769383881:A	ctcccaggac	G→A	agtttcgctg	37.32	2.63	26.34	1.99	6.76	10^{-6}	A			
		rs769383881:T	ctcccaggac	G→T	agtttcgctg	37.32	2.63	19.74	1.89	10.73	10^{-6}	A			
		rs869320648:A	ctgccacgat	G→A	ggaagtgcat	37.32	2.63	21.22	1.73	10.50	10^{-6}	A			
		rs875989899:T	catctctcgg	C→T	agttcgtctg	37.32	2.63	26.69	2.05	6.44	10^{-6}	A			
		rs879254486:T	gtttcgctgc	C→T	acgatgggaa	37.32	2.63	25.82	2.32	6.47	10^{-6}	A			
		rs879254502:A	tctcggcagt	T→A	cgtctgtgac	37.32	2.63	13.23	1.33	16.89	10^{-6}	A			
		rs879254506:A	cagttcgtct	G→A	tgactcagac	37.32	2.63	14.87	1.30	16.39	10^{-6}	A			
11	*MFN2* (9927)	rs568548916:A	gcgcgcggag	G→A	agtggcgcgc	95.27	7.68	39.30	3.05	15.82	10^{-6}	A		within a human POAG model using mice [277]: during glaucomatous neurodegeneration, retinal ganglion cells can selectively accumulate Mfn2-excess in a phosphorylated form	▼
		rs886045216:T	cgccgagtcg	C→T	ggggcagcag	95.27	7.68	69.23	5.83	5.48	10^{-6}	A	↑	the ClinVar database [38] documents that the minor alleles of the SNPs rs568548916:A, rs886045216:T, and rs973376897:A as three clinically proven molecular SNP markers for susceptibility to hereditary motor and sensory neuropathy with optic atrophy, in particular in the form of the Charcot-Marie-Tooth disease type 2, which together with POAG are comorbide to amyotrophic lateral sclerosis within a diseasome gene network encompassing the human genes contributing simultaneously to amyotrophic lateral sclerosis and some other diseases [556]	
		rs973376897:A	gacgcccacc	G→A	gaactacagc	21.45	2.62	16.91	1.95	2.83	10^{-6}	A			

Table S5. Cont.

#	Human Gene		Candidate SNP marker				K_D, nM, *in silico*				Significance				Effect of changes in human gene expression on the development of primary open-angle glaucoma (POAG, ☼: "▼" aggravation, "▲" alleviation) [Reference]	☼ ▲ ▼
	NCBI Gene Symbol (NCBI Gene ID)	dbSNP ID:min [437]	5′ flank, 10 bp	WT →min	3 flank, 10 bp	WT MEAN	± SEM	min MEAN	± SEM	Z	p	ϱ	Δ		
		rs1645057147:C	ccccttctttt	A→C	acagcagtgt	17.071.48		27.202.12		7.99	10^{-6}	A	↓	within human age-related disease models using mice aged 5 to 22 months [293]: Mutyh levels decrease with increasing age, which may contribute to the progression of age-related diseases, including POAG	
		rs752665489:G	ctgagtcgtc	T→G	tcgtctcccg	54.79	3.95	64.66	4.90	3.17	10^{-2}	B			
		rs1060504202:A	tgggtacgct	G→A	tgagtcgtct	54.79	3.95	21.09	1.79	17.17	10^{-6}	A		the ClinVar database [38] documents that the minor alleles of the SNPs rs1645057147:C and rs752665489:G together with rs1060504202:A, rs1064795596:A, rs1338038953:A, rs1553127879:T, rs1553136984:A, rs1553137062:A, rs1570591700:A, rs1570591700:T, rs1570591736:A, rs2275602:T, rs587782837:A, rs753502884:T, rs755928199:A, rs755928199:C, rs755928199:T, rs758246147:A, rs766584437:A, rs766584437:T, rs767402084:A, rs767402084:C, rs774530388:T, rs876658588:A, rs878854188:G, and rs878854188:T as twenty six clinically proven molecular SNP markers for susceptibility to hereditary cancer-predisposing syndrome, in particular in the form of the familial adenomatous polyposis 2 associated with congenital hypertrophy of retinal pigment epithelium capable to case an advanced glaucomatous damage in the optic nerve aggravating POAG [557]	
		rs1064795596:A	agtcgtctgt	G→A	tctcccgcct	54.79	3.95	14.72	1.60	20.18	10^{-6}	A			
		rs1338038953:A	cttcttttag	G→A	agcagtgttc	17.07	1.48	8.19	0.77	11.49	10^{-6}	A			
		rs1553127879:T	gtgttccctt	C→T	gcttcacagc	17.07	1.48	9.97	0.83	8.97	10^{-6}	A			
		rs1553136984:A	tcgtctgtgg	G→A	tcccgcctga	54.79	3.95	37.90	3.53	6.26	10^{-6}	A			
		rs1553137062:A	caccgctcgt	C→A	ggcggccatg	54.79	3.95	34.42	3.20	7.90	10^{-6}	A			
		rs1570591700:A	ccgcctgagt	C→A	ccgctcgtct	54.79	3.95	17.22	1.74	18.62	10^{-6}	A			
		rs1570591700:T	ccgcctgagt	C→T	ccgctcgtct	54.79	3.95	27.89	1.99	13.32	10^{-6}	A			
		rs1570591736:A	tcccgcctga	G→A	caccgctcgt	54.79	3.95	37.40	2.27	8.10	10^{-6}	A			
12	*MUTYH* (4595)	rs2275602:T	gtacgctgga	C→T	gtcgtctgtg	54.79	3.95	41.49	3.62	4.91	10^{-6}	A			▼
		rs587788237:A	ccgctcgtct	C→A	cggccatgac	54.79	3.95	30.73	2.96	9.61	10^{-6}	A			
		rs753502884:T	cgtctcccgc	C→T	atgacaccgc	54.79	3.95	41.76	2.42	5.87	10^{-6}	A			
		rs755928199:A	cgcctgagtc	G→A	cgctcgtctc	54.79	3.95	19.48	1.88	17.14	10^{-6}	A	↑		
		rs755928199:C	cgcctgagtc	G→C	cgctcgtctc	54.79	3.95	32.71	3.75	7.61	10^{-6}	A			
		rs755928199:T	cgcctgagtc	G→T	cgctcgtctc	54.79	3.95	21.96	1.87	16.40	10^{-6}	A			
		rs758246147:A	agtgttccct	T→A	tgcttcacag	17.07	1.48	9.45	0.94	8.97	10^{-6}	A			
		rs766584437:A	gggtacgctg	G→A	gagtcgtctg	54.79	3.95	38.16	3.14	6.61	10^{-6}	A		within comparative human disease models using Mutyh-deficient mice versus normal mice [294]: oxidative activation of microglia increases with increasing levels of Mutyh, leading to retinal degeneration as a progression of POAG [295]	
		rs766584437:T	gggtacgctg	G→T	gagtcgtctg	54.79	3.95	25.68	2.36	12.96	10^{-6}	A			
		rs767402084:A	gtcgtctgtg	G→A	ctcccgcctg	54.79	3.95	20.61	1.87	16.88	10^{-6}	A			
		rs767402084:C	gtcgtctgtg	G→C	ctcccgcctg	54.79	3.95	37.84	4.24	5.55	10^{-6}	A			
		rs774530388:T	tctgtgggta	C→T	cgcctgagtc	54.79	3.95	45.59	4.12	3.18	10^{-2}	C			
		rs876658588:A	ctgtgggtac	G→A	gcctgagtcg	54.79	3.95	26.38	2.74	11.55	10^{-6}	A			
		rs878854188:G	cctgagtcgt	C→G	ctcgtctccc	54.79	3.95	36.43	2.66	7.95	10^{-6}	A			
		rs878854188:T	cctgagtcgt	C→T	ctcgtctccc	54.79	3.95	26.46	2.77	11.44	10^{-6}	A			
13	*PMM2* (5373)	rs751782324:A	ctcgtgccaa	C→A	gtgtcttgta	15.27	1.13	19.37	1.23	4.88	10^{-3}	B	↓	within a cohort-based biomedical study [329]: PMM2 deficiency is a biomedical molecular marker for congenital glycosylation disorders aggravating POAG [330]<br>the ClinVar database [38] documents that the minor alleles of the SNP rs751782324:A as a clinically proven molecular SNP marker for susceptibility to PMM2-congenital disorder of glycosylation aggravating POAG [330]	▼
		rs780393027:A	ggagccgggg	G→A	gactgggagg	105.37	7.43	72.66	4.84	7.66	10^{-6}	A		within human POAG model using rats subjected with a chronic artificially elevated intraocular pressure [378]: STAT3 overexpression in the optic nerve head seems to be a candidate molecular marker for the very earlier stages of the POAG origin and development	
14	*STAT3* (6774)												↑	the ClinVar database [38] documents that the minor alleles of the SNPs rs780393027:A and rs902564848:T as two clinically proven molecular SNP markers for susceptibility to Hyper-IgE recurrent infection syndrome 1 causing both ocular allergy and allergic conjunctivitis, treatment of which using corticosteroids has a side effect in the aggravation of POAG [558]	▼
		rs902564848:T	gcgcagactg	G→T	gagctgcggc	105.37	7.43	37.34	2.77	20.27	10^{-6}	A			

Table S5. Cont.

Human Gene		Candidate SNP marker				K_D, nM, *in silico*				Significance				Effect of changes in human gene expression on the development of primary open-angle glaucoma (POAG, ☼: "▼" aggravation, "▲" alleviation) [Reference]	☼ ▲ ▼
#	*NCBI Gene Symbol* (NCBI Gene ID)	dbSNP ID:min [437]	5′ flank, 10 bp	WT →min	3 flank, 10 bp	WT MEAN	± SEM	min MEAN	± SEM	Z	p	ϱ	Δ		
15	*TAP1* (6890)	rs1408055208:T	gcaggatcag	C→T	ttctcgccag	62.32	5.04	48.34	3.27	4.82	10^{-3}	B	↑	within human diabetes mellitus models using mice [381]: Tap1 overexpression may be a candidate molecular marker for diabetes mellitus, which is comorbid with POAG, at least due to their mutual comorbidity with diabetic retinopathy [198]	▼
		rs202053684:T	tcagcctgtt	C→T	ccagcgcagg	62.32	5.04	40.43	3.02	7.86	10^{-6}	A		the ClinVar database [38] documents that the minor alleles of the SNPs rs1408055208:T and rs202053684:T as two clinically proven molecular SNP marker for MHC class I deficiency, while the human POAG model using the DBA/2J mouse has demonstrated a proper similarity to the MHC class I-knockout phenotype at the very earlier stages of the POAG origin and development [559]	
16	*TCF4* (6925)	rs1555710523:T	atcaacgagg	C>T	gggtccgtga	35.86	2.45	31.76	2.16	2.52	10^{-2}	C	↑	according to a cohort-based biomedical study using postmortem donor corneas obtained from the eye bank of Transplant Services [389]: higher risks of Fuchs' endothelial corneal dystrophy comorbid to POAG [124]	▼
		rs17522826:T	tttatcatag	C>T	tttttttcca	8.08	0.51	6.73	0.41	4.20	10^{-3}	B		the ClinVar database [38] documents that the minor alleles of the SNPs rs1555710523:T, rs17522826:T, rs2047109965:T, and rs2061383201:A as four clinically proven molecular SNP markers for susceptibility to Pitt-Hopkins syndrome, among symptoms of which high myopia [560] is a risk factor for POAG [561] as well as to Fuchs endothelial corneal dystrophy, high severity of which is comorbid with POAG [562]	
		rs2047109965:T	tctgcgggtc	C>T	gcccgagagc	35.86	2.45	30.37	2.05	3.46	10^{-3}	B			
		rs2061383201:A	tcctccagtg	G>A	acccttggag	28.46	2.14	21.98	1.64	4.87	10^{-3}	B			
17	*TP53* (7157)	rs1457582183:T	tctttcctag	C→T	ctttccttgc	7.46	0.63	6.04	0.61	3.22	10^{-2}	C	↑	according to a cohort-based biomedical study in Poland [412]: TP53 upregulation can promote cell death through apoptosis during neurodegenerative processes aggravating POAG	▼
		rs1597400604:A	cagtcaggag	C→A	ttacccaatc	47.80	4.43	34.32	2.52	5.61	10^{-6}	A		the ClinVar database [38] documents that the minor alleles of the SNPs rs1457582183:T, rs1597400604:A, and rs34361146:A as three clinically proven molecular SNP markers for susceptibility to both Li-Fraumeni and Hereditary cancer-predisposing syndromes as well as squamous cell carcinoma of the head and neck, among the risk factors for which is tobacco smoking, as with POAG [563]	
		rs34361146:A	ttgcctcttt	C→A	atcacctttc	7.46	0.63	4.96	0.51	6.17	10^{-6}	A			
18	*TXNRD2* (10587)	rs182857388:T	aagtcaagta	C→T	atctttcaga	5.27	0.49	6.35	0.64	2.70	10^{-2}	C	↓	according to a comprehensive molecular biochemical review [418]: TXNRD2 deficiency can lead to an increase in mitochondrial reactive oxygen species (ROS) playing a key role in the pathogenesis of POAG [419]	▼
		rs886509891:T	aaagtcaagt	A→T	tatctttcag	5.27	0.49	6.82	0.51	4.30	10^{-3}	B		the ClinVar database [38] documents that the minor alleles of the SNPsrs182857388:T and rs886509891:T as two clinically proven molecular SNP markers for susceptibility to cardiovascular phenotype as primary dilated cardiomyopathy, while choroidal thickness in children with chronic heart failure through dilated cardiomyopathy is decreased [564] as with POAG [565]	

Table S6. RNA-Seq data on domestic animals versus their wild counterparts (PubMed data [22]).

#	Domestic Animals	Wild Animals	Tissue	N_{DEG}	Ref.
1	dogs	wolves	blood	450	[566]
2	dogs	wolves	frontal cortex	13	[567]
3	pigs	boars	frontal cortex	30	[567]
4	pigs	boars	frontal cortex	34	[568]
5	pigs	boars	pituitary	22	[569]
6	guinea pigs	cavy	frontal cortex	883	[567]
7	tame foxes	aggressive foxes	pituitary	327	[570]
8	domestic rabbits	wild rabbits	frontal cortex	17	[567]
9	domestic rabbits	wild rabbits	parietal-temporal cortex	216	[571]
10	domestic rabbits	wild rabbits	amygdala	118	[571]
11	domestic rabbits	wild rabbits	hypothalamus	43	[571]
12	domestic rabbits	wild rabbits	hippocampus	100	[571]
13	tame rats	aggressive rats	hypothalamus	46	[572]
14	tame rats	aggressive rats	hypothalamus	7	[573]
15	tame rats	aggressive rats	hippocampus	42	[46]
16	tame rats	aggressive rats	midbrain tegmentum	31	[574]
17	tame rats	aggressive rats	periaqueductal gray matter	39	[45]
18	tame rats	aggressive rats	frontal cortex	20	[567]
19	domestic chicken	wild chicken	pituitary	474	[575]
Σ	7 domestic animal species	7 wild animal species	9 tissues	2912	12 Refs

Table S7. Comparison of the effects of unidirectional changes (a) in the expression of the human POAG-related genes on the alleviation and aggravation of POAG and (b) in the expression of the corresponding animal homologous genes that are differentially expressed (DEG) during domestication on the microevolutionary events leading to differences between domestic and wild animals according to our *in silico* analysis .

#	*NCBI Gene Sumbol* (NCBI Gene ID)	**(a) Humans: effects of changes in gene expression on the alleviation ("▲") and aggravation ("▼") of POAG (☼) [Reference]** Deficit (↓)	☼	Excess (↑)	☼	**(b) Animals: effects of changes in gene expression on the pattern of divergence between domestic animals and their wild counterparts from their most recent ancestor** Domestic	Wild	Tissue	DEG-homolog	log₂	P_{ADG}	Deficit (↓)	Excess (↑)	[Ref]
1	*ABCA1* *(19)*	within human disease models using Abca1-null mice [76]: accelerated vision loss can worsen POAG	▼	in a cohort-based biomedical study [77]: ABCA1 excess is a biomedical molecular marker for POAG	▼	domectic rabbit	wild rabbit	amygdala	*Abca6*	5.98	10^{-8}	wild	domestic	[571]
						tame fox	aggressive fox	pituitary	*Abca8*	0.34	10^{-3}			[570]
2	*ABCB1* *(5243)*	within a biomedical pharmacological study using radiolabeled verapamil [78]: for this adjuvant anti-glaucoma medication [79], an ABCB1 inhibitor increased influx to and decreased outflow from the retina across the blood-retinal barrier	▲	within a cohort-based biomedical study [80]: the increasing level of ABCB1 expression with an increase in the duration of drug treatment may contribute to the molecular mechanisms of the emergence and progression of multidrug resistance, which may delay POAG treatment	▼	domectic chicken	wild chicken	pituarity	*Abcb1*	−1.24	10^{-4}	domestic	wild	[575]
						guinea pig	cavy	frontal cortex	*Abcb10*	0.47	0.05	wild	domestic	[567]
3	*ABCC4* (10257)	within human POAG models using Dutch-Belted rabbits subjected with topical application of MK571 as the low molecular weight Abcc4 inhibitor [81]: reduced intraocular pressure alleviating POAG	▲	within a comparative study of human pre- versus post-chemotherapy retinoblastoma cell transcriptome normalized using adjacent non-cancer cells [82]: anticancer chemotherapy increased ABCC4 expression by 33% along with an alleviation of retinoblastoma, which is comorbid to POAG according to a biomedical review [83]	▲	tame fox	aggressive fox	pituitary	*Abcc8*	−0.50	10^{-2}	domestic	wild	[570]
						guinea pig	cavy	frontal cortex	*Abcc9*	1.61	10^{-6}	wild	domestic	[567]
									Abcc12	1.68	10^{-6}			
4	*ADRB2* (154)	within an in vitro autoradiographic study of beta-adrenergic specific binding sites on the human retinal vessels [90]: ADRB2-blockers are drugs in the POAG treatment to reduce intraocular pressure	▲	within a biomedical cohort-based study [91]: ADRB2-excess along with its agonistic autoantibodies are biomedical molecular markers for any POAG development state	▼	domectic rabbit	wild rabbit	parietal-temporal cortex	*Adrb2*	1.40	10^{-2}	wild	domestic	[571]
5	*AQP1* (358)	within human POAG models using mice lacking Aqp1 [107]: reduced both aqueous fluid production and intraocular pressure that might alleviate POAG	▲	according to a comprehensive biomedical review [108]: within human post-injury trabecular meshwork regeneration cellular models the using adipose-derived stem cells, AQP1 excess along with improved wound healing in that might slow down optic nerve head damage as the alleviation of POAG	▲	tame rat	aggressive rat	frontal cortex	*Aqp1*	1.18	0.05	wild	domestic	[567]
								hippocampus		5.91	10^{-2}			[46]
						domectic rabbit	wild rabbit	hippocampus		−3.46	10^{-2}	domestic	wild	[571]
						tame fox	aggressive fox	pituitary	*Aqp4*	−0.53	10^{-2}			[570]
						guinea pig	cavy	frontal cortex	*Aqp5*	−2.91	10^{-8}			[567]
						domectic chicken	wild chicken	pituarity	*Aqp10*	−1.05	10^{-4}			[575]
6	*ARHGEF12* *(23365)*	within a retrospective literature meta-analysis [109]: ARHGEF12 deficit might be a candidate molecular marker for reduced organism viability	▼	according to a retrospective literature meta-analysis [109]: improved eye development	▲	dog	wolf	blood	*Arhgef12*	−3.29	10^{-4}	domestic	wild	[566]
7	*ASB10* *(136371)*	according to comprehensive review [110]: ASB10 mutational loss-of-function may impair an adequate response to inflammatory signals in the eye that could lead to glaucomatous vision loss	▼	within cellular POAG models using human and mice cells [111]: improved autophagy, which may alleviate POAG associated with increased intraocular pressure [112]	▲	guinea pig	cavy	frontal cortex	*Asb10*	−3.69	10^{-8}	domestic	wild	[567]
									Asb13	0.65	0.05	wild	domestic	

Table S7. Cont.

#	NCBI Gene Sumbol (NCBI Gene ID)	(a) Humans: effects of changes in gene expression on the alleviation ("▲") and aggravation ("▼") of POAG (☼) [Reference]				(b) Animals: effects of changes in gene expression on the pattern of divergence between domestic animals and their wild counterparts from their most recent ancestor								
		Deficit (↓)	☼	Excess (↑)	☼	Domestic	Wild	Tissue	DEG-homolog	$\log_2$	P_{ADG}	Deficit (↓)	Excess (↑)	[Ref]
8	*BMP4* (652)	within human disease models using mice subjected with an artificial glaucoma induction and, next, treated with recombinant adeno-associated virus carrying the human CHRDL1 gene as BMP4-blocker [122]: BMP4-blockade decreases the number of surviving retinal ganglion cells	▼	according to a cohort-based biomedical study [123]: BMP4 excess is a biomedically proven molecular marker for corneal dystrophy comorbid to POAG [124]	▼	tame fox	aggressive fox	pituitary	*Bmp2*	0.37	0.05	wild	domestic	[570]
									Bmp7	−0.38	0.05	domestic	wild	
						domectic chicken	wild chicken	pituarity	*Bmp6*	−0.60	10^{-3}			[575]
9	*CACNA2D1* (781)	within cellular models of human eye injuries [125]: CACNA2D1-blockers improve eye injury repair that can relieve POAG	▲	within cellular models of human diseases using transgenic mice overexpressing Cacna2d1 [126]: pain hypersensitivity that can worsen POAG	▼	tame rat	aggressive rat	hypothalamus	*Cacna2d3*	−0.41	0.05	domestic	wild	[573]
10	*CAV1* (857)	within human disease models using Cav1-knockout mice [129]: blood-retinal barrier breakdown, mural cell alteration, and venous enlargement with branch veins being frequent sites of breakdown that altogether may contribute to retinal disorders with vascular pathologies, including retinopathy, uveoretinitis, and POAG	▼	within human POAG cellular models using human embryonic kidney cell line HEK293 transfected by vector with one more mice Cav1 gene [130]: caveolin-1 excess along with protection against lipotoxicity, which is risk factor for elevated intraocular pressure aggravating POAG [131]	▲	guinea pig	cavy	frontal cortex	*Cav1*	1.03	0.05	wild	domestic	[567]
11	*CAV2* (858)	within a biomedical cohort-based comparative study of minor G versus ancestral T alleles of the known biomedical SNP-marker rs17588172 for POAG using Korean participants [132]: minor G allele downregulates CAV2 that elevates intraocular pressure worsening POAG	▼	within cellular POAG models using differentiated rat PC12 cell line [133]: caveolin-2 is up-regulated in response to the mechanical injury of differentiated PC12 cells that can improve post-traumatic neuron repair retarding POAG development	▲	guinea pig	cavy	frontal cortex	*Cav1*	1.03	0.05	wild	domestic	[567]
12	*CDC7* (8317)	within a cohort-based study [248]: CDC7-blockers within a gene-target therapy can retard atherogenesis as a risk-factor of POAG [135]	▲	within a cohort-based biomedical study [136]: Cdc7 overexpression contributes to DNA-damage resistance that can alleviate POAG [137]	▲	guinea pig	cavy	frontal cortex	*Cdc7*	1.26	0.05	wild	domestic	[567]
13	*CDH1* (999)	within human disease cellular models [138]: CDH1 insufficiency as a molecular marker for differentiation to epithelial cells can elevate risks of epithelial to mesenchymal transition and, thus, fibrosis as a post-POAG-surgery complication	▼	within human disease cellular models [138]: CDH1 excess as a molecular marker for differentiation to epithelial cells can prevent epithelial to mesenchymal transition and, thus, fibrosis as a post-POAG-surgery complication	▲	guinea pig	cavy	frontal cortex	*Cdh1*	1.28	10^{-2}	wild	domestic	[567]
						domectic rabbit	wild rabbit	hippocampus	*Cdh1*	−2.44	10^{-2}	domestic	wild	[571]
								frontal cortex	*Cdh23*	1.46	0.05	wild	domestic	[567]
						domectic chicken	wild chicken	pituarity	*Cdh8*	−0.52	10^{-3}	domestic	wild	[575]
									Cdh12	0.60	10^{-4}			
						tame fox	aggressive fox		*Cdh13*	0.52	10^{-3}	wild	domestic	[570]
						dog	wolf	blood	*Cdh13*	3.76	10^{-3}			[566]
									Cdh24	−4.91	10^{-10}	domestic	wild	
14	*CDKN2A* (1029)	within a retrospective comprehensive complex GWAS and QTL meta-analysis [141]: three biomedically proven SNP-markers rs1063192, rs7865618 and rs2157719 for POAG are associated with CDKN2A decreased expression gene in the cerebral cortex	▼	according to comprehensive biomedical review [142]: C DKN2A excess can prevent atherosclerosis aggravating POAG [143]	▲	pig	boar	pituitary gland	*Cdkn2b*	2.84	10^{-2}	wild	domestic	[569]

Table S7. Cont.

#	NCBI Gene Sumbol (NCBI Gene ID)	(a) Humans: effects of changes in gene expression on the alleviation ("▲") and aggravation ("▼") of POAG (☼) [Reference]				(b) Animals: effects of changes in gene expression on the pattern of divergence between domestic animals and their wild counterparts from their most recent ancestor								
		**Deficit (↓)**	**☼**	**Excess (↑)**	**☼**	**Domestic**	**Wild**	**Tissue**	**DEG-homolog**	**log_2**	**P_{ADG}**	**Deficit (↓)**	**Excess (↑)**	**[Ref]**
15	*CDKN2B* (1030)	within human POAG models using Cdkn2b-knockout mice [144]: more vulnerable to retinal ganglion cell loss in response to elevated intraocular pressure that can aggravate POAG	▼	within a phytopharmaceutical microarray-based transcriptome study using murine microglial cells treated with 15-methoxypinusolidic acid as a modern promising anti-POAG phytomedication extracted from Biota orientalis [145]: Cdkn2b excess causing microglia cell cycle arrest, which can inhibit proliferation of microglia cells and induce apoptosis of these cells that may slow down neurodegeneration of both optic nerve and retina	▲	pig	boar	pituitary gland	*Cdkn2b*	2.84	10^{-2}	wild	domestic	[569]
16	*COL1A1* (1277)	within human POAG filtration surgery models using mice [152]: auxiliary treatment with mitomycin-C during surgery downregulates Col1a1 and simultaneously prevents fibrosis as post-surgery	▲	within a biomedical cohort-based RNA-Seq study [153]: COL1A1 excess is a biomedical molecular marker for POAG	▼	dog	wolf	blood	*Col1a1l*	−2.69	10^{-3}	domestic	wild	[566]
17	*COL8A1* (1295)	within human disease models using Col8a1-knockout mice [154]: thinning of both corneal stroma and of Descemet's membrane as symptoms of POAG [155]	▼	within a biomedical cohort-based transcriptome study of microneedling therapy [156]: COL8A1 upregulation is a biomedical molecular marker for wound healing, which is the major problem of glaucoma surgery according to a comprehensive retrospective review [157]	▲	domectic rabbit	wild rabbit	parietal-temporal cortex	*Col8a1*	0.72	10^{-5}	wild	domestic	[571]
						domectic chicken	wild chicken	pituarity	*Col8a2*	0.60	10^{-5}			[575]
18	*COL8A2* (1296)	within human POAG models using Col8a2-defective mutant mice [158]: retinal ganglion cell damage resistance, which can alleviate POAG	▲	within both biomedical cohort-based transcriptome study of post-surgical corneal wound healing in glaucoma patients and biomedical glaucoma surgery models using mice [159]: COL8A2 upregulation is one among three most vital gene expression changes during the very first week of wound healing immediately after glaucoma surgery until its fibrotic complication may begin that can require auxiliary therapy suppressing collagen-encoding genes	▲	domectic rabbit	wild rabbit	parietal-temporal cortex	*Col8a1*	0.72	10^{-5}	wild	domestic	[571]
						domectic chicken	wild chicken	pituarity	*Col8a2*	0.60	10^{-5}			[575]
19	*CP* (1356)	within a biomedical cohort comparison study of patients with cataracts with and without POAG [160]: low serum ceruloplasmin levels are molecular markers of POAG risk in cataract	▼	within nonhuman primate POAG models using male juvenile cynomolgus monkeys (*Macaca fascicularis*) treated with an argon laser to scar the trabecular meshwork in one eye until an intraocular pressure increased indicating glaucomatous eye [161]: ceruloplasmin excess in the retina that may be needed as part of antioxidant system, which must counteract the inevitable damaging effects of free radicals on the head of the optic nerve when perceiving light flux as a vital function of the eyes	▲	guinea pig	cavy	frontal cortex	*Cp*	1.34	10^{-5}	wild	domestic	[567]
20	*CPNE1* (8904)	within human disease cellular models using siRNA-based CPNE1-knockdown [166]: inhibited tumor growth and promoted cell apoptosis in breast cancer, a complication of which is tamoxifen-associated maculopathy in POAG [167]	▲	within human disease cellular models using human nasopharyngeal carcinoma cell lines NPC under different radiations up to 8 Gy [168]: CPNE1 excess along with improved radioresistance that can prevent glaucomagenesis following ionizing radiation exposure according to comprehensive biomedical review [169]	▲	tame fox	aggressive fox	pituitary	*Cpne2*	0.36	0.05	wild	domestic	[570]
									Cpne8	−0.30	0.05			
						guinea pig	cavy	frontal cortex	*Cpne5*	−0.69	0.05	domestic	wild	[567]
									Cpne7	−1.28	10^{-2}			

Table S7. Cont.

#	*NCBI Gene Sumbol* (NCBI Gene ID)	(a) Humans: effects of changes in gene expression on the alleviation (“▲”) and aggravation (“▼”) of POAG (☼) [Reference]				(b) Animals: effects of changes in gene expression on the pattern of divergence between domestic animals and their wild counterparts from their most recent ancestor								
		**Deficit (↓)**	**☼**	**Excess (↑)**	**☼**	**Domestic**	**Wild**	**Tissue**	**DEG-homolog**	**log_2**	**P_{ADG}**	**Deficit (↓)**	**Excess (↑)**	**[Ref]**
21	*CYP1A1* (1543)	within human disease models using senescence-accelerated OXYS rats as a commonly accepted animal model of human age-related macular degeneration compared with Wistar rats as a norm [172]: Cyp1a1-deficit in the retina may indicate the presence of oxidative stress contributing both retinopathy and age-related macular degeneration, which are both comorbid with POAG, at least as age-related diseases [96]	▼	within models of the effect of smoking on human health using rats [173]: Cyp1a1-upregulation in the iris/ciliary body and retina together with age-related macular degeneration with choroidal neovascularization as the most severe complication	▼	domectic rabbit	wild rabbit	parietal-temporal cortex	*Cyp1b1*	1.40	10^{-2}	wild	domestic	[571]
22	*CYP1B1* (1545)	within a biomedical cohort-based study [174]: both reduced catalytic activity and decreased protein stability can elevate risks of POAG	▼	according to a biomedical radiotherapy review [169]: ultraviolet radiation can upregulate CYP1B1 that may often manifest in glaucomagenesis as side effect following ionizing radiotherapy	▼	domectic rabbit	wild rabbit	parietal-temporal cortex	*Cyp1b1*	1.40	10^{-2}	wild	domestic	[571]
23	*EFEMP1* (2202)	within human disease models using Efemp1-knockout mice [186]: corneal dysfunction, which is comorbid to POAG at least within exfoliation syndrome as the most common identifiable cause of POAG in the world [187]	▼	within a biomedical cohort-based study [188]: fibulin-3 overexpression holds future potential as new promising intraocular pressure lowering therapy for POAG	▲	tame fox	aggressive fox	pituitary	*Efemp1*	−0.33	0.05	domestic	wild	[570]
						domectic chicken	wild chicken			−0.56	10^{-3}			[575]
24	*EGFR* (1956)	within a cohort-based biomedical pharmacological study [189]: anti-cancer medications inhibiting EGFR may elevate risks of dry eye, which is comorbid to POAG at least as a side effect of antihypertensive medications in POAG [104]	▼	within human disease models using chicken [190]: low molecular weight medication brimonidine treats POAG due to trans-activation of Egrf	▲	domectic chicken	wild chicken	pituarity	*Egfr*	0.52	10^{-3}	wild	domestic	[575]
25	*ENO4* (387712)	within a cohort-based biomedical studies [193]: ENO4-deficit is a biomedical molecular marker for autoimmune thyroid disease, a risk-factor of which is ophthalmic surgery against POAG [194]	▼	within a cohort-based biomedical studies [193]: ENO4-excess is a biomedical molecular marker for resistance against the autoimmune thyroid disease, a risk-factor of which is ophthalmic surgery against POAG [194]	▲	domectic rabbit	wild rabbit	hippocampus	*Eno4*	−2.04	10^{-2}	domestic	wild	[571]
						domectic chicken	wild chicken	pituarity		0.77	10^{-6}	wild	domestic	[575]
26	*ESR1* (2099)	within an ethnopharmacological study of anti-diabetic Gegen Qinlian Decoction (GQD) within Chinese traditional medicine [197]: ESR1-deficit is a biomedical molecular marker for diabetes mellitus, which is comorbid with POAG, at least due to their mutual comorbidity with diabetic retinopathy [198]	▼	within a human neuroprotection cellular models using primary neocortical cell cultures established from mouse embryos on the 15th day of gestation, which were first exposed at either hypoxia or ischemic conditions and, next, treated with ospemifene as both new promising neuroprotective medication and novel selective estrogen receptor modulator simultaneously [199]: four-fold ESR1 protein excess compared to the normal condition along with neuroprotective effects by means of both proapoptotic proteins suppression and antiapoptotic proteins upregulation that altogether may slow the progression of POAG in terms of neurodegeneration of both the optic nerve and retinal ganglion cells	▲	tame fox	aggressive fox	pituitary	*Esr2*	−0.32	0.05	domestic	wild	[570]

Table S7. Cont.

#	*NCBI Gene Sumbol* **(NCBI Gene ID)**	**(a) Humans: effects of changes in gene expression on the alleviation (“▲”) and aggravation (“▼”) of POAG (☼) [Reference]**				**(b) Animals: effects of changes in gene expression on the pattern of divergence between domestic animals and their wild counterparts from their most recent ancestor**								
		**Deficit (↓)**	**☼**	**Excess (↑)**	**☼**	**Domestic**	**Wild**	**Tissue**	**DEG-homolog**	**log₂**	P_{ADG}	**Deficit (↓)**	**Excess (↑)**	**[Ref]**
27	*ESR2* *(2100)*	according to a biomedical case report [167]: a complication of tamoxifen therapy for ESR2-deficient pT1 breast benign tumor to prophylaxis carcinogenesis may be ocular toxicity that may cause POAG, retinopathy and loss of visual acuity	▼	within a human neuroprotection cellular models using primary neocortical cell cultures established from mouse embryos on the 15th day of gestation, which were first exposed at either hypoxia or ischemic conditions and, next, treated with ospemifene as both new promising neuroprotective medication and novel selective estrogen receptor modulator simultaneously [199]: nine-fold ESR2 protein excess compared to the ischemic condition along with neuroprotective effects by means of both proapoptotic proteins suppression and antiapoptotic proteins upregulation that altogether may slow the progression of POAG in terms of neurodegeneration of both the optic nerve and retinal ganglion cells	▲	tame fox	aggressive fox	pituitary	*Esr2*	−0.32	0.05	domestic	wild	[570]
28	*GPX1* (2876)	within human disease model using elderly mice subjected to artificial weakening of neuroprotective abilities [213]: in POAG, Gpx1 deficit may be a biomedical molecular marker for both reduced retinal ganglion cell survival and retinal ganglion cell loss in the elderly because of their hypersensitivity to oxidative stress	▼	according to a comprehensive molecular medicine review [214]: medication-derived Gpx1 upregulation might open potential future therapies to harness the beneficial effects of this major ubiquitous antioxidant enzyme, which must counteract the inevitable damaging effects of free radicals on the optic nerve head when perceiving light flux as a vital function of the eyes in line with nonhuman primate POAG models using male juvenile cynomolgus monkeys (Macaca fascicularis) treated with an argon laser to scar the trabecular meshwork in one eye until an intraocular pressure increased indicating glaucomatous eye [161]	▲	dog	wolf	blood	*Gpx1*	−4.18	10^{-5}	domestic	wild	[566]
						domectic chicken	wild chicken	pituarity	*Gpx7*	−0.52	10^{-2}			[575]
29	*GRIN2B* (2904)	within human neurodegenerative disease cellular models using human induced pluripotent stem cells carrying either GRIN2B-deletion or GRIN2B loss-of-function mutation [215]: impaired both calcium influx and membrane depolarization that can aggravate POAG [216]	▼	within human disease models using mice [217]: Grin2b excess can enhance glutamatergic signaling, which can elevate excitotoxicity accelerating neurodegeneration in POAG	▼	tame fox	aggressive fox	pituitary	*Grin3a*	0.46	10^{-2}	wild	domestic	[570]

Table S7. Cont.

#	NCBI Gene Sumbol (NCBI Gene ID)	(a) Humans: effects of changes in gene expression on the alleviation ("▲") and aggravation ("▼") of POAG (☼) [Reference]				(b) Animals: effects of changes in gene expression on the pattern of divergence between domestic animals and their wild counterparts from their most recent ancestor								
		**Deficit (↓)**	**☼**	**Excess (↑)**	**☼**	**Domestic**	**Wild**	**Tissue**	**DEG-homolog**	**log₂**	**P_{ADG}**	**Deficit (↓)**	**Excess (↑)**	**[Ref]**
30	*GSTM1* (2944)	within an exhaustive meta-analysis of the occurrence of single-nucleotide polymorphisms (SNPs) in human subpopulations with different regional and ethnic specificities [218]: GSTM1-null genotypes are associated with increased POAG risk in Asian populations	▼	within human disease model using rats subjected with corneal wounds, which was then regularly irradiated with blue light to slow down its healing and, at the same time, treated orally with the Peucedanum japonicum Thunb. leaf extract to repair these wounds [219]: extract-dose-dependent acceleration in wound healing due to Gstm1-upregulation that can alleviate POAG that can improve post-surgery recovery in POAG	▲	domectic rabbit	wild rabbit	parietal-temporal cortex<br>hippocampus<br>hypothalamus<br>amygdala	*Gstm2*	2.45<br>2.18<br>2.22<br>2.24	10^{-3}<br>0.05<br>0.05<br>10^{-4}	wild	domestic	[571]
31	*GSTP1* (2950)	within a biomedical cohort-based proteomic study [226]: GSTP1 downregulation in aqueous humour is a biomedical molecular marker for POAG	▼	within a phytopharmacological dietary wolfberry study [227]: GSTP1 upregulation can improve retinal mitochondrial biogenesis that may alleviate POAG	▲	dog	wolf	blood	*Gstp1l*	−1.67	0.05	domestic	wild	[566]
32	*GSTT1* (2952)	in a cohort-based biomedical study [228]: GSTT1 is one of the major differentially hypermethylated genes, hence one of the lowest differential expressions in African Americans with metabolic syndrome compared to those without it, while metabolic syndrome is a risk factor for high intraocular pressure exacerbating POAG [229]	▼	within a comprehensive meta-analysis of transcriptomes from patients with and without glaucoma, verified using qPCR [230]: GSTT1 excess is a biomedical molecular marker for POAG	▼	tame fox	aggressive fox	pituitary	*Gstt2*	−0.69	10^{-2}	domestic	wild	[570]
33	*HDAC6* (10013)	within human disease models using rats subjected with an artificial retinal ischaemia/reperfusion injury [233]: HDAC6-inhibitor tubacin elevated survival of the retinal ganglion cell in POAG	▲	according to comprehensive review [234]: HDAC6 excess can improve microtubule dynamics beneficial for axonal growth, as well as supervise and coordinate stress response including stress granule formation, chaperone upregulation, inflammatory tolerance increase and facilitates the autophagy of both misfolded proteins and protein misaggregates leading to neurodegeneration, that altogether can alleviate POAG	▲	domectic chicken	wild chicken	pituarity	*Hdac11*	−1.90	10^{-6}	domestic	wild	[575]
34	*HES1* (3280)	within human POAG cellular models using primary human trabecular meshwork cells subjected with oxidative stress along with a transfection of plasmid carrying shRNA against human HES1 gene [235]: rescued POAG-related defects	▲	within human POAG cellular models using primary human trabecular meshwork cells subjected with oxidative stress [235]: HES1 upregulation leading POAG-related defects	▼	domectic rabbit	wild rabbit	parietal-temporal cortex	*Hes3*	−1.91	10^{-3}	domestic	wild	[571]
						domectic chicken	wild chicken	pituarity	*Hes5*	0.84	10^{-3}	wild	domestic	[575]

Table S7. Cont.

#	NCBI Gene Sumbol (NCBI Gene ID)	(a) Humans: effects of changes in gene expression on the alleviation ("▲") and aggravation ("▼") of POAG (☼) [Reference]				(b) Animals: effects of changes in gene expression on the pattern of divergence between domestic animals and their wild counterparts from their most recent ancestor								
		**Deficit (↓)**	**☼**	**Excess (↑)**	**☼**	**Domestic**	**Wild**	**Tissue**	**DEG-homolog**	**log$_2$**	**P_{ADG}**	**Deficit (↓)**	**Excess (↑)**	**[Ref]**
35	*HSPA4* (3308)	within human diseases models using Hspa4-deficient mice [236]: enhanced apoptosis in inflammatory bowel disease comorbid with POAG [237]	▼	within human disease cellular models using murine cells transfected with plasmid carrying an additional Hspa4 gene copy [238]: Hspa4 excess can protect cells against oxidative damage that may prevent an important pathogenetic step in POAG [239]	▲	tame rat	aggressive rat	midbrain tegmentum	*Hspa1a*	−2.10	0.05	domestic	wild	[574]
										−2.19	10^{-7}			[574]
								hypothalamus	*Hspa1b*	−1.25	0.05			[572]
								periaqueductal gray		−2.14	10^{-7}			[45]
						domectic rabbit	wild rabbit	parietal-temporal cortex	*Hspa5*	−1.12	0.05			[571]
									Hspa8	−1.46	10^{-9}			[571]
								amygdala	*Hspa5*	−1.12	0.05			[571]
									Hspa8	−1.10	10^{-3}			[571]
								hippocampus		−1.36	10^{-2}			[571]
36	*HSPA5* (3309)	within a pharmaceutical study on the dexamethasone side effects during anti-inflammatory treatment using human trabecular meshwork cells [240]: HSPA5 deficit can increase both outflow resistance and intraocular pressure that may aggravate POAG	▼	within a pharmaceutical study using rats subjected with valproate [241]: Hspa5 upregulation, which can protect retina against ischemia-reperfusion injury and, thereby, retard POAG development	▲	tame rat	aggressive rat	midbrain tegmentum	*Hspa1a*	−2.10	0.05	domestic	wild	[574]
										−2.19	10^{-7}			[574]
								hypothalamus	*Hspa1b*	−1.25	0.05			[572]
								periaqueductal gray		−2.14	10^{-7}			[45]
						domectic rabbit	wild rabbit	parietal-temporal cortex	*Hspa5*	−1.12	0.05			[571]
									Hspa8	−1.46	10^{-9}			[571]
								amygdala	*Hspa5*	−1.12	0.05			[571]
									Hspa8	−1.10	10^{-3}			[571]
								hippocampus		−1.36	10^{-2}			[571]

Table S7. Cont.

#	NCBI Gene Sumbol (NCBI Gene ID)	(a) Humans: effects of changes in gene expression on the alleviation ("▲") and aggravation ("▼") of POAG (☼) [Reference]				(b) Animals: effects of changes in gene expression on the pattern of divergence between domestic animals and their wild counterparts from their most recent ancestor								
		Deficit (↓)	☼	Excess (↑)	☼	Domestic	Wild	Tissue	DEG-homolog	$\log_2$	P_{ADG}	Deficit (↓)	Excess (↑)	[Ref]
37	*IL1B* (3553)	within human eye disease models using mice [250]: Il1b-downregulation can reduce apoptosis of retinal ganglion cells that may relieve POAG	▲	within human POAG cellular models using human trabecular meshwork cell line TM-1 transfected with plasmid vector carrying mutant glaucomatous variants of MYOC gene [249]: IL1B upregulation as a candidate molecular marker for POAG	▼	guinea pig	cavy	frontal cortex	*Il1b*	2.27	10^{-2}	wild	domestic	[567]
38	*IL6R* (3570)	according to an exhaustive review [259]: the anti-IL6R humanized monoclonal antibody tocilizumab is a new promising drug for rheumatoid arthritis, a risk factor for which is POAG [194]	▲	within a cohort-based biomedical study [257]: IL6R excess in trabecular meshwork is a biomedical molecular marker for trabecular meshwork fibrosis in POAG	▼	domectic rabbit	wild rabbit	parietal-temporal cortex	*Il6r*	1.38	10^{-2}	wild	domestic	[571]
39	*ITIH1* (3697)	according to an exhaustive retrospective analysis of tumor-related transcriptomes [260]: ITIH1-deficit may be a biomedical molecular marker for breast tumors, a side effect of treatment of which using tamoxifen may be the tamoxifen-associated maculopathy aggravating POAG [167]	▼	within human disease models using mice [261]: Itih1-excess can exacerbate systemic insulin resistance, which may contribute to POAG development [262]	▼	domectic chicken	wild chicken	pituarity	*Itih2*	−0.59	0.05	domestic	wild	[575]
						guinea pig	cavy	frontal cortex	*Itih5*	−0.57	0.05			[567]
40	*LRRC27* (80313)	within a biomedical cohort-based study [268]: LRRC27 hypermethylation reducing its expression is a biomedical molecular marker for resistance to low-fat/low-carbohydrate Mediterranean diet together with physical activity as a treatment for obesity, which is positively correlated with POAG [115]	▼	within a biomedical cohort-based study of patients with preeclampsia in comparison with patients with gestational hypertension [269]: LRRC27 excess is a biomedical molecular marker for preeclampsia with thrombosis, vascular and endothelial dysfunction, the last of which is successfully treated using Tanakan in POAG as its relief [270]	▼	domectic rabbit	wild rabbit	amygdala	*Lrrc2*	2.01	0.05	wild	domestic	[571]
						guinea pig	cavy	frontal cortex	*Lrrc2*	3.77	10^{-9}			[567]
									Lrrc40	1.20	10^{-6}			
						domectic chicken	wild chicken	pituarity	*Lrrc18*	−0.73	10^{-4}	domestic	wild	[575]
41	*LTBP2* (4053)	within biomedical studies of Pakistani families and Gypsy patients [271]: LTBP2-null is a biomedical molecular marker for POAG	▼	within human glaucoma tissue models using mice with the interference RNA-impaired ciliary zonule formation leading lens luxation as POAG symptom and, next, treated with exogenous recombinant purified Ltbp2 within mice eye tissue level ex vivo [272]: restored unfragmented and bundled ciliary zonules to almost their norms	▲	domectic rabbit	wild rabbit	parietal-temporal cortex	*Ltbp2*	1.17	10^{-2}	wild	domestic	[571]
42	*MMP1* (4312)	within a cohort-based biomedical study [280]: pilocarpine-based treatment in POAG can accelerate with a decrease in MMP1 level	▲	within a cohort-based biomedical study [281]: MMP1 excess in aqueous humor can be a biomedical molecular marker for susceptibility to POAG	▼	dog	wolf	blood	*Mmp14*	1.78	0.05	wild	domestic	[566]
43	*MMP2* (4313)	within human glaucoma models using Mmp2-null mice [282]: resistance to retinal neovascularization aggravating POAG as well as attenuated retinal ganglion cell death and suppressed tumor necrosis factor, which may protect against neurodegeneration and neuroinflammation, respectively	▲	within a cohort-based study cohorts: in both aqueous humor [283] and conjunctival stroma [280], MMP2 excess is a biomedical molecular marker for POAG	▼	dog	wolf	blood	*Mmp14*	1.78	0.05	wild	domestic	[566]

Table S7. Cont.

#	NCBI Gene Sumbol (NCBI Gene ID)	(a) Humans: effects of changes in gene expression on the alleviation ("▲") and aggravation ("▼") of POAG (☼) [Reference]				(b) Animals: effects of changes in gene expression on the pattern of divergence between domestic animals and their wild counterparts from their most recent ancestor								
		**Deficit (↓)**	**☼**	**Excess (↑)**	**☼**	**Domestic**	**Wild**	**Tissue**	**DEG-homolog**	**$\log_2$**	P_{ADG}	**Deficit (↓)**	**Excess (↑)**	**[Ref]**
44	*MMP9* (4318)	within human glaucoma models using transgenic Mmp9-deficient mice [284]: elevated intraocular pressure aggravating POAG	▼	within an observational pharmaceutical study using tear sample collection [285]: latanoprost, being a commonly accepted medication against POAG, can simultaneously both upregulate MMP9 and reduce intraocular pressure that can alleviate POAG	▲	dog	wolf	blood	*Mmp14*	1.78	0.05	wild	domestic	[566]
45	*MMP12* (4321)	within human glaucoma models using Mmp12-knockout mice [286]: decreased levels of both adhesion molecule and inflammatory cytokines along with reduced vascular leakage in oxygen-induced retinopathy, as well as markedly reduced macrophage content in the retina with impaired macrophage migratory capacity, attenuated retinal capillary dropout and mitigated pathological retinal neovascularization that all improvements to POAG were reproduced using normal mice subjected with MMP408 as pharmacological inhibitor of MMP12	▲	within a cohort-based biomedical study [281]: MMP12 excess in aqueous humor can be a biomedical molecular marker for susceptibility to POAG	▼	dog	wolf	blood	*Mmp14*	1.78	0.05	wild	domestic	[566]
46	*MPP7* (143098)	within cohort-based study together with POAG models using human trabecular meshwork cells that was verified using murine eye subjected with mechanical stress mimicking the POAG development scenario [287]: MPP7 deficit can be a biomedical molecular marker for susceptibility to POAG	▼	within POAG models using human trabecular meshwork cells [112] along with an observations within cancer-derived xenograft mice [288]: MPP7 excess can stimulate autophagy that can reduce ocular hypertension as a relief in POAG	▲	dog	wolf	blood	*Mpp2*	−4.85	10^{-5}	domestic	wild	[566]
						guinea pig	cavy	frontal cortex	*Mpp3*	0.63	10^{-2}	wild	domestic	[567]
						domectic rabbit	wild rabbit	parietal-temporal cortex	*Mpp4*	−1.44	0.05	domestic	wild	[571]
						domectic chicken	wild chicken	pituarity	*Mpp7*	0.53	10^{-4}	wild	domestic	[575]
47	*NOS2* (4843)	within a phytopharmaceutical study [302]: resveratrol as natural phytoantioxidant with antibacterial and antifungal protective properties can suppress NOS2 to medicate POAG	▲	within cohort-based iris sample postmortem study [303]: both excess and hyperactivity of NOS2 can be a biomedical molecular marker for susceptibility to POAG	▼	tame rat	aggressive rat	hypothalamus	*Nos1*	−0.47	0.05	domestic	wild	[573]
48	*NOS3* (4846)	within human disease models using NOS3 knockout mice [304]: higher risks of both hypertension and atherosclerosis, while both atherosclerosis [143] and hypertension [9] can aggravate POAG	▼	within a cohort-based biomedical study [305]: positive therapeutic effects of mindfulness meditation on intraocular pressure and trabecular meshwork in patients in India with medically uncontrolled primary open angle glaucoma was accompanied by NOS3 upregulation	▲	tame rat	aggressive rat	hypothalamus	*Nos1*	−0.47	0.05	domestic	wild	[573]
49	*NTRK2* (4915)	according to exhaustive review [316]: compared to normal, Ntrk2 knockout mice have accelerated photoreceptor degeneration and loss of retinal ganglion cells, as well as more severe damage to the optic nerve	▼	within a cohort-based retrospective biomedical study [317]: total healing effect of drugs used to treat glaucoma associated with retinal TrkB upregulation as neuroprotector	▲	domectic rabbit	wild rabbit	amygdala	*Ntrk1*	3.51	10^{-2}	wild	domestic	[566]

Table S7. Cont.

#	NCBI Gene Sumbol (NCBI Gene ID)	(a) Humans: effects of changes in gene expression on the alleviation ("▲") and aggravation ("▼") of POAG (☼) [Reference] Deficit (↓)	☼	Excess (↑)	☼	(b) Animals: effects of changes in gene expression on the pattern of divergence between domestic animals and their wild counterparts from their most recent ancestor Domestic	Wild	Tissue	DEG-homolog	$\log_2$	P_{ADG}	Deficit (↓)	Excess (↑)	[Ref]
50	*PARP1* (142)	within human POAG models using Parp1-knockout mice [137]: increases resistance to retinal neurodegeneration without affecting retinal function	▲	within cohort-based elderly women biomedical study [326]: smoking elevates PARP1 level that can speed up both apoptosis and POAG	▼	domectic chicken	wild chicken	pituarity	*Parp3*	−0.95	10^{-3}	domestic	wild	[575]
51	*PMM2* (5373)	within a cohort-based biomedical study [329]: PMM2 deficiency is a biomedical molecular marker for congenital glycosylation disorders aggravating POAG [330]	▼	exogenous recombinant insulin injections can both upregulate PMM2 within human disease cellular models using COS-7 cells [331] and relieve POAG according to an exhaustive review [332]	▲	pig	boar	frontal cortex	*Pmm1*	1.30	10^{-7}	wild	domestic	[567]
						dog	wolf	blood	*Pmm1*	−3.64	10^{-3}	domestic	wild	[566]
52	*PTGFR* (5737)	within a cohort-based biomedical study [340]: the effectiveness of the response to latanoprost as a drug against POAG decreases with a decrease in the transcriptional activity of the PTGFR gene as measured experimentally [341]	▼	according to an exhaustive pharmaceutical review [342]: artificial PTGFR-agonists are drugs against POAG	▲	domectic rabbit	wild rabbit	parietal-temporal cortex	*Ptgfr*	1.53	10^{-2}	wild	domestic	[571]
53	*RHOA* (387)	according to an exhaustive review [346]: drug-induced therapeutic inhibition of RHOA can decrease aqueous humor drainage via the trabecular meshwork in the drainage pathway that can alleviate POAG	▲	within human neuroregeneration models using rats subjected with artificial sciatic transection injuries [347]: post-injury activated GTP-bound RhoA dramatically upregulated in both neurons and axons, which shown a RhoA-dose-related increase in the initiation of neurite outgrowth, and in the proportion with long neurites in the extent and distance of axons, and partnered Schwann cell regrowth	▲	domectic chicken	wild chicken	pituarity	*Rhof*	−0.52	10^{-3}	domestic	wild	[575]
54	*SERPINE1* (5054)	within human POAG cellular model using human trabecular meshwork cells HTM cultivated and treated with TGF-beta2 relieving POAG [356]: increased fibrillar extracellular matrix aggravating POAG along with serpin E1 deficit relatively to those at BMP7 treatment alleviating POAG	▼	according to a cohort-based biomedical study [357]: serpin E1 excess in intraocular aqueous humor can be a biomedical molecular marker for susceptibility to POAG	▲	domectic chicken	wild chicken	pituarity	*Serpina3*	−1.36	10^{-5}	domestic	wild	[575]
						domectic rabbit	wild rabbit	hypothalamus	*Serpinf1*	−2.02	10^{-2}	domestic	wild	[571]
						guinea pig	cavy	frontal cortex	*Serpinf1*	−1.88	10^{-6}	domestic	wild	[567]
55	*SIX1* (6495)	within human POAG models using transgenic mice [360]: Six1 deficiency in eye can be a biomedical molecular marker for susceptibility to POAG	▼	according to an exhaustive biomedical review [361]: SIX1 overexpression can slow down the apoptosis as the most important event in retinal ganglion cells during therapy against POAG in line with a cohort-based biomedical study [362]	▲	domectic chicken	wild chicken	pituarity	*Six1*	0.57	10^{-5}	wild	domestic	[575]
						domectic chicken	wild chicken	pituarity	*Six2*	−0.61	10^{-3}	domestic	wild	[575]
						guinea pig	cavy	frontal cortex	*Six2*	−1.39	0.05	domestic	wild	[567]
						domectic rabbit	wild rabbit	parietal-temporal cortex	*Six2*	1.65	10^{-2}	wild	domestic	[571]
56	*SIX6* (4990)	within a cohort-based biomedical gene copy number variation study of using microarrays of blood [30]: the only SIX6-deletion case happens in POAG	▼	within human neuroregeneration models using embryonic chick retinal pigmented epithelium subjected with an artificial injury [363]: SIX6 excess along with transient dedifferentiation of retinal pigmented epithelium reprogrammed to neuroepithelium, which can differentiate to neural retina cells as neuroregeneration alleviating POAG	▲	domectic chicken	wild chicken	pituarity	*Six1*	0.57	10^{-5}	wild	domestic	[575]
						domectic chicken	wild chicken	pituarity	*Six2*	−0.61	10^{-3}	domestic	wild	[575]
						guinea pig	cavy	frontal cortex	*Six2*	−1.39	0.05	domestic	wild	[567]
						domectic rabbit	wild rabbit	parietal-temporal cortex	*Six2*	1.65	10^{-2}	wild	domestic	[571]

Table S7. Cont.

#	NCBI Gene Sumbol (NCBI Gene ID)	(a) Humans: effects of changes in gene expression on the alleviation ("▲") and aggravation ("▼") of POAG (☼) [Reference]				(b) Animals: effects of changes in gene expression on the pattern of divergence between domestic animals and their wild counterparts from their most recent ancestor								
		Deficit (↓)	☼	Excess (↑)	☼	Domestic	Wild	Tissue	DEG-homolog	log_2	P_{ADG}	Deficit (↓)	Excess (↑)	[Ref]
57	*SLC4A10* (57282)	within human disease models using Slc4a10-deficient mice [364]: impaired visual function in retina that can aggravate POAG	▼	according to a comprehensive biomedical review [365]: a contrast sensitivity level decreases with a decrease Slc4a10 level within a mice model of POAG as well as a visual acuity level increases with an increase in contrast sensitivity level within a cohort-based biomedical study [366]	▲	tame rat	aggressive rat	hippocampus	*Slc4a5*	6.27	10^{-3}	wild	domestic	[46]
						guinea pig	cavy	frontal cortex	*Slc4a11*	−1.56	0.05	domestic	wild	[567]
58	*SLC23A2* (9962)	according to a cohort-based biomedical study [367]: SLC23A2 deficit in tears, aqueous humor, and blood is a biomedical molecular marker for susceptibility to POAG	▼	within human POAG cellular models using an immortalized human lens epithelial cell line HLE-B3 under exposure to the chemical oxidant tert-butylhydroperoxide [368]: an immediate SLC23A2 up-regulation improving Na+-dependent transport of vitamin C (i.e., ascorbic acid) as the most important antioxidant into the normal lens epithelium in order to prevent eye damages under oxidant stressors	▲	dog	wolf	blood	*Slc23a3*	5.01	10^{-3}	wild	domestic	[566]
59	*SPP1* (6696)	within human Alzheimer's disease models using transgenic mice carrying microglia-specific miR-155 knockdown [375]: Spp1 downregulation mitigated retinal inflammation and vasculopathy as protective effects against vascular damages during Alzheimer's disease and associated neurodegenerative ocular diseases, including POAG	▲	within a cohort-based biomedical RNA-Seq analysis of human corneoscleral tissues taken from postmortem human donor eyes with versus without glaucoma [376]: SPP1-excess can be a biomedical molecular biomarker of POAG	▼	domectic chicken	wild chicken	pituarity	*Spp2*	−0.52	10^{-2}	domestic	wild	[575]
60	*STAT3* (6774)	according to an exhaustive mini-review letter [377]: STAT3 gene silencing can contribute to both origin and development of POAG and its severity among patients with diabetes	▼	within human POAG model using rats subjected with a chronic artificially elevated intraocular pressure [378]: STAT3 overexpression in the optic nerve head seems to be a candidate molecular marker for the very earlier stages of the POAG origin and development	▼	tame fox	aggressive fox	pituitary	*Stat3*	0.31	10^{-2}	wild	domestic	[570]
61	*TAP1* (6890)	within human disease cellular models using the human melanoma cells cultured with the synthetic nanomeric peptide IT9302 homologous to the C-terminal of the human IL-10 (i.e., AYMTMKIRN) [379]: TAP1 inhibited along with a IT9302-dose-dependent increase in sensitivity to lysis by natural killer cells of anticancer innate immune response to melanoma, which is comorbid to POAG in line with a comprehensive review [380]	▲	within human diabetes mellitus models using mice [381]: Tap1 overexpression may be a candidate molecular marker for diabetes mellitus, which is comorbid with POAG, at least due to their mutual comorbidity with diabetic retinopathy [198]	▼	guinea pig	cavy	frontal cortex	*Tap1*	0.70	0.05	wild	domestic	[567]
62	*TAP2* (6891)	within human disease cellular models using the human melanoma cells cultured with the synthetic nanomeric peptide IT9302 homologous to the C-terminal of the human IL-10 (i.e., AYMTMKIRN) [379]: TAP2 inhibited along with a IT9302-dose-dependent increase in sensitivity to lysis by natural killer cells of anticancer innate immune response to melanoma, which is comorbid to POAG in line with a comprehensive review [380]	▲	within human transcriptome meta-analysis related to inflammatory bowel disease, most frequent among which are Crohn's disease and ulcerative colitis [382]: TAP2 excess along with abnormal immune response to intestinal tract microbiota that can provoke through microbiota-gut-retina axis autoimmunity impact to POAG pathogenesis [383]	▼	guinea pig	cavy	frontal cortex	*Tap1*	0.70	0.05	wild	domestic	[567]

Table S7. Cont.

#	*NCBI Gene Sumbol* (NCBI Gene ID)	(a) Humans: effects of changes in gene expression on the alleviation (“▲”) and aggravation (“▼”) of POAG (☼) [Reference]				(b) Animals: effects of changes in gene expression on the pattern of divergence between domestic animals and their wild counterparts from their most recent ancestor								
		**Deficit (↓)**	**☼**	**Excess (↑)**	**☼**	**Domestic**	**Wild**	**Tissue**	**DEG-homolog**	**log₂**	P_{ADG}	**Deficit (↓)**	**Excess (↑)**	**[Ref]**
63	*TGFB2* (7042)	within human POAG treatment models using mice subjected with the ocular eye drops containing astragaloside IV, which is a novel saponin isolated from the roots of Astragalus membranaceus as a promising medication against POAG [390]: the Tgfbr2 inhibition reduces intraocular pressure that can alleviate POAG	▲	within human POAG models using mice injected with adenoviral vector carrying an human TGFBR2 gene in addition to their own mice Tgfbr2 gene [390]: TGFBR2-excess can elevate intraocular pressure that can aggravate POAG	▼	tame fox	aggressive fox	pituitary	*Tgfb2*	0.53	10^{-2}	wild	domestic	[570]
						dog	wolf	blood	*Tgfb3*	3.31	0.05			[566]
64	*TGFBR3* (7049)	within a cohort-based biomedical RNA-Seq analysis of human Schlemm's canal endothelial cells isolated from eight different postmortem human eyes with versus without glaucoma [391]: TGFBR3-deficit can be a biomedical molecular marker of POAG	▼	within a cohort-based biomedical cellular study using orbital fibroblasts isolated from specimens obtained intraoperatively from patients with versus without thyroid-eye disease, who underwent decompressive surgery [392]: TGFBR3 excess can prevent fibrosis, which is a complication of POAG [393]	▲	domectic rabbit	wild rabbit	parietal-temporal cortex	*Tgfbr3*	1.19	10^{-2}	wild	domestic	[571]
65	*TLR2* (7097)	within human disease models using Tlr2-knockout mice [400]: susceptibility to experimental autoimmune diseases, while POAG is biomedically well-known risk-factor of autoimmune diseases [194]	▼	within human eye disease models using mice subjected with intravitreal injection of a Gram-positive spherically shaped bacterium Staphylococcus aureus [401]: Tlr2-upregulation in retinal Muller glia contributing to retinal innate defense via production of inflammatory mediators and antimicrobial peptides opposing infectious endophthalmitis following POAG filtration surgery, especially during the coronavirus disease (COVID-19) pandemic period [402]	▲	domectic chicken	wild chicken	pituarity	*Tlr1a*	0.51	10^{-4}	wild	domestic	[575]
						guinea pig	cavy	frontal cortex	*Tlr3*	1.39	0.05			[567]
66	*TLR4* (7099)	within human POAG models using Tlr4-null mice [403]: resistance to both artificially induced POAG and elevated intraocular pressure	▲	according to a retrospective review [404]: TLR4-excess can elevate both trabecular meshwork cell fibrosis and retinal ganglion cell death that can aggravate POAG	▼	domectic chicken	wild chicken	pituarity	*Tlr1a*	0.51	10^{-4}	wild	domestic	[575]
						guinea pig	cavy	frontal cortex	*Tlr3*	1.39	0.05			[567]
67	*TP53* (7157)	according to complex biomedical analysis of association between SNP rs4938723:C (i.e., ancestral allele "tttgacctatTacagctctca" => minor allele "tttgacctatCacagctctca") and POAG in Chinese population using luciferase assays, in-silicon analyses, real-time PCR, Western Blot, and flow cytometry and many other methods [411]: minor allele "C" can downregulate TP53 expression, thus leading to suppressed cell apoptosis and enhanced cell proliferation that could reduce susceptibility to POAG	▲	according to a cohort-based biomedical study in Poland [412]: TP53 upregulation can promote cell death through apoptosis during neurodegenerative processes aggravating POAG	▼	domectic rabbit	wild rabbit	parietal-temporal cortex	*Tp53*	−1.05	10^{-4}	domestic	wild	[571]
								hippocampus		−1.22	0.05			

Table S7. Cont.

#	NCBI Gene Sumbol (NCBI Gene ID)	(a) Humans: effects of changes in gene expression on the alleviation ("▲") and aggravation ("▼") of POAG (☼) [Reference]				(b) Animals: effects of changes in gene expression on the pattern of divergence between domestic animals and their wild counterparts from their most recent ancestor								
		**Deficit (↓)**	**☼**	**Excess (↑)**	**☼**	**Domestic**	**Wild**	**Tissue**	**DEG-homolog**	**log₂**	P_{ADG}	**Deficit (↓)**	**Excess (↑)**	**[Ref]**
74	*TRPM5* (29850)	within human disease models using Trpm5-deficient mice [415]: increased susceptibility to bacterial infections that can aggravate POAG according to a biomedical case report [150]	▼	according to a nutritional study of natural herbal products that can stimulate people to a healthy lifestyle [416]: bitter melon extract upregulates TRPM5 contributing to aversion to high salt concentrations that can prevent high salt provoking cardiovascular diseases, especially hypertension as a risk factor of POAG [417]	▲	guinea pig	cavy	frontal cortex	*Trpm2*	−0.61	0.05	domestic	wild	[567]
						guinea pig	cavy	frontal cortex	*Trpm3*	0.90	10^{-3}	wild	domestic	[567]
						guinea pig	cavy	frontal cortex	*Trpm8*	2.11	10^{-2}	wild	domestic	[567]
						domectic chicken	wild chicken	pituarity	*Trpm6*	0.91	10^{-5}	wild	domestic	[575]
75	*TXNRD2* (10587)	according to a comprehensive molecular biochemical review [418]: TXNRD2 deficiency can lead to an increase in mitochondrial reactive oxygen species (ROS) playing a key role in the pathogenesis of POAG [419]	▼	within human lifespan models using transgenic fruit flies overexpressing Txnrd2 verified with many independent transcriptome profiling data on long-lived primate and rodent species [420]: Txnrd2 excess is a biomedical molecular marker for susceptibility to longevity that seems to be fitting the intraocular pressure decrease with age increase in the human POAG model using transgenic mice [421]	▲	tame rat	aggressive rat	hippocampus	*Txnrd2*	−0.71	10^{-2}	domestic	wild	[46]
						tame rat	aggressive rat	midbrain tegmentum	*Txnrd2*	−0.92	10^{-5}	domestic	wild	[574]
76	*VAV2* (7410)	within human POAG models using Vav2-deficient mice [422]: early onset of iridocorneal angle changes and elevated intraocular pressure, with subsequent selective loss of retinal ganglion cells and optic nerve head cupping, which are the hallmarks of POAG	▼	within human neurodevelopment models using transgenic Vav2 gain-of-function chicken (*Gallus gallus domesticus*) [423]: Vav2 excess in the chick spinal cord along with an improved limb trajectory of motor axon growth that might contribute to both neuroprotection and neuroregeneration during POAG according to an exhaustive biomedical review [424]	▲	dog	wolf	blood	*Vav2*	2.28	10^{-2}	wild	domestic	[566]
						tame fox	aggressive fox	pituitary	*Vav3*	−0.33	0.05	domestic	wild	[570]
77	*VAV3* (10451)	within the human disease models using Vav3-knockout mice [422]: a glaucoma-like phenotype that corresponds to SNP in Japanese patients with POAG	▼	according to an exhaustive review [425]: Vav3 activation may promote granule cell survival in the cerebellum that may slow the progression of POAG accompanied by neurodegeneration throughout the whole brain, especially severe in the cerebellum, oculomotor regions, visual cortices and frontal lobe, in line with a biomedical cohort-based study using magnetic resonance imaging [426]	▲	dog	wolf	blood	*Vav2*	2.28	10^{-2}	wild	domestic	[566]
						tame fox	aggressive fox	pituitary	*Vav3*	−0.33	0.05	domestic	wild	[570]
78	*VDR* (7421)	according to an exhaustive review [427]: Vdr-knockout mice are susceptible to hypertension aggravating POAG [9]	▼	within human disease cellular models using a fast-growing mice neuroblastoma cell line Neuro2a transfected with lentiviral vector carrying another Vdr gene copy [428]: amyloid precursor protein gene expression decreases with an increase in Vdr level that can reduce regional beta-amyloid peptide accumulations, which can block axonal transport as an initial step in retinal ganglion cell degeneration aggravating POAG according to human POAG models using mice injected with microbeads to increase intraocular pressure [429]	▲	guinea pig	cavy	frontal cortex	*Vdr*	−1.82	10^{-2}	domestic	wild	[567]

Section S1. Supplementary methods for DNA sequence analysis

For each pair of the ancestral (WT) and minor (MIN) alleles of a given human SNP, two DNA sequences $S_{WT}=\{s_{WT;-90}\ldots s_{WT;i}\ldots s_{WT;-1}\}$ and $S_{MIN}=\{s_{MIN;-90}\ldots s_{MIN;i}\ldots s_{MIN;-1}\}$, each 90 bp in length, which textually represent these alleles of the corresponding promoter located immediately upstream of the human gene transcription start site (TSS, $s_{WT;0}=s_{MIN;0}$; $s_i \in \{a, c, g, t\}$) were used as inputs to our previously developed Web service SNP_TATA_Comparator run in automatic mode [44].

First, for each of these sequences $S \in \{S_{WT}, S_{MIN}\}$, the value "$-\ln(K_D(S))$" of the TATA-binding protein (TBP) binding affinity for each variant of the promoter examined was estimated using our previously developed three-step model [576], confirmed experimentally [577] and used independently in a clinical study of pulmonary tuberculosis [48]:

$$-\ln(K_D) = 10.9 - 0.2\ [\ln(K_{SLIDE}\ K_{STOP}\ K_{BEND}), \qquad \text{(S1)}$$

where 10.9 natural logarithm units (ln units) calculated *in silico* corresponds to the independently experimentally *in vivo* measured magnitude of non-specific TBP-DNA affinity (10 mM [578]); 0.2 is the stoichiometric coefficient [576]; $-\ln(K_{STOP})$ is the empirical estimate of the affinity of the TBP for the potentially best TBP-binding site [579] by use of Bucher's position-weight matrix [580] among all possible 15-bp fragments of both DNA chains of the promoter under consideration, as follows:

$$\boldsymbol{ln(K_{STOP})} = MAX\left\{\sum_{j=-1}^{13} \boldsymbol{w_{j;s_{i+j}}}\right\}, \qquad \text{(S2)}$$

where w_{js} as an element of Bucher's matrix [580], which corresponds to the case of the nucleotide s located at the *j*-th position of the DNA sequence in question; MAX(ζ) is the highest ζ-value identified.

In Eq. (1), $-\ln(K_{SLIDE})$ is the empirical estimate of TBP affinity for the promoter being examined as the TBP was sliding along DNA in the ±5-bp local environment around the potentially best TBP-binding site found [581], which is heuristically estimated as

$$-\ln(K_{SLIDE}) = \mathbf{MEAN}\ \{0.8[TA] + 3.4\mu + 35.1\}, \qquad \text{(S3)}$$

where [TA] is the weighted number of dinucleotide TA; μ is the mean minor groove width of the DNA helix [582] of the potentially best TBP-binding site considered; 0.8, 3.4, and 35.1 are the linear regression coefficients identified elsewhere [583]; MEAN(ζ) is the arithmetic mean of all observed ζ-values.

In Eq. (1), $-\ln(K_{BEND})$ is the *in silico* estimate of TBP affinity for the potentially best TBP-binding site examined, when the DNA helix of this site bends at right angles as an allosteric rearrangement [584] fixing the TBP-promoter complex [585,586]:

$$-\ln(K_{BEND}) = \mathbf{MEAN}\{0.9[TA, AA, TG, AG] + 2.5[TA, TC, TG] + 14.4\}, \qquad \text{(S4)}$$

where 0.9, 2.5, and 14.4 are the linear regression coefficients as estimated elsewhere [583].

After that, the "$-\ln[K_D]$" values (Eqs. 1-4) go together with their standard errors of the mean (SEM) calculated according to all possible nucleotide substitutions, $s_{\bullet;j} \to \xi$, at each *j*-th position within the same ±5-bp flanks around the potentially best TBP-binding site found, namely:

$$SEM(S_\bullet)=[\ (\Sigma_{1\le i\le 26}\Sigma_{\xi\ \{a,c,g,t\}}[\ln(K_D(\{s_{\bullet;i-13}\ldots\xi\ldots{\bullet}s_{\bullet;i+12}\})/K_D(\{s_{\bullet;i-13}\ldots s_{\bullet;i+j}\ldots s_{\bullet;i+12}\})^2)]/(3*26)^{1/2} \qquad \text{(S5)}$$

This yields two paired estimates, "$-\ln(K_D(S_{WT})) \pm SEM(S_{WT})$" and "$-\ln(K_D(S_{MIN}))\pm SEM(S_{MIN})$", calculated with the input sequences S_{WT} and S_{MIN} using Eqs. (1-5), which are statistically comparable by means of the Fisher's Z-test, namely:

$$Z = \mathbf{abs}\ [\ln(K_{WT;D} / K_{MIN;D})] / [SEM(S_{WT})^2 + SEM(S_{MIN})^2]^{1/2}, \qquad \text{(S6)}$$

where Z is Fisher's Z-score as input for the proper procedure within the statistical software package R [587], the output is the *p*-value of the probability rate of acceptance of the H_0-hypothesis "H_0: $K_D(S_{WT}) \neq K_D(S_{MIN})$".

In the end, the final decision is made at its statistically significant level $p < 0.05$ if and only if $Z > 1.98$:

<u>IF</u> {*INEQUALITY* "$-\ln(K_{WT;D}) > -\ln(K_{MIN;D})$" is statistically significant},

<u>THEN</u> {*DECISION* is "S_{MIN} provides an underexpression of a given gene in comparison with S_{WT} as a norm"};

<u>ELSE</u> [**<u>IF</u>** {*INEQUALITY* "$-\ln(K_{WT;D}) < -\ln(K_{MIN;D})$" is statistically significant},

<u>THEN</u> {*DECISION* is "S_{MIN} provides an overexpression of a given gene in comparison with S_{WT} as a norm"},

<u>OTHERWISE</u> {*DECISION* is "change in the expression of this gene is insignificant"}.

The column "Δ" in Table S3 shows this DECISION as "↑" (overexpression) and "↓" (underexpression).
